# Aerosol therapy in adult critically ill patients: a consensus statement regarding aerosol administration strategies during various modes of respiratory support

**DOI:** 10.1186/s13613-023-01147-4

**Published:** 2023-07-12

**Authors:** Jie Li, Kai Liu, Shan Lyu, Guoqiang Jing, Bing Dai, Rajiv Dhand, Hui-Ling Lin, Paolo Pelosi, Ariel Berlinski, Jordi Rello, Antoni Torres, Charles-Edouard Luyt, Jean-Bernard Michotte, Qin Lu, Gregory Reychler, Laurent Vecellio, Armèle Dornelas de Andrade, Jean-Jacques Rouby, James B. Fink, Stephan Ehrmann

**Affiliations:** 1 Department of Cardiopulmonary Sciences, Division of Respiratory Care, Rush University, 600 S Paulina St, Suite 765, Chicago, IL 60612 USA; 2 Department of Critical Care Medicine, Zhongshan Hospital, Fudan University, Shanghai, China; 3 Critical Care Medicine, Peking University People’s Hospital, Beijing, China; 4 Department of Critical Care Medicine, Binzhou Medical University Hospital, Binzhou, China; 5 Department of Respiratory and Critical Care Medicine, The First Affiliated Hospital of China Medical University, Shenyang, China; 6 Department of Medicine, University of Tennessee Graduate School of Medicine, Knoxville, TN USA; 7 Department of Respiratory Therapy, Chang Gung University, Taoyuan, Taiwan; 8 Anesthesiology and Critical Care, San Martino Policlinico Hospital, IRCCS for Oncology and Neurosciences, Genoa, Italy; 9 Pulmonary and Sleep Medicine Division, Department of Pediatrics, University of Arkansas for Medical Sciences, and Pediatric Aerosol Research Laboratory at Arkansas Children’s Research Institute, Little Rock, AR USA; 10 Clinical Research/Epidemiology in Pneumonia and Sepsis (CRIPS), Vall d’Hebron Institute of Research (VHIR), Barcelona, Spain; 11 Servei de Pneumologia, Hospital Clinic, University of Barcelona, IDIBAPS CIBERES, Icrea, Barcelona, Spain; 12 Médecine Intensive Réanimation, Institut de Cardiologie, Groupe Hospitalier Pitié–Salpêtrière, Assistance Publique–Hôpitaux de Paris, Sorbonne-Université, and INSERM, UMRS_1166-ICAN Institute of Cardiometabolism and Nutrition, Paris, France; 13 School of Health Sciences (HESAV), HES-SO University of Applied Sciences and Arts of Western Switzerland, Lausanne, Switzerland; 14 Department of Emergency Medicine, Second Affiliated Hospital, Zhejiang Province Clinical Research Center for Emergency and Critical Care Medicine, and Key Laboratory of the Diagnosis and Treatment of Severe Trauma and Burn of Zhejiang Province, Zhejiang University School of Medicine, Hangzhou, China; 15 Secteur de Kinésithérapie et Ergothérapie, Cliniques Universitaires Saint-Luc, Brussels, Belgium; 16 PST-A, Faculté de Médecine, Université de Tours, Tours, France; 17 Department of Physical Therapy, Universidade Federal de Pernambuco, Recife, Brazil; 18 Research Department DMU DREAM and Multidisciplinary Intensive Care Unit, Department of Anesthesiology and Critical Care, La Pitié-Salpêtrière Hospital, Sorbonne University of Paris, Paris, France; 19 Chief Science Officer, Aerogen Pharma Corp, San Mateo, CA USA; 20 CHRU Tours, Médecine Intensive Réanimation, CIC INSERM 1415, CRICS-TriggerSep F-CRIN Research Network, and INSERM, Centre d’étude des Pathologies Respiratoires, U1100, Université de Tours, Tours, France; 21 Service de Pneumologie, Cliniques Universitaires Saint-Luc, Brussels, Belgium; 22 Institut de Recherche Expérimentale et Clinique (IREC), Pôle de Pneumologie, ORL and Dermatologie, Université Catholique de Louvain, Brussels, Belgium; 23 Centro de Investigación Biomédica en Red de Enfermedades Respiratorias (CIBERES), Instituto de Salud Carlos III, Madrid, Spain; 24 Clinical Research in the ICU, Anaesthesia Department, CHU Nimes, Université de Nimes-Montpellier, Nimes, France; 25 Department of Surgical Sciences and Integrated Diagnostics, University of Genoa, Genoa, Italy

## Abstract

**Background:**

Clinical practice of aerosol delivery in conjunction with respiratory support devices for critically ill adult patients remains a topic of controversy due to the complexity of the clinical scenarios and limited clinical evidence.

**Objectives:**

To reach a consensus for guiding the clinical practice of aerosol delivery in patients receiving respiratory support (invasive and noninvasive) and identifying areas for future research.

**Methods:**

A modified Delphi method was adopted to achieve a consensus on technical aspects of aerosol delivery for adult critically ill patients receiving various forms of respiratory support, including mechanical ventilation, noninvasive ventilation, and high-flow nasal cannula. A thorough search and review of the literature were conducted, and 17 international participants with considerable research involvement and publications on aerosol therapy, comprised a multi-professional panel that evaluated the evidence, reviewed, revised, and voted on recommendations to establish this consensus.

**Results:**

We present a comprehensive document with 20 statements, reviewing the evidence, efficacy, and safety of delivering inhaled agents to adults needing respiratory support, and providing guidance for healthcare workers. Most recommendations were based on in-vitro or experimental studies (low-level evidence), emphasizing the need for randomized clinical trials. The panel reached a consensus after 3 rounds anonymous questionnaires and 2 online meetings.

**Conclusions:**

We offer a multinational expert consensus that provides guidance on the optimal aerosol delivery techniques for patients receiving respiratory support in various real-world clinical scenarios.

**Supplementary Information:**

The online version contains supplementary material available at 10.1186/s13613-023-01147-4.

## Introduction

Aerosol therapy has been broadly utilized in both inpatient and outpatient settings, due to its advantages of being non-invasive, easy-to-use, quick onset, lower dose, and with less systemic side effects than systemic administration [1]. Unlike most ambulatory patients, intensive care unit (ICU) patients often require respiratory support, including oxygen therapy (low and high flow) through a mask or nasal cannula, and ventilatory support, such as noninvasive ventilation (NIV) or invasive mechanical ventilation (MV), to help them breathe and maintain oxygenation. In most cases, to avoid the disruption of oxygen delivery and ventilation, medical aerosols need to be administered via respiratory support devices, such as high-flow nasal cannula (HFNC), NIV, and MV [2, 3]. Delivering medical aerosols inline with these devices can be challenging due to the interference of flows and positive pressure, while aerosol delivery for patients with low-flow oxygen therapy is similar to ambulatory patients. To date, no aerosol drug/device combination has been specifically approved by regulatory bodies for inline use with respiratory support devices, meaning drugs for inhalation during respiratory support are technically off-label and lacking manufacture guidance for administration. Therefore, this consensus document does not address the issue of delivering specific drugs.

Considerable research, from bench to bedside, has focused on evaluating the effectiveness of aerosol delivery via MV, NIV, and HFNC, and identifying factors that influence aerosol delivery in these settings [2–6]. Aerosol delivery effectiveness is primarily assessed by the responses in the target organ. Nebulization of bronchodilators targeted at the tracheobronchial tree can be assessed by its immediate response, such as the changes in airway resistance, intrinsic positive end-expiratory pressure, or lung compliance. However, other drugs with longer onset time, such as antibiotics and steroids, are challenging, as they require optimal techniques to reach desired levels of drug deposition in the lung parenchyma, and it may be difficult to assess the drug deposition and patient response [7, 8]. Factors that impact aerosol delivery include patient characteristics, breathing parameters, the severity of airway disease, the characteristics of aerosol devices, their integration into respiratory support devices and the interface of these devices to patients, ease of use, and patient comfort [1–4, 9, 10]. The present consensus document is focused on the technical conditions required to optimize aerosol delivery into the respiratory system.

Clinical practice of aerosol delivery in conjunction with respiratory support devices for adult ICU patients varies widely [11–15], with little consensus among clinicians and aerosol scientists. Thus, we performed a thorough search and literature review of aerosol delivery for adult ICU patients receiving various forms of respiratory support. We invited an international panel to review the evidence and make recommendations, with the aim to provide practical guidance on aerosol delivery for adult ICU patients and identify needs for future research.

## Methods

This academic work was investigator-initiated and did not receive any funding from public or private entities. A modified Delphi method was adopted to achieve a consensus on aspects of aerosol delivery for adult critically ill patients receiving various forms of respiratory support, including MV, NIV, and HFNC.

### Working group and panel

We set up a working group responsible for designing and implementing the study, including literature search, extracting and summarizing study findings, drafting and revising recommendations, communicating with panelists, summarizing the scores and comments for three rounds of review, and organizing the online meetings. Authors who had a minimum of three publications in aerosol research and H-index ≥ 10 were invited to participate in the panel, and they were tasked to evaluate the recommendation in light of available evidence, suggest missing literature, score and comment on the recommendations, and revise the manuscript. Details about participants in the panel can be found in Additional file 1: Appendix 1.

### Literature search and preliminary recommendations generation

A literature search was conducted from the PubMed, Medline, and Scopus databases between January 1, 1990, and September 1, 2021. The key literature search strategy included (aerosol* OR nebuliz* OR inhal*) AND adult AND ((mechanical ventilation) OR (noninvasive ventilation) OR (high-flow nasal cannula)). Details of the research strategy are available in Additional file 1: Appendix 2. The working group screened the studies by titles and abstracts, and reviewed the relevant full manuscripts to select the studies included in the consensus. The study findings were extracted and summarized in tables for each question, with preliminary recommendations generated based on these findings. The preliminary recommendations, along with the summary tables and references, were provided to the panelists, who were invited to input and offer relevant references if any were missing.

### Rounds and rules for voting

A modified Delphi method (applying RAND rules) was used to collate the panelists’ views in 3 rounds of voting. Details about the rounds and rules can be found in Additional file 1: Appendix 3. During the review of the recommendations, panelists were requested to assign a Likert score of 1–9 (strongly disagree to strongly agree) to each recommendation and make comments based on their evaluation of the available evidence and their expertise. After each round of voting, the working group revised the recommendations based on panelists’ feedback. The revision and a report composed of the score distributions and a summary of anonymous comments were provided to the panelists in the next round of voting, and they were invited to vote again on both the revised and the recommendations that did not reach a consensus in the previous round. Finally, panelists discussed the final recommendations and next steps for the writing process via online meetings with attendance by ≥ 50% of the panelists. Detailed reports and results are available in Additional file 1: Appendix 4–11.

Trial registration: The study was registered on the Open Science Framework with registration digital object identifier https://osf.io/j8apu.

### Level of consensus and recommendations

The perfect consensus was defined as all panelists scoring between 7 and 9 for agreement (or 1 and 3 for disagreement), while a very good consensus was defined as ≥ 80% of panelists scoring between 7–9 and 1–3 [16, 17]. Only those recommendations with perfect or very good consensus were included in the final recommendations. In contrast, recommendations that did not reach a consensus from the first three rounds and the final online meeting were withdrawn. The writing group consisted of the panel members and the working group who drafted the consensus, with circulation to the full panel for revision and approval of the final manuscript.

## Results

The literature search and review were conducted between April 1, 2021, and September 10, 2021. 25 researchers met the inclusion criteria, and 21 accepted the invitation, of whom 18 panelists completed the scoring and comments in the first round of review, and the second round of review, while 17 panelists completed the third round of review. Two online meetings were held, with attendance by 10 and 13 panelists, respectively. Among the 17 panelists, 4 (22%) were female. The median H-index of the panel was 31 (21–60), representing pulmonologists, intensivists, anesthesiologists, physiotherapists, and respiratory care practitioners from North and South America, Europe, and Asia.

**Fig. 1:**
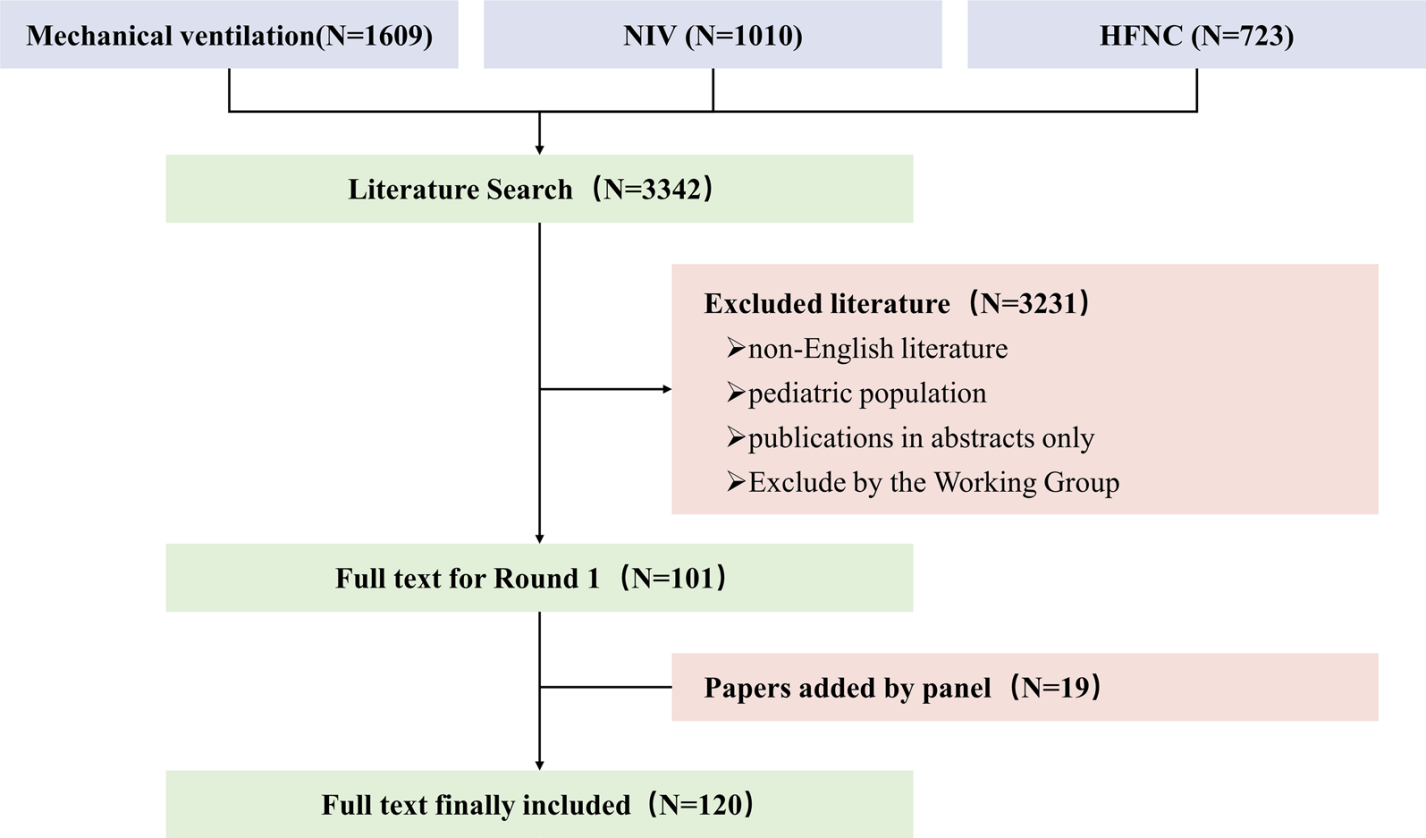
Flowchart of literature search and selection. *NIV* noninvasive ventilation, *HFNC* high-flow nasal cannula

**Fig. 2:**
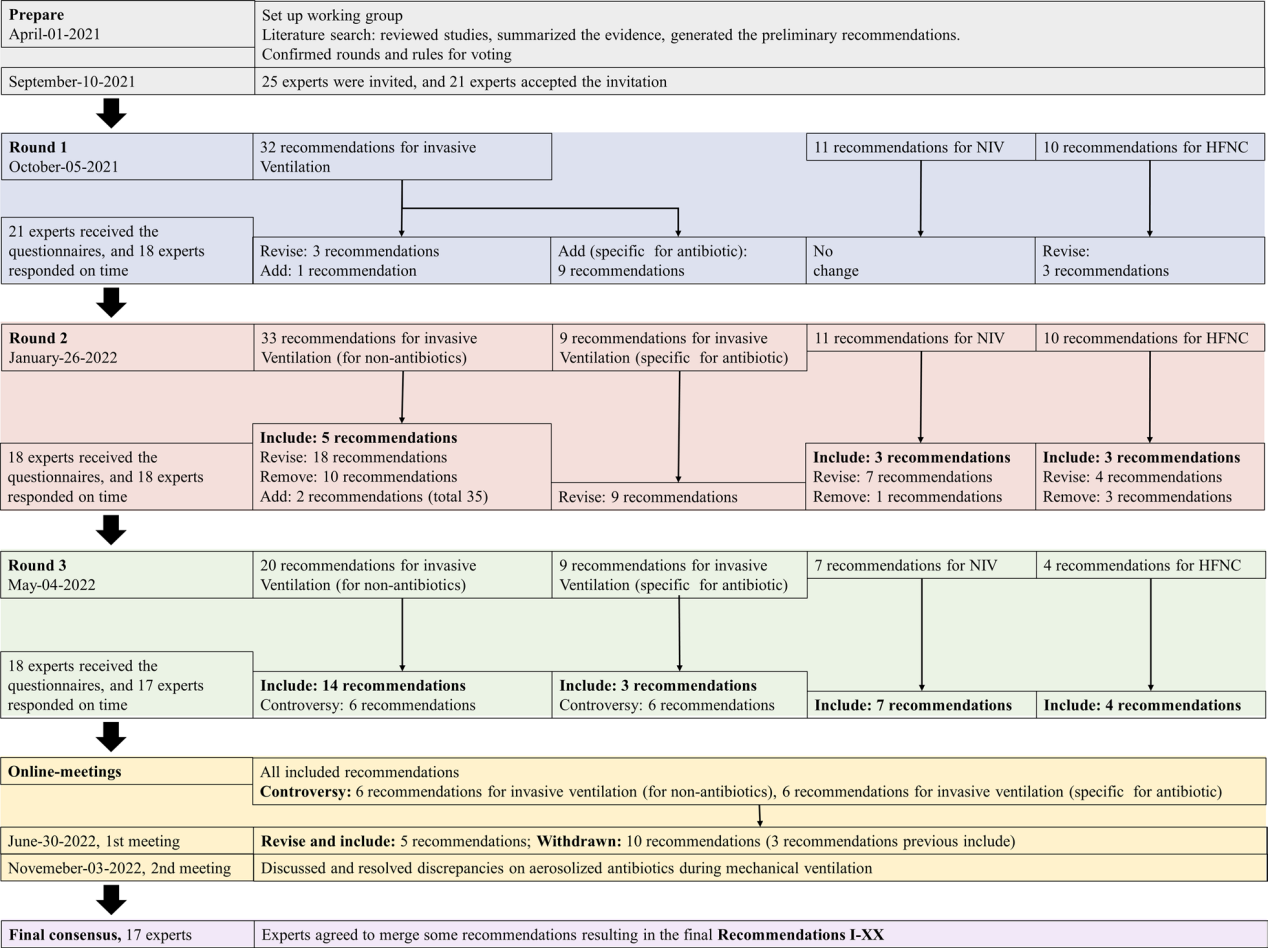
Detailed information on each round of deliberations and online meetings. *NIV* noninvasive ventilation, *HFNC* high-flow nasal cannula

**Fig. 3:**
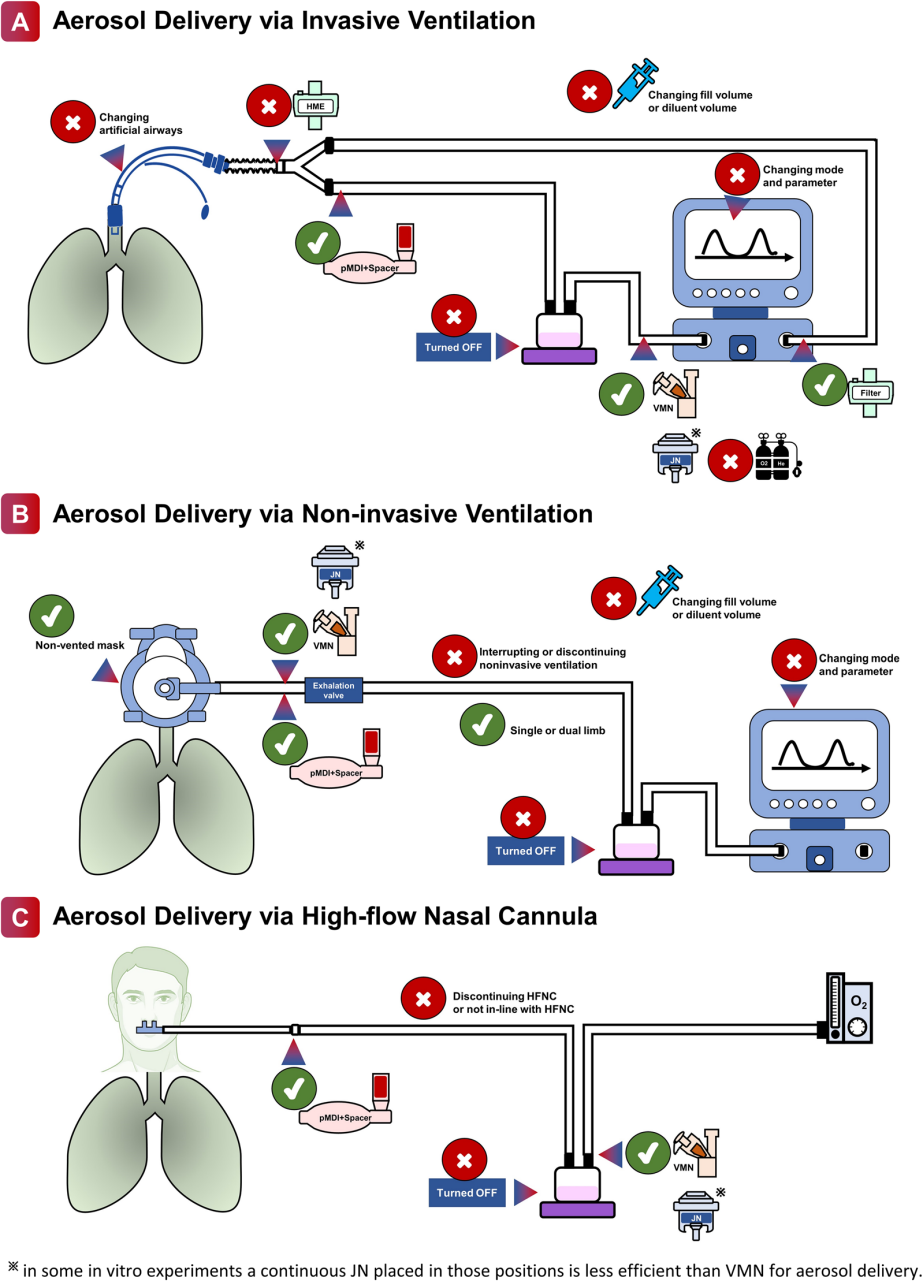
Graphic synopsis of recommendations on aerosol delivery via mechanical ventilation, noninvasive ventilation, and high-flow nasal cannula

In the literature search, 3,342 articles were screened, and 102 full texts were reviewed. After the first round of review, 18 additional relevant articles were provided by the panelists (Fig. 1). In total, 120 studies were summarized in the tables of evidence for nebulization via various forms of respiratory support (the detailed list is available in Additional file 1: Appendix 2). In the first round of review, 53 recommendations were provided to the panel. Finally, panelists agreed to merge some recommendations, culminating in recommendations I–XX, all of which reached > 80% agreement. Detailed information on each round of deliberations is available in Fig. 2 and Table 1, and the recommendations are illustrated in Fig. 3. Recommendations include indications of the source of data, including in vitro (IV), clinical studies (CS), and animal studies (AS).

**Table 1:** List of recommendations

Number	Recommendation	Round 1	Round 2	Round 3	1st and 2nd online meetings	Level of consensus and recommendation	Final results
1–1.1	During invasive ventilation, VMN is more efficient in aerosol delivery than continuous JN, with no influence on flows or fraction of inspired oxygen. VMN is preferred over continuous JN	NC	Revise	**Include**	**Include**	**Very good consensus, strong recommendation**	**Recommendation I:** During mechanical ventilation, VMN or pMDI with spacer are recommended for aerosol delivery, with no preference between the devices. The use of an inline continuous JN results in changes in tidal volume, inspiratory flow patterns, and fraction of inspired oxygen, and aerosol delivery efficiency is low, thus continuous JN is not preferred for aerosol delivery in this setting
1–1.2	During high-frequency oscillatory ventilation, VMN is more efficient in aerosol delivery than continuous JN, with no influence on flows or fraction of inspired oxygen. When available, VMN is recommended over continuous JN	NC	Withdrawn	Withdrawn	Withdrawn	NA	
1–1.3	Based on variation of the reported inhaled doses and lack of definitive clinical outcomes, there is no recommendation for pMDI and spacer versus VMN	NC	Revise	**Include**	**Include**	**Very good consensus, strong recommendation**	
1–1.4	When placed close to the ventilator, the VMN is more efficient in aerosol delivery than ultrasonic nebulizer. When nebulizer is placed at the inspiratory limb before Y-piece, the VMN is as efficient as ultrasonic nebulizer in aerosol delivery	Revise	Withdrawn	Withdrawn	Withdrawn	NA	
1–1.5	During high frequency oscillatory ventilation with nebulizer placed between Y-piece and endotracheal tube, VMN is more efficient in aerosol delivery than ultrasonic nebulizer. When available, VMN is recommended over ultrasonic nebulizer	NC	Withdrawn	Withdrawn	Withdrawn	NA	
1–1.6	When placed at the inspiratory limb before Y-piece, pMDI with a spacer is more efficient in aerosol delivery than the continuous JN, with no influence on flows or fraction of inspired oxygen. When available, pMDI with spacer actuated at beginning of inspiration is recommended over continuous JN	NC	Revise	**Include**	**Include**	**Very good consensus, strong recommendation**	
1–1.7	When placed at 12–15 cm from the Y-piece in the inspiratory limb, ultrasonic nebulizer is more efficient in aerosol delivery than the continuous JN, with no influence on flows or fraction of inspired oxygen. When available, ultrasonic nebulizer is recommended over continuous JN	NC	Withdrawn	Withdrawn	Withdrawn	NA	**Recommendation II:** When a VMN or JN is utilized during invasive ventilation with bias flow, it is recommended to place the nebulizer in the inspiratory limb, away from the Y-piece and towards the ventilator
1–1.8	When VMN is utilized during invasive ventilation with bias flow, it is recommended to be placed close to ventilator	Revise	Revise	Controversy	**Revise and include**	**Very good consensus, strong recommendation**	
1–1.9	When JN is utilized during invasive ventilation, it is recommended to be placed near the ventilator	NC	Revise	Controversy	**Revise and include**	**Very good consensus, strong recommendation**	
1–1.10	When ultrasonic nebulizer is utilized during invasive ventilation without bias flow, it is recommended to be placed at 15 cm from Y-piece at inspiratory limb; With bias flow, ultrasonic nebulizer is recommended to be placed proximal to ventilator	NC	Withdrawn	Withdrawn	Withdrawn	NA	
1–1.11	When ultrasonic nebulizer is placed at the inspiratory limb before Y-piece, adding a spacer is recommended	NC	Withdrawn	Withdrawn	Withdrawn	NA	
1–1.12	When pMDI is utilized during invasive ventilation, it is recommended to be used with a spacer with volume > 150 mL	NC	Revise	**Include**	**Include**	**Perfect consensus, strong recommendation**	**Recommendation III:** When pMDI is utilized during invasive ventilation, it is recommended to be used with a spacer with a volume > 150 mL and placed in the inspiratory limb before the Y-piece. The pMDI is recommended to be actuated at the beginning of inspiratory flow from the ventilator
1–1.13	During invasive ventilation, pMDI and spacer are recommended to be placed in the inspiratory limb before the Y-piece	NC	Revise	**Include**	**Include**	**Perfect consensus, strong recommendation**	
1–1.14	During high-frequency oscillatory ventilation, nebulizers are recommended to be placed between the Y-piece and the endotracheal tube	NC	Withdrawn	Withdrawn	Withdrawn	NA	
1–1.15	The efficiency of aerosol delivery in dry ventilator circuits is higher than that in humidified ventilator circuits. Considering the potential harms of dry gas on patient airway, and the time lapse required for a humidifier and circuits to cool down, turning off humidifier is not recommended for routine aerosol therapy	NC	Revise	Controversy	**Revise and include**	**Very good consensus, strong recommendation**	**Recommendation IV:** For patients using an active heated humidifier, turning off the humidifier is not recommended for routine aerosol therapy; for patients using a heat–moisture exchanger, removing or bypassing the heat moisture exchanger is recommended for aerosol therapy
1–1.16	When aerosol device is placed in the inspiratory limb, removing or bypassing the heat moisture exchanger is recommended	NC	**Include**	**Include**	**Include**	**Perfect consensus, strong recommendation**	
1–1.17	In ventilated patients, using a continuous JN means adding compressed gas independent of the ventilator. The effect on tidal volume, FiO_2_ etc. makes this practice unacceptable. The empirical compensations on ventilator settings may be dangerous and should be avoided. If no integrated inspiration-synchronized JN is available, the use of continuous jet neb in ventilated patients is not recommended	NC	Revise	**Include**	**Include**	**Very good consensus, strong recommendation**	**Recommendation I**
1–1.18	The influence of ventilator integrated breath-actuated JN on ventilator function and aerosol delivery efficiency varies between ventilators	NC	Withdrawn	Withdrawn	Withdrawn	NA	
1–1.19	Metered-dose inhaler should be primed, shaken, with actuation at the beginning of inspiration, with a minimum of 15 s between puffs	NC	**Include**	**Include**	**Include**	**Very good consensus, strong recommendation**	**Recommendation III**
1–1.20.1	For the jet or ultrasonic nebulizer with a residual volume > 0.5 mL, aerosol delivery efficiency is improved with a higher fill volume, but changing fill volume for the sole purpose of improving aerosol delivery efficiency is not recommended for FDA approved inhaled medication	Revise	Revise	Controversy	**Revise and include**	**Very good consensus, strong recommendation**	**Recommendation V:** When a nebulizer is utilized, changing fill volume or diluent volume for the sole purpose of improving aerosol delivery efficiency is not recommended
1–1.20.2	Increasing diluent volume in VMN to improve aerosol delivery efficiency is not recommended	Add	Revise	**Include**	**Include**	**Perfect consensus, strong recommendation**	
1–1.20.3	For viscous formulations, increasing diluent volume in VMN to improve aerosol delivery efficiency is recommended	NC	Add	Controversy	Withdrawn	NA	
1–1.21	Aerosol delivery efficiency varies between endotracheal tube and tracheotomy tube. Changing tubes for the sole purpose of improving aerosol delivery efficiency is not recommended	NC	**Include**	**Include**	**Include**	**Perfect consensus, strong recommendation**	**Recommendation VI:** It is not recommended to change the endotracheal tube or tracheostomy tube to increase the internal diameter of the airway for the sole purpose of improving aerosol delivery efficiency
1–1.22	Aerosol delivery efficiency is higher with a large size of endotracheal tube, but changing endotracheal tube for the sole purpose of improving aerosol delivery efficiency is not recommended	NC	**Include**	**Include**	**Include**	**Perfect consensus, strong recommendation**	
1–1.23	When heliox is utilized for invasive ventilation, aerosol delivery efficiency can improve. However, adding heliox for the sole purpose of improving aerosol delivery efficiency is not recommended	NC	**Include**	**Include**	**Include**	**Very good consensus, strong recommendation**	**Recommendation VII:** Adding heliox for the sole purpose of improving aerosol delivery efficiency is not recommended
1–1.24	When heliox is substituted for oxygen to drive continuous JN at the same driving flow, nebulizer output is reduced. If driving nebulizer with heliox, it is recommended to set at 15 L/min	NC	Revise	Controversy	**Revise and include**	**Very good consensus, strong recommendation**	
1–1.25	It is not recommended to change the ventilator mode for the sole purpose of improving aerosol delivery efficiency	NC	Revise	**Include**	**Include**	**Very good consensus, strong recommendation**	**Recommendations IX:** It is not recommended to change the ventilator mode and parameter settings for the sole purpose of improving aerosol delivery efficiency during routine nebulization in invasive ventilation
1–1.26	When metered-dose inhaler is utilized during invasive mechanical ventilation, there is no recommendation on flow trigger versus pressure trigger solely for aerosol delivery	NC	Withdrawn	Withdrawn	Withdrawn	NA	
1–1.27	It is not recommended to change tidal volume and respiratory rate for the sole purpose of improving aerosol delivery efficiency	NC	Revise	**Include**	**Include**	**Very good consensus, strong recommendation**	
1–1.28	Increasing inspiratory time and lowering inspiratory flows solely for aerosol delivery is not recommended	NC	Revise	**Include**	**Include**	**Very good consensus, strong recommendation**	
1–1.29	It is not recommended to change the inspiratory flow patterns solely for aerosol delivery	NC	Revise	**Include**	**Include**	**Very good consensus, strong recommendation**	
1–1.30	It is not recommended to apply end-inspiratory pause when pMDI is used during invasive mechanical ventilation	NC	Revise	**Include**	**Include**	**Very good consensus, strong recommendation**	
1–1.31	It is not recommended to change the positive end-expiratory pressure (PEEP) for the sole purpose of improving aerosol delivery efficiency	NC	Revise	**Include**	**Include**	**Very good consensus, strong recommendation**	
1–1.32	With nebulizer placed proximal to patient, higher bias flow is associated with lower aerosol delivery efficiency. With nebulizer placed proximal to ventilator, adding bias flow up to 5 L/min improves delivery. It is recommended to set bias flow up to 5 L/min when nebulizer is placed proximal to ventilator	NC	Withdrawn	Withdrawn	Withdrawn	NA	
1–1.33	Placing a filter on the expiratory limb reduces fugitive aerosols and protects the expiratory sensors. Use of an expiratory filter with frequent changes is recommended	NC	Add	**Include**	**Include**	**Very good consensus, strong recommendation**	**Recommendation VIII:** Placing a filter on the expiratory limb reduces fugitive aerosols and protects the ventilator expiratory sensors. Use of an expiratory filter with frequent changes (daily or more frequent based on aerosol administered and effect on filter resistance) is recommended
1–2.1	For antibiotics or other cost-prohibitive medications, changing to a dry circuit immediately before nebulization is recommended	Add	Revise	Controversy	Withdrawn	NA	**No consensus**
1–2.2	When delivering inhaled antibiotics for invasively ventilated patients, spontaneous breathing ventilator modes may reduce aerosol delivery efficiency, thus spontaneous breathing should be avoided and volume-controlled mode is preferred, and assessing overall benefit/risk ratio especially related to sedation	Add	Revise	Include	Withdrawn	NA	
1–2.3	When delivering inhaled antibiotics for invasively ventilated patients, it is recommended to set tidal volume of 8 mL/kg of patient’s predicted body weight, and the clinician must weigh the benefit/risk ratio of increasing tidal volume for improving aerosol delivery with the risk of high tidal volume	Add	Revise	Include	Withdrawn	NA	
1–2.4	When delivering inhaled antibiotics for invasively ventilated patients, it is recommended to keep respiratory rates at 12–15 breaths/min	Add	Revise	Controversy	Withdrawn	NA	
1–2.5	When delivering inhaled antibiotics for invasively ventilated patients, it is recommended to keep inspiratory flow below 40L/min	Add	Revise	Include	Withdrawn	NA	
1–2.6	When delivering inhaled antibiotics for invasively ventilated patients, it is recommended to use inspiratory to expiratory ratio of 50%	Add	Revise	Controversy	Withdrawn	NA	
1–2.7	When delivering inhaled antibiotics for invasively ventilated patients, it is recommended to use a constant inspiratory flow	Add	Revise	Controversy	Withdrawn	NA	
1–2.8	When delivering inhaled antibiotics for invasively ventilated patients, it is recommended to set end-inspiratory pause at 20%	Add	Revise	Controversy	Withdrawn	NA	
1–2.9	When delivering inhaled antibiotics for invasively ventilated patients, it is recommended to set a positive end-expiratory pressure (PEEP) at 5–10 cmH_2_O	Add	Revise	Controversy	Withdrawn	NA	
2.1	Placing the nebulizer inline with NIV has similar or higher aerosol delivery efficiency than using the nebulizer with a mask or mouthpiece. Interrupting or discontinuing NIV to administer aerosol via a mask or mouthpiece is unnecessary and not recommended	NC	Revise	**Include**	**Include**	**Very good consensus, strong recommendation**	**Recommendation X:** Placing the nebulizer inline with NIV has similar or higher aerosol delivery efficiency than using the nebulizer with a mask or mouthpiece. Interrupting or discontinuing NIV to administer aerosol via a mask or mouthpiece is not recommended
2.2	During NIV using single limb circuit, placing pMDI with spacer between exhalation valve and mask, with actuation at the beginning of inspiration is recommended. There is no recommendation on the placement orientation (toward or away from patient) of the spacer	NC	**Include**	**Include**	**Include**	**Very good consensus, strong recommendation**	**Recommendation XI:** During NIV placing a pressurized metered-dose inhaler with a spacer between exhalation valve and mask, with actuation at the beginning of inspiration is recommended
2.3	When placing the continuous nebulizer inline with NIV, VMN is more efficient in aerosol delivery than JN, with no influence on flows or fraction of inspired oxygen. When available, VMN is recommended over JN	NC	Revise	**Include**	**Include**	**Perfect consensus, strong recommendation**	**Recommendation XII:** During NIV using a single-limb circuit, the continuous nebulizer is recommended to be placed between the exhalation valve and the mask. When available, VMN is preferred over JN
2.4	During NIV using single limb circuit, the continuous nebulizer is recommended to be placed between the exhalation valve and the mask	NC	**Include**	**Include**	**Include**	**Very good consensus, strong recommendation**	
2.5	During NIV using a single limb circuit, with the continuous nebulizer placed between mask and exhalation valve, there is no recommendation on the type of exhalation valve	NC	Withdrawn	Withdrawn	Withdrawn	NA	
2.6	During aerosol delivery via NIV, turning off the humidifier is not recommended	NC	**Include**	**Include**	**Include**	**Very good consensus, strong recommendation**	**Recommendation XIV:** During aerosol delivery via NIV, turning off the humidifier is not recommended
2.7	The aerosol delivery efficiency is less affected by the fill volume in the VMN than the continuous JN. For continuous JNs, more dilution is associated with greater aerosol delivery. Increasing fill volume for the sole purpose to improve aerosol delivery efficiency is not recommended	NC	Revise	**Include**	**Include**	**Very good consensus, strong recommendation**	**Recommendation XV:** When a continuous nebulizer is utilized during NIV, increasing the fill volume for the sole purpose of improving aerosol delivery efficiency is not recommended
2.8	The aerosol delivery efficiency is similar between CPAP and NIV, changing the mode for the sole purpose of increasing aerosol delivery is not recommended	NC	Revise	**Include**	**Include**	**Perfect consensus, strong recommendation**	**Recommendation XVI:** During aerosol delivery via NIV, changing the mode or parameters for the sole purpose to improve aerosol delivery efficiency is not recommended
2.9	When continuous nebulizer is placed between the mask and the exhalation valve during NIV with a single limb circuit, the aerosol delivery efficiency increases as IPAP increases or EPAP decreases. Changing the parameters for the sole purpose to improve aerosol delivery efficiency is not recommended	NC	Revise	**Include**	**Include**	**Very good consensus, strong recommendation**	
2.10	When a continuous nebulizer is placed inline with NIV, the aerosol delivery efficiency is higher with a non-vented mask than a vented mask. Aerosol administration with a vented mask is not recommended	NC	Revise	**Include**	**Include**	**Perfect consensus, strong recommendation**	**Recommendation XIII:** When a continuous nebulizer is placed inline with NIV, aerosol administration with a non-vented mask is preferred over a vented mask. When a non-vented mask is used, there is no recommendation for the use of single versus dual limb circuits for aerosol delivery
2.11	When non-vented mask is used during NIV, the aerosol delivery efficiency with optimal position is similar with the single limb and dual limb circuits. There is no recommendation for the use of single versus dual limb circuits for aerosol delivery	NC	Revise	**Include**	**Include**	**Perfect consensus, strong recommendation**	
3.1	The aerosol delivery efficiency with a nebulizer via HFNC at flow ≤ 35 L/min is similar to that with a nebulizer and a mask or mouthpiece. Discontinuing HFNC treatment to administer nebulizer with a mask or mouthpiece is not recommended	Revise	Revise	**Include**	**Include**	**Very good consensus, strong recommendation**	**Recommendation XVII:** The aerosol delivery efficiency with a nebulizer via HFNC is similar to that with a nebulizer and a mask or mouthpiece. Discontinuing HFNC treatment to administer a nebulizer with a mask or mouthpiece is not recommended. Placing a nebulizer with a mask or mouthpiece with concurrent HFNC treatment should be avoided
3.2	Placing a nebulizer with a mask or mouthpiece on a patient who is using concurrent HFNC treatment is not recommended	NC	**Include**	**Include**	**Include**	**Very good consensus, strong recommendation**	
3.3	During aerosol delivery via HFNC, VMN is more efficient in aerosol delivery than JN, with no influence on flows or fraction of inspired oxygen. VMN is recommended for trans-nasal aerosol delivery	NC	**Include**	**Include**	**Include**	**Very good consensus, strong recommendation**	**Recommendation XVIII:** During aerosol delivery via HFNC, a VMN is preferred over a JN. The nebulizer is recommended to be placed at the inlet of the humidifier
3.4	Nebulizers are recommended to be placed at the inlet of humidifier at HFNC flows ≥ 10 L/min	Revise	Revise	**Include**	**Include**	**Perfect consensus, strong recommendation**	
3.5	When pMDI is placed inline with HFNC, it is recommended to be used with a spacer and placed close to nasal cannula with the aerosol plume directed toward the patient	NC	Revise	**Include**	**Include**	**Very good consensus, strong recommendation**	**Recommendation XIX:** When pMDI is placed inline with HFNC, it is recommended to be used with a spacer and placed close to the nasal cannula with the aerosol plume directed toward the patient
3.6	To optimize aerosol delivery via HFNC, gas flow is recommended to be titrated below the patient’s peak inspiratory flow if tolerated	NC	Withdrawn	Withdrawn	Withdrawn	NA	
3.7	Using heliox via HFNC for the sole purpose of improving aerosol delivery is not recommended	NC	Revise	**Include**	**Include**	**Perfect consensus, strong recommendation**	Appendix 10
3.8	Using dry gas to deliver aerosol via HFNC has been shown to improve aerosol delivery efficiency, however, considering the discomfort and the potential harms, routine use of dry gas to deliver aerosol via HFNC is not recommended	NC	**Include**	**Include**	**Include**	**Very good consensus, strong recommendation**	**Recommendation XX:** During aerosol delivery via HFNC, turning off the humidifier is not recommended
3.9	When gas flow exceeds patient inspiratory flow, open mouth breathing reduces inhaled dose. Discontinuing aerosol via HFNC to mouth breathing patients is not recommended	NC	Withdrawn	Withdrawn	Withdrawn	NA	
3.10	For trans-nasal aerosol delivery, Optiflow is preferred over Airvo2 with VMN placed at the inlet of humidifier. Aerosol delivery via Vapotherm should be avoided	Revise	Withdrawn	Withdrawn	Withdrawn	NA	

*NC* no change, *NA* not available, *pMDI* pressurized metered dose inhaler, *VMN* vibrating mesh nebulizer, *JN* jet nebulizer, *NIV* noninvasive ventilation, *CPAP *continuous positive airway pressure, *IPAP* inspiratory positive airway pressure, *EPAP* expiratory positive airway pressure, *HFNC* high-flow nasal cannula, *F*_*I*_*O*_*2*_ fraction of inspired oxygen

### Aerosol delivery via invasive mechanical ventilation

#### Selection of aerosol device

*Recommendation I* During mechanical ventilation, vibrating mesh nebulizer or pressurized metered-dose inhaler with spacer are recommended for aerosol delivery, ^IV,CS^ with no preference between the devices. ^IV,CS^ The use of an inline continuous jet nebulizer results in changes in tidal volume, inspiratory flow patterns, and fraction of inspired oxygen, and aerosol delivery efficiency is low, thus continuous jet nebulizer is not preferred for aerosol delivery in this setting.

When comparing aerosol delivery via MV, in vitro [18–26] studies reported a higher aerosol delivery efficiency with vibrating mesh nebulizer (VMN) than continuous jet nebulizer (JN), regardless of the nebulizer placement and ventilator settings. A bioavailability study in mechanically ventilated patients also showed a higher percentage of urinary salbutamol levels with VMN than continuous JN [27]. Likewise, when pressurized metered-dose inhaler (pMDI) and spacer were placed in the inspiratory limb before the Y-piece, aerosol delivery efficiency with pMDI and spacer was higher than continuous JN in the in vitro studies [19, 28, 29]. However, three clinical studies reported no significant differences in reducing airway resistance for mechanically ventilated patients when inhaling albuterol via pMDI and spacer versus continuous JN [30–32]. Moreover, one randomized controlled trial did not find significant differences in the duration of mechanical ventilation among groups with VMN, JN, and pMDI with spacer for mechanically ventilated patients with asthma [33]. Notably, continuous JN is driven by an external compressed gas, which will affect the ventilation [34], including tidal volume, inspiratory flow patterns, trigger sensitivity, and the fraction of inspired oxygen (F_I_O_2_), in contrast to no influence when VMN, ultrasonic nebulizer (USN), and pMDI with spacer are utilized via MV. Breath-enhanced JNs designed for use with MV utilize less external gas flows and may reduce the impact on ventilation [35]. However, such nebulizers are not yet commercially available. Although ventilator-integrated JN does not affect ventilation, the aerosol delivery time is 2–3 times longer than continuous nebulizers [3], without consistent increases in delivery, limiting its use in clinical practice [11, 12]. Thus, VMN, USN, and pMDI with spacer are preferred over continuous JN. However, the heat generated during the use of USN has been associated with denaturing proteins, so its use with protein-containing drug should be avoided [36, 37]. When VMN and pMDI with spacer were placed at the inspiratory limb before the Y-piece, the inhaled dose of bronchodilator was similar between the two devices, and Dubosky et al. reported no differences in the VAP incidence with the use of VMN and pMDI with a spacer in their cohort study [38]. Thus, both VMN and pMDI with spacer are preferred for aerosol delivery during MV. Notably, VMNs are typically more expensive than JNs, thus it may be more cost-effective to reserve the use of VMN for patients who require frequent aerosol treatments or medications that are costly.

#### Nebulizer placement

*Recommendation II* *When a vibrating mesh nebulizer or jet nebulizer is utilized during invasive ventilation with bias flow, it is recommended to place the nebulizer in the inspiratory limb, away from the Y-piece and toward the ventilator.* ^IV,CS^

With bias flow during MV, a higher inhaled dose is generally found with VMN placed close to the ventilator than when it is placed close to the patient [19, 26, 39–41]. However, in the absence of bias flow, the findings from two in vitro studies were contradictory [18, 41]. For continuous JN, a higher inhaled dose was found with placement close to the ventilator than close to the patient with no bias flow [18], whereas in the presence of bias flow similar inhaled dose was reported with both placements [19]. Furthermore, placing the JN close to the ventilator has the pragmatic advantage of less potential for contamination from the patient’s secretions.

#### The use of pMDI and spacer

*Recommendation III* *When pressurized metered dose inhaler is utilized during invasive ventilation, it is recommended to be used with a spacer with a volume > 150 mL *^IV,CS^ *and placed in the inspiratory limb before the Y-piece.* ^IV,CS^ *The pressurized metered dose inhaler is recommended to be actuated at the beginning of inspiratory flow from the ventilator. *^IV^

When pMDI is utilized during MV, it needs to be used with an accessory device (adapter or spacer), which varies by design and size. The inhaled dose increased as the volume of the spacer/adapter increased, with a minimum volume requirement of 150 mL [24, 29, 42–47]. Among different placements, the inhaled dose was highest with the pMDI and spacer placed in the inspiratory limb 15 cm from the Y-piece [18, 48]. pMDI needs to be actuated with the onset of inspiratory flow from the ventilator, the inhaled dose was significantly reduced if the pMDI was actuated during exhalation [24, 49]. In addition, a minimum of 15 s intervals are required between actuations (puffs) [50].

#### Humidification

*Recommendation IV* *For patients using an active heated humidifier, turning off the humidifier is not recommended for routine aerosol therapy;* ^IV,CS^ *for patients using a heat–moisture exchanger, removing or bypassing the heat moisture exchanger is recommended during aerosol delivery. *^IV^

In vitro studies [18, 42, 49–58] identified a reduction of up to 50% in aerosol delivery efficiency during MV with heated humidification, compared to dry conditions, especially when JN or pMDI was utilized. In contrast, randomized trials reported no significant differences in urinary salbutamol concentrations [27], MV duration [33], and ICU length of stay [33] in groups of patients with or without humidification. Moreover, an in vitro study reported that aerosol delivery via pMDI and spacer immediately after turning off the humidifier was not improved, compared to aerosol delivery during heated humidification [57]. It might not be realistic for clinicians to wait for the circuit to dry or replace circuits after turning off the humidifier, and there is a risk for the clinician to forget to turn the humidifier on after nebulization is completed [57]. Therefore, considering the potential harms of administering dry gas to a patient airway, especially over a duration of several hours, and the time lapse required for a humidifier and circuits to cool down and dry, turning off the humidifier is not recommended for routine aerosol therapy.

Heat–moisture exchangers (HME) are generally considered a barrier to aerosol drug delivery in ventilated patients, with high-efficiency filter HMEs reported to reduce delivered doses to < 0.5% [20]. Thus, HMEs should be removed or bypassed from the pathway between the aerosol generator and the patient’s airway during aerosol delivery. However, removing HMEs from the ventilator circuit may cause lung de-recruitment [59] and increase the infection risk for both patients and care providers [60]. An alternative is to employ HMEs designed to allow aerosol delivery (HME-ADs) by bypassing the HME during aerosol administration. One in vitro study reported a similar inhaled dose with HME-AD compared to no HME [61].

#### Fill volume or diluent volume

*Recommendation V* *When a nebulizer is utilized, changing the fill volume or diluent volume for the sole purpose of improving aerosol delivery efficiency is not recommended. *^IV,CS^

When a VMN was utilized during MV, in vitro and in vivo studies reported a similar efficiency of aerosol delivery with dilution volumes of 6 versus 12 mL [62, 63]. In contrast, aerosol delivery efficiency with USN [64, 65] and inspiration-synchronized JN [64] was higher with the fill volume of ≥ 6 mL, compared to the fill volume of 3 mL. Of note, increasing the amount of drug placed in a JN induces additional manipulations and increases duration of treatment delivery, which need to be taken into account.

#### Artificial airways

*Recommendation VI* *It is not recommended to change the endotracheal tube or tracheostomy tube to increase the internal diameter of the airway for the sole purpose of improving aerosol delivery efficiency.* ^IV^

When VMN and continuous JN were placed in line during MV [66, 67] or when a dry powder inhaler was utilized with a resuscitation bag [68], no significant differences in inhaled dose were found between the endotracheal tube and tracheostomy tube of the same size. Three in vitro studies reported no significant differences in aerosol delivery between size 7.0 and 9.0 mm airways [66, 68, 69]. Changing artificial airways imposes risks and adds to the costs of therapy for patients.

#### Heliox

*Recommendation VII Adding heliox for the sole purpose of improving aerosol delivery efficiency is not recommended.* ^IV,AS^

While there may be some interest in using heliox to enhance aerosol delivery during MV, the use of this low-density gas mixture has fallen out of favor in clinical practice and a detailed discussion about the relative merits of using heliox for aerosol delivery is beyond the scope of this review. When heliox was used to drive the mechanical ventilator, one in vitro study [53] and one animal study [70] with noninfected piglets reported a higher inhaled dose than when the ventilator was driven by air or nitrogen–oxygen; however, no significant differences in inhaled dose were reported in infected piglets [70]. The cost of using heliox offsets the marginal benefits of increased aerosol delivery reported with heliox.

#### Filter on the expiratory limb

*Recommendation VIII* *Placing a filter on the expiratory limb reduces fugitive aerosols and protects the ventilator expiratory sensors. Use of a expiratory filter with frequent changes (daily or more frequent based on aerosol administered and effect on filter resistance) is recommended.* ^CS^

During aerosol delivery via MV, most of the aerosols are emitted into the room air via the ventilator exhalation port. Those fugitive aerosols could pose a health hazard for bedside caregivers [63] and damage ventilator sensors at the exhalation port [71]. Thus, a filter should be placed at the exhalation port during aerosol delivery. High-efficiency particulate air filters are designed for this purpose and are therefore indicated, while heat and moisture exchanger filters should be avoided [72]. The resistance of the filter may increase as aerosols accumulate over time, and filters should be changed periodically.

#### Ventilator mode and parameter settings

*Recommendations IX* It is not recommended to change the ventilator mode and parameter settings for the sole purpose of improving aerosol delivery efficiency during routine nebulization in patients receiving invasive ventilation. ^IV,CS^

Reported effects of ventilator mode on aerosol delivery vary. In vitro reports of no differences in the inhaled dose with pMDI and spacer between volume control (VC) and pressure support (PS) [52], which agrees with similar bronchodilation effects after inhaling salbutamol in VC and PS modes for 10 mechanically ventilated chronic obstructive pulmonary disease (COPD) patients [73]. In contrast, Dugernier et al. reported more radiolabeled aerosols delivered to the lung with VC than PS [74].

Reported effects of ventilator parameter settings on aerosol delivery are also contradictory. In vitro studies reported the inhaled dose increased as tidal volume increased over mechanical dead space but then was stable when pMDI with spacer [52] and USN [75] were used via MV. Similarly, Mouloudi et al. [76] did not find any significant differences in bronchodilation responses between tidal volumes of 8 mL/kg and 12 mL/kg in ventilated COPD patients. When VC mode was used, compared to constant flow, in vitro studies reported that decelerating flow decreased inhaled dose when VMN was used [40], but not for pMDI with spacer [77, 78] or inspiratory synchronized JN [78]. Six in vitro studies reported an increase in inhaled dose as the inspiratory time increased [50, 52, 64, 75, 78, 79], except for pMDI with spacer via MV. Two in vitro [75, 79] and one clinical [80] studies reported no significant differences in the inhaled dose or bronchodilation responses with versus without positive end-expiratory pressure. The use of an end-inspiratory pause of 5 s among 12 COPD mechanically ventilated patients did not improve bronchodilator effects [81].

Considering the contradictory reports and, more importantly, concerns that changing parameters may cause patient–ventilator asynchrony and harm, changing the ventilator mode or parameter settings for the sole purpose of improving aerosol delivery is not recommended.

### Special considerations for antibiotics delivery via invasive mechanical ventilation

Delivering antibiotics to the infected lung parenchyma is challenging and discordant results in terms of patients’ outcomes were observed among clinical studies. High lung concentrations should theoretically be delivered to obtain a bactericidal effect in treating ventilator-associated pneumonia. Therefore, on a patient case-by-case basis, clinicians may consider changing ventilatory settings to improve drug delivery when deciding to implement such off-label therapy. No further consensus could be reached among panelists on this question which may deserve further investigations. The detailed discussions of the panel, pros and cons around several specific questions on this topic are provided in the supplementary Additional file 1: Appendix 11 (see pages 575 ~ 582).

### Aerosol delivery via noninvasive ventilation

#### Aerosol delivery via noninvasive ventilation versus conventional aerosol therapy

*Recommendation X* Placing the nebulizer inline with noninvasive ventilation has similar or higher aerosol delivery efficiency than using the nebulizer with a mask or mouthpiece. Interrupting or discontinuing noninvasive ventilation to administer aerosol via a mask or mouthpiece is not recommended. ^IV,CS^

When a JN is placed inline with NIV, two healthy volunteer studies[82, 83] and one in vitro study[84] reported a lower inhaled dose, while one healthy volunteer study reported a similar inhaled dose, compared to a JN via mask or mouthpiece. Likewise, in the study with stable asthma patients, the forced expiratory volume at the first second (FEV_1_) improvement was lower with JN via continuous positive airway pressure than with JN via mask or mouthpiece [85]. However, in three clinical studies among patients with asthma exacerbation, patient pulmonary function results were better with a JN via NIV with PS settings than JN via mask or mouthpiece [86–88].

#### The use of pMDI with spacer

*Recommendation XI* During noninvasive ventilation, placing a pressurized metered-dose inhaler with a spacer between exhalation valve and mask, with actuation at the beginning of inspiration is recommended. ^IV,CS^

In a randomized crossover study with 18 stable COPD patients, Nava et al. reported that compared to the same dose of albuterol delivery via pMDI and a spacer during spontaneous breathing, the pMDI and spacer placed in line with NIV generated similar improvement of FEV_1_ and greater improvement in forced volume capacity [89]. Notably, Branconnier et al. found a lower inhaled dose with pMDI actuated during exhalation than inhalation when pMDI was used in line with NIV [90].

#### Nebulizer placement

*Recommendation XII During noninvasive ventilation using a single-limb circuit, the continuous nebulizer is recommended to be placed between the exhalation valve and the mask. When available, vibrating mesh nebulizer is preferred over jet nebulizer.* ^IV,CS^

During NIV using a single-limb circuit with a non-vented mask, the inhaled dose with continuous nebulizers (JN and VMN) placed at the ventilator outlet was lower compared to placing the nebulizers between the exhalation valve and the mask [91–93]. During NIV using a dual-limb circuit, little evidence about comparative nebulizer placement is available, nebulizer may be placed in the inspiratory limb the same way as in a dual-limb invasive ventilation circuit [94]. When placing the continuous nebulizer inline with NIV, both in vitro [91–93, 95–99] and in vivo studies [100, 101] reported higher inhaled doses with VMN than JN, regardless of the nebulizer placement and ventilator settings. In addition, JN is driven by an external compressed gas, which may affect the tidal volume and F_I_O_2_ delivered by the ventilator, whereas these parameters are unlikely to be affected when VMN is utilized. Thus, when available, VMN should be preferred over JN for aerosol delivery in this setting.

#### Vented mask versus non-vented mask

*Recommendation XIII* *When a continuous nebulizer is placed inline with noninvasive ventilation, aerosol administration with a non-vented mask is preferred over a vented mask.* ^IV^ *When a non-vented mask is used, there is no recommendation for the use of single versus dual limb circuits for aerosol delivery. *^IV^

When a continuous nebulizer is placed inline with NIV, the aerosol delivery efficiency is higher with a non-vented mask than with a vented mask, regardless of the ventilator settings and nebulizer types [90]. One in vitro study [94] reported no significant differences in inhaled dose when the VMN was placed at the optimal placements in a single-limb noninvasive ventilator or a dual-limb critical care ventilator.

#### Humidification

*Recommendation XIV* *During aerosol delivery via noninvasive ventilation, turning off the humidifier is not recommended. *^IV,CS^

Unlike the impact of humidification on aerosol delivery via MV, both in vivo and in vitro studies reported no significant effect of humidification on aerosol delivery via NIV, regardless of nebulizer types [97, 98, 101]. This difference may be explained by the lower temperatures and humidification of the inspired gas used during NIV than MV, as it traverses the nose. Thus, there is no supporting information to turn off the humidifier during aerosol delivery via NIV. Off note, if an HME is used during NIV (pros and cons of this practice is beyond the scope of this work), it should be removed during aerosol delivery similar to recommendations during dual-limb invasive mechanical ventilation.

#### Fill volume

*Recommendation XV* When a continuous nebulizer is utilized during noninvasive ventilation, increasing the fill volume for the sole purpose of improving aerosol delivery efficiency is not recommended. ^IV,CS^

When a JN was utilized during NIV, higher aerosol delivery was reported when the fill volume was increased from 1 to 2 mL [98, 102]. However, when the fill volume was increased from 2 to 4 mL, two in vitro studies reported a small increment of the inhaled dose but a significant extension of nebulization time [97, 102]. When VMN was utilized during NIV, no significant differences were reported with different fill volumes [97, 98, 102]. Considering that the standard fill volume for most nebulization treatments is 2 mL or higher, increasing the fill volume for improving aerosol delivery is not recommended.

#### Ventilation mode and parameter settings

*Recommendation XVI* During aerosol delivery via noninvasive ventilation, changing the mode or parameters for the sole purpose to improve aerosol delivery efficiency is not recommended. ^IV,CS^

Four in vitro studies of JN during NIV reported that inhaled doses increased as pressure support settings increased [103–106]. However, in an randomized controlled trial with 36 severe asthma patients, a greater improvement in patients’ pulmonary function was found with JN via NIV with inspiratory/expiratory pressure settings of 15/10 and 15/5 cmH_2_O than JN via a mask, particularly with setting of 15/10 cmH_2_O [87]. The discrepancies might be explained by the tidal volume changes during NIV. In the in vitro settings, tidal volume increased as pressure support increased, resulting in a higher inhaled dose. When continuous positive airway pressure was used, both in vitro and in vivo studies reported no significant differences between settings. Clinically, ventilator settings need to be adjusted based on the patient’s needs and it is not recommended to change the ventilator settings for the sole purpose of improving aerosol delivery efficiency.

### Aerosol delivery via high-flow nasal cannula

#### The effectiveness of aerosol delivery via HFNC versus conventional aerosol therapy

*Recommendation XVII* The aerosol delivery efficiency with a nebulizer via high-flow nasal cannula is similar to that with a nebulizer and a mask or mouthpiece. Discontinuing high-flow nasal cannula treatment to administer a nebulizer with a mask or mouthpiece is not recommended. ^IV,CS^ Placing a nebulizer with a mask or mouthpiece with concurrent high-flow nasal cannula treatment should be avoided. ^IV^

Compared to HFNC alone, albuterol delivery via HFNC significantly improved FEV_1_ and peak expiratory flow during COPD exacerbation [107] and in stable patients with reversible airflow obstruction [108]. Compared to conventional aerosol delivery via JN with a mask or mouthpiece, placing a VMN or JN inline with HFNC generated a comparable improvement of FEV_1_ for stable COPD patients [108, 109]. For patients who require HFNC therapy, discontinuing HFNC to use a conventional nebulizer adds the risk of interrupting oxygen and positive pressure. Moreover, placing a nebulizer with a mask or mouthpiece while the patient is concurrently receiving HFNC oxygen therapy significantly reduces the inhaled dose of the aerosolized drug to a negligible level, and this practice is not recommended.

#### Selection of nebulizer: VMN versus JN

*Recommendation XVIII* During aerosol delivery via high-flow nasal cannula, a vibrating mesh nebulizer is preferred over a jet nebulizer. ^IV,CS^ The nebulizer is recommended to be placed at the inlet of the humidifier. ^IV^

During aerosol delivery via HFNC, both in vitro [110] and in vivo studies [111, 112] reported a higher efficiency of aerosol delivery with VMN than JN. Moreover, JN is driven by compressed oxygen or air, the introduction of the additional gas flow would affect flows or F_I_O_2_ delivery during HFNC treatment, whereas VMN is unlikely to influence flows or F_I_O_2_. Thus, VMN is preferred over JN. When HFNC gas flow was ≥ 10 L/min, the inhaled dose was higher with a nebulizer placed at the inlet of the humidifier compared to the nebulizer placed close to the nasal cannula [110, 113].

#### The use of pMDI and spacer

*Recommendation XIX* When pressurized metered dose inhaler is placed inline with high-flow nasal cannula, it is recommended to be used with a spacer and placed close to the nasal cannula with the aerosol plume directed toward the patient. ^IV^

When pMDI was placed inline with HFNC, the use of a spacer increased the inhaled dose by 2–5 times in comparison with no spacer, regardless of pMDI placement and HFNC flow settings [114]. The inhaled dose was higher with the spacer placed close to the nasal cannula than close to the humidifier. When the spacer was placed with the gas flow, i.e., the aerosol plume was directed toward the patient, the inhaled dose was higher than when the pMDI was actuated into the spacer with the plume directed against the direction of gas flow.

#### Humidification

*Recommendation XX* *During aerosol delivery via high-flow nasal cannula, turning off the humidifier is not recommended. *^CS^

Aerosol deposition in the lung was higher with aerosol delivery via HFNC using dry gas than heated humidified gas [115]. However, this improvement in aerosol delivery only existed with gas flow ≥ 30 L/min, which might not be tolerated by patients and might cause potential harm, such as nose bleeding.

Additional information and results from the consensus can be found in Additional file 1: Appendix 11.

## Discussion

Unlike aerosol therapy for ambulatory patients, aerosol delivery for critically ill patients, especially inline placement with various respiratory support equipment, is affected by several factors [116]. However, evidence to support the optimal aerosol delivery via respiratory support for patients is limited. In this consensus, most of the evidence is from in vitro studies, in vivo evidence especially clinical evidence on patient outcomes remains largely unknown and, in many cases, impractical. As a result, the panelist group carefully reviewed the currently available evidence and profoundly discussed the clinical benefits versus harms of applying those findings. Finally, this consensus was made with caution. Even after extensive discussion, consensus could not be reached on some topics among the panelists, such as ventilator settings and humidification for aerosolized antibiotics during MV, we provided the pros and cons of our debates for readers to review in the Additional file 1: Appendix. Clearly, more research is needed to provide firm guidelines for aerosol delivery in a variety of clinical settings encountered among critically ill patients receiving respiratory support.

Similar to other translational research, many of the in vitro findings could not be translated directly into clinical effectiveness, due in large part to the complicated mechanisms at play in the human body and the difficulty of quantifying the actual inhaled dose and the relevant clinical response. Critically ill patients, often receive multiple treatments simultaneously, making it challenging to evaluate the effects of aerosol treatments unless the aerosolized medication has a short onset and a measurable result. As such, albuterol is the most frequently used medication in clinical studies, using the rapid onset of bronchodilation effects to indirectly assess the aerosol deposition in the lung. However, due to the steep dose–response curve, a relatively small inhaled dose can cause patients to reach a plateau response, resulting in insignificant differences in clinical response between various administration settings. A more sensitive clinical measure is needed in future clinical studies. For aerosolized medications that do not have quick onset but are expensive, such as inhaled antibiotics, surfactants, gene therapy, and others, individualized dosing to reach the effective target concentration might play a key role in ensuring treatment success.

Currently, there is significant variation in the clinical practice of aerosol delivery for patients receiving respiratory support [12, 13], one size does not fit all, but the aim of this consensus statement is to clarify the numerous technical factors influencing aerosol delivery in this setting. Clinicians could use it as a reference to guide their practice based on their resources and conditions, such as the available aerosol and respiratory support devices, as well as human resources. More importantly, via this consensus statement and debates among clinical aerosol panelists (Additional file 1: Appendix 12), future directions in clinical aerosol research are suggested in Table 2.

**Table 2:** Future research direction of aerosol delivery via different respiratory support devices

	Mechanical ventilation	NIV	HFNC
Common	• Studies on short-term and long-term clinical outcomes are needed: o Evaluation on the short-term outcomes depends on the effects of aerosolized medications. For example, bronchodilation effects for bronchodilator delivery; the effects on pulmonary arterial resistance and oxygenation for pulmonary vasodilator delivery. If the aerosolized medications do not have short-onset, and short-term effects are lacking, such as inhaled steroids or antibiotics, assessments of bronchoalveolar lavage or systemic levels such as the drug concentration in the urine or blood are also needed. Sufficient intervals for wash out are needed o Long-term outcomes include the duration of respiratory support, the need for escalation of respiratory support, length of ICU stay, etc • Evaluation on the cost-effectiveness is also needed, such as the cost on the aerosol generators, aerosolized medications, respiratory support device, and healthcare providers’ time at bedside • Translational studies on new technology are needed, especially those with significant improvement in aerosol delivery found in bench studies, such as inspiration-synchronized mesh nebulizer, the utilization of soft-mist inhalers via respiratory support devices, etc.		
Specific	• The clinical effects of humidification on aerosol delivery • The effects of antibiotics on the treatment for ventilator-associated pneumonia	• Aerosol delivery via NIV with dual-limb circuits	• The clinical benefits of titrating gas flow in aerosol delivery via HFNC

*NIV* noninvasive ventilation, *HFNC* high-flow nasal cannula,* ICU* intensive care unit.

The authors of this document recognize that there are several limitations to this approach. First, although we performed a thorough search of panelists in clinical aerosol research, we might still have missed some, especially those who published aerosol research in non-English journals. Second, due to various reasons, some panelists could not participate in this consensus. Third, the invited panelists are from a limited number of countries. Although all of them have clinical backgrounds and most of them are working with medical aerosols on a daily basis, they represent a very small proportion of clinicians worldwide. Fourth, due to the lack of robust clinical evidence, we could not use more explicit assessments such as GRADE to make the recommendations, thus the level of most recommendations is low and clinicians are advised to take this into account. Finally, this consensus only evaluates evidence from the adult population, and the recommendations in this document may not apply to aerosol delivery in infants and children receiving various forms of respiratory support.

## Supplementary Information

**Additional file 1.** Appendix 1–11.

## Data Availability

Not applicable.

## Abbreviations

ICU: Intensive care unit
NIV: Noninvasive ventilation
MV: Mechanical ventilation
HFNC: High-flow nasal cannula
IV: In vitro study
CS: Clinical study
AS: Animal study
VMN: Vibrating mesh nebulizer
pMDI: Pressurized metered-dose inhaler
JN: Jet nebulizer
F_I_O_2_: Fraction of inspired oxygen
USN: Ultrasonic nebulizer
HME: Heat–moisture exchanger
VC: Volume control
PS: Pressure support
COPD: Chronic obstructive pulmonary disease
FEV_1_: Forced expiratory volume in the first second
