# Supplementary material for: Aerosol therapy in adult critically ill patients: a consensus statement regarding aerosol administration strategies during various modes of respiratory support
Source: Ann Intensive Care. 2023 Jul 12;13:63. doi: 10.1186/s13613-023-01147-4 (PMC10338422; doi:10.1186/s13613-023-01147-4)
Supplement: Supplementary file 1 — Additional file 1. Appendix 1–11. [file 13613_2023_1147_MOESM1_ESM.pdf]

Appendixes		
**Appendix 1**	Working group and voting panel	Page 2~6
**Appendix 2**	Literature search and included studies	Page 7~15
**Appendix 3**	Rounds and rules for voting	Page 16~20
**Appendix 4**	Round 1-recommendations	Page 21~171
**Appendix 5**	Report of round 1 and comments round 2	Page 172~310
**Appendix 6.0**	Comments round 3	Page 311~340
**Appendix 6.1**	Report of round 2-non-antibiotics	Page 341-445
**Appendix 6.2**	Report of round 2 -antibiotics	Page 446~464
**Appendix 7**	Report of round 3	Page 465~550
**Appendix 8**	Meeting minutes for 1st online meeting	Page 551~558
**Appendix 9**	Meeting minutes for 2nd online meeting	Page 559~567
**Appendix 10**	Additional information and results from the consensus	Page 568~574
**Appendix 11**	Pros and cons of inhaled antibiotics; published RCTs and recommendations in reviews and position papers on inhaled antibiotics	Page 575~582

Appendix 1

Working Group and Voting Panel

1. **Working group and contribution:**
    a) Jie Li, PhD, RRT, RRT-NPS, RRT-ACCS, Respiratory care program, Rush university, Chicago, IL, USA. Conceived and designed the study, organized the study group, conducted literature search and review, and generated recommendations on aerosol delivery via high-flow nasal cannula, supervised the study.
    b) James B Fink, PhD, RRT. Respiratory care program, Rush university, Chicago, IL, USA. and Aerogen Pharma Corp, San Mateo, CA, USA. Conceived the idea, designed and supervised the study.
    c) Bing Dai, MD, PhD, Department of Respiratory and Critical Care Medicine, the First Affiliated Hospital, China Medical University, Shenyang, China. Designed the study, conducted literature search and review, and generated recommendations on aerosol delivery via noninvasive ventilation, supervised the study.
    d) Kai Liu, RT. Department of Critical Care Medicine, Zhongshan Hospital, Fudan University, Shanghai, China. Designed the study, conducted literature search and review, and generated recommendations on aerosol delivery via invasive ventilation.
    e) Shan Lyu, RT, MS. Department of Critical Care Medicine, Peking University People's Hospital, Beijing, China. Designed the study, conducted literature search and review, and generated recommendations on aerosol delivery via invasive ventilation.
    f) Guoqiang Jing, RN, MS, Department of Pulmonary and Critical Care Medicine, Binzhou Medical University Hospital, Binzhou, Shandong, China. Designed the study, conducted literature search and review, and generated recommendations on aerosol delivery via invasive ventilation.

2. **The criteria of voting panel**
    a) ≥3 original research articles on aerosol therapy in acute/critical care applications as the first author or corresponding author in PubMed indexed peer-reviewed journals.

b) more than six published articles in the field of aerosol therapy, regardless of the author's position.
c) H-index≥10 sourced from Web of Science.
d) clinical experience in the management of patients with different respiratory support.

3. Information and regional distribution of voting panelists.

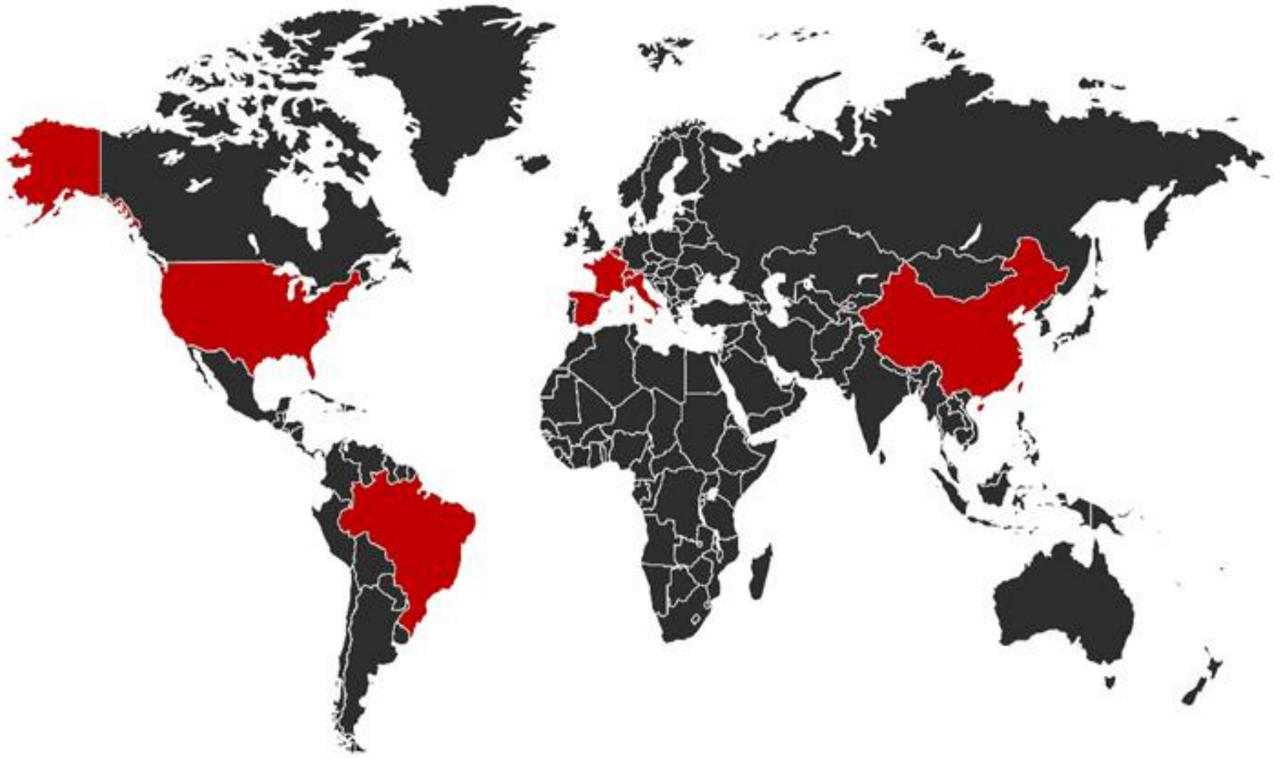

Six were physicians in pulmonary and critical care medicine, six were physicians in anesthesiology and critical care medicine, three were respiratory therapists, and two were physiotherapists, with nine from Europe, four from North America, three from Asia, and one from South America.

N	Name	Gender	Country or Region	Specialty	H-index	Round 1	Round 2	Round 3	1st online-meeting	2nd online-meeting
1	Armele Dornelas de Andrade	Female	Brazil	Respiratory Medicine	27	√	√	√	×	×
2	Antonio Torres	Male	Spain	Respiratory Medicine	122	√	√	√	√	√
3	Ariel Berlinski	Male	USA	Pulmonology, Aerosols	21	√	√	√	√	√
4	Bing Dai	Male	Mainland China	Respiratory and Critical Care Medicine	11	√	√	√	√	√
5	Charles-Edouard Luyt	Male	France	Critical Care Medicine	60	√	√	√	×	√
6	James B Fink	Male	USA	Respiratory Care	53	√	√	√	√	√
7	Huiling Lin	Female	Taiwan	Respiratory Care	17	√	√	√	√	√
8	Jie Li	Female	USA	Respiratory Care	20	√	√	√	√	√
9	Qin Lu	Female	China	Anesthesia and Critical Care Medicine	44	√	√	√	√	×
10	Jean-Bernard Michotte	Male	Switzerland	Respiratory Therapist	11	√	√	√	×	√
11	Paolo Pelosi	Male	Italy	Anesthesia and Critical Care Medicine	110	√	√	√	√	√
12	Jordi Rello	Male	Spain	Critical Care Medicine	124	√	√	√	√	√

13	Gregory Reychler	Male	Belgium	Respiratory Medicine	30	√	√	√	×	×
14	Jean-Jacques Rouby	Male	France	Anesthesia and Critical Care Medicine	70	√	√	√	×	√
15	Laurent Vecellio	Male	France	Respiratory Medicine	31	√	√	√	×	×
16	Stephan Ehrmann	Male	France	Anesthesia and Respiratory Medicine	36	√	√	√	×	√
17	Rajiv Dhand	Male	USA	Respiratory Medicine	45	√	√	√	√	√
					Total	17	17	17	10	13

H-index from Web of Science or ResearchGate or Google Scholar.

Appendix 2

Literature Search

1. Search Strategy and Selection Criteria for included studies

A search of literature was conducted from the database of PubMed, Medline, Scopus between January 1, 1990 and September 1, 2021.

Invasive ventilation

(Meter* OR MDI OR nebuli* OR "dry powder" OR aerosol* OR inhale*) AND ("mechanical ventilation" OR "invasive ventilation" OR "invasively ventilated" OR "mechanically ventilated" OR "critically ill" OR "intubat*") AND (deposit* OR deliv* OR pharmacokinetics OR scintigraphy OR BAL OR plasma OR radiolabel*)

Non-invasive ventilation

("noninvasive ventilat*" OR "noninvasive ventilat*" OR "non-invasive ventilat*" OR niv OR nippv OR nppv OR "helmet ventilation" OR "nasal ventilation" OR "mask ventilation") AND (meter* OR aerosol* OR nebuli* OR mdi OR "dry powder" OR inhale)

High-flow nasal cannula

“high-flow nasal cannul*” OR “high flow cannul*” OR “high flow oxygen therapy” OR “high flow oxygen” OR “high flow therapy” OR “HFNC” OR “trans-nasal”) AND (“aerosol” OR “nebuliz*” OR “inhal*”)

We excluded studies which have non-English literature, pediatric population, and also publications in the form of abstracts.

2. Recommendation draft

The working group screened the studies by titles and abstracts, and reviewed the full manuscripts to select the studies included in the consensus. The study findings were extracted and summarized in tables for each question, and preliminary recommendations were generated based on these findings. The preliminary recommendations, along with the summary tables and references, were provided to the voting panel members, who were invited to offer relevant references if any were missing.

3. Literature search strategy.

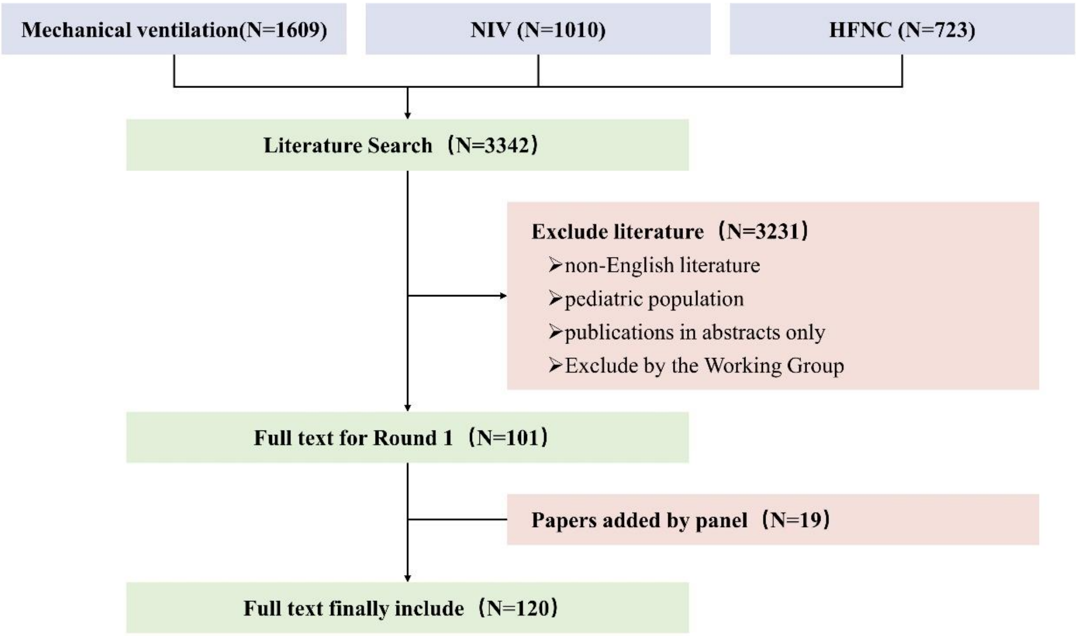

4. Summary of included studies

Number	Title	Journal	Year
1	The Effect of Nebulizer Position on Aerosolized Epoprostenol Delivery in an Adult Lung Model	Respir Care	2017
2	Evaluation of Aerosol Generator Devices at 3 Locations in Humidified and Non-humidified Circuits During Adult Mechanical Ventilation	Respir Care	2010
3	Influence of Nebulizer Type, Position, and Bias Flow on Aerosol Drug Delivery in Simulated Pediatric and Adult Lung Models During Mechanical Ventilation	Respir Care	2010
4	An In Vitro Evaluation of Aerosol Delivery Through Tracheostomy and Endotracheal Tubes Using Different Interfaces	Respir Care	2012
5	Pressurized Metered-Dose Inhalers Versus Nebulizers in the Treatment of Mechanically Ventilated Subjects With Artificial Airways: An In Vitro Study	Respir Care	2015
6	Quantifying Aerosol Delivery in Simulated Spontaneously Breathing Patients With Tracheostomy Using Different Humidification Systems With or Without Exhaled Humidity	Respir Care	2016
7	Effects of Heat and Moisture Exchangers and Exhaled Humidity on Aerosol Deposition in a Simulated Ventilator-Dependent Adult Lung Model	Respir Care	2017
8	Effect of Heat Moisture Exchanger on Aerosol Drug Delivery and Airway Resistance in Simulated Ventilator-Dependent Adults Using Jet and Mesh Nebulizers	J Aerosol Med Pulm Drug Deliv	2018
9	Nebulization associated with Bi-level noninvasive ventilation: Analysis of pulmonary radioaerosol deposition	Respir. Med.	2006
10	Effect of continuous positive airway pressure combined to nebulization on lung deposition measured by urinary excretion of amikacin	Respir. Med.	2007
11	In Vitro Performance of Spacers for Aerosol Delivery during Adult Mechanical Ventilation	J Aerosol Med Pulm	2015

		Drug Deliv	
12	Characterisation of 40 mg/ml and 100 mg/ml tobramycin formulations for aerosol therapy with adult mechanical ventilation	Pulm Pharmacol Ther	2018
13	Albuterol Delivery in a Model of Mechanical Ventilation:Comparison of Metered-Dose Inhaler and Nebulizer Efficiency	Am J Respir Crit Care Med	1995
14	Bronchodilator Therapy with Metered-Dose Inhaler and Spacer Versus Nebulizer in Mechanically Ventilated Patients: Comparison of Magnitude and Duration of Response	Respir Care	2000
15	Vibrating Mesh Nebulizer Compared With Metered-Dose Inhaler in Mechanically Ventilated Subjects	Respir Care	2017
16	Influence of Inspiratory Flow Pattern and Nebulizer Position on Aerosol Delivery with a Vibrating-Mesh Nebulizer During Invasive Mechanical Ventilation:An in Vitro Analysis	J Aerosol Med Pulm Drug Deliv	2015
17	Aerosol delivery with two ventilation modes during mechanical ventilation: a randomized study	Ann. Intensive Care	2016
18	Ventilator-Integrated Jet Nebulization Systems: Tidal Volume Control and Efficiency of Synchronization	Respir Care	2014
19	Inhaled salbutamol dose delivered by jet nebulizer, vibrating mesh nebulizer and metered dose inhaler with spacer during invasive mechanical ventilation	Pulm Pharmacol Ther	2017
20	In vitro aerodynamic characteristics of aerosol delivered from different inhalation methods in mechanical ventilation	Pharm Dev Technol	2017
21	Aerosol Delivery Using Jet Nebulizer and Vibrating Mesh Nebulizer During High Frequency Oscillatory Ventilation:An In Vitro Comparison	J Aerosol Med Pulm Drug Deliv	2016
22	Comparison of lung tissue concentrations of nebulized ceftazidime in ventilated piglets: ultrasonic versus vibrating plate nebulizers	Intensive Care Med	2008
23	Aerosol Delivery from a Metered-Dose Inhaler during Mechanical Ventilation:An in vitro Model	Am J Respir Crit Care Med	1996
24	Reconciling In Vitro and In Vivo Measurements of Aerosol Delivery from a Metered-Dose Inhaler during Mechanical Ventilation and Defining Efficiency-enhancing Factors	Am J Respir Crit Care Med	1999
25	Pressurized Aerosol versus Jet Aerosol Delivery to Mechanically Ventilated Patients	Am Rev Respir Dis	1990
26	Aerosol Delivery During Mechanical Ventilation: A Predictive In-Vitro Lung Model	J Aerosol Med	1992
27	Efficiency of Bronchodilator Aerosol Delivery to the Lungs From the Metered Dose Inhaler in Mechanically Ventilated Patients* A Study Comparing Four Different Actuator Devices	Chest	1994
28	Metered Dose Inhalers for Bronchodilator Delivery in Intubated, Mechanically Ventilated Patients	Chest	1991
29	Effect of Nebulizer Location and Spontaneous Breathing on Aerosol Delivery During Airway Pressure Release Ventilation in Bench Testing	J Aerosol Med Pulm Drug Deliv	2018
30	Improvement in Aerosol Delivery with Helium–Oxygen Mixtures during	Am J Respir	2001

	Mechanical Ventilation	Crit Care Med	
**31**	**Inhaled Fenoterol–Ipratropium Bromide in Mechanically Ventilated Patients with Chronic Obstructive Pulmonary Disease**	**Am J Respir Crit Care Med**	**1999**
32	Effects of inhaled fenoterol and positive end-expiratory pressure on the respiratory mechanics of patients with chronic obstructive pulmonary disease	Can Respir J	2005
33	Effect of a spacer on pulmonary aerosol deposition from a jet nebuliser during mechanical ventilation	Thorax	1995
34	Comparison of jet and ultrasonic nebulizer pulmonary aerosol deposition during mechanical ventilation	Eur Respir J	1997
35	In vitro evaluation of aerosol bronchodilator delivery during mechanical ventilation: pressure-control vs. volume control ventilation	Intensive Care Med	2003
36	In Vitro Evaluation of Aerosol Performance and Delivery Efficiency During Mechanical Ventilation Between Soft Mist Inhaler and Pressurized Metered-Dose Inhaler	Respir Care	2020
37	A Proof-of-Principle Setup for Delivery of Relenza(Zanamivir) Inhalation Powder to Intubated Patients	J Aerosol Med Pulm Drug Deliv	2015
38	Influence of Moisture Accumulation in Inline Spacer on Delivery of Aerosol Using Metered-Dose Inhaler During Mechanical Ventilation	Respir Care	2009
39	Size Distribution of Colistin Delivery by Different Type Nebulizers and Concentrations During Mechanical Ventilation	Pharmaceutics	2019
40	Improving Aerosol Drug Delivery During Invasive Mechanical Ventilation With Redesigned Components	Respir Care	2014
41	Influence of respiratory efforts on b2-agonist induced bronchodilation in mechanically ventilated COPD patients: A prospective clinical study	Respir. Med.	2007
**42**	**Metered-Dose Inhaler Versus Nebulized Albuterol in Mechanically Ventilated Patients**	**Am Rev Respir Dis**	**1993**
43	A Comparison of Bronchodilator Therapy Delivered by Nebulization and Metered-Dose Inhaler in Mechanically Ventilated Patients	Chest	1999
44	The Effect of Humidity on the Size of Particles Delivered from Metered-Dose Inhalers	Aerosol Science and Technology	2005
45	Factors Determining Continuous Infusion Aerosol Delivery During Mechanical Ventilation	Respir Care	2021
46	Aerosol Delivery and Modern Mechanical Ventilation: In Vitro/In Vivo Evaluation	Am J Respir Crit Care Med	2003
47	The Delivery of Chlorofluorocarbon-Propelled Versus Hydrofluoroalkane-Propelled Beclomethasone Dipropionate Aerosol to the Mechanically Ventilated Patient: A Laboratory Study	Respir Care	2003
48	Placement of the nebulizer before the humidifier during mechanical ventilation: Effect on aerosol delivery	Heart & Lung	2009
49	Bronchodilator delivery by metered-dose inhaler in mechanically ventilated COPD patients: influence of end-inspiratory pause	Eur Respir J	1998
50	Bronchodilator delivery by metered-dose inhaler in mechanically ventilated COPD patients: influence of tidal volume	Intensive Care Med	1999

51	Bronchodilator delivery by metered-dose inhaler in mechanically ventilated COPD patients: influence of flow pattern	Eur Respir J	2000
52	Lung deposition and systemic bioavailability of different aerosol devices with and without humidification in mechanically ventilated patients	Heart & Lung	2017
53	Clinical outcome associated with the use of different inhalation method with and without humidification in asthmatic mechanically ventilated patients	Pulm Pharmacol Ther	2017
54	Evaluation of Aerosol Drug Delivery Options during Adult Mechanical Ventilation in the COVID-19 Era	Pharmaceutics	2021
55	Delivery of a Nebulized Aerosol to a Lung Model during Mechanical Ventilation: Effect of Ventilator Settings and Nebulizer Type, Position, and Volume of Fi1l	Am Rev Respir Dis	1992
56	Inhaled Treprostinil Drug Delivery During Mechanical Ventilation and Spontaneous Breathing Using Two Different Nebulizers	Pediatr. Crit. Care Med	2017
57	Factors Influencing the In Vitro Deposition of Tobramycin Aerosol: A Comparison of an Ultrasonic Nebulizer and a High-Frequency Vibrating Mesh Nebulizer	J Aerosol Med	2006
58	Evaluation of a Reservoir Device for Metered-Dose Bronchodilator Delivery to Intubated Adults· An in Vitro Study	Chest	1992
59	A Comparison of Inline MDI Actuators for Delivery of a Beta Agonist and a Corticosteroid with a Mechanically Ventilated Lung Model	Respir Care	1998
60	In vitro evaluation of aerosol delivery of aztreonam lysine (AZLI): an adult mechanical ventilation model	Expert Opin. Drug Deliv	2017
61	Effect of different connection adapters on aerosol delivery in invasive ventilation setting; an in-vitro study	J Drug Deliv Sci Technol	2021
62	Delivery of Ultrasonic Nebulized Aerosols to a Lung Model during Mechanical Ventilation	Am Rev Respir Dis	1993
**63**	**Intravenous versus Nebulized Ceftazidime in Ventilated Piglets with and without Experimental Bronchopneumonia**	**Anesthesiology**	**2005**
64	In vitro study and semiempirical model for aerosol delivery control during mechanical ventilation	Intensive Care Med	2005
65	A simple in vitro method for the evaluation of an ultrasonic nebulizer for drug delivery to intubated, ventilated patients and the effect of nebulizer and ventilator settings on the uptake of fluid from the nebulizer chamber	Eur. J. Anaesthesiol.	1999
66	The Effect of Different Closed Suction Catheter Designs and pMDI Adapters on Aerosol Delivery in Simulated Adult Mechanical Ventilation With and Without Exhaled Humidity	Respir Care	2018
67	Aerosol delivery by an ultrasonic nebulizer during different mechanical ventilation settings in a lung model – a pilot study	Drug Des Devel Ther	2008
**68**	**Size distribution of salbutamol/ipratropium aerosols produced by different nebulizers in the absence and presence of heat and humidification**	**Pulm Pharmacol Ther**	**2018**
**69**	**The Clinical Practice and Best Aerosol Delivery Location in Intubated and Mechanically Ventilated Patients: A Randomized Clinical Trial**	**Biomed Res Int**	**2021**

**70**	**Influence of diluent volume of colistimethate sodium on aerosol characteristics and pharmacokinetics in ventilator-associated pneumonia caused by MDR bacteria**	**J Antimicrob Chemother**	**2018**
71	In-Vitro Characterization of the Aerosolized Dose During Non-Invasive Automatic Continuous Positive Airway Pressure Ventilation	Pulm Ther	2016
72	In-vitro/in-vivo comparison of inhaled salbutamol dose delivered by jet nebulizer, vibrating mesh nebulizer and metered dose inhaler with spacer during non-invasive ventilation	Exp. Lung Res	2017
73	Face Mask Leak Determines Aerosol Delivery in Noninvasive Ventilation	Respir Care	2020
74	Influence of Exhalation Valve and Nebulizer Position on Albuterol Delivery During Noninvasive Positive Pressure Ventilation	J Aerosol Med Pulm Drug Deliv	2014
75	Treatment of Acute Bronchospasm With β-Adrenergic Agonist Aerosols Delivered by a Nasal Bilevel Positive Airway Pressure Circuit	Ann Emerg Med	1995
76	Reversal of Bronchial Obstruction with Bi-level Positive Airway Pressure and Nebulization in Patients with Acute Asthma	J. Asthma	2009
**77**	**Nebulizer Function during Mechanical Ventilation**	**AM REV RESPIR DIS**	**1992**
**78**	**High-efficiency Delivery of Salbutamol with a Metered-dose Inhaler in Narrow Tracheal Tubes and Catheters**	**Anesthesiology**	**1991**
79	Modeling and optimization of nebulizers' performance in non-invasive ventilation using different fill volumes: Comparative study between vibrating mesh and jet nebulizers	Pulm Pharmacol Ther	2018
80	Effects Of Diluent Volume, Nebulizer Flow, and Nebulizer Brand	Chest	1996
81	In Vitro Comparison of Five Nebulizers During Noninvasive Ventilation:Analysis of Inhaled and Lost Doses	J Aerosol Med Pulm Drug Deliv	2014
82	In Vitro Comparison of a Vibrating Mesh Nebulizer Operating in Inspiratory Synchronized and Continuous Nebulization Modes During Noninvasive Ventilation	J Aerosol Med Pulm Drug Deliv	2015
83	Nebulization During Spontaneous Breathing, CPAP, and Bi-Level Positive-Pressure Ventilation: A Randomized Analysis of Pulmonary Radioaerosol Deposition	Respir Care	2014
84	Enhanced delivery of nebulised salbutamol during non-invasive ventilation	J Pharm Pharmacol	2006
85	The relative lung and systemic bioavailability of terbutaline following nebulisation in non-invasively ventilated patients	Int J Pharm	2011
86	Albuterol Delivery During Noninvasive Ventilation	Respir Care	2005
87	In-vitro characterisation of the nebulised dose during non-invasive ventilation	J Pharm Pharmacol	2010
88	Salbutamol delivery during non-invasive mechanical ventilation in patients with chronic obstructive pulmonary disease: a randomized, controlled study	Intensive Care Med	2001
89	Aerosol kinetics and bronchodilator efficacy during continuous positive airway pressure delivered by face mask	Thorax	1997
90	Comparison of response to aerosol drug delivery with mesh and jet nebulizers during non-invasive ventilation (NIV) in acute exacerbation of COPD	Eur. Respir. J	2017

91	In vitro evaluation of aerosol bronchodilator delivery during noninvasive positive pressure ventilation: Effect of ventilator settings and nebulizer position	Crit Care Med	2002
92	The impact of ventilator and circuits on Aerosol Delivery during Noninvasive Ventilation：An in-vitro Comparison between Single Limb Circuit and Dual Limb Circuit	Respir Care	2022
93	Radioaerosol Pulmonary Deposition Using Mesh and Jet Nebulizers During Noninvasive Ventilation in Healthy Subjects	Respir Care	2015
94	Noninvasive Ventilation Coupled With Nebulization During Asthma Crises: A Randomized Controlled Trial	Respir Care	2013
95	Effects of ventilator settings, nebulizer and exhalation port position on albuterol delivery during non-invasive ventilation: an in-vitro study	Pulmonary Medicine	2017
**96**	**Effects of Nebulizer Position, Gas Flow, and CPAP on Aerosol Bronchodilator Delivery: An In Vitro Study**	**Respir Care**	**2015**
**97**	**A mesh nebulizer is more effective than jet nebulizer to nebulize bronchodilators during non-invasive ventilation of subjects with COPD: A randomized controlled trial with radiolabeled aerosols**	**Respir. Med.**	**2019**
**98**	**Effects of Heat and Humidification on Aerosol Delivery during Auto-CPAP non-invasive Ventilatio**	**Arch Pulmonol Respir Care**	**2017**
**99**	**Effects of ventilator settings, nebulizer and exhalation port position on albuterol delivery during non-invasive ventilation: an in-vitro study**	**BMC Pulmonary Medicine**	**2017**
100	Impact of Gas Flow and Humidity on Trans-Nasal Aerosol Deposition via Nasal Cannula in Adults: A Randomized Cross-Over Study	Pharmaceutics	2019
101	Comparison of aerosol delivery across combinations of drug delivery interfaces with and without concurrent high-flow nasal therapy	Intensive Care Med Exp	2019
102	Oral Versus Nasal High-Flow Bronchodilator Inhalation in Chronic Obstructive Pulmonary Disease	J Aerosol Med Pulm Drug Deliv	2018
103	Aerosol Delivery Through Adult High Flow Nasal Cannula with Heliox and Oxygen	Respir Care	2017
104	Aerosol Delivery with Two Nebulizers Through High-Flow Nasal Cannula: A Randomized Cross-Over Single-Photon Emission Computed Tomography-Computed Tomography Study	J Aerosol Med Pulm Drug Deliv	2017
105	Identifying Dose Response to Trans-nasal Pulmonary Administration of Bronchodilator Aerosols via Nasal High-Flow Therapy in Adults with Stable Chronic Obstructive Pulmonary Disease and Asthma	Respiration	2019
106	The Ratio of Nasal Cannula Gas Flow to Patient Inspiratory Flow on Trans-nasal Pulmonary Aerosol Delivery for Adults: An in Vitro Study	Pharmaceutics	2019
107	In vitro comparison between inspiration synchronized and continuous vibrating mesh nebulizer during trans-nasal aerosol delivery	Intensive Care Med Exp	2020
108	Aerosol Delivery Through an Adult High-Flow Nasal Cannula Circuit Using Low-Flow Oxygen	Respir Care	2019
109	Investigation of Fugitive Aerosols Released into the Environment during High-Flow Therapy	Pharmaceutics	2019

110	Aerosol Therapy in Adults Receiving High Flow Nasal Cannula Oxygen Therapy	J Aerosol Med Pulm Drug Deliv	2016
111	Nasal high-flow bronchodilator nebulization: a randomized cross-over study	Ann. Intensive Care	2018
112	Pressurized Metered Dose Inhaler Aerosol Delivery Within Nasal High-Flow Circuits: A Bench Study	J Aerosol Med Pulm Drug Deliv	2021
113	The Impact of High-Flow Nasal Cannula Device, Nebulizer Type, and Placement on Trans-Nasal Aerosol Drug Delivery: An In Vitro Study	Respir Care	2021
**114**	**Bronchodilator Delivery via High-Flow Nasal Cannula: A Randomized Controlled Trial to Compare the Effects of Gas Flows**	**Pharmaceutics**	**2021**
**115**	**β Agonist Delivery by High-Flow Nasal Cannula During COPD Exacerbation: A Prospective Physiological Study**	**Respir Care**	**2021**
**116**	**The Clinical Impact of Flow Titration on Epoprostenol Delivery via High Flow Nasal Cannula for ICU Patients with Pulmonary Hypertension or Right Ventricular Dysfunction: A Retrospective Cohort Comparison Study**	**J Clin Med**	**2020**
**117**	**Mitigating Fugitive Aerosols during Aerosol Delivery via High-Flow Nasal Cannula Devices**	**Respir Care**	**2021**
**118**	**Position of different nebulizer types for aerosol delivery in an adult model of mechanical ventilation.**	**Front Med (Lausanne)**	**2022**
**119**	**Pharmacokinetic characteristics of nebulized colistimethate sodium using two different types of nebulizers in critically ill patients with ventilator-associated respiratory infections**	**Antibiotics (Basel)**	**2022**
**120**	**A hybrid in vitro in silico framework for albuterol delivery through an adult ventilator circuit to a patient-specific lung airway model**	**J Aerosol Science**	**2021**

Bolded: newly added

Appendix 3

Rounds and Rules for Voting

A modified Delphi method (applying RAND rules) used to collate the voting panelists' views in 5 rounds of voting. The first 3 rounds are anonymous questionnaires, and the last round is 2 rounds online meeting.

1. Round

In the three rounds of review of the recommendations, voting panelists were requested to assign a Likert score of 1-9 (strongly disagree to strongly agree) to each recommendation and make comments based on their evaluation of the available evidence and their expertise. In the second and third rounds of review, the working group revised the recommendations based on voting panelists' feedback. A revision with track change was provided to the voting panelists, who were invited to score and comment on those revised or controversial recommendations, which was defined as less than 80% of voting panelists providing scores of 7-9 or 1-3. Simultaneously the distribution of scores and a summary of anonymous comments from the previous round were also provided to the voting panelists. Finally, voting panelists were invited to discuss the final recommendations via online meeting. Before the meeting, a summary report of the scores and anonymous comments were provided to voting panelists. For voting panelists who were unavailable to attend the meeting, the final revision and discussion in the online meeting were sent to them via email for their comments or approval.

2. Voting panelists involved in the process at different stages

- Prepare: 25 voting panelists were invited, and 21 voting panelists accepted the invitation.
- Round 1: 21 voting panelists received the questionnaires, and 18 voting panelists responded on time.
- Round 2: 18 voting panelists received the questionnaires, and 18 voting panelists responded on time.
- Round 3： 18 voting panelists received the questionnaires, and 17 voting panelists responded on time.
- 1st online discussion: 10 voting panelists participated in the online meeting.

- 2nd online discussion: 13 voting panelists participated in the online meeting.

3. Definition

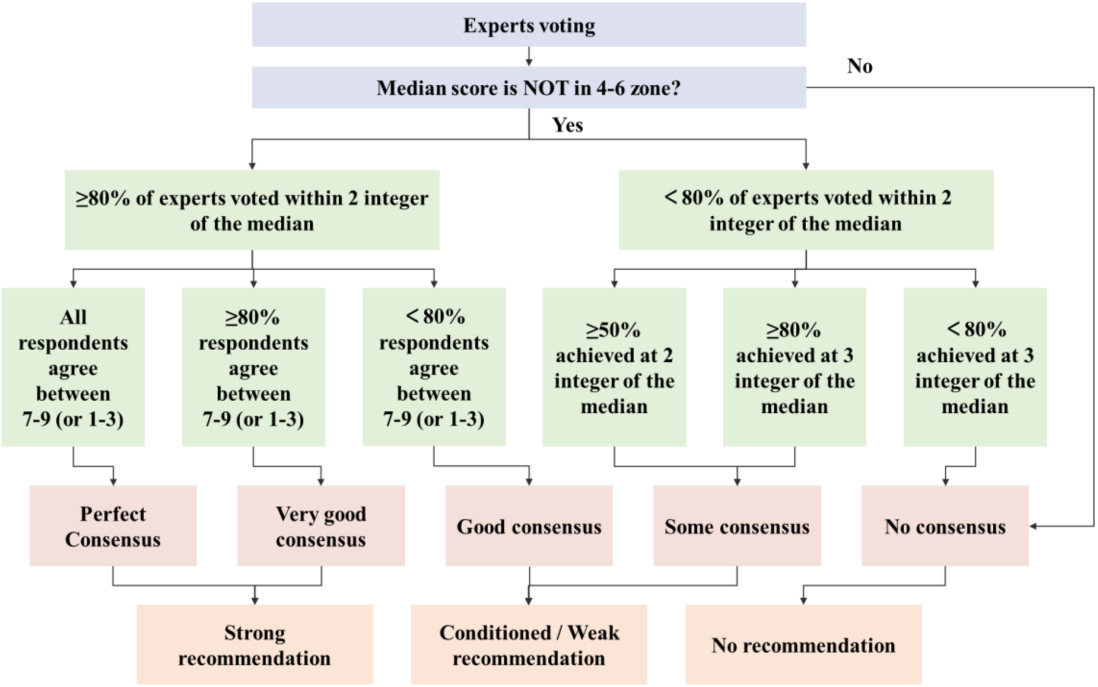

Scale:1-9, 1= strongly disagree, 9 = strongly agree

Zone: 1-3 disagree zone, 4-6 uncertain zone，7-9：agree zone

Level of consensus

- **Perfect consensus:** 100% of voting panelists on one number between 7-9(or 1-3).
- **Very good consensus:** ≥80% of voting panelists are within 2 integer of the median and ≥80% respondents agree between 7-9(or 1-3) (e.g., median is 8, 80% voting panelists are from 7 to 9).
- **Good consensus:** ≥80% of voting panelists are within 2 integer of the median and ＜80% respondents agree between 7-9(or 1-3) (e.g., median is 7, 80% of voting panelists are from 5 to 9，50% of voting panelists are from 7 to 9)
- **Some consensus:** ≥50% voting panelists are within 2 integers of the median (e.g., median is 7, 50% of voting panelists are from 5 to 9) or ≥80% of voting panelists are within 3 integers of the median (e.g., median is 7, 80% of voting panelists are from 4 to 9)
- **No consensus:** All other responses. (e.g., median is 5 or median is 7, more than 20% of voting panelists are from 1 to 3)

Level of recommendation

➢ **Strong recommendation**

- Median is not in 4-6 zone, so it is either in the zone 7-9 or zone 1-3
- **AND** the degree of consensus is at least very good

"Strongly agree" if median score is 7-9, "Strongly disagree" if median score is 1-3.

➢ **Conditional/Weak recommendation**

- Median is not in 4-6 zone, so it is either in the zone 7-9 or zone 1-3
- **AND** the degree of consensus is "good or some consensus"

"Weakly agree" if median score is 7-9, "Weakly disagree" if median score is 1-3.

➢ **No recommendation**

- Median is in 4-6 zone
- **OR** less than 80% of voting panelists are within 3 integers of the median

4. Deal with results in each round.

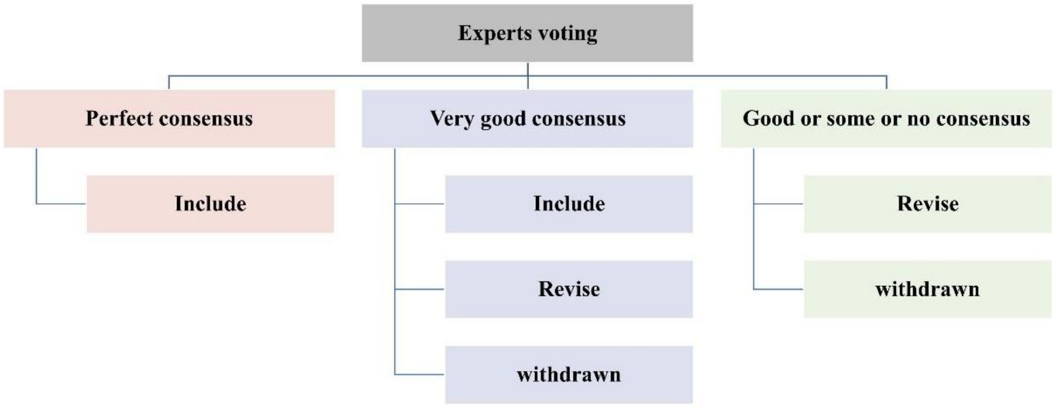

Based on voting and comments, deal with result in each round:

➢ Perfect consensus: include.

➢ Very good consensus: include, revise, withdrawn.

➢ Good or some or no consensus：revise, withdrawn.

Recommendations with perfect or very good consensus were included in the final recommendations. In contrast, those recommendations with fewer than 80% of voting panelists scoring 7-9 or 1-3 in the first three rounds and the final online meeting were withdrawn.

5. Results in each round.

In the first round of review, 53 recommendations were provided to the voting panel.

Due to the perceived differences between inhaled antibiotics and non-antibiotics among mechanically ventilated patients, voting panelists suggested separating the recommendations of nebulization via mechanical ventilation into aerosol administration of antibiotics and non-antibiotics. Thus 63 recommendations were provided for voting panelists in the second review, 11 reached the perfect or very good consensus and 14 were withdrawn after the second round of review. Of the 40 recommendations provided in the third round of review, 28 reached the perfect or very good consensus. Finally, all recommendations were discussed in the 1st and 2nd online meeting, five reached a perfect or very good consensus, 10 recommendations (3 recommendation previous include) been withdrawn. Since some of the content is relevant, voting panelists agreed to merge them into one recommendation. Therefore, **recommendation I-XX** is eventually formed.

Appendix 4

Section 1

Aerosol Delivery via Invasive Ventilation for Adult Patients

Recommendations 1.1

Voting for recommendations 1.1

**Recommendations 1.1**	During invasive ventilation, vibrating mesh nebulizer is more efficient in aerosol delivery than continuous jet nebulizer, with no influence on flows or fraction of inspired oxygen. When available, vibrating mesh nebulizer is preferred over continuous jet nebulizer.
**Evidence**	in vitro[1-6,9], in vivo[7,8]
	If you have additional evidence, please provide references:
**Likert score of 1-9**	□1 □2 □3 □4 □5 □6 □7 □8 □9<br>1= absolutely disagree, 9 = absolutely agree
**Comments**	
**Suggestions for future research**	

Table 1.1. Studies compared aerosol delivery via VMN and continuous jet nebulizer in invasive ventilated patients

Author, year	Study type	Population	Ventilator Setting	Heated-Humidity	Bias flow(L/min)	Inhaled dose with nebulizer placed proximal to ventilator			Inhaled dose with nebulizer placed at inspiratory limb before Y-piece			Distance before Y piece
						VMN	Continuous JN	p	VMN	Continuous JN	p	
Ari, Areabi 2010[1]	In vitro		Vt 500ml, PEEP 5, f 15	Y	0	8.4±2.1%	6.0±0.1%	NS	16.8±2.6%	3.6±0.2%	<0.002	15cm
				N		24.2±1.2%	14.7±1.5%	0.001	30.2±1.0%	9.7±1.5%	<0.002	
Ari, Atalay, 2010[2]	In vitro		Vt 500ml, PEEP 5, f 20	Y	2	23.8±1.0%	5.2±0.2%	<0.05	13.4±1.1%	4.7±0.1%	<0.05	
					5	21.4±0.4%	4.7±0.4%	<0.05	9.7±0.6%	4.0±0.1%	<0.05	
EIHansy, Boules, El Essawy, 2017[3]	Ex-vivo	COPD	PSV targeted Vt 500ml, PEEP 5	N	NR				52.5±13.4%	20.4±4.3%	<0.001	NR
	In vivo								10.6±1.7%	4.2±0.9%	<0.001	
EIHansy, Boules, Farid, 2017[4]	In vitro		Vt 500ml, PEEP 5, f 15	N	NR	30.5±5.9%	17.6±3.8%	<0.05				NR
Ari, 2018[5]	In vitro		Vt 500ml, PEEP 5, f 15	Y plus using exhaled humidity model	NR				10.6±0.5%	5.4±0.2%	<0.05	Immediately
Liu, 2019[6]	In vitro		Vt 500ml, PEEP 5, f 20	Y	2	34.4±9.5%	12.7±2.1%	<0.001				NR
Moustafa, Ali, 2017[7]	In vivo	Asthma or bronchospastic COPD exacerbation	NR	Y	NR	1.7±0.8%	0.7±0.5%	0.001				NR
				N		1.8±1.1%	0.9±0.6%	0.021				NR
									**Days of invasive ventilation**			

									**VMN**	**Continuous JN**	**p**	
Moustafa, ElHansy, 2017[8]	In vivo	Asthma acute exacerbation	Vt 500ml, PEEP 5, f 12	Y	NR				5.8±1.0	6.0±1.5	NS	Inspiratory limb of circuit proximal to patient
				N					5.6±1.0	5.4±1.0	NS	
						**Inlet of humidifier**	**Inspiratory limb before Y-piece immediately**		**Between HME and ETT**			
						**VMN**	**Continuous JN**	**p**	**VMN**	**Continuous JN**	**p**	
Naughton, 2021[9]	In vitro		Vt 500ml, f 15	Y	NR	25.32±2.48 %	11.54±1.76 %	<0.0001				
				N					26.99±1.99 %	9.88±1.21 %	<0.0001	
			Vt 400ml, f 20	Y		22.61±2.65 %	5.24±0.76 %	<0.0001				

VMN, vibrating mesh nebulizer; JN, jet nebulizer; PSV, pressure support ventilation; Vt, tidal volume; f, breathing frequency; PEEP, positive end expiratory pressure; NR, not reported; NS, no significant; Y, yes; N, no; COPD, chronic pulmonary obstructive disease; HME, heat moisture exchanger; ETT, endotracheal tube.

1. Ari A, Areabi H, Fink JB. Evaluation of aerosol generator devices at 3 locations in humidified and non-humidified circuits during adult mechanical ventilation. Respir Care. 2010;55(7):837-844.
2. Ari A, Atalay OT, Harwood R, Sheard MM, Aljamhan EA, Fink JB. Influence of nebulizer type, position, and bias flow on aerosol drug delivery in simulated pediatric and adult lung models during mechanical ventilation. Respir Care. 2010;55(7):845-851.
3. ElHansy MHE, Boules ME, El Essawy AFM, et al. Inhaled salbutamol dose delivered by jet nebulizer, vibrating mesh nebulizer and metered dose inhaler with spacer during invasive mechanical ventilation. Pulm Pharmacol Ther. 2017;45:159-163.
4. ElHansy MHE, Boules ME, Farid H, et al. In vitro aerodynamic characteristics of aerosol delivered from different inhalation methods in mechanical ventilation. Pharm Dev Technol. 2017;22(6):844-849.
5. Ari A, Dang T, Al Enazi FH, et al. Effect of Heat Moisture Exchanger on Aerosol Drug Delivery and Airway Resistance in Simulated Ventilator-Dependent Adults Using Jet and Mesh Nebulizers. J Aerosol Med Pulm Drug Deliv. 2018;31(1):42-48.
6. Liu CY, Ko HK, Fink JB, et al. Size Distribution of Colistin Delivery by Different Type Nebulizers and Concentrations During Mechanical Ventilation. Pharmaceutics. 2019;11(9).

7. Moustafa IOF, Ali MRA, Al Hallag M, et al. Lung deposition and systemic bioavailability of different aerosol devices with and without humidification in mechanically ventilated patients. Heart Lung. 2017;46(6):464-467.
8. Moustafa IOF, ElHansy MHE, Al Hallag M, et al. Clinical outcome associated with the use of different inhalation method with and without humidification in asthmatic mechanically ventilated patients. Pulm Pharmacol Ther. 2017;45:40-46.
9. Naughton PJ, Joyce M, Mac Giolla Eain M, O'Sullivan A, MacLoughlin R. Evaluation of Aerosol Drug Delivery Options during Adult Mechanical Ventilation in the COVID-19 Era. Pharmaceutics. 2021;13(10):1574.

Recommendations 1.2

Voting for recommendations 1.2

**Recommendations 1.2**	During high-frequency oscillatory ventilation, vibrating mesh nebulizer is more efficient in aerosol delivery than continuous jet nebulizer, with no influence on flows or fraction of inspired oxygen. When available, vibrating mesh nebulizer is recommended over continuous jet nebulizer.	
**Evidence**	In vitro[1]	
	If you have additional evidence, please provide references:	
**Likert score of 1-9**	□1 □2 □3 □4 □5 □6 □7 □8 □9<br>1= absolutely disagree, 9 = absolutely agree	
**Comments**		
**Suggestions for future research**		

Table 1.2. The study compared aerosol delivery via VMN and continuous jet nebulizer in HFOV patients

Author, year	Study type	Ventilator Setting	Heated-Humidity	Inhaled dose with nebulizer placed proximal to ventilator			Inhaled dose with nebulizer placed between the circuit and the endotracheal tube		
				VMN	Continuous JN	p	VMN	Continuous JN	p
Fang, 2016[1]	In vitro	MAP 30 cmH_2O, bias flow 40L/min, F 5 Hz, Power 8 cmH_2O, Ti% 33%	Y	26.0±14.0 μg	0.15±7.6 μg	0.164	1139.3±279.2 μg	144.6±53.4 μg	0.01

VMN, vibrating mesh nebulizer; JN, jet nebulizer; HFOV, high frequency oscillatory ventilation; MAP, mean airway pressure; F, frequency; Ti %, (inspiratory time/ respiratory cycle) %.

1. Fang TP, Lin HL, Chiu SH, et al. Aerosol Delivery Using Jet Nebulizer and Vibrating Mesh Nebulizer During High Frequency Oscillatory Ventilation: An In Vitro Comparison. J Aerosol Med Pulm Drug Deliv. 2016;29(5):447-453.

Recommendations 1.3

Voting for recommendations 1.3

**Recommendations 1.3**	Based on variation of the reported inhaled doses and lack of definitive clinical outcomes, there is no recommendation for metered dose inhaler and spacer versus vibrating mesh nebulizer.
**Evidence**	In vitro[1-3,5], in vivo[2,4,6,7]
	If you have additional evidence, please provide references:
**Likert score of 1-9**	□1 □2 □3 □4 □5 □6 □7 □8 □9<br>1= absolutely disagree, 9 = absolutely agree
**Comments**	
**Suggestions for future research**	

Table 1.3.1 Studies compared aerosol delivery via VMN and MDI in invasive ventilated patients

**Author,**	**Study**	**Populati**	**Ventilator**	**Heated-**	**Bias**	**Inhaled dose with nebulizer placed**	**Inhaled dose with nebulizer placed at**

year	type	on	Setting	Humidity	flow (L/min)	proximal to ventilator			inspiratory limb before Y-piece			
						**VMN**	**MDI with spacer**	**p**	**VMN**	**MDI with spacer**	**p**	**Distance before Y piece**
Ari, Areabi 2010[1]	In vitro		Vt 500ml, PEEP 5, f 15	Y	0	8.4±2.1%	2.5±0.8%	0.01	16.8±2.6%	17.0±1.0%	NS	15cm
				N		24.2±1.2%	7.9±1.5%	0.001	30.2±1.0%	27.8±3.3%	NS	
EIHansy, Boules, El Essawy, 2017[2]	In vivo	COPD	PSV targeted Vt 500ml, PEEP 5	N	NR				10.6±1.7%	34.8±7.5%	<0.001	NR
	Ex-vivo								52.5±13.4%	65.2±9.4%	NS	
EIHansy, Boules, Farid, 2017[3]	In vitro		Vt 500ml, PEEP 5, f 15	N	NR	26.9±3.4%	69.4±5.6%	<0.05				
Moustafa, Ali, 2017[4]	In vivo	Asthma or COPD exacerbation	NR	Y	NR	1.7±0.8%	2.0±1.1%	<0.05				
				N		1.8±1.1%	2.5±1.3%	<0.05				
						**Neb placed at the inlet of humidifier**	**Neb placed at inspiratory limb immediately before Y-piece**		**Neb placed between HME and ETT**			
						**VMN**	**MDI with spacer**	**p**	**VMN**	**MDI with spacer**	**p**	
Naughton, 2021[5]	In vitro		Vt 500ml, f 15	Y	NR	25.32±2.48 %	31.15±4.67 %	NR				
				N					26.99±1.99 %	20.00±1.12 %	NR	
			Vt 400ml, f 20	Y		22.61±2.65 %	33.62±1.90 %	NR				

Table 1.3.2 In-vivo studies compared clinical outcomes using VMN and MDI for invasively ventilated patients

Author, year	Study type	Population	Ventilator Setting	Heated-Humidity	Bias flow (L/min)	Ventilator-acquired pneumonia			Days of invasive ventilation			Device position
						VMN	MDI	p	VMN	MDI	p	
Moustafa, ElHansy, 2017[6]	In vivo	Asthma exacerbation	Vt500ml, PEEP 5, f 12	Y	NR	NR			5.8±1.0	6.2±1.1	NS	The inspiratory limb of the circuit proximal to the patient
				N					5.6±1.0	5.7±1.4	NS	
Dubosky, 2017[7]	In vivo	Invasived subjects received treatments with MDI or VMN	NR	NR	NR	9/180	3/48	0.72	6 (4-10)	5 (3-8.5)	0.14	NR

VMN, vibrating mesh nebulizer; MDI, metered dose inhaler; NR, not reported; N, no; PSV, pressure support ventilation; PEEP, positive end expiratory pressure; Y, yes; COPD, chronic pulmonary obstructive disease; HME, heat moisture exchanger; ETT, endotracheal tube.

1. Ari A, Areabi H, Fink JB. Evaluation of aerosol generator devices at 3 locations in humidified and non-humidified circuits during adult mechanical ventilation. Respir Care. 2010;55(7):837-844.
2. ElHansy MHE, Boules ME, El Essawy AFM, et al. Inhaled salbutamol dose delivered by jet nebulizer, vibrating mesh nebulizer and metered dose inhaler with spacer during invasive mechanical ventilation. Pulm Pharmacol Ther. 2017;45:159-163.
3. ElHansy MHE, Boules ME, Farid H, et al. In vitro aerodynamic characteristics of aerosol delivered from different inhalation methods in mechanical ventilation. Pharm Dev Technol. 2017;22(6):844-849.

4. Moustafa IOF, Ali MRA, Al Hallag M, et al. Lung deposition and systemic bioavailability of different aerosol devices with and without humidification in mechanically ventilated patients. Heart Lung. 2017;46(6):464-467.
5. Naughton PJ, Joyce M, Mac Giolla Eain M, O'Sullivan A, MacLoughlin R. Evaluation of Aerosol Drug Delivery Options during Adult Mechanical Ventilation in the COVID-19 Era. Pharmaceutics. 2021;13(10):1574.
6. Moustafa IOF, ElHansy MHE, Al Hallag M, et al. Clinical outcome associated with the use of different inhalation method with and without humidification in asthmatic mechanically ventilated patients. Pulm Pharmacol Ther. 2017;45:40-46.
7. Dubosky MN, Chen YF, Henriksen ME, Vines DL. Vibrating Mesh Nebulizer Compared With Metered-Dose Inhaler in Mechanically Ventilated Subjects. Respir Care. 2017;62(4):391-395.

Recommendations 1.4

Voting for recommendations 1.4

**Recommendations 1.4**	When placed close to the ventilator, the vibrating mesh nebulizer is more efficient in aerosol delivery than ultrasonic nebulizer. When nebulizer is placed at the inspiratory limb before Y-piece, there is no recommendation for vibrating mesh nebulizer versus ultrasonic nebulizer.
**Evidence**	In vitro[1-3], animal research[4]<br>If you have additional evidence, please provide references:
**Likert score of 1-9**	□1 □2 □3 □4 □5 □6 □7 □8 □9<br>1= absolutely disagree, 9 = absolutely agree
**Comments**	
**Suggestions for future research**	

Table 1.4. Studies compared aerosol delivery via VMN and USN in invasive ventilated patients

Author, year	Study	Ventilator Setting	Heated-	Bias	Inhaled dose with nebulizer placed	Inhaled dose with nebulizer placed at

type			Humidity	flow (L/min)	proximal to ventilator			inspiratory limb before Y-piece			
					VMN	USN	p	VMN	USN	p	Distance before Y piece
Ari, Areabi 2010[1]	In vitro	Vt 500ml, PEEP 5, f 15	Y	0	8.4±2.1%	6.0±0.1%	NS	16.8±2.6%	16.5±4.3%	NS	15cm
			N		24.2±1.2%	10.5±0.3	0.01	30.2±1.0%	24.7±4.4%	NS	
Pedersen, 2006[2]	In vitro	MV 5L/min, f 20	Y	NR				9.88±1.42%	12.3±2.47%	0.215	Immediately
Parker, 2017[3]	In vitro	Vt 560ml, PEEP 5, f 15	Y	2	61.5±2.3μg	50.1±4.5μg	<0.05	33.3±4.6 μg	30.1±3.2 μg	0.39	Immediately
		HFOV, 3100B, Amplitude 66, MAP 30, F 6 HZ, bias flow 30 L/min, Ti % 33						47.5±5.5μg	22.7±4.1μg	<0.05	Between Y piece and ETT
Ferrari, 2008[4]	animal study	Vt 300ml, PEEP 5, f 15	N	NR				66±4%	62±5%	NS	USN positioned 40 cm from the Y piece and VMN positioned 15 cm from the Y piece

VMN, vibrating mesh nebulizer; USN, ultrasonic nebulizer; Vt, tidal volume; MAP, mean airway pressure; N, no; PEEP, positive end expiratory pressure; Y, yes; ETT, endotracheal tube.

1. Ari A, Areabi H, Fink JB. Evaluation of aerosol generator devices at 3 locations in humidified and non-humidified circuits during adult mechanical ventilation. Respir Care. 2010;55(7):837-844.
2. Pedersen KM, Handlos VN, Heslet L, Kristensen HG. Factors influencing the in vitro deposition of tobramycin aerosol: a comparison of an ultrasonic nebulizer and a high-frequency vibrating mesh nebulizer. J Aerosol Med. 2006;19(2):175-183.
3. Parker DK, Shen S, Zheng J, et al. Inhaled Treprostinil Drug Delivery During Mechanical Ventilation and Spontaneous Breathing Using Two Different Nebulizers. Pediatr Crit Care Med. 2017;18(6):e253-e260.
4. Ferrari F, Liu ZH, Lu Q, et al. Comparison of lung tissue concentrations of nebulized ceftazidime in ventilated piglets: ultrasonic versus vibrating plate nebulizers. Intensive Care Med. 2008;34(9):1718-1723.

Recommendations 1.5

Voting for recommendations 1.5

**Recommendations 1.5**	During high frequency oscillatory ventilation with nebulizer placed between Y-piece and endotracheal tube, vibrating mesh nebulizer is more efficient in aerosol delivery than ultrasonic nebulizer. When available, vibrating mesh nebulizer is recommended over ultrasonic nebulizer.
**Evidence**	In vitro[1]
	If you have additional evidence, please provide references:
**Likert score of 1-9**	□1 □2 □3 □4 □5 □6 □7 □8 □9<br>1= absolutely disagree, 9 = absolutely agree
**Comments**	
**Suggestions for future research**	

Table 1.5. The Study compared aerosol delivery via VMN and USN in HFOV patients

Author, year	Study type	Ventilator Setting	Heated-Humidity	Between Y piece and ETT / Inhaled dose		
				VMN	USN	p
Parker, 2017	In vitro	HFOV, 3100B, Amplitude 66, MAP 30, F 6 HZ, bias flow 30 L/min, Ti % 33	Yes	47.5±5.5μg	22.7±4.1μg	<0.05

VMN, vibrating mesh nebulizer; USN, ultrasonic nebulizer; Vt, tidal volume; HFOV, high frequency oscillatory ventilation; MAP, mean airway pressure N, no; PEEP, positive end expiratory pressure; Y, yes; ETT, endotracheal tube.

1. Parker DK, Shen S, Zheng J, et al. Inhaled Treprostinil Drug Delivery During Mechanical Ventilation and Spontaneous Breathing Using Two Different Nebulizers. Pediatr Crit Care Med. 2017;18(6): e253-e260.

Recommendations 1.6

Voting for recommendations 1.6

**Recommendations 1.6**	When placed at the inspiratory limb before Y-piece, metered dose inhaler with a spacer is more efficient in aerosol delivery than the continuous jet nebulizer, with no influence on flows or fraction of inspired oxygen. When available, metered dose inhaler with spacer is recommended over continuous jet nebulizer.
**Evidence**	In vitro[1,2,5], in vivo[3,4,6,7]
	If you have additional evidence, please provide references:
**Likert score of 1-9**	□1 □2 □3 □4 □5 □6 □7 □8 □9<br>1= absolutely disagree, 9 = absolutely agree
**Comments**	
**Suggestions for future research**	

Table 1.6. Studies compared aerosol delivery via MDI with spacer and continuous jet nebulizer in invasive ventilated patients

Author, year	Study type	Population	Ventilator Setting	Heated-Humidity	Inhaled dose with nebulizer placed at inspiratory limb before Y-piece			Distance before Y piece		
					**MDI+spacer**	**Continuous JN**	**p**			
Ari, 2015[1]	In vitro		Vt 450ml, f 20, Tracheostomy tube	Y	14.7±0.1%	3.9±0.5%	0.001	15cm		
			Endotracheal tube		11.6±1.9%	3.2±0.1%	0.002			
Fuller, 1990[2]	In vivo	Mechanically ventilated patients received inhaled bronchodilator	Vt 450-800ml,	NR	5.65±1.09(SEM)%	1.22±0.35(SEM)%	<0.001	MDI 15cm<br>Continuous JN 65cm		
Marik, 1999[3]	In vivo	Mechanically ventilated patients who had not received a bronchodilator in the previous 48 h	Vt >500ml, Ti/Ttotal 30%	NR	38%	16%	0.02	MDI before<br>Continuous JN 30cm		
					**MDI+spacer**	**Inspiratory synchronized JN**	**p**			
Diot, 1995[4]	In vitro		Vt 1000ml, f 20	N	25.1±3.7%	42.0±2.6%	NR	MDI 20cm<br>Inspiratory synchronized JN 30cm		
					**Inhaled dose with nebulizer placed at inspiratory limb before Y-piece**			**Inhaled dose with nebulizer placed at between HME and ETT**		
					**MDI+spacer**	**Continuous JN**	**p**	**MDI+spacer**	**Continuous JN**	**p**
Naughton, 2021[5]	In vitro		Vt 500ml, f 15	Y	31.15±4.67 %	11.54±1.76 %	NR			

				N				20.00±1.12 %	9.88±1.21%	NR
			Vt 400ml, f 20	Y	33.62±1.90 %	5.24±0.76 %	NR			
					**Before Y-piece / Airway resistance improvement**			**Distance before Y piece**		
					**MDI+spacer**	**Continuous JN**	**p**			
Duarte, 2000[6]	In vivo	COPD	Vt 630, f 12, Ti/Ttot 0.13	Y	18.3±1.8%	13.7±2.6%	NR	MDI 10cm<br>Continuous JN 30cm		
					**Between Y piece and ETT / The average improvement in flow PMEAN**					
					**MDI+spacer**	**Continuous JN**	**p**			
Gay, 1991[7]	In vivo	Ventilator-dependent patients, in whom were ordered bronchodilators for suspected airways obstruction	NR	NR	0.10±0.12 L/s	0.10±0.09 L/s	>0.8			

MDI, metered dose inhaler; JN, jet nebulizer; NS, no significant; N, no; Y, yes; NR, not reported; ETT, endotracheal tube; SEM, standard error of mean; Flow PMEAN, flows at recoil pressures of mean; HME, heat moisture exchanger.

1. Ari A, Harwood RJ, Sheard MM, Fink JB. Pressurized Metered-Dose Inhalers Versus Nebulizers in the Treatment of Mechanically Ventilated Subjects with Artificial Airways: An In Vitro Study. Respir Care. 2015;60(11):1570-1574.

2. Fuller HD, Dolovich MB, Posmituck G, Pack WW, Newhouse MT. Pressurized aerosol versus jet aerosol delivery to mechanically ventilated patients. Comparison of dose to the lungs. Am Rev Respir Dis. 1990;141(2):440-444.
3. Marik P, Hogan J, Krikorian J. A comparison of bronchodilator therapy delivered by nebulization and metered-dose inhaler in mechanically ventilated patients. Chest. 1999;115(6):1653-1657.
4. Diot P, Morra L, Smaldone GC. Albuterol delivery in a model of mechanical ventilation. Comparison of metered-dose inhaler and nebulizer efficiency. Am J Respir Crit Care Med. 1995;152(4 Pt 1):1391-1394.
5. Naughton PJ, Joyce M, Mac Giolla Eain M, O'Sullivan A, MacLoughlin R. Evaluation of Aerosol Drug Delivery Options during Adult Mechanical Ventilation in the COVID-19 Era. Pharmaceutics. 2021;13(10):1574.
6. Duarte AG, Momii K, Bidani A. Bronchodilator therapy with metered-dose inhaler and spacer versus nebulizer in mechanically ventilated patients: comparison of magnitude and duration of response. Respir Care. 2000;45(7):817-823.
7. Gay PC, Patel HG, Nelson SB, Gilles B, Hubmayr RD. Metered dose inhalers for bronchodilator delivery in intubated, mechanically ventilated patients. Chest. 1991;99(1):66-71.

Recommendations 1.7

Voting for recommendations 1.7

**Recommendations 1.7**	When placed at 12-15cm from the Y-piece in the inspiratory limb, ultrasonic nebulizer is more efficient in aerosol delivery than the continuous jet nebulizer, with no influence on flows or fraction of inspired oxygen. When available, ultrasonic nebulizer is recommended over continuous jet nebulizer.
**Evidence**	In vitro [1,2], in vivo [3]
	If you have additional evidence, please provide references:
**Likert score of 1-9**	□1 □2 □3 □4 □5 □6 □7 □8 □9<br>1= absolutely disagree, 9 = absolutely agree
**Comments**	
**Suggestions for future research**	

Table 1.7. Studies compared aerosol delivery via USN and jet nebulizer in invasive ventilated patients

Author, year	Study type	Population	Ventilator Setting	Heated-Humidity	Bias flow (L/min)	Ultrasonic nebulizer brand	Inhaled dose with nebulizer placed proximal to ventilator			Inhaled dose with nebulizer placed at inspiratory limb before Y-piece			Distance before Y piece
							USN	Continuous JN	p	USN	Continuous JN	p	
Ari, Areabi 2010[1]	In vitro		Vt 500ml, PEEP 5, f 15	Y	0		4.6±2.0%	6.0±0.1%	NS	16.5±4.3%	3.6±0.2%	<0.002	15cm
				N			10.5±0.3%	14.7±1.5%	0.03	24.7±4.4%	9.7±1.5%	<0.002	
O'Doherty, 1992[2]	In vitro		MV 6-15L/min, f 10-20, Ti% 20-50%	Y	NR	Fisoneb				14.8±1.5(SEM)%	10.3±1.3(SEM)%	NR	Immediately + Storage chamber
										4.2±0.4(SEM)%	8.0±0.94(SEM)%	NR	Immediately
						Samsonic				6.8±0.8(SEM)%	10.3±1.3(SEM)%	NR	Immediately + Storage chamber
										3.3±0.11(SEM)%	8.0±0.94(SEM)%	NR	Immediately
Harvey, 1997[3]	In vivo	Patients with open heart surgery	MV 8.1L/min, f 15.2, Ti% 24%	Y	NR					5.3±1.4%	2.3±0.9%	<0.002	12cm

USN, ultrasonic nebulizer; JN, jet nebulizer; NR, not reported; N, no; NS, no significant; PEEP, positive end expiratory pressure; Y, yes; SEM, standard error of mean; MV, minute ventilation.

1. Ari A, Areabi H, Fink JB. Evaluation of aerosol generator devices at 3 locations in humidified and non-humidified circuits during adult mechanical ventilation. Respir Care. 2010;55(7):837-844.
2. O'Doherty MJ, Thomas SH, Page CJ, Treacher DF, Nunan TO. Delivery of a nebulized aerosol to a lung model during mechanical ventilation. Effect of ventilator settings and nebulizer type, position, and volume of fill. Am Rev Respir Dis. 1992;146(2):383-388.
3. Harvey CJ, O'Doherty MJ, Page CJ, Thomas SH, Nunan TO, Treacher DF. Comparison of jet and ultrasonic nebulizer pulmonary aerosol deposition during mechanical ventilation. Eur Respir J. 1997;10(4):905-909.

Recommendations 1.8

Voting for recommendations 1.8

**Recommendations 1.8**	When vibrating mesh nebulizer is utilized during invasive ventilation, it is recommended to be placed at the inlet of humidifier.	
**Evidence**	In vitro[1-5]	
	If you have additional evidence, please provide references:	
**Likert score of 1-9**	□1 □2 □3 □4 □5 □6 □7 □8 □9<br>1= absolutely disagree, 9 = absolutely agree	
**Comments**		
**Suggestions for future research**		

Table 1.8. Studies about the position of VMN in mechanical ventilation

Author, year	Study type	Ventilator Setting	Heated-Humidity	Bias flow (L/min)	Inhaled dose with nebulizer placed proximal to ventilator			Inhaled dose with nebulizer placed at inspiratory limb before Y-piece		Between Y piece and ETT	p
					15cm from ventilator	Inlet of humidifier	Outlet of humidifier	15cm from Y piece	Immediately before Y piece		
Ari, Areabi 2010[1]	In vitro	Vt 500ml, PEEP 5, f 15, Ramp flow pattern, Peak inspiratory flow 60L/min,	Y	0	8.4±2.1%			16.8±2.6%*		12.8±0.5%*	*0.042 compared with be placed 15cm from ventilator
			N		24.2±1.2%*			30.2±1.0%		14.5±1.0%*	*<0.05 compared with be placed 15cm from Y piece
Ari, Atalay, 2010[2]	In vitro	Vt 500ml, PEEP 5, f 20	Y	2	23.8±1.0%				13.4±1.1%		0.01
				5	21.4±0.4				9.7±0.6%		0.001
Anderson, 2017[3]	In vitro	MV 100ml/kg/min, PEEP 5	Y	4.5		19.1%	17.7%		3.4%*	7.1%*	*<0.001 compared with be placed Proximal to ventilator
Dugernier, 2015[4]	In vitro	Vt 500ml, PEEP 5, f 20, Constant Inspiratory Flow Pattern 30L/min	N	10	24.3±1.9%	21.2±0.9%		18.3±0.2%*	16.4±1.2%*	21.2±1.3%	*<0.05 compared with be placed 15cm from ventilator
		Decelerating Inspiratory Flow Pattern, 60L/min			22.1±2.0%	16.1±0.7%*		12.0±0.5%*	10.7±0.4%*	12.9±0.4%*	*<0.05 compared with be placed 15cm from ventilator
Ge, 2019[5]	In vitro	PCV, Vt 519, MV 6.6, PEEP 4, Inspiratory flow	N	0		29.8%	19.4%*		15.9%*		*<0.001 compared with be

	41L/min					placed humidifier inlet
	PCV, Vt 510, MV 6.9, PEEP 4	6	23.6%	21.1%*	20.8%*	
	APRV, Vt 466, MV 5.3, $PEEP_i$ 4	0	23.1%	17.9%*	9.5%*	
	APRVs, Vt 464, MV 9.5, $PEEP_i$ 4	0	34.1%	27.4%*	23.1%*	

VMN, vibrating mesh nebulizer; NR, not reported; Y, yes; N, no; PEEP, positive end expiratory pressure; PEEPi, intrinsic positive end-expiratory pressure; MV, minute ventilation; Vt, tidal volume; PCV, pressure-control ventilation; APRV, airway pressure release ventilation.

1. Ari A, Areabi H, Fink JB. Evaluation of aerosol generator devices at 3 locations in humidified and non-humidified circuits during adult mechanical ventilation. Respir Care. 2010;55(7):837-844.
2. Ari A, Atalay OT, Harwood R, Sheard MM, Aljamhan EA, Fink JB. Influence of nebulizer type, position, and bias flow on aerosol drug delivery in simulated pediatric and adult lung models during mechanical ventilation. Respir Care. 2010;55(7):845-851.
3. Anderson AC, Dubosky MN, Fiorino KA, Quintana V, Kaplan CA, Vines DL. The Effect of Nebulizer Position on Aerosolized Epoprostenol Delivery in an Adult Lung Model. Respir Care. 2017;62(11):1387-1395.
4. Dugernier J, Wittebole X, Roeseler J, et al. Influence of inspiratory flow pattern and nebulizer position on aerosol delivery with a vibrating-mesh nebulizer during invasive mechanical ventilation: an in vitro analysis. J Aerosol Med Pulm Drug Deliv. 2015;28(3):229-236.
5. Ge HQ, Wang JM, Lin HL, et al. Effect of Nebulizer Location and Spontaneous Breathing on Aerosol Delivery During Airway Pressure Release Ventilation in Bench Testing. J Aerosol Med Pulm Drug Deliv. 2019;32(1):34-39.

Recommendations 1.9

Voting for recommendations 1.9

**Recommendations 1.9**	When continuous jet nebulizer is utilized during invasive ventilation, it is

	recommended to be placed proximal to the ventilator. When inspiration synchronized jet nebulizer is used, it is recommended to be placed at the inspiration limb before Y-piece.
**Evidence**	In vitro[1-3]
	If you have additional evidence, please provide references:
**Likert score of 1-9**	□1 □2 □3 □4 □5 □6 □7 □8 □9<br>1= absolutely disagree, 9 = absolutely agree
**Comments**	
**Suggestions for future research**	

Table 1.9. Studies about the position of continuous jet nebulizer in mechanical ventilation

**Author, year**	**Study type**	**Ventilator Setting**	**Jet nebulizer**	**Heated-Humidity**	**Bias flow (L/min)**	**Inhaled dose with nebulizer placed at 15cm from ventilator**	**Inhaled dose with nebulizer placed at inspiratory limb before Y-piece**		**Between Y piece and ETT**	**p**
							**15cm from Y piece**	**immediately before Y**		

								piece		
Ari, Areabi 2010[1]	In vitro	Vt 500ml, PEEP 5, f 15 （ Oxygen 8L/min）	Continuous JN	Y	0	6.0±0.1%*	3.6±0.2%		4.7±0.5%	*0.02 compared with be placed 15cm from Y piece
				N		14.7±1.5%*	9.7±1.5%		7.6±0.9%	*0.006 compared with be placed 15cm from Y piece
Ari, Atalay, 2010[2]	In vitro	Vt 500ml, PEEP 5, f 20, (Oxygen 2.5L/min)	Continuous JN	Y	2	5.2±0.2%	4.7±0.1%			NS
					5	4.7±0.4	4.0±0.1%			NS
O'Doherty, 1992[3]	In vitro	MV 6-15L/min, f 10-20, Ti% 20-50%	Inspiratory synchronized JN	Y	NR Siemens Servo 900C			8.0±0.94%	5.4±0.15%	<0.05

JN, jet nebulizer; N, no; PEEP, positive end expiratory pressure; Y, yes; NR, not reported; MV, minute ventilation; NS, no significant.

1. Ari A, Areabi H, Fink JB. Evaluation of aerosol generator devices at 3 locations in humidified and non-humidified circuits during adult mechanical ventilation. Respir Care. 2010;55(7):837-844.
2. Ari A, Atalay OT, Harwood R, Sheard MM, Aljamhan EA, Fink JB. Influence of nebulizer type, position, and bias flow on aerosol drug delivery in simulated pediatric and adult lung models during mechanical ventilation. Respir Care. 2010;55(7):845-851.
3. O'Doherty MJ, Thomas SH, Page CJ, Treacher DF, Nunan TO. Delivery of a nebulized aerosol to a lung model during mechanical ventilation. Effect of ventilator settings and nebulizer type, position, and volume of fill. Am Rev Respir Dis. 1992;146(2):383-388.

Recommendations 1.10

Voting for recommendations 1.10

**Recommendations 1.10**	When ultrasonic nebulizer is utilized during invasive ventilation without bias

	flow, it is recommended to be placed at 15 cm from Y-piece at inspiratory limb; With bias flow, ultrasonic nebulizer is recommended to be placed at inlet of humidifier.
**Evidence**	In vitro[1-4], in vivo[5]
	If you have additional evidence, please provide references:
**Likert score of 1-9**	□1 □2 □3 □4 □5 □6 □7 □8 □9<br>1= absolutely disagree, 9 = absolutely agree
**Comments**	
**Suggestions for future research**	

Table 1.10. Studies about the position of USN in mechanical ventilation

Author, year	Study type	Ventilator Setting	Heated-Humidity	Bias flow (L/min)	Device brand / Drug volume	Inhaled dose with nebulizer placed proximal to ventilator			Inhaled dose with nebulizer placed at inspiratory limb before Y-piece		Between Y piece and ETT	p
						15cm from ventilator	Inlet of humidifier	Outlet of humidifier	15cm from Y piece	Immediately before Y piece		
Ari, Areabi 2010[1]	In vitro	Vt 500ml, PEEP 5, f 15	Y	0		4.6±2.0%			16.5±4.3%*		10.1±3.9 %	*0.049 compared with be placed 15cm from ventilator
			N			10.5±0.3 %			24.7±4.4%*		10.7±1.5 %	*0.049 compared with be placed 15cm from ventilator
O'Doherty, 1992[2]	In vitro	MV 6-15L/min, f 10-20, Ti% 20-50%	Y	NR	Flsoneb					4.2±0.4 (SEM)%	5.1±0.7 (SEM)%	NS
					Samsonic					3.3±0.11 (SEM)%	3.1±0.19 (SEM)%	NS
Parker, 2017[3]	In vitro	Vt 560ml, PEEP 5, f 15	Y	2			50.1±4.5 μg			30.1±3.2μg		<0.01
									**50cm from Y piece**	**Immediately before Y piece**		
Thomas, 1993[4]	In vitro	MV 9L/min, f 15, Ti% 25%	Y	NR	Samsonic 3ml drug volume				3.2±0.1%	3.1±0.3%		NS
					Samsonic 18ml drug volume				12.0±1.6%	15.9±1.8%		NS

					Fisoneb 3ml drug volume	4.4±1.3%	5.4±1.4%	NS
					**Population**	**Pulmonary bioavailability (%)**		
						**Inlet of humidifier**	**Immediately before Y piece**	
Moraine, 2009[5]	In vivo	MV 7-8L/min, f 12-13 (Evita 2 or 4, Drager)	Y	0	Patients about to undergo elective cardiac surgery	0.9±0.1 (SE)%	1.1±0.2 (SE)%	NS

USN, ultrasonic nebulizer; Vt, tidal volume; MV, minute ventilation; N, no; PEEP, positive end expiratory pressure; Y, yes; NS, no significant; NR, not reported; SEM, standard error of mean.

1. Ari A, Areabi H, Fink JB. Evaluation of aerosol generator devices at 3 locations in humidified and non-humidified circuits during adult mechanical ventilation. Respir Care. 2010;55(7):837-844.
2. O'Doherty MJ, Thomas SH, Page CJ, Treacher DF, Nunan TO. Delivery of a nebulized aerosol to a lung model during mechanical ventilation. Effect of ventilator settings and nebulizer type, position, and volume of fill. Am Rev Respir Dis. 1992;146(2):383-388.
3. Parker DK, Shen S, Zheng J, et al. Inhaled Treprostinil Drug Delivery During Mechanical Ventilation and Spontaneous Breathing Using Two Different Nebulizers. Pediatr Crit Care Med. 2017;18(6):e253-e260.
4. Thomas SH, O'Doherty MJ, Page CJ, Treacher DF, Nunan TO. Delivery of ultrasonic nebulized aerosols to a lung model during mechanical ventilation. Am Rev Respir Dis. 1993;148(4 Pt 1):872-877.
5. Moraine JJ, Truflandier K, Vandenbergen N, Berre J, Melot C, Vincent JL. Placement of the nebulizer before the humidifier during mechanical ventilation: Effect on aerosol delivery. Heart Lung. 2009;38(5):435-439.

Recommendations 1.11

Voting for recommendations 1.11

**Recommendations 1.11**	When ultrasonic nebulizer is placed at the inspiratory limb before Y-piece, adding a spacer is recommended.
**Evidence**	In vitro[1,2]
	If you have additional evidence, please provide references:
**Likert score of 1-9**	□1 □2 □3 □4 □5 □6 □7 □8 □9<br>1= absolutely disagree, 9 = absolutely agree
**Comments**	
**Suggestions for future research**	

Table 1.11. Studies about USN with spacer in mechanical ventilation

**Author, year**	**Study type**	**Ventilator Setting**	**Heated-Humidity**	**Nebulizer brand**	**Position**	**Drug volume**	**Spacer type/ Inhaled dose**		
							No spacer	Spacer (600ml)	**p**
O'Doherty, 1992[1]	In vitro	MV 6-15L/min, f 10-20, Ti% 20-50%	Y	Fisoneb	In the inspiratory limb immediately before the Y		4.2±0.4%	14.8±1.5%	<0.05

				Samsonic	piece		3.3±0.11%	6.8±0.8%	<0.05
				Samsonic		3ml	3.1±0.3%	6.8±1.6%	<0.05
Thomas, 1993[2]	In vitro	MV 9L/min, f 15, Ti% 25%	Y	Samsonic	In the inspiratory limb immediately before the Y piece	18ml	15.9±1.8%	22.3±5.0%	NS
				Fisoneb		3ml	5.4±1.4%	14.4±1.2%	<0.05

USN, ultrasonic nebulizer; MV, minute ventilation; Y, yes; NS, no significant.

1. O'Doherty MJ, Thomas SH, Page CJ, Treacher DF, Nunan TO. Delivery of a nebulized aerosol to a lung model during mechanical ventilation. Effect of ventilator settings and nebulizer type, position, and volume of fill. Am Rev Respir Dis. 1992;146(2):383-388.
2. Thomas SH, O'Doherty MJ, Page CJ, Treacher DF, Nunan TO. Delivery of ultrasonic nebulized aerosols to a lung model during mechanical ventilation. Am Rev Respir Dis. 1993;148(4 Pt 1):872-877.

Recommendations 1.12

Voting for recommendations 1.12

**Recommendations 1.12**	When metered dose inhaler is utilized during invasive ventilation, it is recommended to be used with a spacer with volume > 150mL.
**Evidence**	In vitro[1-7], in vivo[8,9]
	If you have additional evidence, please provide references:
**Likert score of 1-9**	□1 □2 □3 □4 □5 □6 □7 □8 □9<br>1= absolutely disagree, 9 = absolutely agree
**Comments**	
**Suggestions for future research**	

Table 1.12. Studies about MDI with spacer in mechanical ventilation

Author, year	Study type	Population	Ventilator Setting	Heated-Humidity	Position	Spacer type/ Inhaled dose					p
						**A. 165ml**	**B. 20ml**	**C. 210ml**	**D.35ml**		
Boukhettala, 2015[1]	In vitro	Adult	Vt 450ml, PEEP 6, f 15	N	Between the inspiratory circuit and the Y-piece	24.5±1.6%	9.4±1.9%	31.9±0.6%			Each method (<0.05)
				Y		12.8±1.0%	6.1±0.8%	15.5±1.3%			Each method (<0.05)
					**Attached directly to the ETT between the Y and 15-mm adapter using the Medicomp actuator//adapter**		**Inspiratory limb just proximal to the patient Y// Monaghan Aerovent reservoir**		**Inspiratory limb just proximal to the patient Y// Aerosol Cloud Enhancer**		
Harwood, 1994[2]	In vitro	Adult	Vt 800ml f 12 Ti 1s	N	1.69%		21.4%		46.3%		Each method (<0.05)
					**Medicomp MDl actuator/adapter**		**Mona~an AeroVent reservoir placed on the inspiratory limb just proximal to the patient Y connector**		**Monaghan AeroVent reservoir placed inline between the elbow adapter and the 15-mm ETT adapter**		
Rau, 1992[3]	In vitro	Adult	MV 9.6L/min, f 12, PEEP 0	N	7.3%		32.1%*		29%*		*<0.01 compared with Medicomp MDl actuator/adapter
						**Hudson inline**	**Mini Spacer**	**Aero Vent**	**ACE**	**Medi Spacer**	
Rau, 1998[4]	In vitro	Adult	Vt 800ml, f 10,	Y	Albuterol	12.0±0.9%	17.2±1.2	17.7±2.5%	30.0±2.0	31.8±1.6 %	<0.005 (ANOVA)

					Flunisolide	5.0±0.9%	13.1±2.2%	11.4±1.8%	12.4±2.8 %	21.0±1.8 %	<0.005 (ANOVA)
						**Bidirectional, (22 mm inner diameter/22 mm outer diameter)**	**Large unidirectional, (15 mm inner Ammeter /22 mm outer diameter)**	**Small unidirectional, (15 mm inner diameter/15 mm outer diameter)**			
Williams, 2018[5]	In vitro	Adult	Vt 450ml, PEEP 5, f 15	With exhaled humidity	Double swivel	13.1±2.5%	7.1±1.0%	3.4±1.3%			0.001(ANOVA)
				N		31.6±4.4%	24.1±5.5%	18.8±2.5%			0.034(ANOVA)
						**A new version of Combihaler**	**An old version of Combihaler**	**Solo T-piece**	**pMDI T-piece adapter**		
Seif, 2021[6]	In vitro	Adult	Vt 500ml, PEEP 5, f 15	Y	In the inspiratory limb immediately before the Y piece	53.3±20.0μg*	47.4±17.0μg*		79.6±10.6μg		*<0.05 compared with pMDI T-piece adapter
						**CombiHaler / Inspiratory limb**	**MiniSpacer / Inspiratory limb**	**F&P Wye / At the wye**	**Between HME and ETT**		
Naughton, 2021[7]	In vitro	Adult	Vt 500ml, f 15	Y		124.62±18.68μg	84.62±8.97 μg	56.92±5.22μg			<0.0001 (ANOVA)
				N					80.00±4.49 μg		

			Vt 400ml, f 20	Y		134.46±7.58 μg	68.92±3.67 μg	75.38±8.14 μg	<0.0001 (ANOVA)
						**Large chamber (700ml)**	**Small chamber (167ml)**	**Inline device**	
Fuller, 1994[8]	In vivo	Patients undergoing mechanically assisted ventilation for a variety of clinical reasons and each judged to require inhaled bronchodilator therapy	NR	NR	In the inspiratory ventilator tubing, 22 cm from the ETT or TT	6.33%	5.53%	1.67%	<0.05 (ANOVA)
						**A right-angle adaptor attached to the endotracheal tube**		**Small volume spacer**	
Marik, 1999[9]	In vivo	Mechanically ventilated patients who had not received a bronchodilator in the previous 48 h	Vt >500ml, Ti/Ttotal 30%,	NR	In the inspiratory limb immediately before the Y piece	41±61μg（9%）		169±129μg（38%）	0.02

MDI, metered dose inhaler; ACE, aerosol cloud enhancer; MV, minute ventilation; N, no; PEEP, positive end expiratory pressure; Y, yes; ETT, endotracheal tube; TT, tracheotomy; ANOVA, One-way analysis of variance; HME, heat moisture exchanger.

1. Boukhettala N, Poree T, Diot P, Vecellio L. In vitro performance of spacers for aerosol delivery during adult mechanical ventilation. J Aerosol Med Pulm Drug Deliv. 2015;28(2):130-136.
2. Harwood R, Rau JL, Thomas-Goodfellow L. A comparison of three methods of metered dose bronchodilator delivery to a mechanically ventilated adult lung model. Respiratory care. 1994;39(9):886-891.
3. Rau JL, Harwood RJ, Groff JL. Evaluation of a reservoir device for metered-dose bronchodilator delivery to intubated adults. An in vitro study. Chest. 1992;102(3):924-930.
4. Rau JL, Dunlevy CL, Hill RL. A comparison of inline MDI actuators for delivery of a beta agonist and a corticosteroid with a mechanically ventilated lung model. Respiratory care. 1998;43(9):705-712.
5. Williams JP, Ari A, Shanmugam R, Fink JB. The Effect of Different Closed Suction Catheter Designs and pMDI Adapters on Aerosol Delivery in Simulated Adult Mechanical Ventilation

with and Without Exhaled Humidity. Respir Care. 2018;63(9):1154-1161.
6. Seif SM, Elnady MA, Rabea H, Saeed H, Abdelrahim MEA. Effect of different connection adapters on aerosol delivery in invasive ventilation setting; an in-vitro study. Journal of Drug Delivery Science and Technology. 2021;61.
7. Naughton PJ, Joyce M, Mac Giolla Eain M, O'Sullivan A, MacLoughlin R. Evaluation of Aerosol Drug Delivery Options during Adult Mechanical Ventilation in the COVID-19 Era. Pharmaceutics. 2021;13(10):1574.
8. Fuller HD, Dolovich MB, Turpie FH, Newhouse MT. Efficiency of bronchodilator aerosol delivery to the lungs from the metered dose inhaler in mechanically ventilated patients. A study comparing four different actuator devices. Chest. 1994;105(1):214-218.
9. Marik P, Hogan J, Krikorian J. A comparison of bronchodilator therapy delivered by nebulization and metered-dose inhaler in mechanically ventilated patients. Chest. 1999;115(6):1653-1657.

Recommendations 1.13

Voting for recommendations 1.13

**Recommendations 1.13**	During invasive ventilation, metered dose inhaler and spacer are recommended to be placed in the inspiratory limb before the Y-piece.
**Evidence**	In vitro[1,2], in vivo[3]
	If you have additional evidence, please provide references:
**Likert score of 1-9**	□1 □2 □3 □4 □5 □6 □7 □8 □9<br>1= absolutely disagree, 9 = absolutely agree
**Comments**	
**Suggestions for future research**	

Table 1.13. Studies about the position of MDI with spacer in mechanical ventilation

Author, year	Study type	Population	Ventilator Setting	Heated-Humidity	Bias flow (L/min)	Inhaled dose with nebulizer placed proximal to ventilator	Inhaled dose with nebulizer placed at inspiratory limb before Y-piece		Between Y piece and ETT	On the end of the ETT/TT	p
						15cm from ventilator	15cm from Y piece	Immediately before Y piece			
Ari, Areabi 2010[1]	In vitro	Adult	Vt 500ml, PEEP 5, f 15	Y	0	2.5±0.8%	17.0±1.0%*		7.6±1.3%		*0.008 compared with be placed 15cm from ventilator
				N		7.9±1.5%	27.8±3.3%*		22.1±1.5%*		*<0.05 compared with be placed 15cm from ventilator
Ke, 2020[2]	In vitro	Adult	Vt 500ml, PEEP 5, f 15 (PB840)	N	1.5+Trigger flow	1.0±0.7%*	26.1±2.0%		9.6±2.5%*		*<0.001 compared with be placed 15cm from Y piece
Fuller, 1994[3]	In vivo	Patients require inhaled bronchodilator therapy	NR	NR	NR			1.7%		3.9%	NS

MDI, metered dose inhaler; ETT, endotracheal tube; TT, tracheotomy; NR, not reported; NS, no significant

1. Ari A, Areabi H, Fink JB. Evaluation of aerosol generator devices at 3 locations in humidified and non-humidified circuits during adult mechanical ventilation. Respir Care. 2010;55(7):837-844.
2. Ke WR, Wang WJ, Lin TH, et al. In Vitro Evaluation of Aerosol Performance and Delivery Efficiency During Mechanical Ventilation Between Soft Mist Inhaler and Pressurized Metered-Dose Inhaler. Respir Care. 2020;65(7):1001-1010.
3. Fuller HD, Dolovich MB, Turpie FH, Newhouse MT. Efficiency of bronchodilator aerosol delivery to the lungs from the metered dose inhaler in mechanically ventilated patients. A study comparing four different actuator devices. Chest. 1994;105(1):214-218.

Recommendations 1.14

Voting for recommendations 1.14

**Recommendations 1.14**	During high-frequency oscillatory ventilation, nebulizers are recommended to be placed between the Y-piece and the endotracheal tube.	
**Evidence**	In vitro[1]	
	If you have additional evidence, please provide references:	
**Likert score of 1-9**	□1 □2 □3 □4 □5 □6 □7 □8 □9<br>1= absolutely disagree, 9 = absolutely agree	
**Comments**		
**Suggestions for future research**		

Table 1.14. Position of the nebulizer during HFOV

Author, year	Study type	Ventilator Setting	Heated-Humidity	VMN / Inhaled dose			Continuous JN / Inhaled dose		
				Proximal to ventilator	Between the circuit and the endotracheal tube	p	Proximal to ventilator	Between the circuit and the endotracheal tube	p
Fang, 2016[1]	In vitro	MAP 30 cmH_2O, bias flow 40L/min, F 5 Hz, Power 8, Ti% 33%	Y	26.0±14.0 μg	1139.3±279.2 μg	0.001	0.15±7.6 μg	144.6±53.4 μg	0.001

HFOV, high frequency oscillatory ventilation; VMN, vibrating mesh nebulizer; JN, jet nebulizer; MAP, mean airway pressure

1.. Fang TP, Lin HL, Chiu SH, et al. Aerosol Delivery Using Jet Nebulizer and Vibrating Mesh Nebulizer During High Frequency Oscillatory Ventilation: An In Vitro Comparison. J Aerosol Med Pulm Drug Deliv. 2016;29(5):447-453.

Recommendations 1.15

Voting for recommendations 1.15

**Recommendations 1.15**	The efficiency of aerosol delivery in dry ventilator circuits is higher than that in humidified ventilator circuits. Considering the potential harms of dry gas on patient airway, turning off humidifier is not recommended for routine aerosol therapy.
**Evidence**	In vitro[1-11], in vivo[6,13,14]
	If you have additional evidence, please provide references:
**Likert score of 1-9**	□1 □2 □3 □4 □5 □6 □7 □8 □9<br>1= absolutely disagree, 9 = absolutely agree
**Comments**	
**Suggestions for future research**	

Table 1.15-1. In-vitro studies compared aerosol delivery in invasive ventilated patients with and without humidification.

Author, year	Study type	Inhaled medication	Aerosol generator	Inhaled dose			MMAD (μm)			Sputum levels of deposited antibiotics (μg/ml/mg)		
				With humidification	Without humidification	p	With humidification	Without humidification	p	With humidification	Without humidification	p
Fuller,1992[1]	In vitro	^{99m}Tc-SC	Inspiratory synchronized JN	4.6 ± 2.1%	7.8 ± 0.5%	0.002						
Diot,1995[2]	In vitro	Albuterol	MDI	15.4 ± 0.2%	25.1 ± 3.7%	0.011						
Fink,1996[3]	In vitro	Albuterol	MDI+spacer	15.9-20.2%	28.8 - 39%	<0.005						
Fink,1999[4]	In vitro	Albuterol	CFC-propelled MDI+spacer	16.2 ± 2.3%	30.4 ± 3.1%	<0.001						
			HFA-propelled MDI+spacer	31.4 ± 2.2%	42.8 ± 1.2%	<0.001						
Goode,2001[5]	In vitro	Albuterol	MDI+spacer	26.3 ± 2.0%	42.4 ± 3.2%	0.041						
				16.7 ± 2.3%	27.5 ± 1.3%	0.003						
Miller,2003[6]	In vitro	Albuterol	Inspiratory synchronized JN	9.6 ± 1.0%	37.4 ± 1.6%	<0.0001						
			Continuous JN	5.7±0.5%	10.4±0.8%	<0.0001						
	In vivo	Gentamicin or amikacin	Inspiratory synchronized JN							3.2 ± 0.5%	12.6 ± 1.8%	<0.001
			Continuous JN							0.8 ± 0.1%	1.8 ± 0.3%	0.0005
Mitchell,2003[7]	In vitro	Beclomethasone	HFA-propelled MDI+spacer				2.8±0.1	1.2 ± 0.1				
Martin,2005[8]	In vitro	Salbutamol	MDI+spacer				3.75 ± 0.05	1.97 ± 0.13	<0.01			
Lin,2009[9]	In vitro	Albuterol	MDI+spacer	11.4 ± 3.8%	21.8 ± 3.3%	0.002						
Ari,2010[10]	In vitro	Albuterol	Continuus JN	6.0 ± 0.1%	14.7 ± 1.5%	0.005						
			VMN	16.8 ± 2.6%	30.2 ± 1.0%	0.01						
			USN	16.5 ± 4.3%	24.7 ± 4.4%	NS						
			MDI+spacer	17.0 ± 1.0%	27.8 ± 3.3%	0.031						
Boukhettala,	In vitro	Amikacin	VMN+T-adapter	9.9 ± 0.8%	14.3 ± 0.9%	NR						

2014[11]			VMN+spacer	19.5 ± 1.2%	49.1% ±3%	NR
		Salbutamol	MDI+spacer A	12.8±1%	24.5±1.6%	NR
			MDI+spacer B	6.1±0.8%	9.4±1.9%	NR
			MDI+spacer C	15.5±1.3%	31.9±0.6%	NR
Ari,2016[12]	In vitro	Albuterol	Continuous JN	1.40± 0.22	3.09 ± 0.59	0.009
			VMN	3.05 ± 0.27	4.61 ± 0.35	0.009

MMAD, mass median aerodynamic diameter; ^{99m}Tc-SC, sulphur colloid with technetium-99 m; JN,jet nebulizer; MDI, metered dose inhaler; CFC, chlorofluorocarbon; HFA, hydrofluoroalkane; VMN, vibrating mesh nebulizer; USN,ultrasonic nebulizer; NR, not reported; NS, not significant

Table 1.15-2. In-vivo studies compared aerosol delivery in invasive ventilated patients with and without humidification.

Author, year	Study type	Inhaled medication	Aerosol generator	Salbutamol urine levels at 0.5h,as percent [mean (SD)] of dose			Salbutamol urine levels at 24h, as percent [mean (SD)] of dose			MV-days			ICU-days		
				With humidification	Without humidification	p	With humidification	Without humidification	p	With humidification	Without humidification	p	With humidification	Without humidification	p
Moustafa,2017[13]	In vivo	Salbutamol	Continuous JN	0.7 (0.5)	0.9 (0.6)	NS	6.2 (3.3)	6.6 (4.1)	NS						
			VMN	1.7 (0.8)	1.8 (1.1)		10.1 (6.9)	10.5 (4.0)							
			MDI+spacer	2.0 (1.1)	2.5 (1.3)		7.8 (2.5)	8.0 (3.4)							
Moustafa,2017[14]	In vivo	Salbutamol	Continuous JN							6.0 (1.5)	5.4 (1.0)	NS	8.7 (2.5)	8.3 (1.9)	NS
			VMN							5.8 (1.0)	5.6 (1.0)		7.6 (2.5)	7.9 (2.0)	
			MDI+spacer							6.2 (1.1)	5.7 (1.4)		9.7 (2.8)	8.7 (2.3)	

JN,jet nebulizer; VMN, vibrating mesh nebulizer; NS,not significant

Table 1.15-3. The study compared aerosol delivery in invasive ventilated patients with dry and humidified spacer .

Author, year	Study type	Inhaled medication	Aerosol generator	Inhaled dose (%)				
				Dry chamber	Heater on 1h	Heater on 2h	Heater on 3h	Heater off
Lin,2009[9]	In vitro	Albuterol	MDI	21.8 ± 3.3[a, b,c]	23±2.1	11.4±3.8	12.3±0.8	12.7±0.3

MDI, metered dose inhaler

[a] *P*=.004 when compared to heater on 2h

[b] *P*=.009 when compared to heater on 3h

[c] P=.013 when compared to heater off

1. Fuller HD, Dolovich MB, Chambers C, Newhouse MT. Aerosol Delivery During Mechanical Ventilation: A Predictive In-Vitro Lung Model. Journal of Aerosol Medicine. 1992;5(4):251-259.
2. Diot P, Morra L, Smaldone GC. Albuterol delivery in a model of mechanical ventilation. Comparison of metered-dose inhaler and nebulizer efficiency. Am J Respir Crit Care Med. 1995;152(4 Pt 1):1391-1394.
3. Fink JB, Dhand R, Duarte AG, Jenne JW, Tobin MJ. Aerosol delivery from a metered-dose inhaler during mechanical ventilation. An in vitro model. Am J Respir Crit Care Med. 1996;154(2 Pt 1):382-387.
4. Fink JB, Dhand R, Grychowski J, Fahey PJ, Tobin MJ. Reconciling in vitro and in vivo measurements of aerosol delivery from a metered-dose inhaler during mechanical ventilation and defining efficiency-enhancing factors. Am J Respir Crit Care Med. 1999;159(1):63-68.
5. Goode ML, Fink JB, Dhand R, Tobin MJ. Improvement in aerosol delivery with helium-oxygen mixtures during mechanical ventilation. Am J Respir Crit Care Med. 2001;163(1):109-114.
6. Miller DD, Amin MM, Palmer LB, Shah AR, Smaldone GC. Aerosol delivery and modern mechanical ventilation: in vitro/in vivo evaluation. Am J Respir Crit Care Med. 2003;168(10):1205-1209.
7. Mitchell JP, Nagel MW, Wiersema KJ, Doyle CC, Migounov VA. The delivery of chlorofluorocarbon-propelled versus hydrofluoroalkane-propelled beclomethasone dipropionate aerosol to the mechanically ventilated patient: a laboratory study. Respir Care. 2003;48(11):1025-1032.
8. Martin AR, Finlay WH. The Effect of Humidity on the Size of Particles Delivered from Metered-Dose Inhalers. Aerosol Science and Technology. 2005;39(4):283-289.
9. Lin HL, Fink JB, Zhou Y, Cheng YS. Influence of moisture accumulation in inline spacer on delivery of aerosol using metered-dose inhaler during mechanical ventilation. Respir Care. 2009;54(10):1336-1341.
10. Ari A, Areabi H, Fink JB. Evaluation of aerosol generator devices at 3 locations in humidified and non-humidified circuits during adult mechanical ventilation. Respir Care. 2010;55(7):837-844.
11. Boukhettala N, Poree T, Diot P, Vecellio L. In vitro performance of spacers for aerosol delivery during adult mechanical ventilation. J Aerosol Med Pulm Drug Deliv. 2015;28(2):130-136.
12. Ari A, Harwood R, Sheard M, Alquaimi MM, Alhamad B, Fink JB. Quantifying Aerosol Delivery in Simulated Spontaneously Breathing Patients With Tracheostomy Using Different Humidification Systems With or Without Exhaled Humidity. Respir Care. 2016;61(5):600-606.
13. Moustafa IOF, Ali MRA, Al Hallag M, et al. Lung deposition and systemic bioavailability of different aerosol devices with and without humidification in mechanically ventilated patients. Heart & lung : the journal of critical care. 2017;46(6):464-467.
14. Moustafa IOF, ElHansy MHE, Al Hallag M, et al. Clinical outcome associated with the use of different inhalation method with and without humidification in asthmatic mechanically ventilated patients. Pulmonary pharmacology & therapeutics. 2017;45:40-46.

Recommendations 1.16

Voting for recommendations 1.16

**Recommendations 1.16**	When aerosol device is placed in the inspiratory limb, removing or bypassing the heat moisture exchanger is recommended.
**Evidence**	In vitro[1,2]
	If you have additional evidence, please provide references:
**Likert score of 1-9**	□1 □2 □3 □4 □5 □6 □7 □8 □9<br>1= absolutely disagree, 9 = absolutely agree
**Comments**	
**Suggestions for future research**	

Table 1.16. Studies compared aerosol delivery in invasive ventilated patients with HME.

Author,	Study	Exhaled	Inhaled	Aerosol	Placement of	Inhaled dose (%)	Inhaled dose (%)

year	type	humidity	medication	generator	aerosol generator	No HME	HME-AD (AirLife)	HME-AD (CircuVent)	HME-AD (Humid-Flo)	p	ThermoFlo HME	Without HME	p
Ari,2017[1]	In vitro	yes	Albuterol	VMN	In the inspiratory line 12 inches before the Y piece	10.8 ±0.2	9.2±0.7	9.8±1.9	9.9±1.1	0.89			
Ari,2017[2]	In vitro	yes	Albuterol	Continuous JN	In the inspiratory line 6 inches before the Y piece						3.47 ± 0.4	5.44 ± 0.17	0.001
				VMN	at the Y-adapter						6.61 ± 0.3	10.64 ± 0.53	0.001

HME,heat moisture exchanger; HME-AD, heat moisture exchanger specially designed for aerosol therapy; JN, jet nebulizer; VMN, vibrating mesh nebulizer

1. Ari A, Alwadeai KS, Fink JB. Effects of Heat and Moisture Exchangers and Exhaled Humidity on Aerosol Deposition in a Simulated Ventilator-Dependent Adult Lung Model. Respir Care. 2017;62(5):538-543.
2. Ari A, Dang T, Al Enazi FH, et al. Effect of Heat Moisture Exchanger on Aerosol Drug Delivery and Airway Resistance in Simulated Ventilator-Dependent Adults Using Jet and Mesh Nebulizers. J Aerosol Med Pulm Drug Deliv. 2018;31(1):42-48.

Recommendations 1.17

Voting for recommendations 1.17

**Recommendations 1.17**	Ventilator integrated breath-actuated jet nebulizer is not preferred over continuous jet nebulizer.	
**Evidence**	In vitro[1,2], in vivo[1]	
	If you have additional evidence, please provide references:	
**Likert score of 1-9**	□1 □2 □3 □4 □5 □6 □7 □8 □9<br>1= absolutely disagree, 9 = absolutely agree	
**Comments**		
**Suggestions for future research**		

Table 1.17. Studies compared aerosol delivery in invasive ventilated patients with inspiratory synchronized nebulization.

Author,	Study	Aerosol	Placement of	Bias	Humidified	Inhaled dose %	Sputum levels of deposited

year	type	generator	aerosol generator	flow	ventilator circuit				antibiotics (μg/ml/mg)		
						**Breath-actuated nebulization**	**continuous nebulization**	**p**	**Breath-actuated nebulization**	**continuous nebulization**	**p**
Miller,2003[1]	In vitro	JN	In the inspiratory line 12 inches before the Y piece	Yes	Yes	9.6 ±1.0	5.7±0.5	NR			
					No	37.4 ±1.6	10.4 ±0.8	NR			
	In vivo				Yes				3.2 ±0.5	0.8 ±0.1	NR
					No				12.6 ±1.8	1.8 ±0.3	NR
Wan,2014[2]	In vitro	JN	In the ventilator outlet 15 cm from the heater		Yes	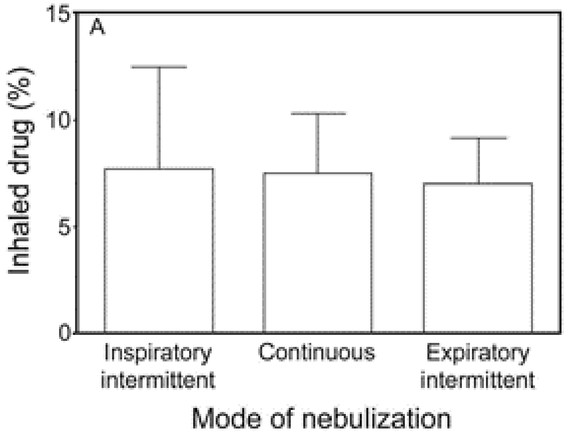		0.99			

JN, jet nebulizer; NR:not reported

1. Miller DD, Amin MM, Palmer LB, Shah AR, Smaldone GC. Aerosol delivery and modern mechanical ventilation: in vitro/in vivo evaluation. Am J Respir Crit Care Med. 2003;168(10):1205-1209.
2. Wan GH, Lin HL, Fink JB, et al. In vitro evaluation of aerosol delivery by different nebulization modes in pediatric and adult mechanical ventilators. Respir Care. 2014;59(10):1494-1500.

Recommendations 1.18

Voting for recommendations 1.18

**Recommendations 1.18**	The influence of ventilator integrated breath-actuated jet nebulizer on ventilator function and aerosol delivery efficiency varies between ventilators. Confirming the aerosol delivery performance for available ventilator before delivering aerosol is recommended.	
**Evidence**	In vitro[1]	
	If you have additional evidence, please provide references:	
**Likert score of 1-9**	□1 □2 □3 □4 □5 □6 □7 □8 □9<br>1= absolutely disagree, 9 = absolutely agree	
**Comments**		
**Suggestions for future research**		

Table 1.18. The study compared aerosol delivery in invasive ventilated patients with jet nebulizer system integrated in ventilators.

Author, year	Study type	Aerosol generator	Placement of aerosol generator	Ventilator	Absolute change after starting nebulization			
					Inspiratory flow(L/min)	Duration of Insufflation (ms)	Tidal volume (ml)	Peak Airway Pressure (cm H_2O)
Ehrmann ,2014[1]	In vitro	inspiratory synchronized JN	Between Y-piece and artificial airway	Evita XL	-1.8±0.4	20±4	-10±7	-0.3±0.1
				Avea	-0.1±0.1	7±3	7±4	0.03±0.06
				Galileo	0.1±0.6	14±4	11±7	0.3±0.4
				G5	-0.5±0.3	7±2	1±8	-0.2±0.1

JN, jet nebulizer

1. Ehrmann S, Lyazidi A, Louis B, et al. Ventilator-integrated jet nebulization systems: tidal volume control and efficiency of synchronization. Respir Care. 2014;59(10):1508-1516.

Recommendations 1.19

Voting for recommendations 1.19

**Recommendations 1.19**	Metered-dose inhaler should be primed, shaken, with actuation at the beginning of inspiration, with a minimum of 15 seconds between two puffs.
**Evidence**	In vitro[1,2]
	If you have additional evidence, please provide references:
**Likert score of 1-9**	□1 □2 □3 □4 □5 □6 □7 □8 □9<br>1= absolutely disagree, 9 = absolutely agree
**Comments**	
**Suggestions for future research**	

Table 1.19. Studies compared aerosol delivery in invasive ventilated patients with different actuation technique of MDI.

Author, year	Study type	Humidification	Spacer	Puffs	MDI delivery dose (%)					
					**Intervals of 15s**	**Intervals of 60s**	**p**	**Synchronized with the beginning of inspiration**	**Synchronized with the beginning of expiration**	**p**
Fink,1999[1]	In vitro	Yes	Yes	four	NR	NR	NS			
				eight	47.5±5.7	41.7±6.5	<0.02			
Diot,1995[2]	In vitro	No	Aerovent					25.1±3.7	16.2±0.98	0.02
			Marquest					7.2±0.7	1±0.05	0.005
					**MDI delivery dose (μg)**					
					**Actuation at the midway point of inhalation**	**Actuation at the start of inhalation**		**Actuation at the end of exhalation**	**Actuation at the start of exhalation**	**p**
Naughton, 2021[3]		Yes	Yes		93.23 ± 9.27	84.62 ± 8.97[a,b,c]		54.15 ± 4.91	36.62 ± 9.00	<0.0001

[a] *P*=.1733 when compared to actuation at the midway point of inhalation

[b] *P*=.0002 when compared to actuation at the end of exhalation

[c] P<.0001 when compared to actuation at the start of exhalation

MDI, metered dose inhaler; NR, not reported; NS, not significant

1. Fink JB, Dhand R, Grychowski J, Fahey PJ, Tobin MJ. Reconciling in vitro and in vivo measurements of aerosol delivery from a metered-dose inhaler during mechanical ventilation and defining efficiency-enhancing factors. Am J Respir Crit Care Med. 1999;159(1):63-68.
2. Diot P, Morra L, Smaldone GC. Albuterol delivery in a model of mechanical ventilation. Comparison of metered-dose inhaler and nebulizer efficiency. Am J Respir Crit Care Med. 1995;152(4 Pt 1):1391-1394.
3. Naughton, P.J, Joyce, M,Mac Giolla Eain, M.; O'Sullivan, A.;MacLoughlin, R. Evaluation of Aerosol Drug Delivery Options during Adult Mechanical Ventilation in the COVID-19 Era. Pharmaceutics.2021, 13, 1574. https://doi.org/10.3390/pharmaceutics13101574

Recommendations 1.20

Voting for recommendations 1.20

**Recommendations 1.20**	Increasing fill volume in jet or ultrasonic nebulizer to improve aerosol delivery efficiency is not recommended.
**Evidence**	In vitro[1,2]
	If you have additional evidence, please provide references:
**Likert score of 1-9**	□1 □2 □3 □4 □5 □6 □7 □8 □9<br>1= absolutely disagree, 9 = absolutely agree
**Comments**	
**Suggestions for future research**	

Table 1.20. Vitro Studies compared fill volume of nebulization in invasive ventilated patients

Author,	Ventilator	Inhaled	Drug	Nebulizer	Inhaled dose (%)	p

year	Setting	medication	concentration		Fill volume				
					3ml	6ml	12ml	18ml	
O'Doherty, 1992[1]	MV 6-15L/min, f 10-20, Ti% 20-50%	^{99m}Tc HSA	16.7μg/ml	Inspiratory synchronized JN	5.4±0.15	10.8±1.45			< 0.05
				USN-Fisoneb	4.4±0.28	7.3±0.33*	7.97±1.0*		* <0.05 compared with 3ml
				USN-Samsonic	3.1±0.2	6.4±0.8*	9.9±1.8*	15.4±1.1*	* <0.05 compared with 3ml
Thomas, 1993[2]	MV 9L/min, f 15, Ti% 25%	^{99m}Tc HSA	16.7μg/ml	USN-DP100	3.2±0.8		14.5±5.3*	11.5±2.0*	* <0.05 compared with 3ml
				USN-Ultraneb	3.8±8.2		8.0±0.2*	8.7±03.1*	* <0.05 compared with 3ml
				USN-Fisoneb	5.4±1.4	8.1±1.8	8.4±2.0		NS
				USN-Samsonic	3.1±0.3	8.8±4.6*	10.0±3.4*	15.9±1.8*	* <0.05 compared with 3ml

JN, jet nebulizer; USN, ultrasonic nebulizer; ^{99m}Tc HAS, 99mTechnetium human serum albumin; N, no; NS, no significant; Y, yes.

1. O'Doherty. M. J,Thomas.S. H,Page.C. J,Treacher. D. F,Nunan. T. O. Delivery of a nebulized aerosol to a lung model during mechanical ventilation. Effect of ventilator settings and nebulizer type, position, and volume of fill. Am Rev Respir Dis. 1992;146(2):383-388.
2. Thomas. S. H, O'Doherty. M. J, Page. C. J, Treacher. D. F, Nunan, T. O. Delivery of ultrasonic nebulized aerosols to a lung model during mechanical ventilation. Am Rev Respir Dis.1993;148(4):872-877

Recommendations 1.21

Voting for recommendations 1.21

**Recommendations 1.21**	Aerosol delivery efficiency varies between endotracheal tube and tracheotomy

	tube. Changing tubes for the sole purpose of improving aerosol delivery efficiency is not recommended.
**Evidence**	In vitro[1-4]
	If you have additional evidence, please provide references:
**Likert score of 1-9**	□1 □2 □3 □4 □5 □6 □7 □8 □9<br>1= absolutely disagree, 9 = absolutely agree
**Comments**	
**Suggestions for future research**	

Table 1.21. Studies compared aerosol delivery in invasive ventilated patients with endotracheal tube and tracheotomy tube.

Author, year	Study type	Aerosol generator	Inhaled medication	Tube size	Respirator support	Inhaled dose (%)		
						Tracheotomy tube	Endotracheal tube	p
Ari,2012[1]	In vitro	Continuous JN	Albuterol	8	Resuscitation bag	45.75±1.80	27.23±8.98	0.025
Ari,2015[2]	In vitro	Continuous JN	Albuterol	8	Mechanical ventilation	3.9±0.5	3.2±0.1	0.10
		MDI				14.7±0.1	11.6±0.9	0.046
Dhanani,2018[3]	In vitro	VMN	40 mg/ml tobramycin	7	Mechanical ventilation	23.7 ± 0.04	39.4 ± 0.07	NS

			100 mg/ml tobramycin			30.7 ± 0.08	26.5 ± 0.01	
			40 mg/ml tobramycin	8		21.9 ± 0.05	39.4 ± 0.02	
			100 mg/ml tobramycin			31.2 ± 0.01	28.1 ± 0.04	
			40 mg/ml tobramycin	9		25.6 ± 0.03	30.9 ± 0.09	
			100 mg/ml tobramycin			28.9 ± 0.04	34.5 ± 0.05	
Leung,2015[4]	In vitro	DPI	Zanamivir	7	Resuscitation bag	35.3±3.3	33.1±2.8	NS

JN, jet nebulizer; MDI, metered dose inhaler; VMN, vibrating mesh nebulizer; DPI,dry powder inhaler; NS,not significant

1. Ari A, Harwood RJ, Sheard MM, Fink JB. An in vitro evaluation of aerosol delivery through tracheostomy and endotracheal tubes using different interfaces. Respir Care. 2012;57(7):1066-1070.
2. Ari A, Harwood RJ, Sheard MM, Fink JB. Pressurized Metered-Dose Inhalers Versus Nebulizers in the Treatment of Mechanically Ventilated Subjects With Artificial Airways: An In Vitro Study. Respir Care. 2015;60(11):1570-1574.
3. Dhanani JA, Tang P, Wallis SC, et al. Characterisation of 40mg/ml and 100mg/ml tobramycin formulations for aerosol therapy with adult mechanical ventilation. Pulmonary pharmacology & therapeutics. 2018;50:93-99.
4. Leung SSY, Parumasivam T, Tang P, Chan HK. A Proof-of-Principle Setup for Delivery of Relenza((R)) (Zanamivir) Inhalation Powder to Intubated Patients. J Aerosol Med Pulm Drug Deliv. 2016;29(1):30-35.

Recommendations 1.22

Voting for recommendations 1.22

**Recommendations 1.22**	Aerosol delivery efficiency is higher with a large size of endotracheal tube, but changing endotracheal tube for the sole purpose of improving aerosol delivery efficiency is not recommended.	
**Evidence**	In vitro[1-3]	
	If you have additional evidence, please provide references:	
**Likert score of 1-9**	□1 □2 □3 □4 □5 □6 □7 □8 □9<br>1= absolutely disagree, 9 = absolutely agree	
**Comments**		
**Suggestions for future research**		

Table 1.22. Studies compared aerosol delivery in invasive ventilated patients with different sized endotracheal tube.

Author, year	Study type	Aerosol generator	Inhaled medication	Inhaled dose (%)				Inhaled dose (%)			
				6.0 ETT	7.5 ETT	9.0 ETT	p	7.0 ETT	8.0 ETT	9.0 ETT	p
Crogan ,1989[1]	In vitro	MDI	Metaproterenol	3.0±1.9	4.7±3.2	6.5±4.4	<0.05				
Longest,2014[2]	In vitro	VMN(Aeroneb Lab)	Albuterol					30.8 ± 2.7	36.4 ± 1.5	42.4 ± 4.3	<0.05
		VMN(eFlow Rapid)						27.6 ± 0.8	36.4 ± 4.9	39.4 ± 4.6	<0.05
Leung, 2015[3]	In vitro	DPI	Zanamivir					33.1±2.8		37.9±2.9	NS

MDI, metered dose inhaler; VMN, vibrating mesh nebulizer;DPI,dry powder inhaler; NS:not significant

1. Crogan SJ, Bishop MJ. Delivery efficiency of metered dose aerosols given via endotracheal tubes. Anesthesiology. 1989;70(6):1008-1010.
2. Longest PW, Azimi M, Golshahi L, Hindle M. Improving aerosol drug delivery during invasive mechanical ventilation with redesigned components. Respir Care. 2014;59(5):686-698
3. Leung SSY, Parumasivam T, Tang P, Chan HK. A Proof-of-Principle Setup for Delivery of Relenza((R)) (Zanamivir) Inhalation Powder to Intubated Patients. J Aerosol Med Pulm Drug Deliv. 2016;29(1):30-35.

Recommendations 1.23

Voting for recommendations 1.23

**Recommendations 1.23**	When heliox is utilized for invasive ventilation, aerosol delivery efficiency can improve. However, adding heliox for the sole purpose of improving aerosol delivery efficiency is not recommended.	
**Evidence**	In vitro[1]	
	If you have additional evidence, please provide references:	
**Likert score of 1-9**	□1 □2 □3 □4 □5 □6 □7 □8 □9<br>1= absolutely disagree, 9 = absolutely agree	
**Comments**		
**Suggestions for future research**		

Table 1.23. Studies compared aerosol delivery in invasive ventilated patients with helium–oxygen mixtures and nitrogen-oxygen mixture.

Author, year	Study type	Aerosol generator	Inhaled medication	Humidification	Inhaled dose (%)		
					**$He–O_2$ 80/20**	**Air**	**p**
Goode,2001[1]	In vitro	MDI+spacer	Albuterol	No	42.4 ± 3.2	27.5±1.3	0.015
				Yes	26.3 ± 2.0	16.7 ± 2.3	0.02

MDI, metered dose inhaler; He-O2: helium and oxygen

1. Goode ML, Fink JB, Dhand R, Tobin MJ. Improvement in aerosol delivery with helium-oxygen mixtures during mechanical ventilation. Am J Respir Crit Care Med. 2001;163(1):109-114

Recommendations 1.24

Voting for recommendations 1.24

**Recommendations 1.24**	When heliox is substituted for oxygen to drive continuous jet nebulizer at the same driving flow, nebulizer output is reduced. If driving nebulizer with heliox, it is recommended to set at 15 L/min.
**Evidence**	In vitro[1]
	If you have additional evidence, please provide references:
**Likert score of 1-9**	□1 □2 □3 □4 □5 □6 □7 □8 □9<br>1= absolutely disagree, 9 = absolutely agree
**Comments**	
**Suggestions for future research**	

Table 1.24.The Study compared aerosol delivery in invasive ventilated patients with helium–oxygen mixtures and nitrogen-oxygen mixture.

**Author, year**	**Study type**	**Aerosol generator**	**Flow(L/min)**	**Nebulizer Output (μg of albuterol)**		
				**He–O_2 70/30**	**O_2**	**p**
Goode,2001[1]	In vitro	Continuous JN	5	10.1 ± 3.8	170.9 ± 6.1	<0.001
			10	177.4 ± 12.2	325.2 ± 14.8	<0.01
			15	633.3 ± 26.3		

JN, jet nebulizer; He-O_2: helium and oxygen

1. Goode ML, Fink JB, Dhand R, Tobin MJ. Improvement in aerosol delivery with helium-oxygen mixtures during mechanical ventilation. Am J Respir Crit Care Med. 2001;163(1):109-114

Recommendations 1.25

Voting for recommendations 1.25

**Recommendations 1.25**	Spontaneous breathing may help increase aerosol delivery efficiency, but it is not recommended to change the ventilator mode for the sole purpose of improving aerosol delivery efficiency.
**Evidence**	In-vitro[1,4], in vivo[2,3]
	If you have additional evidence, please provide references:
**Likert score of 1-9**	□1 □2 □3 □4 □5 □6 □7 □8 □9<br>1= absolutely disagree, 9 = absolutely agree
**Comments**	
**Suggestions for future research**	

Table1.25.Studies comparing of different mode and with/without spontaneous breathing

Author, year	Study	Populatio	Inhaled	Nebulizer	Mode and Finding/Aerosol deposition

	Type	n	medicati on	And Position						
Fink, 1996[1]	In vitro	Adult	Albuterol	MDI+Spacer	(non-humidification, RR 10/min, peak inspiratory flow 40 L/min, square-wave flow, trigger -2 cmH_2O	**CMV, VT 800ml**	**A/C, VT 800ml**	**PS 20, VT 800ml**	**CPAP, VT 800ml**	**P**
						30.3 ± 3.4%	31.9±1.3%	30.9 ± 1.8%	39.2 ± 1.4%	<0.01
Malliotakis, 2007[2]	In vivo	Adult: 10 COPD patients	Salbutam ol	MDI+Spacer, inspiratory limb	Tube diameter 8.0±0.5 mm, tube length 28±1 mm		**VC***		**PS[#]**	**P**
						Baseline	Rrs: 26.5±4.1<br>Rint:18.4± 4<br>PEEPi:8.4±3.2		Rrs:25.8±4.8<br>Rint:18.1±4.3<br>PEEPi:8±2.7	>0.05
						15min	Rrs:23.7±3.7<br>Rint: 15.9±3.5<br>PEEPi:7.2±3.5		Rrs:23±4<br>Rint:15.3±3.5<br>PEEPi:6.8±2.9	
						30min	Rrs:23.1±3.6<br>Rint:15.5±3.6<br>PEEPi:7.1±3.5		Rrs:22.4±4<br>Rint:14.9±3.7<br>PEEPi:6.9±3.2	
						60min	Rrs:23.3±4.3<br>Rint:15.1±3.8<br>PEEPi:7.1±3.5		Rrs:22.6±4<br>Rint:14.8±4<br>PEEPi:6.8±3.1	
						120min	Rrs:23.9±4.4<br>Rint:15.5±4<br>PEEPi:7.4±3.6		Rrs:23.1±3.6<br>Rint:15.6±4.1<br>PEEPi:7±3	
Dugernier J, 2016[3]	In vivo	Adult: 17 postoperati ve neurosurge ry patients	Diethyle netriamin epentaac etic acid labeled with technetiu m-99m	Vibrating mesh nebulizer, between the endotracheal tube and the catheter mount	PEEP 5 cmH_2O, base flow 10 L/min, no end-inspiratory pause, VT 8 ml/kg	**PS[&]**		**VC[^]**		**P**
						10.5 ± 3.0%		15.1 ± 5.0 %		0.038
Ge, 2019[4]	In vitro	Adult	Albuterol	Vibrating mesh nebulizer		**PCV_{BF0}**	**PCV_{BF6}**	**APRV**	**APRVs**	**P**
					Position 1: inspiratory limb at Y	15.9%	20.8%	9.5%	23.1%	<0.001

	Position 2: Humidifier outlet	19.4%	21.1%	17.9%	27.4%
	Position 3: Humidifier inlet	29.8%	23.6%	23.1%	34.1%

**VC*：volume control, VT 7~8 ml/kg, square wave flow, no end-inspiratory pause, zero PEEP, F_IO_2 achieved SpO_2 ＞89%；*

[#]PS：pressure support, pressure-support level (obtain a comparable VT), extrinsic PEEP set as 1~2 cm H_2O lower than PEEPi (measured on controlled mode), triggering 2 L/min.

[&]PS: pressure support ventilation, expiratory trigger was set to obtain an inspiratory/expiratory ratio of 30 %

[^]VC: volume-controlled ventilation, VE 8 L/min, inspiratory time and respiratory rate were then adjusted to ensure inspiratory/ expiratory ratio of 30 %, constant inspiratory flow 30 L/min.

PCV_{BF0}: pressure control ventilation with no bias flow.

PCV_{BF6}: PCV with bias flow of 6 L/min.

APRV: Airway pressure release ventilation (with no spontaneous breaths),

APRVs: APRV with spontaneous breath trigger.

MDI, metered-dose inhaler; VT, tidal volume; CMV, controlled mechanical ventilation; A/C, assist/control; PS: pressure support; CPAP, continuous positive airway pressure; COPD, chronic obstructive pulmonary disease; VC,volume control;PEEP: positive end-expiratory pressure (cmH_2O); PEEPi, intrinsic positive end-expiratory pressure (cmH_2O); Rrs and Rint, maximum and minimum inspiratory resistance, respectively, (cmH_2O/l/s).

1. Fink JB, Dhand R, Duarte AG, Jenne JW, Tobin MJ. Aerosol delivery from a metered-dose inhaler during mechanical ventilation. An in vitro model. Am J Respir Crit Care Med. 1996 Aug;154(2 Pt 1):382~7.
2. Malliotakis P, Mouloudi E, Prinianakis G, Kondili E, Georgopoulos D. Influence of respiratory efforts on b2-agonist induced bronchodilation in mechanically ventilated COPD patients: a prospective clinical study. Respir Med. 2007 Feb;101(2):300-7.
3. Dugernier J, Reychler G, Wittebole X, Roeseler J, Depoortere V, Sottiaux T, Michotte JB, Vanbever R, Dugernier T, Goffette P, Docquier MA, Raftopoulos C, Hantson P, Jamar F, Laterre PF, (2016) Aerosol delivery with two ventilation modes during mechanical ventilation: a randomized study. Ann Intensive Care 6: 73
4. Ge HQ, Wang JM, Lin HL, Fink JB, Luo R, Xu P, Ying K. Effect of Nebulizer Location and Spontaneous Breathing on Aerosol Delivery During Airway Pressure Release Ventilation in Bench Testing. J Aerosol Med Pulm Drug Deliv. 2019 Feb;32(1):34-39.

Recommendations 1.26

Voting for recommendations 1.26

**Recommendations 1.26**	When metered-dose inhaler is utilized during invasive mechanical ventilation, there is no recommendation on flow trigger vs pressure trigger solely for aerosol delivery.	
**Evidence**	In-vitro[1]	
	If you have additional evidence, please provide references:	
**Likert score of 1-9**	□1 □2 □3 □4 □5 □6 □7 □8 □9<br>1= absolutely disagree, 9 = absolutely agree	
**Comments**		
**Suggestions for future research**		

Table1.26.Studies of different type of trigger

Author, year	Study Type	Population	Inhaled medication	Nebulizer And Position	Different type of trigger and Finding/Aerosol deposition			
						Flow-triggered	Pressure-triggered	P
Fink, 1996[1]	In vitro	Adult	Albuterol	MDI+Spacer	PS 10, VT 700 ml	354.5 ± 20.3 ug	311.3 ± 48.4 ug	> 0.05
					CPAP, VT 500 ml	251.0 ± 34.4 ug	336.7 ± 40.3 ug	0.08

MDI, metered-dose inhaler; PS, pressure support; VT, tidal volume; CPAP, continuous positive airway pressure

1. Fink JB, Dhand R, Duarte AG, Jenne JW, Tobin MJ. Aerosol delivery from a metered-dose inhaler during mechanical ventilation. An in vitro model. Am J Respir Crit Care Med. 1996 Aug;154(2 Pt 1):382~7.

Recommendations 1.27

Voting for recommendations 1.27

**Recommendations 1.27**	It is not recommended to change ventilation parameters for the sole purpose of improving aerosol delivery efficiency.
**Evidence**	In-vivo[3] or in-vitro[1,2,4]
	If you have additional evidence, please provide references:
**Likert score of 1-9**	□1 □2 □3 □4 □5 □6 □7 □8 □9<br>1= absolutely disagree, 9 = absolutely agree
**Comments**	
**Suggestions for future research**	

Table1.27.Studies of change ventilation parameters

Author, year	Study Type	Population	Inhaled medication	Nebulizer And Position	Comparison and Finding/Aerosol deposition					
O'Doherty, 1992[1]	In vitro	Adults	99mtc HSA	Inspiratory synchronized JN	RR	**8/min**	**12/min**	**15/min**	**20/min**	**P**
						8.3 ± 0.78%*	11.2 ± 1.6%*	5.4 ± 0.15%	5.12 ± 0.14%	*p < 0.05
					MV	**6 L/min**	**9 L/min**	**12 L/min**	**15 L/min**	
						9.9 ± 0.9%*	5.4 ± 0.15%	5.8 ± 0.27%	3.75 ± 0.46%*	
Fink, 1996[2]	In vitro	Adult	Albuterol	MDI+Spacer	VT (CPAP)	**VT 800ml**	**VT 500ml**	**VT 300ml**	**VT 100ml**	**P**
						39.2 ± 1.4%	31.2±3.7%	21.6 ± 3.9%	4.9± 1.4%	< 0.003
Mouloudi E，1999[3]	In vivo	Adult: 9 COPD	Salbutamol	MDI+Spacer, inspiratory limb	8–9 mm tube, square wave, inspiratory flow 0.55~0.65 l/s and no end-inspiratory pause, individual MV, PEEP 0 cmH_2O		**VT 8 ml/kg**	**VT 12 ml/kg**		**P**
						Baseline	Rrs: 22.7±4.3<br>Rint: 18.2±3.2	Rrs: 21.4±3.4<br>Rint: 16.8±2.1		>0.05
						15min	Rrs: 19.9±2.60<br>Rint: 14.9±2.2	Rrs: 19.1±2.4<br>Rint: 14.1±2.0		
						30min	Rrs: 19.1±2.9<br>Rint: 14.4±2.1	Rrs: 19.3±2.6<br>Rint: 14.2±2.0		
						60min	Rrs: 19.7±3.7<br>Rint: 14.7±2.6	Rrs: 18.8±3.0<br>Rint: 14.5±2.7		
Williams,1999[4]	In vitro	Adult	Saline 0.9%	Ultrasonic nebulizer, inspiratory limb	Inspiratory flow 10.6L/min and inspiratory time 1.67s was constant	**MV 3.6 L/min (6*0.6 L)**		**MV 7.2 L/min (12* 0.6 L)**		**P**
					Nebulizer set at '1'#	3.2±0.8 ml		5.5±1.0 ml		＜0.05
					Nebulizer set at '2' #	6.7±0.4 ml		9.5±0.2 ml		< 0.001
					Nebulizer set at '3' #	8.2±0.6 ml		15.3±0.7 ml		< 0.001
					I:E ratio of 1:2 was constant	**MV 3.6 L/min (6*0.6 L), inspiratory flow 25.2 L/min, inspiratory time 1.67s**		**MV 7.2 L/min (12* 0.6 L), inspiratory flow 10.6 L/min, inspiratory time 3.33s**		**P**
					Nebulizer set at '1'#	4.9±0.3 ml		4.6±0.6 ml		＞0.05
					Nebulizer set at '2' #	5.9±1.0 ml		7.5±0.7 ml		＞0.05
					Nebulizer set at '3' #	9.20±0.6 ml		10.0±0.2 ml		＞0.05

**p < 0.05 compared with the default settings (RR 15/min, MV 9 L/min. Inspiratory time 25%)*

# Nebulizer set at '1': low output, Nebulizer set at '2': medium output, Nebulizer set at '3': full output.

99mtc HAS, technetium~99m human serum albumin; JN, jet nebulizer; MV, minute ventilation; RR, respiratory rates; MDI, metered-dose inhaler; VT, tidal volume; CPAP, continuous positive airway pressure; COPD, chronic obstructive pulmonary disease; PEEP, positive end-expiratory pressure; Rrs and Rint: maximum and minimum inspiratory resistance, respectively ($cmH_2O/l/s$); I: E inspiration to expiration ratio.

1. O'Doherty MJ, Thomas SH, Page CJ, Treacher DF, Nunan TO. Delivery of a nebulized aerosol to a lung model during mechanical ventilation. Effect of ventilator settings and nebulizer type, position, and volume of fill. Am Rev Respir Dis. 1992 Aug;146(2):383~8.
2. Fink JB, Dhand R, Duarte AG, Jenne JW, Tobin MJ. Aerosol delivery from a metered-dose inhaler during mechanical ventilation. An in vitro model. Am J Respir Crit Care Med. 1996 Aug;154(2 Pt 1):382~7.
3. Mouloudi E, Katsanoulas K, Anastasaki M, Hoing S, Georgopoulos D, (1999) Bronchodilator delivery by metered-dose inhaler in mechanically ventilated COPD patients: influence of tidal volume. Intensive Care Med 25: 1215~1221
4. Williams L, Fletcher GC, Daniel M, Kinsella J. A simple in vitro method for the evaluation of an ultrasonic nebulizer for drug delivery to intubated, ventilated patients and the effect of nebulizer and ventilator settings on the uptake of fluid from the nebulizer chamber. Eur J Anaesthesiol. 1999 Jul;16(7):479~84.

Recommendations 1.28

Voting for recommendations 1.28

**Recommendations 1.28**	Longer inspiratory time and lower inspiratory flows are associated with improved aerosol delivery efficiency, however, changing those parameters solely for aerosol delivery is not recommended.	
**Evidence**	In-vitro[1-6]	
	If you have additional evidence, please provide references:	
**Likert score of 1-9**	□1 □2 □3 □4 □5 □6 □7 □8 □9<br>1= absolutely disagree, 9 = absolutely agree	
**Comments**		
**Suggestions for future research**		

Table1.28.Studies of change inspiratory flow and inspiratory time

Author, year	Study Type	Population	Inhaled medication	Nebulizer And Position	Comparison and Finding/Aerosol deposition				
O'Doherty, 1992[1]	In vitro	Adults	99mtc HSA	Inspiratory synchronized JN	**Inspiratory time**				
					**20%**	**25%**	**33%**	**50%**	**P**
					5.0 ± 0.84%	5.4 ± 0.15%	13.3 ± 0.12%*	17.4 ± 0.40%*	*p < 0.05.
Fink, 1996[2]	In vitro	Adult	Albuterol	MDI+Spacer	**Ti/Ttot (CMV, AC, PS)**				
					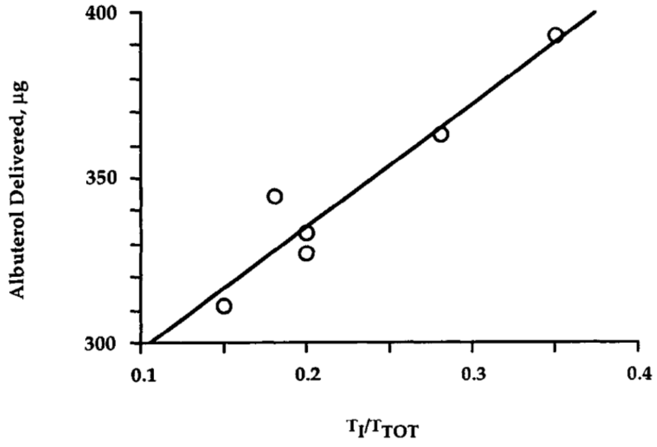<br><br>*Figure 2.* Correlation between albuterol delivery and duty cycle (TI/Ttot) after administration of 12 puffs with controlled mechanical ventilation (VT, 800 and 500 ml), assist control (VT, 800 ml), and pressure support of 10 and 20 cm H_2O (VT, 700 and 800 ml, respectively). Albuterol delivery was linearly related to TI/Ttot (r = 0.96, p < 0.003).<br>Albuterol delivery was linearly related to Ti/Ttot (r =0.96, P < 0.003)				
Fink, 1999[3]	In vitro	Adult	Albuterol	MDI+Spacer			**Ti/Ttot : 0.5**	**Ti/Ttot : 0.25**	**P**
					Inspiratory flow 40L/min		27.4±0.4%	24.1±0.7%	＜0.002
					Inspiratory flow 80L/min		10.2±0.1%	9.4± 0.0%	＜0.04
					Ti/Ttot constant		**Inspiratory flow 40L/min**	**Inspiratory flow 80L/min**	**P**
							25.8±0.6%	9.8 ±0.1%	＜0.0001
Williams,1999[4]	In vitro	Adult	Saline 0.9%	Ultrasonic nebulizer, inspiratory limb	MV:7.2L/min (12*0.6L)		**Inspiratory flow 34.3 L/min, I: E 1:4**	**Inspiratory flow 8.4 L/min, I: E 4:1**	**P**
					Nebulizer set at '1'#		3.2±0.3 ml	2.5±0.8 ml	> 0.05

				Nebulizer set at '2' #	6.4±0.7 ml	11.0±0.6 ml	< 0.01
				Nebulizer set at '3' #	9.1±0.6 ml	18.7±0.2 ml	< 0.001

					VT 0.6 L, RR 15/min, PEEP 5 cmH_2O		**Inspiratory times 1s**	**Inspiratory times 2s**	**P**
Hess, 2003[5]	In vitro	Adult	Albuterol	Inspiratory synchronized JN	VCV, constant flow	Long time constant	0.17±0.01mg	0.59±0.05 mg	<0.001
						Short time constant	0.14±0.01 mg	0.41±0.01 mg	
					VCV, descending ramp flow	Long time constant	0.17±0.04 mg	0.43±0.07 mg	
						Short time constant	0.13±0.01 mg	0.50±0.02 mg	
					PCV	Long time constant	0.34±0.02 mg	0.59±0.05 mg	
						Short time constant	0.10±0.01 mg	0.09±0.01 mg	
				MDI+Spacer	VCV, constant flow	Long time constant	0.20±0.01 mg	0.18±0.02 mg	0.37
						Short time constant	0.23±0.01 mg	0.22±0.01 mg	
					VCV, descending ramp flow	Long time constant	0.20±0.01 mg	0.20±0.03 mg	
						Short time constant	0.20±0.01 mg	0.21±0.01 mg	
					PCV	Long time constant	0.21±0.01 mg	0.24±0.01 mg	
						Short time constant	0.20±0.01 mg	0.21±0.01 mg	

						**Inspiratory flow**			**P**
Vecellio, 2005[6]	In vitro	Adult	Terbutaline	Continuous JN, inspiratory line	VT 500ml, RR 15/min	Ti/Ttot 0.2, inspiratory flow 37.5	Ti/Ttot 0.3, inspiratory flow 25.0	Ti/Ttot 0.4, inspiratory flow 18.8	Ti/Ttot 0.5, inspiratory flow 15.0	p<0.0001

		L/min	L/min	L/min	L/min
		35 (28–37) %	37 (36–41) %	37 (36–40) %	42 (42–44) %
	VT 600ml, RR 12/min	Ti/Ttot 0.2, inspiratory flow 36.0 L/min	Ti/Ttot 0.3, inspiratory flow 24.0 L/min	Ti/Ttot 0.4, inspiratory flow 18.0 L/min	Ti/Ttot 0.5, inspiratory flow 14.4 L/min
		39 (38–42) %	40 (39–42) %	43 (42–45) %	44 (44–48) %

**p < 0.05 compared with the default settings (RR 15/min, MV 9 L/min. Inspiratory time 25%)*

#Nebulizer set at ‘1’: low output, Nebulizer set at ‘2’: medium output, Nebulizer set at ‘3’: full output.

99mtc HAS, technetium~99m human serum albumin; JN, jet nebulizer; MV, minute ventilation; RR, respiratory rates;MDI, metered-dose inhaler; CMV, controlled mechanical ventilation; A/C, assist/control; PS, pressure support; Ti, inspiratory time; Ttot, total time; I:E, inspiration to expiration ratio; PEEP, positive end-expiratory pressure; VT, tidal volume; VCV, volume control ventilation; PCV, pressure control ventilation;VMD, volume median diameter.

1. O'Doherty MJ, Thomas SH, Page CJ, Treacher DF, Nunan TO. Delivery of a nebulized aerosol to a lung model during mechanical ventilation. Effect of ventilator settings and nebulizer type, position, and volume of fill. Am Rev Respir Dis. 1992 Aug;146(2):383~8.
2. Fink JB, Dhand R, Duarte AG, Jenne JW, Tobin MJ. Aerosol delivery from a metered-dose inhaler during mechanical ventilation. An in vitro model. Am J Respir Crit Care Med. 1996 Aug;154(2 Pt 1):382~7.
3. Fink JB, Dhand R, Grychowski J, Fahey PJ, Tobin MJ. Reconciling in vitro and in vivo measurements of aerosol delivery from a metered-dose inhaler during mechanical ventilation and defining efficiency-enhancing factors. Am J Respir Crit Care Med. 1999 Jan;159(1):63~8.
4. Williams L, Fletcher GC, Daniel M, Kinsella J. A simple in vitro method for the evaluation of an ultrasonic nebulizer for drug delivery to intubated, ventilated patients and the effect of nebulizer and ventilator settings on the uptake of fluid from the nebulizer chamber. Eur J Anaesthesiol. 1999 Jul;16(7):479~84.
5. Hess DR, Dillman C, Kacmarek RM. In vitro evaluation of aerosol bronchodilator delivery during mechanical ventilation: pressure-control vs. Volume control ventilation. Intensive Care Med. 2003 Jul;29(7):1145-50.
6. Vecellio L, Guérin C, Grimbert D, De Monte M, Diot P. In vitro study and semiempirical model for aerosol delivery control during mechanical ventilation. Intensive Care Med. 2005 Jun;31(6):871-6.

Recommendations 1.29

Voting for recommendations 1.29

**Recommendations 1.29**	It is not recommended to change the inspiratory flow patterns solely for aerosol delivery.	
**Evidence**	In-vitro[1,3,4] and in-vivo[2]	
	If you have additional evidence, please provide references:	
**Likert score of 1-9**	□1 □2 □3 □4 □5 □6 □7 □8 □9<br>1= absolutely disagree, 9 = absolutely agree	
**Comments**		
**Suggestions for future research**		

Table1.29.Studies of different inspiratory flow pattern

Author, year	Study Type	Population	Inhaled medication	Nebulizer And Position	Comparison and Finding/Aerosol deposition					
Fink, 1996[1]	In vitro	Adult	Albuterol	MDI+spacer	Inspiratory flow pattern (CMV, VT 800 ml)		Sinusoidal flow	Decelerating flow	Square flow	P
							362.9 ± 40.4 ug	392.9 ± 33.9 ug	327.3 ± 36.8 ug	< 0.01
Mouloudi, 2000[2]	In vivo	Adult: 18 COPD	Salbutamol	MDI+Spacer, inspiratory limb	8–9 mm tube, Individual MV, no end-inspiratory pause, PEEP 0 cmH_2O		Volume control*	Pressure control[#]		P
						Baseline	Rrs: 26.3±2.9 Rint: 21.0±2.4	Rrs: 26.3±2.9 Rint: 21.2±3.3		>0.05
						15min	Rrs: 22.8±3.6 Rint: 17.9±3.3	Rrs: 22.4±3.3 Rint: 17.6±3.2		
						30min	Rrs: 22.8±2.7 Rint: 17.9±2.6	Rrs: 22.8±2.8 Rint: 17.9±2.6		
						60min	Rrs: 22.8±2.7 Rint: 17.9±2.6	Rrs: 23.7±3.2 Rint: 18.5±2.9		
Hess, 2003[3]	In vitro	Adult	Albuterol		VT 0.6 L, RR 15/min, PEEP 5 cmH_2O		VCV, constant flow	VCV, descending ramp flow	PCV	P
				Inspiratory synchronized JN	Long time constant	Ti: 1s	0.17±0.01mg	0.17±0.04 mg	0.34±0.02 mg	0.03
						Ti:2s	0.59±0.05 mg	0.43±0.07 mg	0.59±0.05 mg	
					Short time constant	Ti: 1s	0.14±0.01 mg	0.13±0.01 mg	0.10±0.01 mg	
						Ti:2s	0.41±0.01 mg	0.50±0.02 mg	0.09±0.01 mg	
				MDI+Spacer	Long time constant	Ti: 1s	0.20±0.01 mg	0.20±0.01 mg	0.21±0.01 mg	0.37
						Ti:2s	0.18±0.02 mg	0.20±0.03 mg	0.24±0.01 mg	
					Short time constant	Ti: 1s	0.23±0.01 mg	0.20±0.01 mg	0.20±0.01 mg	
						Ti:2s	0.22±0.01 mg	0.21±0.01 mg	0.21±0.01 mg	
Dugernier,	In vitro	Adult	Amikacin	Vibrating mesh	Decelerating inspiratory flow vs constant flow pattern, with or without proximal flow sensor					

2015[4] nebulizer

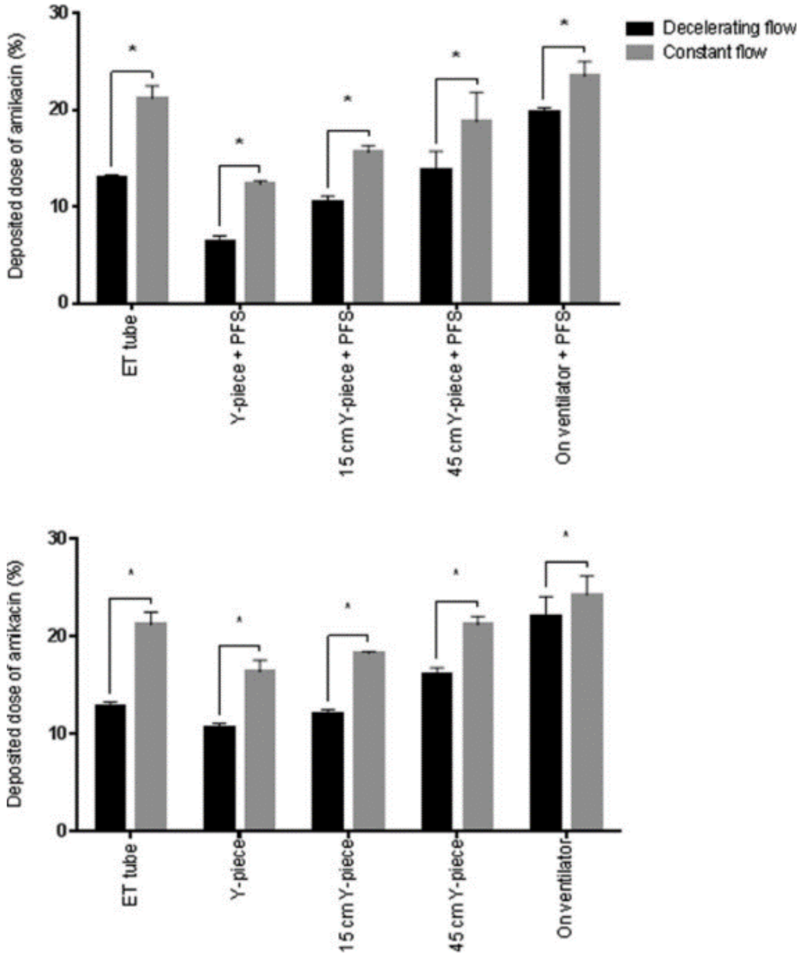

$P < 0.05$

**Volume control: VT 8 ml/kg, square wave flow, inspiratory flow 0.55–0.65 L/s;*

#Pressure control, the pressure level (achieve a VT 8 ml/kg), inspiratory time was kept constant.

pMDI, pressurized metered-dose inhaler; JN, jet nebulizer; VT, tidal volume; CMV, controlled mechanical ventilation; COPD, chronic obstructive pulmonary disease; MV, minute ventilation; PEEP, positive end-expiratory pressure; Rrs and Rint, maximum and minimum inspiratory resistance, respectively, ($cmH_2O/l/s$); RR,respiratory rates; PEEP, positive end-expiratory pressure; VCV, volume control ventilation;PCV, pressure control ventilation.

1. Fink JB, Dhand R, Duarte AG, Jenne JW, Tobin MJ. Aerosol delivery from a metered-dose inhaler during mechanical ventilation. An in vitro model. Am J Respir Crit Care Med. 1996

Aug;154(2 Pt 1):382~7.

2. Mouloudi E, Prinianakis G, Kondili E, Georgopoulos D, (2000) Bronchodilator delivery by metered-dose inhaler in mechanically ventilated COPD patients: influence of flow pattern. The European respiratory journal 16: 263~268
3. Hess DR, Dillman C, Kacmarek RM. In vitro evaluation of aerosol bronchodilator delivery during mechanical ventilation: pressure-control vs. Volume control ventilation. Intensive Care Med. 2003 Jul;29(7):1145-50.
4. Dugernier J, Wittebole X, Roeseler J, Michotte JB, Sottiaux T, Dugernier T, Laterre PF, Reychler G. Influence of inspiratory flow pattern and nebulizer position on aerosol delivery with a vibrating-mesh nebulizer during invasive mechanical ventilation: an in vitro analysis. J Aerosol Med Pulm Drug Deliv. 2015 Jun;28(3):229-36.

Recommendations 1.30

Voting for recommendations 1.30

**Recommendations 1.30**	It is not recommended to apply end-inspiratory pause when metered dose inhaler is used during invasive mechanical ventilation.
**Evidence**	In-vivo[1]
	If you have additional evidence, please provide references:
**Likert score of 1-9**	□1 □2 □3 □4 □5 □6 □7 □8 □9<br>1= absolutely disagree, 9 = absolutely agree
**Comments**	
**Suggestions for future research**	

Table1.30.Studies of with or without end-inspiratory pause

<table>
<tr><th rowspan="2">Author, year</th><th rowspan="2">Study Type</th><th rowspan="2">Population</th><th rowspan="2">Inhaled medication</th><th rowspan="2">Nebulizer and position</th><th colspan="5">Comparison and Finding/Aerosol deposition (%)</th></tr>
<tr></tr>
<tr><td rowspan="5">Mouloudi E，1998[1]</td><td rowspan="5">In vivo</td><td rowspan="5">Adult:
12 COPD</td><td rowspan="5">Salbutamol</td><td rowspan="5">MDI+Spacer, inspiratory limb</td><td rowspan="5">8–9 mm tube, specific VT, square wave, individual MV, PEEP 0 cmH_2O</td><td></td><td>Without EIP 5 s</td><td>With EIP 5 s</td><td>P</td></tr>
<tr><td>Baseline</td><td>Rmin:10.7±3.0
Rmax: 18.4±3.0</td><td>Rmin:11.9±2.8
Rmax: 18.2±4.3</td><td rowspan="4">>0.05</td></tr>
<tr><td>15min</td><td>Rmin:10.7±3.0
Rmax: 18.4±3.0</td><td>Rmin:11.9±2.8
Rmax: 18.2±4.3</td></tr>
<tr><td>30min</td><td>Rmin:10.4±2.1
Rmax: 17.8±2.8</td><td>Rmin:12.3±2.8
Rmax: 20.0±3.2</td></tr>
<tr><td>60min</td><td>Rmin:11.9±2.6
Rmax: 18.0±2.5</td><td>Rmin:12.4±2.5
Rmax: 19.5±2.7</td></tr>
</table>

COPD, chronic obstructive pulmonary disease; MDI, metered-dose inhaler; VT, tidal volume; MV, minute ventilation; PEEP, positive end-expiratory pressure; EIP, end-inspiratory pause; Rmin and Rmax, minimum and maximum inspiratory resistance.

1. Mouloudi E, Katsanoulas K, Anastasaki M, Askitopoulou E, Georgopoulos D, (1998) Bronchodilator delivery by metered-dose inhaler in mechanically ventilated COPD patients: influence of end-inspiratory pause. The European respiratory journal 12: 165~169

Recommendations 1.31

Voting for recommendations 1.31

**Recommendations 1.31**	It is not recommended to change the positive end-expiratory pressure (PEEP) for the sole purpose of improving aerosol delivery efficiency.
**Evidence**	In vitro[1,3] and in vivo[2]
	If you have additional evidence, please provide references:
**Likert score of 1-9**	□1 □2 □3 □4 □5 □6 □7 □8 □9<br>1= absolutely disagree, 9 = absolutely agree
**Comments**	
**Suggestions for future research**	

Table1.31.Studies of change of PEEP

<table>
<tr><th>Author, year</th><th>Study Type</th><th>Population</th><th>Inhaled medication</th><th>Nebulizer And Position</th><th colspan="7">Comparison and Finding/Aerosol deposition</th></tr>
<tr><td rowspan="3">Williams,1999[1]</td><td rowspan="3">In vitro</td><td rowspan="3">Adult</td><td rowspan="3">Saline 0.9%</td><td rowspan="3">Ultrasonic nebulizer, inspiratory limb</td><td colspan="7">MV:7.2L/min(12*0.6L)，I:E 1:2, PEEP: 0 vs 5 vs 10</td></tr>
<tr><td colspan="7">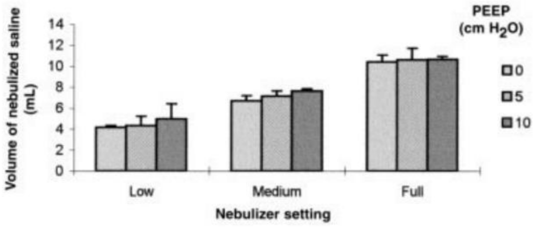

Fig. 2. The effect of positive end expiratory pressure upon the function of the DeVilbiss Ultra-Neb 2000 to nebulize 0.9% saline over a period of 5 min.</td></tr>
<tr><td colspan="7">P> 0.05</td></tr>
<tr><td rowspan="7">Guérin, 2005[2]</td><td rowspan="7">In vivo</td><td rowspan="7">Adult: 10 COPD patients</td><td rowspan="7">Fenoterol</td><td rowspan="7">Inspiratory synchronized JN</td><td colspan="2"></td><td colspan="2">ZEEP</td><td colspan="2">PEEPe (80% PEEPt)</td><td>P</td></tr>
<tr><td colspan="2">Pre PEEPt</td><td colspan="2">8±3 cmH$_2$O</td><td colspan="2">9±3 cmH$_2$O</td><td rowspan="2"><0.05</td></tr>
<tr><td colspan="2">60min PEEPt</td><td colspan="2">6±3 cmH$_2$O</td><td colspan="2">8±3 cmH$_2$O</td></tr>
<tr><td colspan="2">Pre △FRC</td><td colspan="2">0.61±0.34 L</td><td colspan="2">0.62±0.34 L</td><td rowspan="2"><0.05</td></tr>
<tr><td colspan="2">60min △FRC</td><td colspan="2">0.43±0.32 L</td><td colspan="2">0.62±0.37 L</td></tr>
<tr><td colspan="2">Pre Rrs</td><td colspan="2">26±7 cmH$_2$O/L/s</td><td colspan="2">26±9 cmH$_2$O/L/s</td><td rowspan="2">＞0.05</td></tr>
<tr><td colspan="2">60min Rrs</td><td colspan="2">23±6 cmH$_2$O/L/s</td><td colspan="2">25±9 cmH$_2$O/L/s</td></tr>
<tr><td rowspan="4">Vecellio, 2005[3]</td><td rowspan="4">In vitro</td><td rowspan="4">Adult</td><td rowspan="4">Terbutaline</td><td rowspan="4">Continuous JN, inspiratory line</td><td rowspan="4">VT 600 ml RR 12/min, Ti/Ttot 0.5</td><td rowspan="2">10 mg/4 ml of terbutaline</td><td>PEEP 0 cmH$_2$O</td><td>PEEP 2 cmH$_2$O</td><td>PEEP 4 cmH$_2$O</td><td>PEEP 6 cmH$_2$O</td><td>P</td></tr>
<tr><td>44%</td><td>44%</td><td>44%</td><td>46%</td><td>=0.66</td></tr>
<tr><td rowspan="2">20 mg/8 ml of terbutaline</td><td>PEEP 0 cmH$_2$O</td><td>PEEP 2 cmH$_2$O</td><td>PEEP 4 cmH$_2$O</td><td>PEEP 6 cmH$_2$O</td><td>P</td></tr>
<tr><td>52%</td><td>50%</td><td>49%</td><td>52%</td><td>=0.22</td></tr>
</table>

JN, jet nebulizer; MV, minute ventilation; I:E, inspiration to expiration ratio; PEEP, positive end-expiratory pressure;COPD, chronic obstructive pulmonary disease; ZEEP,zero end-expiratory pressure;PEEPe,external positive end-expiratory pressure;PEEPt, total positive end-expiratory pressure; △FRC, change in end-expiratory lung volume; Rrs, inspiratory resistance;VT, tidal volume;RR, respiratory rates;Ti,inspiratory time; VMD, volume median diameter.

1. Williams L, Fletcher GC, Daniel M, Kinsella J. A simple in vitro method for the evaluation of an ultrasonic nebulizer for drug delivery to intubated, ventilated patients and the effect of nebulizer and ventilator settings on the uptake of fluid from the nebulizer chamber. Eur J Anaesthesiol. 1999 Jul;16(7):479~84.

2. Guérin C, Durand PG, Pereira C, Richard JC, Poupelin JC, Lemasson S, Badet M, Philit F, Vecellio L, Chantrel G. Effects of inhaled fenoterol and positive end-expiratory pressure on the respiratory mechanics of patients with chronic obstructive pulmonary disease. Can Respir J. 2005 Sep;12(6):329-35.
3. Vecellio L, Guérin C, Grimbert D, De Monte M, Diot P. In vitro study and semiempirical model for aerosol delivery control during mechanical ventilation. Intensive Care Med. 2005 Jun;31(6):871-6.

Recommendations 1.32

Voting for recommendations 1.32

**Recommendations 1.32**	With nebulizer placed proximal to patient, higher bias flow is associated with lower aerosol delivery efficiency. With nebulizer placed proximal to ventilator, adding bias flow up to 5 L/min improves delivery. It is recommended to set bias flow up to 5 L/min when nebulizer is placed proximal to ventilator.
**Evidence**	In vitro[1,2]
	If you have additional evidence, please provide references:
**Likert score of 1-9**	□1 □2 □3 □4 □5 □6 □7 □8 □9<br>1= absolutely disagree, 9 = absolutely agree
**Comments**	
**Suggestions for future research**	

Table1.32.Studies of change of bias flow

Author,	Study	Populatio	Inhaled	Nebulizer	Comparison and Finding/Aerosol deposition (%)

year	Type	n	medication	And Position					
Miller, 2003[1]	In vitro	Adult	Albuterol	Continuous JN placed in the inspiratory line 12 inches before the Y piece	VT 750 ml, RR 15/min, peak flow 70 L/minute, Ti 0.9s, I: E 1:3.4	Bias flow 10 L/minute	Bias flow 15 L/minute	Bias flow 20 L/minute	P
					Non humidified	10.0±0.7	8.3±1.0	8.6±0.7	＞0.05
					Humidified	5.3±0.4	5.3±0.5	3.8±0.8	
Ari, 2010[2,3]	In vitro	Adult	Albuterol		VT 500 mL, PEEP 5 cm H_2O, RR 20 /min, peak inspiratory flow 60 L/min, descending ramp flow, humidified	Bias flow 0 L/min	Bias flow 2 L/min	Bias flow 5 L/min	P
				Continuous JN	Position 1 15 cm from Y-piece	3.6±0.2	4.7±0.1	4.0±0.1	NR
					Position 2 Prior to the humidifier	6.0±0.1	5.2±0.2	4.7±0.4	
				Vibrating mesh nebulizer	Position 1 Between the Y-piece and the circuit	16.8±2.6	13.4±1.1	9.7±0.6	
					Position 2 Inlet to the humidifier	8.4±2.1	23.8±1.0	21.4±0.4	

VT, tidal volume; RR, respiratory rates; Ti, inspiratory time; I: E ,inspiration to expiration ratio; PEEP, positive end-expiratory pressure.

1. Miller DD, Amin MM, Palmer LB, Shah AR, Smaldone GC. Aerosol delivery and modern mechanical ventilation: in vitro/in vivo evaluation. Am J Respir Crit Care Med. 2003 Nov 15;168(10):1205-9.
2. Ari A, Areabi H, Fink JB. Evaluation of aerosol generator devices at 3 locations in humidified and non-humidified circuits during adult mechanical ventilation. Respir Care. 2010 Jul;55(7):837-44.
3. Ari A, Atalay OT, Harwood R, Sheard MM, Aljamhan EA, Fink JB. Influence of nebulizer type, position, and bias flow on aerosol drug delivery in simulated pediatric and adult lung models during mechanical ventilation. Respir Care. 2010 Jul;55(7):845-51.

Section 2

Aerosol Delivery via Non-invasive Ventilation for Adult Patients

Recommendations 2.1

Voting for recommendations 2.1

**Recommendations 2.1**	Placing the nebulizer in-line with noninvasive ventilation has similar or higher aerosol delivery efficiency than using the nebulizer with a mask or mouthpiece. Interrupting or discontinuing noninvasive ventilation to administer aerosol via a mask or mouthpiece is not recommended.
**Evidence**	In vivo [1,3-10], in vitro[2,9]
	If you have additional evidence, please provide references:
**Likert score of 1-9**	□1 □2 □3 □4 □5 □6 □7 □8 □9<br>1= absolutely disagree, 9 = absolutely agree
**Comments**	
**Suggestions for future research**	

Table 2.1.1. Studies comparing the inhaled dose via aerosol delivery via NIV (Experimental group) vs traditional nebulization via mask/mouthpiece (Control group) among healthy volunteers

Author, year	Study type	Population	Inhaled medication	Ventilator setting	Nebulizer	Measurement				
Franca,2006[1]	In-vivo	Adult (healthy volunteers)	^{99m}Tc—DTPA	BiPAP IPAP=12cm H_2O, EPAP =5cm H_2O	cJN		Control	BiPAP		P
						Lung deposition(counts)	200,510+11,012	106,093+2811		< 0.001
Calvert,2006[2]	In-vitro	Adult(healthy volunteers)	salbutamol	BiPAP IPAP=20cmH_2O, EPAP =5cmH_2O	cJN		Control	BiPAP		P
						Filter deposited（μg）	424±61	647±67		< 0.01
Reychler,2007[3]	In-vivo	Adult (healthy volunteers)	amikacin	CPAP=6 cm H_2O	cJN		Control	CPAP		P
						Residual amount（mg）	541	607		NS
						Aerosol in the urine(mg)	52.11±4.18	21.07±2.95		< 0.001
Maccari, 2014[4]	In-vivo	Adult (healthy volunteers)	^{99m}Tc	BiPAP: IPAP=15cmH_2O, EPAP =5cmH_2O; CPAP=10cmH_2O	cJN		Control	Experimental group		P
								BiPAP	CPAP	
						Right lung deposition（counts）	196.6±167	111.5 ±15	108.7±4	NS.
						Left lung deposition（counts）	225.0 ±293	98.4 ±14	0	NS.
						Trachea deposition（counts）	39.8 ± 26		92.7±15	NS.
								28.3±19	29.8±25	

IPAP, inspiratory positive airway pressure; EPAP, expiratory positive airway pressure; CPAP, continuous positive airway pressure; NIV, noninvasive ventilation; cJN,contunuus jet nebulizer; NS: not significant.

1. Franca E.E.T,Andrade A,Cabral G,et al. Nebulization associated with Bi-level noninvasive ventilation: Analysis of pulmonary radioaerosol deposition[J]. Respiratory Medicine, 2006, 100(4):721-728.
2. Calvert LD, Jackson JM, White J A , et al. Enhanced delivery of nebulised salbutamol during non-invasive ventilation[J]. Journal of Pharmacy and Pharmacology, 2006, 58(11):1553-1557.
3. Reychler G , Leal T , Roeseler J , et al. Effect of continuous positive airway pressure combined to nebulization on lung deposition measured by urinary excretion of amikacin[J]. Respiratory Medicine, 2007, 101(10):2051-2055.
4. Maccari J G , Teixeira C , Savi A , et al. Nebulization During Spontaneous Breathing, CPAP, and Bi-Level Positive-Pressure Ventilation: A Randomized Analysis of Pulmonary Radioaerosol Deposition[J]. Respiratory Care, 2014, 59(4):479.

Table 2.1.2. Studies comparing the inhaled dose via aerosol delivery via NIV(Experimental group) vs traditional nebulization via mask/mouthpiece (Control group) among patients with asthma

Author,year	Study type	Population	Inhaled medication	Ventilator setting	Nebulizer	Measurement

Pollack,1995[5]	In-vivo	Adult with acute bronchospasma	albuterol	BiPAP :<br>IPAP=10cmH_2O<br>EPAP=5cm H_2O.	cJN		Control group	Experimental Group	P
						PEFR (L/min)	279.6±67.3	357.9±108.2	=0.0001
						PPEFR %	57.2±21.0	68.8±18.9	=0.0011
Parkes.1997[6]	In-vivo	Adult with stable asthma	salbutamol	CPAP=10cm H_2O.	cJN		Control group	Experimental Group	P
						Inhaled aerosol,%	6.85±1.52	1.3±0.37	< 0.01
Brandao,2009[7]	In-vivo	Adult with acute exacerbation of asthma	fenoterol and ipratropium	BiPAP :<br>IPAP=15cmH_20<br>EPAP=5cmH_20;<br>IPAP=15cmH_20<br>EPAP=10cmH_20	cJN	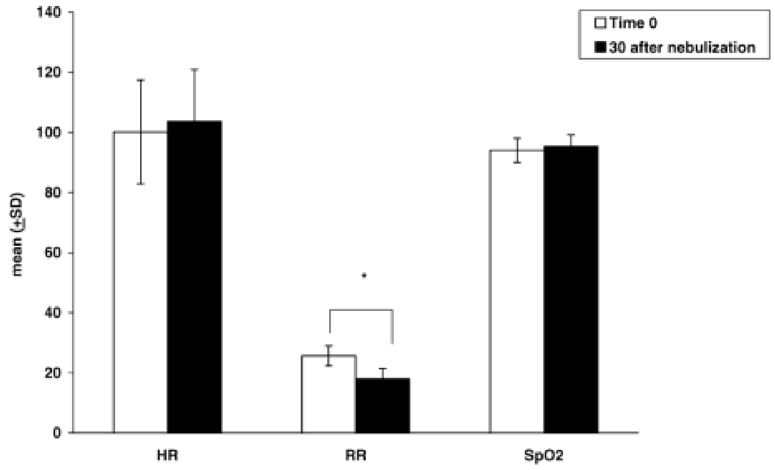			

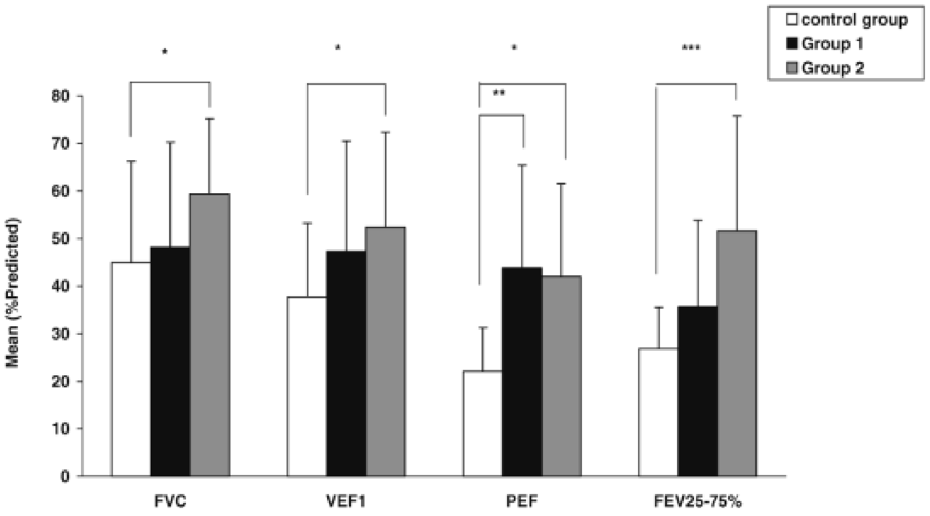

(*p = 0.04) (*p = 0.03, **p = 0.04, ***p = 0.0001)

Galindo-Filho,2013[8]	In-vivo	Adult with moderate to severe asthma	salbutamol and ipratropium	BiPAP : IPAP=12cmH_2O, EPAP =5cmH_2O	cJN		Control group	Experimental Group	P
						RR (breaths/min)			
						VT (L)	21.1 ±2.21	14.3 ± 2.54	< 0.001
						VE (L)	0.46 ± 0.08	0.55±0.07	=0.01
						FEV_1, % predicted	9.65 ± 1.63	7.77 ± 0.84	=0.006
						FVC, % predicted	29.8±8.9	46.7±0.5	=0.02
						PEF, % predicted	23.2±7.1	41.2±1.5	=0.02
						IC,% predicted	26.9±12.1	67.3±38.3	=0.01
							31.2±9.1	54.9±28.8	=0.01

IPAP, inspiratory positive airway pressure; EPAP, expiratory positive airway pressure; CPAP, continuous positive airway pressure; PEFR, peak expiratory flow rate ; PPEFR%,per cent of predicted peak expiratory flow rate; RR, respiratory rate; VT, tidal volume; VE, minute ventilation; cJN, continuous jet nebulizer; FEV1, Forced expiratory volume in on e second; FVC, forced vital capacity; PEF, predicted peak expiratory; IC: inspiratory capacity.

5. Pollack CV, Fleisch K B, Dowsey K. Treatment of Acute Bronchospasm With β-Adrenergic Agonist Aerosols Delivered by a Nasal Bilevel Positive Airway Pressure Circuit - ScienceDirect[J]. Annals of Emergency Medicine, 1995, 26(5):552-557.
6. Parkes S N, Bersten A D. Aerosol kinetics and bronchodilator efficacy during continuous positive airway pressure delivered by face mask. [J]. Thorax, 1997, 52(2):171-175.
7. BrandaoD C, Lima V M, Filho V G, et al. Reversal of bronchial obstruction with bi-level positive airway pressure and nebulization in patients with acute asthma.[J]. Journal of Asthma Research, 2009, 46(4):356-361.
8. Galindo-Filho V C, Brandao D C, Ferreira R D C S , et al. Noninvasive Ventilation Coupled With Nebulization During Asthma Crises: A Randomized Controlled Trial[J]. Respiratory

Care, 2013, 58(2):241-249.

Table 2.1.3. Studies comparing the inhaled dose via aerosol delivery via NIV vs traditional nebulization via mask/mouthpiece among patients with Cystic Fibrosis

Author , year	Study type	Population	Inhaled medication	Ventilator setting	Nebulizer	Measurement			
Fauroux,2000[9]	In-vitro<br>In-vivo	Children with cystic fibrosis（6-21yr）	In Vitro: KCl solution<br>In Vivo：^{99m}Tc-phytates	PSV: Inspiratory pressure ranged from 8 to 10 cm H_2O.RR=20,Ti=1s	Inspiratory synchronized nebulizers	In vitro<br>MMAD（μm）	Control group<br>3.21 ± 0.13	Experimental group<br>3.16±0.02	P<br>NS.
						In vivo			
						Total deposition(MBq)	3.4±2.1	4.4±2.7	=0.016
						Dynamic deposition (MB q/min)	0.32±0.23	0.43±0.07	=0.018
Laube,2005[10]	In-vivo	Adult with cystic fibrosis	^{99m}Tc-DTPA	With expiratory pressure of 10–20 cmH_2O;	Pari LC Plus nebulizer(cJN)		With PEP	Without PEP	P
						MMAD (μm)	3.26±0.37	4.07±0.23	=0.008
						GSD	2.61±0.10	2.78±0.06	NS.
						Deposition fraction,%	6.10±3.05	10.76±4.52	= 0.0078

PSV,pressure-support ventilation; RR, respiratory rate; Ti, inspiratory time; cJN, continuous jet nebulizer; MMAD, mass median aerodynamic diameter; GSD, geometric standard deviation; PEP, positive expiratory pressure; NS:not significant.

9. Fauroux B, Itti E, Pigeot J, et al. Optimization of aerosol deposition by pressure support in children with cystic fibrosis: an experimental and clinical study.[J]. Am J Respir Crit Care Med, 2000, 162(6):2265-2271.
10. Laube B L, Geller D E,Lin T C , et al. Positive expiratory pressure changes aerosol distribution in patients with cystic fibrosis.[J]. Respiratory Care, 2005, 50(11): 1438-1444.

Recommendations 2.2

Voting for recommendations 2.2

**Recommendations 2.2**	During noninvasive ventilation using single limb circuit, placing metered dose inhaler with spacer between exhalation valve and mask, with actuation at the beginning of inspiration is recommended. There is no recommendation on the placement orientation (towards or away from patient) of the spacer.
**Evidence**	In vitro[2-4], in vivo[1,4]
	If you have additional evidence, please provide references:
**Likert score of 1-9**	□1 □2 □3 □4 □5 □6 □7 □8 □9<br>1= absolutely disagree, 9 = absolutely agree
**Comments**	
**Suggestions for future research**	

Table 2.2. Studies about the use of MDI during NIV

Author, year	Study type	Population	Inhaled medication	Ventilator setting	Nebulizer	Measurement							
Nava,2001[1]	In-vivo	Adult	Placebo; Salbutamol	NIV: VAPS pressure support: 14.3±1.8cm H_2O,VT:10ml/kg,	MDI		Placebo NIMV	IPPB	MDI-Spacer	MDI-	P* P**		P#
						ΔFEV$_1$%	NR	7.9 ± 7.1	10.8±11.4	9.6±8.8	< 0.05	NS.	NS.
						ΔFEV$_1$ (mL)	NR	108±91	119±114	112±67	NS.	NS.	< 0.05
Branconnier, 2005[2]	In-vitro	Adult	albuterol	IPAP=15cm H_2O, EPAP =5cm H_2O, VT=0.4L	MDI	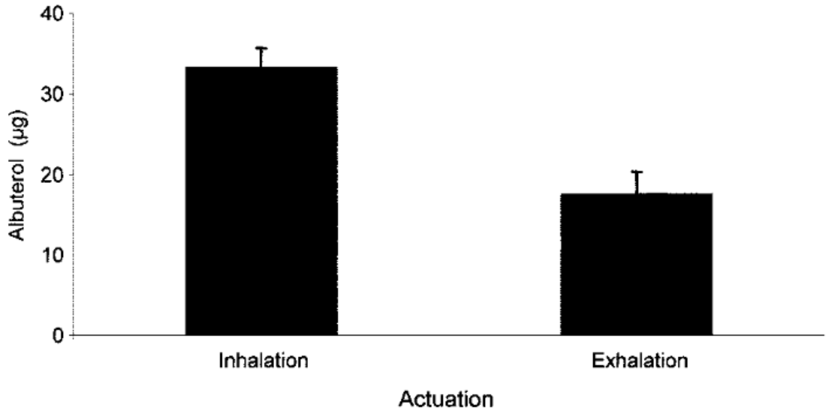 (P=0.001)							
Harb,2017[3]	In-vitro	Adult	salbutamol	BiPAP: IPAP=20cmH_2O, EPAP=5cmH_2O, I:E=1:3,RR=15b/min	MDI	FPD < 5 mm (mg)	T-piece+MDI	Large Spacer With pMDI	P				
							2049.5±383.7	3300.7±111.7	NS.				
AlQuaimi MM,2017[4]	In-vitro	Adult	albuterol	Spontaneous mode: IPAP=20cmH_2O, EPAP=5cmH_2O,Ti=1s, RR=15b/min	MDI		pMDI-N	pMDI-R	P				
						Inhaled mass (mg)	0.10 ± 0.01	0.09 ± 0.01	NS.				
						Inhaled mass percent,%	23.53 ± 2.03	21.38 ± 0.32	NS.				
Harb,2018[5]	In-vivo ex-vivo	Salbutamol		BiPAP: IPAP=20cmH_2O, EPAP=5cmH_2O, I:E=1:3,RR=15b/min	MDI	In-vivo	T-Piece+MDI	Large Spacer +pMDI	P				
						USAL0.5(%)	1.73±0.66	1.99±0.74	NS.				
						USAL24 (μg)	599.84±99.0	631.69±133.39	NS.				
						Ex-vivo SALF,%	54.3	49.9	NS.				

IPAP, inspiratory positive airway pressure; EPAP, expiratory positive airway pressure; VAPS, volume assured pressure support; I:E,:inspiration and expiration ratio;

VT, tidal volume; RR, respiratory rate; VMN, vibrating mesh nebulizer; MDI, metered dose inhaler; ΔFEV1,changes in forced expiratory volume in one second; FPD, fine parti cle dose; pMDI-N, pressurized metered dose inhaler with spacer in normal/recommended position; pMDI-R, pressurized metered dose inhaler with spacer in reversedposition;USAL0. 5,urinary salbutamol at 0.5 h after administration;USAL24,urinary salbutamol over 24 h after administration; SALF ,Salbutamol collected on filter; NS, not significant.

1. Nava S, Karakurt S, Rampulla C, et al.Salbutamol delivery during non-invasive mechanical ventilation in patients with chronic obstructive pulmonary disease: a randomized, controlled study[J]. Intensive Care Med, 2001, 27(10):1627-1635.

2. Branconnier M P , Hess D R . Albuterol delivery during noninvasive ventilation[J]. Respiratory Care, 2005, 50(12):1649-1653.

3. Harb H S, AA Elberry, H Rabea, et al. Is Combihaler usable for aerosol delivery in single limb non-invasive mechanical ventilation?[J]. Journal of Drug Delivery Science and Technology, 2017, 40:28-34.

4. AlQuaimi MM, Fink JB, Ari A. Efficiency of Different Aerosol Devices and Masks during Noninvasive Positive Pressure Ventilation in a Simulated Adult Lung Model. J Respir Med Lung Dis. 2017; 2(3): 1018. ISSN: 2475-5761. 2017.

5. Harb H S ,AA Elberry, H Rabea, et al. Performance of Large Spacer Versus Nebulizer T-Piece in Single-Limb Noninvasive Ventilation[J]. Respiratory care, 2018, 63:1360-1369.

Recommendations 2.3

Voting for recommendations 2.3

**Recommendations 2.3**	When placing the continuous nebulizer in-line with noninvasive ventilation, vibrating mesh nebulizer is more efficient in aerosol delivery than jet nebulizer. When available, vibrating mesh nebulizer is recommended over jet nebulizer.
**Evidence**	In vitro[1-3,5,7-11,13], in vivo[4,6-7,11-12]
	If you have additional evidence, please provide references:
**Likert score of 1-9**	□1 □2 □3 □4 □5 □6 □7 □8 □9<br>1= absolutely disagree, 9 = absolutely agree
**Comments**	
**Suggestions for future research**	

Table 2.3. Comparisons of inhaled dose between VMN and jet nebulizer via NIV

Author , year	Study type and	Population	Inhaled medication	Ventilator setting	Nebulizer	Measurement										
Abdelrahim, 2010[1]	In vitro	Adult	Terbutaline	Bi-level:<br>IPAP=20cmH$_2$0,<br>EPAP=5cmH$_2$O,<br>I:E=1:3,<br>RR=15，<br>VT=500mL	cJN;<br>VMN		Proximal to a breathing simulator			Distal to a breathing simulator						
							JN	VMN	P	JN	VMN	P				
						Inhalation filter (μg)	1270.2±161.3	2572.5±150.9	< 0.001	341.0±69.5	935.5±273.3	< 0.001				
						MMAD (μm)	3.2±0.3	4.1±0.4	< 0.01	3.2±0.2	3.8±0.2	< 0.001				
Michotte, 2014[2]	In vitro	Adult	Amikacin	Bi-level:<br>IPAP=20cmH$_2$0,<br>EPAP=5cmH$_2$0	cJN;VMN:<br>VAP-Pro;<br>VAS-Solo;<br>VAN-NIVO;		Before the exhalation port				After the exhalation port				P	Afte
							JN	VAP	VAS	VAN	JN	VAP	VAS	VAN		JN
						Inhaled dose (mg)	61.2±3.6	198±4.8	221.1±5.1	231.8±6.8	46.2±5.3	59.0±2.5	70.1±6.3	48.4	< 0.001	
Hassan, 2016[3]	In vitro	Adult	Salbutamol	AutoCPAP:<br>CPAP=7cmH$_2$0	cJN;<br>VMN		JN	VMN -PRO	VMN-Solo	VMN-NIVO	P					
						TED (μg)	2056.8±922.0	3186.3±491.4	3042.5±621.0	2056.8±922	< 0.001					
						FPD (μg)	386.2±202.0	465.8±80.8*	611.9±53.5	427.7±83.7	=0.02					
Saeed,2017[4]	In vitro	Adult	Salbutamol	Bi-level:<br>IPAP=20cmH$_2$0<br>EPAP=5cmH$_2$0，	cJN;<br>JN A(China)<br>JN B(Turkey)<br>JN C (UK)<br>VMN;<br>Pro,Solo,NIVO		JN			VMN						
							JN A	JN B	JN C	Solo	Pro	NIVO				
						Inhalation (μg)	395.7±86.17	506.88±156.59	685.21±381.84	1671.08±213.19	1597.12±180.9	1624.99±183.13				
Saeed,2017[5]	In-vitro	Adult	Salbutamol	Bi-level:<br>IPAP=20cmH$_2$O<br>EPAP=5cm H$_2$O	cJN;VMN		JN			VMN						
						Inhalation filter (μg)	1ml	2ml	4mL	1ml	2ml	4mL				
							685.2±381.8	1095.8±166.9	1338.8±42.1	1691.1±266.3	1922.9±207.4	1946.0±157.7				
Fink,2017[6]	In-vitro	Adult	Albuterol sulfate	Bi-level:<br>IPAP=20cmH$_2$O<br>EPAP=5cmH$_2$O	cJN;VMN		JN	VMN	P							
						Inhaled Mass (mg)	0.33 ± 0.02	0.72 ± 0.05	=0.042							
Haw,2020[7]	In-vitro	Adult	Radiolabel saline	Bi-level:<br>IPAP=15cmH$_2$0	cJN;<br>iJN;		At the ventilator outlet				Pre-leak position		P			
						Inhaled mass ,%	JN	BEJN	VMN	P	JN	VMN				

				EPAP=5cmH20	VMN	Low leak(15-20L/min)	8.1±0.2	11.1±0.9	22.8±2.3	=0.001	4.2±1.7	23.7±5.1	< 0.01
				Ti= 1.0s		High leak(55-60L/min)	2.2±1.1	2.3±0.4	7.3±1.8	=0.04	1.5±0.7	6.8%	< 0.01
Abdelrahim, 2011[8]	In vivo	Adult	Terbutaline	Bi-level:	cJN;		JN				VMN		P
				IPAP=20cmH20	VMN	UTER0.5 (μg)	10.4±4.1				9.4±3.7		< 0.001
				EPAP=5cmH20		UTER24 (μg)	205.3±58.0				192.3±52.5		NS.
Galindo-Filho,2015[9]	In vivo	Adult	99mTc-SC	Bi-level:	cJN;	Deposited(counts)	JN				VMN		P
				IPAP=12cmH20	VMN		386,025±130,363				972,013±214,459		=0.005
				EPAP=5cmH20									
Avdeev, 2017[10]	In vivo	Adult	Salbutamol	N.R.	cJN;		JN				VMN		P
					VMN	SPO2 (%)	89±4.39				92.9±1.97		0.0001
						RR (min-1)	20.8±1.64				19.3±1.39		0.0003
						HR (min-1)	84.8±7.19				82.7±6.62		NS.
						Dyspnea Borg score	208.0±92.4				234±95.6		NS.
Galindo-Filho,2019[11]	In vivo	Adult	99mTc-DTPA	Bi-level:	cJN;		JN				VMN		P
				IPAP=12cmH20	VMN	Lungs (%)	3.14±1.71				12.05±2.96		< 0.001
				EPAP=5cmH20		Inhaled dose	12.51±6.31				22.78±3.38		=0.008
Hassan, 2017[12]	In-vitro/ In-vivo/ ex-vivo	Adult	Salbutamol	Bi-level:	cJN;	**In vitro**	JN				VMN		P
				IPAP=20cmH20	VMN；	Inhaled dose (%)	23.5±4.0				32.5±6.0		< 0.001
				EPAP=5cmH20		FPD<5μm (μg)	395.3±102.1				45.7±70.9		< 0.02
				I:E=1:3,RR=15		**Ex-vivo/In-vivo**	JN				VMN		P
						USAL0.5 (μg)	37.3±22.6				53.9±42.8		NS.
						USAL24 (μg)	411.4±184.3				683.8±160.9		NS.
						Inhalation filter(μg)	1078.3±237.8				2010.6±413.9		< 0.001
Saeed, 2018[13]	In-vivo/ ex-vivo	Adult	Salbutamol	Bi-level:	cJN;VMN		JN				VMN		P
				IPAP=20cmH2O			Fill volume:1ml		2ml		1ml	2ml	
				EPAP=5cm H2O		USAL0.5 (μg)	28.3±7.6		47.9±10.6		107.9±28	134.6±44.7	< 0.001
						USAL24 (μg)	146.3±42.3		429.5±106.9		542.1±88.4	599.6±60.9	< 0.001

IPAP, inspiratory positive airway pressure; EPAP, expiratory positive airway pressure; CPAP, continuous positive airway pressure; JN, jet nebulizer; VMN, vibrating mesh nebuliz er; cJN, continuous jet nebulizer; iJN, inspiratory synchronized jet nebulizer; Ti, inspiratory time; RR, respiratory rate; VT, tidal volume; MMAD, mass median aerodynamic dia meter; USAL0.5,urinary salbutamol at 0.5h after administration;USAL24,urinary salbutamol over 24h after administration; TED,total emitted dose; FPD, fine particle dose; HR, h eart rate; NR, not reported; NS, not significant.

1. Abdelrahim M E , Plant P , Chrystyn H . In-vitro characterisation of the nebulised dose during non-invasive ventilation.[J]. Journal of Pharmacy & Pharmacology, 2010, 62(8):966-972.

2. Michotte J B , Jossen E , Roeseler J , et al. In vitro comparison of five nebulizers during noninvasive ventilation: analysis of inhaled and lost doses.[J]. J Aerosol Med Pulm Drug Deliv, 2014, 27(6):430-440.
3.Ahmed, Hassan, Hoda, et al. In-Vitro Characterization of the Aerosolized Dose During Non-Invasive Automatic Continuous Positive Airway Pressure Ventilation[J]. Pulmonary Therapy, 2016, 2:115-126.
4.Saeed H , Elberry A A , Eldin A S , et al. Effect of Nebulizer Designs on Aerosol Delivery During Non-Invasive Mechanical Ventilation: A Modeling Study of In Vitro Data[J]. Pulmonary Therapy, 2017(8):1-9.
5. Saeed H , Mohsen M , Fink J B , et al. Fill volume, humidification and heat effects on aerosol delivery and fugitive emissions during noninvasive ventilation[J]. Journal of Drug Delivery Science and Technology, 2017, 39:372-378.
6. AlQuaimi MM, Fink JB, Ari A. Efficiency of Different Aerosol Devices and Masks during Noninvasive Positive Pressure Ventilation in a Simulated Adult Lung Model. J Respir Med Lung Dis. 2017; 2(3): 1018. ISSN: 2475-5761. 2017.
7. Haw A , Mcpeck M , Cuccia A D , et al. Face Mask Leak Determines Aerosol Delivery in Noninvasive Ventilation[J]. Respiratory Care, 2020, 65(9).
8.Abdelrahim M E , Plant P K , Chrystyn H . The relative lung and systemic bioavailability of terbutaline following nebulisation in non-invasively ventilated patients[J]. International Journal of Pharmaceutics, 2011, 420(2):313-318.
9. Galindo-Filho V C , Ramos M E , Rattes C S , et al. Radioaerosol Pulmonary Deposition Using Mesh and Jet Nebulizers During Noninvasive Ventilation in Healthy Subjects[J]. Respiratory Care, 2015:1238-46.
10. Avdeev S , Nuralieva G , Soe A K , et al. Comparison of response to aerosol drug delivery with mesh and jet nebulizers during non-invasive ventilation (NIV) in acute exacerbation of COPD. European Respiratory Journal 2017, 50.
11. Galindo-Filho VC, Alcoforado L, Rattes C, Paiva DN, et al. A mesh nebulizer is more effective than jet nebulizer to nebulize bronchodilators during non-invasive ventilation of subjects with COPD: A randomized controlled trial with radiolabeled aerosols[J]. Respiratory Medicine, 2019, 153:60-67.
12. Hassan, Ahmed, Abdelrahman, et al In-vitro/in-vivo comparison of inhaled salbutamol dose delivered by jet nebulizer, vibrating mesh nebulizer and metered dose inhaler with spacer during non-invasive ventilation[J]. Experimental Lung Research, 2017, 43(1):19-28.
13. Saeed H , Ali A , Elberry A A , et al. Modeling and optimization of nebulizers' performance in non-invasive ventilation using different fill volumes: Comparative study between vibrating mesh and jet nebulizers[J]. Pulmonary Pharmacology & Therapeutics, 2018, 50:62-71.

Recommendations 2.4

Voting for recommendations 2.4

Recommendations 2.4	During noninvasive ventilation using single limb circuit, the continuous nebulizer is recommended to be placed between the exhalation valve and the mask.
Evidence	In vitro[1-9]
	If you have additional evidence, please provide references:
Likert score of 1-9	□1 □2 □3 □4 □5 □6 □7 □8 □9<br>1= absolutely disagree, 9 = absolutely agree
Comments	
Suggestions for future research	

Table 2.4. In vitro studies of inhaled dose at different nebulizer placements

Author , year	Study type	Inhaled medicat ion	Ventilator setting	Nebulizer	Measurement			
Chatmong-	In vitro	Albuterol	Spontaneous mode: cJN	RR=20		Proximal（At the ventilator outlet）	Distal（Between leak port and lung model）	P

kolchart, 2002[1]			IPAP/EPAP=10/5, 15/5,20/5, 15/10, 20/10, and25/10 cmH20; RR=10/20		Aerosol delivery,% 10/5	8.5±0.2				15.6±0.6				< 0.001
					15/5	8.4±0.2				22.2±1.0				< 0.001
					20/5	5.2±0.4				24.5±1.3				< 0.001
					15/10	10.3±0.4				13.8±0.2				< 0.001
					20/10	10.8±0.1				20.1±1.5				< 0.001
					25/10	7.3±0.3				22.7±0.6				< 0.001
Calvert, 2006[2]	In vitro	Salbutamol	Spontaneous mode: IPAP=20cmH2O, EPAP =5cm H2O	cJN	Mass of salbutamol \(μg)	Proximal	Distal							P
						Pre-ventilator	Before the expiration port	After the expiration port						
						267±26	544± 85	647 ±67						< 0.001
Abdelrahim, 2010[3]	In vitro	Terbutaline	Bi-level: IPAP=20cmH2O, EPAP=5cmH2O, I:E=1:3,RR=15, VT=500mL	cJN; VMN		Before the exhalation port				After th exhalation port				P
						JN	VMN			JN	VMN			
					Inhalation filter (μg)	1270.2±161.3	2572.5±150.9			341.0±69.5	391.3±132.0			< 0.001
					FPD(μg)	559±32.5	1314.4±194.9			935.5±273.3	1126.3±142.5			NS.
Michotte, 2014[4]	In vitro	Amikacin	Bi-level: IPAP=20cmH20, EPAP=5cmH20	cJN; VMN: Pro, Solo, NIVO		Before the exhalation port				After the exhalation port				
						JN	Pro	Solo	NIVO	JN	Pro	Solo	NIVO	
					Inhaled dose (mg)	61.2±3.6	198±4.8	221.1±5.1	231.8±6.8	46.2±5.3	59.0±2.5	70.1±6.3	46.2±5.3	
Dai, 2014[5]	In vitro	Salbutamol	Spontaneous mode: IPAP/EPAP=15/5, 25/5,15/10and 25/10cmH20.	cJN		Proximal (Pre-ventilator)				Distal（Between exhalation valve and lung model）				P
					Aerosol delivery%	Single-arch	Whisper swivel	Plateau valve		Single-arch	Whisper swivel	Plateau valve		
					15/5	20.83±0.54	22.01±1.21	23.97±2.19		23.27±0.57	19.59±0.99	20.17±0.76		< 0.05
					25/5	22.23±0.79	24.59±1.09	28.22±1.59		24.18±1.08	20.7±0.71	22.43±0.72		< 0.05
					15/10	21.11±1.03	22.18±1.04	23.55±1.34		19.83±0.87	18.37±0.37	19.46±1.03		< 0.05
					25/10	22.70±0.93	24.76±0.66	25.02±1.0		20.69±0.59	15.99±1.26	18.31±0.54		< 0.05
Michotte, 2015[6]	In vitro	Amikacin	Bi-level: IPAP=15cmH2O, EPAP=5cmH2O, RR=15/25,Res=10/20	VMN	IND (mg)	Proximal (Pre-ventilator)				Distal(Between exhalation valve and lung model)				P
					RR 15 Res 10	73±1				130±1				< 0.01
					RR 25 Res 10	83±1				151±1				< 0.01
					RR 15 Res 20	69±2				150±2				< 0.01

Peng, 2018[7]	In vitro	Salbutamol	Spontaneous mode : IPAP/EPAP=15/5, 25/5, 15/10 and 25/10 cmH_2O,RR=10/20 bpm/min	cJN			P1	P2	P3	P4	P5	P6		
						Single-arch exhalation port								
					Aerosol delivery% RR=10	15/5	16.73±1.70	9.06±0.34	9.43±0.49	21.61±1.26	24.79±0.42	19.40±1.29		
						25/5	19.87±0.71	5.76±0.83	14.26±1.84	23.47±2.86	25.44±3.20	7.04±0.66		
						15/10	8.39±0.86	5.16±0.48	15.90±0.82	19.32±1.86	15.46±2.06	10.12±2.23		
						25/10	10.02±0.55	7.35±0.51	4.14±0.23	12.69±1.99	15.99±0.61	13.92±1.95		
						Whisper swivel								
						15/5	18.80±1.50	10.17±0.34	10.52±1.73	18.97±1.50	21.71±2.44	20.17±2.81		
						25/5	21.10±1.12	17.36±0.96	13.40±0.56	18.91±1.19	24.84±1.36	19.02±1.07		
						15/10	12.20±0.24	6.90±0.11	2.59±0.85	7.80±1.53	9.89±0.82	4.93±1.57		
						25/10	11.62±0.88	8.15±1.86	1.68±0.44	4.03±2.02	4.60±1.57	6.68±0.10		
						Single-arch exhalation port								
					Aerosol delivery% RR=20	15/5	24.47±0.56	21.00±0.91	22.69±2.56	26.29±3.32	19.79±1.53	6.58±0.44		
						25/5	35.24±1.26	26.36±2.78	26.54±0.54	28.84±1.06	24.41±1.62	3.82±1.75		
						15/10	15.08±2.16	6.13±1.22	2.07±0.51	15.95±2.38	19.95±0.67	4.03±0.72		
						25/10	25.98±1.19	20.16±0.98	17.30±2.09	33.22±2.81	30.77±0.83	7.55±0.53		
						Whisper swivel								
						15/5	20.21±2.77	14.69±1.79	15.04±1.67	27.62±1.42	23.81±2.44	4.62±1.49		
						25/5	35.08±2.26	32.60±1.02	28.36±1.26	30.84±1.66	25.04±1.20	3.29±1.41		
						15/10	15.50±2.07	5.43±0.44	3.49±0.34	11.34±1.97	13.76±1.90	12.92±1.97		
						25/10	25.56±1.57	20.05±2.27	12.41±2.82	26.59±2.84	25.95±2.77	18.42±1.52		
Haw, 2020[8]	In vitro	Radiolabel saline	Bi-level: IPAP=15cmH_2O, EPAP=5cmH_2O, Ti= 1.0s	VMN; cJN		Proximal (At the ventilator)		Distal (Pre-exhalation valve)		P				
					Inhaled mass ,%	JN	VMN	JN	VMN					
					Low leak (15-20L/min)	8.1±0.2	22.8±2.3	4.2±1.7	23.7±5.1	NS.				
					High leak (55-60L/min)	2.2±1.1	7.3±1.8	1.5±0.7	6.8%	NS.				
Tan, 2021[9]	In vitro	Salbutamol	Spontaneous mode : IPAP/EPAP= 16/4, 16/8, 20/4 and 20/8 cm H_2O	VMN		Proximal (Near the mask)				Distal (15 cm away from the mask)				P
					Aerosol output %	OronasalI	OronasalII	Nasal I	Nasal II	OronasalI	OronasalII	Nasal I	Nasal II	
					16/4cmH_2O	7.8±0.9	6.0±0.5	5.3±0.6	3.7±0.5	9.6±0.2	8.3±0.3	8.3±0.70	5.3±0.2	< 0.05
					16/8cmH_2O	2.3±0.4	1.7±0.0	2.1±0.1	2.3±0.1	2.5±0.2	2.8±0.1	1.8±0.0	2.5±0.2	NS
					20/4 cmH_2O	15.8±1.6	13.1±1.2	10.6±1.1	9.6±1.7	21.1±1.1	15.9±0.7	15.2±1.0	13.5±1.4	< 0.001
					20/8 cmH_2O	2.0±0.1	2.6±0.2	2.2±0.2	1.8±0.1	2.4±0.2	3.2±0.4	2.6±0.4	2.3±0.3	NS

IPAP, inspiratory positive airway pressure; EPAP, expiratory positive airway pressure; RR, respiratory rate; VMN, vibrating mesh nebulizer; Res, lung model resistance. IND, inhaled dose ; FPD, fine particle dose; NS, not significant; cJN, continuous jet nebulizer; P1, near the simulated lung and 15 cm from the distal end of the exhalation valve; P2,near the distal end of the exhalation valve;P3,near the proximal end of the exhalation valve; P4,80 cm from the proximal end of the exhalation valves;P5,near the humidifiers;P6,at the ventilator outlet; OronasalI, oronasal mask,BestFit1; OronasalII, oronasal mask,BestFit2; NasalI, nasal mask,BestFit1; NasalII, nasal mask,BestFit2.

1. Chatmongkolchart S, Schettino G, Dillman C, et al. In vitro evaluation of aerosol bronchodilator delivery during noninvasive positive pressure ventilation: effect of ventilator settings and nebulizer position.[J]. Critical Care Medicine, 2002, 30(11):2515-9.
2. Calvert L D, Jackson J M, White J A , et al. Enhanced delivery of nebulised salbutamol during non-invasive ventilation[J]. Journal of Pharmacy and Pharmacology, 2006, 58(11):1553-1557.
3. Abdelrahim M E, Plant P, Chrystyn H. In-vitro characterisation of the nebulised dose during non-invasive ventilation.[J]. Journal of Pharmacy & Pharmacology, 2010, 62(8):966-972.
4. Michotte J B, Jossen E, Roeseler J , et al. In vitro comparison of five nebulizers during noninvasive ventilation: analysis of inhaled and lost doses.[J]. J Aerosol Med Pulm Drug Deliv, 2014, 27(6):430-440.
5. Dai B, Kang J, Sun L F, et al. Influence of exhalation valve and nebulizer position on albuterol delivery during noninvasive positive pressure ventilation.[J]. Journal of Aerosol Medicine & Pulmonary Drug Delivery, 2014, 27(2):125.
6.Michotte J B , Staderini E , Pennec D L , et al. In Vitro Comparison of a Vibrating Mesh Nebulizer Operating in Inspiratory Synchronized and Continuous Nebulization Modes During Noninvasive Ventilation[J]. J Aerosol Med Pulm Drug Deliv, 2015,9:1-9
7.Peng Y, Dai B ,Hu C X , et al. Which Nebulizer Position Should Be Avoided? An Extended Study of Aerosol Delivery and Ventilator Performance during Noninvasive Positive Pressure Ventilation[J]. Respiration, 2018, 95:145-153.
8. Haw A,Mcpeck M, Cuccia A D , et al. Face Mask Leak Determines Aerosol Delivery in Noninvasive Ventilation[J]. Respiratory Care, 2020, 65(9).
9. Tan W, Dai B, Lu C L,et al. The Effect of Different Interfaces on the Aerosol Delivery with Vibrating Mesh Nebulizer During Noninvasive Positive Pressure Ventilation[J]. Journal of Aerosol Medicine and Pulmonary Drug Delivery, 2021.

Recommendations 2.5

Voting for recommendations 2.5

**Recommendations 2.5**	During noninvasive ventilation using a single limb circuit, with the continuous nebulizer placed between mask and exhalation valve, there is no recommendation on the type of exhalation valve
**Evidence**	In vitro[1,2], in vivo[1]
	If you have additional evidence, please provide references:
**Likert score of 1-9**	□1 □2 □3 □4 □5 □6 □7 □8 □9<br>1= absolutely disagree, 9 = absolutely agree
**Comments**	
**Suggestions for future research**	

Table 2.5. In vitro studies associated with the effect of the type of exhalation valve and nebulization position on aerosol delivery

Author,	Study	Inhaled	Ventilator	Nebulizer	Measurement

year	type	medication	setting							
Dai, 2014[1]	In vitro	Salbutamol	Spontaneous mode: cJN IPAP/EPAP=15/5, 25/5,15/10and 25/10cmH$_2$0.	Aerosol delivery%		Single-arch exhalation port	Whisper swivel	Plateau valve	P*	P#	P**
				15/5	Proximal	20.83±0.54	22.01±1.21	23.97±2.19	NS.	<0.05	<0.05
					Distal	23.27±0.57	19.59±0.99	20.17±0.76	<0.05	NS.	<0.05
				25/5	Proximal	22.23±0.79	24.59±1.09	28.22±1.59	<0.05	<0.05	<0.05
					Distal	24.18±1.08	20.7±0.71	22.43±0.72	<0.05	<0.05	<0.05
				15/10	Proximal	21.11±1.03	22.18±1.04	23.55±1.34	NS.	<0.05	<0.05
					Distal	19.83±0.87	18.37±0.37	19.46±1.03	<0.05	<0.05	NS.
				25/10	Proximal	22.70±0.93	24.76±0.66	25.02±1.0	NS.	NS.	NS.
					Distal	20.69±0.59	15.99±1.26	18.31±0.54	<0.05	<0.05	<0.05
Peng, 2018[2]	In vitro	Salbutamol	Spontaneous mode : cJN IPAP/EPAP=15/5, 25/5, 15/10 and 25/10 cmH$_2$O,RR=10/20 bpm/min	Aerosol delivery%		Single-arch exhalation port	Whisper swivel		P		
				RR=10							
				15/5	P1	16.73±1.70	18.80±1.50		<0.05		
					P2	9.06±0.34	10.17±0.34		<0.05		
					P3	9.43±0.49	10.52±1.73		NS.		
					P4	21.61±1.26	18.97±1.50		NS.		
					P5	24.79±0.42	21.71±2.44		<0.05		
					P6	19.40±1.29	20.17±2.81		NS.		
				25/5	P1	19.87±0.71	21.10±1.12		<0.05		
					P2	5.76±0.83	17.36±0.96		<0.05		
					P3	14.26±1.84	13.40±0.56		NS.		
					P4	23.47±2.86	18.91±1.19		<0.05		
					P5	25.44±3.20	24.84±1.36		NS		
					P6	7.04±0.66	19.02±1.07		<0.05		
				15/10	P1	8.39±0.86	12.20±0.24		<0.05		
					P2	5.16±0.48	6.90±0.11		<0.05		
					P3	15.90±0.82	2.59±0.85		<0.05		
					P4	19.32±1.86	7.80±1.53		<0.05		
					P5	15.46±2.06	9.89±0.82		<0.05		
					P6	10.12±2.23	4.93±1.57		<0.05		
				25/10	P1	10.02±0.55	11.62±0.88		<0.05		
					P2	7.35±0.51	8.15±1.86		NS.		

	P3	4.14±0.23	1.68±0.44	<0.05
	P4	12.69±1.99	4.03±2.02	<0.05
	P5	15.99±0.61	4.60±1.57	<0.05
	P6	13.92±1.95	6.68±0.10	<0.05
		RR=20		
15/5	P1	24.47±0.56	20.21±2.77	<0.05
	P2	21.00±0.91	14.69±1.79	<0.05
	P3	22.69±2.56	15.04±1.67	<0.05
	P4	26.29±3.32	27.62±1.42	NS
	P5	19.79±1.53	23.81±2.44	<0.05
	P6	6.58±0.44	4.62±1.49	<0.05
25/5	P1	35.24±1.26	35.08±2.26	NS.
	P2	26.36±2.78	32.60±1.02	<0.05
	P3	26.54±0.54	28.36±1.26	<0.05
	P4	28.84±1.06	30.84±1.66	<0.05
	P5	24.41±1.62	25.04±1.20	NS.
	P6	3.82±1.75	3.29±1.41	NS.
15/10	P1	15.08±2.16	15.50±2.07	NS.
	P2	6.13±1.22	5.43±0.44	NS.
	P3	2.07±0.51	3.49±0.34	NS.
	P4	15.95±2.38	11.34±1.97	<0.05
	P5	19.95±0.67	13.76±1.90	<0.05
	P6	4.03±0.72	12.92±1.97	<0.05
25/10	P1	25.98±1.19	25.56±1.57	NS.
	P2	20.16±0.98	20.05±2.27	NS.
	P3	17.30±2.09	12.41±2.82	<0.05
	P4	33.22±2.81	26.59±2.84	<0.05
	P5	30.77±0.83	25.95±2.77	<0.05
	P6	7.55±0.53	18.42±1.52	<0.05

IPAP, inspiratory positive airway pressure;EPAP,expiratory positive airway pressure;RR,respiratory rate; cJN, continuous jet nebulizer; P1,near the simulated lung and 15 cm from the distal end of the exhalation valve; P2,near the distal end of the ex-halation valve;P3,near the proximal end of the exhalation valve;P4,80 cm from the proximal end of the exhalation valves;P5,near the humidifiers;P6,at the ventilator outlet;

P*:comparation between single-arch exhalation port and whisper swivel; P#: comparation between whisper swivel and plateau valve; P**: comparation between single-arch exhalation port and plateau valve;NS.:not significant.

1. Dai B, Kang J, Sun L F, et al. Influence of exhalation valve and nebulizer position on albuterol delivery during noninvasive positive pressure ventilation.[J]. Journal of Aerosol Medicine & Pulmonary Drug Delivery, 2014, 27(2):125.
2.Peng Y, Dai B ,Hu C X , et al. Which Nebulizer Position Should Be Avoided? An Extended Study of Aerosol Delivery and Ventilator Performance during Noninvasive Positive Pressure Ventilation[J]. Respiration, 2018,95:145-153.

Recommendations 2.6

Voting for recommendations 2.6

**Recommendations 2.6**	During aerosol delivery via noninvasive ventilation, turning off the humidifier for the sole purpose of increasing aerosol delivery is not recommended.	
**Evidence**	In vitro[1,2], in vivo[1]	
	If you have additional evidence, please provide references:	
**Likert score of 1-9**	□1 □2 □3 □4 □5 □6 □7 □8 □9<br>1= absolutely disagree, 9 = absolutely agree	
**Comments**		
**Suggestions for future research**		

Table 2.6. Studies compared aerosol delivery in NIV patients with and without humidification.

Author, year	Study type	Inhaled medication	Ventilator setting	Nebulizer	Measurement							
Saeed, 2017[1]	In-vivo/ ex-vivo	Salbutamol	Bi-level: IPAP=20cmH_2O, EPAP =5cm H_2O	cJN;VMN		VMN		JN		P		
						Without humidification	With humidification	Without humidification	With humidification			
					USAL0.5 (μg)	123.1±39.4	130.8±49.6	54.8±22.6	59.1±26.4	NS.		
					USAL24 (μg)	635.4±119.1	588.7±136.0	405.2±160.5	376.6±156.3	NS.		
Saeed, 2017[2]	In-vitro	Salbutamol	Bi-level: IPAP=20cmH_2O, EPAP =5cm H_2O	cJN;VMN		No heat and no humidification		Heat with humidification		Humidification with no heat		P
					Inhalation filter (μg)	VMN	JN	VMN	JN	VMN	JN	
						1955.0±156.0	1096.9±158.7	1998.4±168.7	1081.2±162.2	1852.9±158.6	1004.3±135.3	NS.

IPAP, inspiratory positive airway pressure;EPAP, expiratory positive airway pressure;JN, jet nebulizer; VMN, vibrating mesh nebulizer; USAL0.5,urinary salbutamol at 0.5 h after administration indices of relative lung bioavailability; USAL24,urinary salbutamol over 24 h after administration indices of relative systemic bioavailability; cJN, continuous jet nebulizer; NS,not significant.

1. Saeed H, Elberry A A , Eldin A S , et al. Effect of Nebulizer Designs on Aerosol Delivery During Non-Invasive Mechanical Ventilation: A Modeling Study of In Vitro Data[J]. Pulmonary Therapy, 2017(8):1-9.
2. Saeed H , Mohsen M , Fink J B , et al. Fill volume, humidification and heat effects on aerosol delivery and fugitive emissions during noninvasive ventilation[J]. Journal of Drug Delivery Science and Technology, 2017, 39:372-378.

Recommendations 2.7

Voting for recommendations 2.7

**Recommendations 2.7**	The aerosol delivery efficiency is less affected by the fill volume in the vibrating mesh nebulizer than the continuous jet nebulizer. For continuous jet nebulizers, more dilution is associated with greater aerosol delivery. Increasing fill volume for the sole purpose to improve aerosol delivery efficiency is not recommended.
**Evidence**	In vitro [1-4], in vivo[2,4]
	If you have additional evidence, please provide references:
**Likert score of 1-9**	□1 □2 □3 □4 □5 □6 □7 □8 □9<br>1= absolutely disagree, 9 = absolutely agree
**Comments**	
**Suggestions for future research**	

Table 2.7.Studies compared aerosol delivery in NIV patients with different fill volume.

<table>
<tr><th>Author, year</th><th>Study type</th><th>Inhaled medication</th><th>Ventilator setting</th><th>Nebulizer</th><th>Measurement</th></tr>
<tr><td>Hess, 1996[1]</td><td>In-vitro</td><td>Albuterol</td><td>NR</td><td>cJN (17 JNs with different brands with driving flow 6L/min, 8L/min and 10L/min).</td><td>
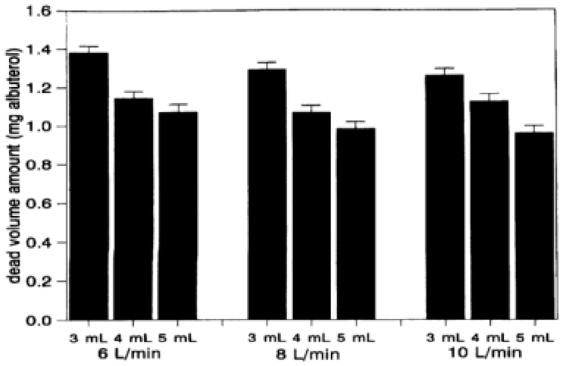

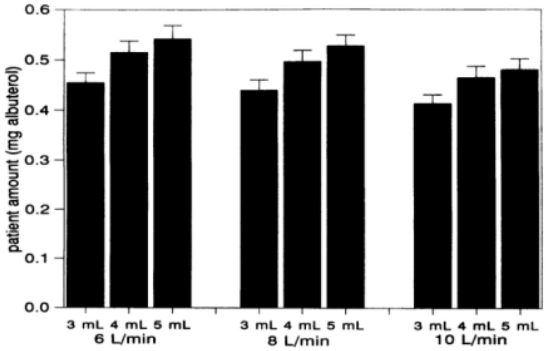

(P < 0.001)
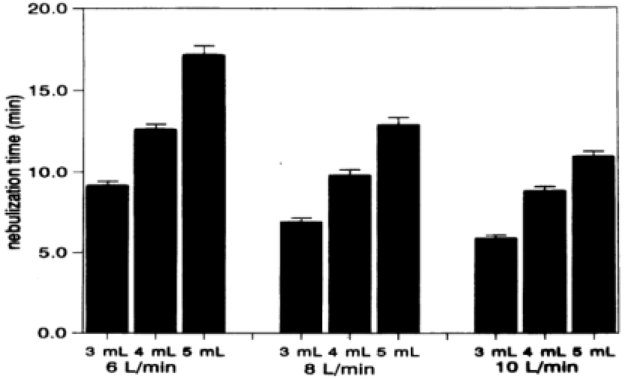

(P < 0.001) (P < 0.001)</td></tr>
<tr><td>Saeed, 2017[2]</td><td>In-vivo/ ex-vivo</td><td>Salbutamol</td><td>Bi-level: IPAP=20cmH2O, EPAP =5cm H2O</td><td>cJN(6L/min); VMN</td><td>
<table>
<tr><th></th><th>VMN</th><th></th><th></th><th>JN</th><th></th><th></th></tr>
<tr><td></td><td>Fill volume:1mL</td><td>2mL</td><td>P</td><td>Fill volume:1mL</td><td>2mL</td><td>P</td></tr>
<tr><td>USAL0.5 (μg)</td><td>107.9±28</td><td>134.6±44.7</td><td>NS.</td><td>28.3±7.6</td><td>47.9±10.6</td><td>< 0.001</td></tr>
<tr><td>USAL24 (μg)</td><td>542.1±88.4</td><td>599.6±60.9</td><td>NS.</td><td>146.3±42.3</td><td>429.5±106.9</td><td>< 0.001</td></tr>
<tr><td>Time (min)</td><td>2.29±0.08</td><td>4.22±0.7</td><td>< 0.05</td><td>3.42±0.08</td><td>6.86±0.42</td><td>< 0.001</td></tr>
</table></td></tr>
<tr><td>Saeed, 2017[3]</td><td>In-vitro</td><td>Salbutamol</td><td>Bi-level: IPAP=20cmH2O, EPAP =5cm H2O</td><td>cJN(6L/min);VMN</td><td>
<table>
<tr><th></th><th>Fill volume:1ml</th><th>2ml</th><th>4ml</th><th>P</th><th></th><th>Fill volume:1ml</th><th>2ml</th><th>4ml</th><th>P</th><th></th></tr>
<tr><td>Inhalation filter (μg)</td><td>VMN</td><td></td><td></td><td>1vs 2</td><td>2vs4</td><td>JN</td><td></td><td></td><td>1vs 2</td><td>2vs4</td></tr>
<tr><td></td><td>1691.1±266.3</td><td>1922.9±207.4</td><td>1946.0±157.7</td><td>< 0.05</td><td>NS</td><td>685.2±381.8</td><td>1095.8±166.9</td><td>1338.8±42.1</td><td>< 0.05</td><td>< 0.05</td></tr>
</table></td></tr>
</table>

Saeed, 2018[4]	In-vitro/ ex-vivo/ in-vivo	Salbutamol	Bi-level: IPAP=20cmH_2O, EPAP =5cm H_2O	cJN(6L/min);VMN		Fill volume:1ml	2ml	4ml	P		Fill volume:1ml	2ml	4ml	P	
					Inhalation filter(μg)	VMN			1vs2	2vs4	JN			1vs 2	2vs4
						1691.1±266.3	1922.9±207.4	1946.0±157.7	< 0.05	NS.	685.2±381.8	1095.8±166.9	1338.8±42.1	< 0.05	< 0.05
					USAL0.5 (μg)	107.9±28	134.6±44.7		NS.		28.3±7.6	47.9±10.6		< 0.05	
					USAL24 (μg)	542.1±88.4	599.6±60.9		NS.		146.3±42.3	429.5±106.9		< 0.05	

IPAP, inspiratory positive airway pressure;EPAP, expiratory positive airway pressure; cJN, continuous jet nebulizer;VMN, vibrating mesh nebulizer;USAL0.5,urinary salbutamol at 0.5 h after administration;USAL24,urinary salbutamol over 24 h after administration; NR,not reported;NS:,not significant.

1.Hess D, Fisher D, Williams P, Pooler S, Kacmarek RM. Medication nebulizer performance: Effects of diluent volume, nebulizer flow, and nebulizer brand. Chest 1996; 110:498-505.

2. Saeed H , Elberry A A , Eldin A S , et al. Effect of Nebulizer Designs on Aerosol Delivery During Non-Invasive Mechanical Ventilation: A Modeling Study of In Vitro Data[J]. Pulmonary Therapy, 2017(8):1-9.

3. Saeed H , Mohsen M , Fink J B , et al. Fill volume, humidification and heat effects on aerosol delivery and fugitive emissions during noninvasive ventilation[J]. Journal of Drug Delivery Science and Technology, 2017, 39:372-378.

4. Saeed H , Ali A , Elberry A A , et al. Modeling and optimization of nebulizers' performance in non-invasive ventilation using different fill volumes: Comparative study between vibrating mesh and jet nebulizers[J]. Pulmonary Pharmacology & Therapeutics, 2018, 50:62-71.

Recommendations 2.8

Voting for recommendations 2.8

**Recommendations 2.8**	The aerosol delivery efficiency is similar between CPAP and BiPAP, changing the noninvasive ventilation mode for the sole purpose of increasing aerosol delivery is not recommended.
**Evidence**	In vitro[2], in vivo[1]
	If you have additional evidence, please provide references:
**Likert score of 1-9**	□1 □2 □3 □4 □5 □6 □7 □8 □9<br>1= absolutely disagree, 9 = absolutely agree
**Comments**	
**Suggestions for future research**	

Table 2.8. Studies compared aerosol delivery in NIV patients with different ventilation mode.

Author, year	Study type	Inhaled medication	Ventilator setting	Nebulizer	Measurement									
Maccari, 2014[1]	In-vivo	^{99m}Tc	IPAP=15cmH_2O,	cJN		BiPAP	CPAP	P						
			EPAP =5cm H_2O;		Right lung deposition (counts)	111.5 ±15	108.7±40	NS.						
			CPAP=10cm H_2O		Left lung deposition (counts)	98.4 ±14	92.7±15	NS.						
					Trachea deposition (counts)	28.3±19	29.8±25	NS.						
Sutherasan, 2017[2]	In-vitro	Albuterol	BiPAP	cJN	Albuterol	CPAP			BiPAP					
			IPAP/EPAP=10/5,15/10,		delivery%	5	10	P	10/5	15/5	15/10	20/10	Pa	Pb
			15/5, and 20/10 cmH_2O;		Config. A	12.5 ± 0.4	11.5 ± 1.7	NS.	9.5 ± 1.0	13.5 ± 3.6	6.7 ± 0.4	8.7 ± 0.9	=0.033	=0.014
			CPAP:		Config. B	26.4 ± 3.6	23.9 ± 1.5	NS.	22.1 ± 3.5	27.7 ± 1.9	25.2 ± 1.1	25.7 ± 2.2	=0.033	=0.014
			P=5,10 cmH_2O		Config. C	37.0 ± 4.3	32.8 ± 2.6	NS.	30.9 ± 1.5	35.1 ± 4.0	34.1 ± 3.6	26.5 ± 6.1	=0.033	=0.014

IPAP, inspiratory positive airway pressure; EPAP, expiratory positive airway pressure; CPAP, continuous positive airway pressure; cJN, continuous jet nebulizer; VMN, vibrating mesh nebulizer; Config. A, a vented mask with the nebulizer directly connected to the mask; Config. B, an unvented mask with a leak port placed before the nebulizer; Config. C,an unvented mask with a leak port placed after the nebulizer; Pa, when compared 15/5 with 10/5; Pb, when compared 15/5 with 20/10; NS: not significant.

1. Maccari J G, Teixeira C, Savi A , et al. Nebulization During Spontaneous Breathing, CPAP, and Bi-Level Positive-Pressure Ventilation: A Randomized Analysis of Pulmonary Radioaerosol Deposition[J]. Respiratory Care, 2014, 59(4):479.

2.Sutherasan Y, Raimondo P, Ball L, et al. The effects of ventilator settings, nebulizer and exhalation port positions on albuterol delivery during noninvasive ventilation: an in vitro study[J]. Intensive Care Med Exp, 2017, 17(1):A169.

Recommendations 2.9

Voting for recommendations 2.9

**Recommendations 2.9**	When continuous nebulizer is placed between the mask and the exhalation valve during noninvasive ventilation with a single limb circuit, the aerosol delivery efficiency increases as IPAP increases or EPAP decreases. Changing the parameters for the sole purpose to improve aerosol delivery efficiency is not recommended.	
**Evidence**	In vitro[1,3-5], in vivo[2]	
	If you have additional evidence, please provide references:	
**Likert score of 1-9**	□1 □2 □3 □4 □5 □6 □7 □8 □9<br>1= absolutely disagree, 9 = absolutely agree	
**Comments**		
**Suggestions for future research**		

Table 2.9. Studies compared aerosol delivery in NIV patients with different ventilation settings.

Author ,	Study	Population	Inhaled	Ventilator setting	Nebulizer	Measurement

<table>
<tr><th>year</th><th>type</th><th></th><th>medication</th><th></th><th></th><th></th></tr>
<tr><td>Chatmong-kolchart, 2002[1]</td><td>In-vitro</td><td>Adult</td><td>Albuterol</td><td>BiPAP: IPAP/EPAP=10/5,15/5, 20/5, 15/10,20/10, and 25/10 cmH$_2$0 ; RR= 10/20</td><td>cJN</td><td>
<table>
<tr><th>Aerosol delivery%</th><th>10/5</th><th>15/5</th><th>20/5</th><th>15/10</th><th>20/10</th><th>25/10</th><th>P(EPAP5vs10)</th><th>P(IPAP10vs15)</th></tr>
<tr><td>Proximal</td><td>8.5±0.2</td><td>8.4±0.2</td><td>5.2±0.4</td><td>10.3±0.4</td><td>10.8±0.1</td><td>7.3±0.3</td><td>NS.</td><td>NS.</td></tr>
<tr><td>Distal</td><td>1 5.6±0.6</td><td>22.2±1.0</td><td>24.5±1.3</td><td>13.8±0.2</td><td>20.1±1.5</td><td>22.7±0.6</td><td>< 0.01</td><td>< 0.01</td></tr>
</table>
</td></tr>
<tr><td>Brandao, 2009[2]</td><td>In-vivo</td><td>Adult</td><td>fenoterol and ipratropium</td><td>BiPAP: IPAP/EPAP=15/5,15/10cmH$_2$0</td><td>cJN</td><td>
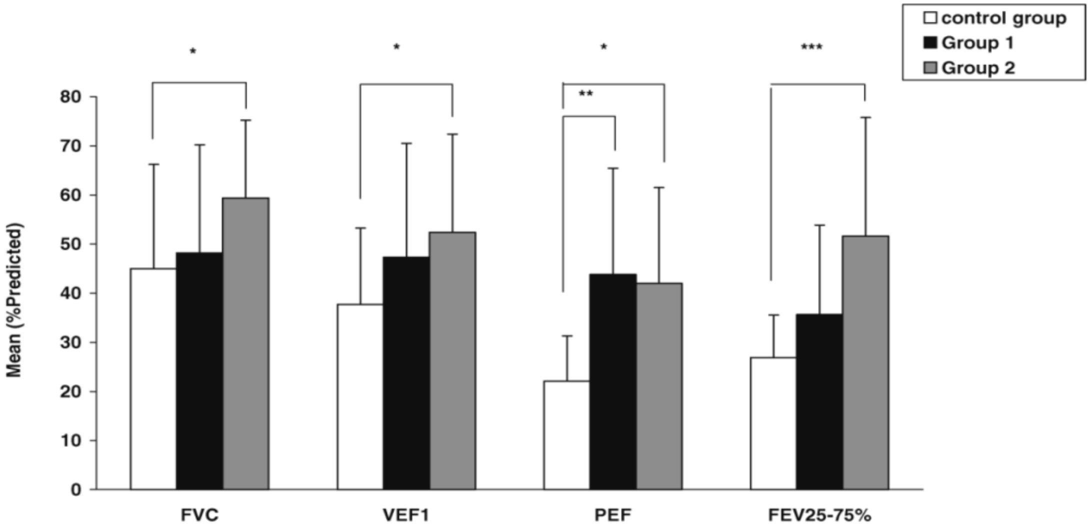

(Group1:15/5cmH$_2$0; Group2: 15/10 cmH$_2$0; * p = 0.03, ** p = 0.04, ***p = 0.0001)
</td></tr>
<tr><td>Dai, 2014[3]</td><td>In-vitro</td><td>Adult</td><td>salbutamol</td><td>BiPAP IPAP/EPAP=15/5,25/5, 15/10 and 25/10 cmH$_2$0.</td><td>cJN</td><td>
<table>
<tr><th>Single-arch</th><th>IPAP/EPAP=15/5</th><th>25/5</th><th>15/10</th><th>25/10</th><th>P (IPAP15vs25)</th><th>P (EPAP 5 vs 10)</th></tr>
<tr><td>Aerosol delivery%</td><td></td><td></td><td></td><td></td><td></td><td></td></tr>
<tr><td>Distal position</td><td>23.27±0.57</td><td>24.18±1.08</td><td>19.83±0.87</td><td>20.69±0.59</td><td>< 0.05</td><td>< 0.05</td></tr>
<tr><td>Proximal position</td><td>20.83±0.54</td><td>22.23±0.79</td><td>21.11±1.03</td><td>22.70±0.93</td><td>< 0.05</td><td>< 0.05</td></tr>
<tr><td>Whisper swivel</td><td></td><td></td><td></td><td></td><td></td><td></td></tr>
<tr><td>Distal position</td><td>19.59±0.99</td><td>20.7±0.71</td><td>18.37±0.37</td><td>15.99±1.26</td><td>< 0.05</td><td>< 0.05</td></tr>
<tr><td>Proximal position</td><td>22.01±1.21</td><td>24.59±1.09</td><td>22.18±1.04</td><td>24.76±0.66</td><td>< 0.05</td><td>< 0.05</td></tr>
<tr><td>Plateau exhalation valve</td><td></td><td></td><td></td><td></td><td></td><td></td></tr>
<tr><td>Distal position</td><td>20.17±0.76</td><td>22.43±0.72</td><td>19.46±1.03</td><td>18.31±0.54</td><td>< 0.05</td><td>< 0.05</td></tr>
<tr><td>Proximal position</td><td>23.97±2.19</td><td>28.22±1.59</td><td>23.55±1.34</td><td>25.02±1.08</td><td>< 0.05</td><td>< 0.05</td></tr>
</table>
</td></tr>
</table>

							CPAP(cmH$_2$O)			BiPAP(cmH$_2$O)					
Sutherasan, 2017[4]	In-vitro	Adult	Albuterol	BiPAP IPAP/EPAP=10/5,15/10, 15/5, and 20/10 cmH$_2$O; CPAP: P=5,10 cmH$_2$O	cJN	Albuterol delivery%	5	10	P	10/5	15/5	15/10	20/10	P_a	P_b
						Config. A*	12.5 ± 0.4	11.5 ± 1.7	NS.	9.5 ± 1.0	13.5 ± 3.6	6.7 ± 0.4	8.7 ± 0.9	=0.033	=0.014
						Config. B	26.4 ± 3.6	23.9 ± 1.5	NS.	22.1 ± 3.5	27.7 ± 1.9	25.2 ± 1.1	25.7 ± 2.2	=0.033	=0.014
						Config. C	37.0 ± 4.3	32.8 ± 2.6	NS.	30.9 ± 1.5	35.1 ± 4.0	34.1 ± 3.6	26.5 ± 6.1	=0.033	=0.014

								16/4cmH$_2$O	16/8cmH$_2$O	P_c	20/4cmH$_2$O	20/8 cmH$_2$O	P_d
Tan,2021[5]	In-vitro	Adult	Salbutamol	BiPAP IPAP/EPAP= 16/4, 16/8, 20/4 and 20/8 cm H$_2$O	VMN	Aerosol output%	Oronasal mask I	7.8±0.9	2.3±0.4	< 0.001	15.8±1.6	2.0±0.1	< 0.001
							Oronasal mask II	6.0±0.5	1.7±0.0	< 0.001	13.1±1.2	2.6±0.2	< 0.001
							Nasal mask I	5.3±0.6	2.1±0.1	< 0.001	10.6±1.1	2.2±0.2	< 0.001
							Nasal mask II	3.7±0.5	2.3±0.1	< 0.05	9.6±1.7	1.8±0.1	< 0.001

IPAP, inspiratory positive airway pressure; EPAP, expiratory positive airway pressure; CPAP, continuous positive airway pressure; cJN, continuous jet nebulizer; VMN, vibrating mesh nebulizer;RR,respiratory rate; Config. A, a vented mask with the nebulizer directly connected to the mask; Config. B, an unvented mask with a leak port placed before the nebulizer; Config. C,an unvented mask with a leak port placed after the nebulizer; P_a,when compared 15/5 with 10/5; P_b, when compared 15/5 with 20/10;P_c, when compared EPAP 4 with 8 ; P_d, when compared IPAP 16with 20;NS,not significant. OronasalI, oronasal mask,BestFit1; OronasalII, oronasal mask,BestFit2; NasalI, nasal mask,BestFit1; NasalII, nasal mask,BestFit2.

1. Chatmongkolchart S, Schettino G, Dillman C, et al. In vitro evaluation of aerosol bronchodilator delivery during noninvasive positive pressure ventilation: effect of ventilator settings and nebulizer position.[J]. Critical Care Medicine, 2002, 30(11):2515-9.
2. BrandaoD C, Lima V M, Filho V G, et al. Reversal of bronchial obstruction with bi-level positive airway pressure and nebulization in patients with acute asthma.[J]. Journal of Asthma Research, 2009, 46(4):356-361.
3. Dai B, Kang J, Sun L F, et al. Influence of exhalation valve and nebulizer position on albuterol delivery during noninvasive positive pressure ventilation.[J]. Journal of Aerosol Medicine & Pulmonary Drug Delivery, 2014, 27(2):125.
4.Sutherasan Y, Raimondo P, Ball L, et al. The effects of ventilator settings, nebulizer and exhalation port positions on albuterol delivery during noninvasive ventilation: an in vitro study[J]. Intensive Care Med Exp, 2017, 17(1):A169.
5. Tan W, Dai B, Lu C L, et al. The Effect of Different Interfaces on the Aerosol Delivery with Vibrating Mesh Nebulizer During Noninvasive Positive Pressure Ventilation[J]. Journal of Aerosol Medicine and Pulmonary Drug Delivery, 2021.

Recommendations 2.10

Voting for recommendations 2.10

**Recommendations 2.10**	When a continuous nebulizer is placed in-line with noninvasive ventilation, the aerosol delivery efficiency is higher with a non-vented mask than a vented mask. Aerosol administration with a vented mask is not recommended.
**Evidence**	In vitro[1-3]
	If you have additional evidence, please provide references:
**Likert score of 1-9**	□1 □2 □3 □4 □5 □6 □7 □8 □9<br>1= absolutely disagree, 9 = absolutely agree
**Comments**	
**Suggestions for future research**	

Table 2.10. Studies compared aerosol delivery in NIV patients using a non-vented mask vs vented mask

Author , year	Study type	Inhaled medication	Ventilator setting	Nebulizer	Measurement
Branconnier,2005[1]	In-vitro	Albuterol	Bi-PAP<br>IPAP=15cm H_2O,<br>EPAP =5cm H_2O,<br>VT=0.4L	MDI; cJN<br>(Spectrum mask : leak port in the circuit; Mirage mask :leak port in the mask).	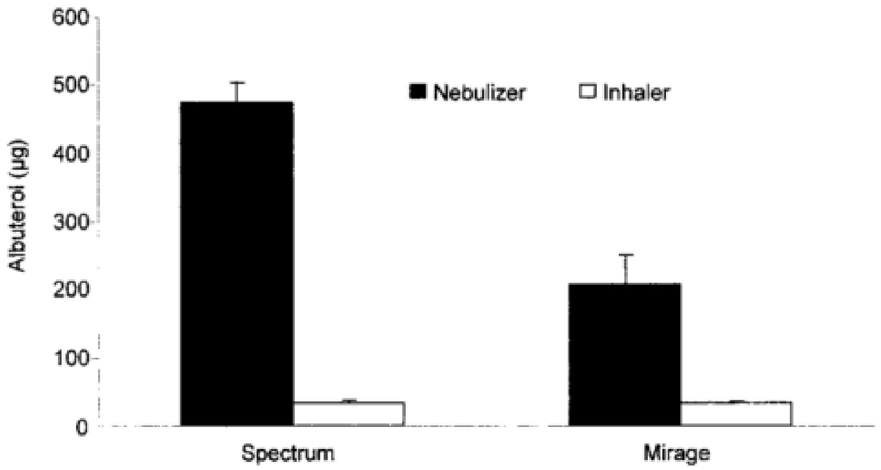<br>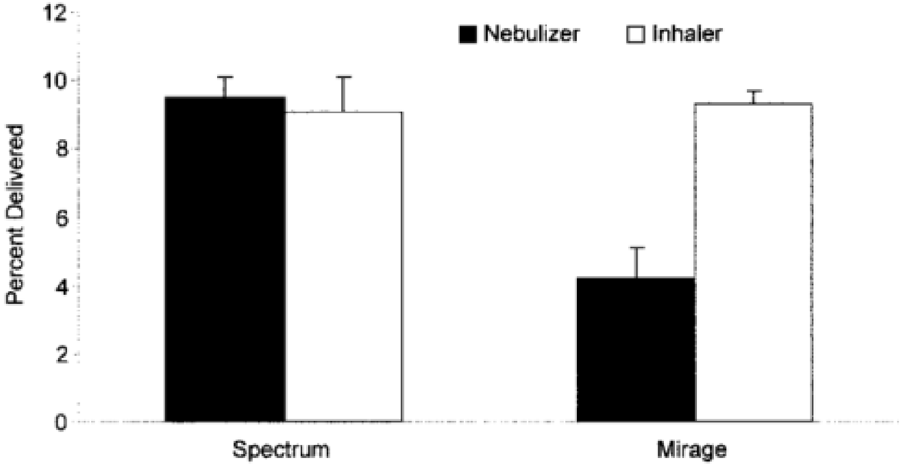<br>(P < 0.001) (P < 0.001)
Sutherasan,2017[2]	In-vitro	Albuterol	Bi-PAP	cJN	Config. A Config. B Config. C P

			IPAP/EPAP=10/5,15/10, 15/5, and 20/10 cm H_2O; CPAP=5, 10 cmH_2O		Albuterol delivery ,%								
					**CPAP:** 5cmH_2O	12.5 ± 0.4	26.4 ± 3.6	37.0 ± 4.3	< 0.001				
					10 cmH_2O	11.5 ± 1.7	23.9 ± 1.5	32.8 ± 2.6	< 0.001				
					**BiPAP:** 10 /5 cmH_2O	9.5 ± 1.0	22.1 ± 3.5	30.9 ± 1.5	< 0.001				
					15 /5 cmH_2O	13.5 ± 3.6	27.7 ± 1.9	35.1 ± 4.0	< 0.001				
					15/10 cmH_2O	6.7 ± 0.4	25.2 ± 1.1	34.1 ± 3.6	< 0.001				
					20 /10cmH_2O	8.7 ± 0.9	25.7 ± 2.2	26.5 ± 6.1	< 0.001				
AlQuaimi MM,2017[3]	In-vitro	Albuterol	Spontaneous mode: IPAP=20cmH_2O, EPAP=5cmH_2O,Ti=1s, RR=15b/min	VMN		NIVO			Aerogen Solo				
						Full-face mask	Oro-nasal mask	P	Full-face mask	Oro-nasal mask	Performa track mask	P*	
					IM(mg)	0.49 ± 0.02	0.58 ± 0.02	< 0.05	0.53 ± 0.04	0.72 ± 0.05	0.46 ± 0.06	< 0.05	
					IM%	19.59 ± 1.05	23.07 ± 0.70	< 0.05	21.02 ± 1.93	28.83 ± 1.93	18.51 ± 2.47	< 0.05	
Tan,2021[4]	In-vitro	Salbutamol	Bi-PAP IPAP/EPAP= 16/4, 16/8, 20/4 and 20/8 cm H_2O	VMN	Albuterol delivery %	OronasalI		Oronasal II		Nasal I		Nasa II	
						Proximal	Distal	Proximal	Distal	Proximal	Distal	Proximal	Distal
					16/4	7.8 ±0.9	9.6 ±0.2	6.0 ±0.5	8.3 ±0.3	5.3 ±0.6	8.3 ±0.70	3.7 ±0.5	5.3 ±0.2
					16/8	2.3±0.4	2.5±0.2	1.7±0.0	2.8±0.1	2.1±0.1	1.8±0.0	2.3±0.1	2.5±0.2
					20/4	15.8±1.6	21.1±1.1	13.1±1.2	15.9±0.7	10.6±1.1	15.2±1.0	9.6±1.7	13.5±1.4
					20/8	2.0±0.1	2.4±0.2	2.6±0.2	3.2±0.4	2.2±0.2	2.6±0.4	1.8±0.1	2.3±0.3

IPAP, inspiratory positive airway pressure;EPAP, expiratory positive airway pressure; CPAP, continuous positive airway pressure; Bi-PAP, bi-phasic positive airway pressure; cJN,continuous jet nebulizer; VMN, vibrating mesh nebulizer; VT,tidal volume;MDI, metered dose inhaler; Config. A, a vented mask with the nebulizer directly connected to the mask; Config. B, an unvented mask with a leak port placed before the nebulizer; Config. C,an unvented mask with a leak port placed after the nebulizer; IM, Inhaled mass; P*:comparison between full-face mask and oro-nasal mask.

1. Branconnier M P, Hess D R . Albuterol delivery during noninvasive ventilation[J]. Respiratory Care, 2005, 50(12):1649-1653.

2.Sutherasan Y, Raimondo P, Ball L , et al. The effects of ventilator settings, nebulizer and exhalation port positions on albuterol delivery during noninvasive ventilation: an in vitro study[J]. Intensive Care Med Exp, 2017, 17(1):A169.

3.AlQuaimi MM, Fink JB, Ari A. Efficiency of Different Aerosol Devices and Masks during Noninvasive Positive Pressure Ventilation in a Simulated Adult Lung Model. J Respir Med Lung Dis. 2017; 2(3): 1018. ISSN: 2475-5761. 2017.

4. Tan W, Dai B, Lu C L, et al. The Effect of Different Interfaces on the Aerosol Delivery with Vibrating Mesh Nebulizer During Noninvasive Positive Pressure Ventilation[J]. Journal of Aerosol Medicine and Pulmonary Drug Delivery, 2021.

Recommendations 2.11

Voting for recommendations 2.11

**Recommendations 2.11**	When non-vented mask is used during noninvasive ventilation, the aerosol delivery efficiency with optimal position is similar with the single limb and dual limb circuits. There is no recommendation for the use of single versus dual limb circuits for aerosol delivery.
**Evidence**	In vitro[1]<br>If you have additional evidence, please provide references:
**Likert score of 1-9**	□1 □2 □3 □4 □5 □6 □7 □8 □9<br>1= absolutely disagree, 9 = absolutely agree
**Comments**	
**Suggestions for future research**	

Table 2.11. Studies compared aerosol delivery in NIV patients when the single and dual limb circuits with non-vented mask are used.

Author, year	Study type	Inhaled medication	Ventilator setting	Nebulizer	Measurement					
Tan,2021[1]	In vitro	Salbutamol	**In single limb circuit:** BiPAP IPAP/EPAP=15/4cmH_2O; **In double limb circuit:** PSV mode, IPAP/EPAP=15/4cmH_2O; .	VMN	Delivery efficiency		Single limb	Double limb	Single limb	Double limb
							Non-humidified		Humidified	
						S1/D1	13.85±0.49	15.93±0.71	12.96±0.58	15.61±1.18
						S2	20.75±0.95		20.03±1.48	
						S3	4.12±0.22		4.34±0.33	
						D3		12.14±0.88		12.31±0.47
						S4	7.55±0.65		7.99±0.45	
						D4		19.45±1.32		18.04±0.93
						S5/D5	7.06±0.30	12.46±0.23	6.41±0.66	8.00±0.30

IPAP, inspiratory positive airway pressure; EPAP, expiratory positive airway pressure; PEEP,,positive end expiratory pressure; PSV,pressure-support ventilation; RR,respiratory rate; Ti, inspiratory time; JN, jet nebulizer; VMN,vibrating mesh nebulizer; N,non-humidified;H:humidified; VT, tidal volume; NS, not significant; S1/D1,before the mask;S2,15cm from the distal end of exhalation valve;S3,near the proximal end of exhalation valve;D3,at the Y-piece in the inspiratory limb;S4,15cm from proxi-mal end of exhalation valve;D4,15cm from Y-piece in inspiratory limb;S5/D5, at humidifier inlet.

1. Wei Tan,et al. Aerosol Delivery in an Adult Model of Noninvasive Ventilation with Single Limb Circuit or Dual Limb Circuit：Comparative study between Single Limb Circuit and Dual Limb Circuit[J]. Respiratory Care (under review)

Section 3

Aerosol Delivery via High-flow Nasal Cannula for Adult Patients

Recommendations 3.1

Voting for recommendations 3.1

**Recommendations 3.1**	The aerosol delivery efficiency with a nebulizer via high-flow nasal cannula is similar to a mask or mouthpiece. Discontinuing high-flow nasal cannula treatment to administer nebulizer with a mask or mouthpiece is not recommended.
**Evidence**	In vivo [1-4], in vitro[5]
	If you have additional evidence, please provide references:
**Likert score of 1-9**	□1 □2 □3 □4 □5 □6 □7 □8 □9<br>1= absolutely disagree, 9 = absolutely agree
**Comments**	
**Suggestions for future research**	

Table 3.1. Studies comparing the inhaled dose via aerosol delivery via HFNC vs traditional nebulization via a mask/mouthpiece

Author, year	Study type	Patient	Inhaled med	Comparison	Finding
Bräunlich, 2018[1]	RCT crossover	26 Stable COPD	Salbutamol 2.5 mg + ipratropium 0.5 mg	cJN via HFNC at 35 L/min vs cJN alone	FEV_1 change: 9.4 ± 13.6 vs 11.1 ± 17.2%, p = 0.5
Réminiac, 2018[2]	RCT crossover	25 Stable patients with reversible airflow obstruction	2.5 mg albuterol	VMN via HFNC at 30 L/min vs cJN with mask	FEV_1 improvement: 0.33 (0.14,0.39) vs 0.35 (0.18,0.55) L, p=0.11
Madney, 2019[3]	RCT crossover	12 stable COPD	5 mg salbutamol	VMN via HFNC at 5 L/min vs cJN via HFNC	Urinary albuterol excretion at 30 min and 24 h were higher with VMN than JN via HFNC (p<0.05)
Li, 2019[4]	Prospective dose response study	42 stable asthma and COPD patients	Albuterol at an escalating dose of 0.5,1.5,3.5 and 7.5 mg	VMN via HFNC at 15-20 L/min vs MDI+Spacer	FEV_1 increment at cumulative dose of 1.5 mg via HFNC was similar to 400 mcg albuterol via MDI+Spacer: 0.34±0.18 vs. 0.34±0.12 L, p = 0.878
Bennett G, 2019[5]	In-vitro study	Adult manikin	Albuterol 2.5mg	VMN via HFNC vs cJN via mask/mouthpiece vs VMN via mask/mouthpiece	Inhaled dose: VMN via HFNC: 6.8±0.5%; JN via mask: 9.1±0.3%; JN via mouthpiece: 12.9±2.5%; VMN via mask: 36.2±0.8%; VMN via mouthpiece: 28.5± 0.4%

HFNC, high-flow nasal cannula; RCT, randomized controlled trial; COPD, chronic obstructive pulmonary disease; cJN, continuous jet nebulizer; VMN, vibrating mesh nebulizer; FEV_1, forced expiratory volume at the first second.

1. Bräunlich J, Wirtz H. Oral versus nasal high-flow bronchodilator inhalation in chronic obstructive pulmonary disease. J Aerosol Med Pulm Drug Deliv. 2018;31(4):248– 54.

2. Réminiac F, Vecellio L, Bodet-Contentin L, Gissot V, Le Pennec D, Salmon Gandonniere C, et al. Nasal high-flow bronchodilator nebulization: a randomized cross-over study. Ann Intensive Care. 2018;8(1):128.

3. Madney YM, Fathy M, Elberry AA, Rabea H, Abdelrahim ME. Aerosol delivery through an adult high-flow nasal cannula circuit using low-flow oxygen. Respir Care 2019;64(4):453-461.

4. Li J, Zhao M, Hadeer M, Luo J, Fink JB. Dose response to transnasal pulmonary administration of bronchodilator aerosols via nasal high-flow therapy in adults with stable chronic obstructive pulmonary disease and asthma. Respiration. 2019;98(5):401–9

5. Bennett G, Joyce M, Fernandez EF, MacLoughlin R. Comparison of aerosol delivery across combinations of drug delivery interfaces with and without concurrent high-flow nasal therapy. Intensive Care Med Exp. 2019;7(1):20.

Recommendations 3.2

Voting for recommendations 3.2

**Recommendations 3.2**	Placing a nebulizer with a mask or mouthpiece on a patient who is using concurrent high-flow nasal cannula treatment is not recommended.	
**Evidence**	In vitro[1]	
	If you have additional evidence, please provide references:	
**Likert score of 1-9**	□1 □2 □3 □4 □5 □6 □7 □8 □9<br>1= absolutely disagree, 9 = absolutely agree	
**Comments**		
**Suggestions for future research**		

Table 3.2. Inhaled dose via aerosol delivery via HFNC vs nebulization via mask/mouthpiece with concurrent HFNC

Author, year	Study type	Nebulizer placement	HFNC flow	Inhaled	Inhaled Dose % with concurrent HFNC

				Dose % of VMN via HFNC	cJN via mask	cJN via mouthpiece	VMN via mask	VMN via mouthpiece
Bennett G, 2019[1]	In vitro	At humidifier	50 L/min	6.8 ± 0.5	5.7 ± 0.7	0.7 ± 0.5	4.2 ± 0.9	3.1 ± 0.5

HFNC, high-flow nasal cannula; cJN, continuous jet nebulizer; VMN, vibrating mesh nebulizer

1. Bennett G, Joyce M, Fernandez EF, MacLoughlin R. Comparison of aerosol delivery across combinations of drug delivery interfaces with and without concurrent high-flow nasal therapy. Intensive Care Med Exp. 2019;7(1):20.

Recommendations 3.3

Voting for recommendations 3.3

**Recommendations 3.3**	During aerosol delivery via high-flow nasal cannula, vibrating mesh nebulizer is more efficient in aerosol delivery than jet nebulizer, with no influence on flows or fraction of inspired oxygen. Vibrating mesh nebulizer is recommended for trans-nasal aerosol delivery.
**Evidence**	In vitro[1], in vivo[2,3]
	If you have additional evidence, please provide references:
**Likert score of 1-9**	□1 □2 □3 □4 □5 □6 □7 □8 □9<br>1= absolutely disagree, 9 = absolutely agree
**Comments**	
**Suggestions for future research**	

Table 3.3. Comparisons of inhaled dose between VMN and JN via HFNC

Author, year	Study type	HFNC device & circuit	Nebulizer placement	Breathing pattern	HFNC flow	Inhaled Dose % VMN	Inhaled Dose % cJN	*P*
Li, 2021[1]	In vitro	Airvo2 with AirSpiral circuit	At humidifier	Quiet	20 L/min	12.0 ± .3	6.7 ± .6	<.001
					40 L/min	5.2 ± .5	3.4 ± .4	.009
					60 L/min	2.7 ± .3	2.9 ± .4	.580
				Distressed	20 L/min	19.8 ± 1.0	14.1 ± .3	.001
					40 L/min	9.1 ± .3	6.4 ± .7	.004
					60 L/min	8.2 ± .3	3.9 ± .3	<.001
			Proximal to nasal cannula	Quiet	20 L/min	6.8 ± .2	6.4 ± .1	.017
					40 L/min	3.8 ± .2	2.9 ± .2	.002
					60 L/min	2.0 ± .1	2.3 ± .3	.241
				Distressed	20 L/min	10.6 ± .1	7.3 ± .5	<.001
					40 L/min	6.8 ± .4	5.3 ± .3	.006
					60 L/min	3.2 ± .1	4.1 ± .2	.002
		Optiflow	At humidifier	Quiet	20 L/min	16.5 ± .8	6.7 ± .5	<.001
					40 L/min	8.8 ± 1.0	4.2 ± .2	.011
					60 L/min	6.3 ± .2	3.3 ± .2	<.001
				Distressed	20 L/min	17.8 ± 1.0	8.2 ± .7	<.001
					40 L/min	16.5 ± .3	8.9 ± .7	<.001
					60 L/min	9.8 ± .7	7.3 ± 1.0	.025
			Proximal to nasal cannula	Quiet	20 L/min	6.7 ± .4	6.6 ± .5	.732
					40 L/min	3.9 ± .3	3.8 ± .4	.577
					60 L/min	2.0 ± .1	3.3 ± .1	<.001
				Distressed	20 L/min	13.6 ± .2	6.0 ± .4	<.001
					40 L/min	8.2 ± .6	5.8 ± .1	.018
					60 L/min	3.8 ± .1	3.8 ± .2	.826
Madney, 2018[2]	In vivo	NR	Between nasal cannula and humidifier	COPD patients	5 L/min	12.2 ± 4.4	7.9 ± 3.1	<0.05
Dugernier, 2017[3]	In vivo	Optiflow	At humidifier	Healthy volunteers	30 L/min	3.6 (2.1-4.4)	1.0 (0.7–2.0)	<0.05

HFNC, high-flow nasal cannula; VMN, vibrating mesh nebulizer; cJN, continuous jet nebulizer;NR, not reported

1. Li J, Lucas Williams, Fink JB. The Impacts of High-flow Nasal Cannula Device, Nebulizer Type and Its Placement on Trans-nasal Aerosol Drug Delivery. Respiratory care (in press)
2. Madney YM, Fathy M, Elberry AA, Rabea H, Abdelrahim ME. Aerosol delivery through an adult high-flow nasal cannula circuit using low-flow oxygen. Respir Care. 2019;64(4):453–61.
3. Dugernier J, Hesse M, Jumetz T, Bialais E, Roeseler J, Depoortere V, et al. Aerosol delivery with two nebulizers through high-flow nasal cannula: a randomized cross-over single-photon emission computed tomography- computed tomography study. J Aerosol Med Pulm Drug Deliv. 2017;30(5): 349–58.

Recommendations 3.4

Voting for recommendations 3.4

**Recommendations 3.4**	Nebulizers are recommended to be placed at the inlet of humidifier at flows ≥ 10 L/min. At flows < 10 L/min, vibrating mesh nebulizer is recommended to be placed close to nasal cannula.
**Evidence**	In vitro[1,2]
	If you have additional evidence, please provide references:
**Likert score of 1-9**	□1 □2 □3 □4 □5 □6 □7 □8 □9<br>1= absolutely disagree, 9 = absolutely agree
**Comments**	
**Suggestions for future research**	

Table 3.4. In vitro studies of inhaled dose at different nebulizer placements

Author, year	Study type	Nebulizer	HFNC devices	Breathing pattern	HFNC gas flow (L/min)	Nebulizer position Inhaled Dose %	
Li,2021[1]	In vitro	VMN	Airvo2 with AirSpiral circuit	Quiet (500mL /15bpm/ 1:2 / 22.5 L/min)		At the humidifier	Proximal to nasal cannula
					10	15.9 ± .2	10.1 ± .3
					20	12.0 ± .3	6.8 ± .2
					40	5.2 ± .5	3.8 ± .2
					60	2.7 ± .3	2.0 ± .1
				Distressed (700 mL /30bpm / 1:1/ 42 L/min)	10	18.6 ± .8	18.5 ± .5
					20	19.8 ± 1.0	10.6 ± .1
					40	9.1 ± .3	6.8 ± .4
					60	8.2 ± .3	3.2 ± .1
			Airvo 2 with 900PT501 circuit	Quiet (500mL /15bpm/ 1:2 / 22.5 L/min)		At the humidifier	Proximal to nasal cannula
					10	12.8 ± .6	10.2 ± .3
					20	10.9 ± .5	5.7 ± .3
					40	5.8 ± .3	3.3 ± .06
					60	3.1 ± .2	2.1 ± .2
				Distressed (700 mL /30bpm / 1:1/ 42 L/min)	10	16.2 ± .5	13.6 ± .4
					20	14.5 ± .9	8.0 ± .3
					40	5.7 ± .3	5.9 ± .5
					60	4.5 ± .1	3.7 ± .1
		cJN	Optiflow	Quiet (500mL /15bpm/ 1:2 / 22.5 L/min)		At the humidifier	Proximal to nasal cannula
					20	6.7 ± .5	6.6 ± .5
					40	4.2 ± .2	3.8 ± .4
					60	3.3 ± .2	3.3 ± .1
				Distressed (700 mL /30bpm / 1:1/ 42 L/min)	20	8.2 ± .7	6.0 ± .4
					40	8.9 ± .7	5.8 ± .1
					60	7.3 ± 1.0	3.8 ± .2
			Airvo2 with AirSpiral circuit	Quiet (500mL /15bpm/ 1:2 / 22.5 L/min)		At the humidifier	Proximal to nasal cannula
					20	6.7 ± .6	6.4 ± .1
					40	3.4 ± .4	2.9 ± .2
					60	2.9 ± .4	2.3 ± .3

				Distressed (700 mL /30bpm / 1:1/ 42 L/min)	20	14.1 ± .3	7.3 ± .5
					40	6.4 ± .7	5.3 ± .3
					60	3.9 ± .3	4.1 ± .2
Li,2020[2]	In vitro	VMN	Optiflow	Quiet (500mL /15bpm/ 1:2 / 22.5 L/min)		Inlet of humidifier	Close to patient
					5	19.5 ± .3	24.1 ± .8
					10	19.8 ± .8	15.8± .6
					20	16.5 ± .8	10.0 ± .4
					40	8.8± 1.0	3.9± .3
					60	6.3 ± .2	2.0 ± .1
				Distressed (700 mL /30bpm / 1:1/ 42 L/min)	5	12.9± 1.6	19.0 ± 1.0
					10	17.5± 1.0	17.8 ± .5
					20	17.8± .2	13.6 ± .2
					40	16.5 ± .3	8.3 ± .6
					60	9.8 ± .7	3.8 ± .1

VMN, vibrating mesh nebulizer; cJN, continuous jet nebulizer.

1. Li J, Lucas Williams, Fink JB. The Impacts of High-flow Nasal Cannula Device, Nebulizer Type and Its Placement on Trans-nasal Aerosol Drug Delivery. Respiratory care (in press)
2. Li J, Wu W, Fink JB. In vitro comparison between inspiration synchronized and continuous vibrating mesh nebulizer during trans-nasal aerosol delivery. Intensive Care Med Exp. 2020;8(1):6

Recommendations 3.5

Voting for recommendations 3.5

**Recommendations 3.5**	When metered dose inhaler is placed in-line with high-flow nasal cannula, it is recommended to be used with a spacer and placed close to nasal cannula with

	plume emitted into the flow.
**Evidence**	In vitro[1]
	If you have additional evidence, please provide references:
**Likert score of 1-9**	□1 □2 □3 □4 □5 □6 □7 □8 □9<br>1= absolutely disagree, 9 = absolutely agree
**Comments**	
**Suggestions for future research**	

Table 3.5. In vitro studies comparing MDI with or without spacers, as well as different placement

Author, year	Study type	Nebulizer position	Flow (LPM)	Inhaled dose %		
				Without spacer	Spacer placed with the flow	Spacer and placed against the flow
Szychowiak, 2021[1]	In vitro	Close to humidifier	30	19.7±0.6	67.3±11.2	38.0±6.8
			45	11.5±1.5	35.0±6.4	37.5±9.4
			60	3.9±1.0	27.0±11.5	20.7±4.6
		Close to nasal cannula	30	34.3±2.1	93.8±6.8	59.2±20.6
			45	32.9±16.1	56.7±11.1	59.7±16.6
			60	31.2±4.1	47.8±7.5	39.5±6.9

1. Szychowiak P, Gensburger S, Bocar T, et al. Pressurized Metered Dose Inhaler Aerosol Delivery Within Nasal High-Flow Circuits: A Bench Study[J]. Journal of Aerosol Medicine and Pulmonary Drug Delivery, 2021.

Recommendations 3.6

Voting for recommendations 3.6

**Recommendations 3.6**	To optimize aerosol delivery via high-flow nasal cannula, gas flow is recommended to be titrated below the patient's peak inspiratory flow if tolerated.
**Evidence**	In vitro[1-6], in vivo[7,8]
	If you have additional evidence, please provide references:
**Likert score of 1-9**	□1 □2 □3 □4 □5 □6 □7 □8 □9<br>1= absolutely disagree, 9 = absolutely agree
**Comments**	
**Suggestions for future research**	

Table 3.6. Studies comparing different gas flow settings for trans-nasal aerosol delivery with HFNC

Study type	Author, year	Neb position	Collection filter placement	Breathing pattern	Inspiratory flow (IF)	Gas Flow (GF)	GF: IF	Inhaled dose (%)
In vitro	Réminiac, 2016[1]	Inlet of humidifier	Trachea	Quiet breathing: Vt 500mL, RR 15bpm, I:E=1:1, Ti 2s	15	30.0	2.0	6.70
						45.0	3.0	3.50
						60.0	4.0	3.0
				Distressed breathing: Vt750mL, RR 30bpm, I:E=1:1, Ti 1s	45	30.0	0.67	10.30
						45.0	1.0	6.70
						60.0	1.33	5.10
	Dailey, 2017[2]	Inlet of humidifier	Nasal prongs	Quiet breathing: Vt 500mL, RR 16bpm, I:E=1:2, Ti 1.25s	24	10.0	0.42	26.70 ± 1.30
						30.0	1.25	11.60 ± 1.20
						50.0	2.08	3.50 ± 0.20
				Distressed breathing: Vt 750mL, RR 30bpm, I:E=1:1, Ti 1s	45	10.0	0.22	13.0 ± 3.0
						30.0	0.67	33.0 ± 5.0
						50.0	1.11	25.0 ± 2.0
	McGrath, 2019[3]	Outlet of humidifier	Trachea	Quiet breathing: Vt 500mL, RR 15bpm, I:E=1:1, Ti 2s	15	10.0	0.67	5.35 ± 2.81
						40.0	2.67	2.56 ± 1.38
						60.0	4.0	1.0 ± 0.26
	Li, 2019[4]	Inlet of humidifier	Trachea	Quiet breathing: Vt 300mL, RR 15bpm, I:E=1:2, Ti 1.33s	13.5	5	0.37	19.6 ± .6
						10	0.74	14.9 ± .3
						20	1.48	9.0 ± .2
						40	2.96	4.5 ± .4
						60	4.44	3.3 ± .6
				Quiet breathing: Vt 500mL, RR 15bpm, I:E=1:2, Ti 1.33s	22.5	5	0.22	25.8 ± .7
						10	0.44	23.6 ± 1.0

					20	0.88	16.7± .7
					40	1.78	9.1 ± .3
					60	2.67	5.8 ± .1
			Quiet breathing: Vt 700mL, RR 15bpm, I:E=1:2, Ti 1.33s	31.5	5	0.16	30.4 ± .4
					10	0.32	26.2 ± .4
					20	0.64	21.6 ± .5
					40	1.27	13.7 ± .5
					60	1.90	7.6 ± .5
			Distressed breathing: Vt 450mL, RR 30bpm, I:E=1:1, Ti 1.0s	27	5	0.19	18.6 ± .5
					10	0.37	20.8 ± .7
					20	0.74	19.4 ± .6
					40	1.48	14.9 ± .9
					60	2.22	7.7 ± 1.7
			Distressed breathing: Vt 700mL, RR 30bpm, I:E=1:1, Ti 1.0s	42	5	0.12	20.4 ± .3
					10	0.24	21.5 ± .5
					20	0.48	22.0 ± .6
					40	0.95	18.1 ± .3
					60	1.43	10.6 ± .6
			Distressed breathing: Vt 700mL, RR 30bpm, I:E=1:1.5, Ti 0.8s	52.5	5	0.10	22.7 ± 1.2
					10	0.19	25.3 ± .7
					20	0.38	26.7 ± .7
					40	0.76	19.3 ± .8
					60	1.14	13.3 ± 1.8
Li, 2020[5]	Inlet of humidifier	Trachea	Quiet breathing: Vt 500mL, RR 15bpm, I:E=1:2, Ti 1.33s	22.5	5	0.22	19.5 ± .3
					10	0.44	19.8 ± .8

						20	0.88	16.5 ± .8
						40	1.78	8.8± 1.0
						60	2.67	6.3 ± .2
				Distressed breathing: Vt 700mL, RR 30bpm, I:E=1:1.1, Ti 1.0s	42	5	0.12	12.9± 1.6
						10	0.24	17.5± 1.0
						20	0.48	17.8± .2
						40	0.95	16.5 ± .3
						60	1.43	9.8 ± .7
	Li, 2021[6]	At the humidifier	Trachea	Quiet breathing （Airvo 2 with AirSpiral circuit）		10	0.44	15.9 ± .2
						20	0.88	12.0 ± .3
						40	1.78	5.2 ± .5
						60	2.67	2.7 ± .3
				Distressed breathing （Airvo 2 with AirSpiral circuit）		10	0.24	18.6 ± .8
						20	0.48	19.8 ± 1.0
						40	0.95	9.1 ± .3
						60	1.43	8.2 ± .3
		Proximal to nasal cannula	Trachea	Quiet breathing （Airvo 2 with AirSpiral circuit）		10	0.44	10.1 ± .3
						20	0.88	6.8 ± .2
						40	1.78	3.8 ± .2
						60	2.67	2.0 ± .1
				Distressed breathing （Airvo 2 with AirSpiral circuit）		10	0.24	18.5 ± .5
						20	0.48	10.6 ± .1
						40	0.95	6.8 ± .4
						60	1.43	3.2 ± .1
In vivo	Alcoforado,	Inlet of	Lung dose	Normal healthy volunteer, Quiet	NR	10.0	NR	17.23 ± 6.78

2019[7]	humidifier	(radiolabeled aerosol)	breathing (n=23)		30.0	NR	5.71 ± 2.04
					50.0	NR	3.46 ± 1.24
Li, 2021[8]	Inlet of humidifier	FEV_1 via HFNC ≥ FEV_1 post MDI+Spacer	Stable COPD or asthma, Quiet breathing (n=75)		GF:IF=0.5	0.5	64%
					GF:IF=1.0	1.0	29%
					50	NR	27%

HFNC, high-flow nasal cannula; Vt, tidal volume; Ti, inspiratory time; RR, respiratory rates; I:E, ratio of inspiratory to expiratory time; NR, not reported; FEV1, forced expiratory volume at the 1st second.

1. Réminiac F, Vecellio L, Heuze-Vourc'h N, Petitcollin A, Respaud R, Cabrera M, et al. Aerosol therapy in adults receiving high flow nasal cannula oxygen therapy. J Aerosol Med Pulm Drug Deliv. 2016;29(2): 134–41.
2. Dailey PA, Harwood R, Walsh K, Fink JB, Thayer T, Gagnon G, et al. Aerosol delivery through adult high flow nasal cannula with heliox and oxygen. Respir Care. 2017;62(9):1186–92.
3. McGrath JA, O'Toole C, Bennett G, Joyce M, Byrne MA, MacLoughlin R. Investigation of fugitive aerosols released into the environment during high-flow therapy. Pharmaceutics. 2019;11(6):254.
4. Li J, Gong L, Fink JB. The Ratio of Nasal Cannula Gas Flow to Patient Inspiratory Flow on Trans-nasal Pulmonary Aerosol Delivery for Adults: An in Vitro Study. *Pharmaceutics*. 2019; 11(5):225.
5. Li J, Wu W, Fink JB. In vitro comparison between inspiration synchronized and continuous vibrating mesh nebulizer during trans-nasal aerosol delivery. Intensive Care Med Exp. 2020;8(1):6
6. Li J, Lucas Williams, Fink JB. The Impacts of High-flow Nasal Cannula Device, Nebulizer Type and Its Placement on Trans-nasal Aerosol Drug Delivery. Respiratory care (in press)
7. Alcoforado L, Ari A, Barcelar JM, Brandao SCS, Fink JB, de Andrade AD. Impact of gas flow and humidity on trans-nasal aerosol deposition via nasal cannula in adults: a randomized cross-over study. Pharmaceutics. 2019;11(7): 320.
8. Li J, Chen Y, Ehrmann S, Wu J, Xie L, Fink JB. A randomized controlled trial to compare the effects of flow rates on inhaled bronchodilator delivery via high-flow nasal cannula for COPD and asthma patients. Pharmaceutics (in press)

Recommendations 3.7

Voting for recommendations 3.7

**Recommendations 3.7**	Using heliox to deliver aerosol via high-flow nasal cannula is not

	recommended
**Evidence**	In vitro[1]
	If you have additional evidence, please provide references:
**Likert score of 1-9**	□1 □2 □3 □4 □5 □6 □7 □8 □9<br>1= absolutely disagree, 9 = absolutely agree
**Comments**	
**Suggestions for future research**	

Table 3.7. In vitro studies comparing aerosol delivery via Heliox vs O_2

Author, year	Study type	Breathing pattern	Flow (LPM)	Inhaled Dose %		P
				O_2	Heliox	
Dailey, 2017[1]	In vitro	Vt 500ml, RR 16bpm, Inspiratory Flow 24lpm	10	26.7 ± 1.3	27.4 ± .4	0.7
			30	11.6± 1.2	14.2± .9	0.1
			50	3.5 ± .2	5.9 ± 1.7	0.1
		Vt 750ml, RR 30bpm, Inspiratory Flow 45lpm	10	13 ± 3	17 ± 3	0.056
			30	33± 5	35 ± 5	0.6

	50	25 ± 2	30 ± 4	0.056

Vt, tidal volume; RR, respiratory rates.

1. Dailey PA, Harwood R, Walsh K, Fink JB, Thayer T, Gagnon G, et al. Aerosol delivery through adult high flow nasal cannula with heliox and oxygen. Respir Care. 2017;62(9):1186–92.

Recommendations 3.8

Voting for recommendations 3.8

**Recommendations 3.8**	Using dry gas to deliver aerosol via high-flow nasal cannula has been shown to improve aerosol delivery efficiency, however considering the discomfort and the potential harms, routine use of dry gas to deliver aerosol via high-flow nasal cannula is not recommended.
**Evidence**	In vivo[1]
	If you have additional evidence, please provide references:
**Likert score of 1-9**	□1 □2 □3 □4 □5 □6 □7 □8 □9<br>1= absolutely disagree, 9 = absolutely agree
**Comments**	
**Suggestions for future research**	

Table 3.8. Trans-nasal aerosol delivery with dry vs humidified gas

	Flow	Lung deposition %	P

Author, year	Study type	Population	(LPM)	Dry gas	Humidified gas	
Alcoforado, 2019[1]	In vivo	Adult	10	18.86±10.01	17.23±6.78	NS.
			30	13.16±6.78	5.71±2.04	.015
			50	8.59±2.54	3.46±1.24	＜.001

NS, not significant

1. Alcoforado L, Ari A, Barcelar JM, Brandao SCS, Fink JB, de Andrade AD. Impact of gas flow and humidity on trans-nasal aerosol deposition via nasal cannula in adults: a randomized cross-over study. Pharmaceutics. 2019;11(7): 320.

Recommendations 3.9

Voting for recommendations 3.9

**Recommendations 3.9**	When gas flow exceeds patient inspiratory flow, open mouth breathing reduces inhaled dose. Discontinuing aerosol via high-flow nasal cannula to mouth breathing patients is not recommended.	
**Evidence**	In vitro[1]	
	If you have additional evidence, please provide references:	
**Likert score of 1-9**	□1 □2 □3 □4 □5 □6 □7 □8 □9<br>1= absolutely disagree, 9 = absolutely agree	
**Comments**		
**Suggestions for future research**		

Table 3.9. Trans-nasal aerosol delivery with open vs closed mouth breathing

Author, year	Study type	Breathing pattern	Flow (LPM)	Lung deposition %	
				Closed mouth breathing	Open mouth breathing
Réminiac, 2016[1]	In vitro	Quiet breathing	30	6.7	4.6
			45	3.5	2.6
			60	3	2

1.Réminiac F, Vecellio L, Heuze-Vourc'h N, Petitcollin A, Respaud R, Cabrera M, et al. Aerosol therapy in adults receiving high flow nasal cannula oxygen therapy. J Aerosol Med Pulm Drug Deliv. 2016;29(2): 134–41.

Recommendations 3.10

Voting for recommendations 3.10

**Recommendations 3.10**	For trans-nasal aerosol delivery, Optiflow is preferred over Airvo2 with vibrating mesh nebulizer placed at the inlet of humidifier.
**Evidence**	In vitro[1]
	If you have additional evidence, please provide references:
**Likert score of 1-9**	□1 □2 □3 □4 □5 □6 □7 □8 □9<br>1= absolutely disagree, 9 = absolutely agree
**Comments**	
**Suggestions for future research**	

Table 3.10. Comparison of inhaled doses with different HFNC set-ups using VMN.

	Nebulizer	Breathing	HFNC flow	Inhaled dose (%)	*P*

Author, year	Study type	Placement	pattern		Optiflow™	Airvo 2 with AirSpiral circuit	Airvo 2 with 900PT501 circuit	
Li,2021[1]	In vitro	At the humidifier	Quiet	10 L/min	19.8 ± .8 [a,b]	15.9 ± .2	12.8 ± .6	<.001
				20 L/min	16.5 ± .8 [a,b]	12.0 ± .3	10.9 ± .5	<.001
				40 L/min	8.8 ± 1.0 [a,b]	5.2 ± .5	5.8 ± .3	.001
				60 L/min	6.3 ±.2 [a,b]	2.7 ± .3	3.1 ± .2	<.001
			Distressed	10 L/min	17.5 ± 1.0	18.6 ± .8 [b]	16.2 ± .5	.027
				20 L/min	17.8 ± .2 [b]	19.8 ± 1.0 [b]	14.5 ± .9	.001
				40 L/min	16.5 ± .3 [a,b]	9.1 ± .3 [b]	5.7 ± .3	<.001
				60 L/min	9.8 ± .7 [a,b]	8.2 ± .3 [b]	4.5 ± .1	<.001
		Proximal to nasal cannula	Quiet	10 L/min	12.0 ± .2 [a,b]	10.1 ± .3	10.2 ± .3	<.001
				20 L/min	6.7 ±.4 [b]	6.8 ±.2 [b]	5.7 ± .3	.006
				40 L/min	3.9 ± .3 [b]	3.8 ± .2	3.3 ± .06	.025
				60 L/min	2.0 ± .1	2.0 ± .1	2.1 ± .2	.440
			Distressed	10 L/min	17.8 ± .5 [b]	18.5 ± .5 [b]	13.6 ± .4	<.001
				20 L/min	13.6 ± .2 [a,b]	10.6 ± .1 [b]	8.0 ± .3	<.001
				40 L/min	8.2 ± .6 [a,b]	6.8 ± .4	5.9 ± .5	.004
				60 L/min	3.8 ± .1 [a]	3.2 ± .1	3.7 ± .1 [a]	.002

HFNC, high-flow nasal cannula; VMN, vibrating mesh nebulizer.

[a] *P*<.05 when compared to Airvo2 with AirSpiral circuit

[b] *P*<.05 when compared to Airvo2 with 900PT501 circuit

1. Li J, Lucas Williams, Fink JB. The Impacts of High-flow Nasal Cannula Device, Nebulizer Type and Its Placement on Trans-nasal Aerosol Drug Delivery. Respiratory care (in press)

Appendix 5

SURVERY REPORT OF ROUND 1

Section 1: Aerosol Delivery via Invasive Ventilation for Adult Patients

Recommendation 1.1： During invasive ventilation, vibrating mesh nebulizer is more efficient in aerosol delivery than continuous jet nebulizer, with no influence on flows or fraction of inspired oxygen. When available, vibrating mesh nebulizer is preferred over continuous jet nebulizer.

Distribution of voting scores

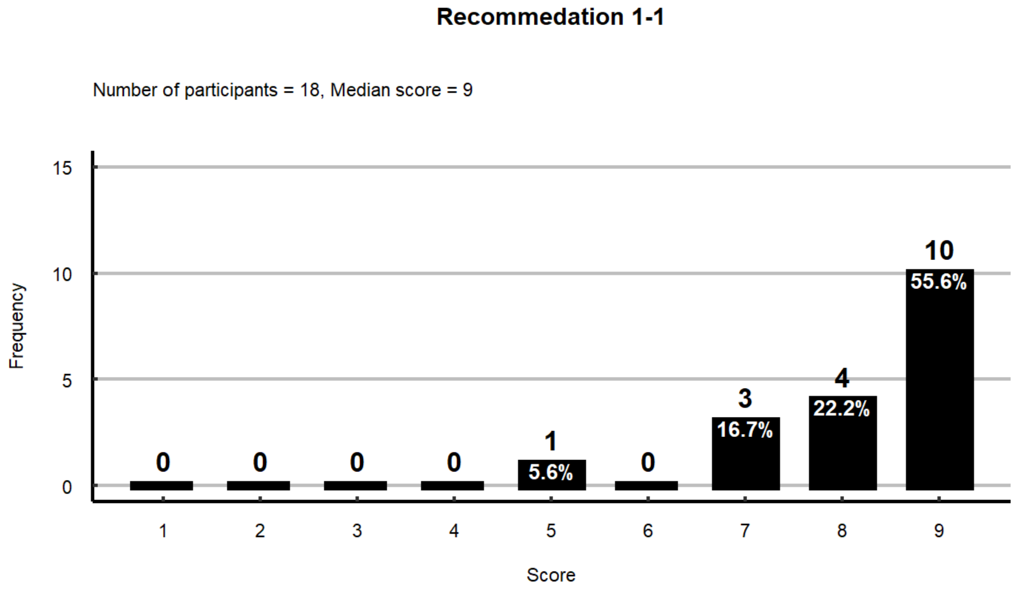

Comments of voting panelists

1. Vibrating mesh nebulizers generate higher inhaled dose than jet nebulizers. Additionally, the absence of influence on ventilator settings and FiO_2 represents an important benefit over jet nebulizer.

2. Yes, but for high viscous drugs, mesh nebulizer could not be able to nebulize drug.

3. The word efficient is misleading. They have a smaller residual volume and are more convenient. Ventilators with jet neb port actually have no interference with flow or at least tidal volume (PMID: 24917450) (or FiO_2 I think but unaware of a study which measured this). Continuous jet nebulizers should just not be used anymore in ventilated patients. There is a need for a recommendation in this regard, thus their performance compared to other systems is not relevant.

4. Most evidence is from bench studies. Although mesh nebulizer use is attractive and

widely used, a concern is device failure when used with continuous delivery.

5. Some concerns related to the evidence for effects of flows or FiO_2 – depending on the positioning of Jet or Mesh in the circuit – as well as – if during mechanical ventilation or CPAP (delivered by ventilator or high flows) – finally not clear evidence on the effects of assisted breathing and different types of assisted breathing modes – most of studies in vitro – with different locations of the nebulizers or mesh as comparison – with bias flow or not – which may make a major difference in the interpretation of the data – finally the role of HHWH should be also considered.

6. I agree with the recommendation concerning the nebulization of bronchodilators and antibiotics (I strongly suggest to provide this information in the recommendation – outlined in yellow). I also suggest to add the following paragraph as a note of caution: An unexpected 30% rate of premature cessation of aerosol production has been reported with the Aerogen mesh nebulizer [see Gowda AA, Cuccia D, Smaldone GC. Reliability of vibrating mesh technology. Respir Care 2016; 62:65–69.]. Failure was associated with a wide range of residual volume averaging 21.3% of the charge compared with 1.5% of the charge when the nebulization was successful. In contrast, older jet nebulizers have been shown to be reliable with repeated use [see Skaria S, Smaldone GC. Omron NE U22: comparison between vibrating mesh and jet nebulizer. J Aerosol Med Pulm Drug Deliv 2010; 23: 173–180].

This note of caution comes from recommendations made by the European Investigator Network for Nebulized Antibiotics in Ventilator Associated Pneumonia (ENAVAP) in a recently published paper : see Monsel A, Torres A, Zhu Y, Pugin J, Rello J, Rouby JJ; European Investigators Network for Nebulized Antibiotics in Ventilator-associated Pneumonia (ENAVAP). Nebulized antibiotics for ventilator-associated pneumonia: methodological framework for future multicenter randomized controlled trials. Curr Opin Infect Dis. 2021 Apr 1;34(2):156-168, 2021

7. This is a 2-part statement. I agree with the first part. Preference is based on several factors besides efficiency.

8. More is not necessarily better. Cost is not a variable being consider. The difference in efficiency varies with position in the circuit. The in Vivo studies not necessarily support the statement.

Round 2

**Recommendations 1.1**	During invasive ventilation, vibrating mesh nebulizer is more efficient in aerosol delivery than continuous jet nebulizer, with no influence on flows or fraction of inspired oxygen. When available, vibrating mesh nebulizer is preferred over continuous jet nebulizer.
**Likert score of 1-9**	□1 □2 □3 □4 □5 □6 □7 □8 □9<br>1= absolutely disagree, 9 = absolutely agree
**Comments**	

Section 1: Aerosol Delivery via Invasive Ventilation for Adult Patients

Recommendation 1.2：During high-frequency oscillatory ventilation, vibrating mesh nebulizer is more efficient in aerosol delivery than continuous jet nebulizer, with no influence on flows or fraction of inspired oxygen. When available, vibrating mesh nebulizer is recommended over continuous jet nebulizer.

Distribution of voting scores

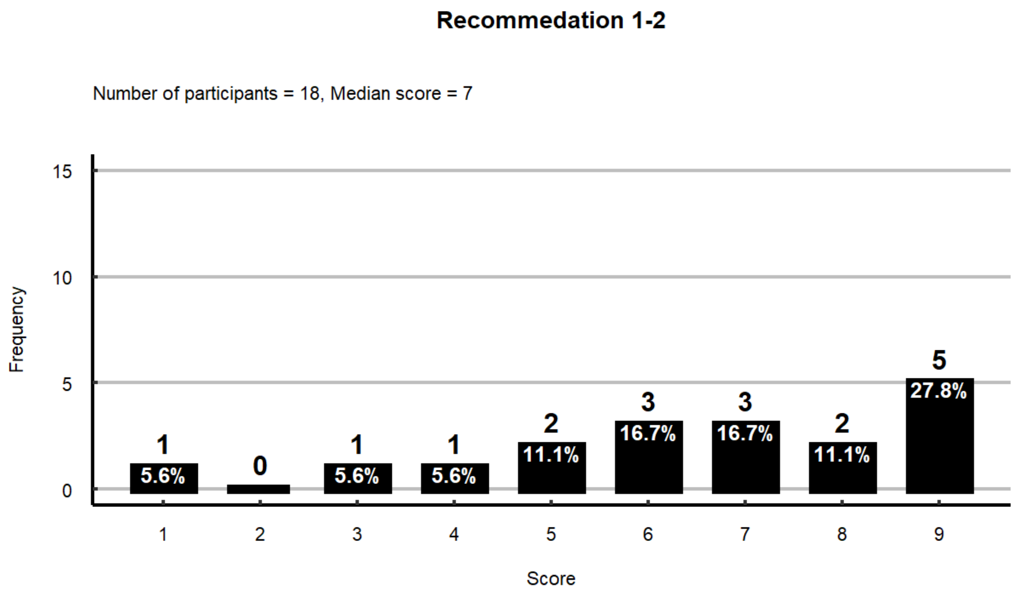

Comments of voting panelists

1. High Frequency ventilatory oscillation is not very much used in clinical practice.
2. Limited in vitro data suggested better drug delivery using vibrating mesh nebulizer during high-frequency oscillatory ventilation. The absence of influence on ventilator settings and FiO2 is an important benefit.
3. Need to be confirm by invivo datas (animal or/and human) – only one study.
4. To my knowledge there is nearly no more indication of HFO. I would maybe not make any recommendation, especially as there is nearly no data.
5. A single in vitro study does not support such a recommendation. Because HFOV is a part of invasive mechanical ventilation, and this study showed greater efficacy of aerosol with VMN, I suggest including this reference in recommendation 1.1.
6. No sufficient data to conclude.
7. Only one in-vitro study.
8. The only evidence is a bench study on salbutamol delivery. There is no indication for aerosol bronchodilator delivery with HFOV. Neither HFOV nor bronchodilators are

indicated for ARDS.

9. This is just coming from very few in vitro studies – can you add also Respiratory Care - February 2020, 65 (2) 227-232; DOI: https://doi.org/10.4187/respcare.07050

10. The recommendation should include the nebulization of bronchodilators and antibiotics (outlined in yellow). There is very little evidence for this recommendation (a single in vitro study). In addition, HFO is now used exclusively in neonates with acute respiratory failure. Since the publication of the OSCILLATE trial showing an excess in mortality in ARDS patients treated by HFO (see Ferguson et al OSCILLATE trial NEJM 2013), HFO is not used anymore in adult critically ill patients. I suggest either to remove this recommendation or to clearly indicate that HFO is not anymore used in adults with ARDS.

11. Depends on the indication.

12. Recommendation needs to specify location because the jet right before the ETT delivers 5-fold more drug than VMN at the ventilator.

Round 2

**Recommendations 1.2**	During high-frequency oscillatory ventilation, vibrating mesh nebulizer is more efficient in aerosol delivery than continuous jet nebulizer, with no influence on flows or fraction of inspired oxygen. When available, vibrating mesh nebulizer is recommended over continuous jet nebulizer.
**Likert score of 1-9**	□1 □2 □3 □4 □5 □6 □7 □8 □9<br>1= absolutely disagree, 9 = absolutely agree
**Comments**	

Section 1: Aerosol Delivery via Invasive Ventilation for Adult Patients

Recommendation 1.3： Based on variation of the reported inhaled doses and lack of definitive clinical outcomes, there is no recommendation for metered dose inhaler and spacer versus vibrating mesh nebulizer.

Distribution of voting scores

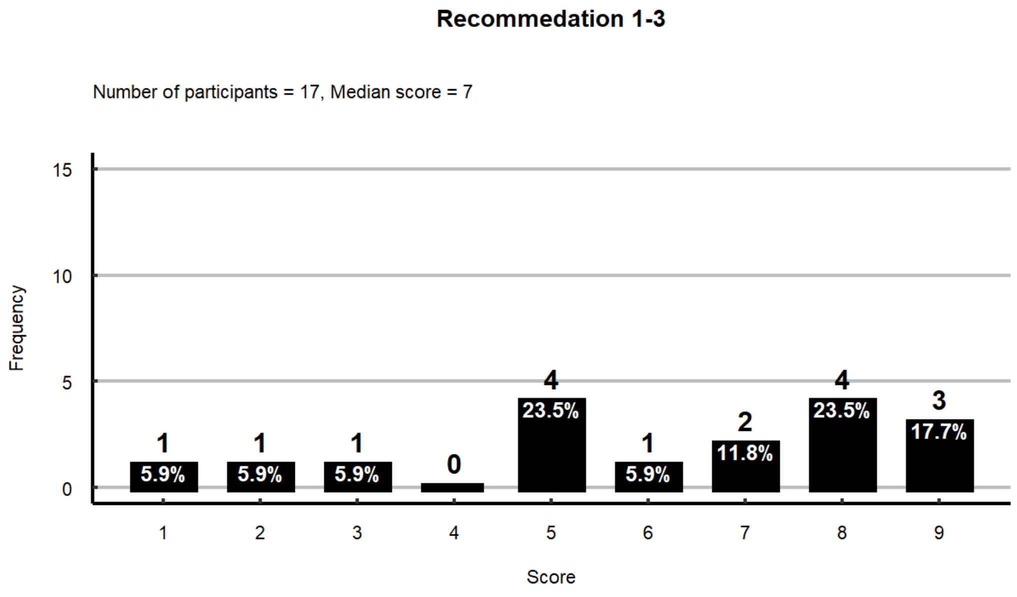

Comments of voting panelists

1. This practice has to be abandoned due to high variable efficacy and lung deposition but it is easy to perform.

2. In vitro studies reported non significant and non reported data (e.g. p value). However, some clinical studies showed a trend in favor of metered dose inhaler and spacer.

3. For bronchodilatators where there is a large window of therapeutic effect, 4-5 puffs of 100µg is recommended in clinical practice vs 2.5mg by nebulizer to obtain similar clinical efficiency (not similar deposition). Duarte AG, Momii K, Bidani A. Bronchodilator therapy with metered-dose inhaler and spacer versus nebulizer in mechanically ventilated patients: comparison of magnitude and duration of response. Respir Care. 2000 Jul;45(7):817-23. PMID: 10926377

4. Based on these various conditions, no difference can be really found between these modalities.There are more data than for 1.2.

5. This boils down to cost of pMDI vs mesh nebulizer.

6. Really, many options are available, and newer aerosol technologies are being used with some success. Studies have suggested that the pressurized metered-dose inhaler

(pMDI) and jet-type nebulizer are essentially equal in the efficacy of delivering aerosol - Chest1997;112(1):24–28 - There are still data lacking overall, however, relating to specific aerosol therapy delivery recommendations during mechanical ventilation. J Aerosol Med Pulm Drug Deliv 2008;21(1):45–60 - Am J Respir Crit Care Med 1995;152(4):1391–1394. The cost of pMDI is high and not easy to use at the bedside. No difference has been reported in contamination. The study by Dubosky et al - Respir Care 2017;62(4):391–395 - suggests that the use of a vibrating mesh nebulizer during mechanical ventilation is an alternative to pMDI use without increasing the risk of VAP. Moreover, the vibrating mesh nebulizer is designed such that its particle size is more consistent, less condensate develops within the circuit during treatments, and residual waste is negligible. Additionally, in analog models, the vibrating mesh nebulizer has been shown to be superior to the traditional jet nebulizer in the delivery of aerosol during mechanical ventilation - Respiratory care 2010;55(7):845–851 - The vibrating mesh nebulizer should be considered a safe option for the delivery of aerosol therapy during mechanical ventilation. Of course, future studies are needed to evaluate the latest nebulizers as their use is increased.

7. I strongly disagree with this recommendation if it concerns the nebulization of antibiotics to treat tracheobronchitis- and ventilator-associated pneumonia. There is a lot of evidence that high doses of amikacin, tobramycin, colistimethate sodium and polymyxin B should be nebulized to treat efficiently Ventilator-Associated Pneumonia (see recent references)

8. This recommendation applies only for bronchodilator therapy.

9. This table has to be re-done you cannot compare efficiency of drug delivery when the loading/delivered dose are diffferent. In addition clinical studies included in the review show equivalent clinical outcomes.

Round 2

**Recommendations 1.3**	Based on variation of the reported inhaled doses and lack of definitive clinical outcomes, there is no recommendation for metered dose inhaler and spacer versus vibrating mesh nebulizer.
**Likert score of 1-9**	□1 □2 □3 □4 □5 □6 □7 □8 □9<br>1= absolutely disagree, 9 = absolutely agree

**Comments**	

Section 1: Aerosol Delivery via Invasive Ventilation for Adult Patients

Recommendation 1.4： When placed close to the ventilator, the vibrating mesh nebulizer is more efficient in aerosol delivery than ultrasonic nebulizer. When nebulizer is placed at the inspiratory limb before Y-piece, there is no recommendation for vibrating mesh nebulizer versus ultrasonic nebulizer.

Distribution of voting scores

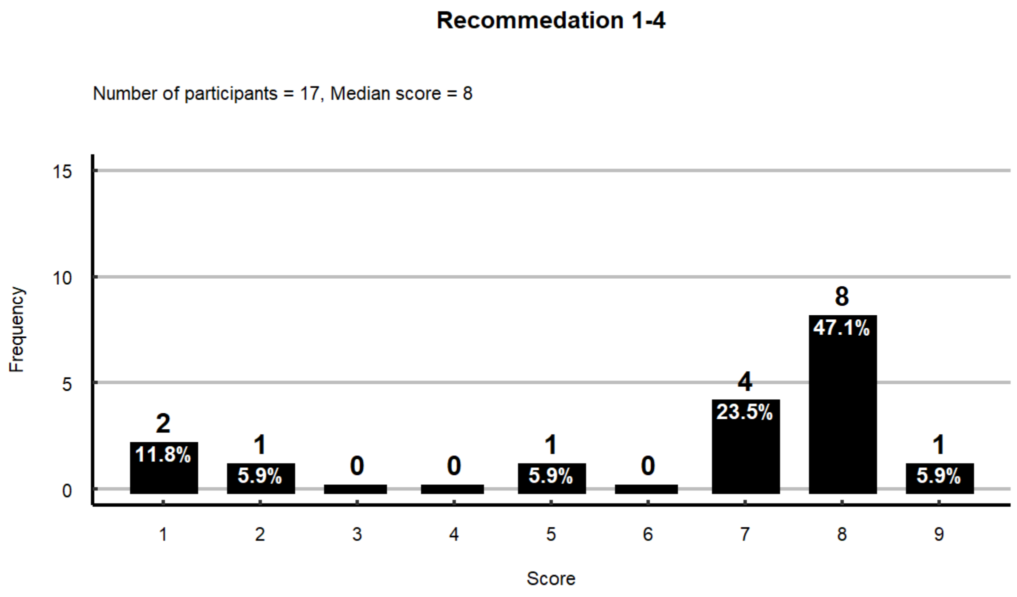

Comments of voting panelists

1. 1. As mentioned there is not much evidence.

2. Limited data suggested better drug delivery with vibrating mesh nebulizer during high-frequency oscillatory ventilation.

3. There is no rational for the types of nebulizer working differently in different locations in the circuit.

4. The results of this study (Paker et al, reference 3) should be interpreted with caution. First, as mentioned in the study, the ultrasonic device used in this study (TIS) was not designed for use with mechanical ventilation, the manual breath key on the Servo-I was used to coordinate ventilator breaths with TIS inhalations, the synchronization between TIS and Servo was difficult, which could decrease efficiency of aerosol delivery. In addition, there was a dose adjustment for VMN to taken into account the dose waste during expiration that could overestimate the results obtained by VMN. So the comparisons between the devices were not made at the same condition.

5. I suggest separating the two sentences

When nebulizer is placed at the inspiratory limb before Y-piece, there is no difference for me between vibrating mesh nebulizer versus ultrasonic nebulizer.

6. Human evidence lacking. USN not commonly used in North America.

7. My comment here “why ultrasonic nebulizer” – while I fully agree for comparison with jet nebulizer.

8. I disagree with the formulation of the recommendation and suggest to change it. The formulation of the Recommendation 1.4 is confusing. The body of in vitro evidence shows that either ultrasonic or vibrating mesh nebulizers should be placed after the conventional humidifyer and close to the Y piece and have an equivalent aerosol efficiency (see F. Ferrari, Z.H. Liu, Q. Lu, M.H. Becquemin, K. Louchahi, G. Aymard, C.H. Marquette, J.J. Rouby. Comparison of lung tissue concentrations of nebulized ceftazidime in ventilated piglets: ultrasonic versus vibrating plate nebulizers. Int Care Med 34: 1718-1723, 2008). Therefore, I suggest to reformulate the recommendation as “When placed close to the ventilator, ultrasonic and vibrating mesh nebulizers are much less efficient in aerosol delivery of bronchodilators and antibiotics than when placed close to the Y piece. Both types of nebulizers are equivalent when placed at the inspiratory limb before Y-piece.” With such a formulation , I would absolutely agree. I would also add the following reference concerning the nebulization of amikacin: Dugernier J, Wittebole X, Roeseler J, Michotte JB, Sottiaux T, Dugernier T, et al. Influence of inspiratory flow pattern and nebulizer position on aerosol delivery with a vibrating-mesh nebulizer during invasive mechanical ventilation: an in vitro analysis. J Aerosol Med Pulm Drug Deliv 2015;28:229–36.

9. Should the latter part of the statement say – there is no difference between efficiency of vibrating mesh versus ultrasonic nebulizer.

8. Instead of saying there is no recommendation we could say both are equally efficient. Remove Parker 2017 and move it to recommendation #1.2.

Round 2

**Recommendations 1.4**	~~When placed close to the ventilator, the vibrating mesh nebulizer is more efficient in aerosol delivery than ultrasonic nebulizer. When nebulizer is placed at the inspiratory limb before Y-piece, there is no recommendation for vibrating mesh nebulizer versus~~

	~~ultrasonic nebulizer.~~<br>**When placed at inspiratory limb before Y piece, the vibrating mesh nebulizer is as efficient as ultrasonic nebulizer in aerosol delivery. When nebulizer is placed proximal to ventilator, the vibrating mesh nebulizer is more efficient in aerosol delivery than ultrasonic nebulizer.**
**Likert score of 1-9**	□1 □2 □3 □4 □5 □6 □7 □8 □9<br>1= absolutely disagree, 9 = absolutely agree
**Comments**	

Section 1: Aerosol Delivery via Invasive Ventilation for Adult Patients

Recommendation 1.5： During high frequency oscillatory ventilation with nebulizer placed between Y-piece and endotracheal tube, vibrating mesh nebulizer is more efficient in aerosol delivery than ultrasonic nebulizer. When available, vibrating mesh nebulizer is recommended over ultrasonic nebulizer.

Distribution of voting scores

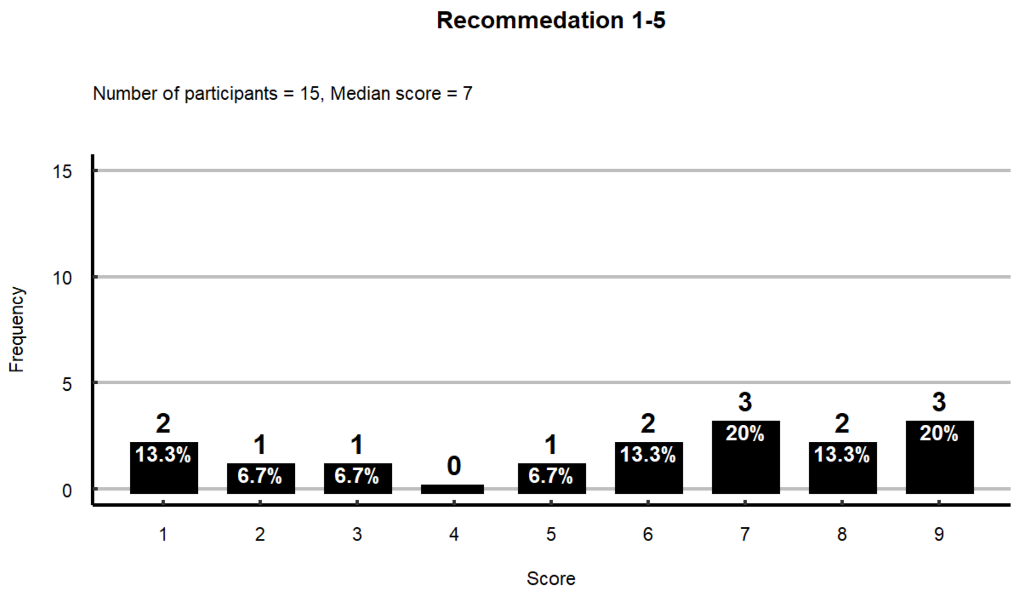

Comments of voting panelists

1. Low evidence.

2. Only one in vitro study suggested better drug delivery with vibrating mesh nebulizer during high-frequency oscillatory ventilation.

3. Only one in vitro study… but agree in term of safety regarding the dead volume of the nebulizer connexion.

4. The results presented in this study may be related to the unique devices used and not the type of nebulizer per se.

5. No sufficient data.

6. The results of this study (Paker et al, reference 3) should be interpreted with caution. The ultrasonic device used in this study (TIS) was not designed for use with mechanical ventilation, making comparison difficult.

7. Only one in-vitro study.

8. Difficult to place USN between circuit and wye.

9. Evidence lacking for aerosol delivery with HFOV. Neither HFOV nor bronchodilator

indicated for ARDS.

10. The recommendation should include the nebulization of bronchodilators and antibiotics (outlined in yellow). There is very little evidence for this recommendation (a single in vitro study). In addition, HFO is now used exclusively in neonates with acute respiratory failure. Since the publication of the OSCILLATE trial showing an excess in mortality in ARDS patients treated by HFO (see Ferguson et al OSCILLATE trial NEJM 2013), HFO is not used anymore in adult critically ill patients. I suggest either to remove this recommendation or to clearly indicate that HFO is not anymore used in adults with ARDS.

11. Depends on the indication.

12. 1.2 and 1.5 ask the same question and data should be combined.

Round 2

**Recommendations 1.5**	During high frequency oscillatory ventilation with nebulizer placed between Y-piece and endotracheal tube, vibrating mesh nebulizer is more efficient in aerosol delivery than ultrasonic nebulizer. When available, vibrating mesh nebulizer is recommended over ultrasonic nebulizer.
**Likert score of 1-9**	□1 □2 □3 □4 □5 □6 □7 □8 □9<br>1= absolutely disagree, 9 = absolutely agree
**Comments**	

Section 1: Aerosol Delivery via Invasive Ventilation for Adult Patients

Recommendation 1.6：When placed at the inspiratory limb before Y-piece, metered dose inhaler with a spacer is more efficient in aerosol delivery than the continuous jet nebulizer, with no influence on flows or fraction of inspired oxygen. When available, metered dose inhaler with spacer is recommended over continuous jet nebulizer.

Distribution of voting scores

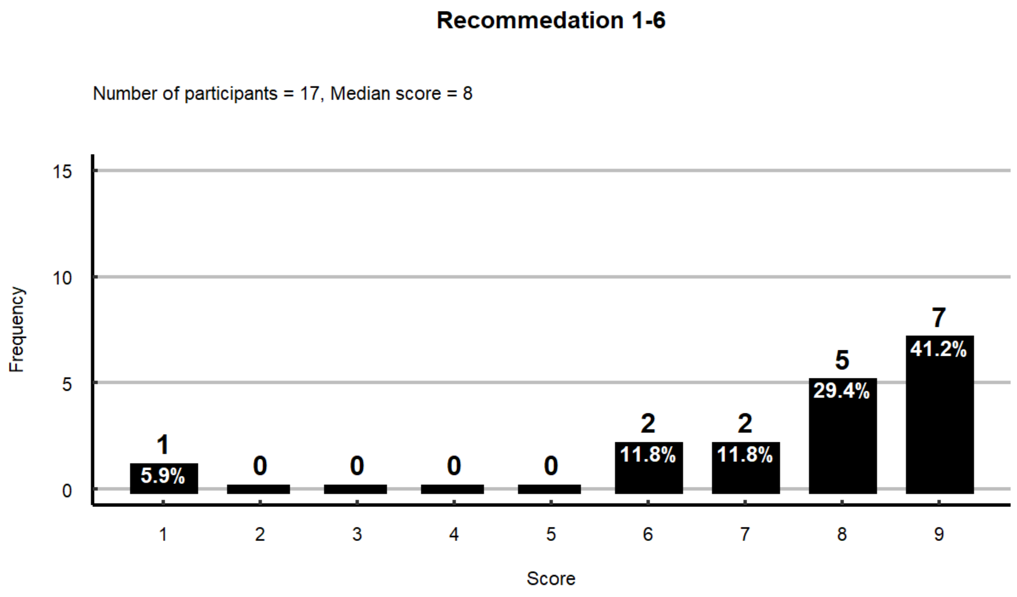

Comments of voting panelists

1. In vitro studies presented non reported data (e.g. p-value, humidification, distance before y piece). However, some clinical studies showed a trend in favor of metered dose inhaler and spacer. The absence of influence on ventilator settings or fraction of inspired oxygen is an important benefit when metered dose inhaler and spacer is used.

2. It depends when the pmdi is actuated-if the pMDI is actuated at beginning of the inspiratory phase: yes.

3. Again, continuous jet neb which exposed to uncontrolled tidal volume should not be used anymore in ventilated patients. The relative performance towards pMDI is not interesting. In terms of bronchodilation efficicieny is similar.

4. In Vivo study to study compared clinical effects

a. Inhaled fenoterol-ipratropium bromide in mechanically ventilated patients with chronic obstructive pulmonary disease. Guerin C, Chevre A, Dessirier P, Poncet T, Becquemin MH, Dequin PF, Le Guellec C, Jacques D, Fournier G. Am J Respir Crit Care Med. 1999 Apr;159(4 Pt 1):1036-42. doi: 10.1164/ajrccm.159.4.9710081.

b. Manthous CA, Hall JB, Schmidt GA, Wood LDH. Metered-dose inhaler versus nebulized albuterol in mechanically ventilated patients. American Review of Respiratory Disease 1993;148(6P+1):1567–70.

5. If the recommendation is only based on fraction of inhaled dose, it is probably true. But the clinical studies in terms of reduction of resistance did not show any difference between the 2 devices. One study (Manthous et al 1993) showed results in favor of jet nebulizer.

6. I generally agree with this statement, but wonder if it is clinically important, particularly for bronchodilator delivery. Does improved efficiency translate into better clinical outcomes? I don’t think that has been studied. For some of these efficiency studies, I think a long run for a short slide.

7. The issue related to comparison among dosage and specific ventilatory modes and setting investigated make some weakness in the direct comparison – further mainly in vitro studies – not much clinical studies on this specific topic.

8. I agree with the recommendation if it concerns the nebulization of bronchodilators (I strongly suggest to provide this information in the recommendation – outlined in yellow). The use of metered dose inhaler cannot be recommended for the nebulization of antibiotics.

9. Depends on indication. Some therapies are not available in MDI formulations. Cost of treatment is an additional consideration.

10. In the end what matters is how much drug reaches the lungs. Therefore, the comparison should be made mcg to mcg.

Round 2

**Recommendations 1.6**	When placed at the inspiratory limb before Y-piece, metered dose inhaler with a spacer is more efficient in aerosol delivery than the continuous jet nebulizer, with no influence on flows or fraction of inspired oxygen. When available, metered dose inhaler with spacer is recommended over continuous jet nebulizer.
**Likert score of 1-9**	□1 □2 □3 □4 □5 □6 □7 □8 □9<br>1= absolutely disagree, 9 = absolutely agree
**Comments**	

Additional references：

1. Inhaled fenoterol-ipratropium bromide in mechanically ventilated patients with chronic obstructive pulmonary disease. Guerin C, Chevre A, Dessirier P, Poncet T, Becquemin MH, Dequin PF, Le Guellec C, Jacques D, Fournier G. Am J Respir Crit Care Med. 1999 Apr;159(4 Pt 1):1036-42. doi: 10.1164/ajrccm.159.4.9710081.
2. Manthous CA, Hall JB, Schmidt GA, Wood LDH. Metered-dose inhaler versus nebulized albuterol in mechanically ventilated patients. American Review of Respiratory Disease 1993;148(6P+1):1567–70.

Section 1: Aerosol Delivery via Invasive Ventilation for Adult Patients

Recommendation 1.7： When placed at 12-15cm from the Y-piece in the inspiratory limb, ultrasonic nebulizer is more efficient in aerosol delivery than the continuous jet nebulizer, with no influence on flows or fraction of inspired oxygen. When available, ultrasonic nebulizer is recommended over continuous jet nebulizer.

Distribution of voting scores

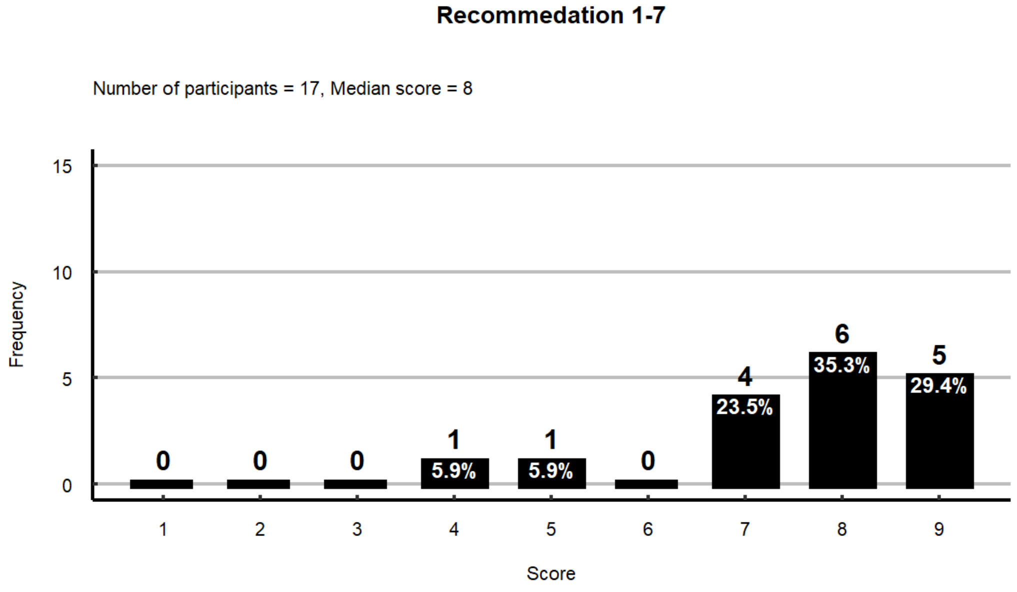

Comments of voting panelists

1. Limited data suggested better drug delivery with ultrasonic nebulizer. The absence of influence on ventilator settings and FiO2 is an important benefit when ultrasonic nebulizer is used.

2. Agree for bronchodilatators, but disagree for high viscous solution or suspension. There is a risk of active compound damage with ultrasonic nebuizer and a risk of no nebulization due to high viscosity. But I agree regarding the security for the patient where there is a risk when using continuous jet nebulizer.

3. Same comment. Continuous jet is to be avoided all together.

4. Not commonly utilized.

5. Human data lacking. USN not commonly used, thus clinical implication unclear. Evidence outdated; not sure if these devices are still available.

6. Not clear evidence of the statement from studies reported – mainly in vitro.

7. Recommendation 1.7 is mostly redundant with recommendation 1.1. Both recommendations could be simplified in a single recommendation stating “When

placed at 12-15cm from the Y-piece in the inspiratory limb, ultrasonic nebulizer and vibrating mesh nebulizers are more efficient in aerosol delivery than the continuous jet nebulizer, with no influence on flows or fraction of inspired oxygen. When available, ultrasonic nebulizer is recommended over continuous jet nebulizer." I agree with the recommendation which concerns the nebulization of bronchodilators and antibiotics (I strongly suggest to provide this information in the recommendation – outlined in yellow).

8. Not enough evidence to make a recommendation. Other factors include cost and maintenance

9. Again cost/efficiency is not considered in this recommendation

Round 2

**Recommendations 1.7**	When placed at 12-15cm from the Y-piece in the inspiratory limb, ultrasonic nebulizer is more efficient in aerosol delivery than the continuous jet nebulizer, with no influence on flows or fraction of inspired oxygen. When available, ultrasonic nebulizer is recommended over continuous jet nebulizer.
**Likert score of 1-9**	□1 □2 □3 □4 □5 □6 □7 □8 □9<br>1= absolutely disagree, 9 = absolutely agree
**Comments**	

Section 1: Aerosol Delivery via Invasive Ventilation for Adult Patients

Recommendation 1.8： When vibrating mesh nebulizer is utilized during invasive ventilation, it is recommended to be placed at the inlet of humidifier.

Distribution of voting scores

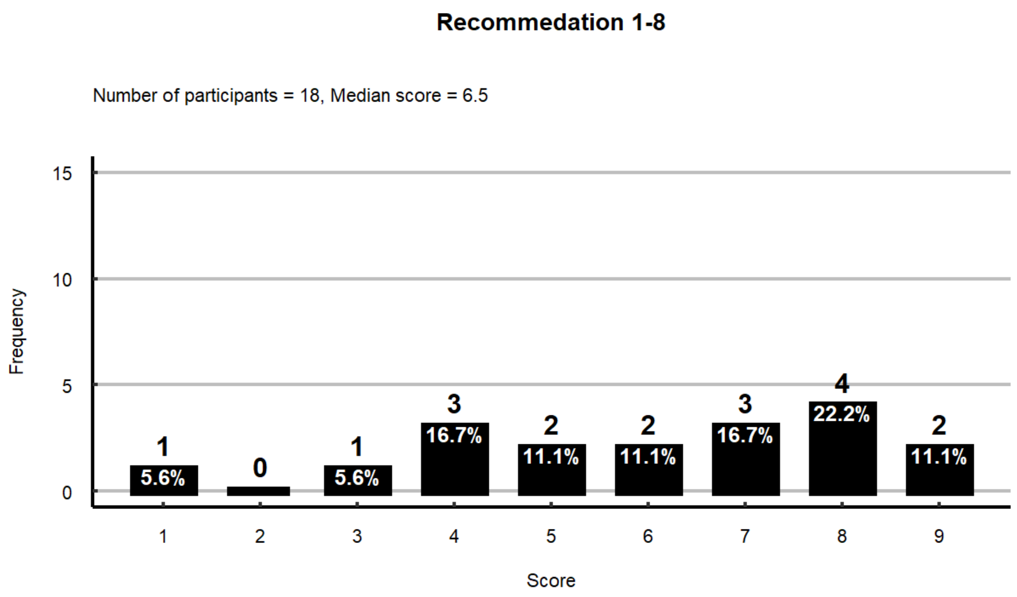

Comments of voting panelists

1. For antibiotic prescription, humidifiers need to be removed.

2. Depending on the duration of administration: can used without humidification?

3. There are no clinical studies to correlate in vitro data.

4. There is no clinical data supporting this recommendation whereas clinical data has been obtained with different nebulizer placement. Furthermore, there is a risk to drug damage due to high temperature in the humidifier.

5. Optimal placement depends on bias flow.

6. Based on the results of the in vitro studies, the ""humidifier inlet"" position with heated humidifier was only studied in reference 3 (Anderson 2007). This study showed that the ""inlet or outlet"" position had better aerosol delivery efficiency than the ""immediately before the Y-piece"" position and the position between the ETT and the Y-piece, but the position 15-40 cm before the Y-piece was not studied.

Most studies have shown a greater inhaled mass when the VMN was positioned near the ventilator, but not necessarily at the "inlet" position. Would it be more suitable to recommend the position "proximal to ventilator"?

7. With bias flow.

8. Human data lacking.

9. Also after Y – mesh is very efficient as well as jet nebulizer.

10. The scoring of recommendation 1.8 depends on the scoring of recommendation 1.15. I strongly suggest to place recommendation 1.15 before recommendation 1.8. As I disagree with placing any nebulizer before the conventional heated-humidifier, I score recommendations 1.9 and 1.10 as “1= absolutely disagree”

11. Need more clinical data to support this recommendation.

Round 2

**Recommendations 1.8**	When vibrating mesh nebulizer is utilized during invasive ventilation, it is recommended to be placed ~~at the inlet of humidifier~~ **proximal to ventilator**.
**Likert score of 1-9**	□1 □2 □3 □4 □5 □6 □7 □8 □9<br>1= absolutely disagree, 9 = absolutely agree
**Comments**	

Section 1: Aerosol Delivery via Invasive Ventilation for Adult Patients

Recommendation 1.9： When continuous jet nebulizer is utilized during invasive ventilation, it is recommended to be placed proximal to the ventilator. When inspiration synchronized jet nebulizer is used, it is recommended to be placed at the inspiration limb before Y-piece.

Distribution of voting scores

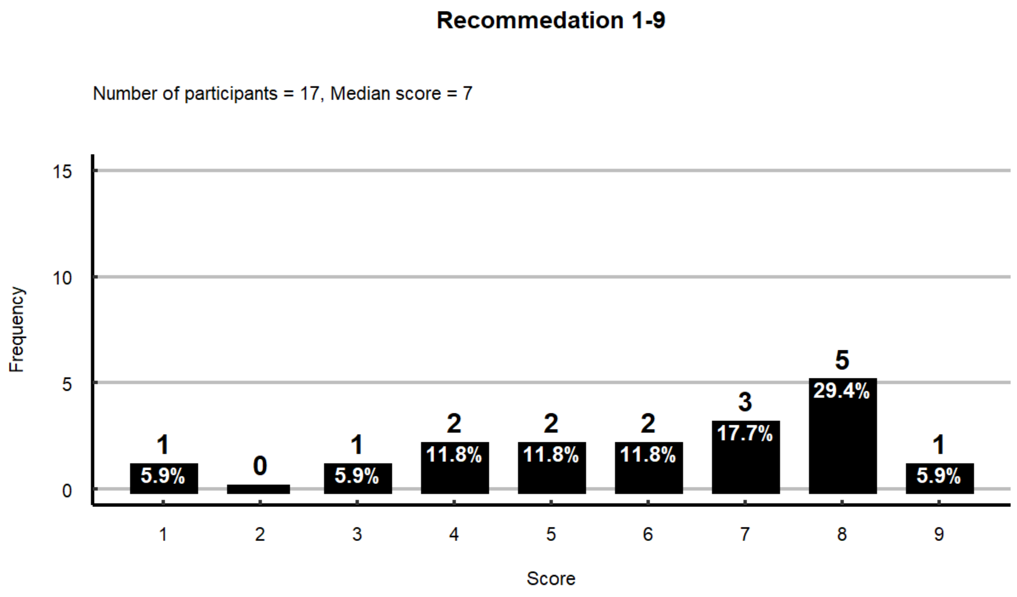

Comments of voting panelists

1. Synchronization is problematic for antibiotic administration.

2. Clinical studies are needed.

3. Limited data suggested better drug delivery with synchronized jet nebulizer placed before Y-piece. Moreover, there is no study using recent ventilators (synchronization system on current ventilators probably better than on the Siemens Servo 900C).

4. Continuous should not be used. Synchonized, is actually not well synchronized in many ventilator settings thus placement proximal towards the ventilator should be preferred. All those question on optimal placement need to be separated depending on the drug one wants to deliver: for bronchodilators, efficiency is high in any location so there is no need to favor one and we should avoid making recommandations. For antibiotics the story is different.

5. “When inspiration synchronized jet nebulizer is used, it is recommended to be placed at the inspiration limb before Y-piece” is not support by sufficient evidence if it is based on one early study3.

6. “Continuous” has 2 meanings. It could mean that neb is not breath actuated or it could mean that aerosol is delivered continuously over a long time. No human data.
7. Jet nebulizer is also efficient when positioned at the ventilator – further it depends on syncronization or not of jet nebulizer as well as presence or not of active humidification on or off – more complex issue for discussion.
8. Jet nebulizer is also efficient when positioned at the ventilator – further it depends on syncronization or not of jet nebulizer as well as presence or not of active humidification on or off – more complex issue for discussion.
9. The scoring of the first part of the recommendation 1.9 depends on the scoring of recommendation 1.15. I strongly suggest to place recommendation 1.15 before recommendation 1.8. As I disagree with placing any nebulizer before the conventional heated-humidifyer, I score the first part of recommendations 1.9 as “1= absolutely disagree”.
Inspiration synchronized jet nebulization can be used for aerosol delivery of bronchodilators but not for aerosol delivery of antibiotics (see references Rouby JJ, Monsel A, Ehrmann S, Bouglé A, Laterre PF. The INHALE trial: multiple reasons for a negative result. Lancet Infect Dis. 20:778-779, 2020 and Rouby JJ, Monsel A, Leone M, Mimoz O, Laterre PF, Pugin J. The IASIS, INHALE and VAPORISE trials. Reasons for a triple failure: study design, aminoglycosides dosing and technique of nebulisation. Anaesth Crit Care Pain Med. 39 : 179-183, 2020). Therefore, I suggest to add for aerosol delivery of bronchodilators in the second part of the recommendation. With this formulation, I score 9 = absolutely agree the second part of the recommendation. It seems to me also necessary to add “it is not recommended to use inspiration synchronized jet nebulizer for aerosol delivery of antibiotics”. For clarity, I suggest to split recommendation 1.9 into three recommendations:
- When continuous jet nebulizer is utilized during invasive ventilation for aerosol delivery of bronchodilators and antibiotics, it is recommended to be placed proximal to the ventilator.
- When inspiration synchronized jet nebulizer is used for aerosol delivery of bronchodilators, it is recommended to be placed at the inspiration limb before Y-piece.
- It is not recommended to use inspiration synchronized jet nebulizer for aerosol delivery of antibiotics.

10. The differences are not likely to be clinically meaningful.
11. Reference #2 is closer to clinical practice and showed no difference.Rec 1.10

discusses bias flow, maybe this should too.

Round 2

**Recommendations 1.9**	When continuous jet nebulizer is utilized during invasive ventilation, it is recommended to be placed proximal to the ventilator. When inspiration synchronized jet nebulizer is used, it is recommended to be placed at the inspiration limb before Y-piece.
**Likert score of 1-9**	□1 □2 □3 □4 □5 □6 □7 □8 □9<br>1= absolutely disagree, 9 = absolutely agree
**Comments**	

Additional references：

1. Zhang C, Mi J, Zhang Z, et al. The Clinical Practice and Best Aerosol Delivery Location in Intubated and Mechanically Ventilated Patients: A Randomized Clinical Trial. Biomed Res Int. 2021; 2021:6671671.

Section 1: Aerosol Delivery via Invasive Ventilation for Adult Patients

Recommendation 1.10： When ultrasonic nebulizer is utilized during invasive ventilation without bias flow, it is recommended to be placed at 15 cm from Y-piece at inspiratory limb; With bias flow, ultrasonic nebulizer is recommended to be placed at inlet of humidifier.

Distribution of voting scores

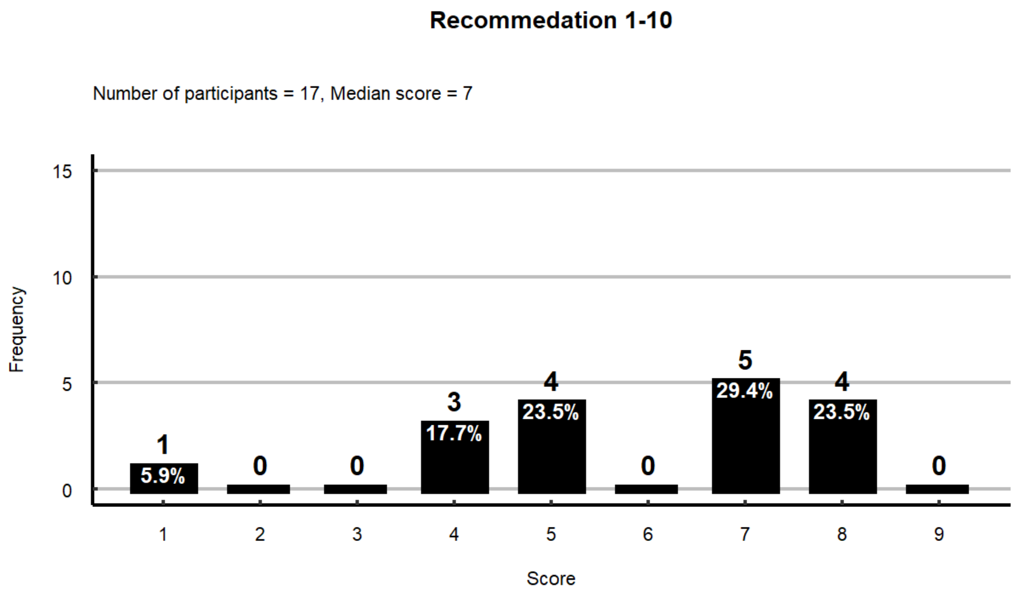

Comments of voting panelists

1. Only three studies available.

2. There are limited studies testing the ultrasonic nebulizer with different bias flows.

3. NS for clinical datas and risk of drug damage with humidifier when placing at inlet of humidifier.

4. To the best of my knowledge all modern ventilators now have a bias flow, so the question in the absence of bias flow is not really relevant. In all cases the value of the bias flow is important, because if it is very low a placement to close to the ventilator could be suboptimal.

5. Only a few data for the second part of the recommendation.

6. In clinical practice, a clinician does not necessarily distinguish between a ventilator with bias flow and that without bias flow. Without bias flow, Ari et al. showed a higher inhaled mass when the nebulizer was positioned 15 cm from the Y piece. Paker et al (reference 3) using a bias flow, but did not study the ""15 cm Y-piece"" position.

In some animal studies using an ultrasonic nebulizer positioned 40 cm from the Y-piece;

high antibiotic concentrations were found in both healthy and diseased lungs (references added above). So the position "15-40 cm from the Y-piece" might be a good compromise for ultrasonic nebulizer with and without bias flow.

7. No human data; USN not commonly used.

8. Studies in vitro with fixed ventilatory settings – not information at different settings or modes of ventilations – as well as with HHWH off or on.

9. The scoring of the recommendation 1.10 depends on the scoring of recommendation 1.15. I strongly suggest to place recommendation 1.15 before recommendation 1.8. As I disagree with placing any nebulizer before the conventional heated-humidifyer, I score the recommendations 1.10 as "1= absolutely disagree".

10. Differences are not likely to be clinically meaningful.

11. Half of the studies don't specify bias flow. The clinical study does not favor any site.

Round 2

**Recommendations 1.10**	When ultrasonic nebulizer is utilized during invasive ventilation without bias flow, it is recommended to be placed at 15 cm from Y-piece at inspiratory limb; With bias flow, ultrasonic nebulizer is recommended to be placed at inlet of humidifier.
**Likert score of 1-9**	□1 □2 □3 □4 □5 □6 □7 □8 □9<br>1= absolutely disagree, 9 = absolutely agree
**Comments**	

Section 1: Aerosol Delivery via Invasive Ventilation for Adult Patients

Recommendation 1.11 : When ultrasonic nebulizer is placed at the inspiratory limb before Y-piece, adding a spacer is recommended.

Distribution of voting scores

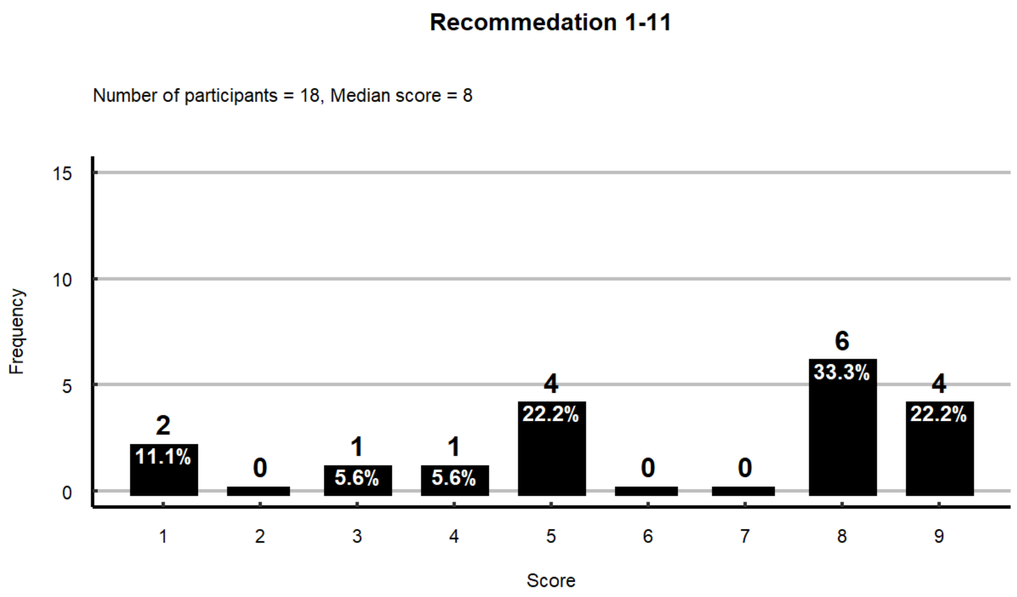

Comments of voting panelists

1. This may be true for bronchodilators. Absolutely disagree for antibiotics

2. I am not sure about this recommendation bases upon two non clinical studies.

3. Limited in vitro data suggested better drug delivery when adding a spacer.

4. Yes but for adult only.

5. Studies are too old and plus neither USN nor spacer with USN is commonly used.

6. There is no commercial option for spacer designed for use with USN. Spacer data was not done with modern vent or bias flow.

7. Common practice, but not strong evidence. No human data. Studies very old and not using contemporary devices.

8. Not clear clinical role of ultrasonic nebulizer in mechanically ventilated patients.

9. I agree with the recommendation if it concerns the nebulization of bronchodilators (I strongly suggest to provide this information in the recommendation – outlined in yellow). The use of a spacer cannot be recommended for the nebulization of antibiotics as there are no data supporting this technique. In fact, the inspiratory limb between the ultrasonic nebulizer and the Y piece serves as a spacer when continuous noninspiration-synchronized nebulization of antibiotics is performed (see the figure of the

supplementary appendix of reference Rouby JJ, Monsel A, Ehrmann S, Bouglé A, Laterre PF. The INHALE trial: multiple reasons for a negative result. Lancet Infect Dis. 20:778-779, 2020).

10. In vitro data show variability; need more clinical data.

11. Are spacers for this purpose even available? Are these devices even available?The 2 studies are almost 30 years old.

Round 2

**Recommendations 1.11**	When ultrasonic nebulizer is placed at the inspiratory limb before Y-piece, adding a spacer is recommended.
**Likert score of 1-9**	□1 □2 □3 □4 □5 □6 □7 □8 □9<br>1= absolutely disagree, 9 = absolutely agree
**Comments**	

Section 1: Aerosol Delivery via Invasive Ventilation for Adult Patients

Recommendation 1.12： When metered dose inhaler is utilized during invasive ventilation, it is recommended to be used with a spacer with volume > 150mL.

Distribution of voting scores

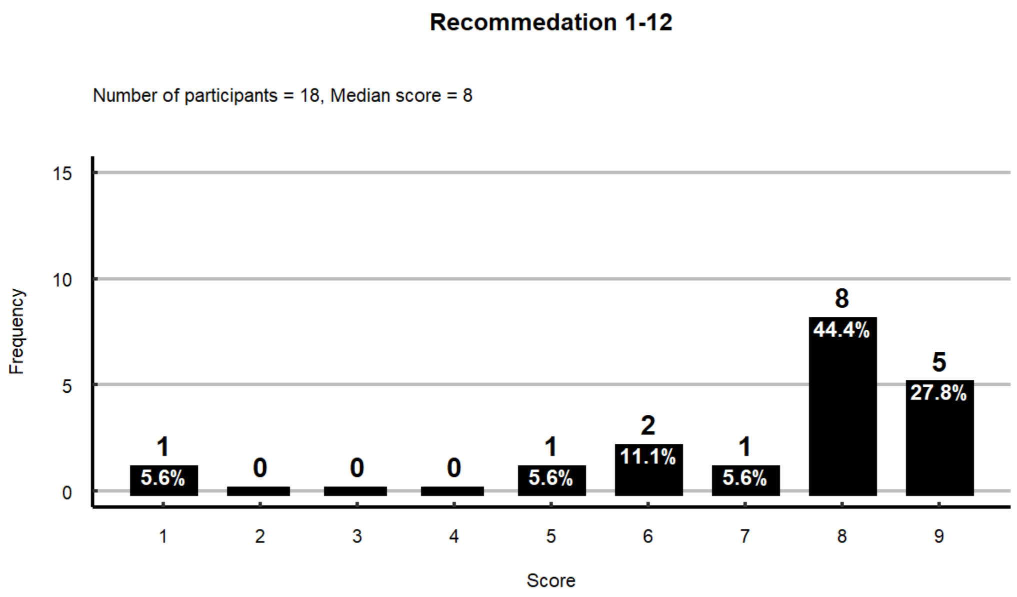

Comments of voting panelists

1. Same as 1.11

Not possible to do strong recommendations lacking large studies in vivo.

2. I agree with this recommendation.

3. Limited in vivo data showed higher drug delivery when large spacer is used.

4. Or bidirectional /small volume adapter shown to have similar delivery efficiency.

5. Mostly old data and not much human data. Evidence lacking for improved outcomes.

6. Spacer should be always used for MDI – however not easy to be performed during controlled mechanical ventilation and during assisted ventilation – no clear effects related to the ventilatory setting and mode of ventilation have been reported also in in vitro studies.

7. I agree with the recommendation if it concerns the nebulization of bronchodilators (I strongly suggest to provide this information in the recommendation – outlined in yellow). The use of a metered dose inhaler cannot be recommended for the nebulization of antibiotics.

8. There are good clinical data to support this recommendation

Manthous et al Am Rev Respir Dis 1993;148:1567; Manthous et al Chest 1995;107:210;

Dhand R et al AJRCCM 1995;151:1827; Dhand R et al AJRCCM 1996;154:189; Duarte AG et al AJRCCM 1996;154:1658; Tzoufi M et al Anesth Analg 2005;101:843; Guerin C et al AJRCCM 1999;15:1036.

9. We should use micrograms in addition to % to allow the reader comparison with other devices/situations.

Round 2

**Recommendations 1.12**	When metered dose inhaler is utilized during invasive ventilation, it is recommended to be used with a spacer with volume > 150mL.
**Likert score of 1-9**	□1 □2 □3 □4 □5 □6 □7 □8 □9<br>1= absolutely disagree, 9 = absolutely agree
**Comments**	

Section 1: Aerosol Delivery via Invasive Ventilation for Adult Patients

Recommendation 1.13： During invasive ventilation, metered dose inhaler and spacer are recommended to be placed in the inspiratory limb before the Y-piece.

Distribution of voting scores

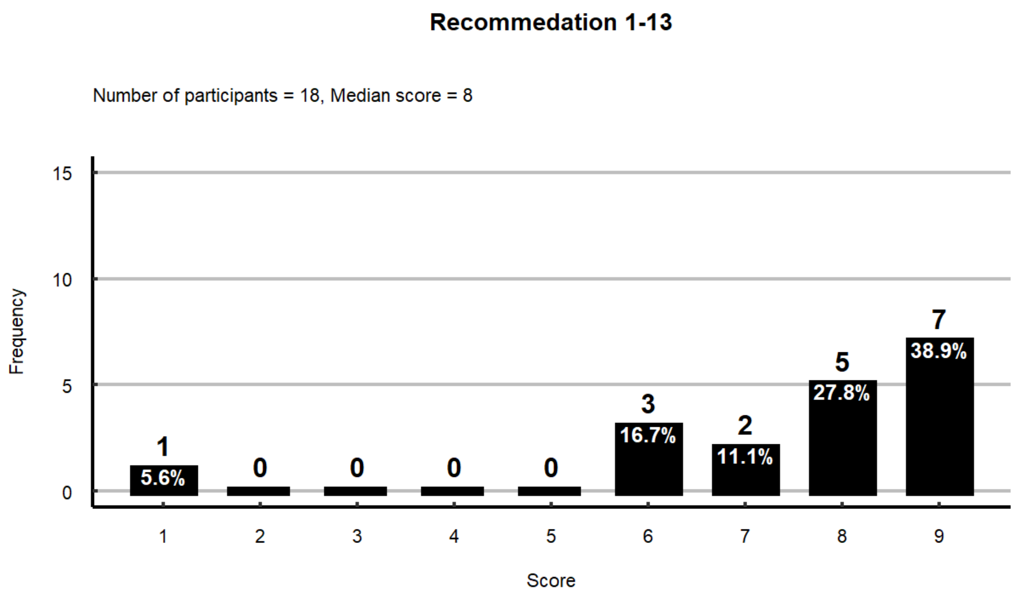

Comments of voting panelists

1. Only for bronchodilators.

2. I am not totally confident with this recommendation.

3. Limited data suggested better drug delivery with metered dose inhaler and spacer placed before the Y-piece (compared to proximal to ventilator).

4. Only for adults-For children, it could me different by placing the spacer between ETT and Y piece during actuation and then disconnect the spacer to avoid rebreathing.

5. Just before or just after the Y does not make a big difference if using a collapsible chamber to avoid dead space problems (and circuit compliance compensation problems).

6. 15 cm from Y piece.

7. This is the common practice, but evidence weak.

8. This is what it appears from the few in vitro studies available.

9. I agree with the recommendation if it concerns the nebulization of bronchodilators (I strongly suggest to provide this information in the recommendation – outlined in yellow). The use of a metered dose inhaler cannot be recommended for the nebulization of antibiotics.

10. See comments in Recommendations 1.12.
11. We should use micrograms in addition to % to allow the reader comparison with other devices/situations. The table says nebulizer instead of pMDI.

Round 2

**Recommendations 1.13**	During invasive ventilation, metered dose inhaler and spacer are recommended to be placed in the inspiratory limb before the Y-piece.
**Likert score of 1-9**	□1 □2 □3 □4 □5 □6 □7 □8 □9<br>1= absolutely disagree, 9 = absolutely agree
**Comments**	

Section 1: Aerosol Delivery via Invasive Ventilation for Adult Patients

Recommendation 1.14：During high-frequency oscillatory ventilation, nebulizers are recommended to be placed between the Y-piece and the endotracheal tube.

Distribution of voting scores

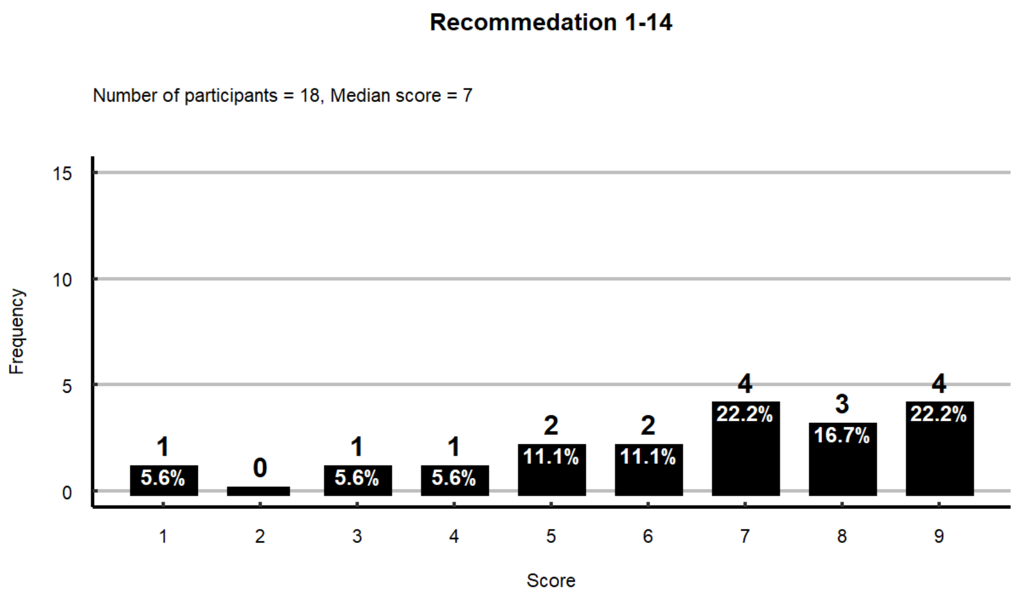

Comments of voting panelists

1. Lack of in vivo studies.

2. Scarce evidence.

3. During high-frequency oscillatory ventilation, only one in vitro study suggested better drug delivery when nebulizers (vibrating mesh and jet nebulizers) are placed between the Y-piece and the endotracheal tube.

4. Agree but no clinical/animal datas.

5. Only one study and HFOV is not used clinically for adult patients.

6. See previous comments about HFOV and aerosol delivery.

7. Few in vitro studies available – with major concerns related to the comparison between groups.

8. The recommendation should include the nebulization of bronchodilators and antibiotics (outlined in yellow). There is very little evidence for this recommendation (a single in vitro study). In addition, HFO is now used exclusively in neonates with acute respiratory failure. Since the publication of the OSCILLATE trial showing an excess in mortality in ARDS patients treated by HFO (see Ferguson et al OSCILLATE trial NEJM 2013), HFO is not used anymore in adult critically ill patients. I suggest

either to remove this recommendation or to clearly indicate that HFO is not anymore used in adults with ARDS.

9. Should be combined with 1.2 and 1.5.

Round 2

**Recommendations 1.14**	During high-frequency oscillatory ventilation, nebulizers are recommended to be placed between the Y-piece and the endotracheal tube.
**Likert score of 1-9**	□1 □2 □3 □4 □5 □6 □7 □8 □9<br>1= absolutely disagree, 9 = absolutely agree
**Comments**	

Section 1: Aerosol Delivery via Invasive Ventilation for Adult Patients

Recommendation 1.15： The efficiency of aerosol delivery in dry ventilator circuits is higher than that in humidified ventilator circuits. Considering the potential harms of dry gas on patient airway, turning off humidifier is not recommended for routine aerosol therapy.

Distribution of voting scores

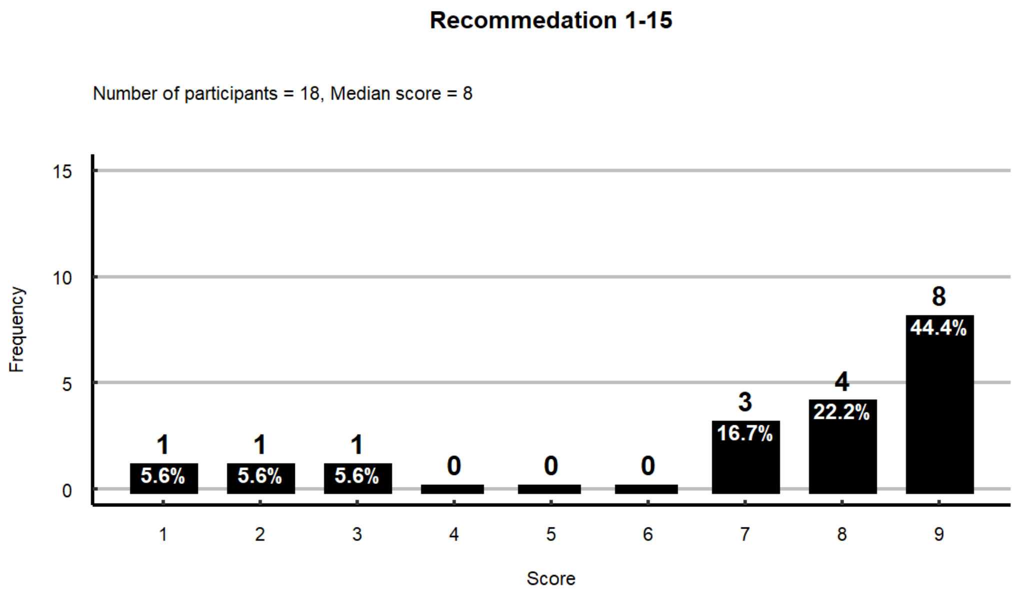

Comments of voting panelists

1. Humidifiers must be removed before antibiotic administration.

2. What about short term duration of administration?

3. Clinical studies showed that humidification does not influence bioavailability and clinical outcomes with any of the devices tested.

4. Not completely agree. The efficiency of aerosol delivery in dry ventilator circuits is similar to humidified ventilator circuits for in vivo studies. But I absolutely agree that “. Considering the potential harms of dry gas on patient airway, turning off humidifier is not recommended for routine aerosol therapy”.

5. Most In Vitro study showed that aerosol delivery was higher with dry ventilator circuits. However, 2 of the 3 in vivo studies showed no difference in aerosol delivery between humidified and non-humidified ventilator circuits. Lin et al (reference 9) showed that turning off the heated humidifier did not increase drug delivery. These results suggests that the transition from a dry circuit to a humidified circuit, or vice versa, takes time, Therefore, in vivo, aerosol delivery may not be increased if aerosol

is administered immediately or within 1 hour of turning off the humidifier.
However, the administration of bronchodilators must be distinguished from that of inhaled antibiotics. The later need distal lung deposition to treat infected parenchyma. As shown in the reference 6 (Miller et al), the antibiotic level deposited in sputum was significantly decreased with humidification that could reduce treatment efficacy.
Therefore, this recommendation could be relevant for the administration of bronchodilators, but not for inhaled antibiotics. This issue needs to be discussed.
6. Much potential for harm with dry circuit.
7. Data suggest minor effect of HHWH on aerosol delivery with jet nebulizers or mesh – thus we do not recommend switching off the HHWH during aerosol delivery – However data suggest less drug deposition with HHWH without bias flow – and no need to switch off the HHWH with bias flow – again more data are in any case needed about the interaction with bias flow (active in almost all ventilator now) and different type of ventilation and settings.
8. I suggest to improve (and modify) the formulation of this recommendation. Aerosol delivery of bronchodilators and antibiotics should be differentiated. I suggest to split recommendation 1.15 into two recommendations.

- The first recommendation could concern the aerosol delivery of bronchodilators, specifying the reasons why turning off the humidifyer is not recommended (see above corrections outlined in yellow). I would fully agree with the recommendation as newly formulated “9 = absolutely agree”
- A second recommendation could be added and formulated as “The efficiency of aerosol delivery of antibiotics in dry ventilator circuits is higher than that in humidified ventilator circuits. Considering 1) that the infected lung parenchyma is the target organ, 2) that the decreased efficiency of “dry” aerosol compromise lung deposition of aerosolized particles and 3) the potential harms of dry gas on patient airway is limited if the nebulization period does not exceed 30 minutes, turning off humidifier is recommended for aerosol delivery of antibiotics.” This recommendation is based on recommendations coming from the European Society of infectious disease (see Rello J, Rouby JJ, Sole-Lleonart C, Chastre J, Blot S, Luyt CE, Riera J, Vos MC, Monsel A, Dhanani J, Roberts JA. Key considerations on nebulization of antimicrobial agents to mechanically ventilated patients. Clin Microbiol Infect. Sep;23(9):640-646, 2017) and from the European Research Network for Nebulized Antibiotics in Ventilator-Associated Pneumonia (ENAVAP).

9. The efficiency is higher in in vitro studies but not in vivo. Please modify the recommendation to reflect this.

Round 2

**Recommendations 1.15**	The efficiency of aerosol delivery in dry ventilator circuits is higher than that in humidified ventilator circuits. Considering the potential harms of dry gas on patient airway, turning off humidifier is not recommended for routine aerosol therapy.
**Likert score of 1-9**	□1 □2 □3 □4 □5 □6 □7 □8 □9<br>1= absolutely disagree, 9 = absolutely agree
**Comments**	

Additional reference：

1. Yang SH, Yang TM, Lin HL, Tsai YH, Fang TP, Wan GH. Size distribution of salbutamol/ipratropium aerosols produced by different nebulizers in the absence and presence of heat and humidification. Pulm Pharmacol Ther. 2018; 48:22-27. doi: 10.1016/j.pupt.2017.10.009.

Section 1: Aerosol Delivery via Invasive Ventilation for Adult Patients

Recommendation 1.16： When aerosol device is placed in the inspiratory limb, removing or bypassing the heat moisture exchanger is recommended.

Distribution of voting scores

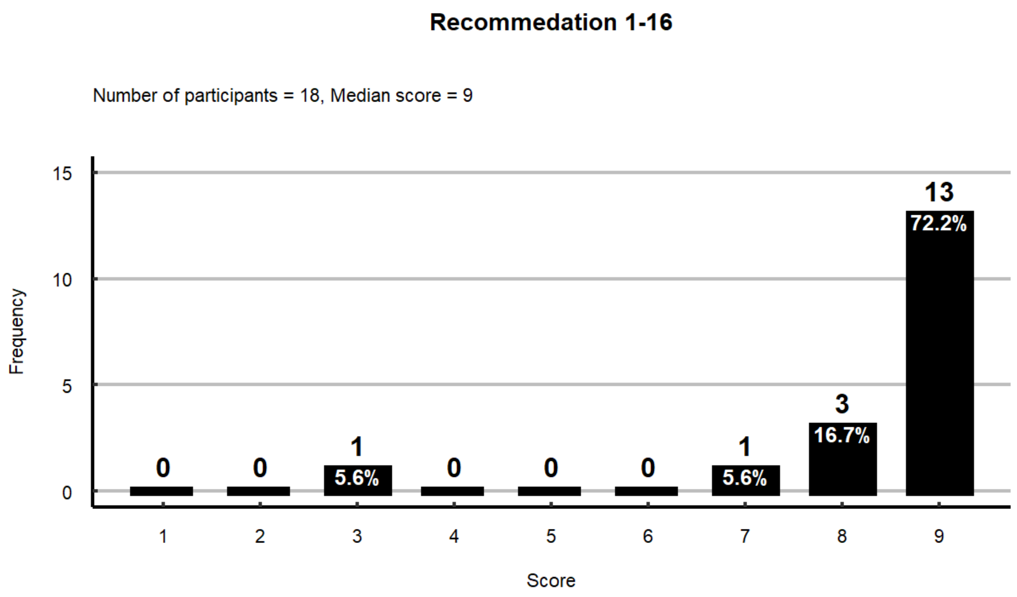

Comments of voting panelists

1. Accidents can occur.

2. Heat moisture exchanger is a physical barrier and should not be placed between the aerosol device and the patient.

3. As indicated in the results of the study (reference 1): Drug delivery without exhaled humidity exceeded aerosol deposition obtained with exhaled humidity in all conditions tested in this study. As a result, in this table, the real control condition NO HME is "without exhaled humidity", so I think that the dose delivered in the control condition is 18% instead of 10.8%. Therefore, both studies support strongly this recommendation.

4. Generally, agree. However, I believe that there might be an HME commercially available that can be used inline with aerosol.

5. I suggest to add that the recommendation concerns both bronchodilators and antibiotics.

6. This is logical but further investigation is needed.

Round 2

**Recommendations 1.16**	When aerosol device is placed in the inspiratory limb, removing or bypassing the heat moisture exchanger is recommended.
**Likert score of 1-9**	□1 □2 □3 □4 □5 □6 □7 □8 □9<br>1= absolutely disagree, 9 = absolutely agree
**Comments**	

Section 1: Aerosol Delivery via Invasive Ventilation for Adult Patients

Recommendation 1.17： Ventilator integrated breath-actuated jet nebulizer is not preferred over continuous jet nebulizer.

Distribution of voting scores

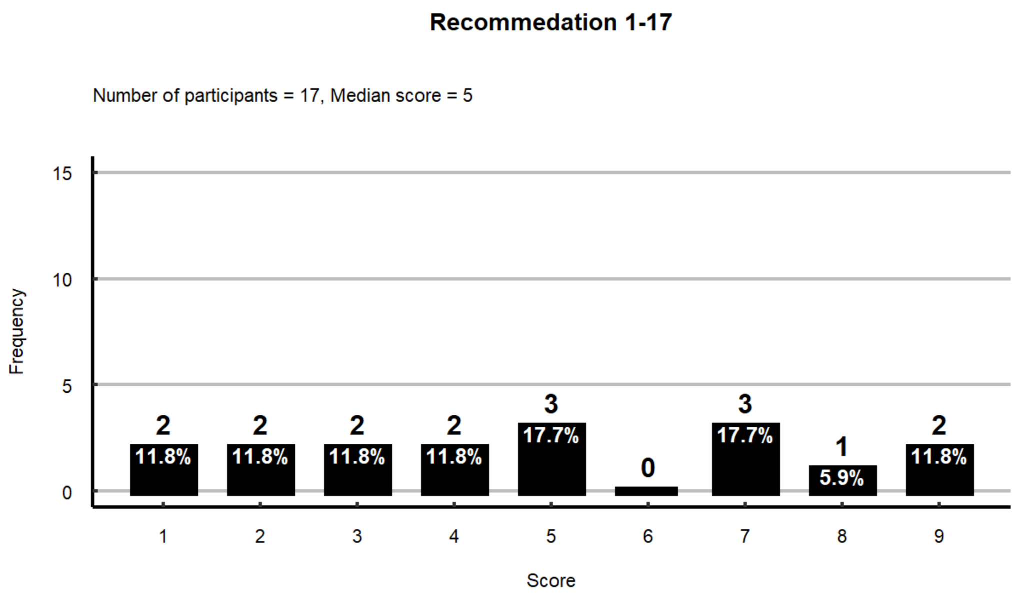

Comments of voting panelists

1. Confirmed in clinical practice.

2. Limited in vitro data showed a trend in favor of ventilator integrated breath-actuated jet nebulizer. However, its aerosol delivery may appear poor, due to persistent nebulization during the inspiratory pause and expiration, in part because of gas compression/decompression proximal to the nebulizer (Ehrmann S: Respiratory Care October 2014, 59 (10) 1508-1516)).

3. Ventilator integrated breath-actuated jet nebulizer increases drug delivery and reduce the risk of patient injuries by the air flow adjustment in comparison to continuous jet nebulizer.

4. This recommendation is too strong in the opposite direction from the results.

5. Two studies using different methods, different positions of the nebulizer obtained different results.

6. Not only because of the aerosol delivery efficiency, but also the staff time spent at the bedside for aerosol delivery.

7. Weak evidence. No human study.

8. At 15 cm from Y and before Y – syncro jet nebulizers might be less effective than

continuous – again it depends on the type of ventilation, presence or not of HHWH switch off or on – and bias flow.

9. It is important to differentiate aerosol delivery of bronchodilators where breath-actuated or continuous nebulization can be indifferently used, from aerosol delivery of antibiotics in ventilator-associated pneumonia where breath-actuated nebulization is unable to deliver high doses of antibiotics

10. Results of investigations are not conclusive and further clinical studies are needed.

11. Miller study states the opposite and Wan has similar inhaled doses.

Round 2

**Recommendations 1.17**	Ventilator integrated breath-actuated jet nebulizer is not preferred over continuous jet nebulizer.
**Likert score of 1-9**	□1 □2 □3 □4 □5 □6 □7 □8 □9<br>1= absolutely disagree, 9 = absolutely agree
**Comments**	

Section 1: Aerosol Delivery via Invasive Ventilation for Adult Patients

Recommendation 1.18：The influence of ventilator integrated breath-actuated jet nebulizer on ventilator function and aerosol delivery efficiency varies between ventilators. Confirming the aerosol delivery performance for available ventilator before delivering aerosol is recommended.

Distribution of voting scores

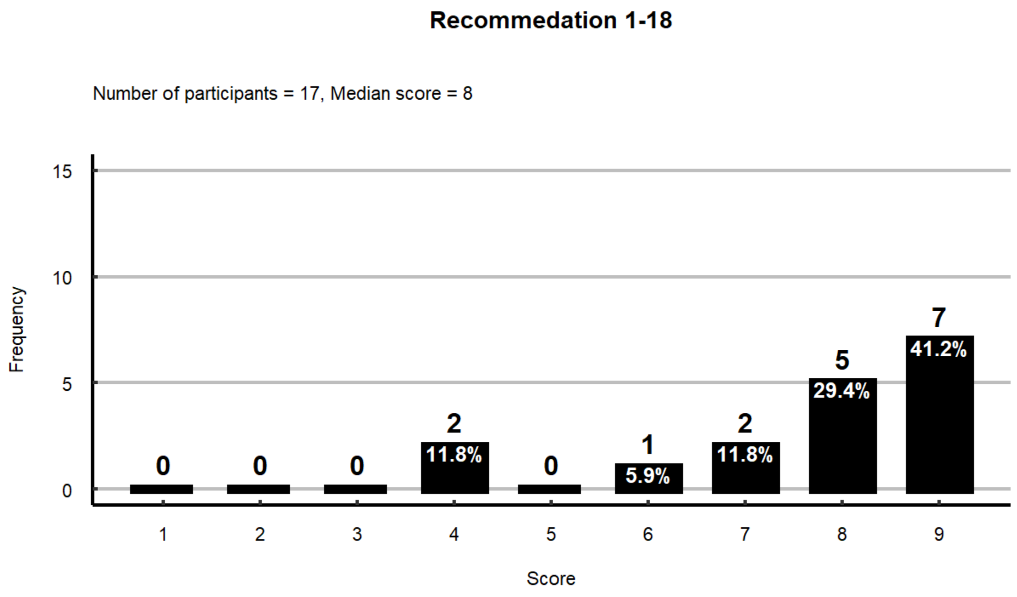

Comments of voting panelists

1. It is a logical recommendation but very low evidence

2. I don't understand what is meant by "Confirming the aerosol delivery performance before delivering aerosol is recommended". How should clinicians do that?

3. Evidence is weak. No human data.

4. This is a very important issue – the effects depend on different variables – more difficult to be kept constant in vivo.

Round 2

**Recommendations 1.18**	The influence of ventilator integrated breath-actuated jet nebulizer on ventilator function and aerosol delivery efficiency varies between ventilators. Confirming the aerosol delivery performance for available ventilator before delivering aerosol is recommended.

**Likert score of 1-9**	□1 □2 □3 □4 □5 □6 □7 □8 □9<br>1= absolutely disagree, 9 = absolutely agree
**Comments**	

Section 1: Aerosol Delivery via Invasive Ventilation for Adult Patients

Recommendation 1.19： Metered-dose inhaler should be primed, shaken, with actuation at the beginning of inspiration, with a minimum of 15 seconds between two puffs.

Distribution of voting scores

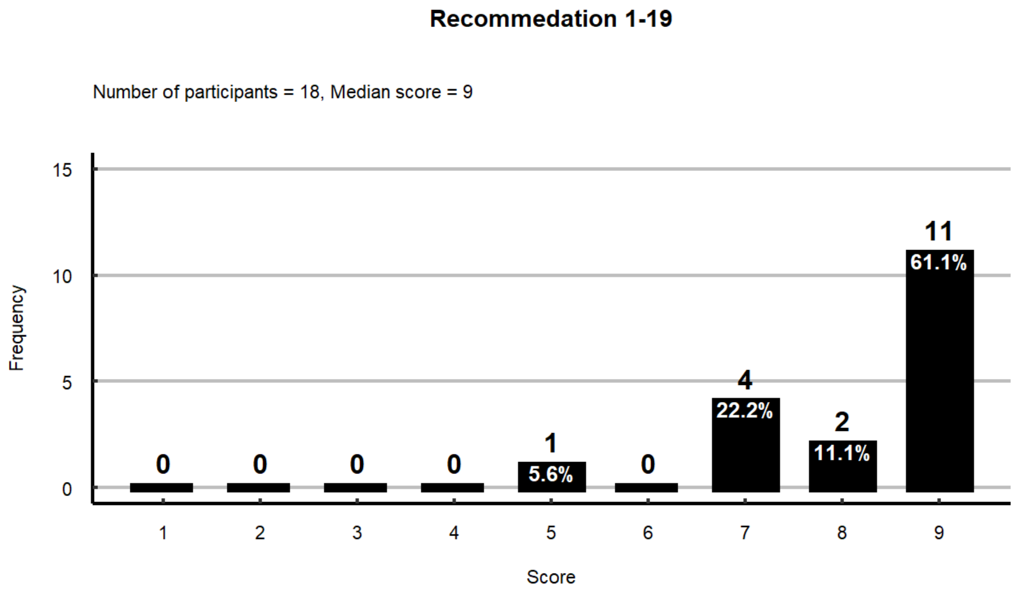

Comments of voting panelists

1. Lack of in vivo studies, Low evidence.

2. Few clinical evidence.

3. Evidence weak. No human study. Naughton is review, not a study.

4. Evidence might also suggest the inhalation after expiration – not many data supporting the statement - also breath slowly – differences during mechanical ventilation? where it is also recommended to start at expiration – fill the spacer and then make a full inspiration by ventilator.

5. See References in Recommendation 1.12.

Round 2

**Recommendations 1.19**	Metered-dose inhaler should be primed, shaken, with actuation at the beginning of inspiration, with a minimum of 15 seconds between two puffs.
**Likert score of 1-9**	□1 □2 □3 □4 □5 □6 □7 □8 □9

	1= absolutely disagree, 9 = absolutely agree
**Comments**	

Section 1: Aerosol Delivery via Invasive Ventilation for Adult Patients

Recommendation 1.20.1： Increasing fill volume in jet or ultrasonic nebulizer to improve aerosol delivery efficiency is not recommended.

Distribution of voting scores

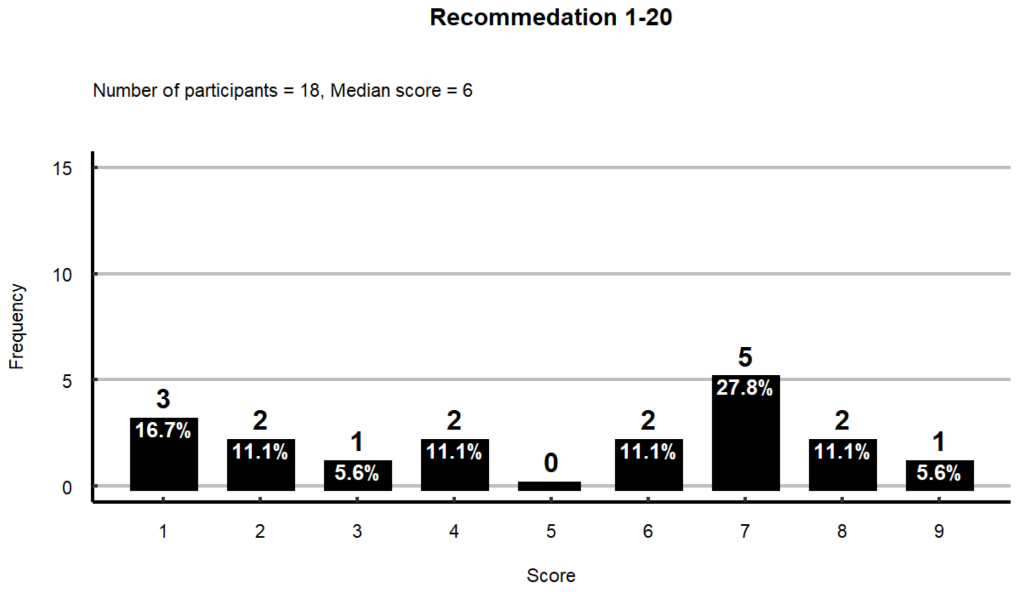

Comments of voting panelists

1. Absence of clinical evidence.

2. In vitro data suggested better drug delivery when increasing fill volume in jet or ultrasonic nebulizers.

3. 3mL was systematically worse than 6mL… It is difficult to recommend to not increase the fill volume.

4. Please clarify the meaning of “fill volume” in the recommendation. In these 2 references, the drug concentration remained unchanged while the “fill volume” was increased. In this case, increasing the volume of fill means increasing the dosage of the drug. Sometimes, the filling volume can be also increased without changing the dosage, in other words, the dilution is increased. Please specify.

Furthermore, both studies showed that when increasing the “fill volume” induced an increased in inhaled dose. Based on theses 2 studies, I do not understand why you state that “increasing fill volume in jet or ultrasonic nebulizer to improve aerosol delivery efficiency is not recommended” whereas it can increase aerosol delivery.

5. For FDA approved drug, such as bronchodilator, the fill volume and dose are prepared in the package, it is unnecessary to dilute. But for off-label drug, such as

inhaled antibiotics, if increasing the fill volume can increase the aerosol deposition, it might be considered. Moreover, the staff time spent on aerosol therapy (cost-effectiveness) should also be considered.

6. Drugs for aerosol include label concentration. Adding diluent is a change from label that might have an effect, based on drug.

7. Aerosol delivery efficiency of jet or ultrasonic nebulizer depends on the residual dose. Thus we must consider discrepancy of residual volume among each brand.

8. Evidence weak. No human data.

9. Data suggest that higher volume might be associated with increased delivery – at least from in vitro studies.

10. The statement is true for US but not for jet N.

Round 2

**Recommendations 1.20.1**	~~Increasing fill volume in jet or ultrasonic nebulizer to improve aerosol delivery efficiency is not recommended.~~ **For the jet or ultrasonic nebulizer with a moderate residual volume, aerosol delivery efficiency is higher with a higher fill volume, but changing fill volume for the sole purpose of improving aerosol delivery efficiency is not recommended.**
**Likert score of 1-9**	□1 □2 □3 □4 □5 □6 □7 □8 □9<br>1= absolutely disagree, 9 = absolutely agree
**Comments**	

[Additional recommendation based on reviewer suggestion]

**Recommendations 1.20.2**	Increasing diluent volume in vibrating mesh nebulizer to improve aerosol delivery efficiency is not recommended.
**Evidence**	In vivo[1], in vitro[1-3]
	If you have additional evidence, please provide references:
**Likert score of 1-9**	□1 □2 □3 □4 □5 □6 □7 □8 □9<br>1= absolutely disagree, 9 = absolutely agree
**Comments**	

Section 1: Aerosol Delivery via Invasive Ventilation for Adult Patients

Recommendation 1.21： Aerosol delivery efficiency varies between endotracheal tube and tracheotomy tube. Changing tubes for the sole purpose of improving aerosol delivery efficiency is not recommended.

Distribution of voting scores

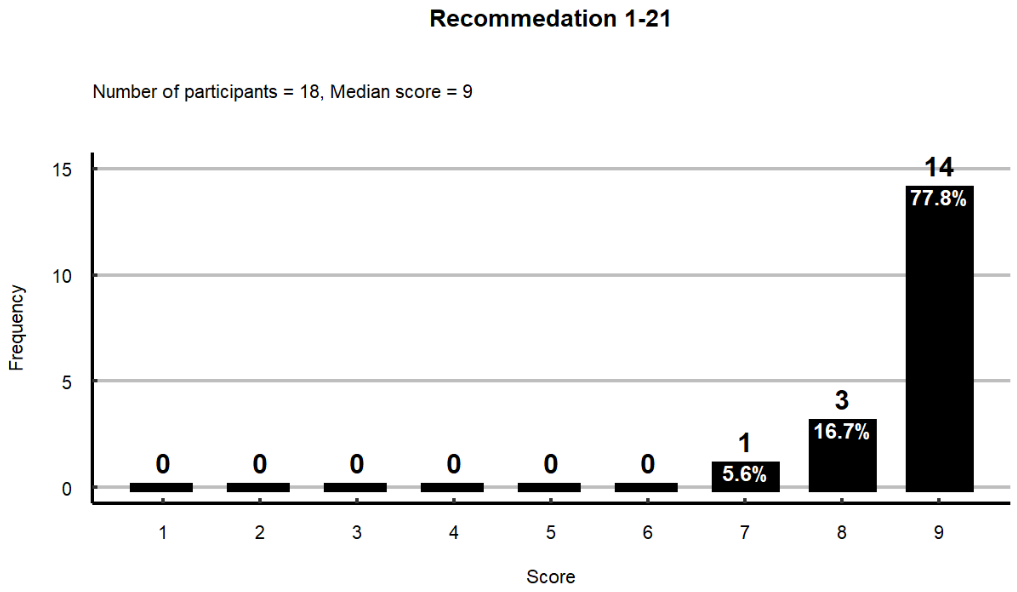

Comments of voting panelists

1. Risk benefit abosutely in favor of no removing tubes.

2. In vitro studies showed that the type of tube does not influence inhaled dose in mechanical ventilation.

3. Only one in vitro study (reference 1, Ari et al) showed a higher inhaled dose with the tracheostomy than with the endotracheal tube. The other 3 studies found no differences. In the recommendation, I would delete the first sentence "Aerosol delivery efficiency varies between the endotracheal tube and the tracheostomy tube“.

4. Harm outweighs benefit, but evidence weak.

5. The second line, although I agree, is not supported by the studies.

Round 2

**Recommendations 1.21**	Aerosol delivery efficiency varies between endotracheal tube and tracheotomy tube. Changing tubes for the sole purpose of improving aerosol delivery efficiency is not

	recommended.
**Likert score of 1-9**	□1 □2 □3 □4 □5 □6 □7 □8 □9<br>1= absolutely disagree, 9 = absolutely agree
**Comments**	

Section 1: Aerosol Delivery via Invasive Ventilation for Adult Patients

Recommendation 1.22： Aerosol delivery efficiency is higher with a large size of endotracheal tube, but changing endotracheal tube for the sole purpose of improving aerosol delivery efficiency is not recommended.

Distribution of voting scores

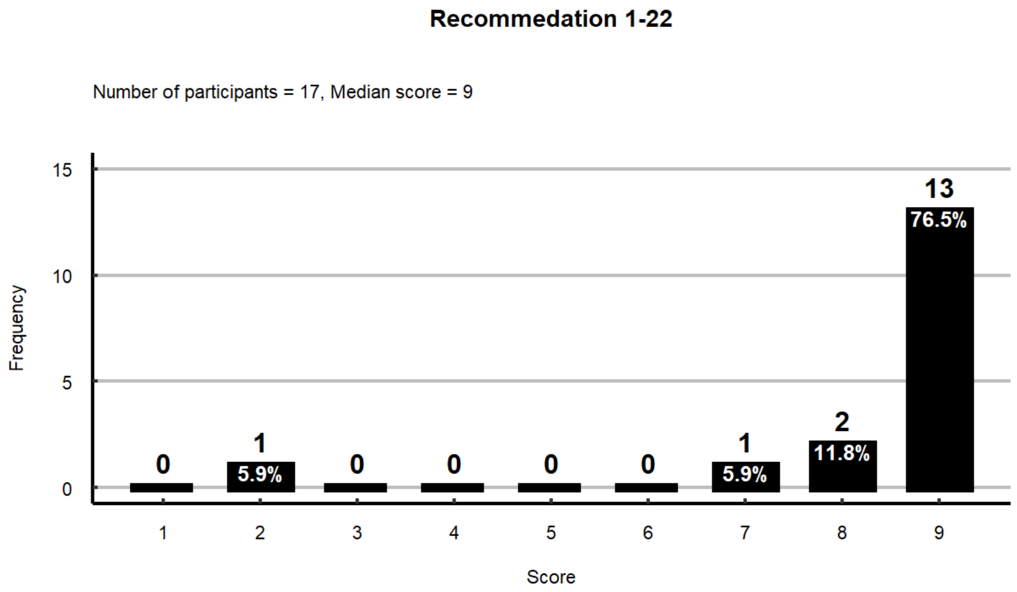

Comments of voting panelists

1. Absolutely logical.

2. Potential harm outweighs benefit, but evidence is weak.

3. I agree with the statement that the tube should not be changed just to increase aerosol delivery. I had written a paper on the effect of the ET tube size on aerosol delivery several years ago. Dhand R Respir Care 2000 Jun;45(6):636. After a certain size (7mm ID), the size of the tube may not be a crucial factor.

Round 2

**Recommendations 1.22**	Aerosol delivery efficiency is higher with a large size of endotracheal tube, but changing endotracheal tube for the sole purpose of improving aerosol delivery efficiency is not recommended.
**Likert score of 1-9**	□1 □2 □3 □4 □5 □6 □7 □8 □9<br>1= absolutely disagree, 9 = absolutely agree

**Comments**	

Additional reference:

1. Taylor RH, Lerman J. High-efficiency delivery of salbutamol with a metered-dose inhaler in narrow tracheal tubes and catheters. Anesthesiology 1991;74(2):360–363

2. O'riordan, Thomas, G, et al. Nebulizer Function during Mechanical Ventilation[J]. Am Rev Respir Dis, 1992;145(5):1117-22

Section 1: Aerosol Delivery via Invasive Ventilation for Adult Patients

Recommendation 1.23： When heliox is utilized for invasive ventilation, aerosol delivery efficiency can improve. However, adding heliox for the sole purpose of improving aerosol delivery efficiency is not recommended.

Distribution of voting scores

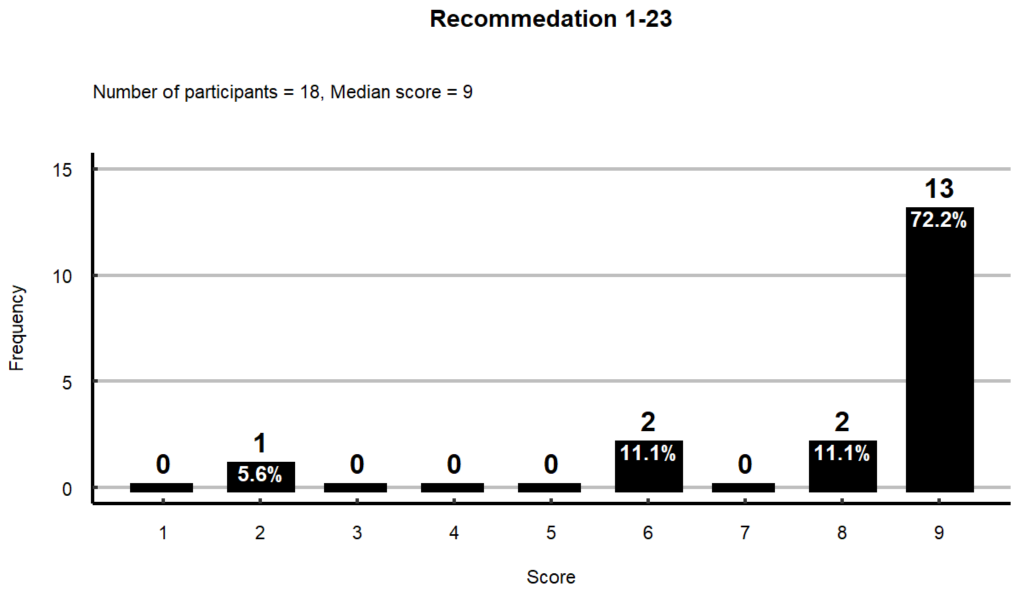

Comments of voting panelists

1. Totally logical.

2. The use of Heliox with ventilators is complicated by technical difficulties and the availability (cost). Some mechanical ventilators work poorly (e.g alters tidal volume, alarms, and other parameters) with Heliox.

3. Cost-effectiveness.

4. Agree, but evidence is weak.

5. Not always available and costly.

6. Heliox improves aerosol delivery of bronchodilators in vitro and of antibiotics in animals with normal lungs. This benefit is lost in animals with infected lungs (see M. Tonnellier, F. Ferrari, I. Goldstein, A. Sartorius, C.-H. Marquette, J. J. Rouby. Intravenous versus Nebulized Ceftazidime in Ventilated Piglets with and without Experimental Bronchopneumonia. Comparative effects of Helium and Nitrogen. ANESTHESIOLOGY 102, 995-1000, 2005). I suggest to modify the formulation of the recommendation as outlined in yellow.

7. Should specify pMDI + spacer since it was the only device tested.

Round 2

**Recommendations 1.23**	When heliox is utilized for invasive ventilation, aerosol delivery efficiency can improve. However, adding heliox for the sole purpose of improving aerosol delivery efficiency is not recommended.
**Likert score of 1-9**	□1 □2 □3 □4 □5 □6 □7 □8 □9<br>1= absolutely disagree, 9 = absolutely agree
**Comments**	

Additional reference:

1. Tonnellier M, Ferrari F, Goldstein I, Sartorius A, Marquette CH, Rouby JJ. Intravenous versus nebulized ceftazidime in ventilated piglets with and without experimental bronchopneumonia: comparative effects of helium and nitrogen. Anesthesiology. 2005 May;102(5):995-1000

Section 1: Aerosol Delivery via Invasive Ventilation for Adult Patients

Recommendation 1.24： When heliox is substituted for oxygen to drive continuous jet nebulizer at the same driving flow, nebulizer output is reduced. If driving nebulizer with heliox, it is recommended to set at 15 L/min.

Distribution of voting scores

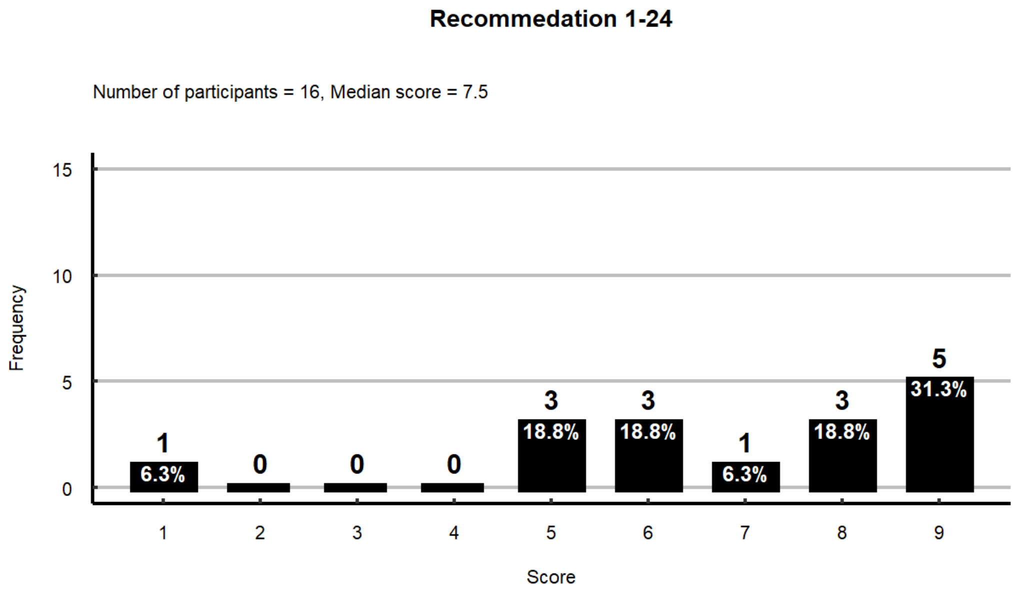

Comments of voting panelists

1. It is based in a single study.
2. Limited data showed a higher drug delivery with a driving flow set at15 L/min.
3. There is a risk in terms of ventilation parameters modification, modifying the gas volume delivery to the patient.
4. There is no evidence to support the use of heliox in patients. Thus I would not make any recommendation on this.
5. If heliox is not recommended, I would remove this recommendation which is based on only one in vitro study.
6. Weak evidence. Not studied in humans.
7. Minor evidence – only few studiesin vitro.
8. Thee recommendation is not supported by the paper. Why 15? That delivers twice as much as 10 L/min of O2.

Round 2

**Recommendations 1.24**	When heliox is substituted for oxygen to drive continuous jet nebulizer at the same driving flow, nebulizer output is reduced. If driving nebulizer with heliox, it is recommended to set at 15 L/min.
**Likert score of 1-9**	□1 □2 □3 □4 □5 □6 □7 □8 □9<br>1= absolutely disagree, 9 = absolutely agree
**Comments**	

Section 1: Aerosol Delivery via Invasive Ventilation for Adult Patients

Recommendation 1.25.1：Spontaneous breathing may help increase aerosol delivery efficiency, but it is not recommended to change the ventilator mode for the sole purpose of improving aerosol delivery efficiency.

Distribution of voting scores

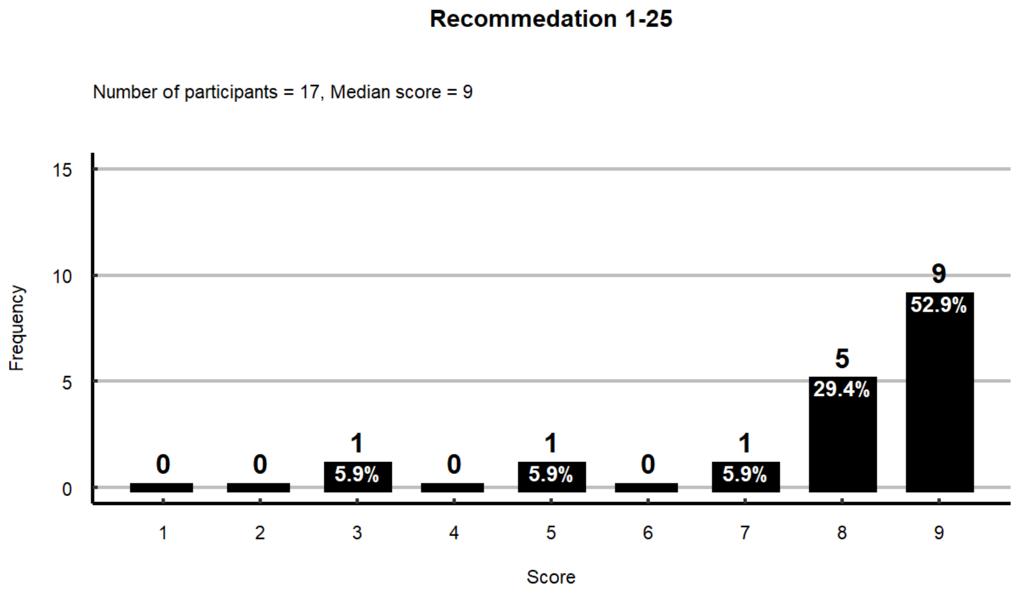

Comments of voting panelists

1. No Logical recommendation.

2. There is no evidence that spontaneous breathing increases aerosol delivery efficiency during invasive ventilation.

3. I disagree spontaneous breathing improves delivery efficiency. Again it depends on the drug delivered if there is a interest in changing the ventilator settings.

4. It is extremely difficult to make a recommendation based on these references, as each reference used a different method and studied a different objective. For example, reference 1 (Fink et al) studied aerosol delivery by MDI. Using this device, spontaneous breathing may help increase the efficiency aerosol delivery. However, reference 3 (Dugernier et al) compared 2 ventilatory mode (volume control and pressure support with spontaneous ventilation) using VMN. They found lower lung deposition with the pressure support mode because of the increased inspiratory flow with this mode. This result doesn't support the sentence above in the recommendantion "Spontaneous breathing may help increase aerosol delivery efficiency". Finally, in reference 4 (Ge et al), the authors studied another ventilatory APRV mode and the increase in inhaled

mass with APRVs, according to the authors, was explained by the increase in minute ventilation.

It should be noted that in the in vivo study of Dugernier et al (reference 3), the authors found a higher endotracheal tube and tracheal deposition with pressure assisted ventilation (extrapulmonary deposition). This result cannot be observed in the in vitro study because the endotracheal tube and tracheal deposition included in the inhaled mass but it was not considered as extrapulmonary deposition.

5. Potential harm outweighs benefit.

6. Spontaneous breathing may increase aerosol delivery of bronchodilators (the target organ is the bronchial tree) but markedly reduces lung deposition of nebulized antibiotics (the target organ is the infected lung parenchyma). I suggest to include this information in the recommendation (outlined in yellow).

7. Settings may need to be changed if the patient is not synchronizing with the ventilator.

Round 2

**Recommendations 1.25.1**	Spontaneous breathing may help increase aerosol delivery efficiency, but it is not recommended to change the ventilator mode for the sole purpose of improving aerosol delivery efficiency.
**Likert score of 1-9**	□1 □2 □3 □4 □5 □6 □7 □8 □9<br>1= absolutely disagree, 9 = absolutely agree
**Comments**	

[Additional recommendation based on reviewer suggestion]

**Recommendations 1.25.2**	**When delivering inhaled antibiotics for invasively ventilated patients, spontaneous breathing ventilator modes reduce aerosol delivery efficiency, thus spontaneous breathing should be prohibited and volume controlled mode is recommended during antibiotics nebulization.**
**Likert score of 1-9**	□1 □2 □3 □4 □5 □6 □7 □8 □9<br>1= absolutely disagree, 9 = absolutely agree
**Comments**	

Table1.25.1 Studies comparing of different mode and with/without spontaneous breathing

Author, year	Study Type	Population	Inhaled medication	Nebulizer And Position	Mode and Finding/Aerosol deposition					
Fink, 1996[1]	In vitro	Adult	Albuterol	MDI+Spacer	(non-humidification, RR 10/min, peak inspiratory flow 40 L/min, square-wave flow, trigger -2 cmH_2O	**CMV, VT 800ml**	**A/C, VT 800ml**	**PS 20, VT 800ml**	**CPAP, VT 800ml**	**P**
						30.3 ± 3.4%	31.9±1.3%	30.9 ± 1.8%	39.2 ± 1.4%	<0.01
Malliotakis, 2007[2]	In vivo	Adult: 10 COPD patients	Salbutamol	MDI+Spacer, inspiratory limb	Tube diameter 8.0±0.5 mm, tube length 28±1 mm		**VC***	**PS#**		**P**
						Baseline	Rrs: 26.5±4.1<br>Rint:18.4± 4<br>PEEPi:8.4±3.2	Rrs:25.8±4.8<br>Rint:18.1±4.3<br>PEEPi:8±2.7		>0.05
						15min	Rrs:23.7±3.7<br>Rint: 15.9±3.5<br>PEEPi:7.2±3.5	Rrs:23±4<br>Rint:15.3±3.5<br>PEEPi:6.8±2.9		
						30min	Rrs:23.1±3.6<br>Rint:15.5±3.6<br>PEEPi:7.1±3.5	Rrs:22.4±4<br>Rint:14.9±3.7<br>PEEPi:6.9±3.2		
						60min	Rrs:23.3±4.3<br>Rint:15.1±3.8<br>PEEPi:7.1±3.5	Rrs:22.6±4<br>Rint:14.8±4<br>PEEPi:6.8±3.1		
						120min	Rrs:23.9±4.4<br>Rint:15.5±4<br>PEEPi:7.4±3.6	Rrs:23.1±3.6<br>Rint:15.6±4.1<br>PEEPi:7±3		
Dugernier J, 2016[3]	In vivo	Adult: 17 postoperative neurosurgery patients	Diethylenetriaminepentaacetic acid labeled with technetium-99m	Vibrating mesh nebulizer, between the endotracheal tube and the catheter mount	PEEP 5 cmH_2O, base flow 10 L/min, no end-inspiratory pause, VT 8 ml/kg	**PS&**		**VC^**		**P**
						10.5 ± 3.0%		15.1 ± 5.0 %		0.038

						PCV BF0	PCVBF6	APRV	APRVs	P
Ge, 2019[4]	In vitro	Adult	Albuterol	Vibrating mesh nebulizer	Position 1: inspiratory limb at Y	15.9%	20.8%	9.5%	23.1%	<0.001
					Position 2: Humidifier outlet	19.4%	21.1%	17.9%	27.4%	
					Position 3: Humidifier inlet	29.8%	23.6%	23.1%	34.1%	

**VC：volume control, VT 7~8 ml/kg, square wave flow, no end-inspiratory pause, zero PEEP, F_IO_2 achieved SpO_2＞89%；*

#PS：pressure support, pressure-support level (obtain a comparable VT), extrinsic PEEP set as 1~2 cm H_2O lower than PEEPi (measured on controlled mode), triggering 2 L/min.

&PS: pressure support ventilation, expiratory trigger was set to obtain an inspiratory/expiratory ratio of 30 %

^VC: volume-controlled ventilation, VE 8 L/min, inspiratory time and respiratory rate were then adjusted to ensure inspiratory/ expiratory ratio of 30 %, constant inspiratory flow 30 L/min.

PCV_{BF0}: pressure control ventilation with no bias flow.

PCV_{BF6}: PCV with bias flow of 6 L/min.

APRV: Airway pressure release ventilation (with no spontaneous breaths),

APRVs: APRV with spontaneous breath trigger.

MDI, metered-dose inhaler; VT, tidal volume; CMV, controlled mechanical ventilation; A/C, assist/control; PS: pressure support; CPAP, continuous positive airway pressure; COPD, chronic obstructive pulmonary disease; VC, volume control; PEEP: positive end-expiratory pressure (cmH_2O); PEEPi, intrinsic positive end-expiratory pressure (cmH_2O); Rrs and Rint, maximum and minimum inspiratory resistance, respectively, (cmH_2O/l/s).

1. Fink JB, Dhand R, Duarte AG, Jenne JW, Tobin MJ. Aerosol delivery from a metered-dose inhaler during mechanical ventilation. An in vitro model. Am J Respir Crit Care Med. 1996 Aug;154(2 Pt 1):382~7.
2. Malliotakis P, Mouloudi E, Prinianakis G, Kondili E, Georgopoulos D. Influence of respiratory efforts on b2-agonist induced bronchodilation in mechanically ventilated COPD patients: a prospective clinical study. Respir Med. 2007 Feb;101(2):300-7.
3. Dugernier J, Reychler G, Wittebole X, Roeseler J, Depoortere V, Sottiaux T, Michotte JB, Vanbever R, Dugernier T, Goffette P, Docquier MA, Raftopoulos C, Hantson P, Jamar F, Laterre PF, (2016) Aerosol delivery with two ventilation modes during mechanical ventilation: a randomized study. Ann Intensive Care 6: 73
4. Ge HQ, Wang JM, Lin HL, Fink JB, Luo R, Xu P, Ying K. Effect of Nebulizer Location and Spontaneous Breathing on Aerosol Delivery During Airway Pressure Release Ventilation in Bench Testing. J Aerosol Med Pulm Drug Deliv. 2019 Feb;32(1):34-39.

1.25.2 Recommendations for nebulized antibiotics in the consensuses and reviews.

Author, year	Nebulizer	Position	Mode	Vt	Inspiratory flow	I: E	RR	Bias flow	End-inspiratory pause	PEEP	Others
Rello J,2017			**Volume-controlled**	8ml/kg	Constant inspiratory flow	≤ 50%			20%	5-10 cmH_2O	• Avoid sharp angles and rough inner surfaces in circuit<br>• Avoid asynchronies and triggering<br>• Increase level of sedation if necessary<br>• Remove HME<br>• Turn off HH<br>• Change expiratory filter after each nebulization
Rouby JJ, 2020	VMN	10–15 cm before the Y piece on the inspiratory limb	**Volume-controlled**	8ml/kg	Limiting inspiratory flow velocity	1: 2	12-15 bpm		20%	5-10 cmH_2O	• Remove HME<br>• Turn off HH
Monsel A, 2021	Continuous VMN	15 cm before the Y piece on the inspiratory limb	**Volume-controlled**	8ml/kg	Constant inspiratory flow	1: 1	12-15 bpm	Absence or minimum (2 l/min)	20%		• Smooth angles and inner surface tube<br>• Increase level of sedation if necessary<br>• Remove HME<br>• Turn off HH

VMN vibrating mesh nebulizer, PDDS pulmonary drug delivery system, JN jet nebulizer, UN ultrasonic nebulizer, MV minute ventilation, Vt tidal volume, I: E inspiratory to expiratory ratio, RR respiratory rate, PEEP positive end-expiration pressure, HME heat and moisture exchanger, HH heated humidifiers,

1. Rello J, Rouby JJ, Sole-Lleonart C, Chastre J, Blot S, Luyt CE, Riera J, Vos MC, Monsel A, Dhanani J, Roberts JA. Key considerations on nebulization of antimicrobial agents to mechanically ventilated patients. Clin Microbiol Infect. 2017 Sep;23(9):640-646.
2. Rouby JJ, Sole-Lleonart C, Rello J; European Investigators Network for Nebulized Antibiotics in Ventilator-associated Pneumonia. Ventilator-associated pneumonia caused by multidrug-resistant Gram-negative bacteria: understanding nebulization of aminoglycosides and colistin. Intensive Care Med. 2020 Apr;46(4):766-770. Rouby JJ, Sole-Lleonart C, Rello J; European Investigators Network for Nebulized Antibiotics in Ventilator-associated Pneumonia. Ventilator-associated pneumonia caused by multidrug-resistant Gram-negative bacteria: understanding nebulization of aminoglycosides and colistin. Intensive Care Med. 2020 Apr;46(4):766-770.

Monsel A, Torres A, Zhu Y, Pugin J, Rello J, Rouby JJ; European Investigators Network for Nebulized Antibiotics in Ventilator-associated Pneumonia (ENAVAP). Nebulized antibiotics for ventilator-associated pneumonia: methodological framework for future multicenter randomized controlled trials. Curr Opin Infect Dis. 2021 Apr 1;34(2):156-168.

Section 1: Aerosol Delivery via Invasive Ventilation for Adult Patients

Recommendation 1.26： When metered-dose inhaler is utilized during invasive mechanical ventilation, there is no recommendation on flow trigger vs pressure trigger solely for aerosol delivery.

Distribution of voting scores

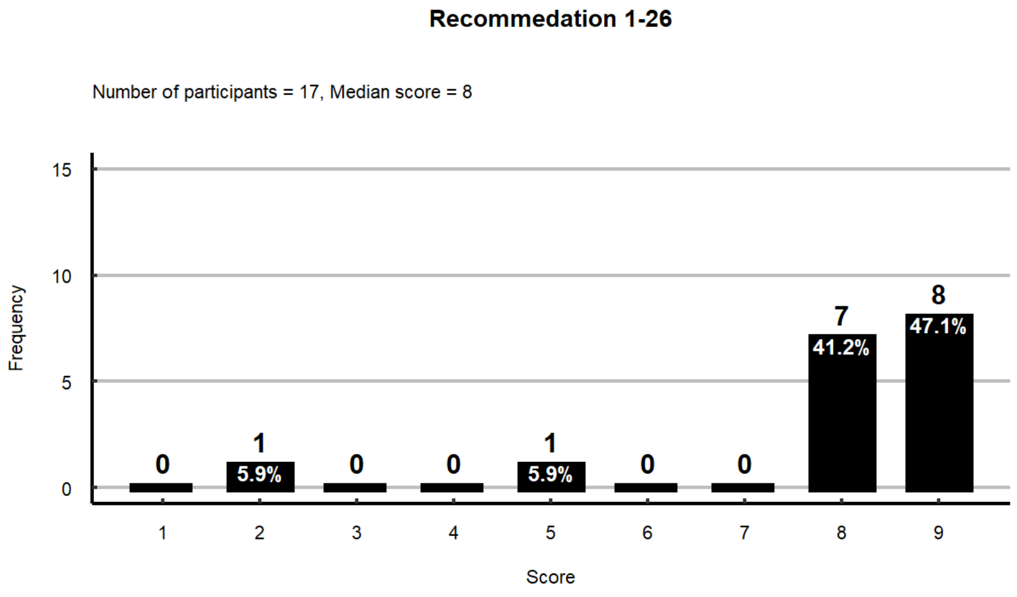

Comments of voting panelists

1. No evidence.

2. There are no more ventilators with pressure trigger nowadays. Even on ventilators displaying a pressure trigger on the screen it is a flow triggering system with a bias flow which is implemented. No recommendation on this.

3. Weak evidence. Not human data. Old study.

4. This might also be affected by the type of assisted ventilation and presence or not of bias flow.

Round 2

**Recommendations 1.26**	When metered-dose inhaler is utilized during invasive mechanical ventilation, there is no recommendation on flow trigger vs pressure trigger solely for aerosol delivery.
**Likert score of 1-9**	□1 □2 □3 □4 □5 □6 □7 □8 □9<br>1= absolutely disagree, 9 = absolutely agree

**Comments**	

Section 1: Aerosol Delivery via Invasive Ventilation for Adult Patients

Recommendation 1.27： It is not recommended to change ventilation parameters for the sole purpose of improving aerosol delivery efficiency.

Distribution of voting scores

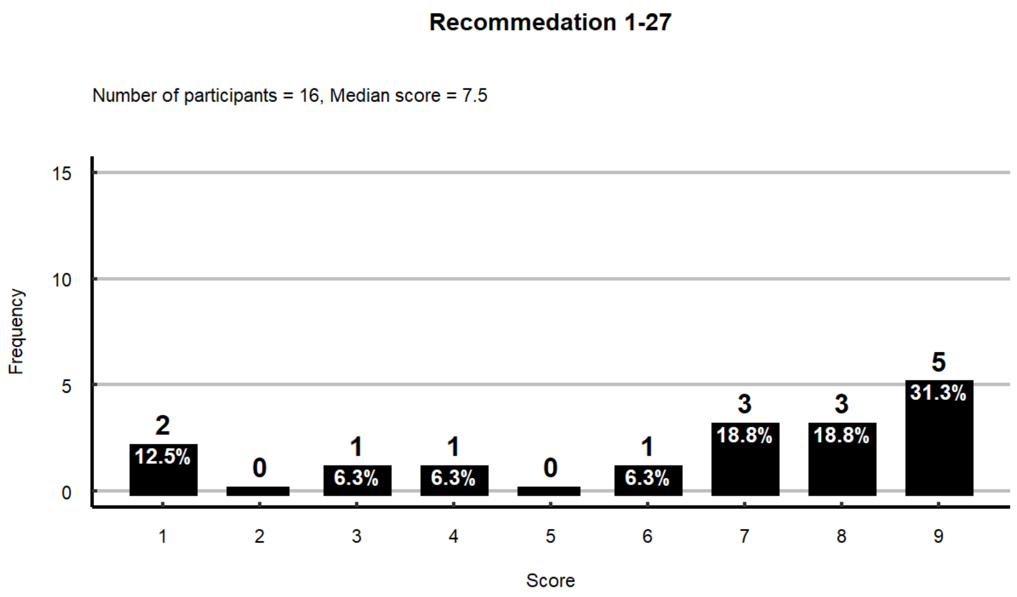

Comments of voting panelists

1. Ventilatory setting MUST be changed to increase efficiency in antibiotic aerosolization. Check ESCMID Position paper reported in CMI 2017

Specific ventilator settings should be used to decrease turbulences and proximal deposition of aerosol droplets:

– Volume-controlled mode,

– Low (6-8L/min) minute ventilation,

– Inspiratory flow kept below 40L/min,

– Inversed inspiratory to expiratory ratio of 50%

– Low (<12/min) respiratory frequency,

– Humidification system discontinued.

Applying these recommendations provides the possibility of delivering ~25% to 40% of the initial dose.

2. Definitely we need more studies in this point.

3. Changing ventilator settings may have adverse effects (e.g alterations of cardiac output, ventilator-induced lung injury, increasing PEEPi, increasing driving pressure …)

4. I think that based on the results, we can say that to change ventilation parameters

modify the delivery. I agree it is not recommend to change them but in some situation, when the nebulization is the main objective (more than the ventilation), the recommendation could be different…

5. In vitro studies have shown increased efficiency of aerosol delivery with low respiratory rate, low minute volume and high VT. Unfortunately, we have very few in vivo and/or clinical studies to confirm these in vitro findings. Because this recommendation is for all aerosol therapies, not just for bronchodilators, and because some aerosoltherapies require a high distal lung concentration, I do not think it is appropriate to say here "it is not recommended..."

6. Potential harm outweighs benefit.

7. Inspiratory flow turbulences cause the impaction of aerosolized particles on ventilator circuits and tracheobronchial walls. Specific ventilator settings selected to decrease inspiratory flow turbulences reduce the impaction of aerosolized particles in ventilator circuits and bronchi and promote lung parenchymal deposition. If bronchodilation is the therapeutic target, then inspiratory flow turbulences resulting from spontaneous breathing are beneficial for the asthmatic patients. If bacterial killing in the infected lung parenchyma is the therapeutic target, then inspiratory flow turbulences resulting from spontaneous breathing, are deleterious for the patients with ventilator-associated pneumonia. Although there is no formal demonstration of the latter statement, the majority of experts and academic societies recommend to adopt specific ventilator settings during the nebulization of antibiotics to treat ventilator-associated pneumonia. As a consequence, I suggest to follow these recommendations based on the corresponding references (above) and to provide this information in the recommendation (outlined in yellow). I also suggest to recommend experimental studies based on lung microdialysis to demonstrate that adopting specific ventilator settings during the nebulization period increases lung deposition of aerosolized antibiotics.

8. May need to change if settings are clearly sub-optimal for aerosol delivery.

9. Please rephrase: Ventilation parameters affect aerosol delivery efficiency.However, …

Round 2

**Recommendations 1.27.1**	It is not recommended to change ~~ventilation parameters~~ tidal volume and respiratory rate for the sole purpose of improving aerosol delivery efficiency.
**Likert score of 1-9**	□1 □2 □3 □4 □5 □6 □7 □8 □9<br>1= absolutely disagree, 9 = absolutely agree
**Comments**	

[Additional recommendations based on reviewer suggestion]

**Recommendations 1.27.2**	**When delivering inhaled antibiotics for invasively ventilated patients, it is recommended to set tidal volume of 8ml/kg of patient predicted body weight. .**
**Likert score of 1-9**	□1 □2 □3 □4 □5 □6 □7 □8 □9<br>1= absolutely disagree, 9 = absolutely agree
**Comments**	

**Recommendations 1.27.3**	When delivering inhaled antibiotics for invasively ventilated patients, it is recommended to keep respiratory rates at 12-15 breaths/min.
**Likert score of 1-9**	□1 □2 □3 □4 □5 □6 □7 □8 □9<br>1= absolutely disagree, 9 = absolutely agree
**Comments**	

Table1.27.1 Studies of change ventilation parameters

Author, year	Study Type	Population	Inhaled medication	Nebulizer And Position	Comparison and Finding/Aerosol deposition					
O'Doherty, 1992[1]	In vitro	Adults	99mtc HSA	Inspiratory synchronized JN	RR	**8/min**	**12/min**	**15/min**	**20/min**	**P**
						8.3 ± 0.78%*	11.2 ± 1.6%*	5.4 ± 0.15%	5.12 ± 0.14%	*p < 0.05
					MV	**6 L/min**	**9 L/min**	**12 L/min**	**15 L/min**	
						9.9 ± 0.9%*	5.4 ± 0.15%	5.8 ± 0.27%	3.75 ± 0.46%*	
Fink, 1996[2]	In vitro	Adult	Albuterol	MDI+Spacer	VT (CPAP)	**VT 800ml**	**VT 500ml**	**VT 300ml**	**VT 100ml**	**P**
						39.2 ± 1.4%	31.2±3.7%	21.6 ± 3.9%	4.9± 1.4%	< 0.003
Mouloudi E，1999[3]	In vivo	Adult: 9 COPD	Salbutamol	MDI+Spacer, inspiratory limb	8–9 mm tube, square wave, inspiratory flow 0.55~0.65 l/s and no end-inspiratory pause, individual MV, PEEP 0 cmH_2O		**VT 8 ml/kg**		**VT 12 ml/kg**	**P**
						Baseline	Rrs: 22.7±4.3<br>Rint: 18.2±3.2		Rrs: 21.4±3.4<br>Rint: 16.8±2.1	>0.05
						15min	Rrs: 19.9±2.60<br>Rint: 14.9±2.2		Rrs: 19.1±2.4<br>Rint: 14.1±2.0	
						30min	Rrs: 19.1±2.9<br>Rint: 14.4±2.1		Rrs: 19.3±2.6<br>Rint: 14.2±2.0	
						60min	Rrs: 19.7±3.7<br>Rint: 14.7±2.6		Rrs: 18.8±3.0<br>Rint: 14.5±2.7	
Williams,1999[4]	In vitro	Adult	Saline 0.9%	Ultrasonic nebulizer, inspiratory limb	Inspiratory flow 10.6L/min and inspiratory time 1.67s was constant	**MV 3.6 L/min (6*0.6 L)**		**MV 7.2 L/min (12* 0.6 L)**		**P**
					Nebulizer set at ‘1’#	3.2±0.8 ml		5.5±1.0 ml		< 0.05
					Nebulizer set at ‘2’ #	6.7±0.4 ml		9.5±0.2 ml		< 0.001
					Nebulizer set at ‘3’ #	8.2±0.6 ml		15.3±0.7 ml		< 0.001
					I:E ratio of 1:2 was constant	**MV 3.6 L/min (6*0.6 L), inspiratory flow 25.2 L/min, inspiratory time 1.67s**		**MV 7.2 L/min (12* 0.6 L), inspiratory flow 10.6 L/min, inspiratory time 3.33s**		**P**
					Nebulizer set at ‘1’#	4.9±0.3 ml		4.6±0.6 ml		> 0.05
					Nebulizer set at ‘2’ #	5.9±1.0 ml		7.5±0.7 ml		> 0.05
					Nebulizer set at ‘3’ #	9.20±0.6 ml		10.0±0.2 ml		> 0.05

**p < 0.05 compared with the default settings (RR 15/min, MV 9 L/min. Inspiratory time 25%)*
# Nebulizer set at ‘1’: low output, Nebulizer set at ‘2’: medium output, Nebulizer set at ‘3’: full output.

99mtc HAS, technetium~99m human serum albumin; JN, jet nebulizer; MV, minute ventilation; RR, respiratory rates; MDI, metered-dose inhaler; VT, tidal volume; CPAP, continuous positive airway pressure; COPD, chronic obstructive pulmonary disease; PEEP, positive end-expiratory pressure; Rrs and Rint: maximum and minimum inspiratory resistance, respectively ($cmH_2O/l/s$); I: E inspiration to expiration ratio.

1. O'Doherty MJ, Thomas SH, Page CJ, Treacher DF, Nunan TO. Delivery of a nebulized aerosol to a lung model during mechanical ventilation. Effect of ventilator settings and nebulizer type, position, and volume of fill. Am Rev Respir Dis. 1992 Aug;146(2):383~8.
2. Fink JB, Dhand R, Duarte AG, Jenne JW, Tobin MJ. Aerosol delivery from a metered-dose inhaler during mechanical ventilation. An in vitro model. Am J Respir Crit Care Med. 1996 Aug;154(2 Pt 1):382~7.
3. Mouloudi E, Katsanoulas K, Anastasaki M, Hoing S, Georgopoulos D, (1999) Bronchodilator delivery by metered-dose inhaler in mechanically ventilated COPD patients: influence of tidal volume. Intensive Care Med 25: 1215~1221
4. Williams L, Fletcher GC, Daniel M, Kinsella J. A simple in vitro method for the evaluation of an ultrasonic nebulizer for drug delivery to intubated, ventilated patients and the effect of nebulizer and ventilator settings on the uptake of fluid from the nebulizer chamber. Eur J Anaesthesiol. 1999 Jul;16(7):479~84

1.27.2 Recommendations for nebulized antibiotics in the consensuses and reviews.

Author, year	Nebulizer	Position	Mode	Vt	Inspiratory flow	I: E	RR	Bias flow	End-inspiratory pause	PEEP	Others
Rello J,2017	VMN	10–15 cm before the Y piece on the inspiratory limb	Volume-controlled	8ml/kg	Constant inspiratory flow	≤ 50%	12-15 bpm	/	20%	5-10 cmH_2O	• Avoid sharp angles and rough inner surfaces in circuit<br>• Avoid asynchronies and triggering<br>• Increase level of sedation if necessary<br>• Remove HME<br>• Turn off HH<br>• Change expiratory filter after each nebulization
Rouby JJ, 2020	VMN	10–15 cm before the Y piece on the inspiratory limb	Volume-controlled	8ml/kg	Limiting inspiratory flow velocity	1: 2	12-15 bpm	/	20%	5-10 cmH_2O	• Remove HME<br>• Turn off HH

Monsel A, 2021	VMN	15 cm before the Y piece on the inspiratory limb	Volume-controlled	8ml/kg	Constant inspiratory flow	1: 1	12-15 bpm	Absence or minimum (2 l/min)	20%	/	• Smooth angles and inner surface tube<br>• Increase level of sedation if necessary<br>• Remove HME<br>• Turn off HH

VMN vibrating mesh nebulizer, PDDS pulmonary drug delivery system, JN jet nebulizer, UN ultrasonic nebulizer, MV minute ventilation, Vt tidal volume, I: E inspiratory to expiratory ratio, RR respiratory rate, PEEP positive end-expiration pressure, HME heat and moisture exchanger, HH heated humidifiers,

1. Rello J, Rouby JJ, Sole-Lleonart C, Chastre J, Blot S, Luyt CE, Riera J, Vos MC, Monsel A, Dhanani J, Roberts JA. Key considerations on nebulization of antimicrobial agents to mechanically ventilated patients. Clin Microbiol Infect. 2017 Sep;23(9):640-646.
2. Rouby JJ, Sole-Lleonart C, Rello J; European Investigators Network for Nebulized Antibiotics in Ventilator-associated Pneumonia. Ventilator-associated pneumonia caused by multidrug-resistant Gram-negative bacteria: understanding nebulization of aminoglycosides and colistin. Intensive Care Med. 2020 Apr;46(4):766-770. Rouby JJ, Sole-Lleonart C, Rello J; European Investigators Network for Nebulized Antibiotics in Ventilator-associated Pneumonia. Ventilator-associated pneumonia caused by multidrug-resistant Gram-negative bacteria: understanding nebulization of aminoglycosides and colistin. Intensive Care Med. 2020 Apr;46(4):766-770.
3. Monsel A, Torres A, Zhu Y, Pugin J, Rello J, Rouby JJ; European Investigators Network for Nebulized Antibiotics in Ventilator-associated Pneumonia (ENAVAP). Nebulized antibiotics for ventilator-associated pneumonia: methodological framework for future multicenter randomized controlled trials. Curr Opin Infect Dis. 2021 Apr 1;34(2):156-168.

Section 1: Aerosol Delivery via Invasive Ventilation for Adult Patients

Recommendation 1.28： Longer inspiratory time and lower inspiratory flows are associated with improved aerosol delivery efficiency, however, changing those parameters solely for aerosol delivery is not recommended.

Distribution of voting scores

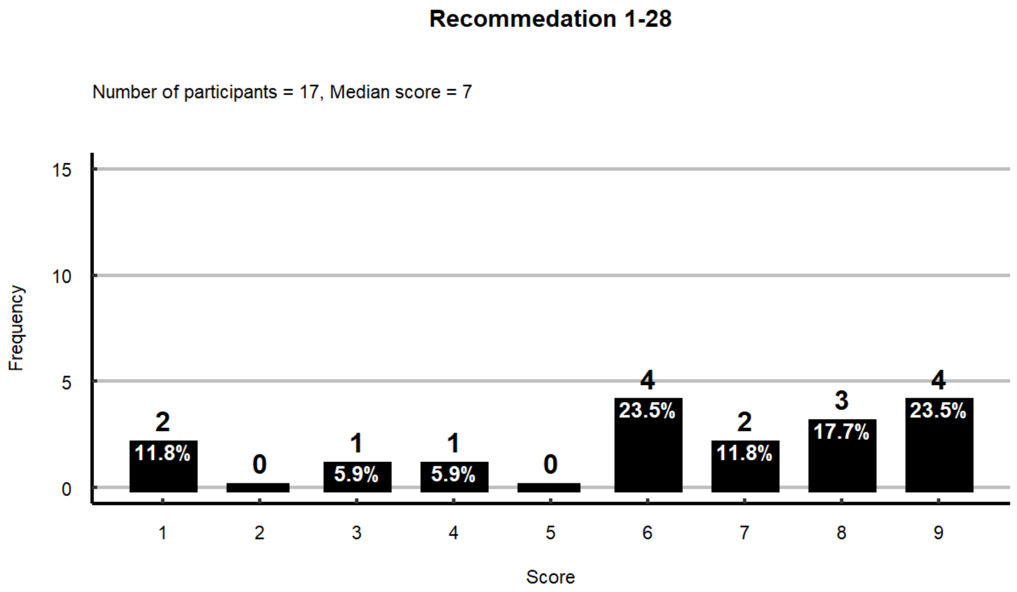

Comments of voting panelists

1. Not possible to deliver recommendation based only in in vivo. Rouby JJ, Sole-Lleonart C, Rello J; Intensive Care Med. 2020;46:832

2. If there is not harm why not to do it just for aerosolization?

3. Changing ventilator settings may have adverse effects. Longer inspiratory time and slower inspiratory flows may reduce time for exhalation and have unintended consequence of increasing PEEPi (e.g. COPD, asthma).

4. It can be necessary for antibiotics.

5. In vitro studies have clearly shown the benefits of longer inspiratory time and lower inspiratory flow rate in terms of aerosol delivery efficiency. Unfortunately, we have very few clinical studies to compare clinical outcomes between optimized and non-optimized ventilator settings, especially when it comes to antibiotic nebulization.

6. Potential harm outweighs benefit but evidence weak.

7. Again this applies only during controlled mechanical ventilation and not assisted ventilation – this should be commented and considered.

8. Inspiratory flow turbulences resulting from short inspiration time (≤ 30% of the duty

cycle) and high inspiratory flows (constant inspiratory flows > 30 L/min or decelerating flows with a high peak inspiratory flow used in pressure support mode) cause the impaction of aerosolized particles on ventilator circuits and tracheobronchial walls. Lengthening inspiration time and using low constant inspiratory flows to decrease inspiratory flow turbulences reduce the impaction of aerosolized particles in ventilator circuits and bronchi and promote lung parenchymal deposition. If bronchodilation is the therapeutic target, limiting inspiratory flow turbulences resulting from short inspiration time and high inspiratory flows does not really improves the bronchodilating effect. If bacterial killing in the infected lung parenchyma is the therapeutic target, then inspiratory flow turbulences resulting from short inspiration time and high inspiratory flows, are deleterious for the patients with ventilator-associated pneumonia. Although there is no formal demonstration of the latter statement, the majority of experts and academic societies recommend to lengthen inspiration time and reduce high inspiratory flows during the nebulization of antibiotics to treat ventilator-associated pneumonia. As a consequence, I suggest to follow these recommendations based on the corresponding references (above) and to provide this information in the recommendation (outlined in yellow). I also suggest to recommend experimental studies based on lung microdialysis to demonstrate that increasing inspiration time and reducing high inspiratory flows during the nebulization period increases lung deposition of aerosolized antibiotics.

9. This should be merged with 1.27.

Round 2

**Recommendations 1.28.1**	Longer inspiratory time and lower inspiratory flows are associated with improved aerosol delivery efficiency, however, changing those parameters solely for aerosol delivery is not recommended.
**Likert score of 1-9**	□1 □2 □3 □4 □5 □6 □7 □8 □9<br>1= absolutely disagree, 9 = absolutely agree
**Comments**	

[Additional recommendations based on reviewer suggestion]

**Recommendations 1.28.2**	**When delivering inhaled antibiotics for invasively ventilated patients, it is recommended to keep inspiratory flow below 40L/min.**
**Likert score of 1-9**	□1 □2 □3 □4 □5 □6 □7 □8 □9<br>1= absolutely disagree, 9 = absolutely agree
**Comments**	

**Recommendations 1.28.3**	**When delivering inhaled antibiotics for invasively ventilated patients, it is recommended to use inversed inspiratory to expiratory ratio of 50%.**
**Likert score of 1-9**	□1 □2 □3 □4 □5 □6 □7 □8 □9<br>1= absolutely disagree, 9 = absolutely agree
**Comments**	

Table1.28.1 Studies of change inspiratory flow and inspiratory time

<table>
<tr><th>Author, year</th><th>Study Type</th><th>Population</th><th>Inhaled medication</th><th>Nebulizer And Position</th><th colspan="5">Comparison and Finding/Aerosol deposition</th></tr>
<tr><td rowspan="3">O'Doherty, 1992[1]</td><td rowspan="3">In vitro</td><td rowspan="3">Adults</td><td rowspan="3">99mtc HSA</td><td rowspan="3">Inspiratory synchronized JN</td><td colspan="5">Inspiratory time</td></tr>
<tr><td>20%</td><td>25%</td><td>33%</td><td>50%</td><td>P</td></tr>
<tr><td>5.0 ± 0.84%</td><td>5.4 ± 0.15%</td><td>13.3 ± 0.12%*</td><td>17.4 ± 0.40%*</td><td>*p < 0.05.</td></tr>
<tr><td rowspan="2">Fink, 1996[2]</td><td rowspan="2">In vitro</td><td rowspan="2">Adult</td><td rowspan="2">Albuterol</td><td rowspan="2">MDI+Spacer</td><td colspan="5">Ti/Ttot (CMV, AC, PS)</td></tr>
<tr><td colspan="5">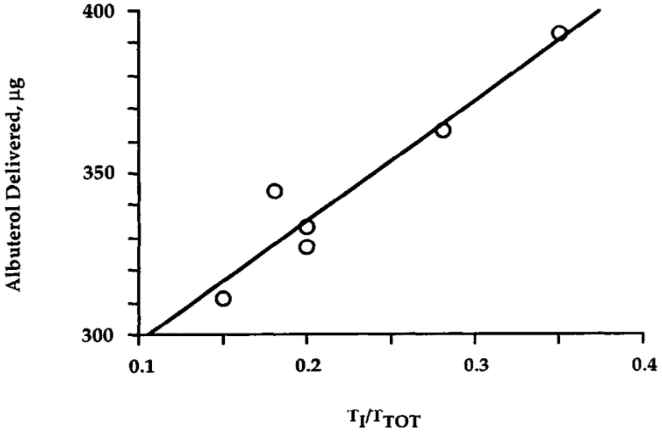

Figure 2. Correlation between albuterol delivery and duty cycle (TI/Ttot) after administration of 12 puffs with controlled mechanical ventilation (VT, 800 and 500 ml), assist control (VT, 800 ml), and pressure support of 10 and 20 cm H_2O (VT, 700 and 800 ml, respectively). Albuterol delivery was linearly related to TI/Ttot (r = 0.96, p < 0.003).
Albuterol delivery was linearly related to Ti/Ttot (r =0.96, P < 0.003)</td></tr>
<tr><td rowspan="5">Fink, 1999[3]</td><td rowspan="5">In vitro</td><td rowspan="5">Adult</td><td rowspan="5">Albuterol</td><td rowspan="5">MDI+Spacer</td><td colspan="2"></td><td>Ti/Ttot : 0.5</td><td>Ti/Ttot : 0.25</td><td>P</td></tr>
<tr><td colspan="2">Inspiratory flow 40L/min</td><td>27.4±0.4%</td><td>24.1±0.7%</td><td><0.002</td></tr>
<tr><td colspan="2">Inspiratory flow 80L/min</td><td>10.2±0.1%</td><td>9.4± 0.0%</td><td><0.04</td></tr>
<tr><td colspan="2">Ti/Ttot constant</td><td>Inspiratory flow 40L/min</td><td>Inspiratory flow 80L/min</td><td>P</td></tr>
<tr><td colspan="2"></td><td>25.8±0.6%</td><td>9.8 ±0.1%</td><td><0.0001</td></tr>
</table>

Williams,1999[4]	In vitro	Adult	Saline 0.9%	Ultrasonic nebulizer, inspiratory limb	MV:7.2L/min (12*0.6L)	**Inspiratory flow 34.3 L/min, I: E 1:4**	**Inspiratory flow 8.4 L/min, I: E 4:1**	**P**
					Nebulizer set at ‘1’#	3.2±0.3 ml	2.5±0.8 ml	> 0.05
					Nebulizer set at ‘2’ #	6.4±0.7 ml	11.0±0.6 ml	< 0.01
					Nebulizer set at ‘3’ #	9.1±0.6 ml	18.7±0.2 ml	< 0.001

Hess, 2003[5]	In vitro	Adult	Albuterol	Inspiratory synchronized JN	VT 0.6 L, RR 15/min, PEEP 5 cmH_2O		**Inspiratory times 1s**	**Inspiratory times 2s**	**P**
					VCV, constant flow	Long time constant	0.17±0.01mg	0.59±0.05 mg	<0.001
						Short time constant	0.14±0.01 mg	0.41±0.01 mg	
					VCV, descending ramp flow	Long time constant	0.17±0.04 mg	0.43±0.07 mg	
						Short time constant	0.13±0.01 mg	0.50±0.02 mg	
					PCV	Long time constant	0.34±0.02 mg	0.59±0.05 mg	
						Short time constant	0.10±0.01 mg	0.09±0.01 mg	
				MDI+Spacer	VCV, constant flow	Long time constant	0.20±0.01 mg	0.18±0.02 mg	0.37
						Short time constant	0.23±0.01 mg	0.22±0.01 mg	
					VCV, descending ramp flow	Long time constant	0.20±0.01 mg	0.20±0.03 mg	
						Short time constant	0.20±0.01 mg	0.21±0.01 mg	
					PCV	Long time constant	0.21±0.01 mg	0.24±0.01 mg	
						Short time constant	0.20±0.01 mg	0.21±0.01 mg	

Vecellio, 2005[6]	In vitro	Adult	Terbutaline	Continuous JN, inspiratory line		**Inspiratory flow**				**P**
					VT 500ml, RR 15/min	Ti/Ttot 0.2, inspiratory	Ti/Ttot 0.3, inspiratory	Ti/Ttot 0.4, inspiratory	Ti/Ttot 0.5, inspiratory	p<0.0001

		flow 37.5 L/min	flow 25.0 L/min	flow 18.8 L/min	flow 15.0 L/min
		35 (28–37) %	37 (36–41) %	37 (36–40) %	42 (42–44) %
	VT 600ml, RR 12/min	Ti/Ttot 0.2, inspiratory flow 36.0 L/min	Ti/Ttot 0.3, inspiratory flow 24.0 L/min	Ti/Ttot 0.4, inspiratory flow 18.0 L/min	Ti/Ttot 0.5, inspiratory flow 14.4 L/min
		39 (38–42) %	40 (39–42) %	43 (42–45) %	44 (44–48) %

**p < 0.05 compared with the default settings (RR 15/min, MV 9 L/min. Inspiratory time 25%)*

#Nebulizer set at ‘1’: low output, Nebulizer set at ‘2’: medium output, Nebulizer set at ‘3’: full output.

99mtc HAS, technetium~99m human serum albumin; JN, jet nebulizer; MV, minute ventilation; RR, respiratory rates;MDI, metered-dose inhaler; CMV, controlled mechanical ventilation; A/C, assist/control; PS, pressure support; Ti, inspiratory time; Ttot, total time; I:E, inspiration to expiration ratio; PEEP, positive end-expiratory pressure; VT, tidal volume; VCV, volume control ventilation; PCV, pressure control ventilation;VMD, volume median diameter.

1. O'Doherty MJ, Thomas SH, Page CJ, Treacher DF, Nunan TO. Delivery of a nebulized aerosol to a lung model during mechanical ventilation. Effect of ventilator settings and nebulizer type, position, and volume of fill. Am Rev Respir Dis. 1992 Aug;146(2):383~8.
2. Fink JB, Dhand R, Duarte AG, Jenne JW, Tobin MJ. Aerosol delivery from a metered-dose inhaler during mechanical ventilation. An in vitro model. Am J Respir Crit Care Med. 1996 Aug;154(2 Pt 1):382~7.
3. Fink JB, Dhand R, Grychowski J, Fahey PJ, Tobin MJ. Reconciling in vitro and in vivo measurements of aerosol delivery from a metered-dose inhaler during mechanical ventilation and defining efficiency-enhancing factors. Am J Respir Crit Care Med. 1999 Jan;159(1):63~8.
4. Williams L, Fletcher GC, Daniel M, Kinsella J. A simple in vitro method for the evaluation of an ultrasonic nebulizer for drug delivery to intubated, ventilated patients and the effect of nebulizer and ventilator settings on the uptake of fluid from the nebulizer chamber. Eur J Anaesthesiol. 1999 Jul;16(7):479~84.
5. Hess DR, Dillman C, Kacmarek RM. In vitro evaluation of aerosol bronchodilator delivery during mechanical ventilation: pressure-control vs. Volume control ventilation. Intensive Care Med. 2003 Jul;29(7):1145-50.
6. Vecellio L, Guérin C, Grimbert D, De Monte M, Diot P. In vitro study and semiempirical model for aerosol delivery control during mechanical ventilation. Intensive Care Med. 2005 Jun;31(6):871-6.

1.28.2 Recommendations for nebulized antibiotics in the consensuses and reviews.

Author, year	Nebulizer	Position	Mode	Vt	Inspiratory flow	I: E	RR	Bias flow	End-inspiratory pause	PEEP	Others
Rello J,2017	VMN	10–15 cm before the Y piece on the inspiratory limb	Volume-controlled	8ml/kg	Constant inspiratory flow	≤ 50%	12-15 bpm	/	20%	5-10 cmH_2O	• Avoid sharp angles and rough inner surfaces in circuit<br>• Avoid asynchronies and triggering<br>• Increase level of sedation if necessary<br>• Remove HME<br>• Turn off HH<br>• Change expiratory filter after each nebulization
Rouby JJ, 2020	VMN	10–15 cm before the Y piece on the inspiratory limb	Volume-controlled	8ml/kg	Limiting inspiratory flow velocity	1: 2	12-15 bpm	/	20%	5-10 cmH_2O	• Remove HME<br>• Turn off HH
Monsel A, 2021	VMN	15 cm before the Y piece on the inspiratory limb	Volume-controlled	8ml/kg	Constant inspiratory flow	1: 1	12-15 bpm	Absence or minimum (2 l/min)	20%	/	• Smooth angles and inner surface tube<br>• Increase level of sedation if necessary<br>• Remove HME<br>• Turn off HH

VMN vibrating mesh nebulizer, PDDS pulmonary drug delivery system, JN jet nebulizer, UN ultrasonic nebulizer, MV minute ventilation, Vt tidal volume, I: E inspiratory to expiratory ratio, RR respiratory rate, PEEP positive end-expiration pressure, HME heat and moisture exchanger, HH heated humidifiers,

1. Rello J, Rouby JJ, Sole-Lleonart C, Chastre J, Blot S, Luyt CE, Riera J, Vos MC, Monsel A, Dhanani J, Roberts JA. Key considerations on nebulization of antimicrobial agents to mechanically ventilated patients. Clin Microbiol Infect. 2017 Sep;23(9):640-646.
2. Rouby JJ, Sole-Lleonart C, Rello J; European Investigators Network for Nebulized Antibiotics in Ventilator-associated Pneumonia. Ventilator-associated pneumonia caused by multidrug-resistant Gram-negative bacteria: understanding nebulization of aminoglycosides and colistin. Intensive Care Med. 2020 Apr;46(4):766-770. Rouby JJ, Sole-Lleonart C, Rello J; European Investigators Network for Nebulized Antibiotics in Ventilator-associated Pneumonia. Ventilator-associated pneumonia caused by multidrug-resistant Gram-negative bacteria: understanding nebulization of aminoglycosides and colistin. Intensive Care Med. 2020 Apr;46(4):766-770.

3. Monsel A, Torres A, Zhu Y, Pugin J, Rello J, Rouby JJ; European Investigators Network for Nebulized Antibiotics in Ventilator-associated Pneumonia (ENAVAP). Nebulized antibiotics for ventilator-associated pneumonia: methodological framework for future multicenter randomized controlled trials. Curr Opin Infect Dis. 2021 Apr 1;34(2):156-168.

Section 1: Aerosol Delivery via Invasive Ventilation for Adult Patients

Recommendation 1.29：It is not recommended to change the inspiratory flow patterns solely for aerosol delivery.

Distribution of voting scores

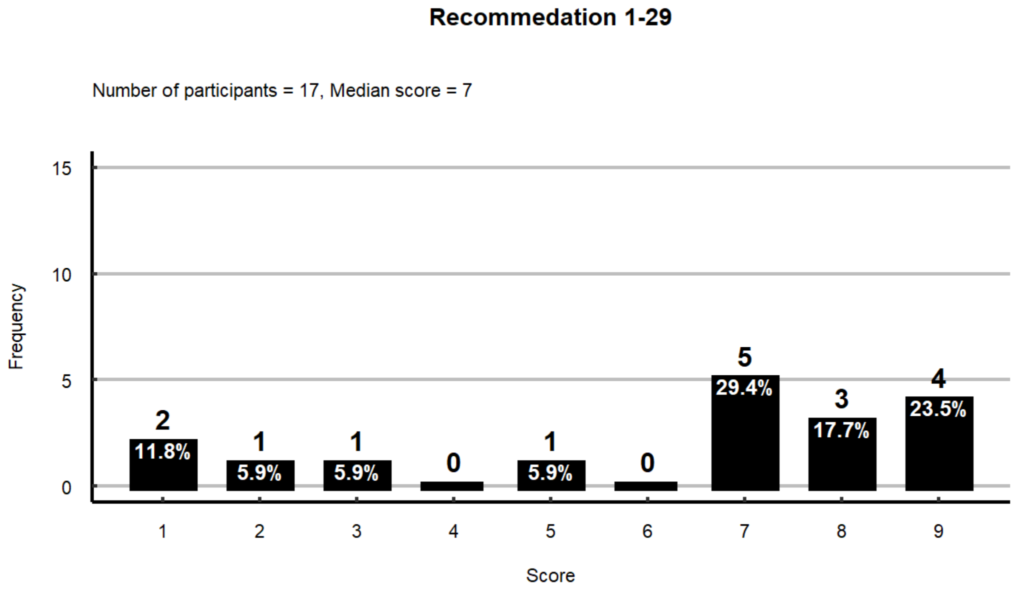

Comments of voting panelists

1. Inspiratory flow must be 6-8 L Rouby JJ, Sole-Lleonart C, Rello J; Intensive Care Med. 2020;46:832.

2. I agree as a general rule.

3. Limited in vitro data suggested that inspiratory flow patterns may influence aerosol delivery.

4. Potential harm outweighs benefit.

5. Again this is considered during controlled mechanical ventilation – different is during assisted ventilation or CPAP (especially with high flow or high flow oxygen nasal therapy).

6. Inspiratory flow turbulences resulting from a decelarating inspiratory flow (as used during pressure support) cause the impaction of aerosolized particles on ventilator circuits and tracheobronchial walls. Using a low constant inspiratory flows to decrease inspiratory flow turbulences reduce the impaction of aerosolized particles in ventilator circuits and bronchi and promote lung parenchymal deposition. If bronchodilation is the therapeutic target, limiting inspiratory flow turbulences resulting from a decelarating inspiratory flow does not really improves the bronchodilating effect. If

bacterial killing in the infected lung parenchyma is the therapeutic target, then inspiratory flow turbulences resulting from a decelarating inspiratory flow, are deleterious for the patients with ventilator-associated pneumonia. Although there is no formal demonstration of the latter statement, the majority of experts and academic societies recommend to a constant low inspiratory flow during the nebulization of antibiotics to treat ventilator-associated pneumonia. As a consequence, I suggest to follow these recommendations based on the corresponding references (above) and to provide this information in the recommendation (outlined in yellow). I also suggest to recommend experimental studies based on lung microdialysis to demonstrate that using a constant inspiratory flow rather than a decelerating inspiratory flow during the nebulization period increases lung deposition of aerosolized antibiotics.

7. May be needed to improve synchronization.
8. This should be merged with 1.27 and 1.28.

Round 2

**Recommendations 1.29.1**	It is not recommended to change the inspiratory flow patterns solely for aerosol delivery.
**Likert score of 1-9**	□1 □2 □3 □4 □5 □6 □7 □8 □9<br>1= absolutely disagree, 9 = absolutely agree
**Comments**	

[Additional recommendations based on reviewer suggestion]

**Recommendations 1.29.2**	**When delivering inhaled antibiotics for invasively ventilated patients, it is recommended to use a constant inspiratory flow.**
**Likert score of 1-9**	□1 □2 □3 □4 □5 □6 □7 □8 □9<br>1= absolutely disagree, 9 = absolutely agree
**Comments**	

Table1.29.1 Studies of different inspiratory flow pattern

Author, year	Study Type	Population	Inhaled medication	Nebulizer And Position	Comparison and Finding/Aerosol deposition					
Fink, 1996[1]	In vitro	Adult	Albuterol	MDI+spacer	Inspiratory flow pattern (CMV, VT 800 ml)		Sinusoidal flow	Decelerating flow	Square flow	P
							362.9 ± 40.4 ug	392.9 ± 33.9 ug	327.3 ± 36.8 ug	< 0.01
Mouloudi, 2000[2]	In vivo	Adult: 18 COPD	Salbutamol	MDI+Spacer, inspiratory limb	8–9 mm tube, Individual MV, no end-inspiratory pause, PEEP 0 cmH_2O		Volume control*	Pressure control[#]		P
						Baseline	Rrs: 26.3±2.9<br>Rint: 21.0±2.4	Rrs: 26.3±2.9<br>Rint: 21.2±3.3		>0.05
						15min	Rrs: 22.8±3.6<br>Rint: 17.9±3.3	Rrs: 22.4±3.3<br>Rint: 17.6±3.2		
						30min	Rrs: 22.8±2.7<br>Rint: 17.9±2.6	Rrs: 22.8±2.8<br>Rint: 17.9±2.6		
						60min	Rrs: 22.8±2.7<br>Rint: 17.9±2.6	Rrs: 23.7±3.2<br>Rint: 18.5±2.9		
Hess, 2003[3]	In vitro	Adult	Albuterol	Inspiratory synchronized JN	VT 0.6 L, RR 15/min, PEEP 5 cmH_2O		VCV, constant flow	VCV, descending ramp flow	PCV	P
					Long time constant	Ti: 1s	0.17±0.01mg	0.17±0.04 mg	0.34±0.02 mg	0.03
						Ti:2s	0.59±0.05 mg	0.43±0.07 mg	0.59±0.05 mg	
					Short time constant	Ti: 1s	0.14±0.01 mg	0.13±0.01 mg	0.10±0.01 mg	
						Ti:2s	0.41±0.01 mg	0.50±0.02 mg	0.09±0.01 mg	
				MDI+Spacer	Long time constant	Ti: 1s	0.20±0.01 mg	0.20±0.01 mg	0.21±0.01 mg	0.37
						Ti:2s	0.18±0.02 mg	0.20±0.03 mg	0.24±0.01 mg	
					Short time constant	Ti: 1s	0.23±0.01 mg	0.20±0.01 mg	0.20±0.01 mg	
						Ti:2s	0.22±0.01 mg	0.21±0.01 mg	0.21±0.01 mg	
Dugernier,	In vitro	Adult	Amikacin	Vibrating mesh	Decelerating inspiratory flow vs constant flow pattern, with or without proximal flow sensor					

2015[4]

nebulizer

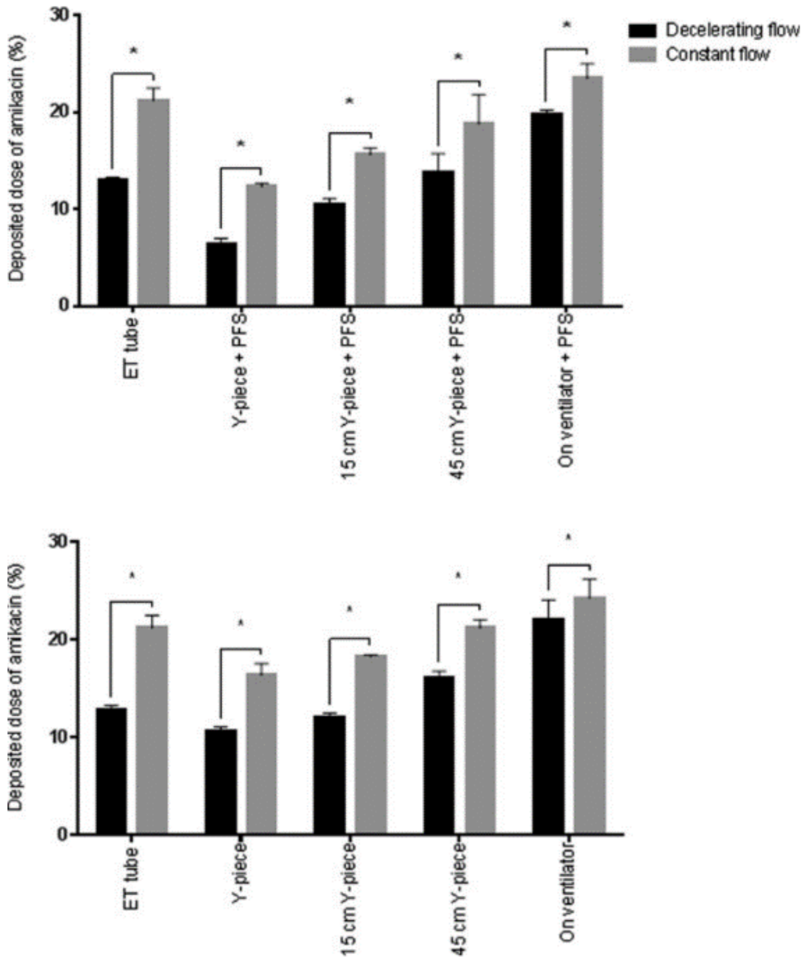

$P < 0.05$

**Volume control: VT 8 ml/kg, square wave flow, inspiratory flow 0.55–0.65 L/s;*
#Pressure control, the pressure level (achieve a VT 8 ml/kg), inspiratory time was kept constant.

pMDI, pressurized metered-dose inhaler; JN, jet nebulizer; VT, tidal volume; CMV, controlled mechanical ventilation; COPD, chronic obstructive pulmonary disease; MV, minute ventilation; PEEP, positive end-expiratory pressure; Rrs and Rint, maximum and minimum inspiratory resistance, respectively, (cmH_2O/l/s); RR,respiratory rates; PEEP, positive end-expiratory pressure; VCV, volume control ventilation;PCV, pressure control ventilation.

1. Fink JB, Dhand R, Duarte AG, Jenne JW, Tobin MJ. Aerosol delivery from a metered-dose inhaler during mechanical ventilation. An in vitro model. Am J Respir Crit Care Med. 1996 Aug;154(2 Pt 1):382~7.
2. Mouloudi E, Prinianakis G, Kondili E, Georgopoulos D, (2000) Bronchodilator delivery by metered-dose inhaler in mechanically ventilated COPD patients: influence of flow pattern. The European respiratory journal 16: 263~268
3. Hess DR, Dillman C, Kacmarek RM. In vitro evaluation of aerosol bronchodilator delivery during mechanical ventilation: pressure-control vs. Volume control ventilation. Intensive Care Med. 2003 Jul;29(7):1145-50.
4. Dugernier J, Wittebole X, Roeseler J, Michotte JB, Sottiaux T, Dugernier T, Laterre PF, Reychler G. Influence of inspiratory flow pattern and nebulizer position on aerosol delivery with a vibrating-mesh nebulizer during invasive mechanical ventilation: an in vitro analysis. J Aerosol Med Pulm Drug Deliv. 2015 Jun;28(3):229-36.

1.29.2 Recommendations for nebulized antibiotics in the consensuses and reviews.

Author, year	Nebulizer	Position	Mode	Vt	Inspiratory flow	I: E	RR	Bias flow	End-inspiratory pause	PEEP	Others
Rello J,2017	VMN	10–15 cm before the Y piece on the inspiratory limb	Volume-controlled	8ml/kg	Constant inspiratory flow	≤ 50%	12-15 bpm	/	20%	5-10 cmH_2O	• Avoid sharp angles and rough inner surfaces in circuit<br>• Avoid asynchronies and triggering<br>• Increase level of sedation if necessary<br>• Remove HME<br>• Turn off HH<br>• Change expiratory filter after each nebulization
Rouby JJ, 2020	VMN	10–15 cm before the Y piece on the inspiratory limb	Volume-controlled	8ml/kg	Limiting inspiratory flow velocity	1: 2	12-15 bpm	/	20%	5-10 cmH_2O	• Remove HME<br>• Turn off HH

Monsel A, 2021	VMN	15 cm before the Y piece on the inspiratory limb	Volume-controlled	8ml/kg	Constant inspiratory flow	1: 1	12-15 bpm	Absence or minimum (2 l/min)	20%	/	• Smooth angles and inner surface tube<br>• Increase level of sedation if necessary<br>• Remove HME<br>• Turn off HH

VMN vibrating mesh nebulizer, PDDS pulmonary drug delivery system, JN jet nebulizer, UN ultrasonic nebulizer, MV minute ventilation, Vt tidal volume, I: E inspiratory to expiratory ratio, RR respiratory rate, PEEP positive end-expiration pressure, HME heat and moisture exchanger, HH heated humidifiers,

1. Rello J, Rouby JJ, Sole-Lleonart C, Chastre J, Blot S, Luyt CE, Riera J, Vos MC, Monsel A, Dhanani J, Roberts JA. Key considerations on nebulization of antimicrobial agents to mechanically ventilated patients. Clin Microbiol Infect. 2017 Sep;23(9):640-646.
2. Rouby JJ, Sole-Lleonart C, Rello J; European Investigators Network for Nebulized Antibiotics in Ventilator-associated Pneumonia. Ventilator-associated pneumonia caused by multidrug-resistant Gram-negative bacteria: understanding nebulization of aminoglycosides and colistin. Intensive Care Med. 2020 Apr;46(4):766-770. Rouby JJ, Sole-Lleonart C, Rello J; European Investigators Network for Nebulized Antibiotics in Ventilator-associated Pneumonia. Ventilator-associated pneumonia caused by multidrug-resistant Gram-negative bacteria: understanding nebulization of aminoglycosides and colistin. Intensive Care Med. 2020 Apr;46(4):766-770.
3. Monsel A, Torres A, Zhu Y, Pugin J, Rello J, Rouby JJ; European Investigators Network for Nebulized Antibiotics in Ventilator-associated Pneumonia (ENAVAP). Nebulized antibiotics for ventilator-associated pneumonia: methodological framework for future multicenter randomized controlled trials. Curr Opin Infect Dis. 2021 Apr 1;34(2):156-168.

Section 1: Aerosol Delivery via Invasive Ventilation for Adult Patients

Recommendation 1.30： It is not recommended to apply end-inspiratory pause when metered dose inhaler is used during invasive mechanical ventilation.

Distribution of voting scores

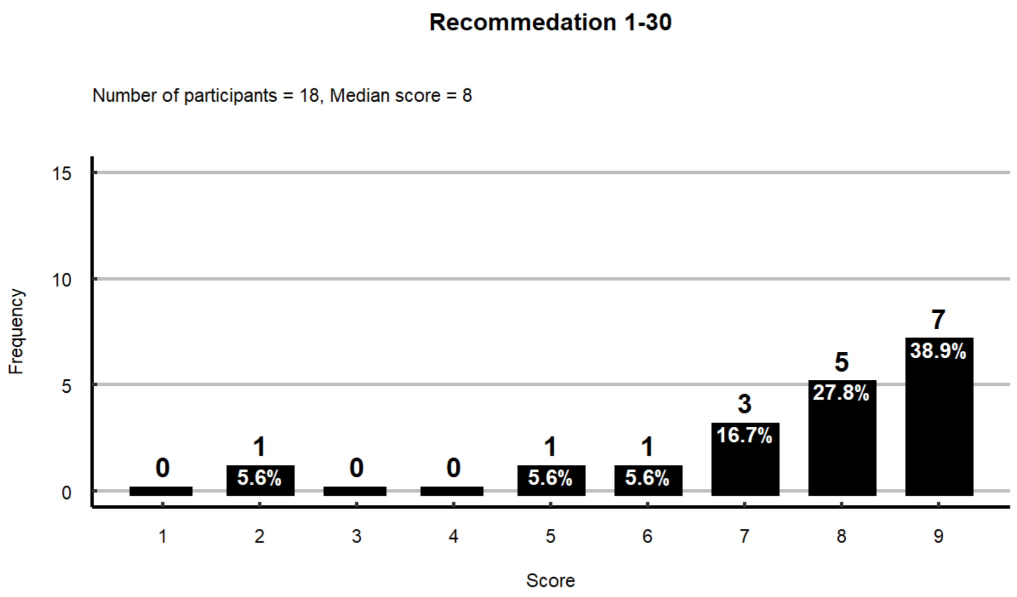

Comments of voting panelists

1. Only one study.

2. Limited data suggested that applying end-inspiratory pause doesn't enhance bronchodilator response when pMDI is used in COPD patients.

3. Potential harm outweighs benefit.

4. Complicated – and again applicable only in controlled and not assisted ventilation or spontaneous respiratory support.

5. Would this be considered a change in ventilator settings? If so, it should be merged with 1.27, 1.28, and 1.29.

Round 2

**Recommendations 1.30.1**	It is not recommended to apply end-inspiratory pause when metered dose inhaler is used during invasive mechanical ventilation.
**Likert score of 1-9**	□1 □2 □3 □4 □5 □6 □7 □8 □9<br>1= absolutely disagree, 9 = absolutely agree

**Comments**	

[Additional recommendations based on reviewer suggestion]

**Recommendations 1.30.2**	**When delivering inhaled antibiotics for invasively ventilated patients, it is recommended to set end-inspiratory pause at 20%.**
**Likert score of 1-9**	□1 □2 □3 □4 □5 □6 □7 □8 □9<br>1= absolutely disagree, 9 = absolutely agree
**Comments**	

Table1.30.1 Studies of with or without end-inspiratory pause

Author, year	Study Type	Population	Inhaled medication	Nebulizer and position	Comparison and Finding/Aerosol deposition (%)				
							Without EIP 5 s	With EIP 5 s	P
Mouloudi E，1998[1]	In vivo	Adult: 12 COPD	Salbutamol	MDI+Spacer, inspiratory limb	8–9 mm tube, specific VT, square wave, individual MV, PEEP 0 cmH_2O	Baseline	Rmin:10.7±3.0<br>Rmax: 18.4±3.0	Rmin:11.9±2.8<br>Rmax: 18.2±4.3	>0.05
						15min	Rmin:10.7±3.0<br>Rmax: 18.4±3.0	Rmin:11.9±2.8<br>Rmax: 18.2±4.3	
						30min	Rmin:10.4±2.1<br>Rmax: 17.8±2.8	Rmin:12.3±2.8<br>Rmax: 20.0±3.2	
						60min	Rmin:11.9±2.6<br>Rmax: 18.0±2.5	Rmin:12.4±2.5<br>Rmax: 19.5±2.7	

COPD, chronic obstructive pulmonary disease; MDI, metered-dose inhaler; VT, tidal volume; MV, minute ventilation; PEEP, positive end-expiratory pressure; EIP, end-inspiratory pause; Rmin and Rmax, minimum and maximum inspiratory resistance.

1. Mouloudi E, Katsanoulas K, Anastasaki M, Askitopoulou E, Georgopoulos D, (1998) Bronchodilator delivery by metered-dose inhaler in mechanically ventilated COPD patients: influence of end-inspiratory pause. The European respiratory journal 12: 165~169

1.30.2 Recommendations for nebulized antibiotics in the consensuses and reviews.

Author, year	Nebulizer	Position	Mode	Vt	Inspiratory flow	I: E	RR	Bias flow	End-inspiratory pause	PEEP	Others
Rello J,2017	VMN	10–15 cm before the Y piece on the inspiratory limb	Volume-controlled	8ml/kg	Constant inspiratory flow	≤ 50%	12-15 bpm	/	20%	5-10 cmH_2O	• Avoid sharp angles and rough inner surfaces in circuit<br>• Avoid asynchronies and triggering<br>• Increase level of sedation if necessary<br>• Remove HME<br>• Turn off HH<br>• Change expiratory filter after each nebulization

Rouby JJ, 2020	VMN	10–15 cm before the Y piece on the inspiratory limb	Volume-controlled	8ml/kg	Limiting inspiratory flow velocity	1: 2	12-15 bpm	/	20%	5-10 cmH_2O	• Remove HME<br>• Turn off HH
Monsel A, 2021	VMN	15 cm before the Y piece on the inspiratory limb	Volume-controlled	8ml/kg	Constant inspiratory flow	1: 1	12-15 bpm	Absence or minimum (2 l/min)	20%	/	• Smooth angles and inner surface tube<br>• Increase level of sedation if necessary<br>• Remove HME<br>• Turn off HH

VMN vibrating mesh nebulizer, PDDS pulmonary drug delivery system, JN jet nebulizer, UN ultrasonic nebulizer, MV minute ventilation, Vt tidal volume, I: E inspiratory to expiratory ratio, RR respiratory rate, PEEP positive end-expiration pressure, HME heat and moisture exchanger, HH heated humidifiers,

1. Rello J, Rouby JJ, Sole-Lleonart C, Chastre J, Blot S, Luyt CE, Riera J, Vos MC, Monsel A, Dhanani J, Roberts JA. Key considerations on nebulization of antimicrobial agents to mechanically ventilated patients. Clin Microbiol Infect. 2017 Sep;23(9):640-646.
2. Rouby JJ, Sole-Lleonart C, Rello J; European Investigators Network for Nebulized Antibiotics in Ventilator-associated Pneumonia. Ventilator-associated pneumonia caused by multidrug-resistant Gram-negative bacteria: understanding nebulization of aminoglycosides and colistin. Intensive Care Med. 2020 Apr;46(4):766-770.
3. Monsel A, Torres A, Zhu Y, Pugin J, Rello J, Rouby JJ; European Investigators Network for Nebulized Antibiotics in Ventilator-associated Pneumonia (ENAVAP). Nebulized antibiotics for ventilator-associated pneumonia: methodological framework for future multicenter randomized controlled trials. Curr Opin Infect Dis. 2021 Apr 1;34(2):156-168.

Section 1: Aerosol Delivery via Invasive Ventilation for Adult Patients

Recommendation 1.31： It is not recommended to change the positive end-expiratory pressure (PEEP) for the sole purpose of improving aerosol delivery efficiency.

Distribution of voting scores

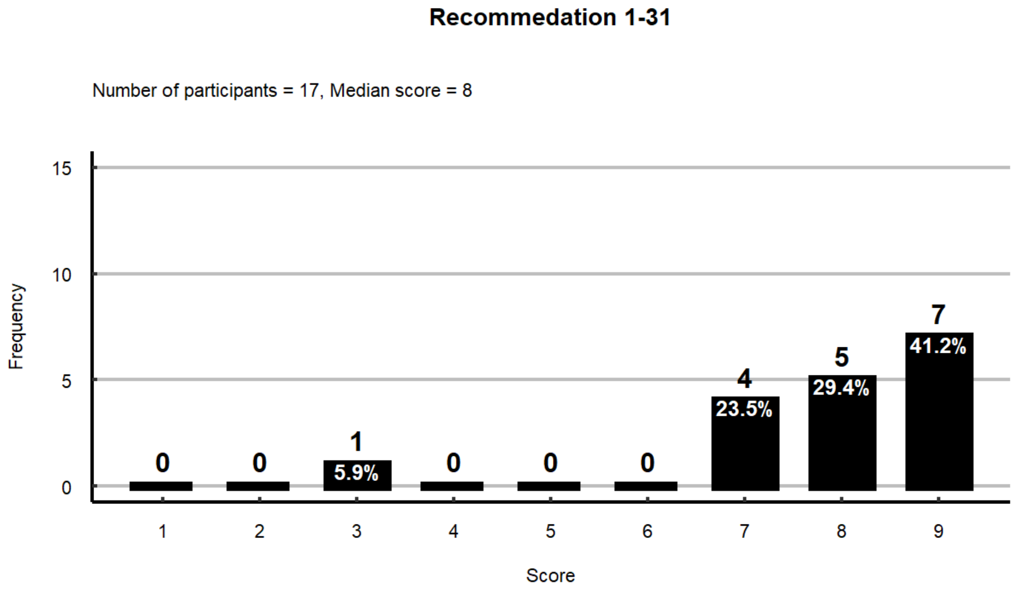

Comments of voting panelists

1. Scarce evidence but very logical evidence.

2. Limited clinical data suggested that increasing the PEEP level to 80% of the PEEPi level doesn't enhance the bronchodilator response in COPD patients. Moreover, changing PEEP may have adverse effects (e.g. alter cardiac output, oxygenation…).

3. Potential harm outweighs benefit.

4. This might be different during CPAP .See our studies: Sutherasan Y, Ball L, Raimondo P, Caratto V, Sanguineti E, Costantino F, Ferretti M, Kacmarek RM, Pelosi P. Effects of ventilator settings, nebulizer and exhalation port position on albuterol delivery during non-invasive ventilation: an in-vitro study. BMC Pulm Med. 2017 Jan 10;17(1):9. doi: 10.1186/s12890-016-0347-5. PMID: 28068958; PMCID: PMC5223303.Ball L, Sutherasan Y, Caratto V, Sanguineti E, Marsili M, Raimondo P, Ferretti M, Kacmarek RM, Pelosi P. Effects of Nebulizer Position, Gas Flow, and CPAP on Aerosol Bronchodilator Delivery: An In Vitro Study. Respir Care. 2016 Mar;61(3):263-8. doi: 10.4187/respcare.04275. Epub 2015 Nov 17. PMID: 26577198.

5. If bronchodilation is the therapeutic target, a PEEP implementation during nebulization does not improve the bronchodilating effect as the tracheobronchial tree is

open with or without a PEEP. If bacterial killing in the infected lung parenchyma is the therapeutic target, then a PEEP implementation during nebulization improves aerosol delivery of aerosolized antibiotics by recruiting the infected lung and is potentially beneficial in patients with ventilator-associated pneumonia. Although there is no formal demonstration of the latter statement, the majority of experts and academic societies recommend to a use PEEP during the nebulization of antibiotics to treat ventilator-associated pneumonia. As a consequence, I suggest to follow these recommendations based on the corresponding references (above) and to provide this information in the recommendation (outlined in yellow). I also suggest to recommend experimental studies based on lung microdialysis to demonstrate that using a PEEP during the nebulization period increases lung deposition of aerosolized antibiotics.

6. This should be merged with 1.27, 1.28, 1.29, 1.30.

Round 2

**Recommendations 1.31.1**	It is not recommended to change the positive end-expiratory pressure (PEEP) for the sole purpose of improving aerosol delivery efficiency.
**Likert score of 1-9**	□1 □2 □3 □4 □5 □6 □7 □8 □9<br>1= absolutely disagree, 9 = absolutely agree
**Comments**	

[Additional recommendations based on reviewer suggestion]

**Recommendations 1.31.2**	**When delivering inhaled antibiotics for invasively ventilated patients, it is recommended to set a positive end-expiratory pressure (PEEP) at 5-10 cmH$_2$O.**
**Likert score of 1-9**	□1 □2 □3 □4 □5 □6 □7 □8 □9<br>1= absolutely disagree, 9 = absolutely agree
**Comments**	

Table1.31.1 Studies of change of PEEP

<table>
<tr><th>Author, year</th><th>Study Type</th><th>Population</th><th>Inhaled medication</th><th>Nebulizer And Position</th><th colspan="7">Comparison and Finding/Aerosol deposition</th></tr>
<tr><td rowspan="2">Williams,1999[1]</td><td rowspan="2">In vitro</td><td rowspan="2">Adult</td><td rowspan="2">Saline 0.9%</td><td rowspan="2">Ultrasonic nebulizer, inspiratory limb</td><td colspan="7">MV:7.2L/min(12*0.6L)， I:E 1:2, PEEP: 0 vs 5 vs 10</td></tr>
<tr><td colspan="7">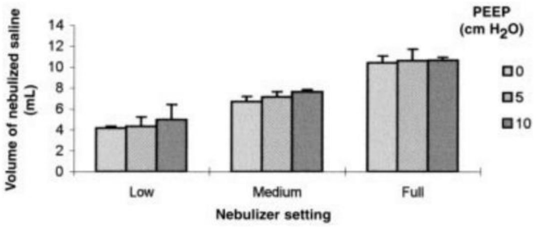

Fig. 2. The effect of positive end expiratory pressure upon the function of the DeVilbiss Ultra-Neb 2000 to nebulize 0.9% saline over a period of 5 min.
P> 0.05</td></tr>
<tr><td rowspan="7">Guérin, 2005[2]</td><td rowspan="7">In vivo</td><td rowspan="7">Adult: 10 COPD patients</td><td rowspan="7">Fenoterol</td><td rowspan="7">Inspiratory synchronized JN</td><td colspan="2"></td><td colspan="2">ZEEP</td><td colspan="2">PEEPe (80% PEEPt)</td><td>P</td></tr>
<tr><td colspan="2">Pre PEEPt</td><td colspan="2">8±3 cmH_2O</td><td colspan="2">9±3 cmH_2O</td><td rowspan="2"><0.05</td></tr>
<tr><td colspan="2">60min PEEPt</td><td colspan="2">6±3 cmH_2O</td><td colspan="2">8±3 cmH_2O</td></tr>
<tr><td colspan="2">Pre △FRC</td><td colspan="2">0.61±0.34 L</td><td colspan="2">0.62±0.34 L</td><td rowspan="2"><0.05</td></tr>
<tr><td colspan="2">60min △FRC</td><td colspan="2">0.43±0.32 L</td><td colspan="2">0.62±0.37 L</td></tr>
<tr><td colspan="2">Pre Rrs</td><td colspan="2">26±7 $cmH_2O/L/s$</td><td colspan="2">26±9 $cmH_2O/L/s$</td><td rowspan="2">> 0.05</td></tr>
<tr><td colspan="2">60min Rrs</td><td colspan="2">23±6 $cmH_2O/L/s$</td><td colspan="2">25±9 $cmH_2O/L/s$</td></tr>
<tr><td rowspan="4">Vecellio, 2005[3]</td><td rowspan="4">In vitro</td><td rowspan="4">Adult</td><td rowspan="4">Terbutaline</td><td rowspan="4">Continuous JN, inspiratory line</td><td rowspan="4">VT 600 ml RR 12/min, Ti/Ttot 0.5</td><td rowspan="2">10 mg/4 ml of terbutaline</td><td>PEEP 0 cmH_2O</td><td>PEEP 2 cmH_2O</td><td>PEEP 4 cmH_2O</td><td>PEEP 6 cmH_2O</td><td>P</td></tr>
<tr><td>44%</td><td>44%</td><td>44%</td><td>46%</td><td>=0.66</td></tr>
<tr><td rowspan="2">20 mg/8 ml of terbutaline</td><td>PEEP 0 cmH_2O</td><td>PEEP 2 cmH_2O</td><td>PEEP 4 cmH_2O</td><td>PEEP 6 cmH_2O</td><td>P</td></tr>
<tr><td>52%</td><td>50%</td><td>49%</td><td>52%</td><td>=0.22</td></tr>
</table>

JN, jet nebulizer; MV, minute ventilation; I:E, inspiration to expiration ratio; PEEP, positive end-expiratory pressure;COPD, chronic obstructive pulmonary disease; ZEEP,zero end-expiratory pressure;PEEPe,external positive end-expiratory pressure;PEEPt, total positive end-expiratory pressure; △FRC, change in end-expiratory lung volume; Rrs, inspiratory resistance;VT, tidal volume;RR, respiratory rates;Ti,inspiratory time; VMD, volume median diameter.

1. Williams L, Fletcher GC, Daniel M, Kinsella J. A simple in vitro method for the evaluation of an ultrasonic nebulizer for drug delivery to intubated, ventilated patients and the effect of nebulizer and ventilator settings on the uptake of fluid from the nebulizer chamber. Eur J Anaesthesiol. 1999 Jul;16(7):479~84.
2. Guérin C, Durand PG, Pereira C, Richard JC, Poupelin JC, Lemasson S, Badet M, Philit F, Vecellio L, Chantrel G. Effects of inhaled fenoterol and positive end-expiratory pressure on the respiratory mechanics of patients with chronic obstructive pulmonary disease. Can Respir J. 2005 Sep;12(6):329-35.
3. Vecellio L, Guérin C, Grimbert D, De Monte M, Diot P. In vitro study and semiempirical model for aerosol delivery control during mechanical ventilation. Intensive Care Med. 2005 Jun;31(6):871-6.

1.31.2 Recommendations for nebulized antibiotics in the consensuses and reviews.

Author, year	Nebulizer	Position	Mode	Vt	Inspiratory flow	I: E	RR	Bias flow	End-inspiratory pause	PEEP	Others
Rello J,2017	VMN	10–15 cm before the Y piece on the inspiratory limb	Volume-controlled	8ml/kg	Constant inspiratory flow	≤ 50%	12-15 bpm	/	20%	5-10 cmH_2O	• Avoid sharp angles and rough inner surfaces in circuit<br>• Avoid asynchronies and triggering<br>• Increase level of sedation if necessary<br>• Remove HME<br>• Turn off HH<br>• Change expiratory filter after each nebulization
Rouby JJ, 2020	VMN	10–15 cm before the Y piece on the inspiratory limb	Volume-controlled	8ml/kg	Limiting inspiratory flow velocity	1: 2	12-15 bpm	/	20%	5-10 cmH_2O	• Remove HME<br>• Turn off HH
Monsel A, 2021	VMN	15 cm before the Y piece on the inspiratory limb	Volume-controlled	8ml/kg	Constant inspiratory flow	1: 1	12-15 bpm	Absence or minimum (2 l/min)	20%	/	• Smooth angles and inner surface tube<br>• Increase level of sedation if necessary<br>• Remove HME<br>• Turn off HH

VMN vibrating mesh nebulizer, PDDS pulmonary drug delivery system, JN jet nebulizer, UN ultrasonic nebulizer, MV minute ventilation, Vt tidal volume, I: E inspiratory to

expiratory ratio, RR respiratory rate, PEEP positive end-expiration pressure, HME heat and moisture exchanger, HH heated humidifiers,

1. Rello J, Rouby JJ, Sole-Lleonart C, Chastre J, Blot S, Luyt CE, Riera J, Vos MC, Monsel A, Dhanani J, Roberts JA. Key considerations on nebulization of antimicrobial agents to mechanically ventilated patients. Clin Microbiol Infect. 2017 Sep;23(9):640-646.
2. Rouby JJ, Sole-Lleonart C, Rello J; European Investigators Network for Nebulized Antibiotics in Ventilator-associated Pneumonia. Ventilator-associated pneumonia caused by multidrug-resistant Gram-negative bacteria: understanding nebulization of aminoglycosides and colistin. Intensive Care Med. 2020 Apr;46(4):766-770. Rouby JJ, Sole-Lleonart C, Rello J; European Investigators Network for Nebulized Antibiotics in Ventilator-associated Pneumonia. Ventilator-associated pneumonia caused by multidrug-resistant Gram-negative bacteria: understanding nebulization of aminoglycosides and colistin. Intensive Care Med. 2020 Apr;46(4):766-770.
3. Monsel A, Torres A, Zhu Y, Pugin J, Rello J, Rouby JJ; European Investigators Network for Nebulized Antibiotics in Ventilator-associated Pneumonia (ENAVAP). Nebulized antibiotics for ventilator-associated pneumonia: methodological framework for future multicenter randomized controlled trials. Curr Opin Infect Dis. 2021 Apr 1;34(2):156-168.

Section 1: Aerosol Delivery via Invasive Ventilation for Adult Patients

Recommendation 1.32： With nebulizer placed proximal to patient, higher bias flow is associated with lower aerosol delivery efficiency. With nebulizer placed proximal to ventilator, adding bias flow up to 5 L/min improves delivery. It is recommended to set bias flow up to 5 L/min when nebulizer is placed proximal to ventilator.

Distribution of voting scores

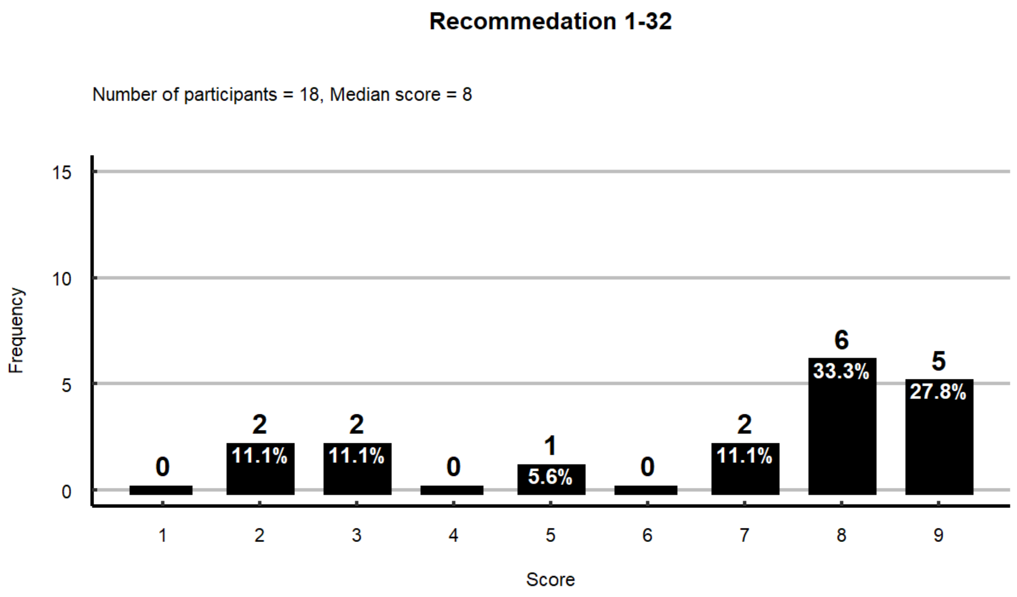

Comments of voting panelists

1. • Inspiratory limb 10 cm proximal Y piece
- Diluted in 10mL saline
- Removal HME
- Volume controlled, constant inspiratory flow
- RR 12x' 50% I:E ratio Tidal V 8ml/Kg
- End-inspiratory pause 20% duty cicle
- Delivered over 60 minutes

Expired aerosolized particles collected in a filter.

2. Limited in vitro data suggested that bias flow may influence aerosol delivery (depending on the type of nebulizer and its position)

3. The advantage of using a bias flow rate of up to 5L/min remains controversial. This concept is based on a single in vitro reference, and the increase in inhaled dose was only seen when the VMN was used. Therefore, this recommendation cannot be generalized to all types of nebulizers.

4. The greatest dose delivered with bias flow of 0 L/min for JN and 2 L/min for VMN when the nebulizer was placed proximal to ventilator.

5. Evidence weak. No human data. Not practical as this is not a variable that can be changed on many contemporary ventilators.

6. Also after Y – again we should aslo consider possible different effects with different ventilatory settings and assisted vs controlled ventilation.

7. Nebulization of antibiotic require positioning the nebulizer proximal to patients (see recommendation 1.4 that should be split into two different recommendations), whereas nebulization of bronchodilators can be indifferently administered proximal to ventilator or patient. When placed close to the ventilator, nebulizers are much less efficient in aerosol delivery of bronchodilators and antibiotics than when placed close to the Y piece. I suggest to indirectly provide this information in the recommendation (outlined in yellow).

8. Need in vivo investigations to corroborate the findings.

9. Effects are different for jet and VM nebulizers.

Round 2

**Recommendations 1.32**	With nebulizer placed proximal to patient, higher bias flow is associated with lower aerosol delivery efficiency. With nebulizer placed proximal to ventilator, adding bias flow up to 5 L/min improves delivery. It is recommended to set bias flow up to 5 L/min when nebulizer is placed proximal to ventilator.
**Likert score of 1-9**	□1 □2 □3 □4 □5 □6 □7 □8 □9<br>1= absolutely disagree, 9 = absolutely agree
**Comments**	

Section 2: Aerosol Delivery via Non-invasive Ventilation for Adult Patients

Recommendation 2.1：Placing the nebulizer in-line with noninvasive ventilation has similar or higher aerosol delivery efficiency than using the nebulizer with a mask or mouthpiece. Interrupting or discontinuing noninvasive ventilation to administer aerosol via a mask or mouthpiece is not recommended.

Distribution of voting scores

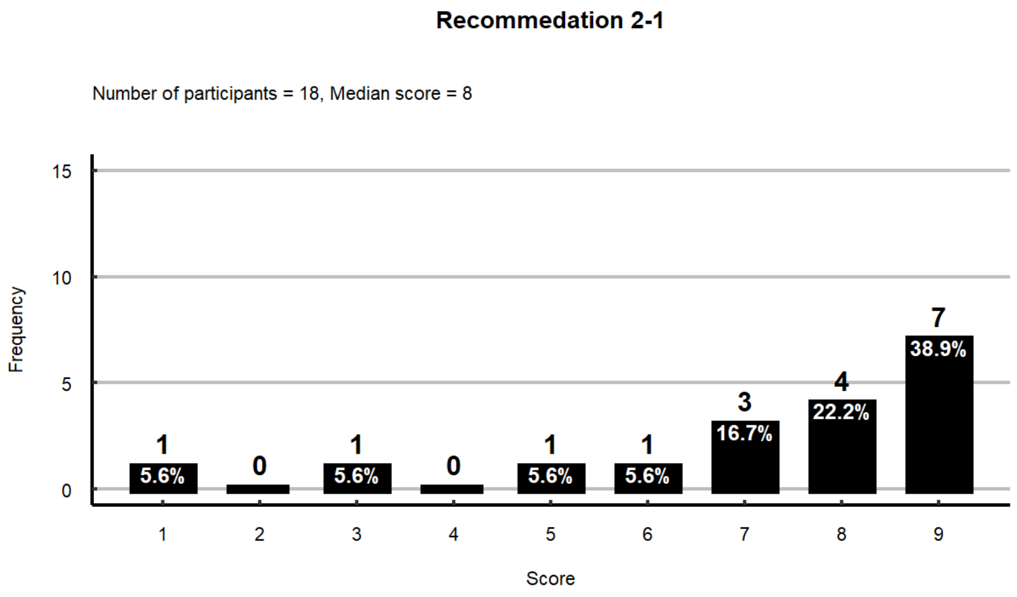

Comments of voting panelists

1. Restricted to asthma.

2. Limited data on healthy volunteers suggested better drug delivery when using the nebulizer with a mask or mouthpiece. However, limited clinical studies reported that inhaled bronchodilator response may be improved with the use of noninvasive ventilation in asthma. If patient can withstand a temporary discontinuation, it is preferable to interrupt noninvasive ventilation to administer aerosol via a mask or mouthpiece.

3. Placing the nebulizer in-line with noninvasive ventilation has sometimes similar lower aerosol delivery efficiency than using the nebulizer with a mask or mouthpiece. But I agree that Interrupting or discontinuing noninvasive ventilation to administer aerosol via a mask or mouthpiece is not recommended for patient safety.

4. I wonder if it can be said that ""placing the nebulizer in line with non-invasive ventilation has similar or better aerosol delivery efficiency than using the nebulizer with a mask or mouthpiece"". Because according to the references 1, 3 and 6 (3 and6 are in

vivo studies), the inhaled mass or lung deposition is higher in the control group than in the NIV group.

In asthma patients, in the in vivo studies, it remains unclear whether the improvement in lung function when nebulization was delivered with NIV was related solely to the effect of NIV or because in-line nebulization induced an additive or synergistic effect. In the reference 8 (Galindo-Filho), no difference in aerosol lung deposion was found between the control and the experimental groups.

The 2017 combined European Respiratory Society and American Thoracic Society clinical practice guidelines on NIV in acute respiratory failure were unable to make a recommendation for or against the use of NIV in acute asthma, in light of a growing number of clinical studies with positive clinical outcomes, widespread adoption, and accumulating observational evidence of the use of NIV to treat acute asthma, it seems reasonable to deliver aerosols during NIV. I suggest rewriting with caution the first sentence of the recommendation.

5. Results in the included studies are contradictory (for example, Calvert's study showed higher deposition with BiPAP, while the other three studies showed lower deposition). Moreover, NIV should not be used 24*7 non-stop, thus if the aerosol delivery is short-term and intermittent, discontinuing NIV to deliver aerosol is also feasible.

6. This section is target to adult patients, yet reference 9 is studied with children age 6-21 yrs.

7. Potential for harm with removing NIV. But need more human data.

8. Change is needed if patient is unable to tolerate NIV and nebulizer.

9. Most studies show a decrease in lung deposition when NIV is used. The study by Laube et al included a PEP device not NIV. I think it should be removed.

Round 2

**Recommendations 2.1**	Placing the nebulizer in-line with noninvasive ventilation has similar or higher aerosol delivery efficiency than using the nebulizer with a mask or mouthpiece. Interrupting or discontinuing noninvasive ventilation to administer aerosol via a mask or mouthpiece is not recommended.
**Likert score of 1-9**	□1 □2 □3 □4 □5 □6 □7 □8 □9

	1= absolutely disagree, 9 = absolutely agree
**Comments**	

Section 2: Aerosol Delivery via Non-invasive Ventilation for Adult Patients

Recommendation 2.2：During noninvasive ventilation using single limb circuit, placing metered dose inhaler with spacer between exhalation valve and mask, with actuation at the beginning of inspiration is recommended. There is no recommendation on the placement orientation (towards or away from patient) of the spacer.

Distribution of voting scores

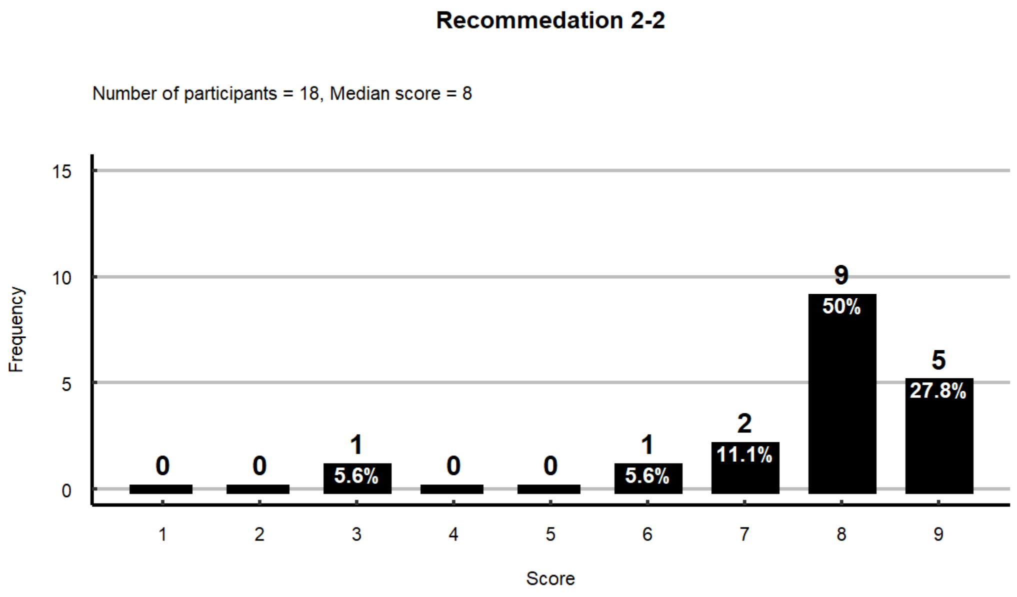

Comments of voting panelists

1. Limited studies reported non significant data regarding the placement orientation.
2. I did not see the comparisons of different placements of MDI and spacer.
3. Agree, but evidence weak.
4. The effects depends also on the position of nebulizer according to the leak valve – the best is to use a non vented mask and the nebulizer after the leak valve – see literature below.See our studies: Sutherasan Y, Ball L, Raimondo P, Caratto V, Sanguineti E, Costantino F, Ferretti M, Kacmarek RM, Pelosi P. Effects of ventilator settings, nebulizer and exhalation port position on albuterol delivery during non-invasive ventilation: an in-vitro study. BMC Pulm Med. 2017 Jan 10;17(1):9. doi: 10.1186/s12890-016-0347-5. PMID: 28068958; PMCID: PMC5223303.

Ball L, Sutherasan Y, Caratto V, Sanguineti E, Marsili M, Raimondo P, Ferretti M, Kacmarek RM, Pelosi P. Effects of Nebulizer Position, Gas Flow, and CPAP on Aerosol Bronchodilator Delivery: An In Vitro Study. Respir Care. 2016 Mar;61(3):263-8. doi: 10.4187/respcare.04275. Epub 2015 Nov 17. PMID: 26577198.

5. This is a compound statement. I think the second part is not needed. The statement could say actuation at the beginning of inspiration with the spray directed towards the patient is recommended

Round 2

**Recommendations 2.2**	During noninvasive ventilation using single limb circuit, placing metered dose inhaler with spacer between exhalation valve and mask, with actuation at the beginning of inspiration is recommended. There is no recommendation on the placement orientation (towards or away from patient) of the spacer.
**Likert score of 1-9**	□1 □2 □3 □4 □5 □6 □7 □8 □9<br>1= absolutely disagree, 9 = absolutely agree
**Comments**	

Section 2: Aerosol Delivery via Non-invasive Ventilation for Adult Patients

Recommendation 2.3： When placing the continuous nebulizer in-line with noninvasive ventilation, vibrating mesh nebulizer is more efficient in aerosol delivery than jet nebulizer. When available, vibrating mesh nebulizer is recommended over jet nebulizer.

Distribution of voting scores

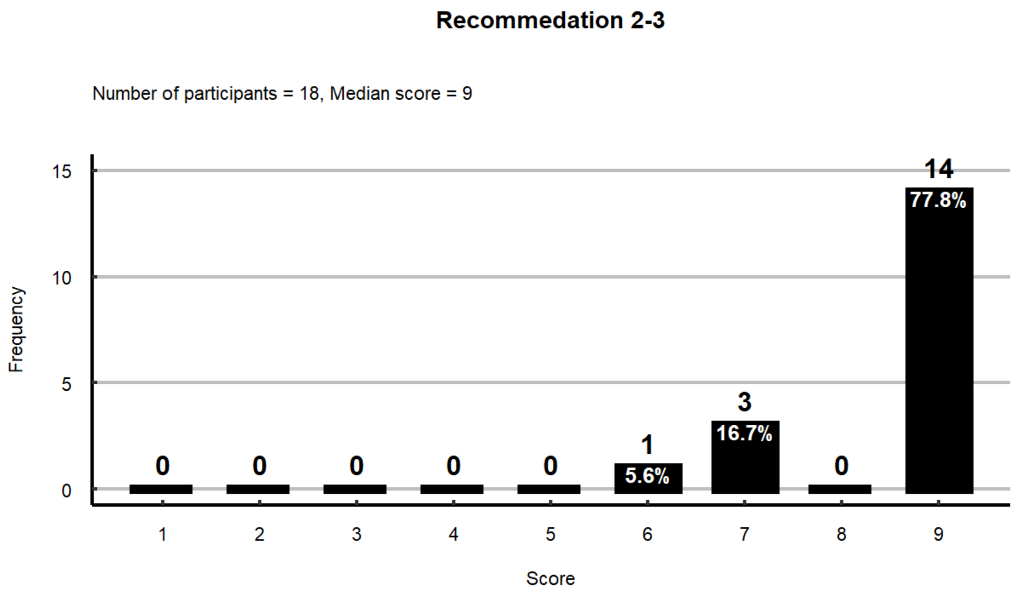

Comments of voting panelists

1. Vibrating mesh nebulizers generate higher inhaled dose than jet nebulizers. Additionally, the absence of influence on ventilator settings represents an important benefit over jet nebulizer.

2. Not clear if it is a continuous or synchronized jet neb?

3. Seem comment above about confusing meaning of "continuous." Evidence is lacking for better clinical outcomes. More drug delivery does not necessarily mean better clinical outcomes, which has not been studied.

4. If mesh is placed at the ventilator or Y – not the same if located at 15 cm from Y or before Y (also HHWH off or on might affect the efficiency in these conditions) – in other words the statement is correct pending several factors described below.

5. Agree with the comment about efficiency. However, more is not necessarily better. Cost is not a variable being consider. The difference in efficiency varies with position in the circuit.

Round 2

**Recommendations 2.3**	When placing the continuous nebulizer in-line with noninvasive ventilation, vibrating mesh nebulizer is more efficient in aerosol delivery than jet nebulizer. When available, vibrating mesh nebulizer is recommended over jet nebulizer.
**Likert score of 1-9**	□1 □2 □3 □4 □5 □6 □7 □8 □9<br>1= absolutely disagree, 9 = absolutely agree
**Comments**	

Additional references：

1. Mohsen M , Elberry A A , Eldin A S , et al. Effects of Heat and Humidification on Aerosol Delivery during Auto-CPAP noninvasive Ventilation.Arch Pulmonology Respir Care 3(1):011-015.
2. Galindo-Filho VC, Alcoforado L, Rattes C, Paiva DN, et al. A mesh nebulizer is more effective than jet nebulizer to nebulize bronchodilators during non-invasive ventilationof subjects with COPD: A randomized controlled trial with radiolabeled aerosols[J]. Respiratory Medicine, 2019, 153:60-67.

Section 2: Aerosol Delivery via Non-invasive Ventilation for Adult Patients

Recommendation 2.4： During noninvasive ventilation using single limb circuit, the continuous nebulizer is recommended to be placed between the exhalation valve and the mask.

Distribution of voting scores

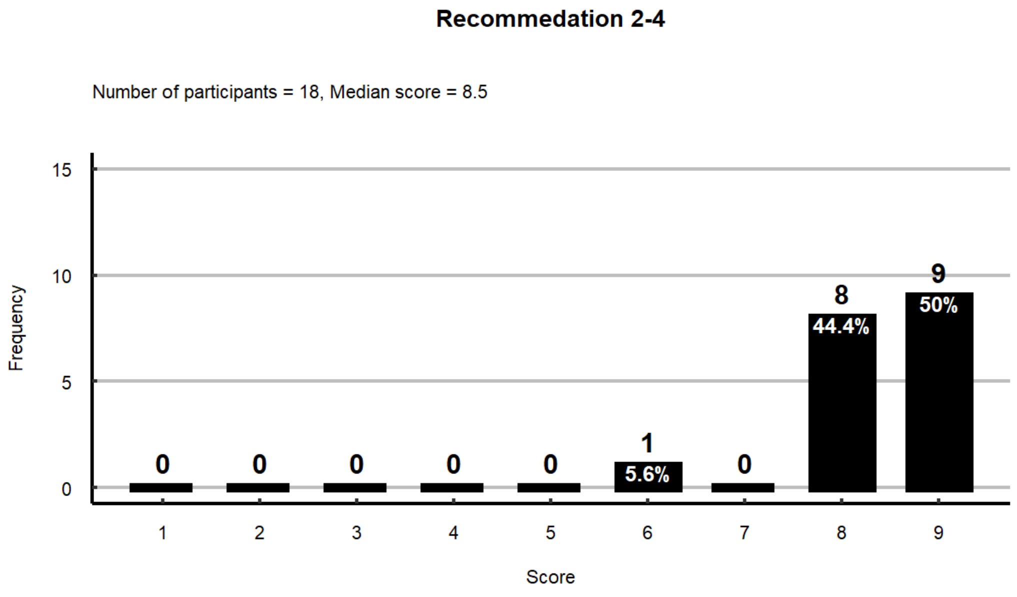

Comments of voting panelists

1. Limited data reported that the type of exhalation valve and ventilator settings may influence aerosol delivery of continuous nebulizer and hence, its optimum position.

2. In the whole section 2: in vitro studies are of very very limited value to inform the question. Indeed most patients on NIV are on a pressure regulated mode, thus the patient determines the flow and volume of each breath which are the main determinants of aerosol delivery performance.. this cannont be simulated by most bench studies using simple test-lungs.

3. No human data. Note again confusion with the use of "continuous"

4. See our studies: Sutherasan Y, Ball L, Raimondo P, Caratto V, Sanguineti E, Costantino F, Ferretti M, Kacmarek RM, Pelosi P. Effects of ventilator settings, nebulizer and exhalation port position on albuterol delivery during non-invasive ventilation: an in-vitro study. BMC Pulm Med. 2017 Jan 10;17(1):9. doi: 10.1186/s12890-016-0347-5. PMID: 28068958; PMCID: PMC5223303.

Ball L, Sutherasan Y, Caratto V, Sanguineti E, Marsili M, Raimondo P, Ferretti M, Kacmarek RM, Pelosi P. Effects of Nebulizer Position, Gas Flow, and CPAP on

Aerosol Bronchodilator Delivery: An In Vitro Study. Respir Care. 2016 Mar;61(3):263-8. doi: 10.4187/respcare.04275. Epub 2015 Nov 17. PMID: 26577198.

5. Need additional in vivo data.

Round 2

**Recommendations 2.4**	During noninvasive ventilation using single limb circuit, the continuous nebulizer is recommended to be placed between the exhalation valve and the mask.
**Likert score of 1-9**	□1 □2 □3 □4 □5 □6 □7 □8 □9<br>1= absolutely disagree, 9 = absolutely agree
**Comments**	

Additional references：

1. Ball L, Sutherasan Y, et al. Effects of Nebulizer Position, Gas Flow, and CPAP on Aerosol Bronchodilator Delivery: An In Vitro Study[J]. Respiratory Care, 2015, 61(3).

2. Sutherasan Y, Ball L, Raimondo P, et al . Effects of ventilator settings, nebulizer and exhalation port position on albuterol delivery during non-invasive ventilation: an in-vitro study. BMC Pulm Med. 2017 Jan 10;17(1):9.

Section 2: Aerosol Delivery via Non-invasive Ventilation for Adult Patients

Recommendation 2.5： During noninvasive ventilation using a single limb circuit, with the continuous nebulizer placed between mask and exhalation valve, there is no recommendation on the type of exhalation valve.

Distribution of voting scores

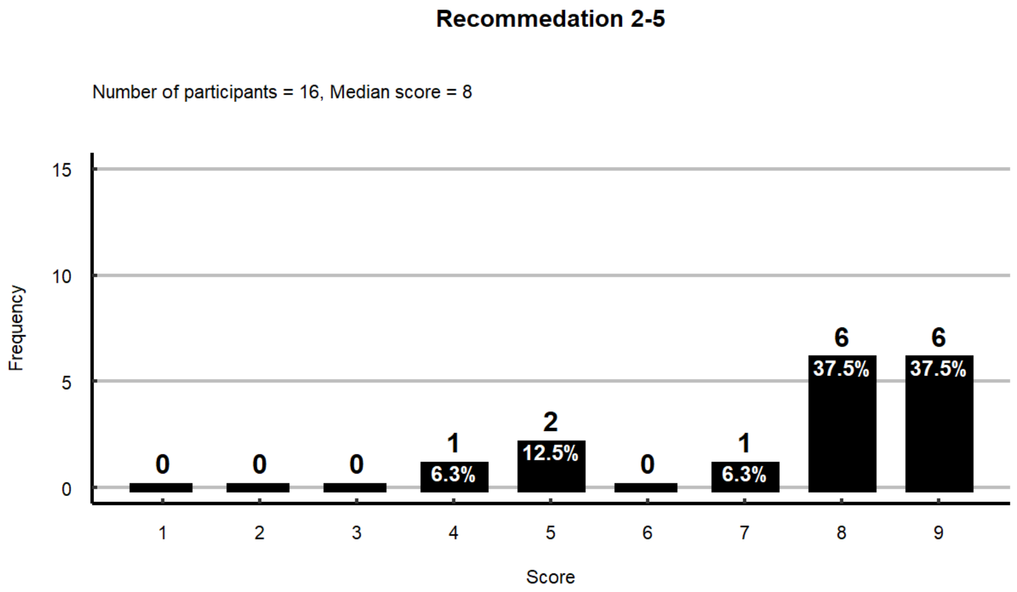

Comments of voting panelists

1. Limited data reported that the type of exhalation valve and ventilator settings may influence aerosol delivery of continuous jet nebulizer. There is no data with vibrating mesh nebulizer.

2. Only one exhalation value is used in the US market.

3. For this and previous recommendations, I think you mean "exhalation port," not "exhalation valve." There is not valve in passive circuits. Do you mean a single limb circuit with a passive exhalation port or a single limb circuit with an active exhalation valve?

4. In any case better with nonvented mask – to be discussed.

5. I don't have a specific score.

Round 2

**Recommendations 2.5**	During noninvasive ventilation using a single limb circuit, with the continuous nebulizer placed between mask and

	exhalation valve, there is no recommendation on the type of exhalation valve.
**Likert score of 1-9**	□1 □2 □3 □4 □5 □6 □7 □8 □9<br>1= absolutely disagree, 9 = absolutely agree
**Comments**	

Section 2: Aerosol Delivery via Non-invasive Ventilation for Adult Patients

Recommendation 2.6： During aerosol delivery via noninvasive ventilation, turning off the humidifier for the sole purpose of increasing aerosol delivery is not recommended.

Distribution of voting scores

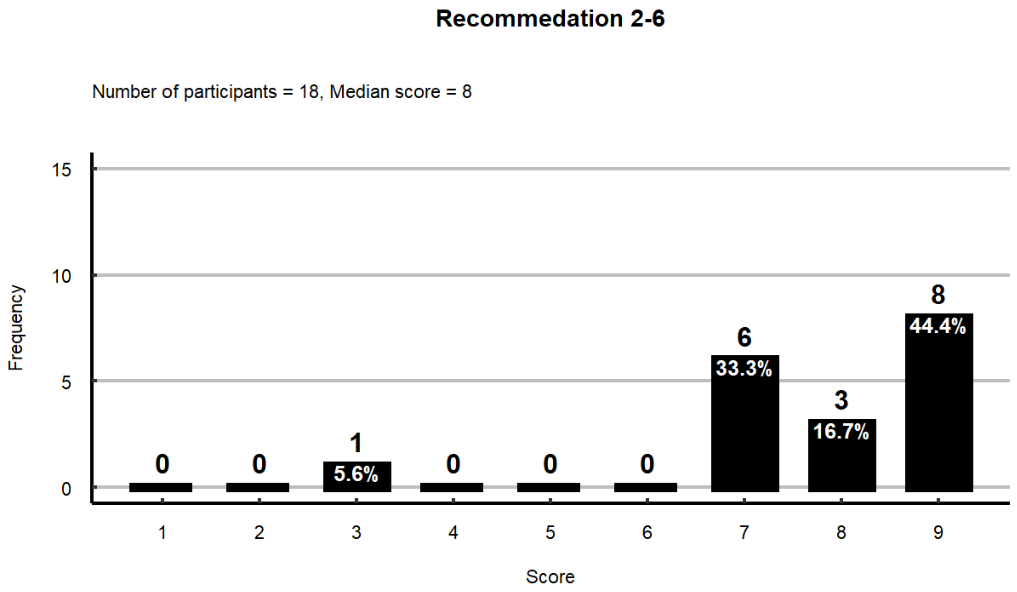

Comments of voting panelists

1. Different dutaions of administration should be explored.

2. Limited data suggested that there is no significant difference in aerosol delivery during NIV with and without humidification.

Moreover, turning off the humidifier may have undesirable effects (e.g. mucosal dryness, patient discomfort…).

3. Most in vitro and in vivo studies evaluating aerosol delivery during NIV have used a single-limb ventilation circuit. The effect of heat and humidification on the nebulized dose may be different if a dual-limb circuit is used.

4. Lack of data.

5. Humifiers should be removed if administering antibiotics.

6. Potential harm outweighs any benefit.

7. This question needs to be investigated further.

8. The statement should read: During aerosol delivery via noninvasive ventilation, turning off the humidifier does not improve aerosol delivery” the way is written implies that there is benefit but we should not try it.

Round 2

**Recommendations 2.6**	During aerosol delivery via noninvasive ventilation, turning off the humidifier for the sole purpose of increasing aerosol delivery is not recommended.
**Likert score of 1-9**	□1 □2 □3 □4 □5 □6 □7 □8 □9<br>1= absolutely disagree, 9 = absolutely agree
**Comments**	

Additional references：

1. Mohsen M , Elberry A A , Eldin A S , et al. Effects of Heat and Humidification on Aerosol Delivery during Auto-CPAP noninvasive Ventilation. Arch pulmonol Respir Care3(1) :011-015.

Section 2: Aerosol Delivery via Non-invasive Ventilation for Adult Patients

Recommendation 2.7：The aerosol delivery efficiency is less affected by the fill volume in the vibrating mesh nebulizer than the continuous jet nebulizer. For continuous jet nebulizers, more dilution is associated with greater aerosol delivery. Increasing fill volume for the sole purpose to improve aerosol delivery efficiency is not recommended.

Distribution of voting scores

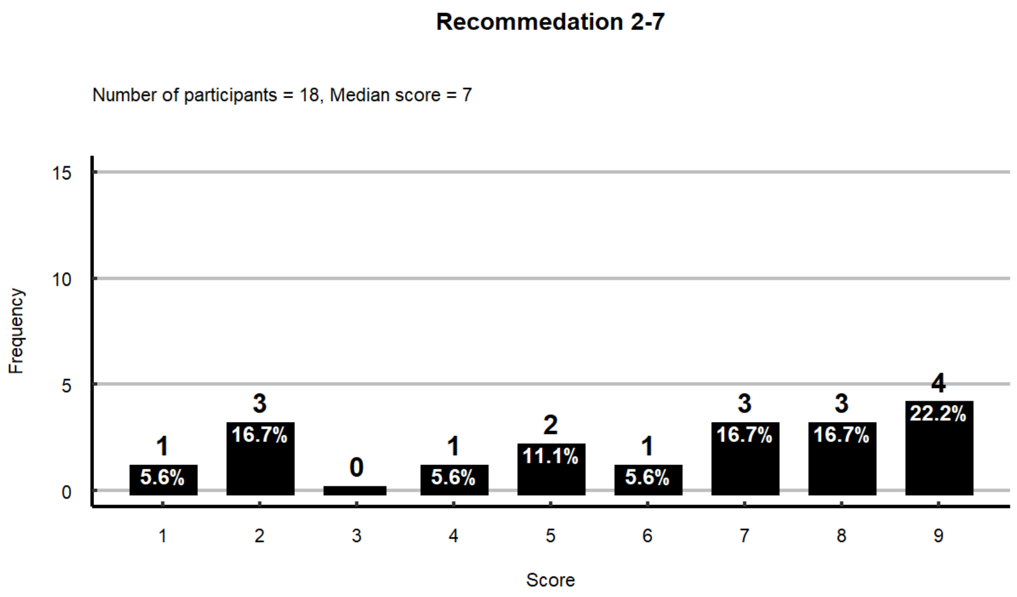

Comments of voting panelists

1. Why not?

2. Limited data reported that increasing fill volume improves aerosol delivery of jet nebulizers during NIV.

3. I agree for the first part of the recommendation "The aerosol delivery efficiency is less affected by the fill volume in the vibrating mesh nebulizer than the continuous jet nebulizer. For continuous jet nebulizers, more dilution is associated with greater aerosol delivery." But disagree for the second part : Increasing fill volume of jet nebulizer to improve aerosol delivery could be recommended.

4. a. Please explain the meaning of ""fill volume"" in the recommendation. According to these 4 references, the drug concentration decreased when the ""fill volume"" was increased. In this case, it is ""diluent volume"" rather than ""fill volume"".
b. I don't think there is enough clinical rationale to make such a recommendation as: ""Increasing the filling volume for the sole purpose of improving the efficiency of

aerosol delivery is not recommended"".

c. Since VMN is less affected by the “diluent volume”, this device could be a better choice.

5. I think this is mainly true for the VMN but not for CJN

6. See my answers to similar section for invasive ventilation

7. Evidence weak.

8. The study Hess 1996 should be removed because devices were not operated during NIV. The data sugest that loading volume should be increased .

Round 2

**Recommendations 2.7**	The aerosol delivery efficiency is less affected by the fill volume in the vibrating mesh nebulizer than the continuous jet nebulizer. For continuous jet nebulizers, more dilution is associated with greater aerosol delivery. Increasing fill volume for the sole purpose to improve aerosol delivery efficiency is not recommended.
**Likert score of 1-9**	□1 □2 □3 □4 □5 □6 □7 □8 □9<br>1= absolutely disagree, 9 = absolutely agree
**Comments**	

Section 2: Aerosol Delivery via Non-invasive Ventilation for Adult Patients

Recommendation 2.8： The aerosol delivery efficiency is similar between CPAP and BiPAP, changing the noninvasive ventilation mode for the sole purpose of increasing aerosol delivery is not recommended.

Distribution of voting scores

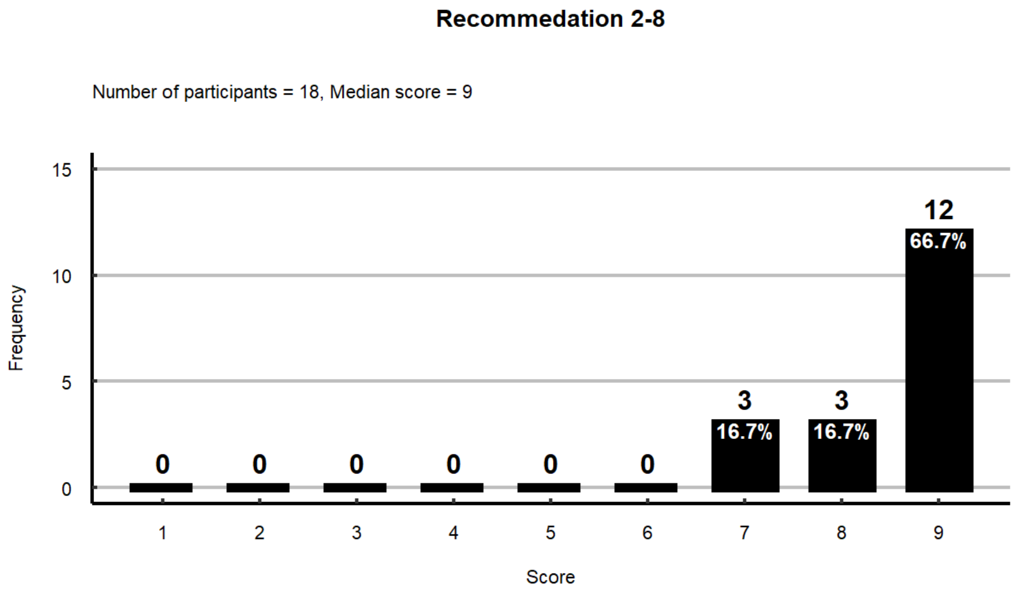

Comments of voting panelists

1. Considering the different indications (e.g hypoxemia for CPAP vs hypercapnia for BiPAP), physiological effects (e.g augment minute ventilation and reduce muscle loading for BiPAP) and the non significant results in the in vivo study, I would not recommend changing the noninvasive ventilation mode for the sole purpose of improving aerosol delivery efficiency.

2. I disagee BiPAP is better than CPAP this is only based on the in vitro data, the in vivo data below is NS.

3. Evidence weak, but high potential for harm.

4. It might be affected by settings (CPAP and BIPAP) – as well as interfaces and leak valves (mono or bi tube) – more complex.

Round 2

**Recommendations 2.8**	The aerosol delivery efficiency is similar between CPAP and BiPAP, changing the noninvasive ventilation mode for

	the sole purpose of increasing aerosol delivery is not recommended.
**Likert score of 1-9**	□1 □2 □3 □4 □5 □6 □7 □8 □9<br>1= absolutely disagree, 9 = absolutely agree
**Comments**	

Section 2: Aerosol Delivery via Non-invasive Ventilation for Adult Patients

Recommendation 2.9：When continuous nebulizer is placed between the mask and the exhalation valve during noninvasive ventilation with a single limb circuit, the aerosol delivery efficiency increases as IPAP increases or EPAP decreases. Changing the parameters for the sole purpose to improve aerosol delivery efficiency is not recommended.

Distribution of voting scores

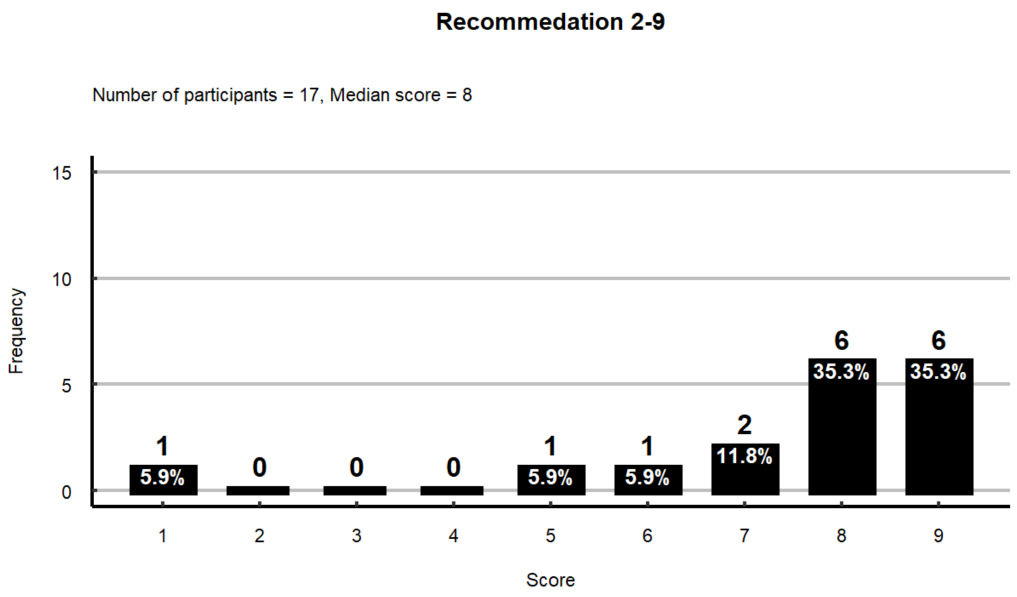

Comments of voting panelists

1. Increasing IPAP or decreasing EPAP may have adverse effects (e.g hyperventilation, hypoxemia, patient intolerance, unintentional leaks…).

2. No real in vivo data to draw definitive conclusions.

3. It could be useful to change the settings and it is not necessary deleterious as it is for mechanical ventilation.

4. Potential for harm. Note confusion definition of "harm" .

5. I agree more on the statement - Changing the parameters for the sole purpose to improve aerosol delivery efficiency is not recommended. – some more discussion about the other statement -When continuous nebulizer is placed between the mask and the exhalation valve during noninvasive ventilation with a single limb circuit, the aerosol delivery efficiency increases as IPAP increases or EPAP decreases – it depends on the device and interface as well as inspiratory effort of the patient and total minute ventilation – again interaction with bias flow and HHWH off or on has to be considered.

6. In my review article in JAMPDD 2012;25:63, I pointed out that the difference between IPAP and EPAP, i.e., the driving pressure appears to be important for aerosol delivery during NIV with the nebulizer placed closer to the patient.

Round 2

**Recommendations 2.9**	When continuous nebulizer is placed between the mask and the exhalation valve during noninvasive ventilation with a single limb circuit, the aerosol delivery efficiency increases as IPAP increases or EPAP decreases. Changing the parameters for the sole purpose to improve aerosol delivery efficiency is not recommended.
**Likert score of 1-9**	□1 □2 □3 □4 □5 □6 □7 □8 □9<br>1= absolutely disagree, 9 = absolutely agree
**Comments**	

Section 2: Aerosol Delivery via Non-invasive Ventilation for Adult Patients

Recommendation 2.10： When a continuous nebulizer is placed in-line with noninvasive ventilation, the aerosol delivery efficiency is higher with a non-vented mask than a vented mask. Aerosol administration with a vented mask is not recommended.

Distribution of voting scores

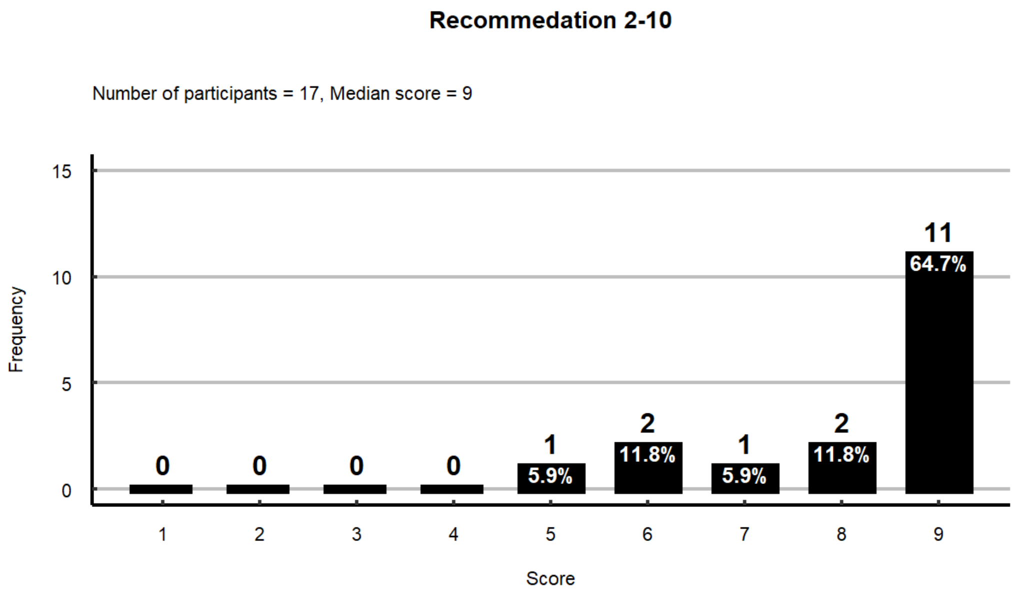

Comments of voting panelists

1. Limited data suggested better drug delivery when using a non-vented mask.

2. Not completely agree. It depends in terms of clinical efficiency . For example, in the case of vented mask, you can have a low drug deposition efficiency but enough drug mass to have a clinical effect with bronchodilatators. In other hands, it seems thrue that “non vented<vented”, but regarding the clinical efficiency and the large therapeutic window with bronchodilatator, it seems difficult to conclude without clinical study.

3. Taken together all those in vitro studies tend to show that the further away from the leak and the closer to the patient you place the nebulizer the better it is. On a non vented mask if the leak is close to the mask the same result occurs than with a vented mask.

4. Agree, but evidence weak. No human data. Do not know if clinical outcomes are affected. Note confusing definition of “continuous”.

5. See our papers quoted above to be added in the list of literature analyzed.

Round 2

**Recommendations 2.10**	When a continuous nebulizer is placed in-line with noninvasive ventilation, the aerosol delivery efficiency is higher with a non-vented mask than a vented mask. Aerosol administration with a vented mask is not recommended.
**Likert score of 1-9**	□1 □2 □3 □4 □5 □6 □7 □8 □9<br>1= absolutely disagree, 9 = absolutely agree
**Comments**	

Section 2: Aerosol Delivery via Non-invasive Ventilation for Adult Patients

Recommendation 2.11： When non-vented mask is used during noninvasive ventilation, the aerosol delivery efficiency with optimal position is similar with the single limb and dual limb circuits. There is no recommendation for the use of single versus dual limb circuits for aerosol delivery.

Distribution of voting scores

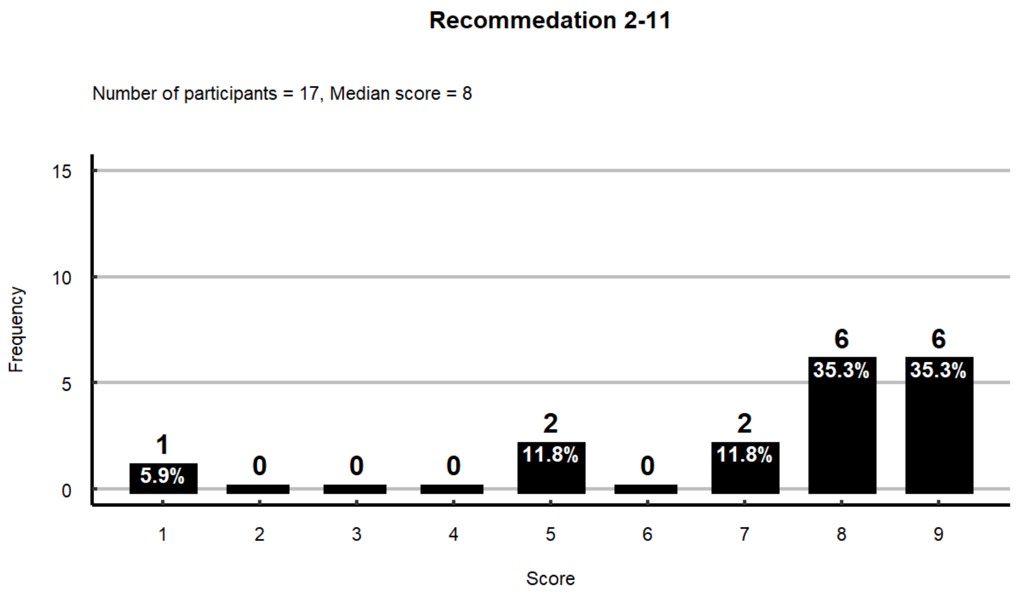

Comments of voting panelists

1. Limited data showed a trend in favor of dual limb circuits.
2. Nebulizer (or mesh nebulization) instead of aerosol delivery.
3. More evidence is needed.
4. Only in vitro studies: no conclusions can be drawn.
5. Almost all of the recommendations on NIV above are made on the basis of results obtained with a single-limb circuit. The results in this table show that the position "15 cm from Y-piece in the inspiratory limbs appears to have greater aerosol delivery efficiency when a dual-limb circuit is used. As I don’t have access to full text of this paper, I wonder if single and double limbs are really similar.
6. Very weak evidence.
7. Few studies with limited data – caution.
8. Studies under review cannot be included. The cut-off for inclusion occurred when you sent out the document for comments.

Round 2

**Recommendations 2.11**	When non-vented mask is used during noninvasive ventilation, the aerosol delivery efficiency with optimal position is similar with the single limb and dual limb circuits. There is no recommendation for the use of single versus dual limb circuits for aerosol delivery.
**Likert score of 1-9**	□1 □2 □3 □4 □5 □6 □7 □8 □9<br>1= absolutely disagree, 9 = absolutely agree
**Comments**	

Section 3: Aerosol Delivery via High-flow Nasal Cannula for Adult Patients

Recommendation 3.1：The aerosol delivery efficiency with a nebulizer via high-flow nasal cannula is similar to a mask or mouthpiece. Discontinuing high-flow nasal cannula treatment to administer nebulizer with a mask or mouthpiece is not recommended.

Distribution of voting scores

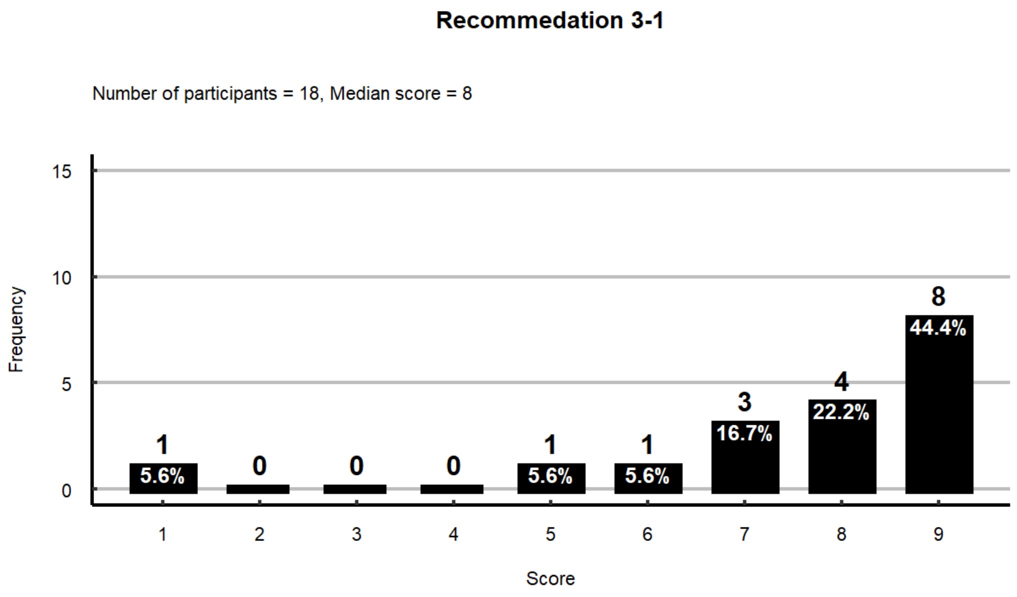

Comments of voting panelists

1. Totally logical and safe.

2. Limited clinical data on COPD reported no significant results (FEV1 improvement) between both administration techniques. However, limited in vitro data showed that inhaled dose may be improved when using nebulizer with a mask or mouthpiece. If patient can withstand a temporary discontinuation, it is preferable to interrupt high-flow nasal cannula to administer aerosol via a mask or mouthpiece.

3. The bronchodiltators aerosol delivery efficiency with a nebulizer via high-flow nasal cannula can be similar to a mask or mouthpiece for.

4. It is rather the bronchodilatatory effect than the delivery efficiency which is similar.

5. In vivo studies have used a flow rate ≤35 L/min. It is not yet known whether the use of high-flow oxygen ≥ 40L/min could produce the same effect.

6. The evidences listed are not sufficient support this recommendation. Refereces 1-4 demostrated the delivered dose was greater with VMN via HFNC. Results of References 5 illustrated JN/VMN via HFNT delivered signigicant lower dose than via

a mouthpiece or a mask.

7. The information in the table does not follow previous format. Please amend before it can be rated.

Round 2

**Recommendations 3.1**	The aerosol delivery efficiency with a nebulizer via high-flow nasal cannula is similar to **that with a nebulizer and** a mask or mouthpiece. Discontinuing high-flow nasal cannula treatment to administer nebulizer with a mask or mouthpiece is not recommended.
**Likert score of 1-9**	□1 □2 □3 □4 □5 □6 □7 □8 □9<br>1= absolutely disagree, 9 = absolutely agree
**Comments**	

Additional reference：

1. Beuvon C, Coudroy R, Bardin J, Marjanovic N, Rault C, Bironneau V, et al. β Agonist Delivery by High-Flow Nasal Cannula During COPD Exacerbation: A Prospective Physiological Study. Respir Care. 2021 Oct 26:respcare.09242. doi: 10.4187/respcare.09242.

Section 3: Aerosol Delivery via High-flow Nasal Cannula for Adult Patients

Recommendation 3.2：Placing a nebulizer with a mask or mouthpiece on a patient who is using concurrent high-flow nasal cannula treatment is not recommended.

Distribution of voting scores

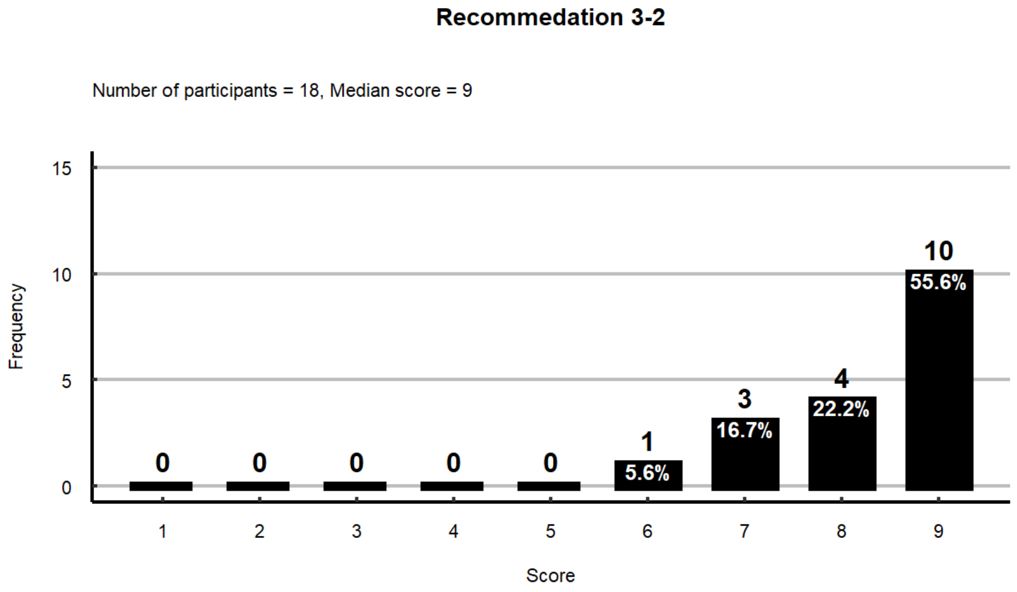

Comments of voting panelists

1. Logical recommendation as well.
2. Limited data suggested better drug delivery when vibrating mesh nebulizer is integrated with high-flow nasal cannula.
3. Agree based on sound principle, but evidence is weak.
4. Not many data available – caution.
5. Different from Recommendation 3.1?

Round 2

**Recommendations 3.2**	Placing a nebulizer with a mask or mouthpiece on a patient who is using concurrent high-flow nasal cannula treatment is not recommended.
**Likert score of 1-9**	□1 □2 □3 □4 □5 □6 □7 □8 □9<br>1= absolutely disagree, 9 = absolutely agree
**Comments**	

Section 3: Aerosol Delivery via High-flow Nasal Cannula for Adult Patients

Recommendation 3.3： During aerosol delivery via high-flow nasal cannula, vibrating mesh nebulizer is more efficient in aerosol delivery than jet nebulizer, with no influence on flows or fraction of inspired oxygen. Vibrating mesh nebulizer is recommended for trans-nasal aerosol delivery.

Distribution of voting scores

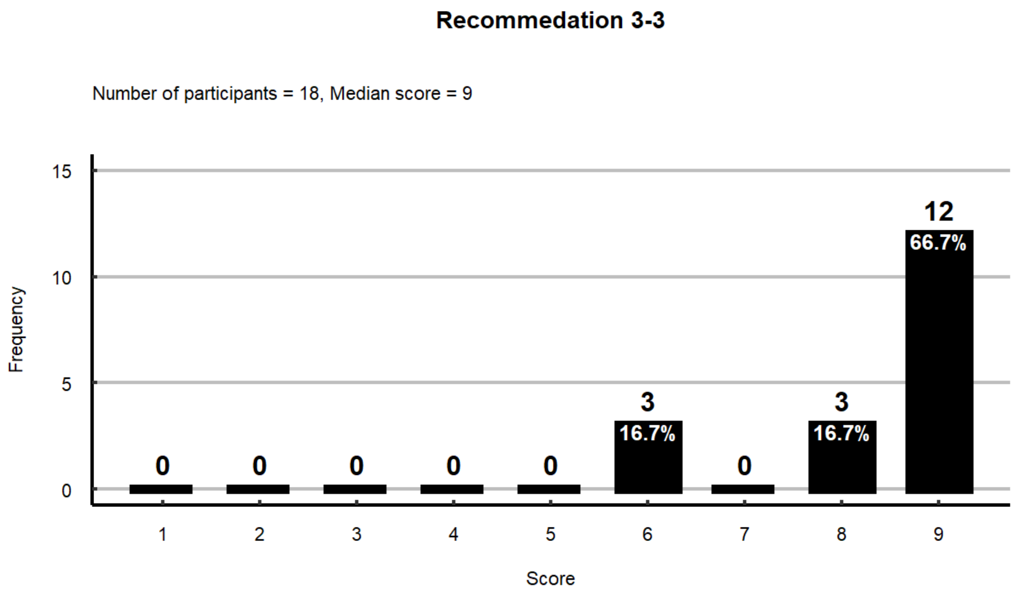

Comments of voting panelists

1. Agreed as well.

2. Vibrating mesh nebulizers generate higher inhaled dose than jet nebulizers. Additionally, the absence of influence on flows or fraction of inspired oxygen represents an important benefit.

3. Again “efficient” is misleading. Some bench data show similar efficiency of jet neb.

4. Agree in principle, but evidence weak.

5. Not many data available - caution – it depends also on the type of HFONT and patient’s drive.

6. We need data to show that these differences are clinically meaningful. The evidence is more directed toward efficiency of aerosol delivery.

7. Need to add “when available” to the recommendation.

Round 2

**Recommendations 3.3**	During aerosol delivery via high-flow nasal cannula, vibrating mesh nebulizer is more efficient in aerosol delivery than jet nebulizer, with no influence on flows or fraction of inspired oxygen. Vibrating mesh nebulizer is recommended for trans-nasal aerosol delivery.
**Likert score of 1-9**	□1 □2 □3 □4 □5 □6 □7 □8 □9<br>1= absolutely disagree, 9 = absolutely agree
**Comments**	

Section 3: Aerosol Delivery via High-flow Nasal Cannula for Adult Patients

Recommendation 3.4： Nebulizers are recommended to be placed at the inlet of humidifier at flows ≥ 10 L/min. At flows < 10 L/min, vibrating mesh nebulizer is recommended to be placed close to nasal cannula.

Distribution of voting scores

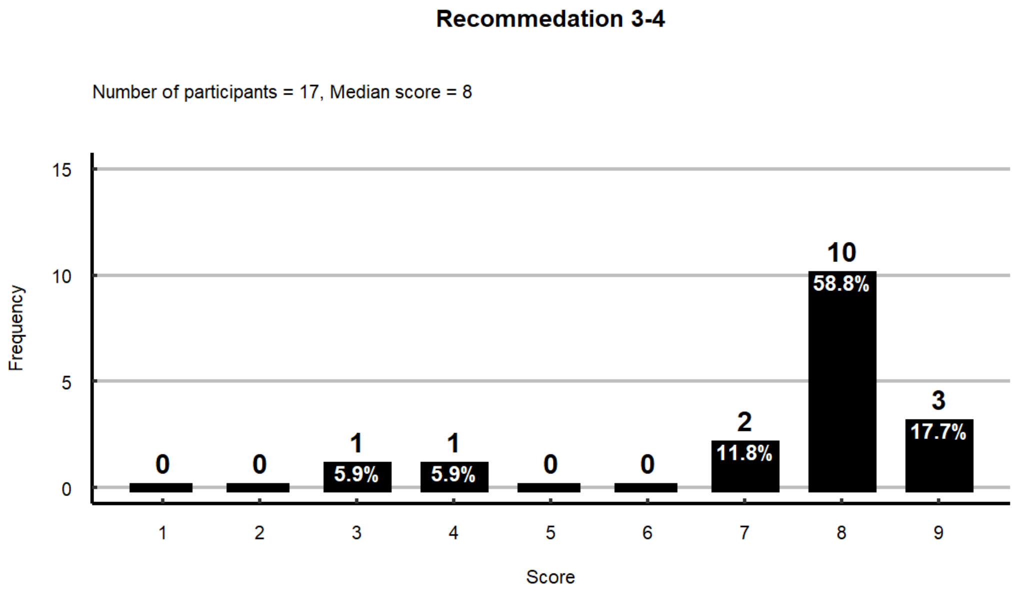

Comments of voting panelists

1. Limited data suggested better drug delivery using jet and vibrating mesh nebulizers at lower flows.

2. Flow below 10 L/Min is not high flow. Thus I would not make this recommendation.

3. To my opinion, a flow <10L/min cannot be considered as “high flow”.

4. I wonder if it is clinically relevant to make a recommendation for a gas flow rate < 10L/min during HFNC, as a flow rate below 10L/min is not considered high-flow oxygen therapy and it is rarely used in clinical situation. In addition, the recommendation on "< 10L/min" is based on only one reference. I suggest that this part of the recommendation ("< 10L/min) be deleted.

5. Very weak evidence.

6. Jet nebulized should be placed upstream from cannula (patient side); or before HHWH humidifier; mesh should be placed before HHWH during HFONT.

7. Please define what is “close to nasal cannula”. Again, it is unclear if these differences are clinically meaningful.

8. The recommendation should generalizable. I think it should always be at the

humidifier.

Round 2

**Recommendations 3.4**	Nebulizers are recommended to be placed at the inlet of humidifier at **HFNC** flows ≥ 10 L/min. At **HFNC** flows < 10 L/min, **a** vibrating mesh nebulizer is recommended to be placed close to nasal cannula.
**Likert score of 1-9**	□1 □2 □3 □4 □5 □6 □7 □8 □9<br>1= absolutely disagree, 9 = absolutely agree
**Comments**	

Section 3: Aerosol Delivery via High-flow Nasal Cannula for Adult Patients

Recommendation 3.5：When metered dose inhaler is placed in-line with high-flow nasal cannula, it is recommended to be used with a spacer and placed close to nasal cannula with plume emitted into the flow.

Distribution of voting scores

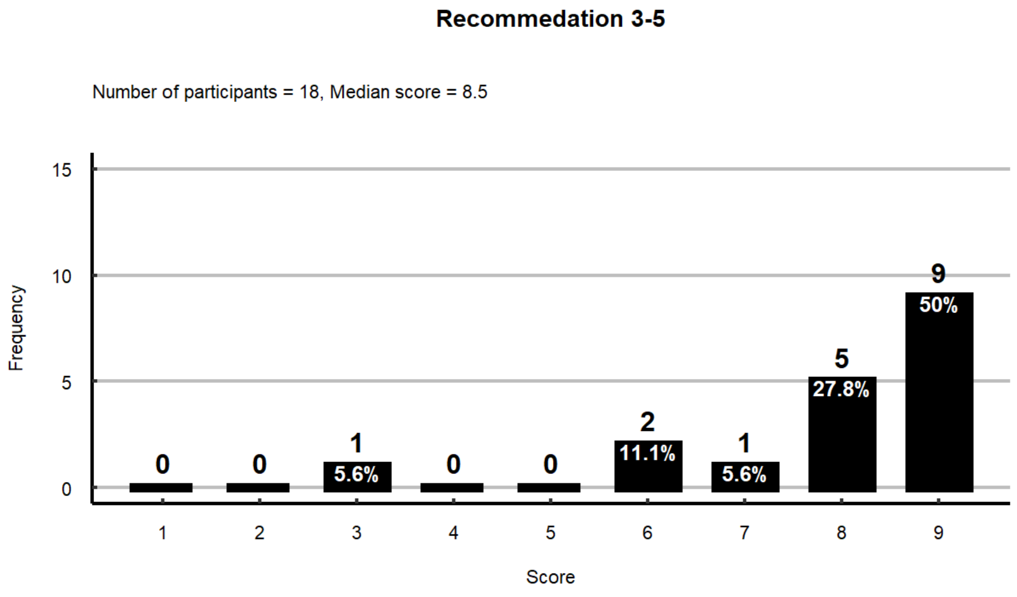

Comments of voting panelists

1. More evidence needed.

2. Limited data showed a trend in favor of spacer placed close to nasal canula and with the flow.

3. The ariticle stated "Data are indicated in micrograms (mean ± SD and inhalable mass %)" and the list belowe should be in micrograms.

4. Very weak evidence.

5. Minor few data – caution.

6. The statement could be modified to

When MDI is placed in-line with HFNC, the recommended technique for obtaining higher efficiency of aerosol delivery is to place the MDI and spacer close to the nasal cannula with the aerosol plume directed toward the patient.

Round 2

**Recommendations 3.5**	When metered dose inhaler is placed in-line with high-flow

	nasal cannula, it is recommended to be used with a spacer and placed close to nasal cannula with plume emitted into the flow.
**Likert score of 1-9**	□1 □2 □3 □4 □5 □6 □7 □8 □9<br>1= absolutely disagree, 9 = absolutely agree
**Comments**	

Section 3: Aerosol Delivery via High-flow Nasal Cannula for Adult Patients

Recommendation 3.6：To optimize aerosol delivery via high-flow nasal cannula, gas flow is recommended to be titrated below the patient's peak inspiratory flow if tolerated.

Distribution of voting scores

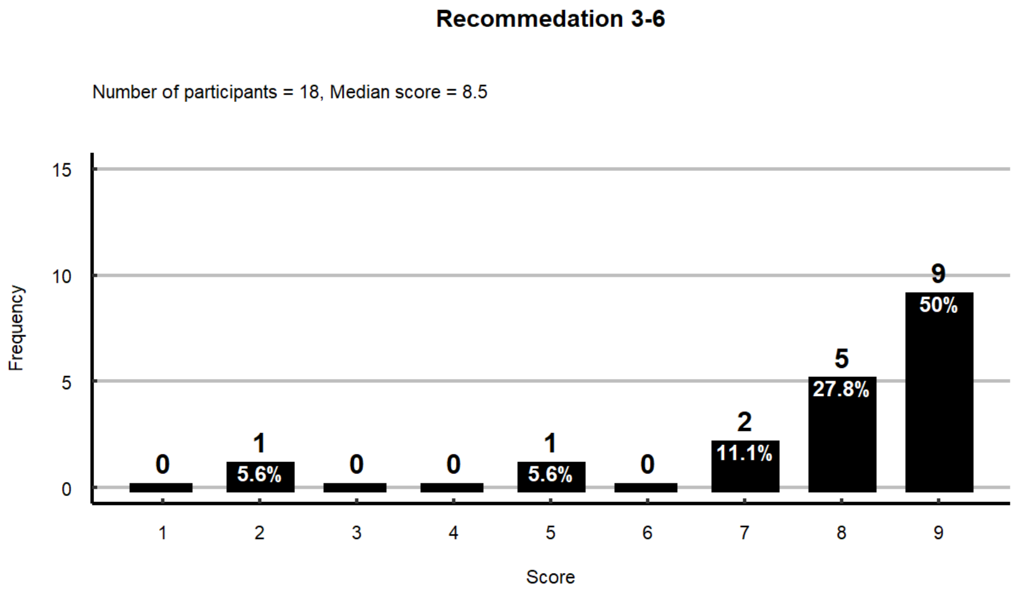

Comments of voting panelists

1. Limited data reported that limiting gas flow rates (if patient tolerated) have a beneficial effect on the inhaled dose due to decreased turbulence, inertial impaction and hence aerosol loss within the circuit.

2. If a patient cannot tolerate a low gas flow, particularly if the patient is hypoxic and requires high-flow oxygen, it is not known whether nebulization with high-flow oxygen ≥ 40L/min is effective.

3. Evidence lacking for differences in clinical outcomes.

4. Not easy in clinical practice – in most cases 40 L/min – in rare cases 60 L/min.Patient's drive is very important in this specific setting.

5. I understand the data. How do you do it in real life? If you can't do it then we should not make a recommendation.

Round 2

**Recommendations 3.6**	To optimize aerosol delivery via high-flow nasal cannula, gas flow is recommended to be titrated below the patient's

	peak inspiratory flow if tolerated.
**Likert score of 1-9**	□1 □2 □3 □4 □5 □6 □7 □8 □9<br>1= absolutely disagree, 9 = absolutely agree
**Comments**	

Additional references：

1. Li J, Gurnani PK, Roberts KM, Fink JB, Vines D. The Clinical Impact of Flow Titration on Epoprostenol Delivery via High Flow Nasal Cannula for ICU Patients with Pulmonary Hypertension or Right Ventricular Dysfunction: A Retrospective Cohort Comparison Study. J Clin Med, 2020, 9 (2), 464.
2. Li J, Chen Y, Ehrmann S, Wu J, Xie L, Fink JB. Bronchodilator Delivery via High-Flow Nasal Cannula: A Randomized Controlled Trial to Compare the Effects of Gas Flows. Pharmaceutics. 2021;13(10):1655

Section 3: Aerosol Delivery via High-flow Nasal Cannula for Adult Patients

Recommendation 3.7： Using heliox to deliver aerosol via high-flow nasal cannula is not recommended.

Distribution of voting scores

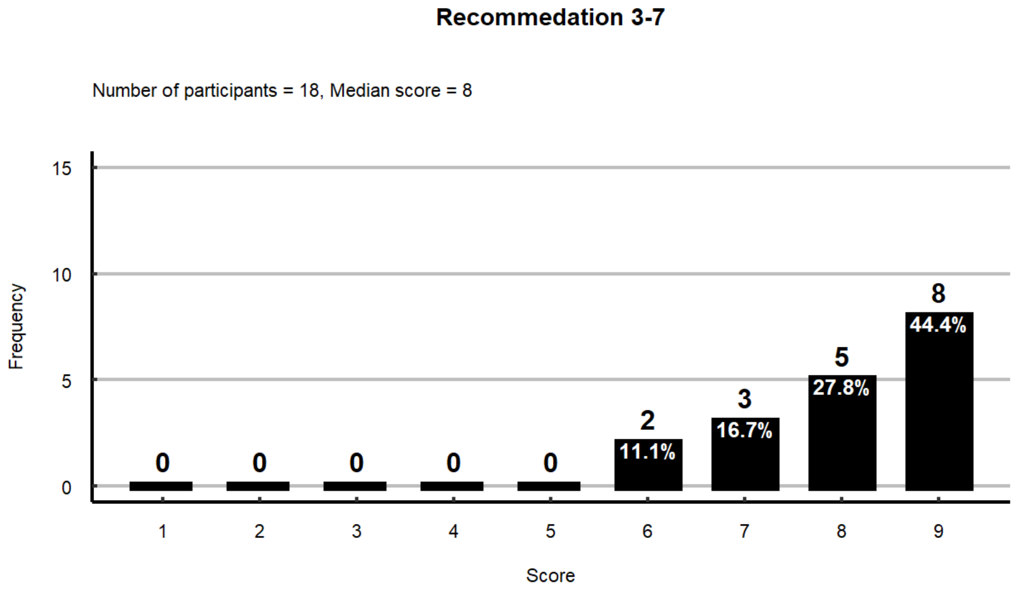

Comments of voting panelists

1. Limited data reported that aerosol delivery with Heliox trough high-flow nasal cannula trends to improve inhaled dose. However, the differences are non significant.
2. Not enough studies.
3. Agree, but very weak evidence.
4. Too complicate even if some cases have been reported.
5. Heliox could be beneficial if there is a narrowed upper airway causing hypoxemia.
6. Rephrase: The use of Heliox to deliver aerosol via high-flow nasal cannula is not recommended.

Round 2

**Recommendations 3.7**	Using heliox to deliver aerosol via high-flow nasal cannula is not recommended.
**Likert score of 1-9**	□1 □2 □3 □4 □5 □6 □7 □8 □9<br>1= absolutely disagree, 9 = absolutely agree
**Comments**	

Section 3: Aerosol Delivery via High-flow Nasal Cannula for Adult Patients

Recommendation 3.8：Using dry gas to deliver aerosol via high-flow nasal cannula has been shown to improve aerosol delivery efficiency, however considering the discomfort and the potential harms, routine use of dry gas to deliver aerosol via high-flow nasal cannula is not recommended.

Distribution of voting scores

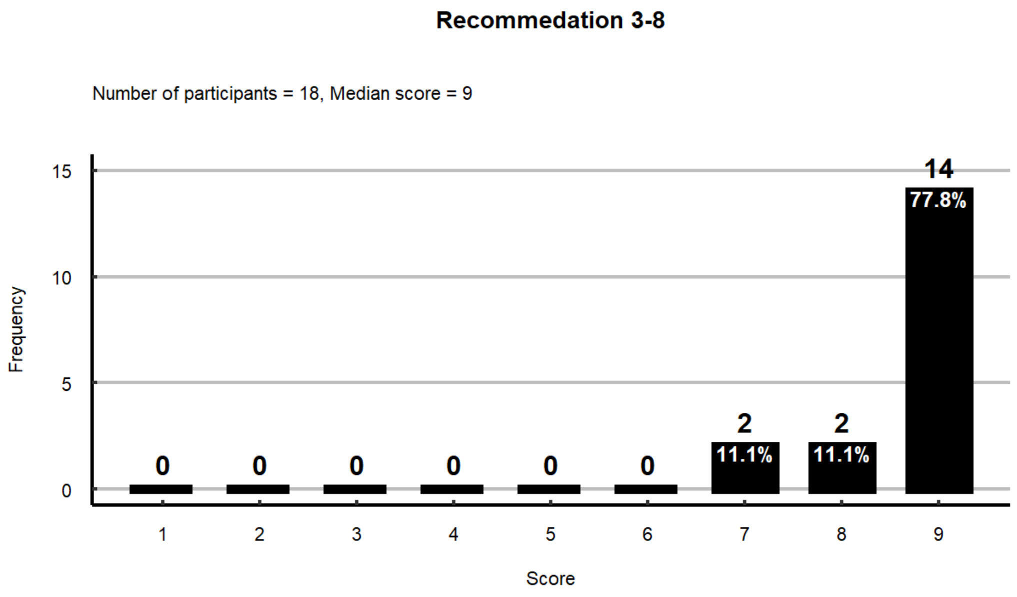

Comments of voting panelists

1. Very weak evidence, but high potential for harm.

2. Jet nebulized should be placed upstream from cannula (patient side); or before HHWH humidifier; mesh should be placed before HHWH during HFONT.

3. I think the statement is logical. Dry gas would be detrimental, regardless of aerosol delivery efficiency.

4. Change using by The use of.

Round 2

**Recommendations 3.8**	Using dry gas to deliver aerosol via high-flow nasal cannula has been shown to improve aerosol delivery efficiency, however considering the discomfort and the potential harms, routine use of dry gas to deliver aerosol via high-flow nasal cannula is not recommended.

**Likert score of 1-9**	□1 □2 □3 □4 □5 □6 □7 □8 □9<br>1= absolutely disagree, 9 = absolutely agree
**Comments**	

Section 3: Aerosol Delivery via High-flow Nasal Cannula for Adult Patients

Recommendation 3.9： When gas flow exceeds patient inspiratory flow, open mouth breathing reduces inhaled dose. Discontinuing aerosol via high-flow nasal cannula to mouth breathing patients is not recommended.

Distribution of voting scores

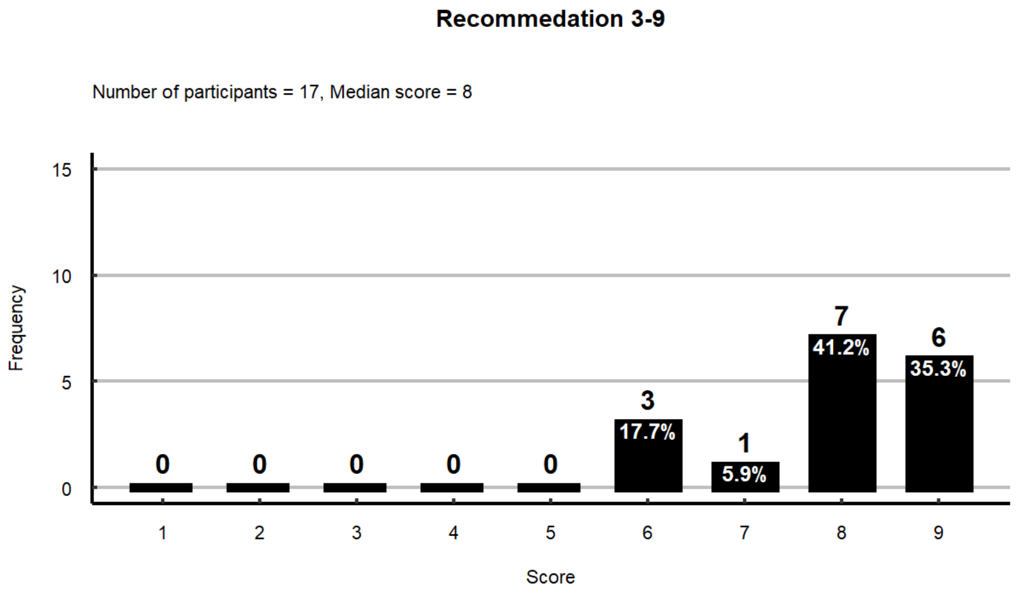

Comments of voting panelists

1. More evidence needed.

2. Limited data reported better drug delivery during high-flow nasal cannula with closed mouth than open mouth breathing.

3. The study Reminiac et al uses a nasal cast model without physiological breathing regading the soft palate operation. It is difficult to conclude without clinical study about the open mouth breathing reduces inhaled dose . I agree with the second part of the recommendation 'Discontinuing aerosol via high-flow nasal cannula to mouth breathing patients is not recommended".

4. I don't understand the question. Open mouth may reduce inhaled dose whatever the flow. What for do we want to discontinue aerosol? It is certainly me but I don't understand the question.

5. In patients in acute respiratory distress, they breathe mainly open-mouthed and require high-flow oxygen, it is not yet known whether nebulization with high-flow oxygen ≥ 40L/min combined with mouth breathing is effective in terms of aerosol delivery efficiency.

6. Makes sense, but very weak evidence.

7. Jet nebulized should be placed upstream from cannula (patient side); or before HHWH humidifier; mesh should be placed before HHWH during HFONT.

8. I believe this is not clinically meaningful. A patient may breathe through the mouth open and at other times with the mouth shut.

9. Not sure I understand the recommendation: why not encouraging keeping mouth close if possible?

Round 2

**Recommendations 3.9**	When gas flow exceeds patient inspiratory flow, open mouth breathing reduces inhaled dose. Discontinuing aerosol via high-flow nasal cannula to mouth breathing patients is not recommended.
**Likert score of 1-9**	□1 □2 □3 □4 □5 □6 □7 □8 □9<br>1= absolutely disagree, 9 = absolutely agree
**Comments**	

Section 3: Aerosol Delivery via High-flow Nasal Cannula for Adult Patients

Recommendation 3.10：For trans-nasal aerosol delivery, Optiflow is preferred over Airvo2 with vibrating mesh nebulizer placed at the inlet of humidifier.

Distribution of voting scores

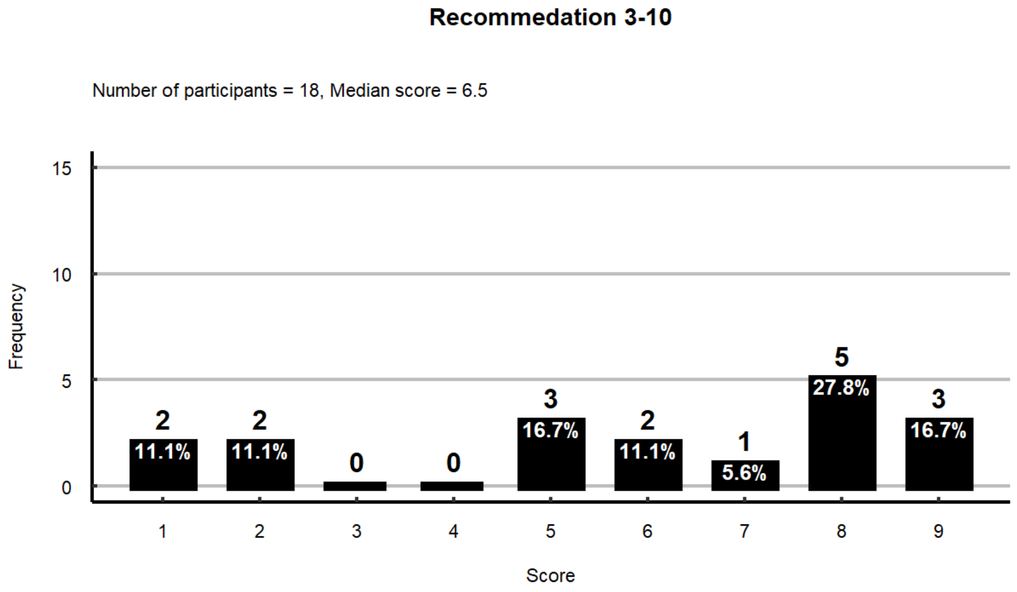

Comments of voting panelists

1. Limited in vitro data reported various results depending on the flow rates, the breathing patterns, and the circuit.

2. It seems difficult to give recommendation to a specific / commercial device with the name of the commercial device. Companies can change the device operation and perfomances in the future. I am not in the favor to use commercial name in recommendations.

3. I would not make recommandations of trademarks. What about Vapotherm etc…

4. Very weak evidence. I would avoid the use of brand names.

5. The difference in aerosol delivery may not be clinically meaningful.

6. I do not think one can recommend a brand over another. This might need to be changed to home vs hospital device.

Round 2

**Recommendations 3.10**	For trans-nasal aerosol delivery, Optiflow is preferred over Airvo2 with vibrating mesh nebulizer placed at the inlet of

	humidifier. **Aerosol delivery via Vapotherm should be avoided.**
**Likert score of 1-9**	□1 □2 □3 □4 □5 □6 □7 □8 □9<br>1= absolutely disagree, 9 = absolutely agree
**Comments**	

Additional references：

1. Li J, Alolaiwat A, Harnois L, Fink JB, Dhand R. Mitigating Fugitive Aerosols during Aerosol Delivery via High-Flow Nasal Cannula Devices. Respir Care. 2021 Nov 17:respcare.09589. doi: 10.4187/respcare.09589.

Appendix 6.0

Round 3 Scoring and Comments

Section 1-1: Aerosol Delivery via Invasive Ventilation for Adult Patients (**not specific to antibiotics**)

Round 3 (please score and comment)

**Recommendations 1-1.1**	During invasive ventilation, vibrating mesh nebulizer is more efficient in aerosol delivery than continuous jet nebulizer, with no influence on flows or fraction of inspired oxygen. ~~**When available,**~~ Vibrating mesh nebulizer is preferred over continuous jet nebulizer.
**Likert score of 1-9**	□1 □2 □3 □4 □5 □6 □7 □8 □9<br>1= absolutely disagree, 9 = absolutely agree
**Comments**	

Round 3 - Recommendation Removed

**Recommendations 1-1.2**	~~**During high-frequency oscillatory ventilation, vibrating mesh nebulizer is more efficient in aerosol delivery than continuous jet nebulizer, with no influence on flows or fraction of inspired oxygen. When available, vibrating mesh nebulizer is recommended over continuous jet nebulizer.**~~
**Comments**	

Round 3 (please score and comment)

**Recommendations 1-1.3**	Based on variation of the reported inhaled doses and lack of definitive clinical outcomes, there is no recommendation for metered dose inhaler and spacer versus vibrating mesh nebulizer.
**Likert score of 1-9**	□1 □2 □3 □4 □5 □6 □7 □8 □9<br>1= absolutely disagree, 9 = absolutely agree

**Comments**	

Round 3 Recommendation Removed

Those recommendations regarding ultrasonic nebulizers will be put in main text as statements.

**Recommendations 1-1.4**	~~**When placed close to the ventilator, the vibrating mesh nebulizer is more efficient in aerosol delivery than ultrasonic nebulizer. When nebulizer is placed at the inspiratory limb before Y-piece, the vibrating mesh nebulizer is as efficient as ultrasonic nebulizer in aerosol delivery.**~~
**Comments**	

Round 3 - Recommendation Removed

**Recommendations 1-1.5**	~~**During high frequency oscillatory ventilation with nebulizer placed between Y-piece and endotracheal tube, vibrating mesh nebulizer is more efficient in aerosol delivery than ultrasonic nebulizer. When available, vibrating mesh nebulizer is recommended over ultrasonic nebulizer.**~~
**Comments**	

Round 3 (please score and comment)

**Recommendations 1-1.6**	When placed at the inspiratory limb before Y-piece, metered

	dose inhaler with a spacer is more efficient in aerosol delivery than the continuous jet nebulizer, with no influence on flows or fraction of inspired oxygen. When available, metered dose inhaler with spacer actuated at beginning of inspiration is recommended over continuous jet nebulizer.
**Likert score of 1-9**	□1 □2 □3 □4 □5 □6 □7 □8 □9<br>1= absolutely disagree, 9 = absolutely agree
**Comments**	

Round 3 - Recommendation Removed

**Recommendations 1-1.7**	~~**When placed at 12-15cm from the Y-piece in the inspiratory limb, ultrasonic nebulizer is more efficient in aerosol delivery than the continuous jet nebulizer, with no influence on flows or fraction of inspired oxygen. When available, ultrasonic nebulizer is recommended over continuous jet nebulizer.**~~
**Comments**	

Round 3 (please score and comment)

**Recommendations 1-1.8**	When vibrating mesh nebulizer is utilized during invasive ventilation with bias flow, it is recommended to be placed ~~**proximal**~~ **close** to ventilator.
**Likert score of 1-9**	□1 □2 □3 □4 □5 □6 □7 □8 □9<br>1= absolutely disagree, 9 = absolutely agree
**Comments**	

Round 3 (please score and comment)

**Recommendations 1-1.9**	When ~~**continuous**~~ jet nebulizer is utilized during invasive ventilation, it is recommended to be placed ~~**proximal to**~~ **near** the ventilator. ~~**When inspiration synchronized jet nebulizer is used, it is recommended to be placed at the inspiration limb before Y-piece.**~~
**Likert score of 1-9**	□1 □2 □3 □4 □5 □6 □7 □8 □9<br>1= absolutely disagree, 9 = absolutely agree
**Comments**	

Round 3 Recommendation Removed

**Recommendations 1-1.10**	~~**When ultrasonic nebulizer is utilized during invasive ventilation without bias flow, it is recommended to be placed at 15 cm from Y-piece at inspiratory limb; With bias flow, ultrasonic nebulizer is recommended to be placed proximal to ventilator.**~~
**Comments**	

Round 3 - Recommendation Removed

**Recommendations 1-1.11**	~~**When ultrasonic nebulizer is placed at the inspiratory limb before Y-piece, adding a spacer is recommended.**~~
**Comments**	

Round 3 (please score and comment)

**Recommendations 1-1.12**	When metered dose inhaler is utilized during invasive ventilation, it is recommended to be used with a spacer with volume > 150mL.
**Likert score of 1-9**	□1 □2 □3 □4 □5 □6 □7 □8 □9<br>1= absolutely disagree, 9 = absolutely agree
**Comments**	

Round 3 (please score and comment)

**Recommendations 1-1.13**	During invasive ventilation, metered dose inhaler and spacer are recommended to be placed in the inspiratory limb before the Y-piece.
**Likert score of 1-9**	□1 □2 □3 □4 □5 □6 □7 □8 □9<br>1= absolutely disagree, 9 = absolutely agree
**Comments**	

Round 3 - Recommendation Removed

**Recommendations 1-1.14**	~~**During high-frequency oscillatory ventilation, nebulizers are recommended to be placed between the Y-piece and the endotracheal tube.**~~
**Comments**	

Round 3 (please score and comment)

**Recommendations 1-1.15**	The efficiency of aerosol delivery in dry ventilator circuits is higher than that in humidified ventilator circuits. Considering the potential harms of dry gas on patient airway, **and the time lapse required for a humidifier and circuits to cool down,** turning off humidifier is not recommended for routine aerosol therapy.
**Likert score of 1-9**	□1 □2 □3 □4 □5 □6 □7 □8 □9<br>1= absolutely disagree, 9 = absolutely agree
**Comments**	

Round 3

Recommendation 1-1.16： When aerosol device is placed in the inspiratory limb, removing or bypassing the heat moisture exchanger is recommended. **(In vitro[1,2], perfect consensus, strong recommendation)**

No need to score.

Round 3 (please score and comment)

**Recommendations 1-1.17**	**In ventilated patients, using a continuous jet nebulizer means adding compressed gas independent of the ventilator. The effect on tidal volume, FiO2 etc makes this practice unacceptable. The empirical compensations on ventilator settings may be dangerous and should be avoided. If no integrated inspiration-synchronized jet nebulizer is available, the use of continuous jet neb in**

	**ventilated patients is not recommended.**
**Likert score of 1-9**	□1 □2 □3 □4 □5 □6 □7 □8 □9<br>1= absolutely disagree, 9 = absolutely agree
**Comments**	

Round 3 Recommendation Removed -This recommendation will be moved to main text and presented as statements

**Recommendations 1-1.18**	~~The influence of ventilator integrated breath-actuated jet nebulizer on ventilator function and aerosol delivery efficiency varies between ventilators.~~
**Comments**	

.

Round 3

Recommendation 1-1.19 : Metered-dose inhaler should be primed, shaken, with actuation at the beginning of inspiration, with a minimum of 15 seconds between puffs. **(In vitro[1,2], very good consensus, strong recommendation)**

<u>**No need to score.**</u>

Round 3 (please score and comment)

**Recommendations 1-1.20.1**	For the jet or ultrasonic nebulizer with a ~~**moderate**~~ residual volume > **0.5 ml**, aerosol delivery efficiency is **improved** ~~**higher**~~ with a higher fill volume, but changing fill volume for the sole purpose of improving aerosol delivery efficiency is not recommended for FDA approved inhaled

	medication.
**Likert score of 1-9**	□1 □2 □3 □4 □5 □6 □7 □8 □9<br>1= absolutely disagree, 9 = absolutely agree
**Comments**	

Round 3 (please score and comment)

**Recommendations 1-1.20.2**	Increasing diluent volume in vibrating mesh nebulizer to improve aerosol delivery efficiency is not recommended.
**Likert score of 1-9**	□1 □2 □3 □4 □5 □6 □7 □8 □9<br>1= absolutely disagree, 9 = absolutely agree
**Comments**	

Round 3 (please score and comment)

**Recommendations 1-1.20.3 (newly added)**	**For viscous formulations, increasing diluent volume in vibrating mesh nebulizer to improve aerosol delivery efficiency is recommended**
**Likert score of 1-9**	□1 □2 □3 □4 □5 □6 □7 □8 □9<br>1= absolutely disagree, 9 = absolutely agree
**Comments**	

Round 3

Recommendation 1-1.21: Aerosol delivery efficiency varies between endotracheal tube and tracheotomy tube. Changing tubes for the sole purpose of improving aerosol delivery efficiency is not recommended. **(In vitro[1-4], perfect consensus, strong recommendation)**

<u>No need to score.</u>

Round 3

Recommendation 1-1.22： Aerosol delivery efficiency is higher with a large size of endotracheal tube, but changing endotracheal tube for the sole purpose of improving aerosol delivery efficiency is not recommended. **(In vitro[1-3], perfect consensus, strong recommendation)**

No need to score.

Round 3

Recommendation 1-1.23： When heliox is utilized for invasive ventilation, aerosol delivery efficiency can improve. However, adding heliox for the sole purpose of improving aerosol delivery efficiency is not recommended. **(In vitro[1], very good consensus, strong recommendation)**

No need to score.

Round 3 (please score and comment)

**Recommendations 1-1.24**	When heliox is substituted for oxygen to drive continuous jet nebulizer at the same driving flow, nebulizer output is reduced. If driving nebulizer with heliox, it is recommended to set at 15 L/min.
**Likert score of 1-9**	□1 □2 □3 □4 □5 □6 □7 □8 □9<br>1= absolutely disagree, 9 = absolutely agree
**Comments**	

Round 3 (please score and comment)

**Recommendations 1-1.25**	~~**Spontaneous breathing may help increase aerosol delivery efficiency, but**~~ It is not recommended to change the ventilator mode for the sole purpose of improving aerosol delivery efficiency.
**Likert score of 1-9**	□1 □2 □3 □4 □5 □6 □7 □8 □9<br>1= absolutely disagree, 9 = absolutely agree
**Comments**	

Round 3 – Recommendation Removed

**Recommendations 1-1.26**	~~**When metered-dose inhaler is utilized during invasive mechanical ventilation, there is no recommendation on flow trigger vs pressure trigger solely for aerosol delivery.**~~
**Comments**	

Round 3 (please score and comment)

**Recommendations 1-1.27**	It is not recommended to change tidal volume and respiratory rate for the sole purpose of improving aerosol delivery efficiency.
**Likert score of 1-9**	□1 □2 □3 □4 □5 □6 □7 □8 □9<br>1= absolutely disagree, 9 = absolutely agree
**Comments**	

Round 3 (please score and comment)

**Recommendations 1-1.28**	~~**Longer inspiratory time and lower inspiratory flows are associated with improved aerosol delivery efficiency, however,**~~ Increasing inspiratory time and lowering inspiratory flows solely for aerosol delivery is not recommended.
**Likert score of 1-9**	□1 □2 □3 □4 □5 □6 □7 □8 □9<br>1= absolutely disagree, 9 = absolutely agree
**Comments**	

Round 3 (please score and comment)

**Recommendations 1-1.29**	It is not recommended to change the inspiratory flow patterns solely for aerosol delivery.
**Likert score of 1-9**	□1 □2 □3 □4 □5 □6 □7 □8 □9<br>1= absolutely disagree, 9 = absolutely agree
**Comments**	

Round 3 (please score and comment)

**Recommendations 1-1.30**	It is not recommended to apply end-inspiratory pause when metered dose inhaler is used during invasive mechanical ventilation.
**Likert score of 1-9**	□1 □2 □3 □4 □5 □6 □7 □8 □9<br>1= absolutely disagree, 9 = absolutely agree

**Comments**	

Round 3 (please score and comment)

**Recommendations 1-1.31**	It is not recommended to change the positive end-expiratory pressure (PEEP) for the sole purpose of improving aerosol delivery efficiency.
**Likert score of 1-9**	□1 □2 □3 □4 □5 □6 □7 □8 □9<br>1= absolutely disagree, 9 = absolutely agree
**Comments**	

Round 3. Recommendation Removed

**Recommendations 1-1.32**	~~**With nebulizer placed proximal to patient, higher bias flow is associated with lower aerosol delivery efficiency. With nebulizer placed proximal to ventilator, adding bias flow up to 5 L/min improves delivery. It is recommended to set bias flow up to 5 L/min when nebulizer is placed proximal to ventilator.**~~
**Comments**	

Round 3 (please score and comment)

**Recommendations 1-1.33**<br>**Newly added**	**Placing a filter on the expiratory limb reduces fugitive aerosols and protects the expiratory sensors. Use of an expiratory filter with frequent changes is recommended.**

**Likert score of 1-9**	□1 □2 □3 □4 □5 □6 □7 □8 □9<br>1= absolutely disagree, 9 = absolutely agree
**Comments**	

Section 1-2: Antibiotics Aerosol Delivery via Invasive Ventilation for Adult Patients

Round 3 (please score and comment)

**Recommendations 1-2.1**	For antibiotics or other cost-prohibitive medications, changing to a dry circuit immediately before nebulization is recommended.
**Likert score of 1-9**	□1 □2 □3 □4 □5 □6 □7 □8 □9<br>1= absolutely disagree, 9 = absolutely agree
**Comments**	

Round 3 (please score and comment)

**Recommendations 1-2.2**	When delivering inhaled antibiotics for invasively ventilated patients, spontaneous breathing ventilator modes **may** reduce aerosol delivery efficiency, thus spontaneous breathing should be ~~**prohibited**~~ **avoided** and volume-controlled mode is ~~**recommended**~~ **preferred, and assessing overall benefit/risk ratio especially related to sedation.**
**Likert score of 1-9**	□1 □2 □3 □4 □5 □6 □7 □8 □9<br>1= absolutely disagree, 9 = absolutely agree
**Comments**	

Round 3 (please score and comment)

**Recommendations 1-2.3**	When delivering inhaled antibiotics for invasively ventilated patients, it is recommended to set tidal volume of 8ml/kg of patient's predicted body weight, **and the clinician must weigh the benefit/risk ratio of increasing tidal volume for improving aerosol delivery with the risk**

	**of high tidal volume.**
**Likert score of 1-9**	□1 □2 □3 □4 □5 □6 □7 □8 □9<br>1= absolutely disagree, 9 = absolutely agree
**Comments**	

Round 3 (please score and comment)

**Recommendations 1-2.4**	When delivering inhaled antibiotics for invasively ventilated patients, it is recommended to keep respiratory rates at 12-15 breaths/min.
**Likert score of 1-9**	□1 □2 □3 □4 □5 □6 □7 □8 □9<br>1= absolutely disagree, 9 = absolutely agree
**Comments**	

Round 3 (please score and comment)

**Recommendations 1-2.5**	When delivering inhaled antibiotics for invasively ventilated patients, it is recommended to keep inspiratory flow below 40L/min.
**Likert score of 1-9**	□1 □2 □3 □4 □5 □6 □7 □8 □9<br>1= absolutely disagree, 9 = absolutely agree
**Comments**	

Round 3 (please score and comment)

**Recommendations 1-2.6**	When delivering inhaled antibiotics for invasively ventilated patients, it is recommended to use ~~inversed~~ inspiratory to expiratory ratio of 50%.
**Likert score of 1-9**	□1 □2 □3 □4 □5 □6 □7 □8 □9<br>1= absolutely disagree, 9 = absolutely agree
**Comments**	

Round 3 (please score and comment)

**Recommendations 1-2.7**	When delivering inhaled antibiotics for invasively ventilated patients, it is recommended to use a constant inspiratory flow.
**Likert score of 1-9**	□1 □2 □3 □4 □5 □6 □7 □8 □9<br>1= absolutely disagree, 9 = absolutely agree
**Comments**	

Round 3 (please score and comment)

**Recommendations 1-2.8**	When delivering inhaled antibiotics for invasively ventilated patients, it is recommended to set end-inspiratory pause at 20%.
**Likert score of 1-9**	□1 □2 □3 □4 □5 □6 □7 □8 □9<br>1= absolutely disagree, 9 = absolutely agree
**Comments**	

Round 3 (please score and comment)

**Recommendations 1-2.9**	When delivering inhaled antibiotics for invasively ventilated patients, it is recommended to set a positive end-expiratory pressure (PEEP) at 5-10 cmH_2O.
**Likert score of 1-9**	□1 □2 □3 □4 □5 □6 □7 □8 □9<br>1= absolutely disagree, 9 = absolutely agree
**Comments**	

Section 2: Aerosol Delivery via **Non-invasive Ventilation** for Adult Patients

Round 3 (please score and comment)

**Recommendations 2.1**	Placing the nebulizer in-line with noninvasive ventilation has similar or higher aerosol delivery efficiency than using the nebulizer with a mask or mouthpiece. Interrupting or discontinuing noninvasive ventilation to administer aerosol via a mask or mouthpiece is ~~recommended~~ **unnecessary and not recommended.**
**Likert score of 1-9**	□1 □2 □3 □4 □5 □6 □7 □8 □9<br>1= absolutely disagree, 9 = absolutely agree
**Comments**	

Round 3

Recommendation 2.2： During noninvasive ventilation using single limb circuit, placing metered dose inhaler with spacer between exhalation valve and mask, with actuation at the beginning of inspiration is recommended. There is no recommendation on the placement orientation (towards or away from patient) of the spacer. **(In vitro[2-4], in vivo[1,4], very good consensus, strong recommendation)**

<u>**No need to score.**</u>

Round 3 (please score and comment)

**Recommendations 2.3**	When placing the continuous nebulizer in-line with noninvasive ventilation, vibrating mesh nebulizer is more efficient in aerosol delivery than jet nebulizer**, with no influence on flows or fraction of inspired oxygen**. When available, vibrating mesh nebulizer is recommended over jet nebulizer.
**Likert score of 1-9**	□1 □2 □3 □4 □5 □6 □7 □8 □9

	1= absolutely disagree, 9 = absolutely agree
**Comments**	

Round 3

Section 2: Aerosol Delivery via Non-invasive Ventilation for Adult Patients

Recommendation 2.4: During noninvasive ventilation using single limb circuit, the continuous nebulizer is recommended to be placed between the exhalation valve and the mask. **(In vitro[1-9], very good consensus, strong recommendation)**

<u>No need to score.</u>

Round 3. Recommendation Removed

**Recommendations 2.5**	~~**During noninvasive ventilation using a single limb circuit, with the continuous nebulizer placed between mask and exhalation valve, there is no recommendation on the type of exhalation valve.**~~
**Comments**	

Round 3

Recommendation 2.6: During aerosol delivery via noninvasive ventilation, turning off the humidifier is not recommended. **(In vitro[1,2], in vivo[1], very good consensus, strong recommendation)**

<u>No need to score.</u>

Round 3 (please score and comment)

**Recommendations 2.7**	The aerosol delivery efficiency is less affected by the fill volume in the vibrating mesh nebulizer than the continuous jet nebulizer. For continuous jet nebulizers, more dilution is associated with greater aerosol delivery. Increasing fill volume for the sole purpose to improve aerosol delivery efficiency is not recommended.
**Likert score of 1-9**	□1 □2 □3 □4 □5 □6 □7 □8 □9<br>1= absolutely disagree, 9 = absolutely agree
**Comments**	

Round 3 (please score and comment)

**Recommendations 2.8**	The aerosol delivery efficiency is similar between CPAP and ~~**BiPAP**~~ **noninvasive ventilation**, changing the ~~**noninvasive ventilation**~~ mode for the sole purpose of increasing aerosol delivery is not recommended.
**Likert score of 1-9**	□1 □2 □3 □4 □5 □6 □7 □8 □9<br>1= absolutely disagree, 9 = absolutely agree
**Comments**	

Round 3 (please score and comment)

**Recommendations 2.9**	When continuous nebulizer is placed between the mask and the exhalation valve during noninvasive ventilation with a single limb circuit, the aerosol delivery efficiency increases as IPAP increases or EPAP decreases. Changing the parameters for the sole purpose to improve aerosol delivery

	efficiency is not recommended.
**Likert score of 1-9**	□1 □2 □3 □4 □5 □6 □7 □8 □9<br>1= absolutely disagree, 9 = absolutely agree
**Comments**	

Round 3 (please score and comment)

**Recommendations 2.10**	When a continuous nebulizer is placed in-line with noninvasive ventilation, the aerosol delivery efficiency is higher with a non-vented mask than a vented mask. Aerosol administration with a vented mask is not recommended.
**Likert score of 1-9**	□1 □2 □3 □4 □5 □6 □7 □8 □9<br>1= absolutely disagree, 9 = absolutely agree
**Comments**	

Round 3 (please score and comment)

**Recommendations 2.11**	When non-vented mask is used during noninvasive ventilation, the aerosol delivery efficiency with optimal position is similar with the single limb and dual limb circuits. There is no recommendation for the use of single versus dual limb circuits for aerosol delivery.
**Likert score of 1-9**	□1 □2 □3 □4 □5 □6 □7 □8 □9<br>1= absolutely disagree, 9 = absolutely agree
**Comments**	

Section 3: Aerosol Delivery via **High-flow Nasal Cannula** for Adult Patients

Round 3 (please score and comment)

**Recommendations 3.1**	The aerosol delivery efficiency with a nebulizer via high-flow nasal cannula **at flow ≤ 35 L/min** is similar to that with a nebulizer and a mask or mouthpiece. Discontinuing high-flow nasal cannula treatment to administer nebulizer with a mask or mouthpiece is not recommended.
**Likert score of 1-9**	□1 □2 □3 □4 □5 □6 □7 □8 □9<br>1= absolutely disagree, 9 = absolutely agree
**Comments**	

Round 3

Recommendation 3.2： Placing a nebulizer with a mask or mouthpiece on a patient who is using concurrent high-flow nasal cannula treatment is not recommended. **(In vitro[1], very good consensus, strong recommendation)**

<u>No need to score.</u>

Round 3

Recommendation 3.3： During aerosol delivery via high-flow nasal cannula, vibrating mesh nebulizer is more efficient in aerosol delivery than jet nebulizer, with no influence on flows or fraction of inspired oxygen. Vibrating mesh nebulizer is recommended for trans-nasal aerosol delivery. **(In vitro[1], in vivo[2,3], very good consensus, strong recommendation)**

<u>No need to score.</u>

Round 3 (please score and comment)

**Recommendations 3.4**	Nebulizers are recommended to be placed at the inlet of humidifier at HFNC flows ≥ 10 L/min.
**Likert score of 1-9**	□1 □2 □3 □4 □5 □6 □7 □8 □9<br>1= absolutely disagree, 9 = absolutely agree
**Comments**	

Round 3 (please score and comment)

**Recommendations 3.5**	When metered dose inhaler is placed in-line with high-flow nasal cannula, it is recommended to be used with a spacer and placed close to nasal cannula with the aerosol plume directed toward the patient.
**Likert score of 1-9**	□1 □2 □3 □4 □5 □6 □7 □8 □9<br>1= absolutely disagree, 9 = absolutely agree
**Comments**	

Round 3 Recommendation Removed

Those recommendations regarding the flow titration during trans-nasal aerosol delivery will be put in the main text as statements.

**Recommendations 3.6**	~~**To optimize aerosol delivery via high-flow nasal cannula, gas flow is recommended to be titrated below the patient's peak inspiratory flow if tolerated.**~~
**Comments**	

Round 3 (please score and comment)

**Recommendations 3.7**	Using heliox **via high-flow nasal cannula for the sole purpose of improving aerosol delivery** is not recommended.
**Likert score of 1-9**	□1 □2 □3 □4 □5 □6 □7 □8 □9<br>1= absolutely disagree, 9 = absolutely agree
**Comments**	

Round 3

Recommendation 3.8: Using dry gas to deliver aerosol via high-flow nasal cannula has been shown to improve aerosol delivery efficiency, however considering the discomfort and the potential harms, routine use of dry gas to deliver aerosol via high-flow nasal cannula is not recommended. **(In vitro[1], very good consensus, strong recommendation)**

<u>**No need to score.**</u>

Round 3 Recommendation Removed

Those recommendations regarding the flow titration during trans-nasal aerosol delivery will be put in the main text as statements.

**Recommendations 3.9**	~~**When gas flow exceeds patient inspiratory flow, open mouth breathing reduces inhaled dose. Discontinuing aerosol via high-flow nasal cannula to mouth breathing patients is not recommended.**~~
**Comments**	

Round 3 Recommendation Removed

Those recommendations regarding the flow titration during trans-nasal aerosol delivery will be put in the main text as statements.

**Recommendations 3.10**	~~**For trans-nasal aerosol delivery, Optiflow is preferred over Airvo2 with vibrating mesh nebulizer placed at the inlet of humidifier. Aerosol delivery via Vapotherm should be avoided.**~~
**Comments**	

Appendix 6.1

Report of Round 2 scoring and comments

Section 1-1: Aerosol Delivery via Invasive Ventilation for Adult Patients (**not specific to antibiotics**)

Section 1-1: Aerosol Delivery via Invasive Ventilation for Adult Patients

Recommendation 1-1.1: During invasive ventilation, vibrating mesh nebulizer is more efficient in aerosol delivery than continuous jet nebulizer, with no influence on flows or fraction of inspired oxygen. When available, vibrating mesh nebulizer is preferred over continuous jet nebulizer.

Distribution of voting scores

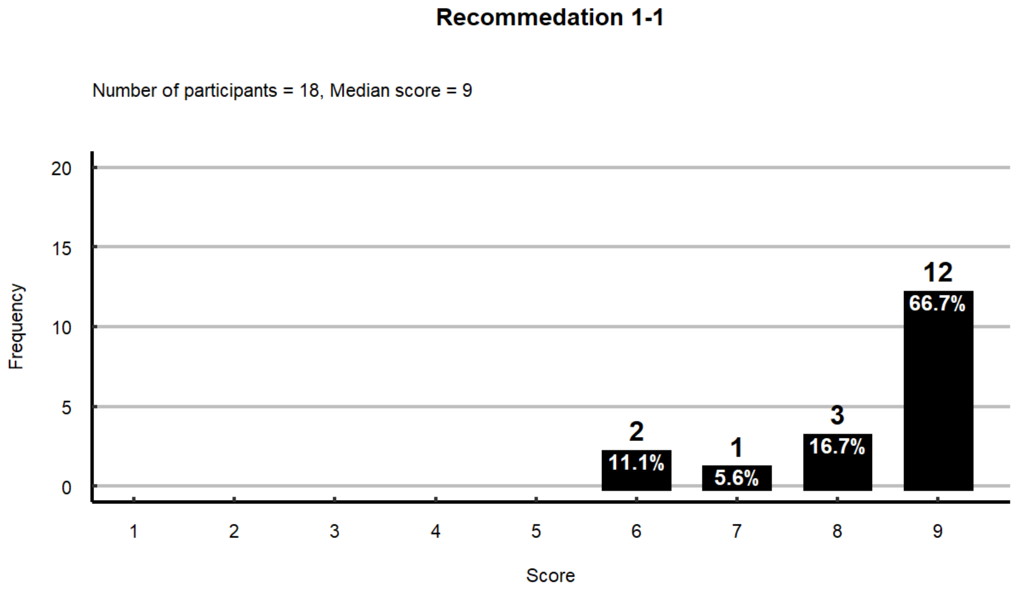

In vitro[1-6,9], in vivo[7,8], very good consensus, strong recommendation

Comments of voting panelists

1. There are some caveats to this recommendation.

2. If the efficiency of nebulizers is defined by the emitted dose and/or the inhaled dose, in the absence of further multi-center clinical studies, I agree with the recommendation regarding bronchodilators and most of antibiotics (question arises for high viscous drugs).I also suggest to add a note of caution: the reliability of reusable vibrating mesh nebulizers may be decreased with repeated use over time.

- Michotte JB, Jossen E, Roeseler J, Liistro G, and Reychler G: In vitro comparison of five nebulizers during noninvasive ventilation: Analysis of inhaled and lost doses. J Aerosol Med Pulm Drug Deliv. 2014;27:430–440.

- Gowda AA, Cuccia AD, and Smaldone GC: Reliability of vibrating mesh

technology. Respir Care. 2017;62:65–69.

3. I agree with comments 2 and 7: preferences may also be based on other factors, such as viscous drugs, cost, reuse or disposable use.

4. I would delete When available.

5. I agree with the recommendation – however in the discussion of the paper we should consider this important issue – that most of the findings are coming from bench studies – and this is also understandable – for specific measurements of delivery – but there are very few studies or almost none - investigating physiologic or relevant clinical outcomes.

6. More is not necessarily better. Cost is not a variable being considered. The difference in efficiency varies with position in the circuit. The in vivo studies not necessarily support your statement.The term flows needs clarification: "ventilator flows".

7. I agree with the statement, but that is my opinion. Guidelines should be based on evidence, not opinion. High level evidence is lacking to support this statement. In GRADE methodology, evidence would be rated very low.

Round 3 (please score and comment)

**Recommendations 1-1.1**	During invasive ventilation, vibrating mesh nebulizer is more efficient in aerosol delivery than continuous jet nebulizer, with no influence on flows or fraction of inspired oxygen. ~~**When available,**~~ Vibrating mesh nebulizer is preferred over continuous jet nebulizer.
**Likert score of 1-9**	□1 □2 □3 □4 □5 □6 □7 □8 □9<br>1= absolutely disagree, 9 = absolutely agree
**Comments**	

Section 1-1: Aerosol Delivery via Invasive Ventilation for Adult Patients

Recommendation 1-1.2: During high-frequency oscillatory ventilation, vibrating mesh nebulizer is more efficient in aerosol delivery than continuous jet nebulizer, with no influence on flows or fraction of inspired oxygen. When available, vibrating mesh nebulizer is recommended over continuous jet nebulizer.

Distribution of voting scores

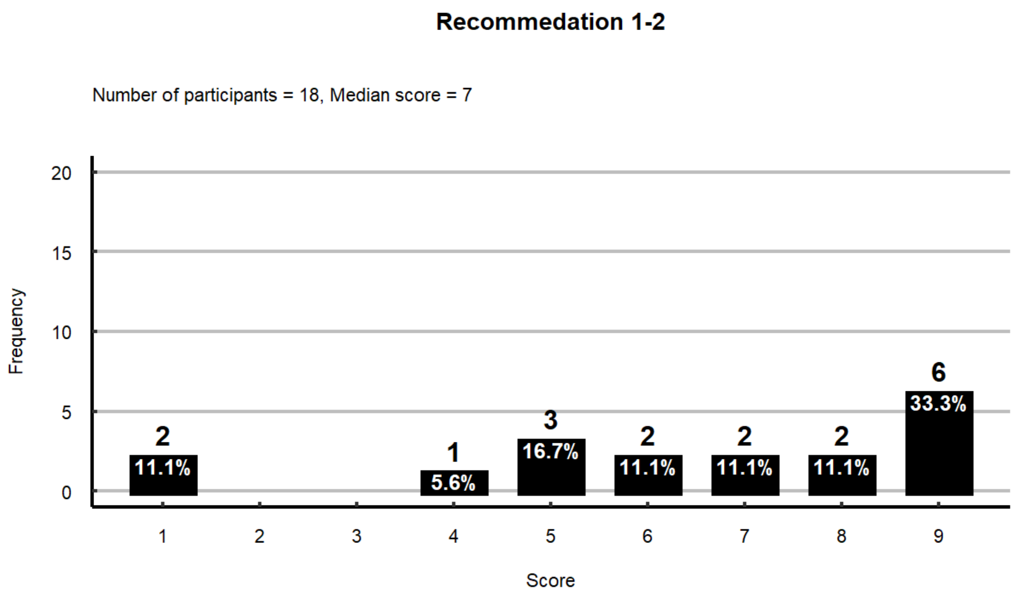

In vitro[1], good consensus, weak recommendation

Comments of voting panelists

1. Insufficient data to make a recommendation.

2. If I understood correctly this recommendation will be taken out at the next round. I agree with removing it as HFO is of marginal practice.

3. I agree with the recommendation, but there is not sufficient data to conclude.

4. Lack of in vitro and in vivo studies, probably because of the limited clinical use of HFO. Therefore, is there any interest for this recommendation?

5. There are 2 different ideas in this recommendation. One is for the efficacy, one is for safety. For safety , I agree- for efficiency I disagree because not enough data to conclude. Maybe it could be interesting to has a specific recommendation about safety/risk regarding nebulizer/device in mechanical ventilation …for example: risk of ventilation

parameters modification with jet ….

6. Again I would delete" when available.

7. Too limited evidence from bench studies.

8. Recommendation needs to specify location because in one study jet before ETT delivered 5x more drug than VMN at the ventilator.

9. I suggest to modify for "During high-frequency oscillatory ventilation in preterm infants,.. "With the actual formulation I vote 4, with the new formulation I would vote 7.

10. Eliminate this. Evidence is lacking to ever use HFOV + bronchodilator. No clinical support for this therapy.

Round 3 - Recommendation Removed

**Recommendations 1-1.2**	**~~During high-frequency oscillatory ventilation, vibrating mesh nebulizer is more efficient in aerosol delivery than continuous jet nebulizer, with no influence on flows or fraction of inspired oxygen. When available, vibrating mesh nebulizer is recommended over continuous jet nebulizer.~~**
**Comments**	

Section 1-1: Aerosol Delivery via Invasive Ventilation for Adult Patients

Recommendation 1-1.3： Based on variation of the reported inhaled doses and lack of definitive clinical outcomes, there is no recommendation for metered dose inhaler and spacer versus vibrating mesh nebulizer.

Distribution of voting scores

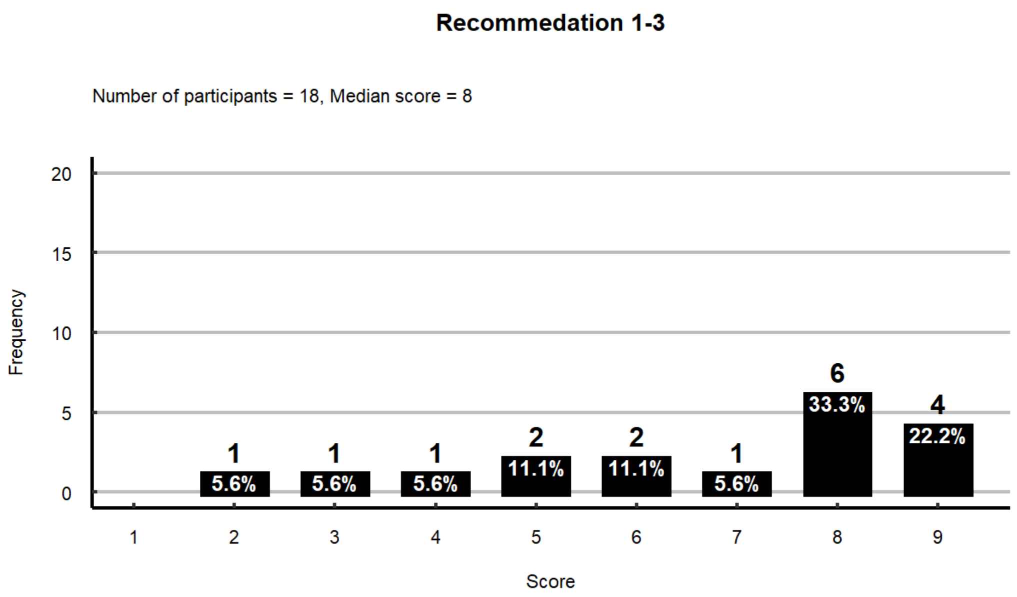

In vitro[1-3,5], in vivo[2,4,6,7], some consensus, weak recommendation

Comments of voting panelists

1. With the caveat that this applies only to administration of bronchodilators.

2. I would change it as follows:

Among ventilated patients, based on variation of the reported inhaled doses and lack of definitive clinical outcomes, there is no recommendation for metered dose inhaler and spacer versus vibrating mesh for delivering bronchodiltors.

3. Except non-significant results in favor of metered dose inhaler in some studies, no difference can be really found between both modalities.

4. I suggest adding “for bronchodilator therapy” after the sentence “there is no recommendation for metered dose inhaler and spacer versus vibrating mesh”.

5. When I read the Comments of voting panelistss, it seems that there is a consensus that pMDI=neb in term of efficacy but there is no consensus in reported score. Maybe

the recommendation could be modify for a better consensus for exemple: “Metered dose inhaler and spacer vs vibrating mesh is similar in terms of efficiency for bronchodilatator delivery”.

6. We should avoid to use metered dose inhaler.

7. Add “Based on variation of the reported inhaled doses and lack of definitive clinical outcomes for the use of bronchodilators”.

8. I agree with most of comments below – that mesh should be preferred to MDI – especially during mechanical ventilation – due to best dosage control – less complicate application and workload – highest delivery mainly for antibiotics but also in my opinion for bronchodilators and other drugs – curious to see the final responses.

9. The table needs to be redone. You should not compare efficiency when the loading doses are different.

10. I suggest to modify for “Based on variation of the reported inhaled doses of bronchodilators… “. Metered dose inhaler are not recommended for antibiotic nebulization.

11. Needs to state that this only applies to bronchodilators. Very low level of evidence. This is based more on opinion than evidence.

12. The preference should be based on available drugs in pMDI and cost.

Round 3 (please score and comment)

**Recommendations 1-1.3**	Based on variation of the reported inhaled doses and lack of definitive clinical outcomes, there is no recommendation for metered dose inhaler and spacer versus vibrating mesh nebulizer.
**Likert score of 1-9**	□1 □2 □3 □4 □5 □6 □7 □8 □9<br>1= absolutely disagree, 9 = absolutely agree
**Comments**	

Section 1-1: Aerosol Delivery via Invasive Ventilation for Adult Patients

Recommendation 1-1.4: When placed close to the ventilator, the vibrating mesh nebulizer is more efficient in aerosol delivery than ultrasonic nebulizer. When nebulizer is placed at the inspiratory limb before Y-piece, the vibrating mesh nebulizer is as efficient as ultrasonic nebulizer in aerosol delivery.

Distribution of voting scores

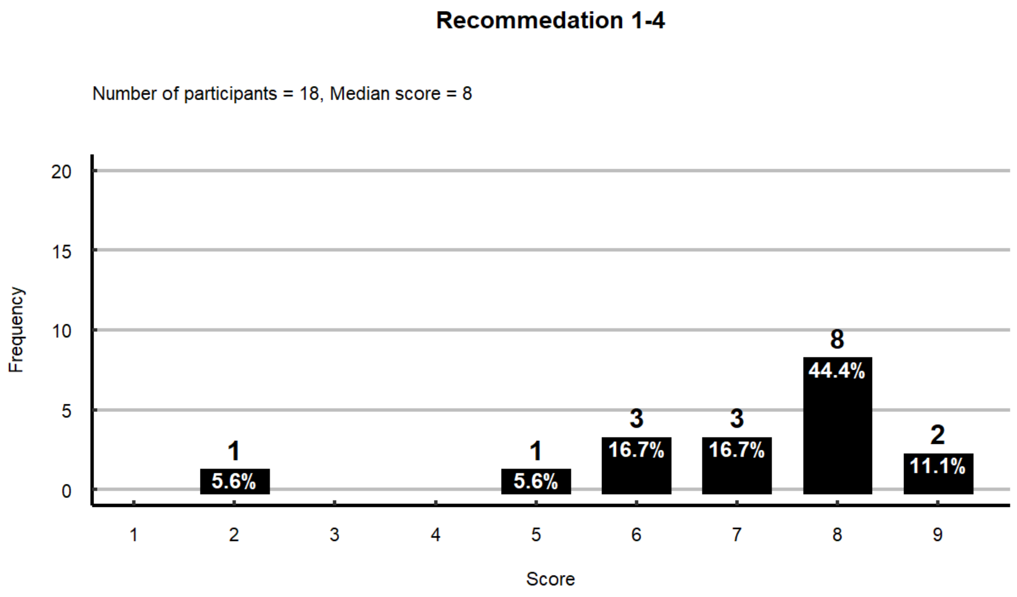

In vitro[1-3], animal research[4], good consensus, weak recommendation

Comments of voting panelists

1. (When placed at inspiratory limb before Y piece, the vibrating mesh nebulizer is as efficient as ultrasonic nebulizer in aerosol delivery. When nebulizer is placed proximal to ventilator, the vibrating mesh nebulizer is more efficient in aerosol delivery than ultrasonic nebulizer.)Not enough evidence to make a recommendation. The revised statement is not a recommendation.

2. I don’t think there is sufficient evidence for this recommendation and there is for me a complet lack of rational why the two types of devices would behave differently at the Y versus at the ventilator.

3. Ultrasonic nebulizer is less likely to be used through ventilator in the current practice. I suggest abandon this recommendation.

4. To my knowledge, ultrasonic nebulizer is not commonly used in aerosol delivery in Europe. Therefore, is there any interest for this recommendation?

I disagree with the new formulation of the recommendation and suggest changing it to the previous one. Personally, I prefer to say that “there is no recommendation” rather than “as efficient as”.

5. I agree with the second part of the proposal. As for the first part about the nebulizer close to the ventilator, there is not much evidence to support this.

6. Seems to be as efficient…..

7. USN has not been commonly used in many countries.

8. Remove parker 2017.

9. Low level evidence and the statement about USN is of dubious importance. Does anyone use USN during mechanical ventilation? Seems to be a “who cares” statement.

Round 3 Recommendation Removed

Those recommendations regarding ultrasonic nebulizers will be put in main text as statements.

**Recommendations 1-1.4**	~~**When placed close to the ventilator, the vibrating mesh nebulizer is more efficient in aerosol delivery than ultrasonic nebulizer. When nebulizer is placed at the inspiratory limb before Y-piece, the vibrating mesh nebulizer is as efficient as ultrasonic nebulizer in aerosol delivery.**~~
**Comments**	

Section 1-1: Aerosol Delivery via Invasive Ventilation for Adult Patients

Recommendation 1-1.5: During high frequency oscillatory ventilation with nebulizer placed between Y-piece and endotracheal tube, vibrating mesh nebulizer is more efficient in aerosol delivery than ultrasonic nebulizer. When available, vibrating mesh nebulizer is recommended over ultrasonic nebulizer.

Distribution of voting scores

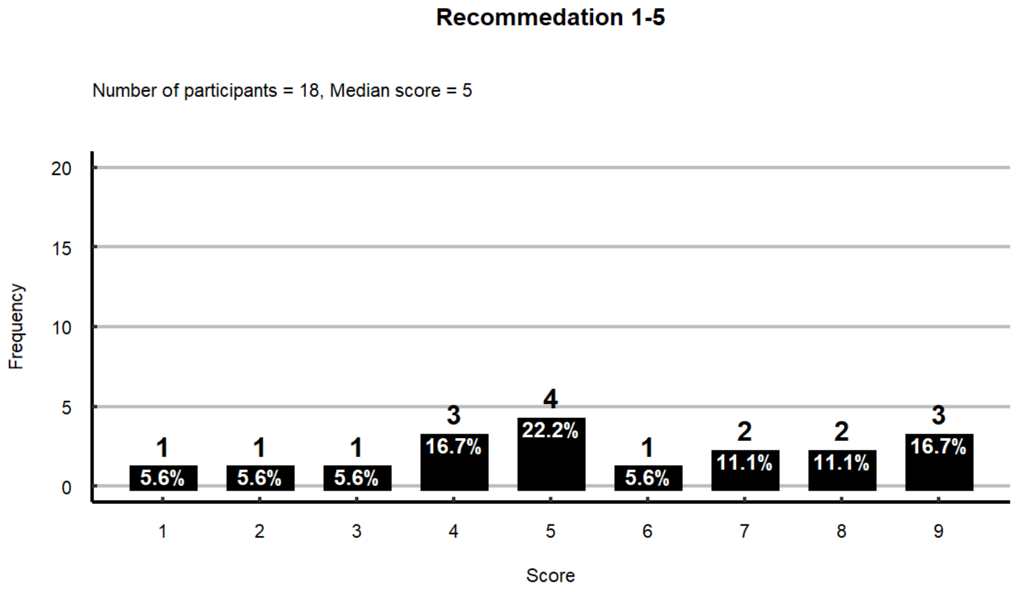

In vitro[1], no consensus, no recommendation

Comments of voting panelists

1. Insufficient evidence to make a recommendation.

2. This will be removed at round 3, correct? I think it is not very useful to compare mesh and ultrasonic nebulizers in general.

3. Evidence is still low.

4. Low evidence.

5. Lack of in vitro and in vivo studies, probably because of the limited clinical use of HFO and ultrasonic nebulizer. Therefore, is there any interest for this recommendation?

6. Lack of evidence.

7. Again I would delete" when available".

8. Suggest delete this recommendation, due to lack of evidence.

9. I personally agree on the skepticism of other members of the Team … no clear consensus on this important point.

10. lack of evidence.

11. 1.2 and 1.5 should be combined.

12. I suggest to modify for “During high-frequency oscillatory ventilation in preterm infants,.. “With the actual formulation I vote 4, with the suggested new formulation I would vote 7.

13. No indication for aerosol delivery with HFOV.

14. I would likely remove this recommendation.

Round 3 - Recommendation Removed

**Recommendations 1-1.5**	**~~During high frequency oscillatory ventilation with nebulizer placed between Y-piece and endotracheal tube, vibrating mesh nebulizer is more efficient in aerosol delivery than ultrasonic nebulizer. When available, vibrating mesh nebulizer is recommended over ultrasonic nebulizer.~~**
**Comments**	

Section 1-1: Aerosol Delivery via Invasive Ventilation for Adult Patients

Recommendation 1-1.6: When placed at the inspiratory limb before Y-piece, metered dose inhaler with a spacer is more efficient in aerosol delivery than the continuous jet nebulizer, with no influence on flows or fraction of inspired oxygen. When available, metered dose inhaler with spacer actuated at beginning of inspiration is recommended over continuous jet nebulizer.

Distribution of voting scores

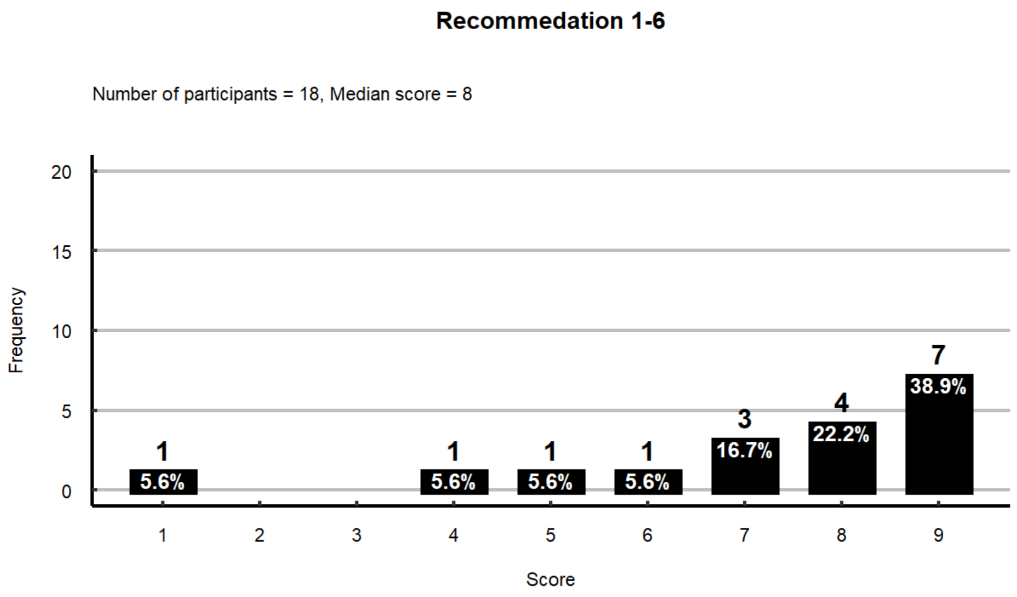

In vitro[1,2,5], in vivo[3,4,6,7], good consensus, weak recommendation

Comments of voting panelists

1. Insufficient evidence to make a recommendation.

In the Manthous study (1993), the adapter used with the pMDI had very poor efficiency. When a cylindrical adapter is used the pMDI and spacer had an equivalent bronchodilator effect as a jet nebulizer (please see references that I mentioned in my earlier response). This study did not show superiority of nebulizers, it just showed that one has to use an optimal technique with either device.

2. I agree with the new formulation of the recommendation.

3. Caution: It does not apply to antibiotics.

4. In the end what matters is how much drug reaches the lungs. Therefore, the

comparison should be made mcg to mcg.

5. I suggest to modify for “When placed at the inspiratory limb before Y-piece, metered dose inhaler with a spacer is more efficient in aerosol delivery of bronchodilators and/or corticosteroids…

With the actual formulation, I vote 4, with the suggested new formulation, I would vote 9.

6. I agree, but based on opinion. Very low level of evidence.

Round 3 (please score and comment)

**Recommendations 1-1.6**	When placed at the inspiratory limb before Y-piece, metered dose inhaler with a spacer is more efficient in aerosol delivery than the continuous jet nebulizer, with no influence on flows or fraction of inspired oxygen. When available, metered dose inhaler with spacer actuated at beginning of inspiration is recommended over continuous jet nebulizer.
**Likert score of 1-9**	□1 □2 □3 □4 □5 □6 □7 □8 □9<br>1= absolutely disagree, 9 = absolutely agree
**Comments**	

Section 1-1: Aerosol Delivery via Invasive Ventilation for Adult Patients

Recommendation 1-1.7: When placed at 12-15cm from the Y-piece in the inspiratory limb, ultrasonic nebulizer is more efficient in aerosol delivery than the continuous jet nebulizer, with no influence on flows or fraction of inspired oxygen. When available, ultrasonic nebulizer is recommended over continuous jet nebulizer.

Distribution of voting scores

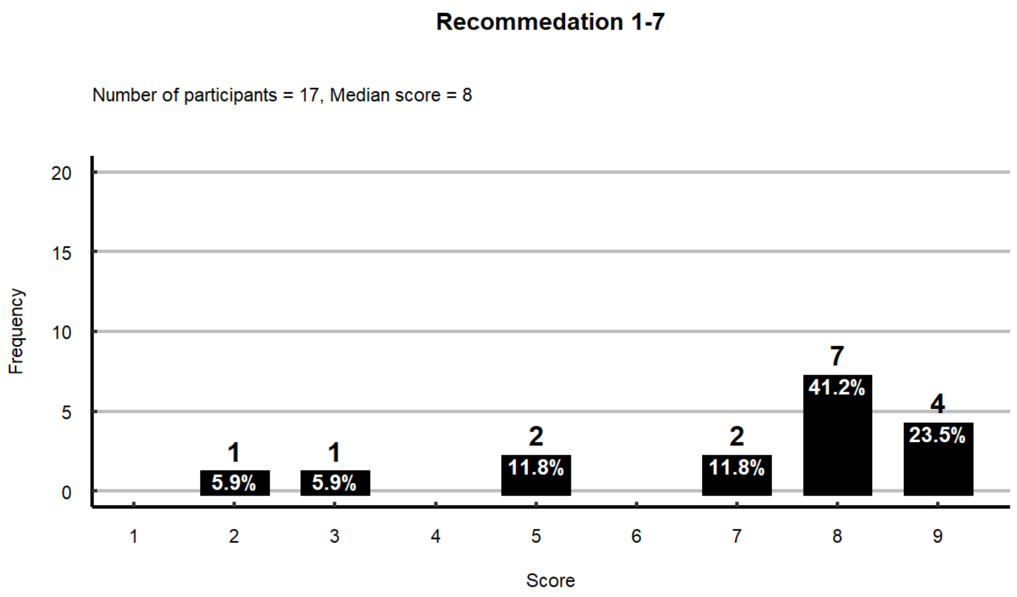

In vitro [1,2], in vivo [3], some consensus, weak recommendation

Comments of voting panelists

1. Insufficient evidence to make a recommendation.

2. The recommendation is true comparing to the delivery percent of initial dose. However, the actual delivered dose is much greater with a jet nebulizer.

3. Limited data suggested better drug delivery with ultrasonic nebulizer. I agree regarding no influence on flows or fraction of inspired oxygen.

4. Recommendations and preferences need to consider other factors. Ultrasound equipment is bulky, and the temperature it produces may change the physical properties of some drugs.

5. Why we have to say always when available ?

6. USN has not been commonly used in many countries.

7. Cost efficiency is not considered when making this recommendation.

8. Question clinical relevance. Does anyone use USN during mechanical ventilation?

Round 3 - Recommendation Removed

**Recommendations 1-1.7**	~~**When placed at 12-15cm from the Y-piece in the inspiratory limb, ultrasonic nebulizer is more efficient in aerosol delivery than the continuous jet nebulizer, with no influence on flows or fraction of inspired oxygen. When available, ultrasonic nebulizer is recommended over continuous jet nebulizer.**~~
**Comments**	

Section 1-1: Aerosol Delivery via Invasive Ventilation for Adult Patients

Recommendation 1-1.8: When vibrating mesh nebulizer is utilized during invasive ventilation with bias flow, it is recommended to be placed proximal to ventilator.

Distribution of voting scores

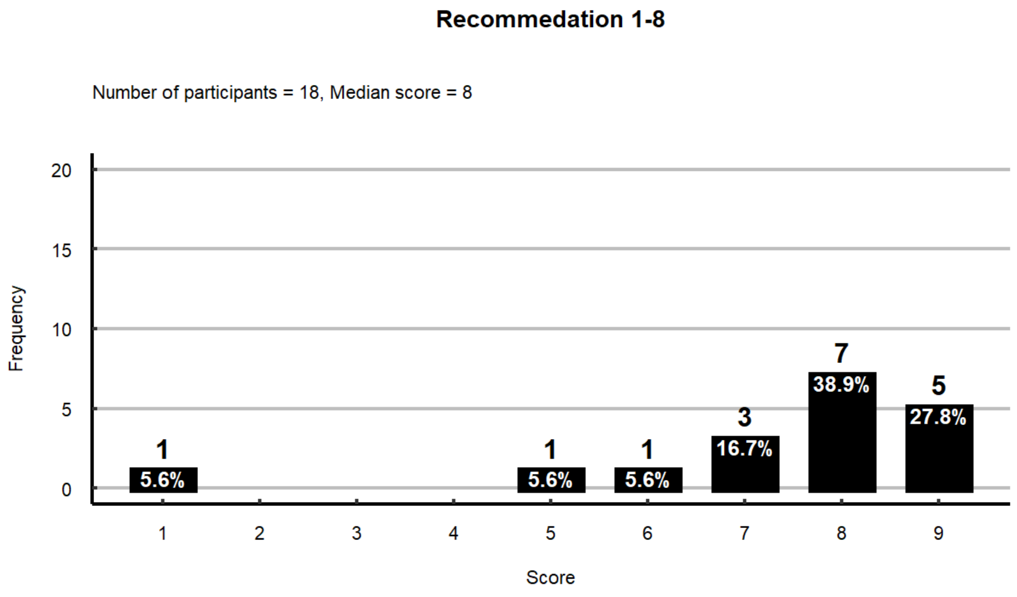

In vitro[1-5], very good consensus, strong recommendation

Comments of voting panelists

1. the positioning of Jet or Mesh in the circuit – as well as – if during mechanical ventilation or CPAP (delivered by ventilator or high flows) – finally not clear.

2. Need more clinical data to support this recommendation.

3. Careful with the word proximal some people use it in opposite ways. I would rather write "close to the ventilator and at distance of the Y piece".

4. I disagree with the new formulation of the recommendation. To my knowledge, all modern mechanical ventilators have a bias flow. Therefore, is it necessary to specify it? I agree to replace "at the inlet of humidifier" by "proximal to ventilator". The optimal position of the nebulizer depends on the bias flow.

5. I theoretically agree but need clinical data to support this.

6. … but without HME – it is my opinion that there is a bias that all patients have HWH – but more than 90% of mechanically ventilated patients have HME – please check.

7. I suggest to modify for “When vibrating mesh nebulizer is utilized during invasive ventilation with bias flow to deliver bronchodilators and/or corticosteroids, it is recommended to be placed proximal to ventilators.

With the actual formulation, I vote 1, with the suggested new formulation I would vote 7. It has to be pointed out that 99% of modern ventilators have a minimum bias flow of 2 L/min that cannot be cancelled…

8. I think you mean distal to the ventilator, proximal to the humidifier. Very low level of evidence.

Round 3 (please score and comment)

**Recommendations 1-1.8**	**When vibrating mesh nebulizer is utilized during invasive ventilation with bias flow, it is recommended to be placed ~~proximal~~ close to ventilator.**
**Likert score of 1-9**	□1 □2 □3 □4 □5 □6 □7 □8 □9<br>1= absolutely disagree, 9 = absolutely agree
**Comments**	

Section 1-1: Aerosol Delivery via Invasive Ventilation for Adult Patients

Recommendation 1-1.9: When continuous jet nebulizer is utilized during invasive ventilation, it is recommended to be placed proximal to the ventilator. When inspiration synchronized jet nebulizer is used, it is recommended to be placed at the inspiration limb before Y-piece.

Distribution of voting scores

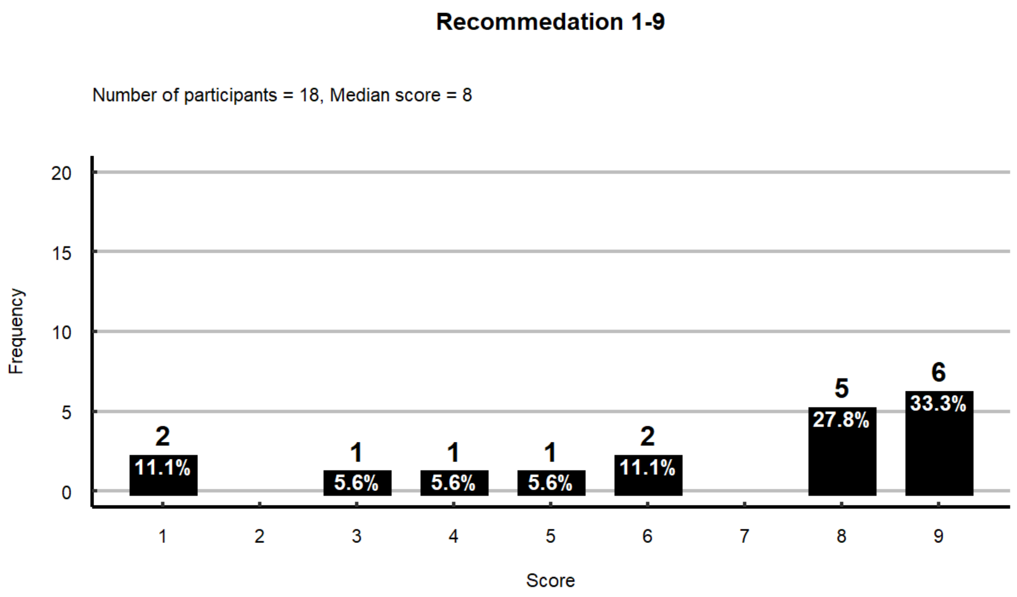

In vitro[1-3], no consensus, no recommendation

Comments of voting panelists

1. The differences are not likely to be clinically meaningful.

2. Bench data shows synchronized jet nebulizers (ie breath actuated) in fact are not synchronized with significant nebulization during expiration. Thus placement should be the same than for continuous nebulization.

3. A jet nebulizer should be used with continue mode, and synchronization is not recommended due to a much longer treatment time.

Wan GH, Lin HL, Fink JB et al, In Vitro Evaluation of Aerosol Delivery by Different Nebulization Modes in Pediatric and Adult Mechanical Ventilators. Respir Care. 2014(10):1494-1500.

4. I agree with this recommendation, however clinical studies with modern mechanical

ventilators are needed.

5. … but without HME – it is my opinion that there is a bias that all patients have HWH – but more than 90% of mechanically ventilated patients have HME – please check.

6. Reference 2 is closer to clinical practice and showed no difference. Should the reference to bias flow also added?

7. I suggest to modify for "When continuous jet nebulizer is utilized during invasive ventilation bronchodilators and/or corticosteroids, it is recommended to be placed proximal to the ventilator. When inspiration synchronized jet nebulizer is used, it is recommended to be placed at the inspiration limb before Y-piece.

With the actual formulation, I vote 1, with the new formulation I would vote 7.Placing jet nebulizers proximal to the ventilator (before the conventional humidifier) is not recommended for delivering antibiotics.

8. "proximal to the ventilator"? does not make sense. Do you mean proximal to the humidifier? Very low evidence.

Round 3 (please score and comment)

**Recommendations 1-1.9**	When ~~**continuous**~~ jet nebulizer is utilized during invasive ventilation, it is recommended to be placed ~~**proximal to**~~ **near** the ventilator. ~~**When inspiration synchronized jet nebulizer is used, it is recommended to be placed at the inspiration limb before Y-piece.**~~
**Likert score of 1-9**	□1 □2 □3 □4 □5 □6 □7 □8 □9<br>1= absolutely disagree, 9 = absolutely agree
**Comments**	

Section 1-1: Aerosol Delivery via Invasive Ventilation for Adult Patients

Recommendation 1-1.10 : When ultrasonic nebulizer is utilized during invasive ventilation without bias flow, it is recommended to be placed at 15 cm from Y-piece at inspiratory limb; With bias flow, ultrasonic nebulizer is recommended to be placed proximal to ventilator.

Distribution of voting scores

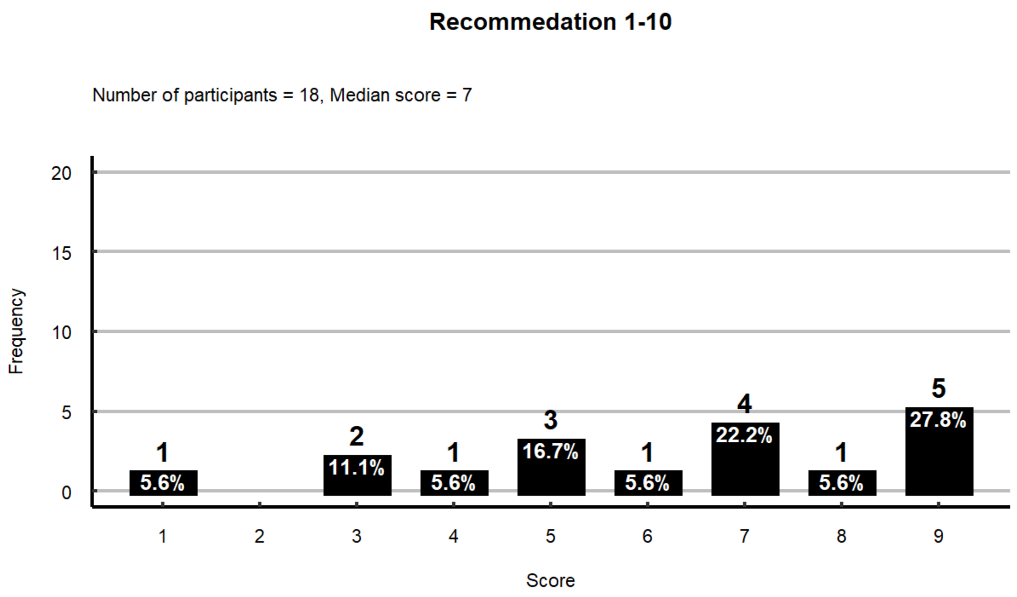

In vitro[1-4], in vivo[5], some consensus, weak recommendation

Comments of voting panelists

1. Differences are not likely to be clinically meaningful.

2. I don't think any current ventilator works without bias flow, so I would suppress this recommendation.

3. USN has been poorly studied to conclude.

4. I agree to replace "at the inlet of humidifier" by "proximal to ventilator".

To my knowledge, all modern mechanical ventilators have a bias flow. Therefore, is it necessary to make a recommendation about invasive ventilation without bias flow?

The optimal position of the nebulizer depends on the bias flow.

5. … but without HME – it is my opinion that there is a bias that all patients have HWH – but more than 90% of mechanically ventilated patients have HME – please check.

6. Half of the studies don't specify bias flow. The clinical study does not favor any site.

7. I suggest to modify for "When ultrasonic nebulizer is utilized during invasive ventilation without bias flow to deliver bronchodilators and/or corticosteroids,, it is recommended to be placed at 15 cm from Y-piece at inspiratory limb; With bias flow, ultrasonic nebulizer is recommended to be placed proximal to ventilator it is recommended to be placed proximal to ventilators.

With the actual formulation, I vote 1, with the suggested new formulation I would vote

7. Placing ultrasonic nebulizers proximal to the ventilator (before the conventional humidifier) is not recommended for delivering antibiotics.

It has to be pointed out that 99% of modern ventilators have a minimum bias flow of 2 L/min that cannot be cancelled…

8. "proximal to the ventilator"? questionable clinical importance. Very low evidence.

Round 3 Recommendation Removed

**Recommendations 1-1.10**	~~**When ultrasonic nebulizer is utilized during invasive ventilation without bias flow, it is recommended to be placed at 15 cm from Y-piece at inspiratory limb; With bias flow, ultrasonic nebulizer is recommended to be placed proximal to ventilator.**~~
**Comments**	

Section 1-1: Aerosol Delivery via Invasive Ventilation for Adult Patients

Recommendation 1-1.11： When ultrasonic nebulizer is placed at the inspiratory limb before Y-piece, adding a spacer is recommended.

Distribution of voting scores

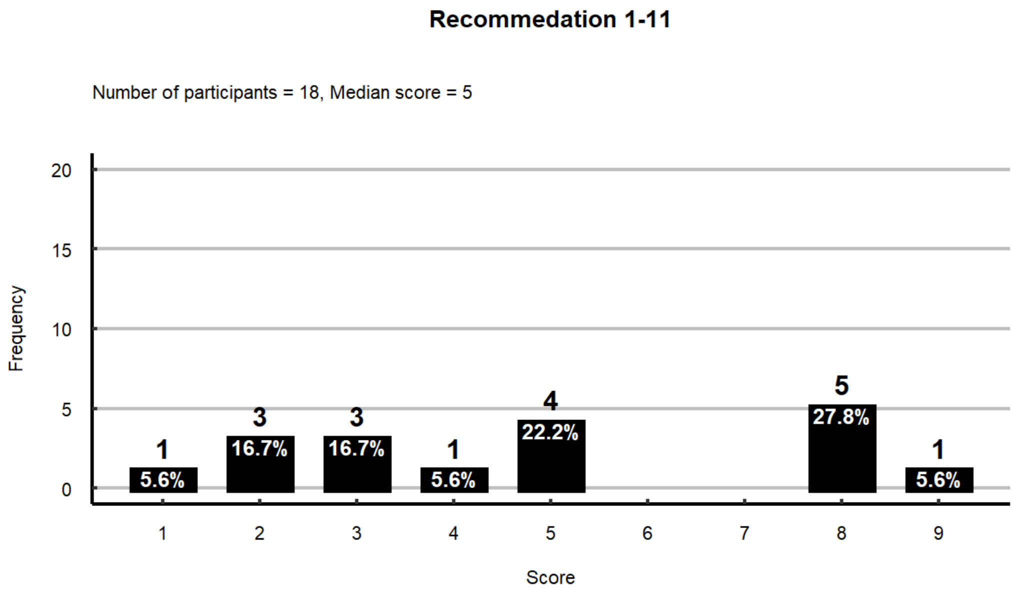

In vitro[1,2], no consensus, no recommendation

Comments of voting panelists

1. In vitro data show variability; need more clinical data.

2. This is not specific of a nebulizer type. The spacer is just a way to place the nebulizer away from the Y.

3. Not enough data to generalize.

4. Studies are too old. It may not apply to current technology.

5. Limited in vitro data suggested better drug delivery when adding a spacer.

Lack of in vitro and in vivo studies, probably because of the limited clinical use of ultrasonic nebulizer in mechanical ventilation. Therefore, is there any interest for this recommendation?

6. I agree with the Comments of voting panelistss: studies too old, no strong data to support this recommendation.

7. Warning: It does not apply to antibiotics.

8. A spacer for ultrasonic nebulizer is not commercially available in lots of countries/areas, plus adding a spacer in ventilator circuit increases the dead space.

9. The spacer with USN is not commonly used.

10. Are spacers for these purpose commercially available? Are these devices available?The 2 studies are 3 decades old.

11. I suggest to modify for “When ultrasonic nebulizer is placed at the inspiratory limb before Y-piece, adding a spacer is recommended for delivering bronchodilators and corticosteroids, With the actual formulation I vote 1, with the suggested new formulation I would vote 7

12. Questionable relevance. Very low level of evidence.

Round 3 - Recommendation Removed

**Recommendations 1-1.11**	~~**When ultrasonic nebulizer is placed at the inspiratory limb before Y-piece, adding a spacer is recommended.**~~
**Comments**	

Section 1-1: Aerosol Delivery via Invasive Ventilation for Adult Patients

Recommendation 1-1.12： When metered dose inhaler is utilized during invasive ventilation, it is recommended to be used with a spacer with volume > 150mL.

Distribution of voting scores

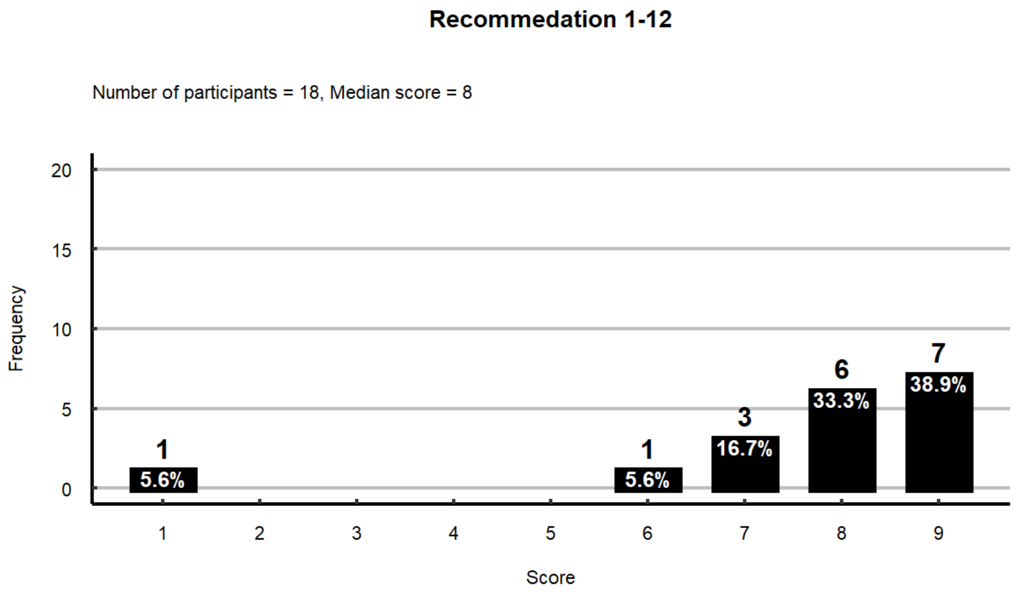

In vitro[1-7], in vivo[8,9], very good consensus, strong recommendation

Comments of voting panelists

1. The type of adapter used could have a significant effect on the clinical response.
2. Limited in vivo data showed higher drug delivery when large spacer is used.

To respond to comment 7: To my knowledge, antibiotics are not available in pMDI formulations. Therefore, the recommendation concerns corticosteroid and bronchodilators.

3. Warning: It does not apply to antibiotics.
4. We should use mcg as well as % to allow comparison with other devices/situations.
5. I suggest to modify for “When metered dose inhaler is utilized during invasive ventilation for delivering bronchodilators and corticosteroids,.., it is recommended to be used with a spacer with volume > 150mL. With the actual formulation I vote 1, with the suggested new formulation I would vote 7

Metered dose inhaler are not recommended for antibiotic nebulization.

6. I agree, but it is my opinion. Very low level of evidence.

Round 3 (please score and comment)

**Recommendations 1-1.12**	When metered dose inhaler is utilized during invasive ventilation, it is recommended to be used with a spacer with volume > 150mL.
**Likert score of 1-9**	□1 □2 □3 □4 □5 □6 □7 □8 □9<br>1= absolutely disagree, 9 = absolutely agree
**Comments**	

Section 1-1: Aerosol Delivery via Invasive Ventilation for Adult Patients

Recommendation 1-1.13: During invasive ventilation, metered dose inhaler and spacer are recommended to be placed in the inspiratory limb before the Y-piece.

Distribution of voting scores

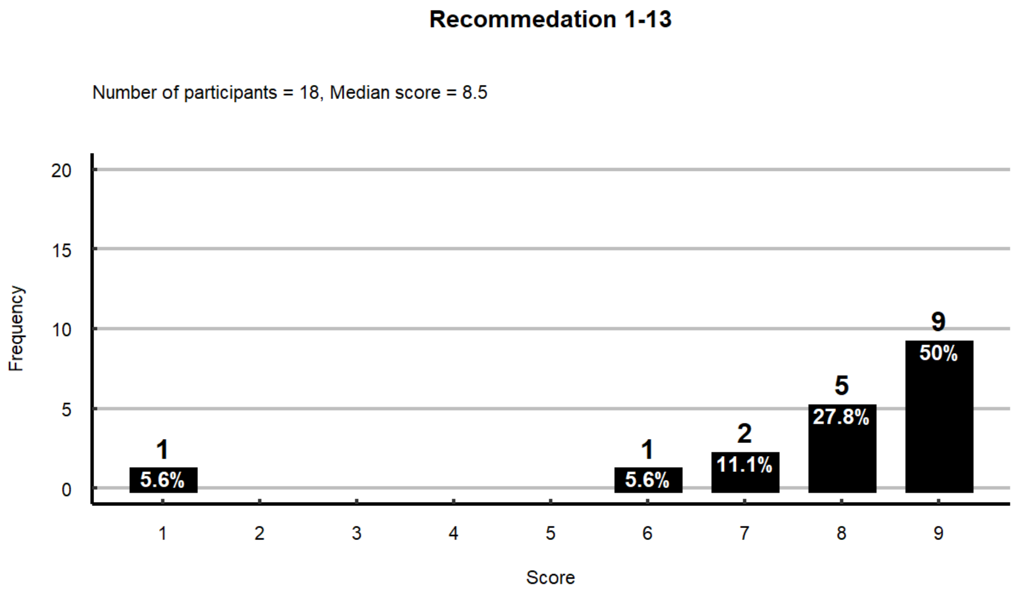

In vitro[1,2], in vivo[3], very good consensus, strong recommendation

Comments of voting panelists

1. Even if there are limited data suggesting better drug delivery with metered dose inhaler and spacer placed before the Y-piece (compared to proximal to ventilator), I agree with this recommendation.

2. Depending on the drug delivevered: antibiotics vs bronchodilators.

3. The table says nebulizer instead of pMDI.We should use mcg as well as % to allow comparison with other devices/situations.

4. I suggest to modify for "During invasive ventilation, metered dose inhaler and spacer are recommended to be placed in the inspiratory limb before the Y-piece for delivering bronchodilators and corticosteroids".

With the actual formulation I vote 1, with the suggested new formulation I would vote 7.

Metered dose inhaler are not recommended for antibiotic nebulization.

5. Agree, but it is my opinion. Very low level of evidence.

Round 3 (please score and comment)

**Recommendations 1-1.13**	During invasive ventilation, metered dose inhaler and spacer are recommended to be placed in the inspiratory limb before the Y-piece.
**Likert score of 1-9**	□1 □2 □3 □4 □5 □6 □7 □8 □9<br>1= absolutely disagree, 9 = absolutely agree
**Comments**	

Section 1-1: Aerosol Delivery via Invasive Ventilation for Adult Patients

Recommendation 1-1.14: During high-frequency oscillatory ventilation, nebulizers are recommended to be placed between the Y-piece and the endotracheal tube.

Distribution of voting scores

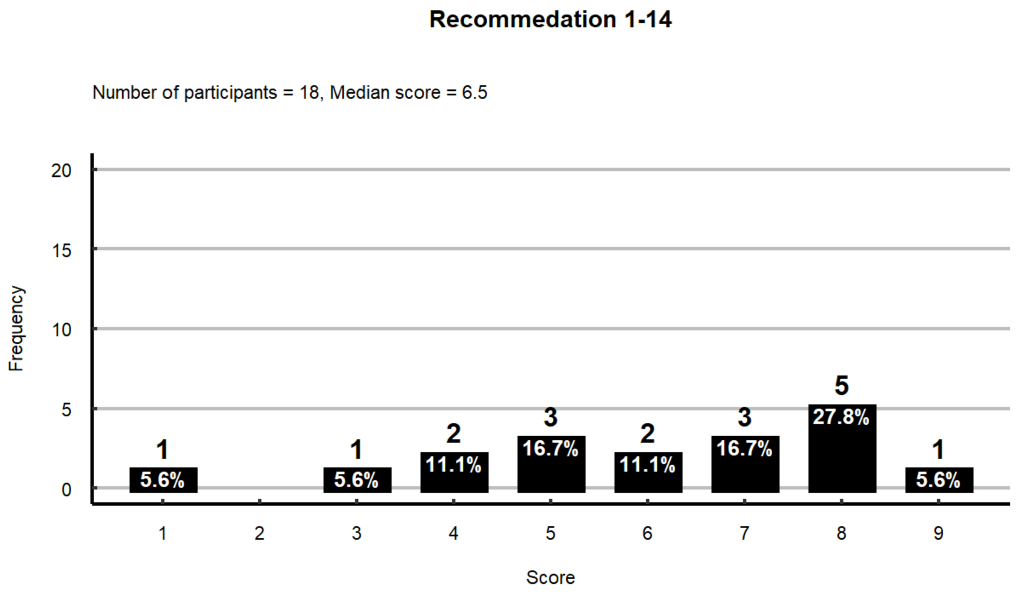

In vitro[1], in vivo[5], some consensus, weak recommendation

Comments of voting panelists

1. Evidence is insufficient to make a recommendation.

2. To be removed at next round if I understood correctly.

3. Poor evidence.

4. Lack of in vitro and in vivo studies, probably because of the limited clinical use of HFO. Therefore, is there any interest for this recommendation?

Moreover, I think that the optimal position of the nebulizer depends on the bias flow.

5. Scanty evidence.

6. Suggest delete this recommendation, as HFOV has not been used for adult patients for few years.

7. No clear evidence.

8. HFOV has not been used for adult patients commonly.

9. Should be combined with 1.2 and 1.5.

10. I suggest to modify for “During high-frequency oscillatory ventilation in preterm infants,..

With the actual formulation I vote 4, with the new formulation I would vote 7.

11. Questionable relevance. No indication for aerosol delivery with HFOV.

12. Other positions reduce inhaled dose 10 fold.

Round 3 - Recommendation Removed

**Recommendations 1-1.14**	~~**During high-frequency oscillatory ventilation, nebulizers are recommended to be placed between the Y-piece and the endotracheal tube.**~~
**Comments**	

Section 1-1: Aerosol Delivery via Invasive Ventilation for Adult Patients

Recommendation 1-1.15: The efficiency of aerosol delivery in dry ventilator circuits is higher than that in humidified ventilator circuits. Considering the potential harms of dry gas on patient airway, turning off humidifier is not recommended for routine aerosol therapy.

Distribution of voting scores

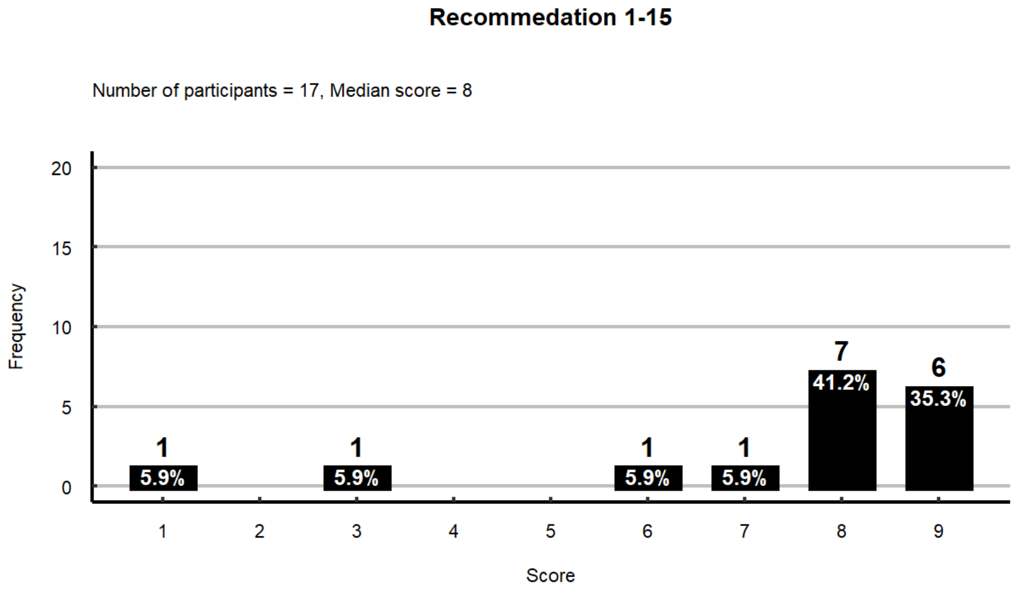

In vitro[1-11], in vivo[6,13,14], very good consensus, strong recommendation

Comments of voting panelists

1. Although humidification could reduce aerosol delivery, this could be compensated by modestly increasing the dose of the drug instead of switching off the humidifier.

2. If some experts remain in disagreement for antibiotics I suggest the following rephrasing.

The efficiency of aerosol delivery in dry ventilator circuits is higher than that in humidified ventilator circuits. Considering the potential harms of dry gas on patient airway, and the time laps required for humidifier to cool down, turning off humidifier is not recommended for routine aerosol therapy. We recommend changing the circuit if one aims at nebulizing in a dry circuit. (Ehrmann)"

3. I totally agree if we consider that the main point is the potential harms of dry gas on

patient airways.

4. For routine aerosol therapy, excluding antibiotherapy.

5. Although the efficiency……..

6. Although true for in vitro, it is not for in vitro. Please modify statement to reflect this.Missing Parker 2017 as a reference.

7. Suggested change for recommendation 1.15 "The efficiency of aerosol delivery in dry ventilator circuits is higher than that in humidified ventilator circuits. Considering that the aerosol provides a relative humidity close to 100% and that the administration of a humid gas at room air temperature for less than 1 hour does not induce significant injury of the tracheobronchial mucosa, turning off the humidifier is recommended for routine aerosol therapy".

8. Agree, but it is my opinion. Very low level of evidence.

9. Turning off humidifier for short period of time is harmless. I would not say “not recommended”, which is too strong to my mind, but choice should be left to clinicians: either turning off humidifier or not.

10. Safer to increase dose than take risk of prolonged exposure of cold dry air to lower respiratory tract.

Round 3 (please score and comment)

**Recommendations 1-1.15**	The efficiency of aerosol delivery in dry ventilator circuits is higher than that in humidified ventilator circuits. Considering the potential harms of dry gas on patient airway, **and the time lapse required for a humidifier and circuits to cool down,** turning off humidifier is not recommended for routine aerosol therapy.
**Likert score of 1-9**	□1 □2 □3 □4 □5 □6 □7 □8 □9<br>1= absolutely disagree, 9 = absolutely agree
**Comments**	

Rouby's recommendation

**Recommendations 1-1.15**	**The efficiency of aerosol delivery in dry ventilator circuits is higher than that in humidified ventilator circuits. Considering that the aerosol provides humidification of dry inspiratory gas and that ambient temperature alone does not produce tracheobronchial injury for exposures less than several hours, ~~potential harms of dry gas on patient airway, and the time lapse required for a humidifier and circuits to cool down,~~ turning off humidifier is ~~not recommended~~ a possible option for routine aerosol therapy**

Section 1-1: Aerosol Delivery via Invasive Ventilation for Adult Patients

Recommendation 1-1.16: When aerosol device is placed in the inspiratory limb, removing or bypassing the heat moisture exchanger is recommended.

Distribution of voting scores

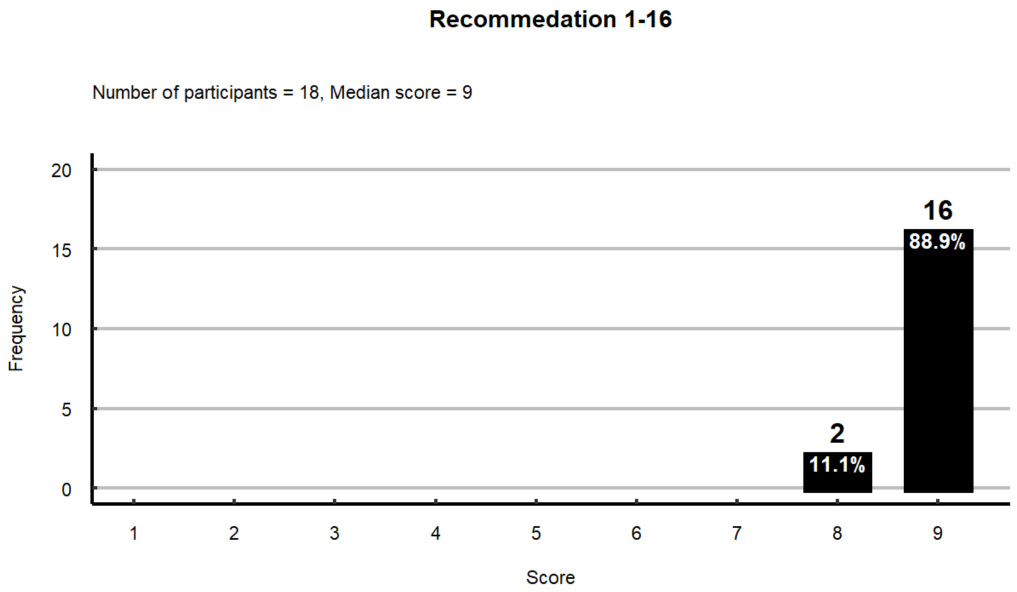

In vitro[1,2], perfect consensus, strong recommendation

Comments of voting panelists

1. This is logical but further investigation is needed to make a categorical recommendation. Devices that bypass the HME during aerosol delivery, such as Circuvent, should also be included in such a comparison.

2. I totally agree with the recommendation.

3. Absolutely agree, but low-level evidence.

<u>ROUND 3 No need to score.</u>

Section 1-1: Aerosol Delivery via Invasive Ventilation for Adult Patients

Recommendation 1-1.17: Ventilator integrated breath-actuated jet nebulizer is not preferred over continuous jet nebulizer.

Distribution of voting scores

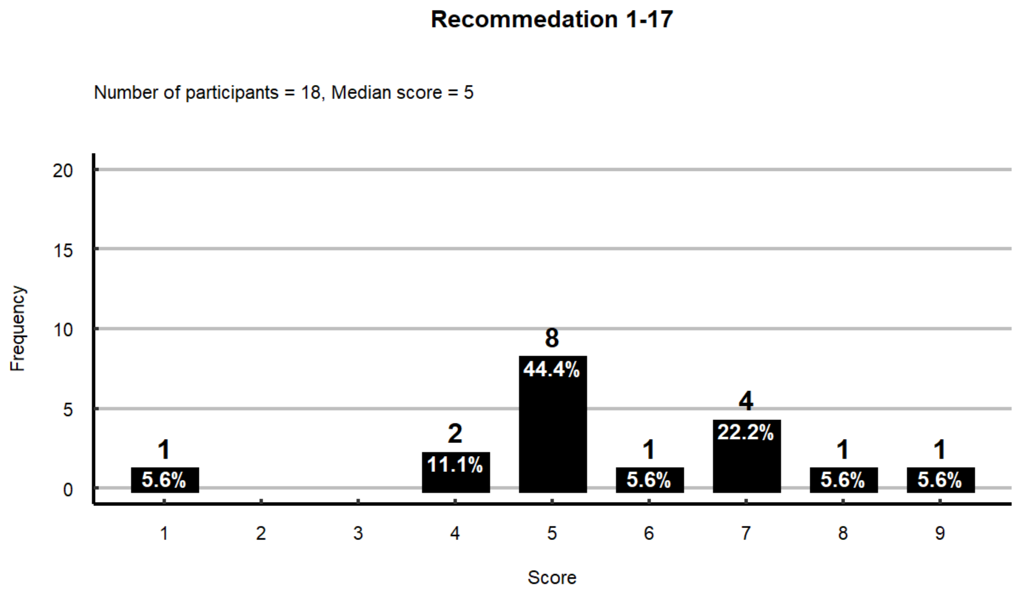

In vitro[1,2], in vivo[1], no consensus, no recommendation

Comments of voting panelists

1. In a ventilated patients, using a continuous jet nebulizer means one will use compressed gas independent of the ventilator. The effect on tidal volume, FiO2 etc… makes this practice unacceptable. The empirical compensations on ventilator settings some practitioners perform are dangerous and should be avoided. If no integrated breath system is available : no use of jet neb in a ventilated patient.

2. Disagree with the statement, but low level evidence and my opinion.

3. Results of investigations are not conclusive and further clinical studies are needed.

4. Results are discordant.

5. Nebulization of a patient on the ventilator is breath-actuated, but rather it is synchronized with the ventilator. I suggest change the name as “breath-synchronized”.

6. Further clinical studies are needed. Some ventilators do not provide adequate driving gas pressure, which may have an adverse influence on the efficiency of drug delivery

from the nebulizer. (Ehrmann S, Lyazidi A, Louis B, Isabey D, Le Pennec D, Brochard L, Apriou-Sbirlea G. Ventilator-integrated jet nebulization systems: tidal volume control and efficiency of synchronization. Respir Care 2014;59(10):1508-1516.) Therefore, I would rather agree with the recommendation.

Round 3 (please score and comment)

**Recommendations 1-1.17**	**In ventilated patients, using a continuous jet nebulizer means adding compressed gas independent of the ventilator. The effect on tidal volume, FiO2 etc makes this practice unacceptable. The empirical compensations on ventilator settings may be dangerous and should be avoided. If no integrated inspiration-synchronized jet nebulizer is available, the use of continuous jet neb in ventilated patients is not recommended.**
**Likert score of 1-9**	□1 □2 □3 □4 □5 □6 □7 □8 □9<br>1= absolutely disagree, 9 = absolutely agree
**Comments**	

Section 1-1: Aerosol Delivery via Invasive Ventilation for Adult Patients

Recommendation 1-1.18: The influence of ventilator integrated breath-actuated jet nebulizer on ventilator function and aerosol delivery efficiency varies between ventilators.

Distribution of voting scores

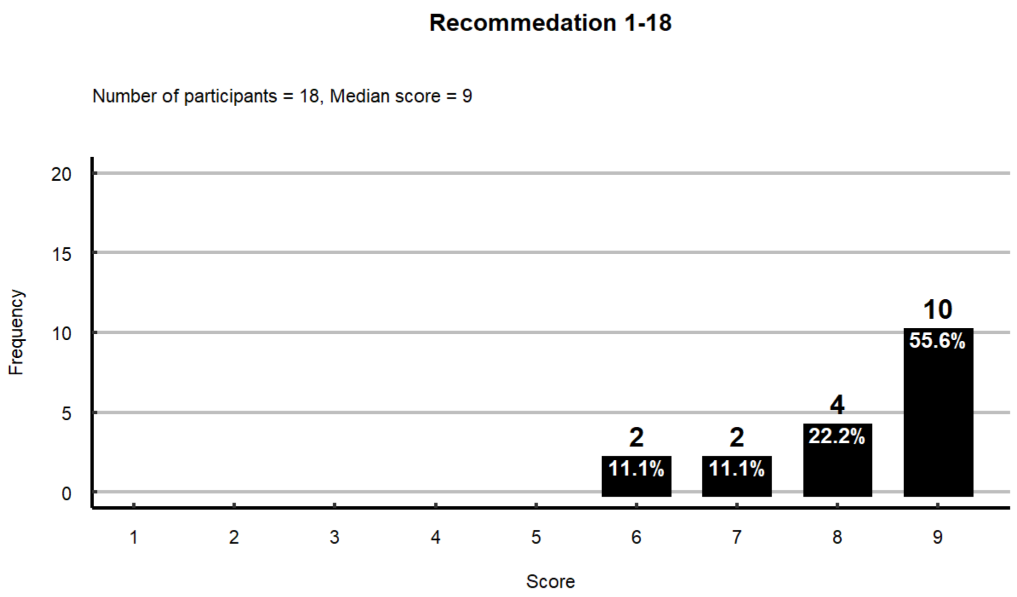

In vitro[1], very good consensus, strong recommendation

Comments of voting panelists

1. This is logical but further investigation is needed to show clinical relevance.

2. This is actually not a recommendation but just an affirmative sentence. If there is insufficient data on all ventilators we may recommend: use it only among ventilators in which the influence has been shown to be clinically insignificant and put the references so readers can go and look which are the ventilators.

3. I totally agree with the new formulation of the recommendation.

4. Low level evidence.

Round 3 Recommendation Removed -This recommendation will be moved to main text and presented as statements

**Recommendations 1-1.18**	~~**The influence of ventilator integrated breath-actuated**~~

	~~**jet nebulizer on ventilator function and aerosol delivery efficiency varies between ventilators.**~~
**Comments**	

Section 1-1: Aerosol Delivery via Invasive Ventilation for Adult Patients

Recommendation 1-1.19： Metered-dose inhaler should be primed, shaken, with actuation at the beginning of inspiration, with a minimum of 15 seconds between puffs.

Distribution of voting scores

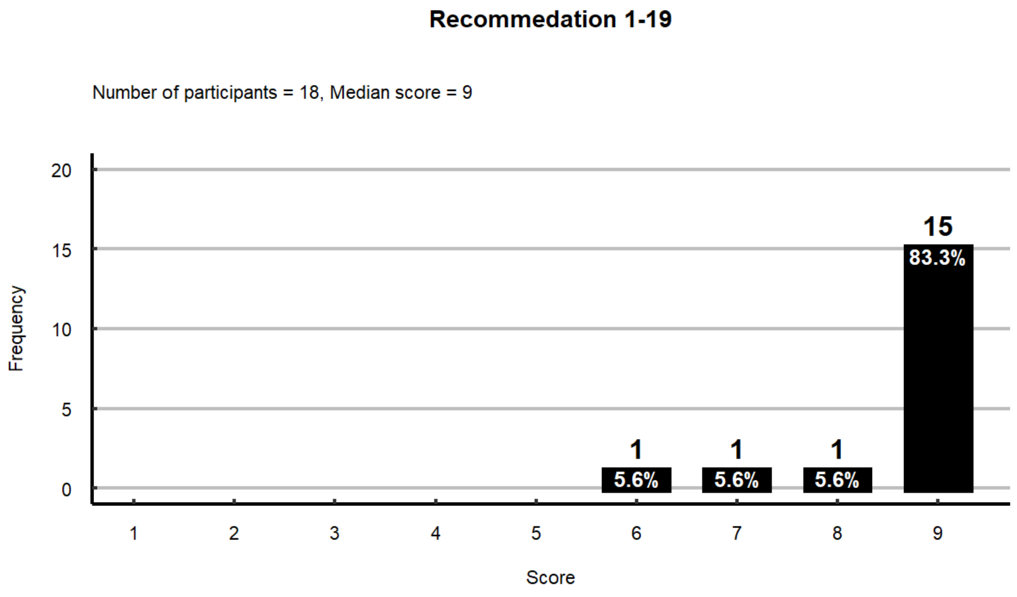

In vitro[1,2], very good consensus, strong recommendation

Comments of voting panelists

1. Please see citations of clinical studies mentioned in my earlier remarks.

2. I totally agree with the new formulation of the recommendation.

3. Warning: It does not apply to antibiotics.

4. Low level evidence.

<u>Round 3 No need to score.</u>

Section 1-1: Aerosol Delivery via Invasive Ventilation for Adult Patients

Recommendation 1-1.20.1: For the jet or ultrasonic nebulizer with a moderate residual volume, aerosol delivery efficiency is improved with a higher fill volume, but changing fill volume for the sole purpose of improving aerosol delivery efficiency is not recommended for FDA approved inhaled medication.

Distribution of voting scores

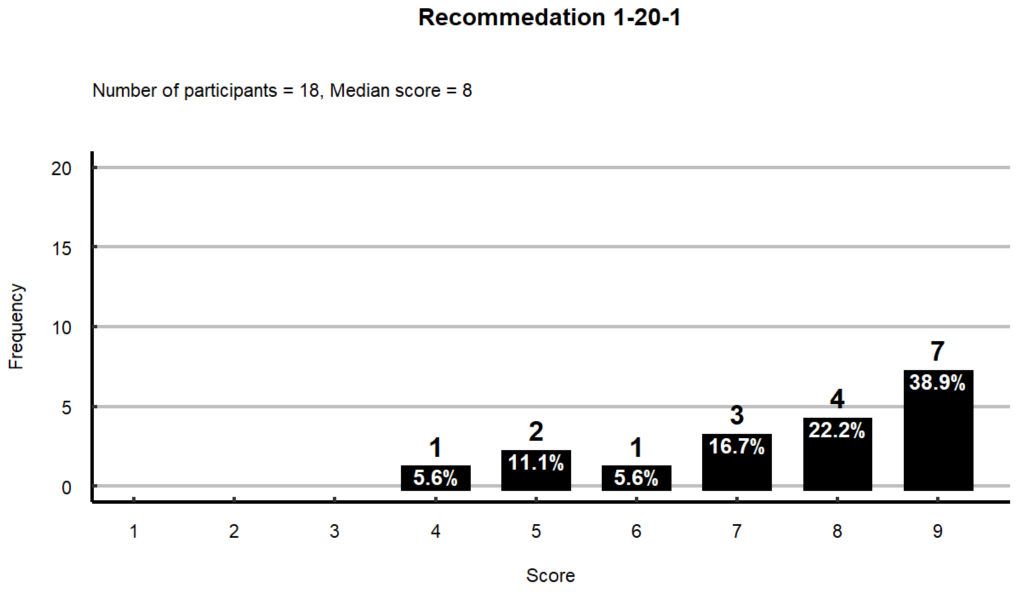

In vitro[1,2], good consensus, weak recommendation

Comments of voting panelists

1. Insufficient evidence for this recommendation.

2. Why "moderate" residual volume? I would write "with a significant residual volume".

3. Better but not sure we have enough evidence to conclude.

4. What does a nebulizer with a moderate residual volume mean? How should clinicians determine what a moderate residual volume is?

To clarify this recommendation, I would delete "with a moderate residual volume".

5. The new wording is confusing. I think you are referring to medications provided as a unit dose. Low level evidence.

Round 3 (please score and comment)

**Recommendations 1-1.20.1**	For the jet or ultrasonic nebulizer with a ~~**moderate**~~ residual volume **> 0.5 ml**, aerosol delivery efficiency is **improved** ~~**higher**~~ with a higher fill volume, but changing fill volume for the sole purpose of improving aerosol delivery efficiency is not recommended for FDA approved inhaled medication.
**Likert score of 1-9**	□1 □2 □3 □4 □5 □6 □7 □8 □9<br>1= absolutely disagree, 9 = absolutely agree
**Comments**	

Recommendation 1-1.20.2： Increasing diluent volume in vibrating mesh nebulizer to improve aerosol delivery efficiency is not recommended.

Distribution of voting scores

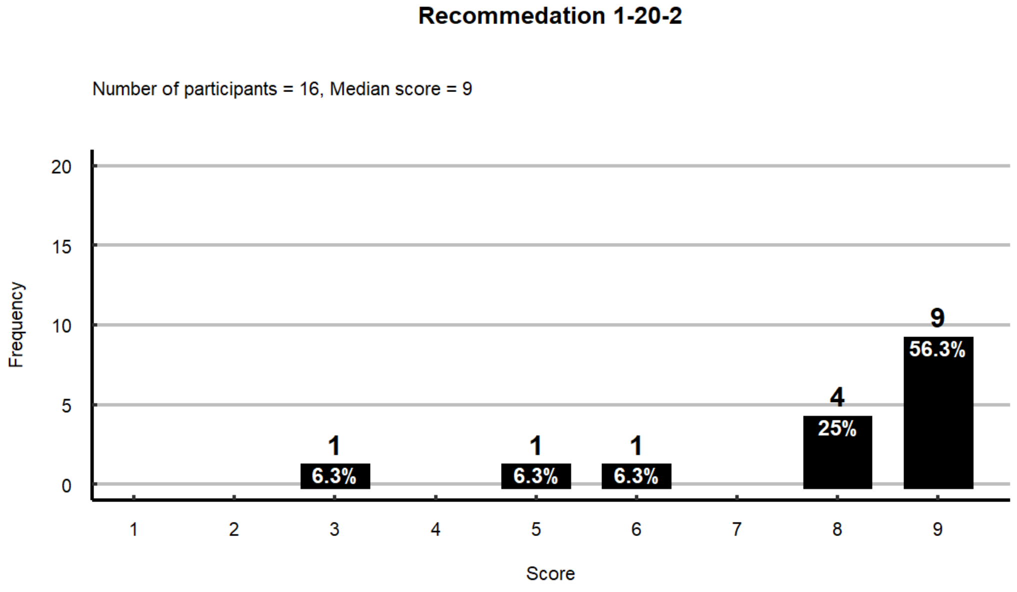

In vitro[1,2], very good consensus, strong recommendation

Comments of voting panelists

1. Considering the low residual volume of vibrating mesh nebulizer, I think there is no need to increase diluent volume to improve aerosol delivery efficiency.

More in vivo studies are needed.

I also suggest to add a note of caution: the efficiency of vibrating mesh nebulizers may be decreased with viscous drug, therefore it is necessary to dilute.

2. Evidence remains weak, based on drug. For some high viscous drugs, increasing dilution might be helpful.

3. Should be combined with the previous one.

4. Very low level evidence.

Round 3 (please score and comment)

**Recommendations 1-1.20.2**	Increasing diluent volume in vibrating mesh nebulizer to improve aerosol delivery efficiency is not recommended.
**Likert score of 1-9**	□1 □2 □3 □4 □5 □6 □7 □8 □9<br>1= absolutely disagree, 9 = absolutely agree
**Comments**	

Round 3 (please score and comment)

**Recommendations 1-1.20.3 (newly added)**	**For viscous formulations, increasing diluent volume in vibrating mesh nebulizer to improve aerosol delivery efficiency is recommended**
**Likert score of 1-9**	□1 □2 □3 □4 □5 □6 □7 □8 □9<br>1= absolutely disagree, 9 = absolutely agree
**Comments**	

Section 1-1: Aerosol Delivery via Invasive Ventilation for Adult Patients

Recommendation 1-1.21: Aerosol delivery efficiency varies between endotracheal tube and tracheotomy tube. Changing tubes for the sole purpose of improving aerosol delivery efficiency is not recommended.

Distribution of voting scores

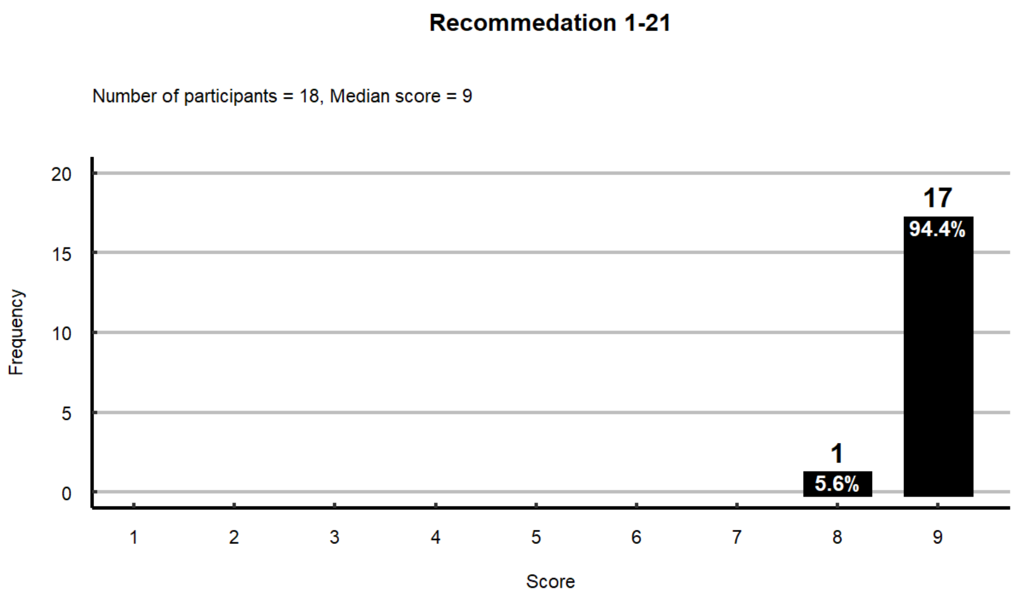

In vitro[1-4], perfect consensus, strong recommendation

Comments of voting panelists

1. This is so obvious; I don't know if there is a need for a recommendation on this.

2. I absolutely agree that the risk outweighs benefit, therefore I confirm that changing tubes for the sole purpose of improving aerosol delivery efficiency is not recommended.

3. Makes sense, but also seems ridiculous. Would anyone even consider doing trach to affect aerosol delivery.

Round 3 No need to score.

Section 1-1: Aerosol Delivery via Invasive Ventilation for Adult Patients

Recommendation 1-1.22: Aerosol delivery efficiency is higher with a large size of endotracheal tube, but changing endotracheal tube for the sole purpose of improving aerosol delivery efficiency is not recommended.

Distribution of voting scores

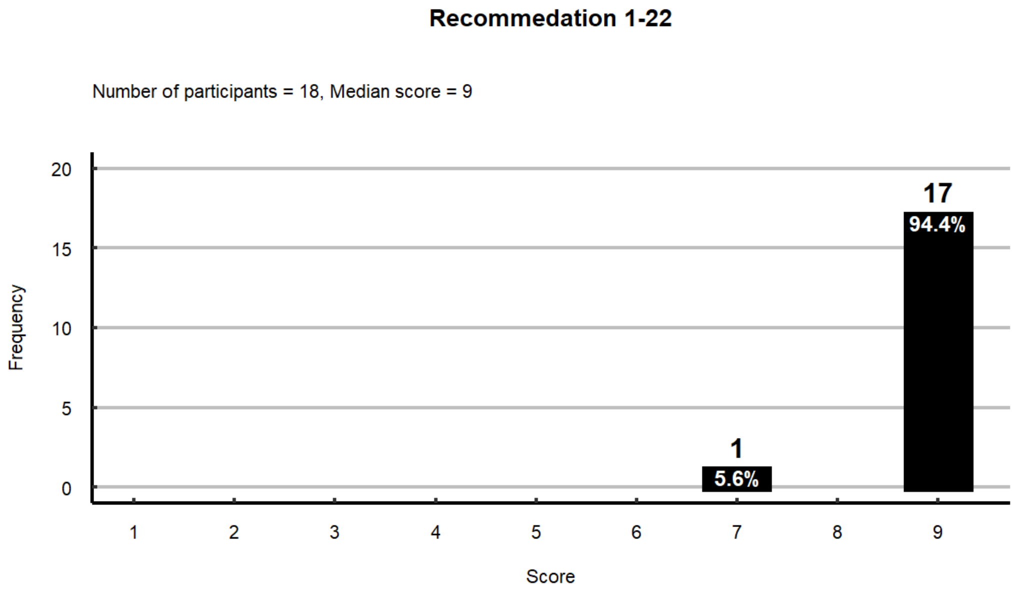

In vitro[1-3], perfect consensus, strong recommendation

Comments of voting panelists

1. There could be some debate about the first part of the statement. The lumen of the tube that is placed in the patient may not be the actual lumen depending on the presence of mucus, biofilm etc. The inspiratory flow rate and the placement of the aerosol generator also influence aerosol deposition in the tube. There is no argument about the second statement.

2. Indeed no clinical evidence for changing.

3. I absolutely agree that the risk outweighs benefit, therefore I confirm that changing the endotracheal tubes (large size) for the sole purpose of improving aerosol delivery efficiency is not recommended.

4. Agree, but would anyone ever consider reintubation to improve aerosol delivery? Seems far-fetched. Very low evidence.

<u>Round 3 no need to score.</u>

Section 1-1: Aerosol Delivery via Invasive Ventilation for Adult Patients

Recommendation 1-1.23: When heliox is utilized for invasive ventilation, aerosol delivery efficiency can improve. However, adding heliox for the sole purpose of improving aerosol delivery efficiency is not recommended.

Distribution of voting scores

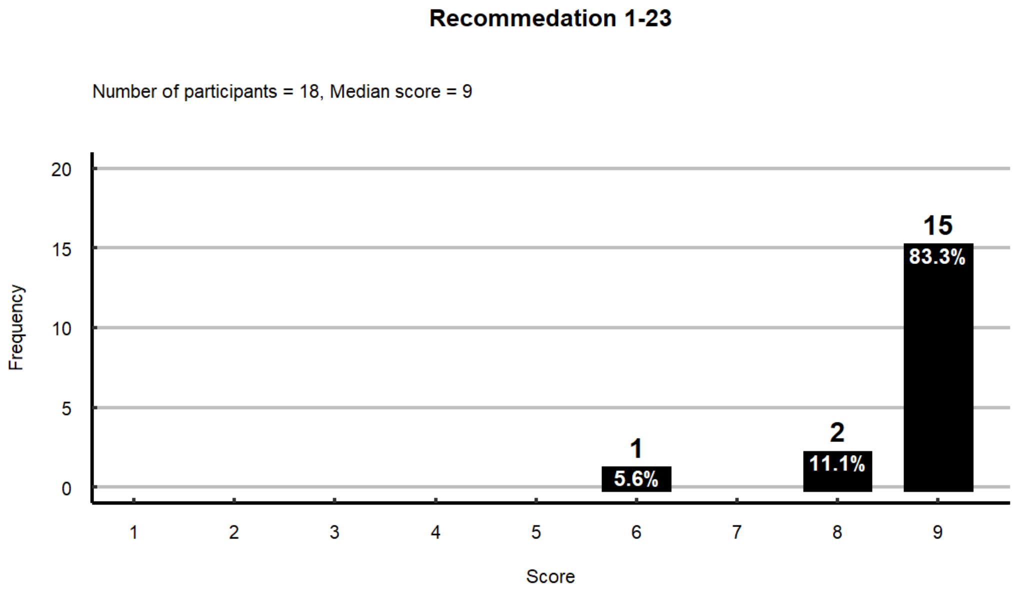

In vitro[1], very good consensus, strong recommendation

Comments of voting panelists

1. Heliox is costly, not commonly used and complicated by technical difficulties with some ventilators (e.g. alarms, calibration of tidal volume…). Therefore, I totally agree with the recommendation.

2. Warning: It does not apply to antibiotics.

3. Agree in my opinion. But very low level evidence.

Round 3 No need to score.

Section 1-1: Aerosol Delivery via Invasive Ventilation for Adult Patients

Recommendation 1-1.24： When heliox is substituted for oxygen to drive continuous jet nebulizer at the same driving flow, nebulizer output is reduced. If driving nebulizer with heliox, it is recommended to set at 15 L/min.

Distribution of voting scores

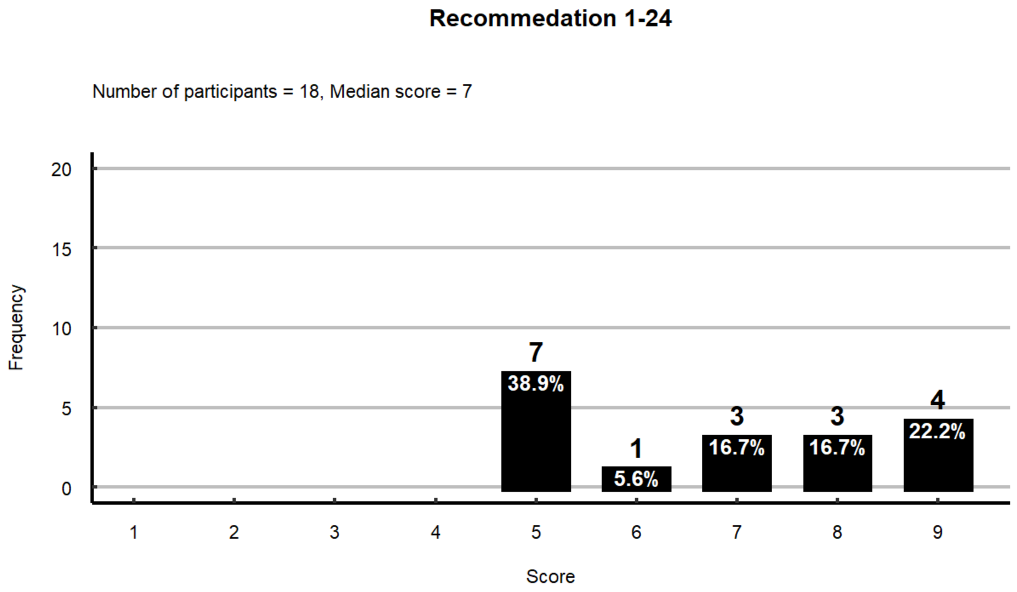

In vitro[1], good consensus, weak recommendation

Comments of voting panelists

1. The flow has to be increased from 6 to 15 L/min to have similar efficiency of nebulizer operation with a gas that is 2.5 times less dense than air or oxygen.

2. I would recommend against using heliox to drive a jet nebulizer. Too complicated, not enough evidence.

3. Weak evidence.

4. This recommendation is based on a single in vitro study. I would not make a recommendation on this.

5. Warning: It does not apply to antibiotics.

6. Scarce evidence.

7. Agree but low level evidence.

Round 3 (please score and comment)

**Recommendations 1-1.24**	When heliox is substituted for oxygen to drive continuous jet nebulizer at the same driving flow, nebulizer output is reduced. If driving nebulizer with heliox, it is recommended to set at 15 L/min.
**Likert score of 1-9**	□1 □2 □3 □4 □5 □6 □7 □8 □9<br>1= absolutely disagree, 9 = absolutely agree
**Comments**	

Section 1: Aerosol Delivery via Invasive Ventilation for Adult Patients

Recommendation 1-1.25: Spontaneous breathing may help increase aerosol delivery efficiency, but it is not recommended to change the ventilator mode for the sole purpose of improving aerosol delivery efficiency.

Distribution of voting scores

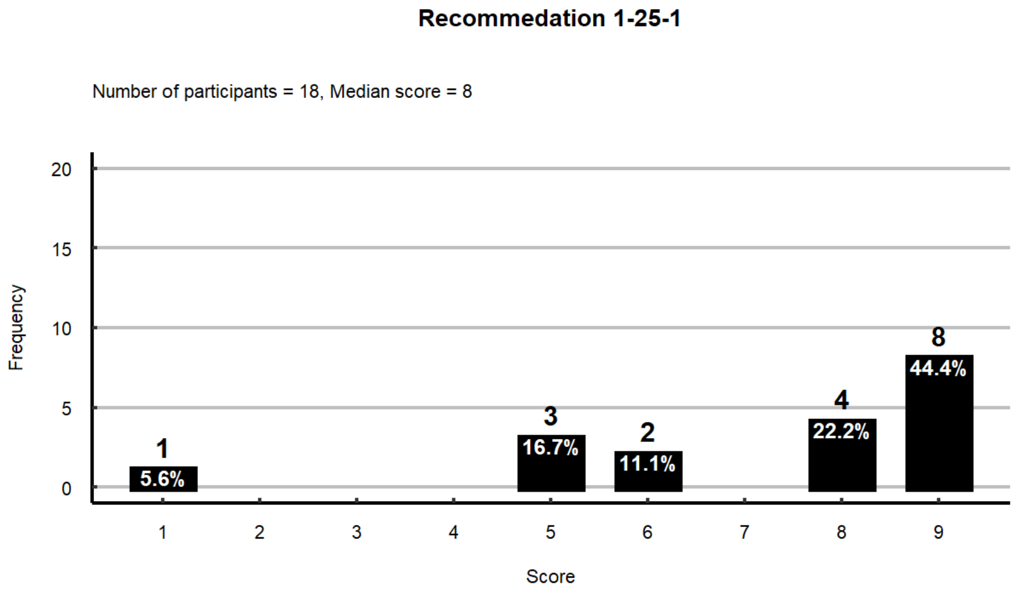

In-vitro[1,4], in vivo[2,3], some consensus, weak recommendation

Comments of voting panelists

1. There is insufficient evidence to make a categorical recommendation. I also think the statement needs some modification.

2. I don't think there is data strongly supporting this. It is in contradiction with the next recommendation.

3. The sentence could be inverted.

4. I absolutely agree that the risk outweighs benefit, therefore I confirm that changing ventilator mode for the sole purpose of improving aerosol delivery efficiency is not recommended.

5. I agree with the second part of the recommendation if it relates to bronchodilators. But there is weak evidence to support the first part of the sentence.

6. I would delete this recommendation Not logical.

7. Changing the mode does not mean allowing spontaneous breathing, patients can still have spontaneous breathing with any mode, suggest delete this recommendation.

8. “Spontaneous breathing may help increase aerosol delivery efficiency” should be deleted.

9. Very low level evidence.

Round 3 (please score and comment)

**Recommendations 1-1.25**	~~**Spontaneous breathing may help increase aerosol delivery efficiency, but**~~ It is not recommended to change the ventilator mode for the sole purpose of improving aerosol delivery efficiency.
**Likert score of 1-9**	□1 □2 □3 □4 □5 □6 □7 □8 □9<br>1= absolutely disagree, 9 = absolutely agree
**Comments**	

Section 1-1: Aerosol Delivery via Invasive Ventilation for Adult Patients

Recommendation 1-1.26 : When metered-dose inhaler is utilized during invasive mechanical ventilation, there is no recommendation on flow trigger vs pressure trigger solely for aerosol delivery.

Distribution of voting scores

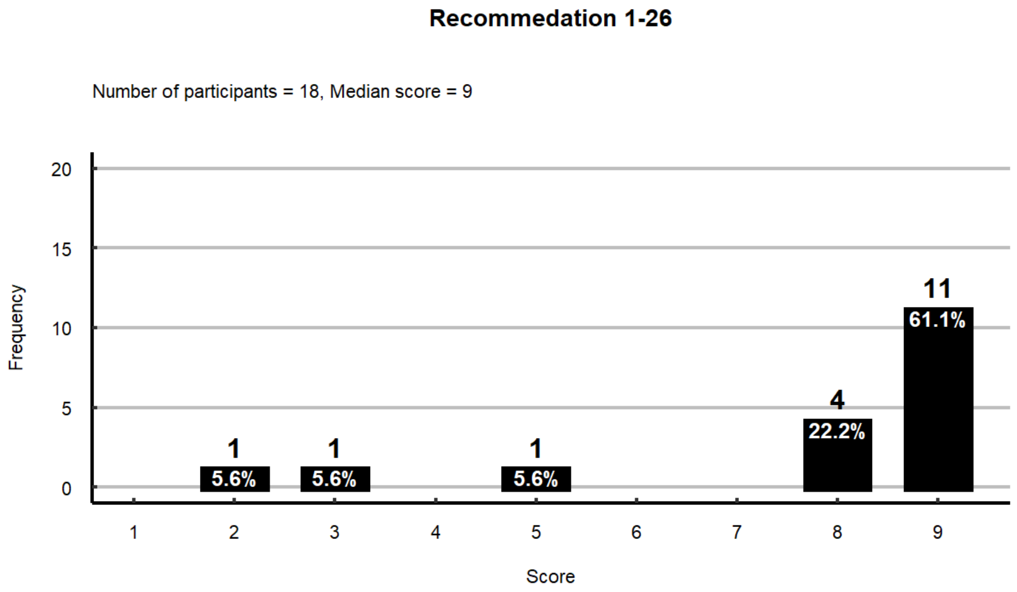

In-vitro[1], very good consensus, strong recommendation

Comments of voting panelists

1. Insufficient evidence to make a recommendation.

2. I would remove this recommendation as pressure triggers almost disappeared from ventilators.

3. Lack of in vitro and in vivo studies, probably because of the limited clinical use of pressure trigger. Therefore, is there any interest for this recommendation?

4. Comment 2 is true.

5. Suggest remove this recommendation, as pressure trigger is barely used nowadays.

6. Lack of evidence.

7. Very low level evidence.

Round 3 – Recommendation Removed

**Recommendations 1-1.26**	~~**When metered-dose inhaler is utilized during invasive mechanical ventilation, there is no recommendation on flow trigger vs pressure trigger solely for aerosol delivery.**~~
**Comments**	

Section 1-1: Aerosol Delivery via Invasive Ventilation for Adult Patients

Recommendation 1-1.27 : It is not recommended to change tidal volume and respiratory rate for the sole purpose of improving aerosol delivery efficiency.

Distribution of voting scores

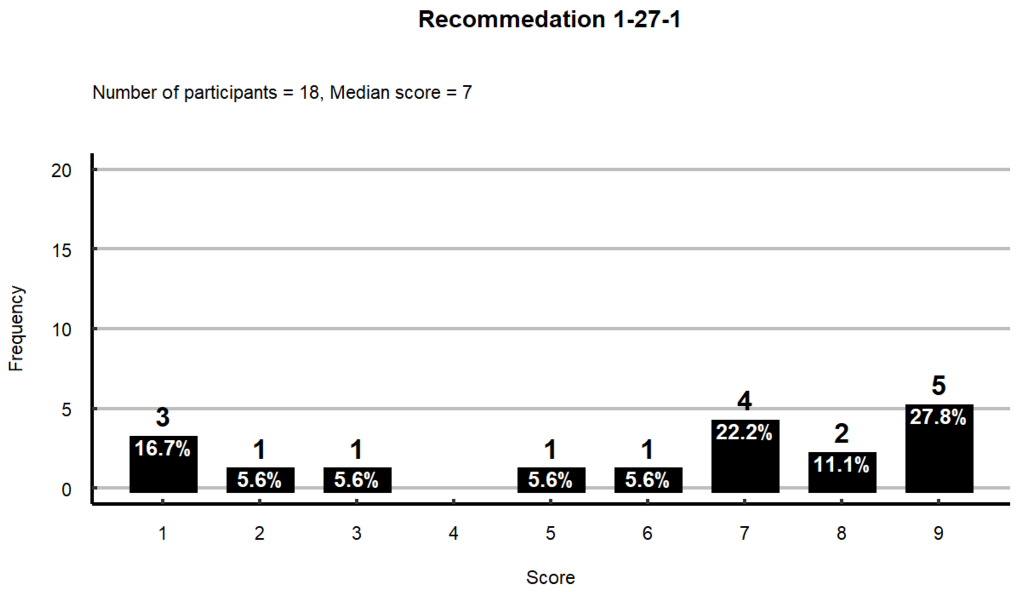

In-vivo[3] , in-vitro[1,2,4], no consensus, no recommendation

Comments of voting panelists

1. There can be some controversy over this statement (as seen in the comments of the experts). A blanket statement is not possible considering the vast number of scenarios encountered during mechanical ventilation.

2. I would add "bronchodilator aerosol delivey".

3. It could depend on the initial settings. Not for antibiotics.

4. Changing ventilator settings may have adverse effects. The ventilator settings should be adjusted to the patient's general status and the severity of respiratory insufficiency. Therefore, I agree that changing tidal volume and respiratory rate for the sole purpose of improving aerosol delivery efficiency is not recommended.

5. I do not agree.

6. Inhaled antibiotics for invasively ventilated patients may be different from bronchodilator and inhaled Glucocorticoids. Separate recommendation about mode and

parameters may be needed.

7. Agree but very low level of evidence.

8. Volume and rate needs to be set to meet needs of ventilated patients. Compromising ventilator parameters rather than just increasing dose is malpractice.

Round 3 (please score and comment)

**Recommendations 1-1.27**	It is not recommended to change tidal volume and respiratory rate for the sole purpose of improving aerosol delivery efficiency.
**Likert score of 1-9**	□1 □2 □3 □4 □5 □6 □7 □8 □9<br>1= absolutely disagree, 9 = absolutely agree
**Comments**	

Section 1: Aerosol Delivery via Invasive Ventilation for Adult Patients

Recommendation 1-1.28: Longer inspiratory time and lower inspiratory flows are associated with improved aerosol delivery efficiency, however, changing those parameters solely for aerosol delivery is not recommended.

Distribution of voting scores

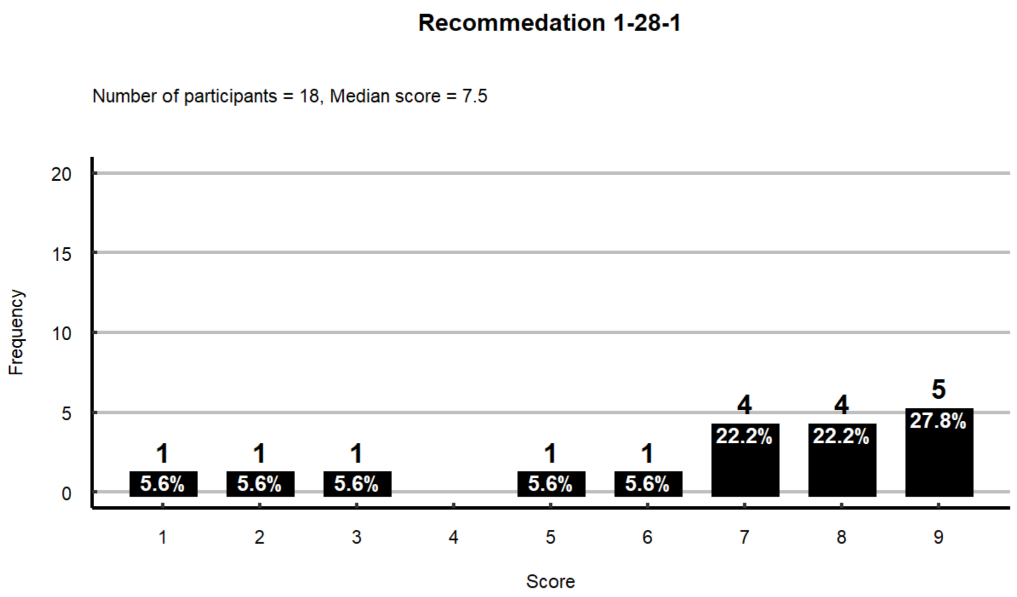

In-vitro[1-6], some consensus, weak recommendation

Comments of voting panelists

1. There is lack of agreement over this statement (as seen in the comments of the experts). A blanket statement is not possible considering the vast number of scenarios encountered during mechanical ventilation. Recommendations also differ between what drug/agent is being aerosolized.

2. I would add "bronchodilator".

3. Lengthening the inspiratory time and reducing inspiratory flows may have adverse effect. Therefore, changing those parameters solely for aerosol delivery is not recommended.

I also suggest to add a note of caution: check the persistence of end-expiratory flow at the end of the respiratory cycle on the flow-time curve which indicates the existence of auto-PEEP.

4. It would be with suboptimal atb dosing. If implemented, sedation with propofol is required.
5. I understand that some data are in favor but not so clear evidence – further changing the setting of the ventilator is always difficult in mechanically ventilated patients especially those more severe – and may discourage from using these systems for drug delivery – to be carefully discussed .
6. This should be merged with 1.27.1 It is artificial to specify for each of the ventilator settings.
7. Agree that harm outweighs benefit, but very low level evidence.

Round 3 (please score and comment)

**Recommendations 1-1.28**	~~**Longer inspiratory time and lower inspiratory flows are associated with improved aerosol delivery efficiency, however,**~~ Increasing inspiratory time and lowering inspiratory flows solely for aerosol delivery is not recommended.
**Likert score of 1-9**	□1 □2 □3 □4 □5 □6 □7 □8 □9<br>1= absolutely disagree, 9 = absolutely agree
**Comments**	

Section 1-1: Aerosol Delivery via Invasive Ventilation for Adult Patients

Recommendation 1-1.29： It is not recommended to change the inspiratory flow patterns solely for aerosol delivery.

Distribution of voting scores

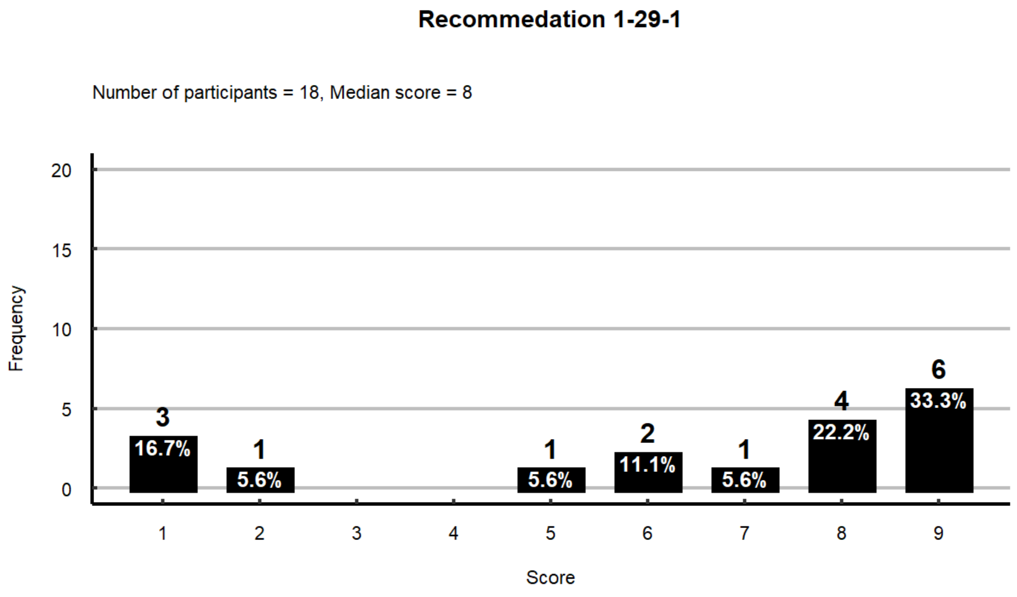

In-vitro[1,3,4], in-vivo[2], no consensus, no recommendation

Comments of voting panelists

1. There is lack of agreement over this statement (as seen in the comments of the experts). A blanket statement is not possible considering the vast number of scenarios encountered during mechanical ventilation. Recommendations also differ between what drug/agent is being aerosolized. For any inhaled therapy it is crucial to have the patient's breathing synchronized with the ventilator breath. Adjustment of the inspiratory airflow (or other ventilator settings) may be required to achieve this synchronization.

2. Too general as recommendation.

3. Weak evidence. I agree that changing ventilator mode for the sole purpose of improving aerosol delivery efficiency is not recommended.

4. I suggest adding "during controlled mechanical ventilation".

5. Agree with Ref1.

6. This should be merged with 1.27.1 and 1.28.1.

7. Agree, but very low evidence.

Round 3 (please score and comment)

**Recommendations 1-1.29**	It is not recommended to change the inspiratory flow patterns solely for aerosol delivery.
**Likert score of 1-9**	□1 □2 □3 □4 □5 □6 □7 □8 □9<br>1= absolutely disagree, 9 = absolutely agree
**Comments**	

Section 1-1: Aerosol Delivery via Invasive Ventilation for Adult Patients

Recommendation 1-1.30: It is not recommended to apply end-inspiratory pause when metered dose inhaler is used during invasive mechanical ventilation.

Distribution of voting scores

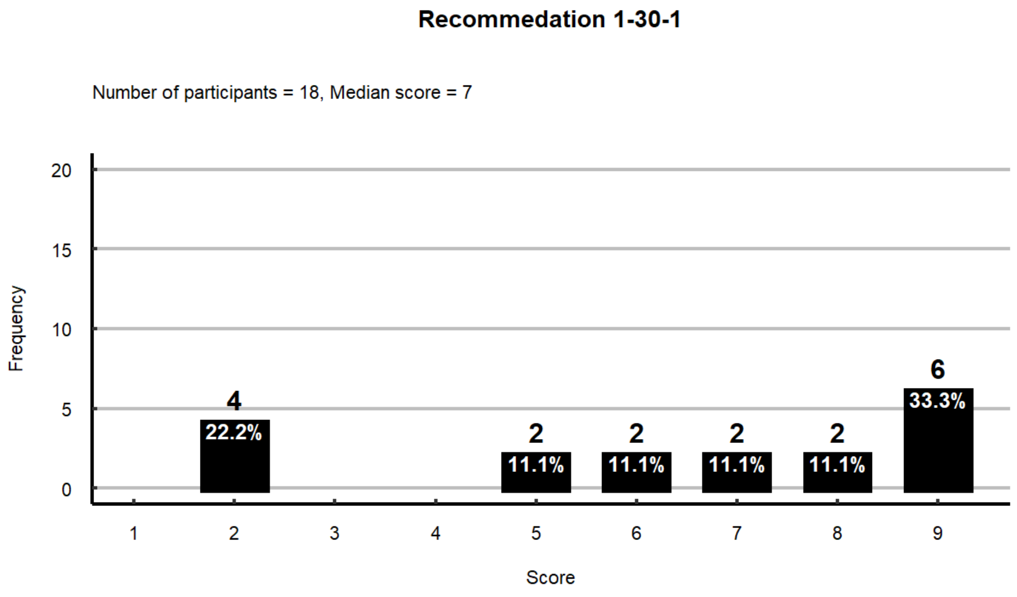

In-vivo[1], no consensus, no recommendation

Comments of voting panelists

1. It may be beneficial in achieving more peripheral deposition of aerosol when antibiotics are administered or if the lung parenchyma is being targeted. More studies are needed.

2. No reason to make different recommendation for MDI vs other aerosol generators.

3. Poor evidence. Can we really recommend that? I have the feeling we are missing evidence.

4. Limited data to recommend an end-inspiratory pause.

I suggest to specify the % or the duration of end inspiratory pause needed.

5. Warning: It does not apply to antibiotics.

6. This should be considered a change in ventilator setting and should be merged with 1.27.1, 1.28.1, and 1.29.1.

7. Agree, but low level evidence.

Round 3 (please score and comment)

**Recommendations 1-1.30**	It is not recommended to apply end-inspiratory pause when metered dose inhaler is used during invasive mechanical ventilation.
**Likert score of 1-9**	□1 □2 □3 □4 □5 □6 □7 □8 □9<br>1= absolutely disagree, 9 = absolutely agree
**Comments**	

Section 1-1: Aerosol Delivery via Invasive Ventilation for Adult Patients

Recommendation 1-1.31: It is not recommended to change the positive end-expiratory pressure (PEEP) for the sole purpose of improving aerosol delivery efficiency.

Distribution of voting scores

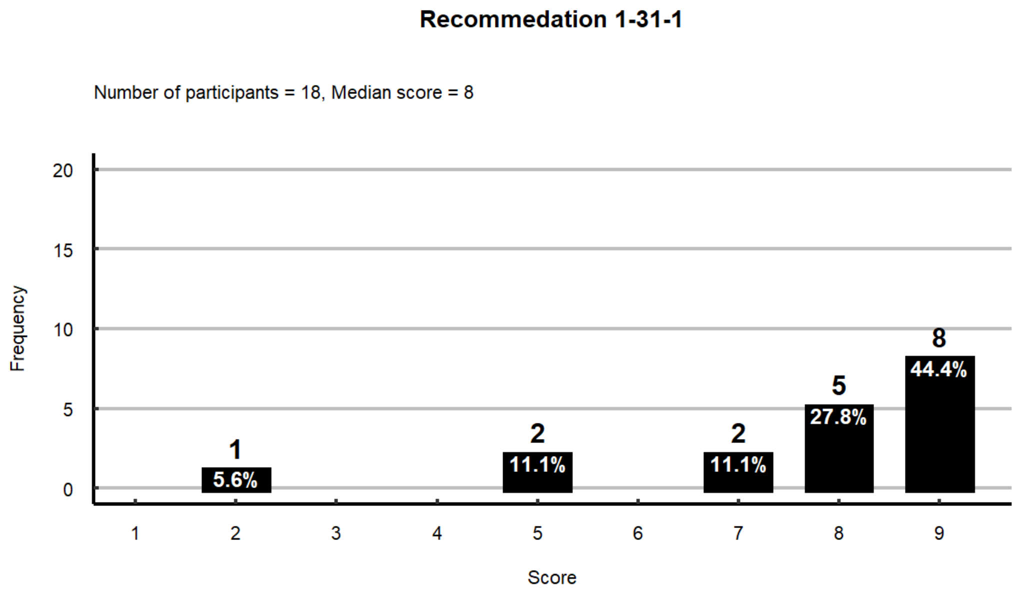

In vitro[1,3], vivo[2], very good consensus, strong recommendation

Comments of voting panelists

1. Some level of PEEP is better than zero PEEP. I agree that it should not be changed solely for enhancing aerosol delivery efficiency.

2. Add bronchodilator.

3. Potential harm outweighs benefit, therefore changing PEEP for the sole purpose of improving aerosol delivery efficiency is not recommended.

4. Warning: It does not apply to antibiotics.

5. Should be merged into one recommendation with other changes in ventilator settings.

6. Low level evidence. Potential harm outweighs benefit.

7. There is poor correlation of peep levels to deposition.

Round 3 (please score and comment)

**Recommendations 1-1.31**	It is not recommended to change the positive end-expiratory

	pressure (PEEP) for the sole purpose of improving aerosol delivery efficiency.
**Likert score of 1-9**	□1 □2 □3 □4 □5 □6 □7 □8 □9<br>1= absolutely disagree, 9 = absolutely agree
**Comments**	

Section 1-1: Aerosol Delivery via Invasive Ventilation for Adult Patients

Recommendation 1-1.32: With nebulizer placed proximal to patient, higher bias flow is associated with lower aerosol delivery efficiency. With nebulizer placed proximal to ventilator, adding bias flow up to 5 L/min improves delivery. It is recommended to set bias flow up to 5 L/min when nebulizer is placed proximal to ventilator.

Distribution of voting scores

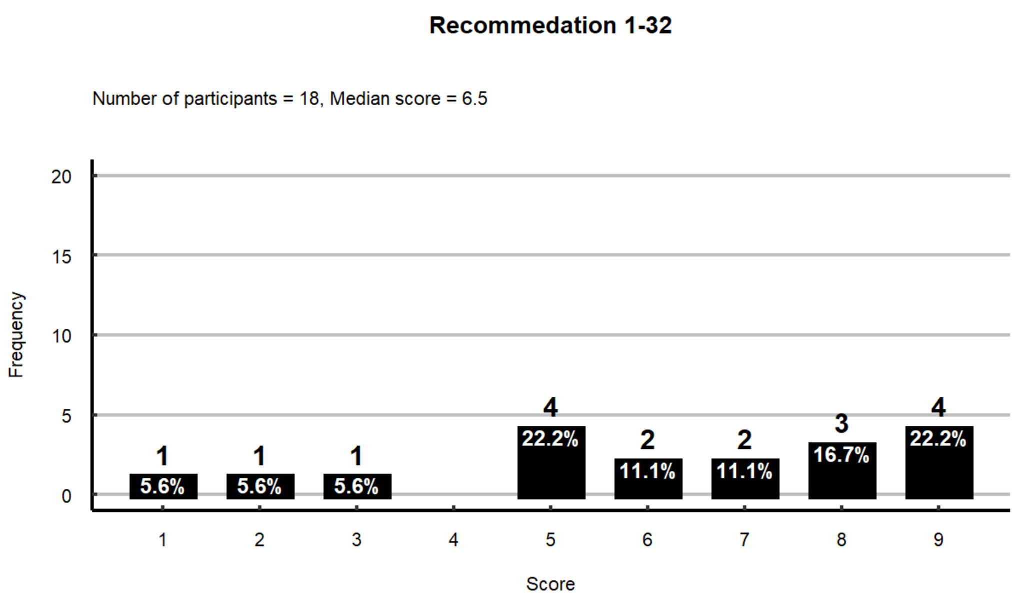

In vitro[1,2], some consensus, weak recommendation

Comments of voting panelists

1. Insufficient clinical evidence to make a recommendation.

2. Avoid proximal/distal terminology. There is an important recommendation lacking for invasive mechanical ventilation: there is a mandatory need to place a filter on the expiratory limb to protect the expiratory block and to change this filter regularely. This absolutely needs to be added.

3. In addition to my comments in the first round, I agree with the comment 5: this comment is not a recommendation for clinical use as this is not a variable that can be changed on many ventilators.

4. Not all ventilators can adjust the bias flow.

5. Most of ventilators do not allow manual changing of bias flow – please check.

6. Effects are different between jet and VMN.

7. I do not see any reason why the nebulizer should be placed proximal to ventilator (before the humidifier).

8. What is "proximal to ventilator"? Before the ventilator?! Very low level of evidence.

9. More clinical studies are needed to confirm the results of in vitro studies.

Round 3. Recommendation Removed

**Recommendations 1-1.32**	~~**With nebulizer placed proximal to patient, higher bias flow is associated with lower aerosol delivery efficiency. With nebulizer placed proximal to ventilator, adding bias flow up to 5 L/min improves delivery. It is recommended to set bias flow up to 5 L/min when nebulizer is placed proximal to ventilator.**~~
**Comments**	

Round 3 (please score and comment)

**Recommendations 1-1.33**<br>**Newly added**	**Placing a filter on the expiratory limb reduces fugitive aerosols and protects the expiratory sensors. Use of an expiratory filter with frequent changes is recommended.**
**Likert score of 1-9**	□1 □2 □3 □4 □5 □6 □7 □8 □9<br>1= absolutely disagree, 9 = absolutely agree
**Comments**	

Section 2: Aerosol Delivery via **Non-invasive Ventilation** for Adult Patients

Section 2: Aerosol Delivery via Non-invasive Ventilation for Adult Patients

Recommendation 2.1： Placing the nebulizer in-line with noninvasive ventilation has similar or higher aerosol delivery efficiency than using the nebulizer with a mask or mouthpiece. Interrupting or discontinuing noninvasive ventilation to administer aerosol via a mask or mouthpiece is unnecessary.

Distribution of voting scores

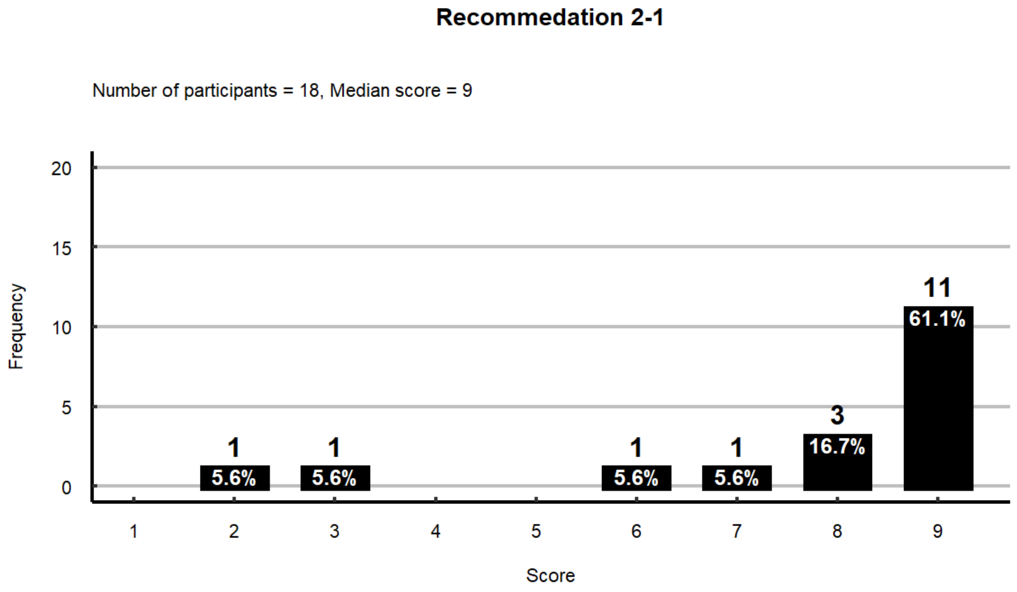

In vivo [1,3-10], in vitro[2,9], very good consensus, strong recommendation

Comments of voting panelists

1. In view of the comments, I'm not able to make a categorical statement.

2. limited clinical studies reported that inhaled bronchodilator response may be improved with the use of noninvasive ventilation in asthma. However, it's not clear whether the improvement in lung function was related solely to the effect of NIV or an additive bronchodilator effect.

If patient can tolerate withdrawal of NIV, using the nebulizer with a mask or mouthpiece is preferable.

3. The results of the included studies are conflicting, and the evidence regarding "greater efficacy of aerosol delivery with in-line nebulization compared with noninvasive ventilation than with a nebulizer using a mask or mouthpiece" is weak.

However, I agree that if patients require noninvasive ventilation, it is not necessary to interrupt noninvasive ventilation to administer aerosols.

4. Most studies show a decrease in lung deposition when niv is used. The study by laube included a pep device not NIV, this I think it needs to be removed.

5. Agree but low level of evidence.

Round 3 (please score and comment)

**Recommendations 2.1**	Placing the nebulizer in-line with noninvasive ventilation has similar or higher aerosol delivery efficiency than using the nebulizer with a mask or mouthpiece. Interrupting or discontinuing noninvasive ventilation to administer aerosol via a mask or mouthpiece is ~~**recommended**~~ **unnecessary and not recommended.**
**Likert score of 1-9**	□1 □2 □3 □4 □5 □6 □7 □8 □9<br>1= absolutely disagree, 9 = absolutely agree
**Comments**	

Section 2: Aerosol Delivery via Non-invasive Ventilation for Adult Patients

Recommendation 2.2 : During noninvasive ventilation using single limb circuit, placing metered dose inhaler with spacer between exhalation valve and mask, with actuation at the beginning of inspiration is recommended. There is no recommendation on the placement orientation (towards or away from patient) of the spacer.

Distribution of voting scores

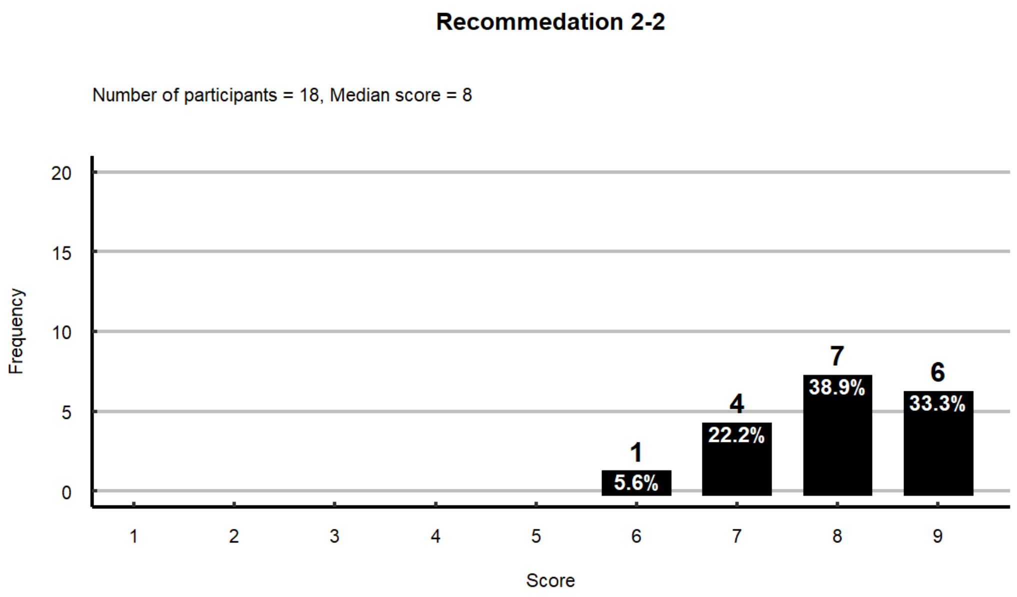

In vitro[2-4], in vivo[1,4], very good consensus, strong recommendation

Comments of voting panelists

1. Agree but low level evidence.

<u>ROUND 3 No need to score.</u>

Section 2: Aerosol Delivery via Non-invasive Ventilation for Adult Patients

Recommendation 2.3 : When placing the continuous nebulizer in-line with noninvasive ventilation, vibrating mesh nebulizer is more efficient in aerosol delivery than jet nebulizer. When available, vibrating mesh nebulizer is recommended over jet nebulizer.

Distribution of voting scores

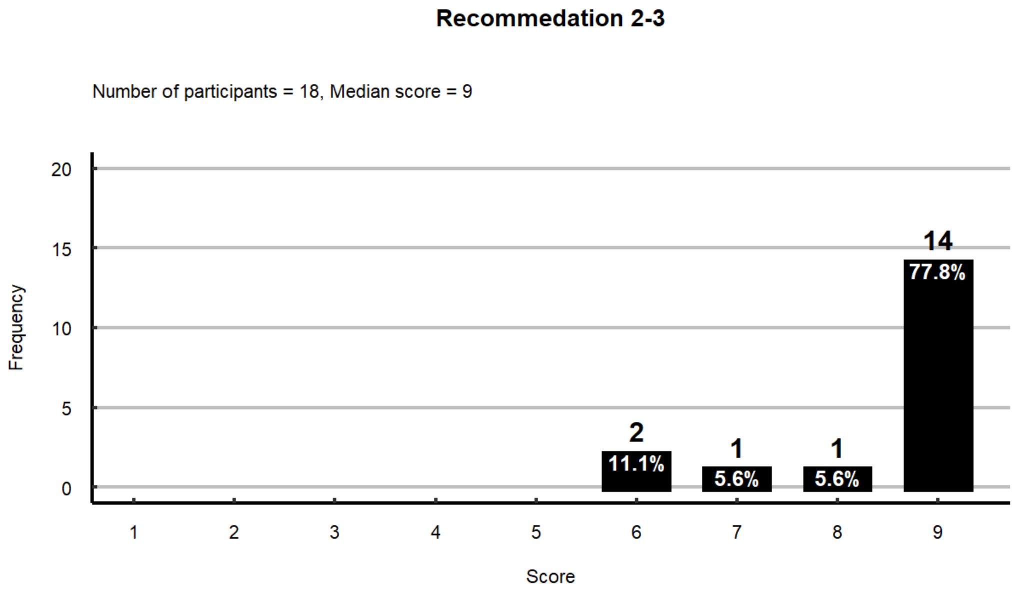

In vitro[1-3,5,7-11,13], in vivo[4,6-7,11-12], very good consensus, strong recommendation

Comments of voting panelists

1. As for the invasive part I would add the short mention on lack of interference with flow volume etc of the ventilator.

Actually on this question there is no difference between invasive and non invasive.

2. If the efficiency of nebulizers is defined by % inhaled mass and/or urinary drug, in the absence of further multi-center clinical studies, I agree with the recommendation regarding bronchodilators and most of antibiotics (question arises for high viscous drugs).

I also suggest to add a note of caution: the reliability of reusable vibrating mesh nebulizers may be decreased with repeated use over time.

- Michotte JB, Jossen E, Roeseler J, Liistro G, and Reychler G: In vitro comparison of five nebulizers during noninvasive ventilation: Analysis of inhaled and lost doses. J

Aerosol Med Pulm Drug Deliv. 2014;27:430–440.

- Gowda AA, Cuccia AD, and Smaldone GC: Reliability of vibrating mesh technology. Respir Care. 2017;62:65–69.

3. Delete when available.
4. Agree with comment about efficiency however more is not necessarily better. Also, the difference in efficiencies varies with position in the circuit.
5. Agree but low level evidence.

Round 3 (please score and comment)

**Recommendations 2.3**	When placing the continuous nebulizer in-line with noninvasive ventilation, vibrating mesh nebulizer is more efficient in aerosol delivery than jet nebulizer**, with no influence on flows or fraction of inspired oxygen.** When available, vibrating mesh nebulizer is recommended over jet nebulizer.
**Likert score of 1-9**	□1 □2 □3 □4 □5 □6 □7 □8 □9<br>1= absolutely disagree, 9 = absolutely agree
**Comments**	

Section 2: Aerosol Delivery via Non-invasive Ventilation for Adult Patients

Recommendation 2.4: During noninvasive ventilation using single limb circuit, the continuous nebulizer is recommended to be placed between the exhalation valve and the mask.

Distribution of voting scores

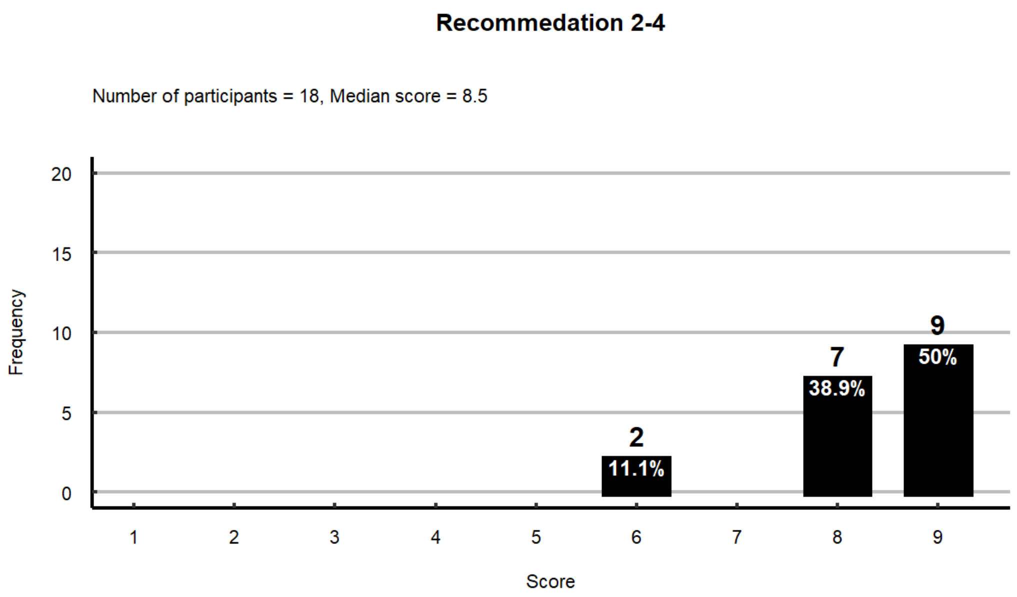

In vitro[1-9], very good consensus, strong recommendation

Comments of voting panelists

1. Need more in vivo data.

2. Further clinical studies are needed to confirm in vitro data.

3. Agree but low level evidence.

<u>Round 3 No need to score.</u>

Section 2: Aerosol Delivery via Non-invasive Ventilation for Adult Patients

Recommendation 2.5: During noninvasive ventilation using a single limb circuit, with the continuous nebulizer placed between mask and exhalation valve, there is no recommendation on the type of exhalation valve.

Distribution of voting scores

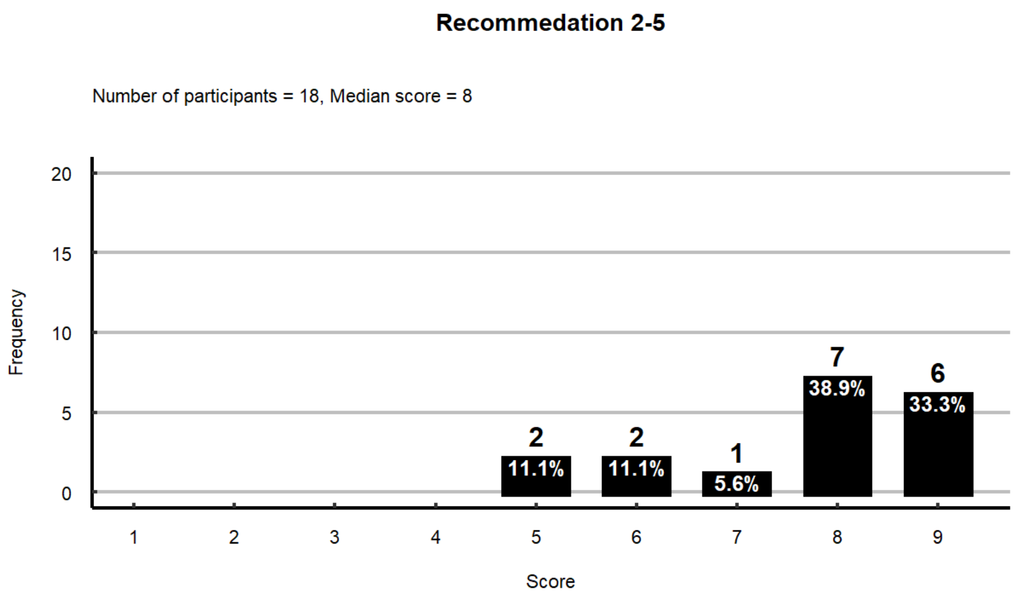

In vitro[1,2], in vivo[1], good consensus, weak recommendation

Comments of voting panelists

1. The statement needs clarification. No specific recommendation.

2. I would rather recommend something like "there is no need to change exhalation valve for the sole purpose of aerosol".

3. I agree with comment 3. It's important to specify what kind of exhalation port we are talking about.

The choice of the type of exhalation port or valve depends mainly on its performance in removing CO2.

4. Suggest delete this recommendation.

5. Question importance of this. Low level evidence.

Round 3. Recommendation Removed

**Recommendations 2.5**	~~**During noninvasive ventilation using a single limb circuit, with the continuous nebulizer placed between mask and exhalation valve, there is no recommendation on the type of exhalation valve.**~~
**Comments**	

Section 2: Aerosol Delivery via Non-invasive Ventilation for Adult Patients

Recommendation 2.6: During aerosol delivery via noninvasive ventilation, turning off the humidifier is not recommended.

Distribution of voting scores

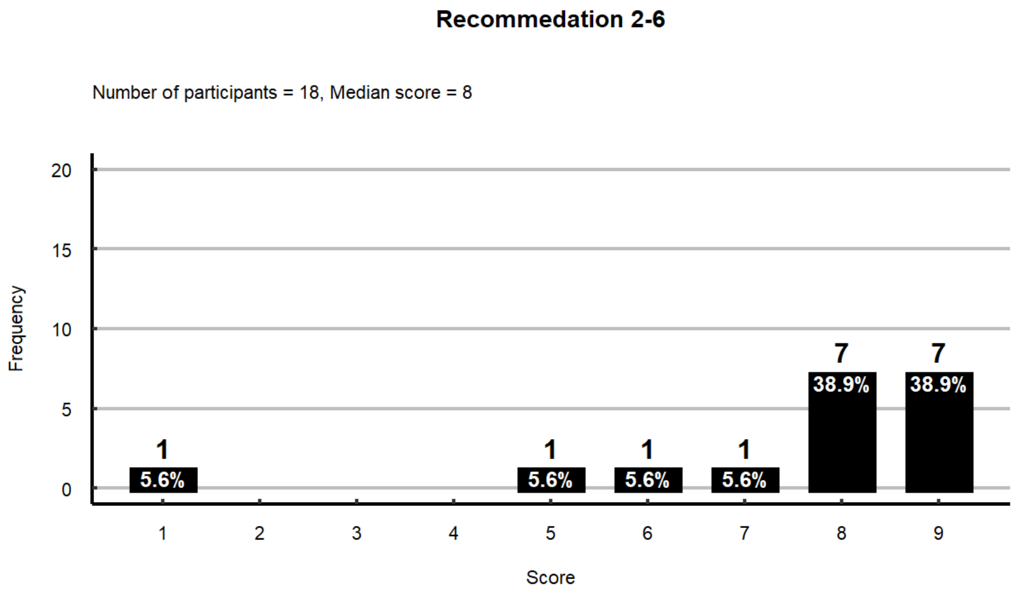

In vitro[1,2], in vivo[1], very good consensus, strong recommendation

Comments of voting panelists

1. This question needs to be investigated further. Unlike invasive mechanical ventilation, gases usually employed for NIV are not totally dry gases. Moreover, the nose is not bypassed so the relative humidity of the tracheal gas needs to be determined when the inspired gas is not fully humidified. Administration of aerosols for short period may not have as much effect on the trachea-bronchial mucosa as that occurring during invasive mechanical ventilation. Urinary albuterol levels are very crude and unreliable measures of pulmonary drug deposition and results of those studies need to be interpreted with caution

2. I agree with the new formulation of the recommendation

Considering the potential harms of dry gas on patient airway, turning off humidifier is not recommended for routine aerosol therapy.

3. I totally disagree: see my comments for recommendation 1.15.

4. Agree but low level evidence.

Round 3 No need to score.

Section 2: Aerosol Delivery via Non-invasive Ventilation for Adult Patients

Recommendation 2.7: The aerosol delivery efficiency is less affected by the fill volume in the vibrating mesh nebulizer than the continuous jet nebulizer. For continuous jet nebulizers, more dilution is associated with greater aerosol delivery. Increasing fill volume for the sole purpose to improve aerosol delivery efficiency is not recommended.

Distribution of voting scores

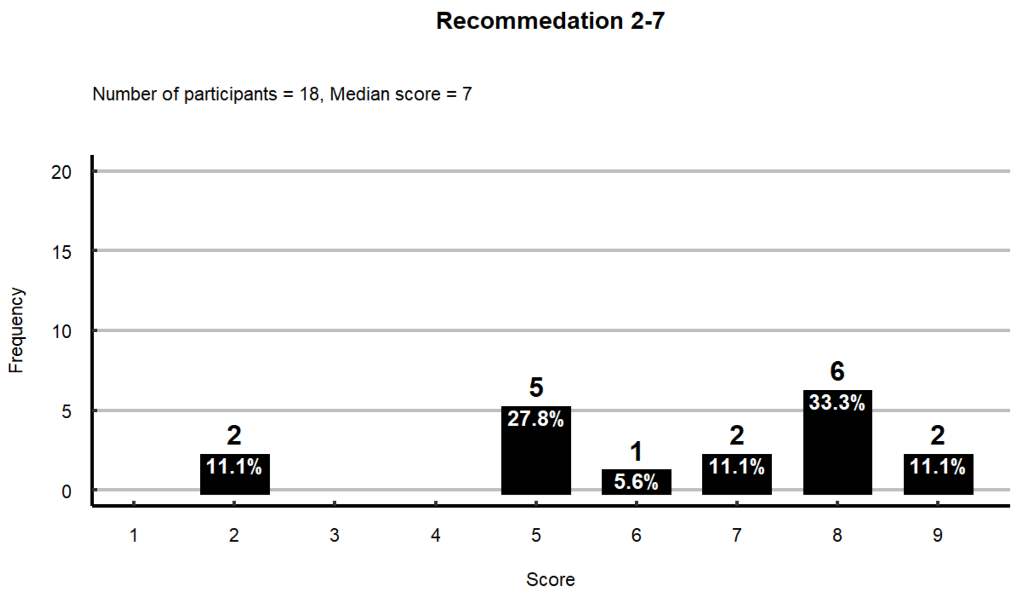

In vitro [1-4], in vivo[2,4], good consensus, weak recommendation

Comments of voting panelists

1. Unable to give a specific recommendation. The statement is directed towards aerosol delivery, but we do not know the clinical scenarios and whether these differences could be of clinical importance, e.g. when inhaled antibiotics are administered as aerosols.

2. This is not specific to non invasive ventilation.

I would just keep the general recommendation already made (may be useful but don't do it for FDA approved drugs with a specific approved volume).

3. Considering the higher residual dose of jet nebulizers (compared to vibrating mesh nebulizers), I would recommend increasing diluent volume to improve aerosol delivery when using jet nebulizers. However, more in vivo studies are needed.

4. Aerosol delivery efficiency with VMN is affected by fill volume due to viscous or high concentration drugs, whereas aerosol delivery efficiency with JN is affected by fill volume due to its high residual volume. Therefore, I do not think it can be said that VMN is less affected by dilution. It would be more accurate to state that the comparison between the 2 devices was made with respect to residual volume.

5. The study by Hess 1996 should be removed because devices were not operated during NIV. The data suggest a benefit of increasing loading volume.

6. Agree but low level evidence.

Round 3 (please score and comment)

**Recommendations 2.7**	The aerosol delivery efficiency is less affected by the fill volume in the vibrating mesh nebulizer than the continuous jet nebulizer. For continuous jet nebulizers, more dilution is associated with greater aerosol delivery. Increasing fill volume for the sole purpose to improve aerosol delivery efficiency is not recommended.
**Likert score of 1-9**	□1 □2 □3 □4 □5 □6 □7 □8 □9<br>1= absolutely disagree, 9 = absolutely agree
**Comments**	

Section 2: Aerosol Delivery via Non-invasive Ventilation for Adult Patients

Recommendation 2.8: The aerosol delivery efficiency is similar between CPAP and BiPAP, changing the noninvasive ventilation mode for the sole purpose of increasing aerosol delivery is not recommended.

Distribution of voting scores

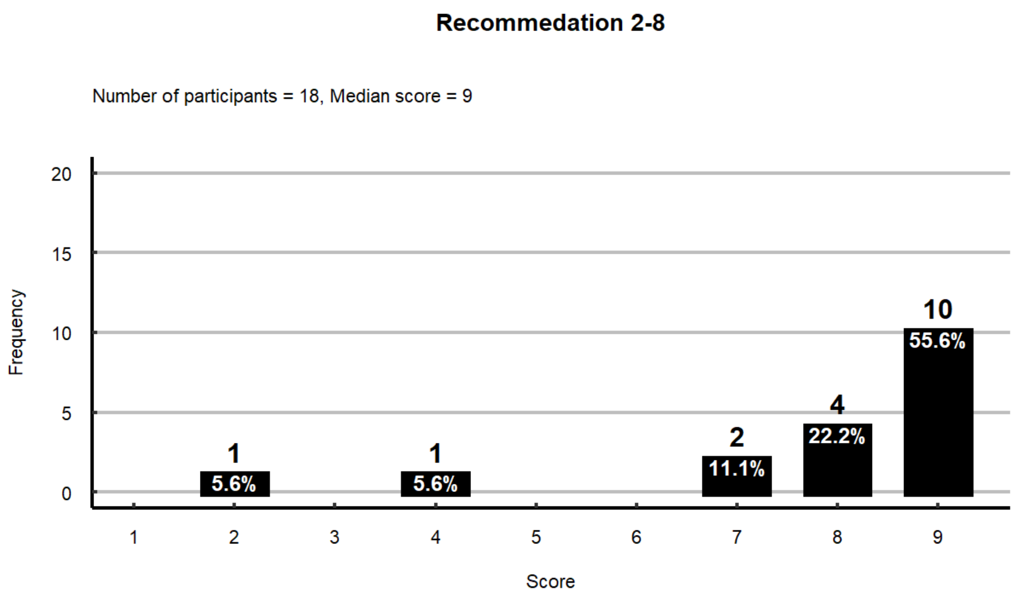

In vitro[2], in vivo[1], very good consensus, strong recommendation

Comments of voting panelists

1. Changing the mode is unlikely to make a material difference in response to an inhaled medication, unless the patient is breathing at a very low tidal volume with CPAP and change to BiPAP could improve tidal volume and drug deposition in the lung.

2. Clinically it seems not logical to change BiPAP by CPAP. Maybe it is a non sense to propose this.

3. Considering the different indications and the weak evidence, I would not recommend changing the noninvasive ventilation mode for the sole purpose of improving aerosol delivery efficiency.

4. Disagree

BIPAP better than CPAP

5. BiPAP is a brand name. Logical and I agree, but low level evidence.

Round 3 (please score and comment)

**Recommendations 2.8**	The aerosol delivery efficiency is similar between CPAP and ~~BiPAP~~ **noninvasive ventilation**, changing the ~~**noninvasive ventilation**~~ mode for the sole purpose of increasing aerosol delivery is not recommended.
**Likert score of 1-9**	□1 □2 □3 □4 □5 □6 □7 □8 □9<br>1= absolutely disagree, 9 = absolutely agree
**Comments**	

Section 2: Aerosol Delivery via Non-invasive Ventilation for Adult Patients

Recommendation 2.9: When continuous nebulizer is placed between the mask and the exhalation valve during noninvasive ventilation with a single limb circuit, the aerosol delivery efficiency increases as IPAP increases or EPAP decreases. Changing the parameters for the sole purpose to improve aerosol delivery efficiency is not recommended.

Distribution of voting scores

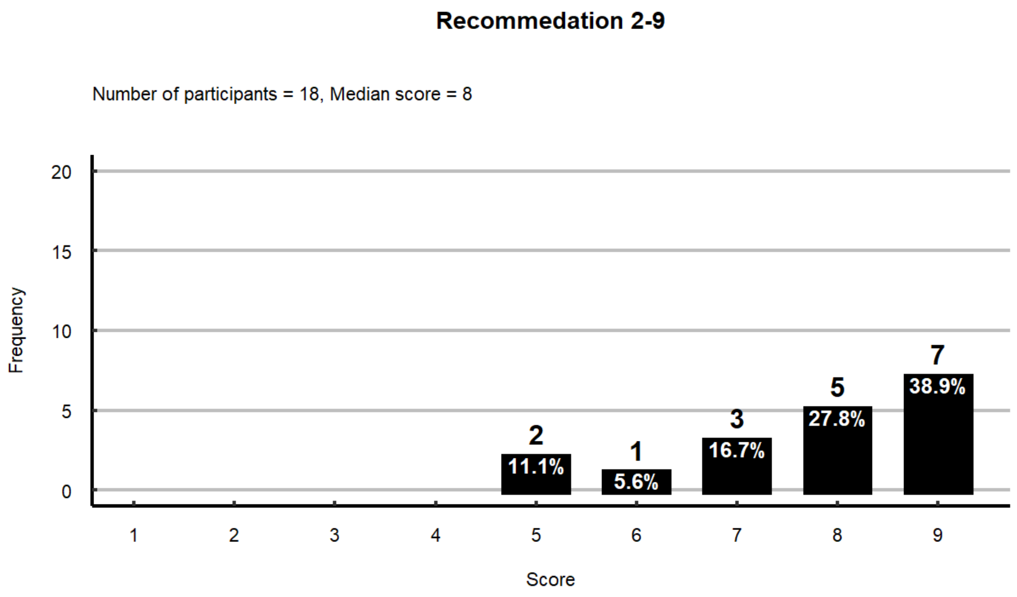

In vitro[1,3-5], in vivo[2], very good consensus, strong recommendation

Comments of voting panelists

1. There is some debate about the statement itself. No firm recommendation can be given.

2. This is true whatever the nebulizer placement. Thus I would just make a general recommendation for invasive and non invasive : except for antibiotics, don't change the ventilator settings for the sole purpose of aerosol delivery.

3. Increasing IPAP or decreasing EPAP may have adverse physiological effects (e.g hyperventilation, hypoxemia, increasing work of breathing, patient-ventilator asynchrony…).

Considering the potential harms and the risk of discomfort, I would not recommend

changing the parameters for the sole purpose to improve aerosol delivery.

4. In clinical settings during noninvasive ventilation, increasing driving pressure does not always generate higher tidal volume. Thus the parameter settings should be adjusted based on patient's clinical condition.

5. Agree, but low level evidence.

Round 3 (please score and comment)

**Recommendations 2.9**	When continuous nebulizer is placed between the mask and the exhalation valve during noninvasive ventilation with a single limb circuit, the aerosol delivery efficiency increases as IPAP increases or EPAP decreases. Changing the parameters for the sole purpose to improve aerosol delivery efficiency is not recommended.
**Likert score of 1-9**	□1 □2 □3 □4 □5 □6 □7 □8 □9<br>1= absolutely disagree, 9 = absolutely agree
**Comments**	

Section 2: Aerosol Delivery via Non-invasive Ventilation for Adult Patients

Recommendation 2.10 : When a continuous nebulizer is placed in-line with noninvasive ventilation, the aerosol delivery efficiency is higher with a non-vented mask than a vented mask. Aerosol administration with a vented mask is not recommended.

Distribution of voting scores

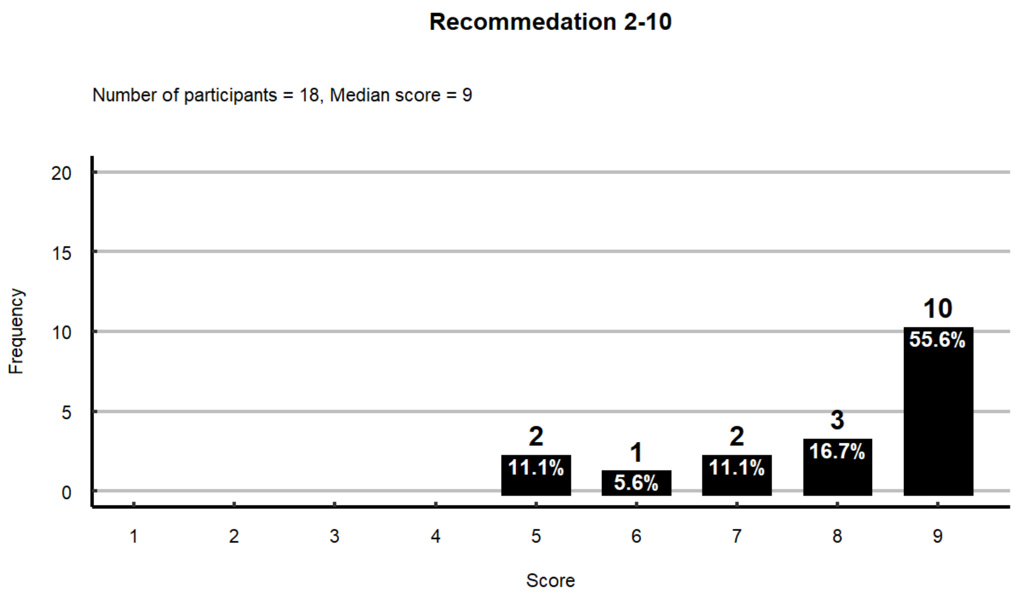

In vitro[1-3], very good consensus, strong recommendation

Comments of voting panelists

1. Need more clinical data.

2. I totally agree with the recommendation, especially to limit bioaerosol dispersion carrying pathogens.

3. Agree, but low level evidence.

Round 3 (please score and comment)

**Recommendations 2.10**	When a continuous nebulizer is placed in-line with noninvasive ventilation, the aerosol delivery efficiency is higher with a non-vented mask than a vented mask. Aerosol administration with a vented mask is not recommended.

**Likert score of 1-9**	□1 □2 □3 □4 □5 □6 □7 □8 □9<br>1= absolutely disagree, 9 = absolutely agree
**Comments**	

Section 2: Aerosol Delivery via Non-invasive Ventilation for Adult Patients

Recommendation 2.11 : When non-vented mask is used during noninvasive ventilation, the aerosol delivery efficiency with optimal position is similar with the single limb and dual limb circuits. There is no recommendation for the use of single versus dual limb circuits for aerosol delivery.

Distribution of voting scores

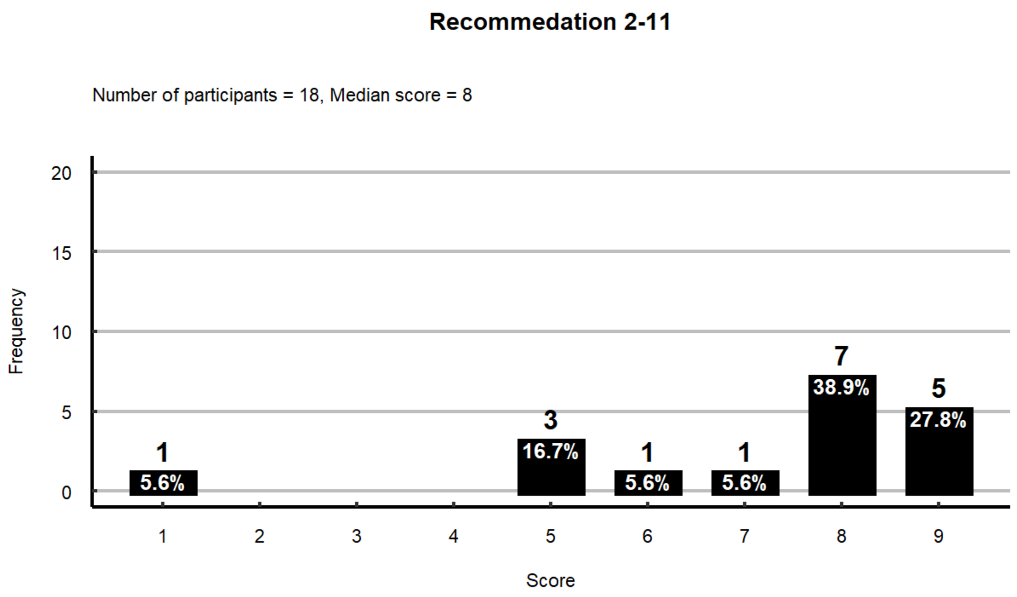

In vitro[1], some consensus, weak recommendation

Comments of voting panelists

1. Need more clinical data.

2. Dual limb are much easier to understand than single limb in terms of were the aerosol may be lost. + they benefit of all the evidence from invasive ventilation concerning position etc… I would not be against a recommendation favoring dual limb as there is more evidence.

3. More evidence is needed.

4. Only one in vitro study, so no conclusions can be drawn regarding similar efficacy of aerosol delivery between the single and double limb. I suggest deleting this sentence. I agree with the second sentence ""no recommendation".

5. Studies under review at the time of 1st round should not be included.

6. Agree, but low level evidence.

Round 3 (please score and comment)

**Recommendations 2.11**	When non-vented mask is used during noninvasive ventilation, the aerosol delivery efficiency with optimal position is similar with the single limb and dual limb circuits. There is no recommendation for the use of single versus dual limb circuits for aerosol delivery.
**Likert score of 1-9**	□1 □2 □3 □4 □5 □6 □7 □8 □9<br>1= absolutely disagree, 9 = absolutely agree
**Comments**	

Section 3: Aerosol Delivery via **High-flow Nasal Cannula** for Adult Patients

Section 3: Aerosol Delivery via High-flow Nasal Cannula for Adult Patients

Recommendation 3.1： The aerosol delivery efficiency with a nebulizer via high-flow nasal cannula is similar to that with a nebulizer and a mask or mouthpiece. Discontinuing high-flow nasal cannula treatment to administer nebulizer with a mask or mouthpiece is not recommended.

Distribution of voting scores

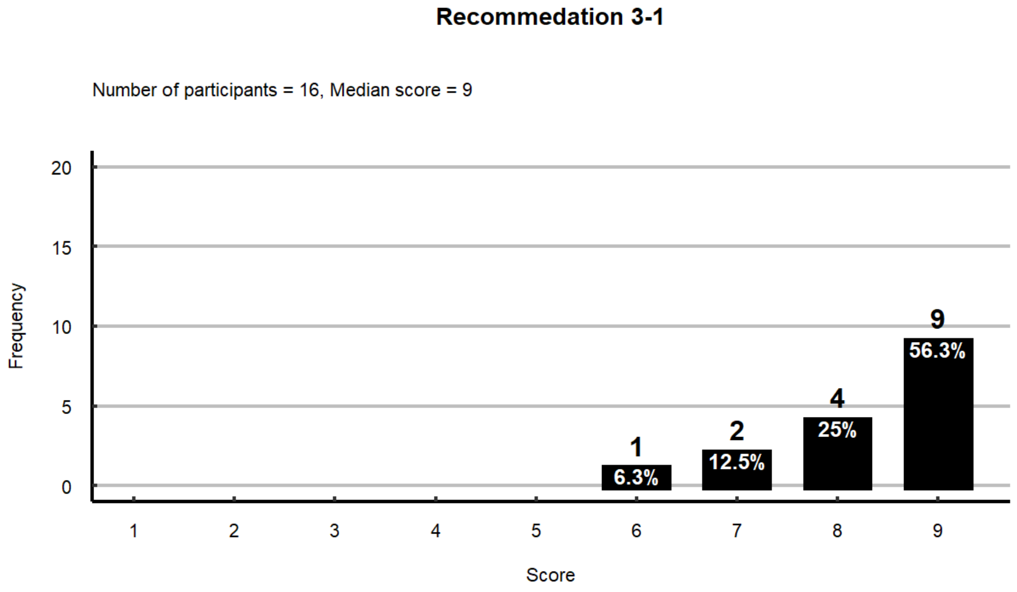

In vivo [1-4], in vitro[5], very good consensus, strong recommendation

Comments of voting panelists

1. With the appropriate technique the response to bronchodilators is shown both in stable patients and in patients with acute exacerbations. More clinical studies would be helpful

I would qualify the statement to include vibrating mesh nebulizer.

2. Only true for bronchodilators : needs to be specified in the recommendation.

3. If it is tolerated by the patient, it is preferable discontinuing high-flow nasal cannula to administer aerosol via a mask or mouthpiece.

Limited in vitro and in vivo data with flow rate > 35 L/min.

4. In patients with acute respiratory distress, a high flow rate >40 L/min is very often

required. Higher flow rates produce greater turbulence which decreases the efficiency of aerosol delivery.

Therefore, the efficiency of aerosol delivery with a nebulizer via a high-flow nasal cannula is similar to that of a nebulizer via a mask or mouthpiece if the high flow rate is less than 40 L/min.

5. Is totally contraindicated.

6. In information in the table does not follow previous format.

Round 3 (please score and comment)

**Recommendations 3.1**	The aerosol delivery efficiency with a nebulizer via high-flow nasal cannula **at flow ≤ 35 L/min** is similar to that with a nebulizer and a mask or mouthpiece. Discontinuing high-flow nasal cannula treatment to administer nebulizer with a mask or mouthpiece is not recommended.
**Likert score of 1-9**	□1 □2 □3 □4 □5 □6 □7 □8 □9<br>1= absolutely disagree, 9 = absolutely agree
**Comments**	

Section 3: Aerosol Delivery via High-flow Nasal Cannula for Adult Patients

Recommendation 3.2: Placing a nebulizer with a mask or mouthpiece on a patient who is using concurrent high-flow nasal cannula treatment is not recommended.

Distribution of voting scores

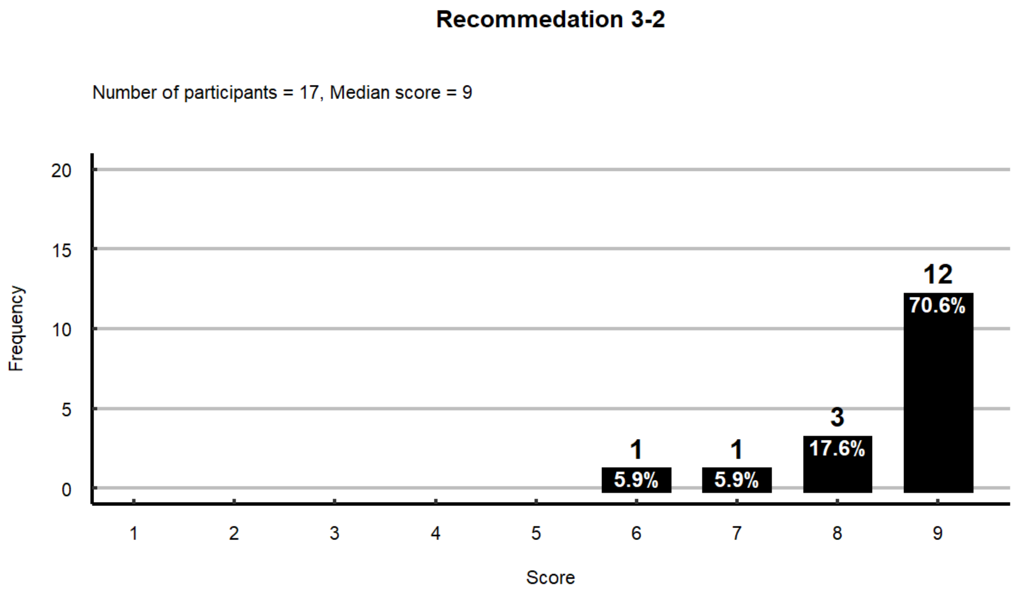

In vitro[1], very good consensus, strong recommendation

Comments of voting panelists

1. For sake of clarity, and to differentiate it from statement in 3.1, the statement could be revised as mentioned above (see highlighted text).

2. Data are needed to support this recommendation, especially when placing a nebulizer with a mask (reservoir effect ???).

Using an aerosol mask with concurrent high-flow nasal cannula treatment may increase bioaerosol dispersion carrying pathogens.

3. Agree, but low level evidence.

Round 3 No need to score.

Section 3: Aerosol Delivery via High-flow Nasal Cannula for Adult Patients

Recommendation 3.3: During aerosol delivery via high-flow nasal cannula, vibrating mesh nebulizer is more efficient in aerosol delivery than jet nebulizer, with no influence on flows or fraction of inspired oxygen. Vibrating mesh nebulizer is recommended for trans-nasal aerosol delivery.

Distribution of voting scores

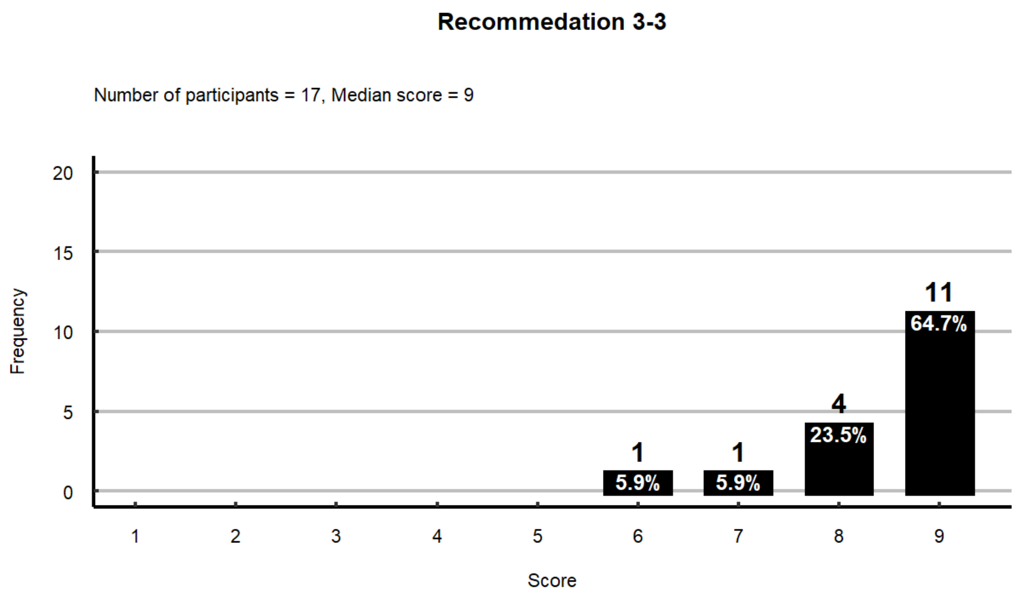

In vitro[1], in vivo[2,3], very good consensus, strong recommendation

Comments of voting panelists

1. We need more data to show that these differences are clinically meaningful to make a firm recommendation.

2. If the efficiency of nebulizers is defined by the inhaled dose, in the absence of further multi-center clinical studies, I agree with the recommendation regarding bronchodilators and most of antibiotics (question arises for high viscous drugs).

3. Agree, but low level evidence.

<u>Round 3 No need to score.</u>

Section 3: Aerosol Delivery via High-flow Nasal Cannula for Adult Patients

Recommendation 3.4 : Nebulizers are recommended to be placed at the inlet of humidifier at HFNC flows ≥ 10 L/min.

Distribution of voting scores

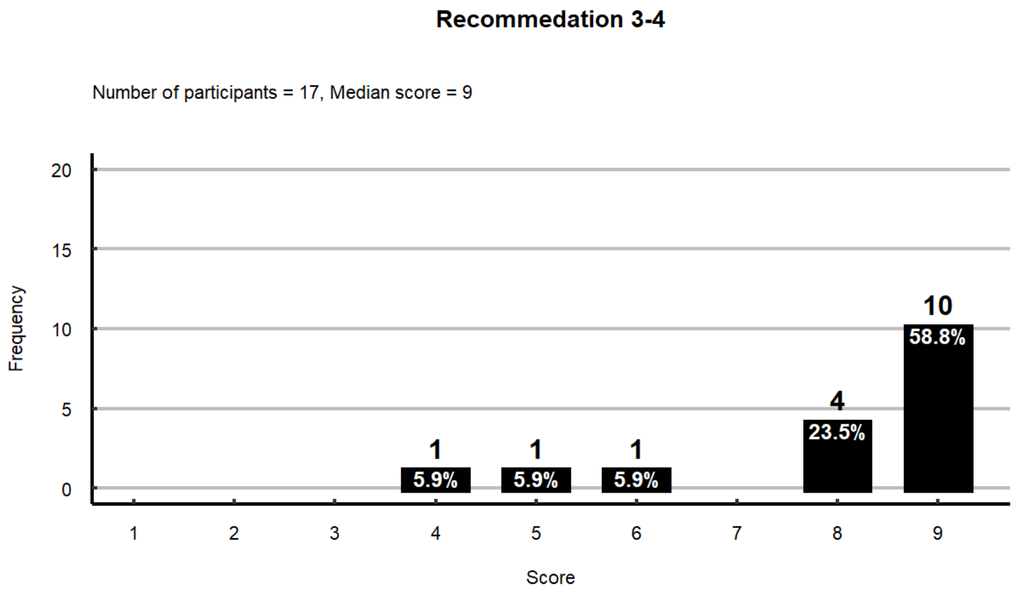

In vitro[1,2], very good consensus, strong recommendation

Comments of voting panelists

1. The comments about HFNC flow at flows <10 L /min are valid. Recommend changing the statement according to the suggestions made.

In general, compound statements should be avoided because the level of agreement (or disagreement) with the 2 parts of the statement could differ .

2. I agree with the reformulation of the recommendation.

However, more clinical data are needed, especially with flow rate > 30-40 L/min (close to most clinical situations).

3. Very low evidence.

Round 3 (please score and comment)

**Recommendations 3.4**	Nebulizers are recommended to be placed at the inlet of humidifier at HFNC flows ≥ 10 L/min.

**Likert score of 1-9**	□1 □2 □3 □4 □5 □6 □7 □8 □9<br>1= absolutely disagree, 9 = absolutely agree
**Comments**	

Section 3: Aerosol Delivery via High-flow Nasal Cannula for Adult Patients

Recommendation 3.5: When metered dose inhaler is placed in-line with high-flow nasal cannula, it is recommended to be used with a spacer and placed close to nasal cannula with the aerosol plume directed toward the patient.

Distribution of voting scores

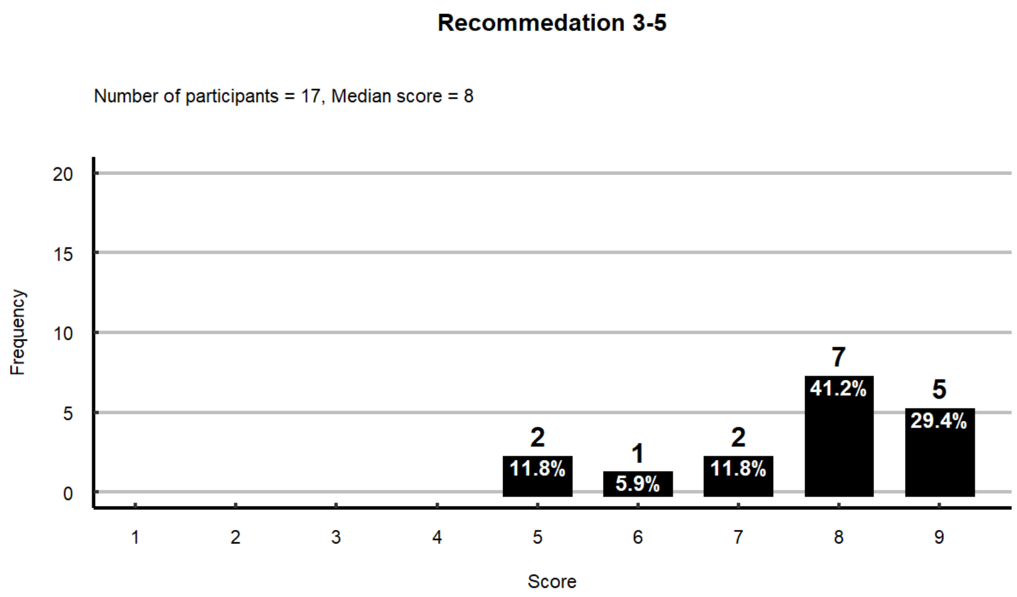

In vitro[1], very good consensus, strong recommendation

Comments of voting panelists

1. Agree with comments that more data are needed. Information so far is more towards efficiency of aerosol delivery.
2. More clinical studies are needed.
3. Question relevance. Does anyone use pMDI with HFNC?

Round 3 (please score and comment)

**Recommendations 3.5**	When metered dose inhaler is placed in-line with high-flow nasal cannula, it is recommended to be used with a spacer and placed close to nasal cannula with the aerosol plume directed toward the patient.
**Likert score of 1-9**	□1 □2 □3 □4 □5 □6 □7 □8 □9

	1= absolutely disagree, 9 = absolutely agree
**Comments**	

Section 3: Aerosol Delivery via High-flow Nasal Cannula for Adult Patients

Recommendation 3.6： To optimize aerosol delivery via high-flow nasal cannula, gas flow is recommended to be titrated below the patient's peak inspiratory flow if tolerated.

Distribution of voting scores

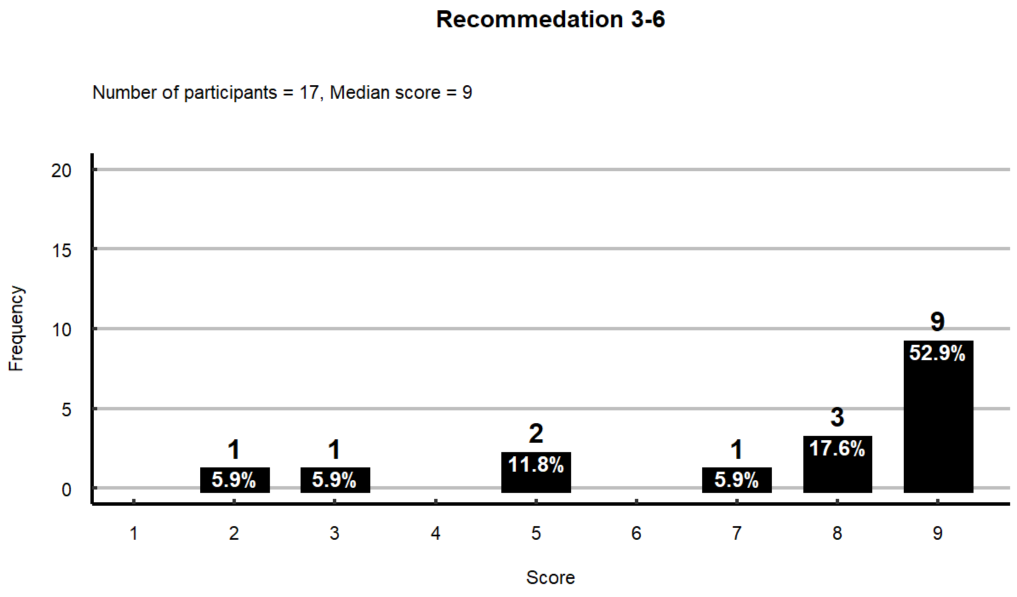

In vitro[1-6], in vivo[7,8], good consensus, weak recommendation

Comments of voting panelists

1. What improves aerosol delivery is not being below the patients peak inspiratory flow but having a low flow. So if tolerated I would recommend to set the flow down to 40 L/min (this would be in line with the recommendation for the inspiratory flow of invasive MV and would appear logical).

2. Absolutely agree, if it is tolerated by the patient.

3. How this can be implemented in real life? If unable it should not be recommended.

4. Low evidence. I patient needs the titrated flow, potential for harm if flow changed for aerosol delivery.

Recommendation Removed

Those recommendations regarding the flow titration during trans-nasal aerosol delivery will be put in the main text as statements.

**Recommendations 3.6**	~~To optimize aerosol delivery via high-flow nasal cannula, gas flow is recommended to be titrated below the patient's peak inspiratory flow if tolerated.~~
**Comments**	

Section 3: Aerosol Delivery via High-flow Nasal Cannula for Adult Patients

Recommendation 3.7: Using heliox to deliver aerosol via high-flow nasal cannula is not recommended.

Distribution of voting scores

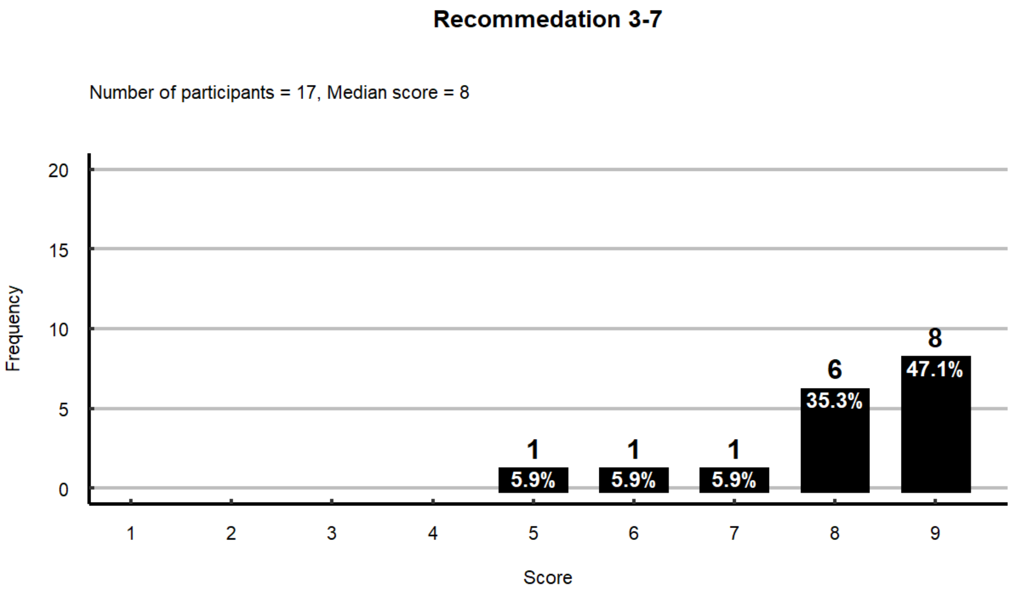

In vitro[1], very good consensus, strong recommendation

Comments of voting panelists

1. No supporting data to conclude the contrary.

2. Weak clinical benefice and cost-effectiveness, therefore I totally agree with the recommendation.

3. Agree but low level evidence.

4. Heliox via HFNC to treat airway obstruction and reduce work of breathing might be appropriate and then aerosol with heliox makes sense. Heliox for aerosol alone does not make sense. I would modify 3.7

Round 3 (please score and comment)

**Recommendations 3.7**	Using heliox **via high-flow nasal cannula for the sole purpose of improving aerosol delivery** is not recommended.

**Likert score of 1-9**	□1 □2 □3 □4 □5 □6 □7 □8 □9<br>1= absolutely disagree, 9 = absolutely agree
**Comments**	

Section 3: Aerosol Delivery via High-flow Nasal Cannula for Adult Patients

Recommendation 3.8: Using dry gas to deliver aerosol via high-flow nasal cannula has been shown to improve aerosol delivery efficiency, however considering the discomfort and the potential harms, routine use of dry gas to deliver aerosol via high-flow nasal cannula is not recommended.

Distribution of voting scores

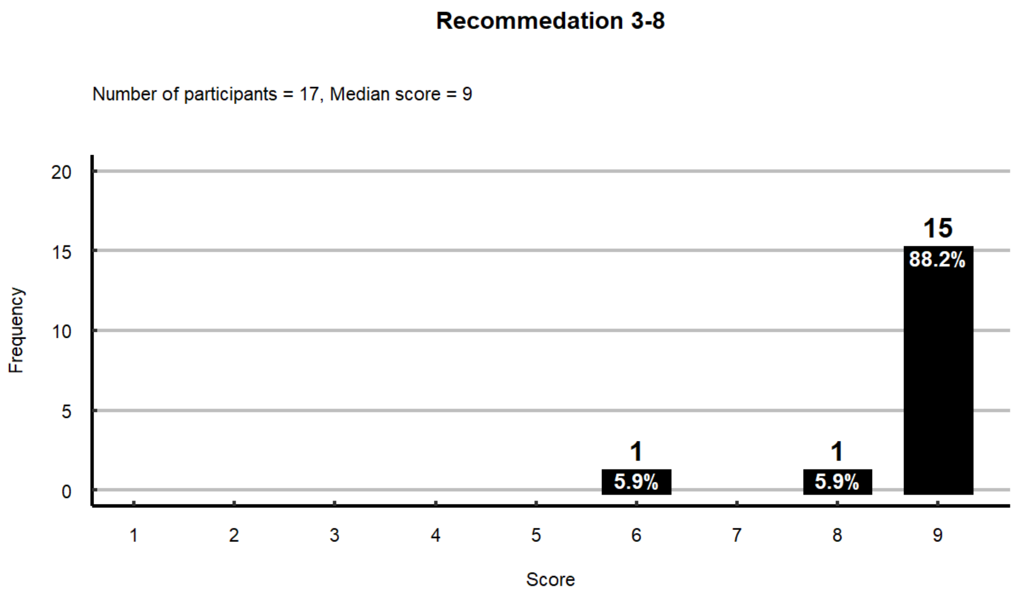

In vitro[1], very good consensus, strong recommendation

Comments of voting panelists

1. Indeed, it is only related to HFNC.

2. I totally agree with the recommendation.

3. Agree and makes sense, but low level evidence.

<u>Round 3 No need to score.</u>

Section 3: Aerosol Delivery via High-flow Nasal Cannula for Adult Patients

Recommendation 3.9： When gas flow exceeds patient inspiratory flow, open mouth breathing reduces inhaled dose. Discontinuing aerosol via high-flow nasal cannula to mouth breathing patients is not recommended.

Distribution of voting scores

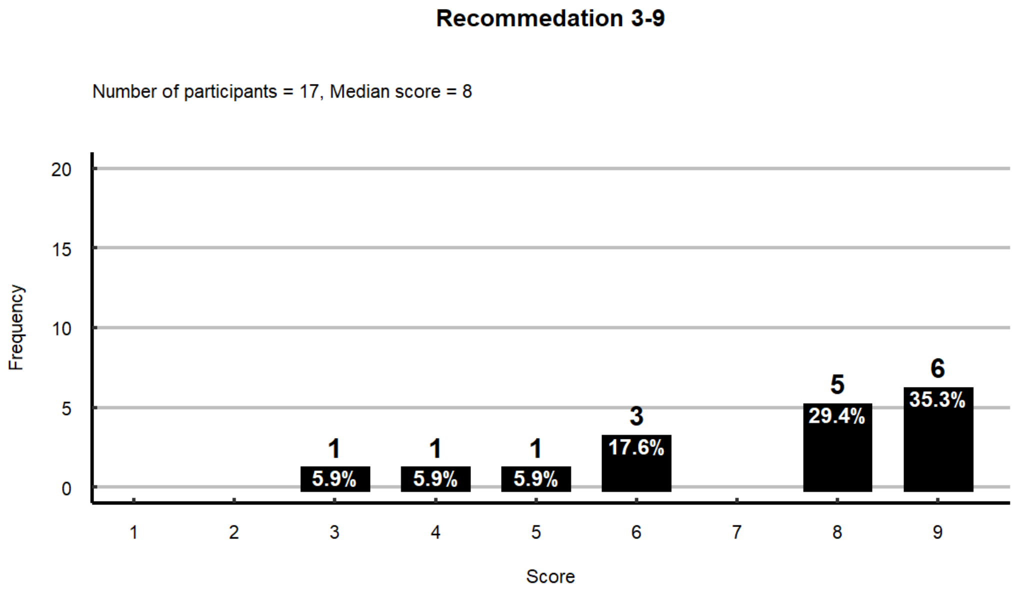

In vitro[1], good consensus, weak recommendation

Comments of voting panelists

1. I would suppress this recommendation. Too complicated, not really useful. Actually not really clear to me what we recommend. Discontiouing aerosol… is not an option if we want to deliver an aerosol. Maybe need to rephrase, but suppression would be the easiest.

2. Logical but difficult to state.

3. More evidence is needed.

Discontinuing aerosol via high-flow nasal cannula may minimize exhaled air dispersion and viral transmission.

4. Not sure I understand the recommendation.

5. Very low level of evidence.

6. Marginal reduction with mouth breathing is still better than interruption of HFNC to

administer aerosol.

Recommendation Removed

Those recommendations regarding the flow titration during trans-nasal aerosol delivery will be put in the main text as statements.

**Recommendations 3.9**	**~~When gas flow exceeds patient inspiratory flow, open mouth breathing reduces inhaled dose. Discontinuing aerosol via high-flow nasal cannula to mouth breathing patients is not recommended.~~**
**Comments**	

Section 3: Aerosol Delivery via High-flow Nasal Cannula for Adult Patients

Recommendation 3.10: For trans-nasal aerosol delivery, Optiflow is preferred over Airvo2 with vibrating mesh nebulizer placed at the inlet of humidifier. Aerosol delivery via Vapotherm should be avoided.

Distribution of voting scores

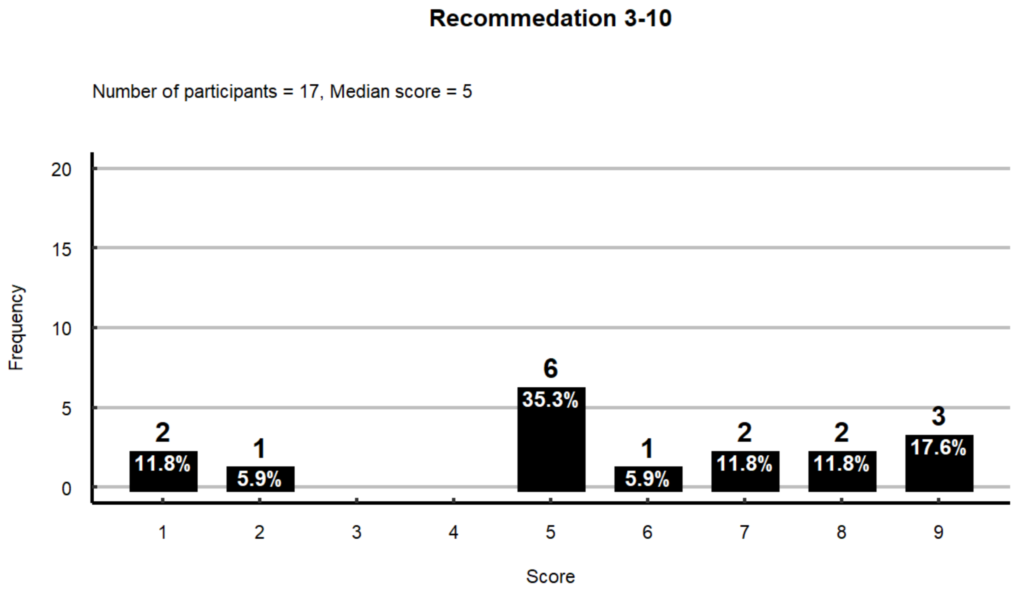

In vitro[1], no consensus, no recommendation

Comments of voting panelists

1. I agree, I am not in the favor to use commercial name in recommendations.

2. More data are needed to substantiate this statement. Preferably, some in vivo data

There may be more agreement for a more general statement such as

For transnasal aerosol delivery, Optiflow with vibrating mesh nebulizer placed at the inlet of the humidifier is recommended for efficient aerosol delivery

3. I would not go into trademarks: they will put energy in weakening the diffusion and impact of the guidelines.

The clinical evidence is with Airvo, no head to head comparisons have been made.

4. I think it depends more on the cross-sectional area of the cannulas used than the specific commercial device. Vapotherm system uses narrow nasal cannulas similar in appearance to a regular nasal oxygen cannula, whereas Optiflow system uses larger

nasal cannulas and circuit.

More evidence is needed. (Michotte)".

5. I agree with the comments above: avoid using brand names in a recommendation.

6. I would avoid recommendation to a specific / commercial mark

Maybe a recommendation like “Trans-nasal aerosol delivery depends not only to nebulizer but also humidifier type”.

7. Avoid to comment brands.

8. I would not make recommendations of trademarks. Especially with so few studies available and mainly in bench tests

Airvo2 is the machine – Optiflow are the cannulas – please check – no sense.

9. No recommendation should be made on a specific brand. Needs rewriting

10. Very low level evidence. Don’t like the use of specific brands.

Recommendation Removed

Those recommendations regarding the flow titration during trans-nasal aerosol delivery will be put in the main text as statements.

**Recommendations 3.10**	~~**For trans-nasal aerosol delivery, Optiflow is preferred over Airvo2 with vibrating mesh nebulizer placed at the inlet of humidifier. Aerosol delivery via Vapotherm should be avoided.**~~
**Likert score of 1-9**	□1 □2 □3 □4 □5 □6 □7 □8 □9<br>1= absolutely disagree, 9 = absolutely agree
**Comments**	

Appendix 6.2

Report of Round 2 scoring and comments

Section 1-2: Antibiotics Aerosol Delivery via Invasive Ventilation for Adult Patients

Recommendation 1-2.1: For antibiotics or other cost-prohibitive medications, changing to a dry circuit immediately before nebulization is recommended.

Round 3 (please score and comment)

**Recommendations 4.1**	For antibiotics or other cost-prohibitive medications, changing to a dry circuit immediately before nebulization is recommended.
**Likert score of 1-9**	□1 □2 □3 □4 □5 □6 □7 □8 □9<br>1= absolutely disagree, 9 = absolutely agree
**Comments**	

Recommendation 1-2.2: When delivering inhaled antibiotics for invasively ventilated patients, spontaneous breathing ventilator modes reduce aerosol delivery efficiency, thus spontaneous breathing should be prohibited and volume controlled mode is recommended during antibiotics nebulization.

Distribution of voting scores

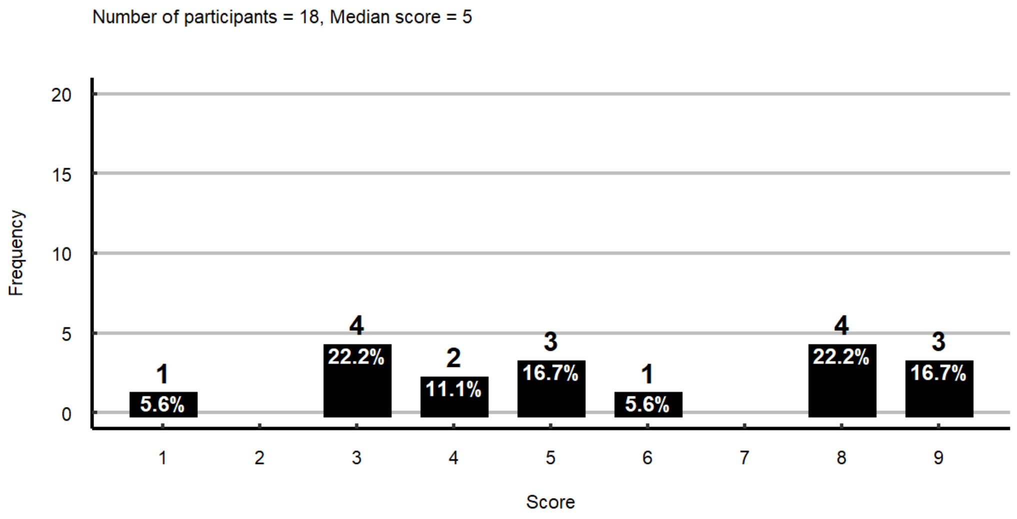

In-vitro[1,4], in vivo[2,3], no consensus, no recommendation

Comments of voting panelists

1. I Suggest the following rephrasing:

When delivering inhaled antibiotics for invasively ventilated patients, spontaneous breathing ventilator modes may reduce aerosol delivery efficiency, thus spontaneous breathing should be prohibited avoided and volume controlled mode is recommended prefered during antibiotics nebulization, assessing overall benefit/risk ratio especially related to sedation.

2. I absolutely disagree to prohibit spontaneous breathing and to recommend volume-controlled mode during antibiotics nebulization.

I think this recommendation should not be proposed in these terms (the term ""prohibited"" is too strong).

3. With optimized sedation with propofol.

4. Only one study (Dugernier J, 2016) showed better deposition with VC than PS, but in that study, mesh neb is placed between ETT and Y-piece, which is not the common

practice in clinical. The comparison of aerosol deposition with VC vs PS with mesh neb placed at inlet of humidifier is still unknown.

5. Why to paralyze a patient only to give inhaled antibiotics ?

6. The optimal ventilation mode and parameters about inhaled antibiotics during mechanical ventilation were from clinical trials proposal and expert consensus not from comparative studies. There were lack of direct data about inhaled antibiotics.

7. Very low evidence.

8. Weaning off mechanical ventilation should not be delayed for nebulization, even for antibiotics nebulization. Given the absence of true study showing clinical efficacy of nebulized antibiotics, weaning off MV should be prioritized over nebulization.

Round 3 (please score and comment)

**Recommendations 1-2.2**	When delivering inhaled antibiotics for invasively ventilated patients, spontaneous breathing ventilator modes **may** reduce aerosol delivery efficiency, thus spontaneous breathing should be ~~**prohibited**~~ **avoided** and volume-controlled mode is– ~~**recommended**~~ **preferred, and assessing overall benefit/risk ratio especially related to sedation.**
**Likert score of 1-9**	□1 □2 □3 □4 □5 □6 □7 □8 □9<br>1= absolutely disagree, 9 = absolutely agree
**Comments**	

Recommendation 1-2.3： When delivering inhaled antibiotics for invasively ventilated patients, it is recommended to set tidal volume of 8ml/kg of patient predicted body weight.

Distribution of voting scores

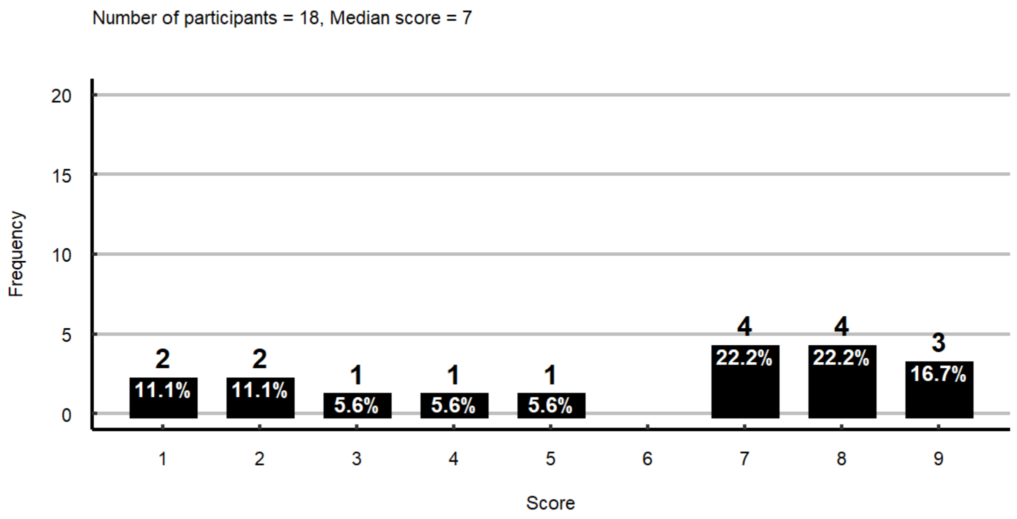

In-vivo[3], in-vitro[1,2,4], no consensus, no recommendation

Comments of voting panelists

1. If the patients has severe ARDS this is not acceptable. I think we need to recommend that the clinician weights the benefit/risk ratio of increasing tidal volume for improve aerosol delivery with the risk of high tidal volume.

2. I think we need to recommend that the clinician weights the benefit/risk ratio of increasing tidal volume for improve aerosol delivery with the risk of high tidal volume.

3. Increasing tidal volume may have adverse effects (e.g ventilator-induced lung injury …), therefore I also suggest to add a note of caution: In order to avoid VALI, plateau pressure (alveolar pressure) should not exceed 30 cmH2O.

4. In some patients with severe ARDS, setting the tidal volume from 6 to 8 ml / kg can increase significantly the plateau pressure and driving pressure, increasing the risk of ventilator-induced lung injury.

5. No evidence (except those expert opinions) shows aerosol delivery with 8ml/kg is better than with 6ml/kg. plus, it is hard to control tidal volume unless sedate or even

paralyze the patients. Notably, expert opinions are the lowest level of evidence.

6. Again, these data are coming from few studies in vitro – further changing ventilatory settings might be difficult and problematic especially for some more severe patients.

7. To most patients the parameters was difficult to achieve, and the benefits was not clear.

8. If all the sources regarding the optimization of inhaled antibiotics are the same, then you just need one comprehensive statement.

9. Cannot agree with such low level evidence. Increasing tidal volume increases risk of lung injury!

10. Potentially harmful for ARDS patients.

11. Vent parameters need to meet patient needs. No evidence that higher than 6 ml/kg Vt improves aerosol delivery.

Round 3 (please score and comment)

**Recommendations 1-2.3**	When delivering inhaled antibiotics for invasively ventilated patients, it is recommended to set tidal volume of 8ml/kg of patient's predicted body weight, **and the clinician must weigh the benefit/risk ratio of increasing tidal volume for improving aerosol delivery with the risk of high tidal volume.**
**Likert score of 1-9**	□1 □2 □3 □4 □5 □6 □7 □8 □9<br>1= absolutely disagree, 9 = absolutely agree
**Comments**	

Recommendation 1-2.4: When delivering inhaled antibiotics for invasively ventilated patients, it is recommended to keep respiratory rates at 12-15 breaths/min.

Distribution of voting scores

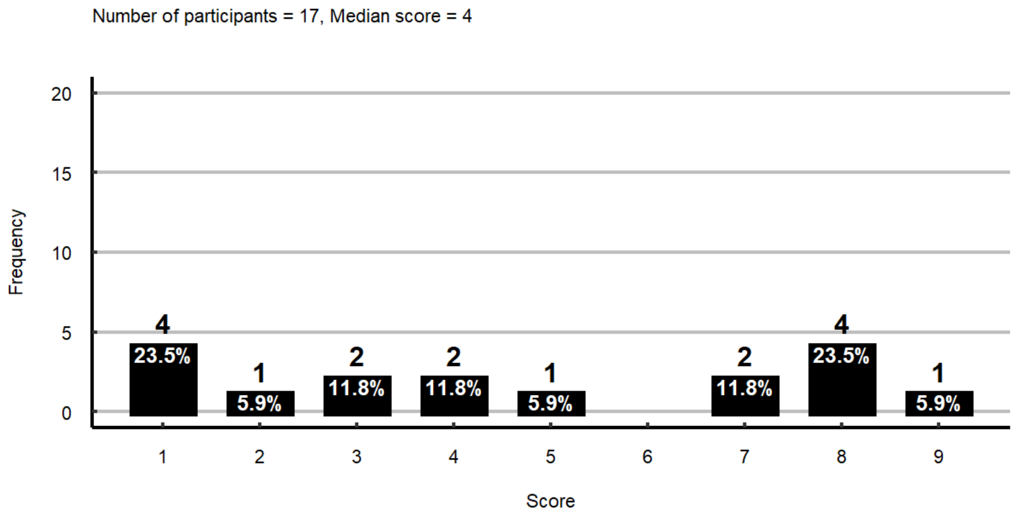

In-vivo[3], in-vitro[1,2,4], no consensus, no recommendation

Comments of voting panelists

1. <12/min) respiratory frequency.

2. Same comment. There is a need to try to reduce the RR, or to consider to reduce if otherwise clinically feasible.

Of note, the benefit is probably mostly due to inspiratory flow reduction on ventilators using I/E ratio to set the inspiratory flow. If flow remains constant… unsure why aerosol deposition should increase with decreasing respiratory rate.

3. More evidence is needed.

I would suggest to reformulate this recommendation. Indeed, it is not logical to recommend respiratory rate without specifying I:E ratio (e.g. aerosol delivery with 15 breaths/min with a I:E ratio of 1:1 is different than with 15 breaths/min with a I:E ratio of 1:3)

4. Lower (10 bpm) is better.

5. There is no clinical datas to support this recommendation. But I agree from an aerosol kinetic point of view and from aerosol datas depositon in ambulatory patient

Maybe specify “adult patient”

6. No evidence (except those expert opinions) shows aerosol delivery with 12-15 breaths/min is better than other respiratory rates. More importantly, it is hard to control respiratory rate unless sedate or even paralyze the patients

7. To most patients the parameters was difficult to achieve, and the benefits was not clear. It may be potentially harmful to achieve target parameter through sedate or even paralyze the patients.

8. If all the sources regarding the optimization of inhaled antibiotics are the same, then you just need one comprehensive statement.

9. Low level evidence. Might result in hypercarbia!

10. Difficult to implement, and need increase in sedative drugs, which may delay MV weaning. Lu et al. (AJRCCM 2011, PMID 21474643) found a non-significant increased duration of MV and ICU stay in patients who received nebulization, as compared to IV treatment.

11. Vent needs of patient trump aerosol delivery. One can always increase dose to improve target deposition.

Round 3 (please score and comment)

**Recommendations 1-2.4**	When delivering inhaled antibiotics for invasively ventilated patients, it is recommended to keep respiratory rates at 12-15 breaths/min.
**Likert score of 1-9**	□1 □2 □3 □4 □5 □6 □7 □8 □9<br>1= absolutely disagree, 9 = absolutely agree
**Comments**	

Recommendation 1-2.5: When delivering inhaled antibiotics for invasively ventilated patients, it is recommended to keep inspiratory flow below 40L/min.

Distribution of voting scores

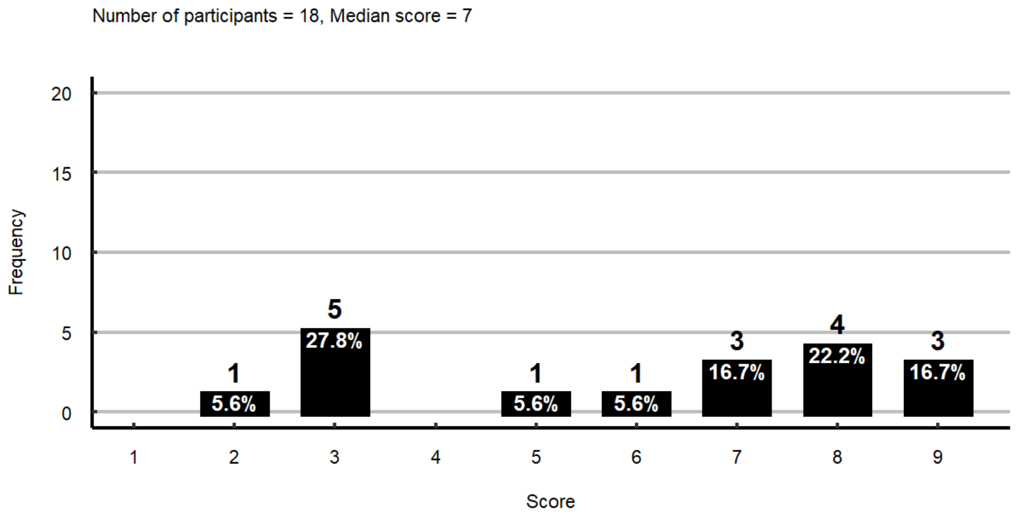

In-vitro[1-6], no consensus, no recommendation

Comments of voting panelists

1. In vitro studies showed that using low flow improves aerosol delivery. Further clinical studies are needed

I suggest to add a note of caution: check the minute volume ventilation or the undelivered tidal volume when setting low inspiratory flow.

2. Clinically, it is hard to control inspiratory flow unless sedate or even paralyze the patients.

3. I understand that some data are in favor but not so clear evidence – further changing the setting of the ventilator is always difficult in mechanically ventilated patients especially those more severe – and may discourage from using these systems for drug delivery – to be carefully discussed.

4. It may be potentially harmful to achieve target parameter through sedate or even paralyze the patients.

5. If all the sources regarding the optimization of inhaled antibiotics are the same, then you just need one comprehensive statement.

6. Low level evidence. Potential harm outweighs benefit.

7. Only if tolerated by patient.

Round 3

**Recommendations 1-2.5**	When delivering inhaled antibiotics for invasively ventilated patients, it is recommended to keep inspiratory flow below 40L/min.
**Likert score of 1-9**	□1 □2 □3 □4 □5 □6 □7 □8 □9<br>1= absolutely disagree, 9 = absolutely agree
**Comments**	

Recommendation 1-2.6: When delivering inhaled antibiotics for invasively ventilated patients, it is recommended to use inversed inspiratory to expiratory ratio of 50%.

Distribution of voting scores

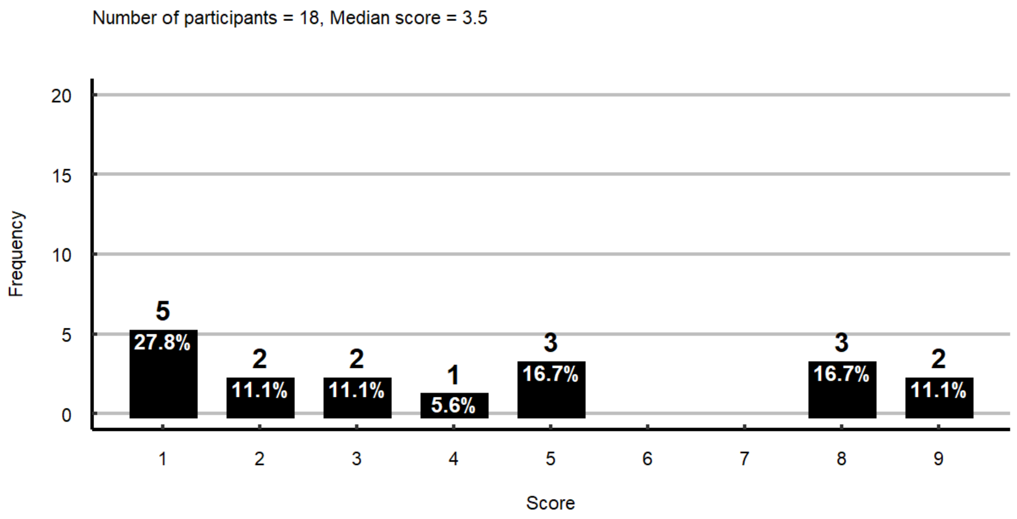

In-vitro[1-6], no consensus, no recommendation

Comments of voting panelists

1. Inspiratory to expiratory ratio is only a (complex) indirect way to set the inspiratory flow. This recommendation is redundant with the one just before. We need to suppress it.

2. Using inversed inspiratory to expiratory ratio of 50% may have adverse effect, especially in patient with obstructive diseases.

I also suggest to add a note of caution: check the persistence of end-expiratory flow at the end of the respiratory cycle on the flow-time curve which indicates the existence of auto-PEEP.

3. Need to aassciate sedation with propofol for patient's tolerance.

4. When the nebulizer operates continuously and is near to the ventilator, the tube acts like a spacer storing the aerosol during expiratory phase. In this context, there is no interest to inverse inspiratory to expiratory ratio. This recommendation depends on the nebulizer set up.

5. In the two in vitro studies by Vecellio, 2005 and Fink 1999, aerosol deposition increased by 3-6% when Ti/Ttot increased from 0.25 to 0.5, however, using of inversed

inspiratory to expiratory ratio of 50% might cause harms, such as air-trapping, barotrauma, particularly for patients who have obstructive airways. Is it really worthwhile to do so?

6. It may be potentially harmful to achieve target parameter through sedate or even paralyze the patients.

7. If all the sources regarding the optimization of inhaled antibiotics are the same, then you just need one comprehensive statement.

8. “inversed” should be removed.

9. First, I don’t understand the recommendation. Second, low level evidence. Third harm could outweigh any benefit.

10. Same comment as 1.27.3: need an increase in sedative drugs, which may or increase MV duration and delay MV weaning.

11. Unless Inverse ratio ventilation is needed for ventilator support. But carries grave cardiovascular risks for patients, and is often only tolerated with stiff lungs and severe ARDS.

Round 3 (please score and comment)

**Recommendations 1-2.6**	When delivering inhaled antibiotics for invasively ventilated patients, it is recommended to use ~~**inversed**~~ inspiratory to expiratory ratio of 50%.
**Likert score of 1-9**	□1 □2 □3 □4 □5 □6 □7 □8 □9<br>1= absolutely disagree, 9 = absolutely agree
**Comments**	

Recommendation 1-2.7： When delivering inhaled antibiotics for invasively ventilated patients, it is recommended to use a constant inspiratory flow.

Distribution of voting scores

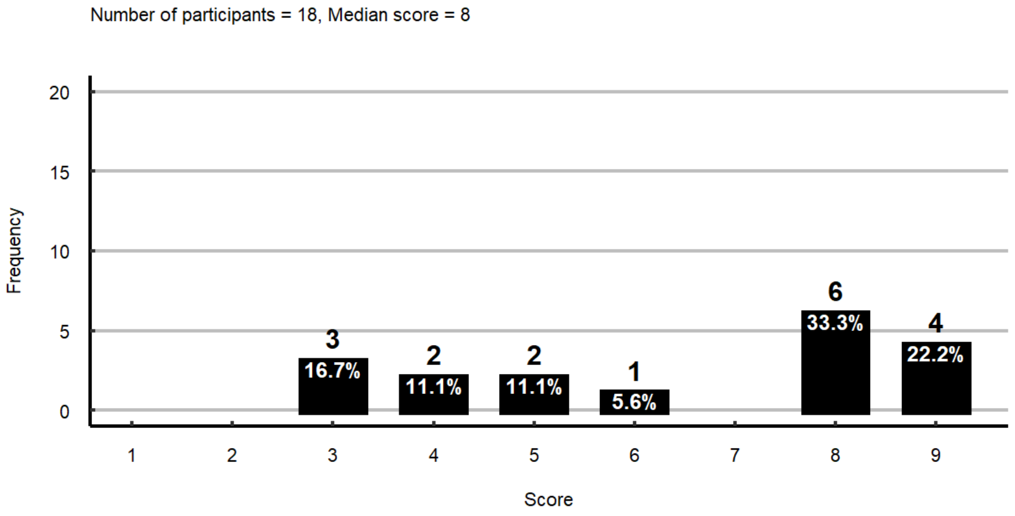

In-vitro[1,3,4] and in-vivo[2], some consensus, weak recommendation

Comments of voting panelists

1. Limited in vitro data suggested that inspiratory flow patterns may influence aerosol delivery. Further clinical studies are needed.

2. There not enough clinical data to support this, particularly in tems of aerosol distribution/clinical efficacy. For example, an increasing flow during inspiratory time could be theoretical better to target distal region than a constant flow (considering a similar tidal volume and inspiratory time).

3. It is hard to control inspiratory flow pattern unless sedate or even paralyze the patients.

4. This recommendation should be carefully discussed with that above – first if possible don’t change inspiratory flow – but in case constant is better – to be clearly explained in the discussion section.

5. If all the sources regarding the optimization of inhaled antibiotics are the same, then you just need one comprehensive statement.

6. Very low level of evidence.

7. Limited evidence to support this. Sinusoidal, accelerating wave form may work as

well.

Round 3 (please score and comment)

**Recommendations 1-2.7**	When delivering inhaled antibiotics for invasively ventilated patients, it is recommended to use a constant inspiratory flow.
**Likert score of 1-9**	□1 □2 □3 □4 □5 □6 □7 □8 □9<br>1= absolutely disagree, 9 = absolutely agree
**Comments**	

Recommendation 1-2.8： When delivering inhaled antibiotics for invasively ventilated patients, it is recommended to set end-inspiratory pause at 20%.

Distribution of voting scores

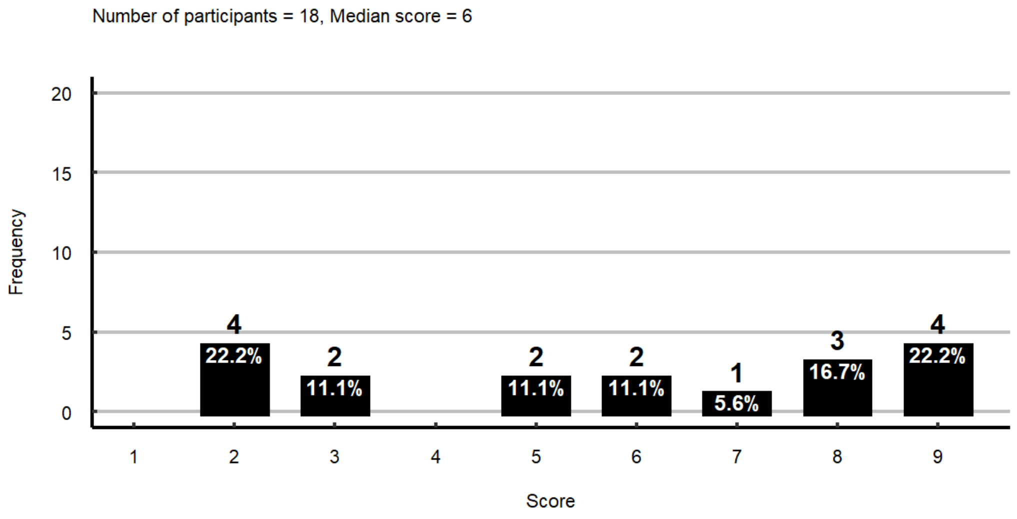

In-vivo[1], no consensus, no recommendation

Comments of voting panelists

1. Again, recommendation should be to aim for this.

2. I totally agree with the reformulation of the recommendation.

Limited data to recommend an end-inspiratory pause at 20%.

3. There is no data demonstrating the influence of pause at 20% on deposition using nebulizer.

4. Only one study.

5. The in vivo evidence shows the use of end-inspiratory of 5s did not have better results on reducing airway resistance, in comparison to no use of end-inspiratory hold.

6. Scarce evidence – difficult to do in practice – discourage use of nebulization therapy.

7. Lack of evidence.

8. If all the sources regarding the optimization of inhaled antibiotics are the same, then you just need one comprehensive statement.

9. Low level evidence. 20% results in different amounts of time depending on inspiratory time. Seems this is based on one brand of ventilator.

10. No evidence to support increase in lung retention is greater than the percent of continuous aerosol inhaled. And with 1:1 I:E ratio, even more dangerous potential for Cardiovascular insult.

Round 3 (please score and comment)

**Recommendations 1-2.8**	When delivering inhaled antibiotics for invasively ventilated patients, it is recommended to set end-inspiratory pause at 20%.
**Likert score of 1-9**	□1 □2 □3 □4 □5 □6 □7 □8 □9<br>1= absolutely disagree, 9 = absolutely agree
**Comments**	

Recommendation 1-2.9: When delivering inhaled antibiotics for invasively ventilated patients, it is recommended to set a positive end-expiratory pressure (PEEP) at 5-10 cmH_2O.

Distribution of voting scores

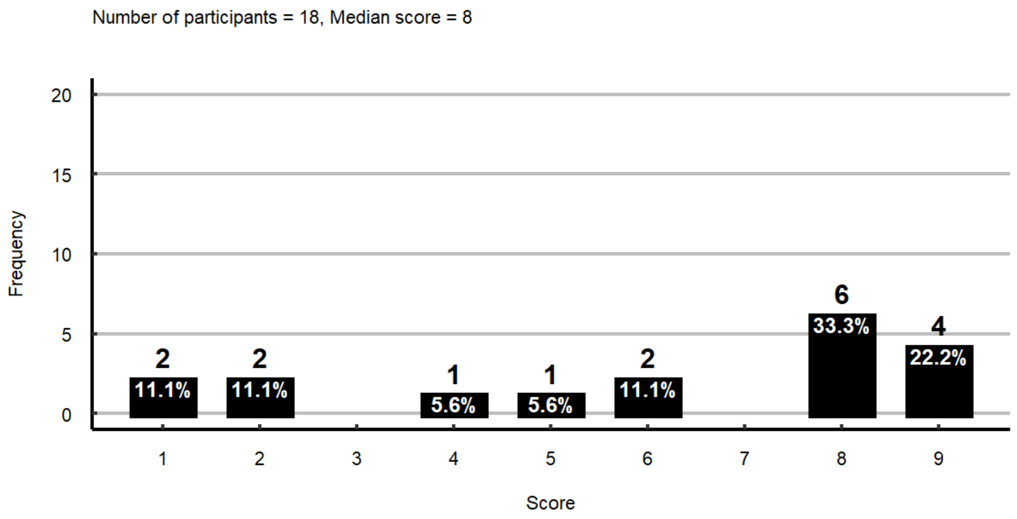

In vitro[1,3] and in vivo[2], no consensus, no recommendation

Comments of voting panelists

1. Add "At least". It would not be incorrect if PEEP is higher due to other reasons.

2. The priority of the PEEP setting should remain the ventilation (e.g. oxygenation, cardiac output…).

Further clinical studies are needed to assess the additive effect of the PEEP setting in the aerosol delivery efficiency

3. I agree to use of PEEP during antibiotic nebulization, but I disagree with defining the range of PEEP levels. As for alveolar recruitment, we can reasonably believe that physician set the PEEP level according to each patient's lung function. In clinical practice, PEEP levels are rarely set lower than 5 cmH2O.

4. No datas supporting this recommendation in mechanical ventilation but few clinical reported datas in ambulatory patients.

5. In the studies conducted by Williams 1999 and Vecellio, 2005, no significant differences of aerosol deposition with PEEP at 0-10 cmH2O. clinically, PEEP setting

should be adjusted based on patient needs.

6. See comments above.

7. If all the sources regarding the optimization of inhaled antibiotics are the same, then you just need one comprehensive statement.

8. Very low level of evidence.

9. Limited evidence that increase peep improves aerosol delivery. So would you not use higher levels of PEEP if patient needs it?

Round 3 (please score and comment)

**Recommendations 1-2.9**	When delivering inhaled antibiotics for invasively ventilated patients, it is recommended to set a positive end-expiratory pressure (PEEP) at 5-10 cmH_2O.
**Likert score of 1-9**	□1 □2 □3 □4 □5 □6 □7 □8 □9<br>1= absolutely disagree, 9 = absolutely agree
**Comments**	

Appendix 7

Report of Round 3

Section 1-1: Aerosol Delivery via Invasive Ventilation for Adult Patients **(not specific to antibiotics)**

Recommendation 1-1.1：During invasive ventilation, vibrating mesh nebulizer is more efficient in aerosol delivery than continuous jet nebulizer, with no influence on flows or fraction of inspired oxygen. Vibrating mesh nebulizer is preferred over continuous jet nebulizer.

Distribution of voting scores

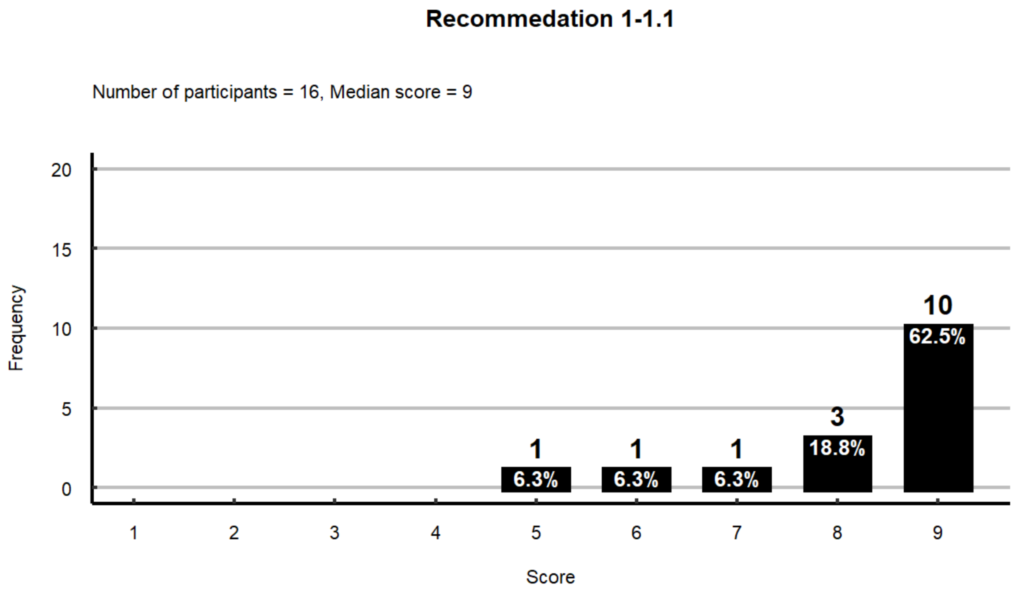

In vitro[1-6,9], in vivo[7,8], very good consensus, strong recommendation

Comments of voting panelists

5	Not sure that for bronchial tract target, these differences mean a benefit. Particle size delivery is more important
9	
9	
7	
9	It is very clear to me
9	
9	
9	Vibrating mesh nebulizers generate higher inhaled dose than jet nebulizers. Additionally, the absence of influence on ventilator settings and FiO2 represents an important benefit over jet nebulizer. Question arises for high viscous drugs
9	

8	
9	
8	
9	
8	
9	
6	Cost is not included. More is not necessary better.

Recommendation 1-1.3：Based on variation of the reported inhaled doses and lack of definitive clinical outcomes, there is no recommendation for metered dose inhaler and spacer versus vibrating mesh nebulizer.

Distribution of voting scores

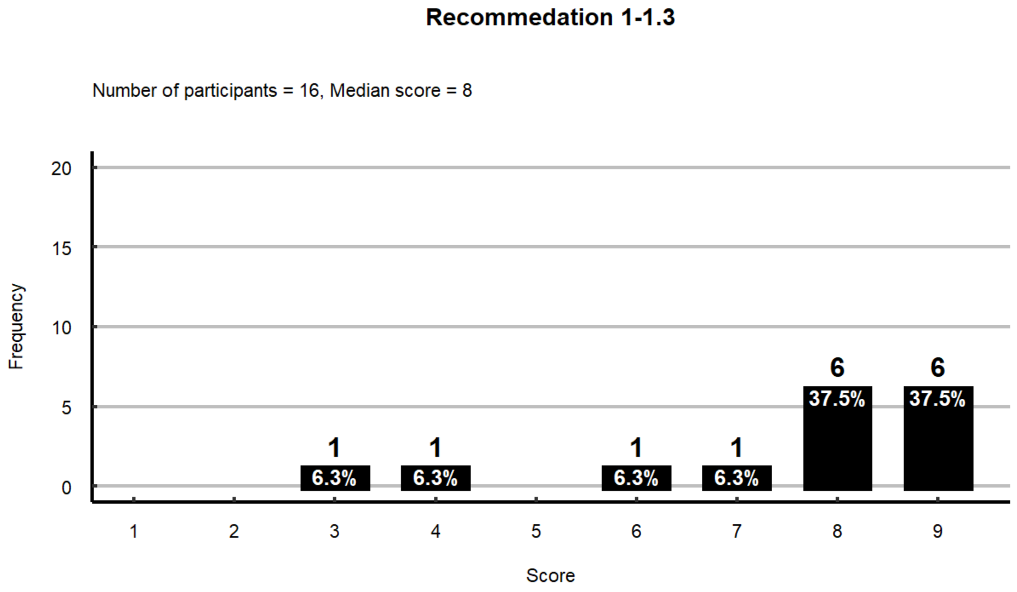

In vitro[1-3,5], in vivo[2,4,6,7], very good consensus, strong recommendation

Comments of voting panelists

7	It depends of the delivered drug.
9	
9	
8	
9	Could we comment on the minimal dosage required?
9	
8	
9	No difference can be really found between these modalities, more clinical studies are needed.
8	Only true for bronchodilators
3	Dhand et al (Rajiv Dhand, How Should Aerosols Be Delivered During Invasive Mechanical Ventilation? Respiratory Care Oct 2017, 62 (10) 1343-1367; DOI: 10.4187/respcare.05803.)

	specified in a publication than in mechanically ventilated subjects, significant bronchodilator effects occurred after administration of 4 puffs (400 µg) with a pMDI or 2.5 mg of albuterol with a standard nebulizer. This recommendation is based on the clinical study : Duarte AG, Momii K, Bidani A. Bronchodilator therapy with metered-dose inhaler and spacer versus nebulizer in mechanically ventilated patients: comparison of magnitude and duration of response. Respir Care 2000;45(7):817–823.<br>Indeed, this recommendation is with a jet nebulizer instead of a mesh nebulizer, it is important to know that bronchodilatator achieves a plateau in terms of FEV1. So there is no reason to no apply this recommendation for bronchodilatator with nebulizers in general (including mesh)
8	
6	
8	
9	
8	
4	

Recommendation 1-1.6： When placed at the inspiratory limb before Y-piece, metered dose inhaler with a spacer is more efficient in aerosol delivery than the continuous jet nebulizer, with no influence on flows or fraction of inspired oxygen. When available, metered dose inhaler with spacer actuated at beginning of inspiration is recommended over continuous jet nebulizer.

Distribution of voting scores

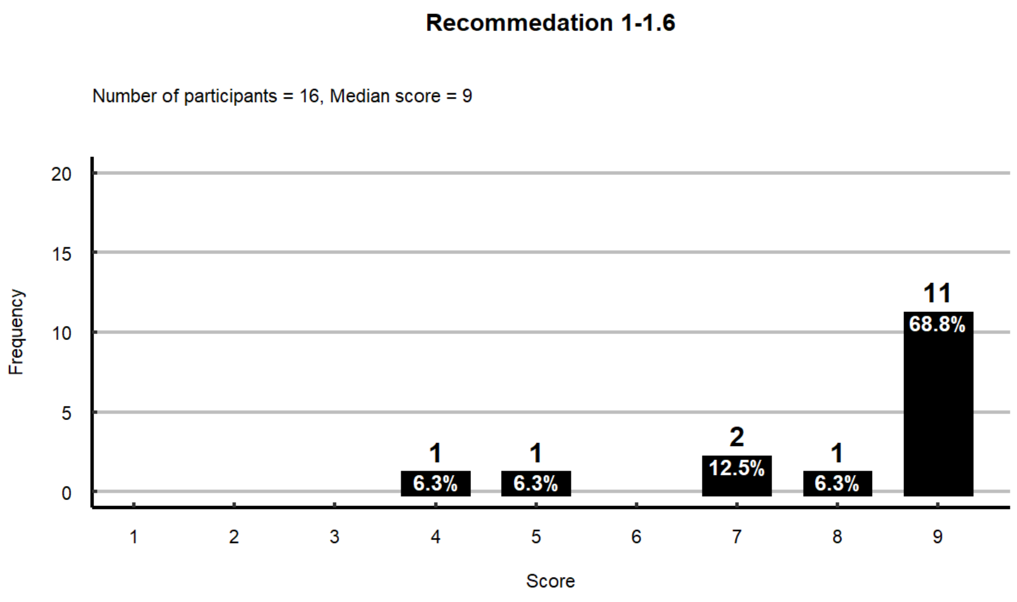

In vitro[1,2,5], in vivo[3,4,6,7], very good consensus, strong recommendation

Comments of voting panelists

7	
9	
9	
8	
9	
9	
7	Lack of clinical evidence.
9	I agree with this statement if we consider the fraction of inhaled dose and regarding the no influence on flows.<br>More clinical studies are needed.
9	

5	Disagree in terms of clinical efficacy : continuous jet = pMDI+spacer (Duarte AG, Momii K, Bidani A. Bronchodilator therapy with metered-dose inhaler and spacer versus nebulizer in mechanically ventilated patients: comparison of magnitude and duration of response. Respir Care 2000;45(7):817–823.) But agree in terms of security.
9	
9	
9	
9	“When place in” rather than “placed at”?
9	
4	Efficiency versus actual amount delivered is not considered.<br>Cost is also not considered.

Recommendation 1-1.8： When vibrating mesh nebulizer is utilized during invasive ventilation with bias flow, it is recommended to be placed close to ventilator.

Distribution of voting scores

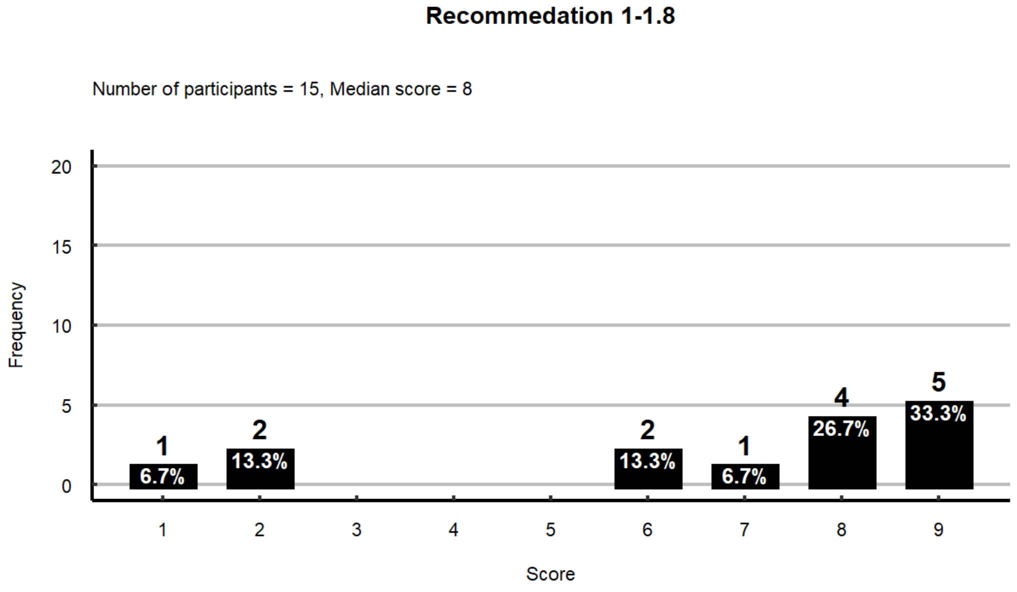

In vitro[1-5], good consensus, weak recommendation

Comments of voting panelists

	I don't understand what means bias flow.
1	The effect of bias flow on bronchodilator and/or corticosteroid aerosol delivery is limited. It does not justify placement of the vibrating mesh nebulizer "close to the ventilator"
2	I personally believe that the effect of bias flow on bronchodilator and/or corticosteroid aerosol delivery is clearly defined. For these reasons it does not justify placement of the vibrating mesh nebulizer "close to the ventilator" in any case – important issue for discussion also in the paper
9	
2	The effect on bias flow is low
9	
7	
8	The optimal position of the nebulizer depends on the bias flow level. More clinical studies are needed to support this recommendation
8	Could be different depending on the value of the bias flow

8	
9	
9	
6	
6	I would rather write at distance from the Y piece.
9	
8	

Recommendation 1-1.9：When jet nebulizer is utilized during invasive ventilation, it is recommended to be placed near the ventilator.

Distribution of voting scores

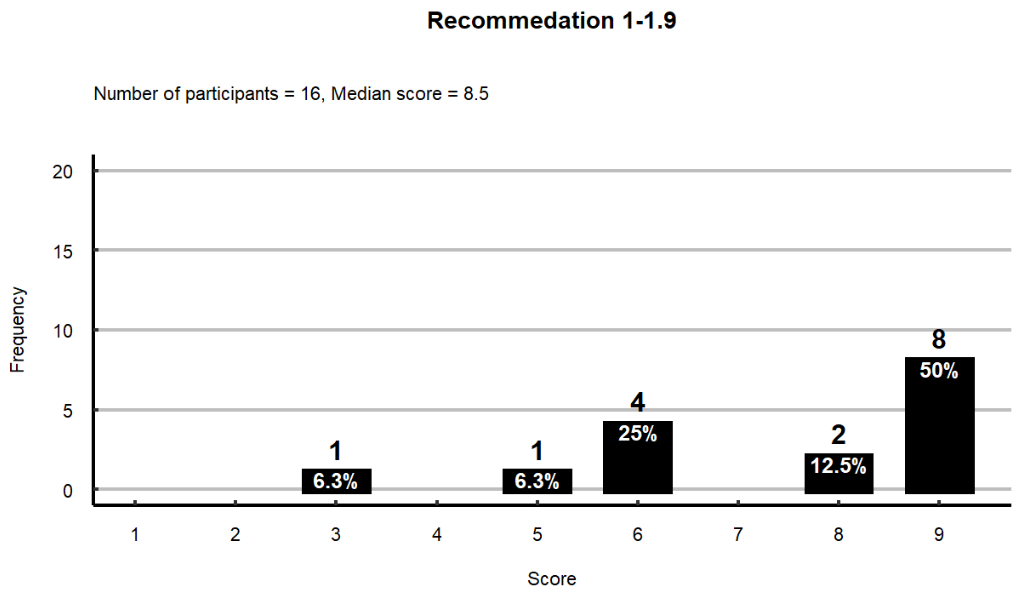

In vitro[1-3], good consensus, weak recommendation

Comments of voting panelists

6	
9	
9	
9	
9	
9	
6	This sentence may cause confusion. If the word "continuous" is removed, could it mean it is also recommended that the inspiration synchronized jet nebulizer be placed near the ventilator?
8	The optimal position of the (continuous) jet nebulizer depends on the bias flow level. More clinical studies are needed.
9	
3	I suggest "When continuous jet nebulizer is utilized during invasive ventilation, it is recommended to be placed near the ventilator"
8	If possible, jet nebulizers should be avoided during invasive ventilation.

9	
6	
6	
9	
5	

Recommendation 1-1.12：When metered dose inhaler is utilized during invasive ventilation, it is recommended to be used with a spacer with volume > 150mL.

Distribution of voting scores

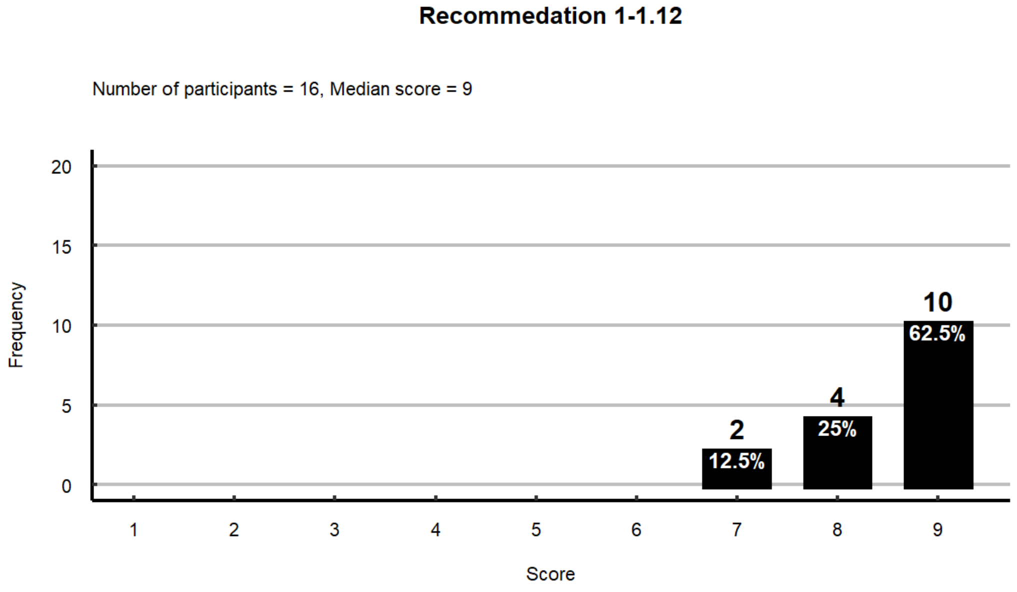

In vitro[1-7], in vivo[8,9], perfect consensus, strong recommendation

Comments of voting panelists

9	
7	
7	
8	
8	
9	
8	
9	There are limited clinical data to support this statement
9	
9	
9	
9	
9	
9	
9	

8	

Recommendation 1-1.13： During invasive ventilation, metered dose inhaler and spacer are recommended to be placed in the inspiratory limb before the Y-piece.

Distribution of voting scores

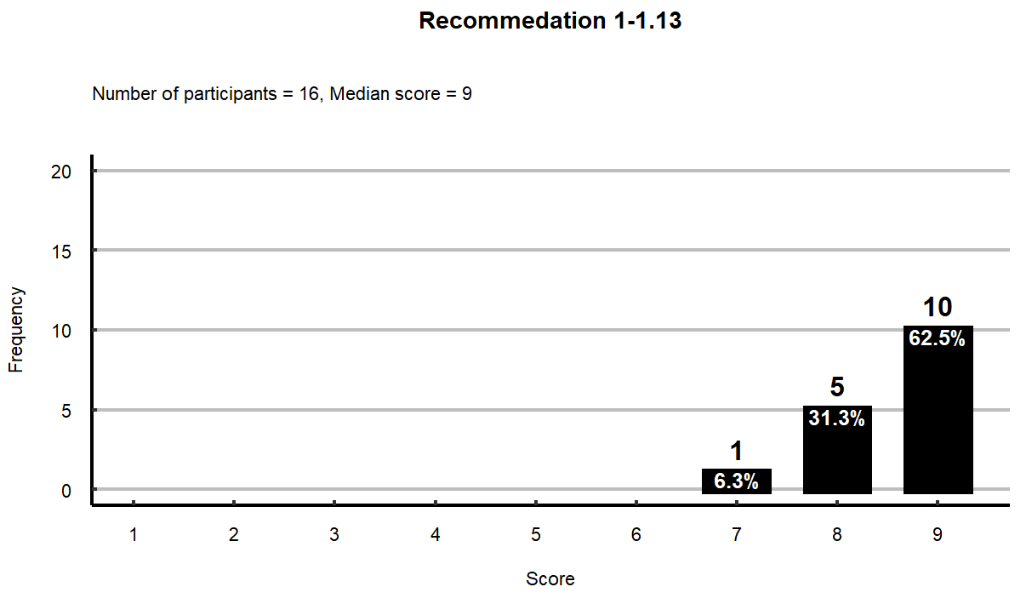

In vitro[1,2], in vivo[3], perfect consensus, strong recommendation

Comments of voting panelists

9	
9	
9	
8	
9	
9	
8	
9	I agree with this statement, but evidence is weak
9	
8	Between the Y piece an ETT is another possibility but the spacer must be removed just after aerosol delivery to avoid rebreathing (potential risk).
8	
9	Lin HL, Fink JB, Ge H. Aerosol delivery via invasive ventilation: a narrative review. Ann Transl Med. 2021;9(7):588. doi:10.21037/atm-20-5665

	How Should Aerosols Be Delivered During Invasive Mechanical Ventilation?Dhand R.Respir Care. 2017 Oct;62(10):1343-1367. doi: 10.4187/respcare.05803
9	
8	Immediately before or immediately after Y piece is equivalent. I would write “close to the Y piece”. If placed between the Y piece and patients it can only be temporary (remove or use collapsible chamber).
9	
7	

Recommendation 1-1.15：The efficiency of aerosol delivery in dry ventilator circuits is higher than that in humidified ventilator circuits. Considering the potential harms of dry gas on patient airway, and the time lapse required for a humidifier and circuits to cool down, turning off humidifier is not recommended for routine aerosol therapy.

Distribution of voting scores

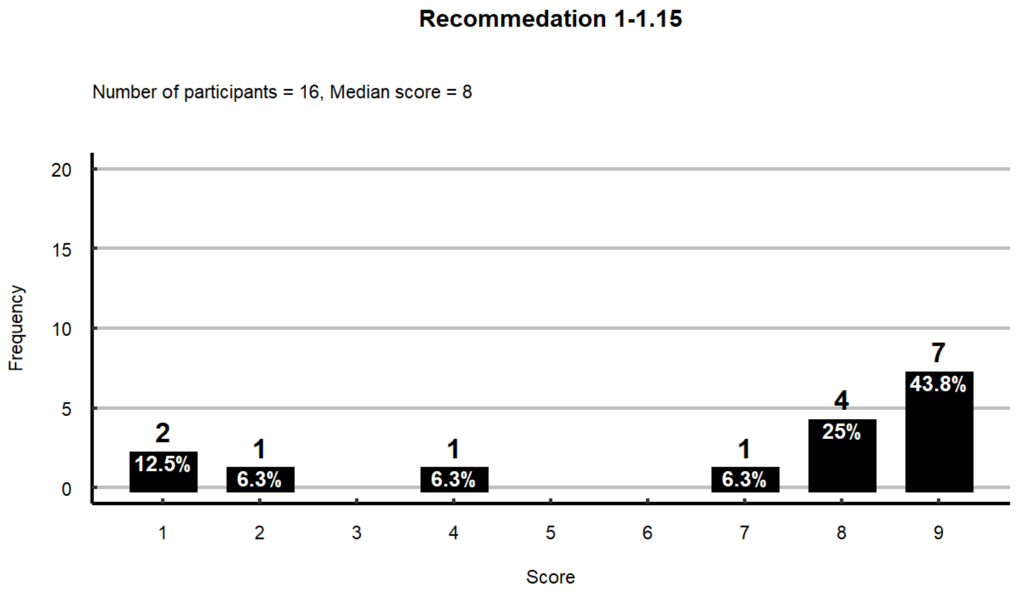

In vitro[1-11], in vivo[6,13,14], some consensus, weak recommendation

Comments of voting panelists

1	No evidence to make a recommendation. I suggest that degree of evidence need to be added with each recommendation.
1	There is compelling evidence that the administration of an aerosol on top of a dry inspiratory gas at room temperature, is associated with enough humidification and does not induce harm if the nebulization is less than one hour. I suggest to modify the recommendation for<br>"The efficiency of aerosol delivery in dry ventilator circuits is higher than that in humidified ventilator circuits. Considering that the aerosol provides humidification of dry inspiratory gas and that ambient temperature alone does not produce tracheobronchial injury for exposures less than several hours, turning off humidifier is a possible option for routine aerosol therapy"
4	I carefully checked the literature, and my conclusion is that the administration of aerosol in dry inspiratory gas at room temperature might be associated with

	harm only if the nebulization is for more than one hour. For these reasons I suggest modifying the recommendation above as follows:<br>“The efficiency of aerosol delivery in dry ventilator circuits is higher than that in humidified ventilator circuits. Considering dry gases might induce tracheobronchial injury only after at least 1 hour, turning off humidifier might be considered but not fully recommended for routine aerosol therapy” – what do you think?
9	
2	There is evidence that the administration of an aerosol on top of a dry humification produces enough humidification if the nebulization period is short
9	
8	I agree with this recommendation from a safety perspective. It should be noted that when a heat-moisture exchanger is used for airway humidification, removal of the HME during aerosol administration also means no humidification (Recommendation 1-1.16). A 1-hour nebulization without humidification may not be dangerous, but forgetting to turn on the humidifier or reconnect the HME after aerosolization can be dangerous. This issue should be discussed in the main text. In addition, I suggest adding a sentence in the recommendation 1-1.16 to remind the reconnection of the HME after aerosolization.<br>Regarding adverse effects caused by unhumidified gas during nebulization, in the references provided by Dr. Rouby, bronchial hyperactivity caused by cold and dry gas in asthmatic patients is not mentioned. Since this section deals with bronchodilators, I agree that humidifier discontinuation is not recommended for routine aerosol therapy.
9	I totally agree if we consider that the main point is the potential harms of dry gas on patient airways.
9	
9	
9	
8	
8	
9	

7	
8	The increased in drug delivery on a dry circuit is offset by the risk of leaving the humidification off.

Recommendation 1-1.17： In ventilated patients, using a continuous jet nebulizer means adding compressed gas independent of the ventilator. The effect on tidal volume, FiO_2 etc makes this practice unacceptable. The empirical compensations on ventilator settings may be dangerous and should be avoided. If no integrated inspiration-synchronized jet nebulizer is available, the use of continuous jet neb in ventilated patients is not recommended.

Distribution of voting scores

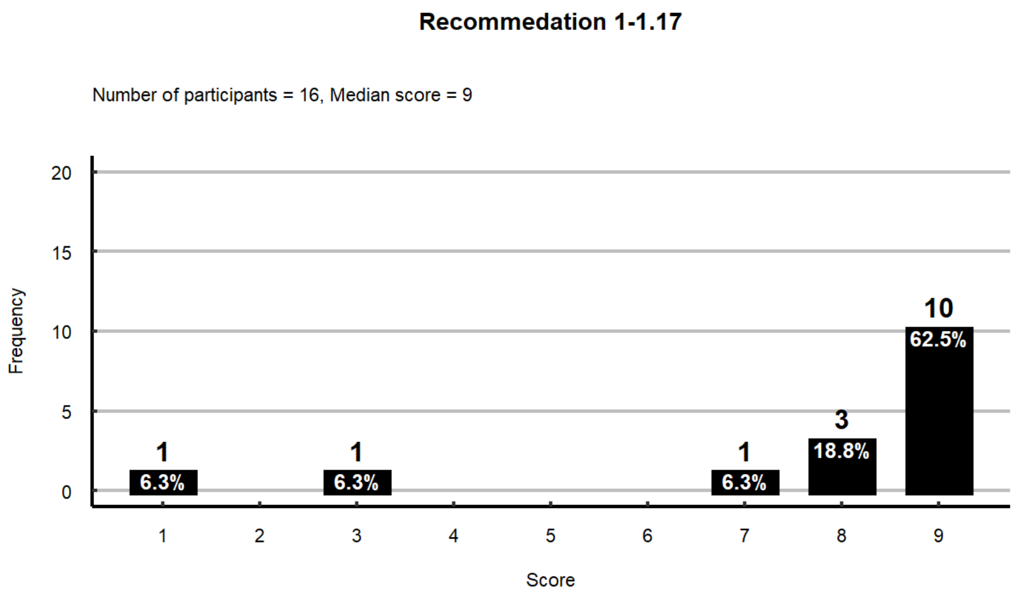

In vitro[1,2], in vivo[1], very good consensus, strong recommendation

Comments of voting panelists

9	
9	
9	
3	The Hamilton ventilator has a "continuous nebulization" function, using a fraction of flow from the ventilator. To avoid the confusion, the first sentence could change to "In ventilated patients, using an external gas-powered jet nebulizer means adding compressed gas independent of the ventilator." Mesh nebulizer is not affordable in many countries; I suggest to delete the last sentence. The sentence of "The effect on tidal volume, FiO2 etc makes this practice unacceptable" is too strongly negative when clinicians have no choice rather using it. This can be revised like "the clinician must weigh the

	benefit/risk ratio of alternating of tidal volume and FiO2 in the ventilator system form the external gas.
9	
7	
8	I agree with this recommendation. But if the continuous jet nebulizer cannot be recommended for this reason, do the other recommendations regarding the continuous jet nebulizer during invasive mechanical ventilation still make sense?
9	I totally agree with this statement, but evidence is weak
9	
9	I agree. But some words should be removed in the recommendation. Because in pressure control mode, the ventilator can adjust a part of parameters to compensate the additional gas from the nebulizer.<br>I suggest this recommendation "In ventilated patients, using a continuous jet nebulizer means adding compressed gas independent of the ventilator. The effect on tidal volume, FiO2 etc makes this practice unacceptable. The empirical compensations on ventilator settings may be dangerous and should be avoided. If no integrated inspiration-synchronized jet nebulizer is available, the use of continuous jet neb in ventilated patients is not recommended."
8	
9	
8	
9	
9	
1	This statement is your expert opinion not based on animal or human data. The effects of using different technologies could be discussed in the text.

Recommendation 1-1.20.1： For the jet or ultrasonic nebulizer with a residual volume > 0.5 ml, aerosol delivery efficiency is improved with a higher fill volume, but changing fill volume for the sole purpose of improving aerosol delivery efficiency is not recommended for FDA approved inhaled medication.

Distribution of voting scores

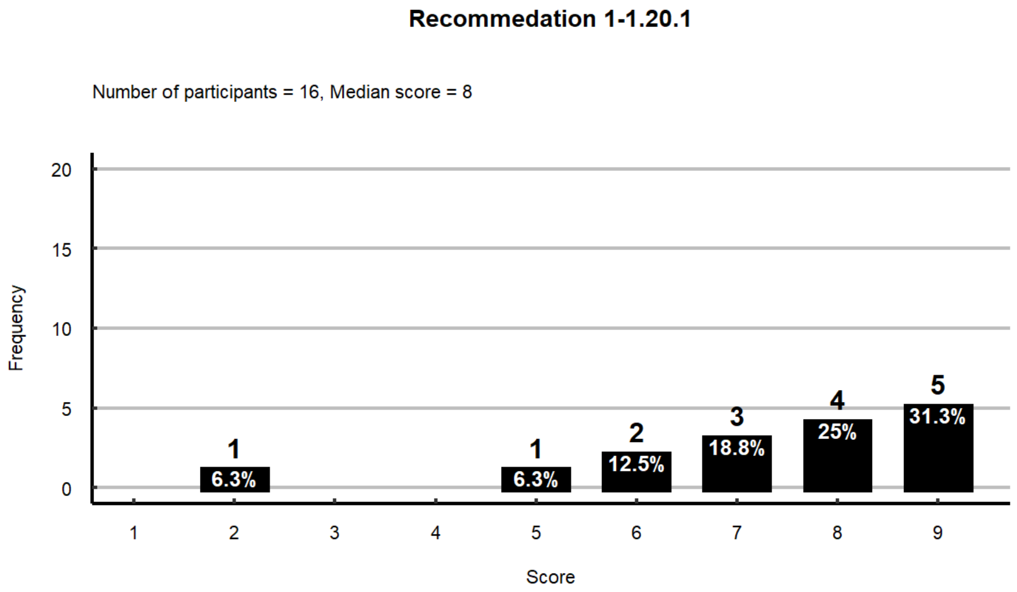

In vitro[1,2], good consensus, weak recommendation

Comments of voting panelists

9	
5	
6	
8	
7	
9	
9	
2	Data suggest that increasing diluent volume might be associated with improving of aerosol delivery efficiency.<br>However, I really don’t know the conditions for
7	I’m not convince because if a higher fill volume improves the efficiency, why we recommend to doing differently?
8	Why 0.5mL and not 1mL ?

8	
6	
9	
8	
9	
7	If this plans to be an international recommendation FDA<br>should be replaced by regulatory agencies.<br>What is an FDA approved medication? Albuterol, atrovent are FDA approved.<br>Do you mean approved as drug device formulation?

Recommendation 1-1.20.2：Increasing diluent volume in vibrating mesh nebulizer to improve aerosol delivery efficiency is not recommended.

Distribution of voting scores

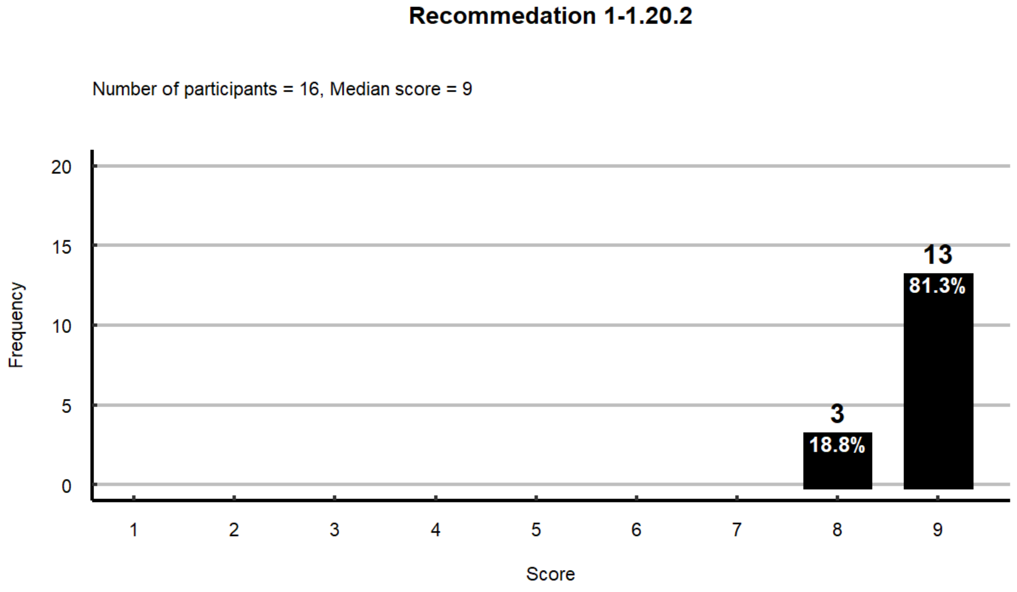

In vitro[1,2], perfect consensus, strong recommendation

Comments of voting panelists

9	
9	
9	
9	
8	
9	
8	
9	Considering the low residual volume of vibrating mesh devices, I agree with this statement.
9	
9	
9	
9	
9	
9	

9	
8	Add “ except for viscous formulations” and remove next recommendation.

Recommendation 1-1.20.3：For viscous formulations, increasing diluent volume in vibrating mesh nebulizer to improve aerosol delivery efficiency is recommended.

Distribution of voting scores

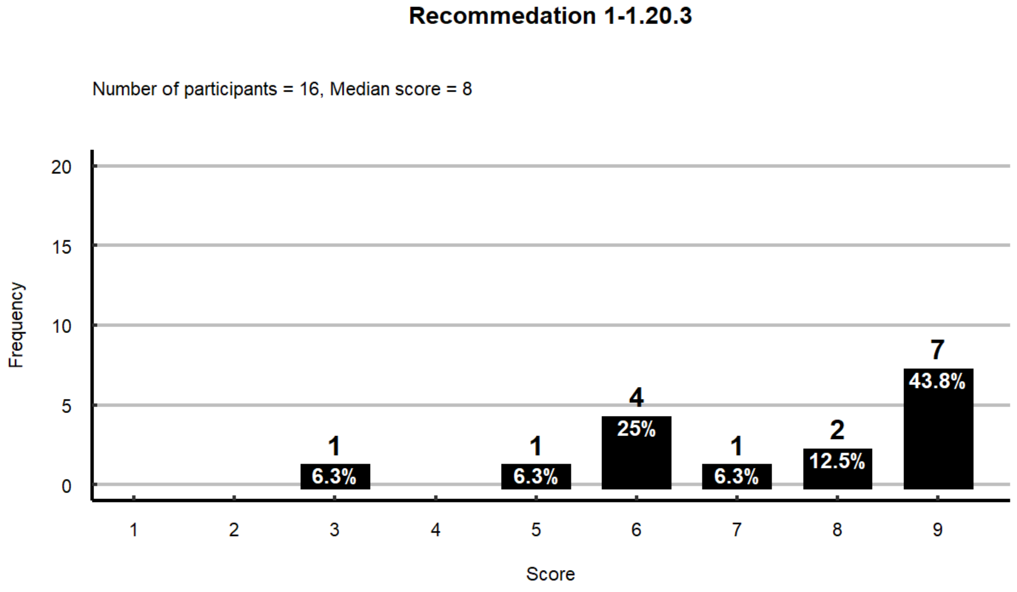

In vitro[1,2], good consensus, weak recommendation

Comments of voting panelists

6	
9	
9	
8	
9	
9	
7	This information is useful for clinical practice. It should be noted that changes in concentration or distribution could modify the final characteristics of delivered aerosols in terms of aerodynamic diameter and aerosol output.
9	The efficiency of vibrating mesh nebulizers may be decreased with viscous drugs, therefore adding diluent might improve aerosol delivery efficiency.
6	No sufficient evidence I think
5	Theoritically yes, but how do you define the viscous formulation?. Maybe it could be interestering to provide quantitative information in terms of Centipoise.

	Furthermore, it raises the problem of the diluent formualtion (water or physiological serum or other ?) and its influence on toxicity/stability (ie in terms of pharmacology and regulatory).<br>For exemple, using Tobi for nebulization, it is not recommended to dilute it (IFU).
6	
6	Viscous formulations is not recommended in vibrating mesh nebulizers
9	
3	I don’t think it makes sense to make a recommendation on this topic : this would require to define what is a viscous formulation (how viscous to set a cut off), this means in any case we discuss about off label use.
9	
8	Should be combined with 1-1.20.2

Recommendation 1-1.24：When heliox is substituted for oxygen to drive continuous jet nebulizer at the same driving flow, nebulizer output is reduced. If driving nebulizer with heliox, it is recommended to set at 15 L/min.

Distribution of voting scores

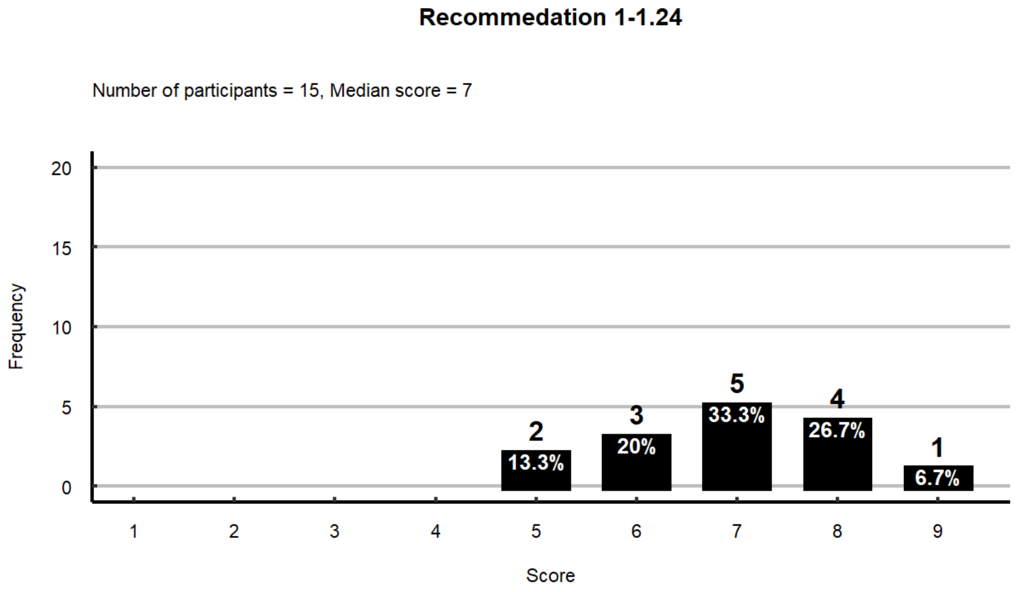

In vitro[1], good consensus, weak recommendation

Comments of voting panelists

	No heliox experience
6	Very few teams use Heliox for driving continuous jet nebulization (very high cost/benefit ratio) and I suggest to remove this recommendation.
6	This is not a relevant recommendation from practical point of view – should we remove it from final analysis ?
8	
7	Heliox is uncommonly used
8	
5	
7	I agree with increasing the flow when using Heliox, but evidence is weak
7	Evidence is too low
5	It could depend on the type of jet nebulizer. It is difficult to give a recommendation without validation on different commercial jet nebulizers. Furthermore no clinical study.

8	Physically makes sense.
8	
7	
6	
9	
7	

Recommendation 1-1.25：It is not recommended to change the ventilator mode for the sole purpose of improving aerosol delivery efficiency.

Distribution of voting scores

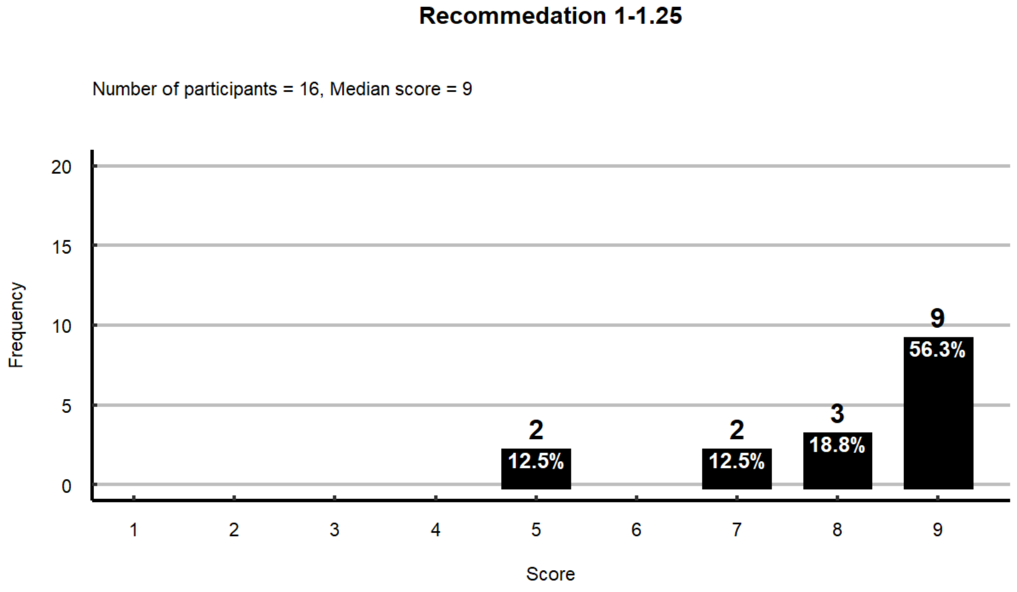

In-vitro[1,4], in vivo[2,3], very good consensus, strong recommendation

Comments of voting panelists

5	
5	The delivery to the tracheobronchial tree of nebulized bronchodilators and/or corticosteroids is higher with constant inspiratory volume controlled ventilation. However, alternative modes of mechanical ventilation still provide enough delivery to the tracheobronchial tree and can be used.
7	I agree that the delivery of nebulized bronchodilators or other drugs is greater during controlled ventilation at constant inspiratory flow. But I also think that some other alternative type mechanical ventilation are available providing enough delivery and for these reasons can be used in clinical practice – I would smooth the reccomendation.
9	
7	There are some modes of MV that improve the efficiency of delivery
9	
8	
9	The risk outweighs benefit, I totally agree with this statement

8	I agree even if the evidences are low
9	
9	
8	
9	
9	
9	
9	

Section 1-1: Aerosol Delivery via Invasive Ventilation for Adult Patients

Recommendation 1-1.27： It is not recommended to change tidal volume and respiratory rate for the sole purpose of improving aerosol delivery efficiency.

Distribution of voting scores

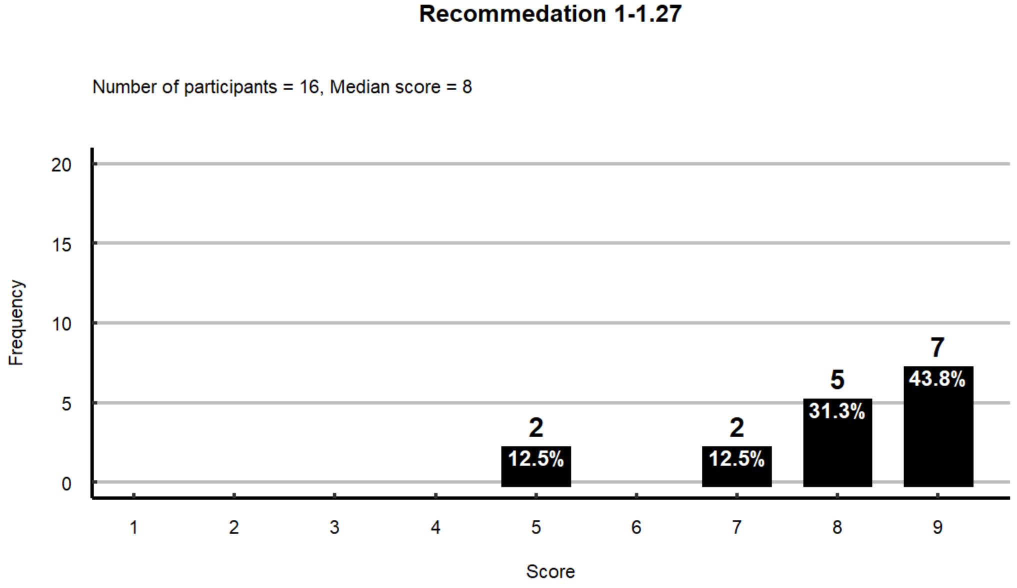

In-vivo[3] or in-vitro[1,2,4], very good consensus, strong recommendation

Comments of voting panelists

5	
5	The delivery to the tracheobronchial tree of nebulized bronchodilators and/or corticosteroids increases when increasing tidal volume and decreasing respiratory rate. However, low tidal volume and high respiratory rate still provide enough delivery to the tracheobronchial tree and can be used.
9	Absolutely agree
8	
7	Increasing Vt and decreasing RR increase eficiency
9	
8	I agree with this recommendation for safety reasons and when bronchodilators are involved. The increased efficacy of aerosol administration with low respiratory rate and high VT has only been demonstrated in in vitro studies. Clinical evidence is still lacking.

9	Changing ventilator settings may have adverse effects. The ventilator settings should foremost be adjusted to meet needs of ventilated patients.
7	The initial settings should play a role in this recommendation. However, for practical reasons, I think it is a good recommendation
9	
9	
8	
8	
9	
9	
8	

Section 1: Aerosol Delivery via Invasive Ventilation for Adult Patients

Recommendation 1-1.28：Increasing inspiratory time and lowering inspiratory flows solely for aerosol delivery is not recommended.

Distribution of voting scores

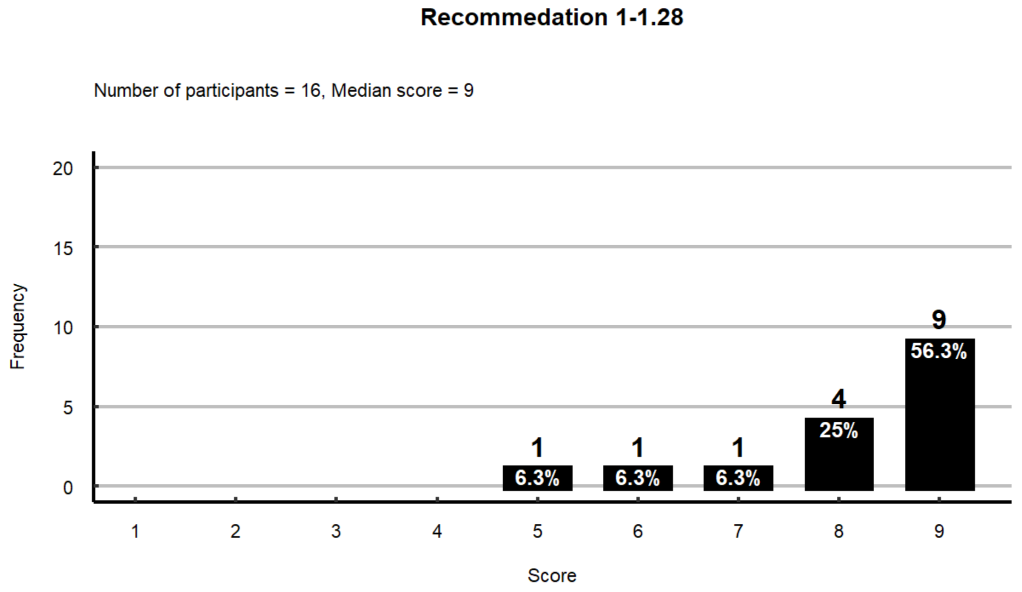

In-vitro[1-6], very good consensus, strong recommendation

Comments of voting panelists

5	
9	
9	
8	
6	Constant insp flow increases the efficacy of delivery
9	
8	
9	Changing ventilator settings may have adverse effects. The ventilator settings should foremost be adjusted to meet needs of ventilated patients.
7	The initial settings should play a role in this recommendation. However, for practical reasons, I think it is a good recommendation
9	
9	
8	

9	
9	
9	
8	This should be combined with 1-1.27

Recommendation 1-1.29：It is not recommended to change the inspiratory flow patterns solely for aerosol delivery.

Distribution of voting scores

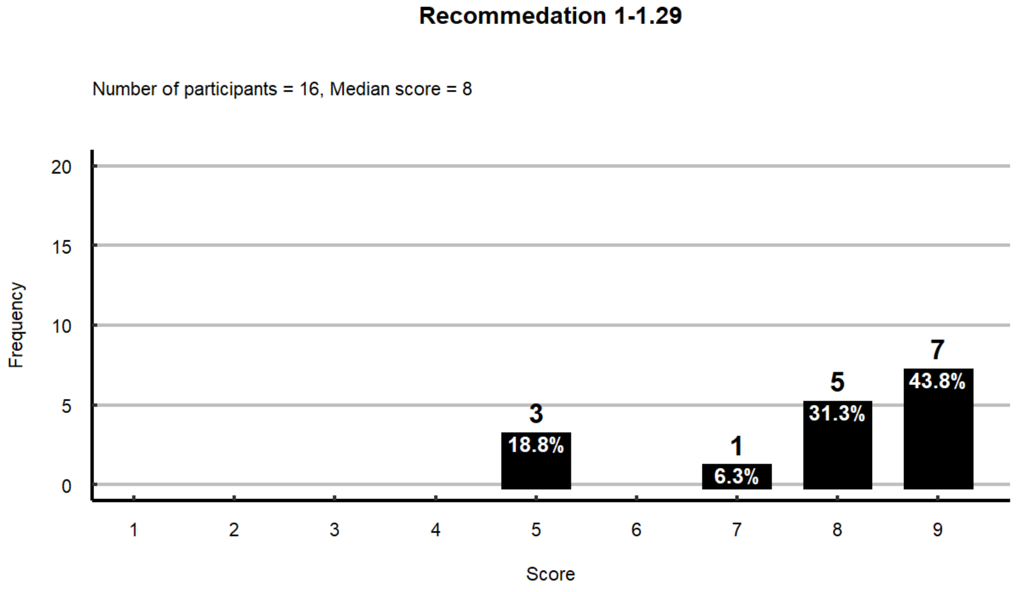

In-vitro[1,3,4], in-vivo[2], very good consensus, strong recommendation

Comments of voting panelists

5	
5	The delivery to the tracheobronchial tree of nebulized bronchodilators and/or corticosteroids is optimized by the administration of constant inspiratory flow. However, delivering a decelarating inspiratoty flow still provide enough delivery to the tracheobronchial tree and can be used.
9	Absolutely agree
8	
5	Constant insp flow increases eficiency of delivery
8	
8	
9	Changing ventilator settings may have adverse effects. The ventilator settings should foremost be adjusted to meet needs of ventilated patients.
7	The initial settings should play a role in this recommendation. However, for practical reasons, I think it is a good recommendation
9	

9	
8	
9	
9	
9	
8	This should be merged with 1-1.27 and 1-1.28

Recommendation 1-1.30： It is not recommended to apply end-inspiratory pause when metered dose inhaler is used during invasive mechanical ventilation.

Distribution of voting scores

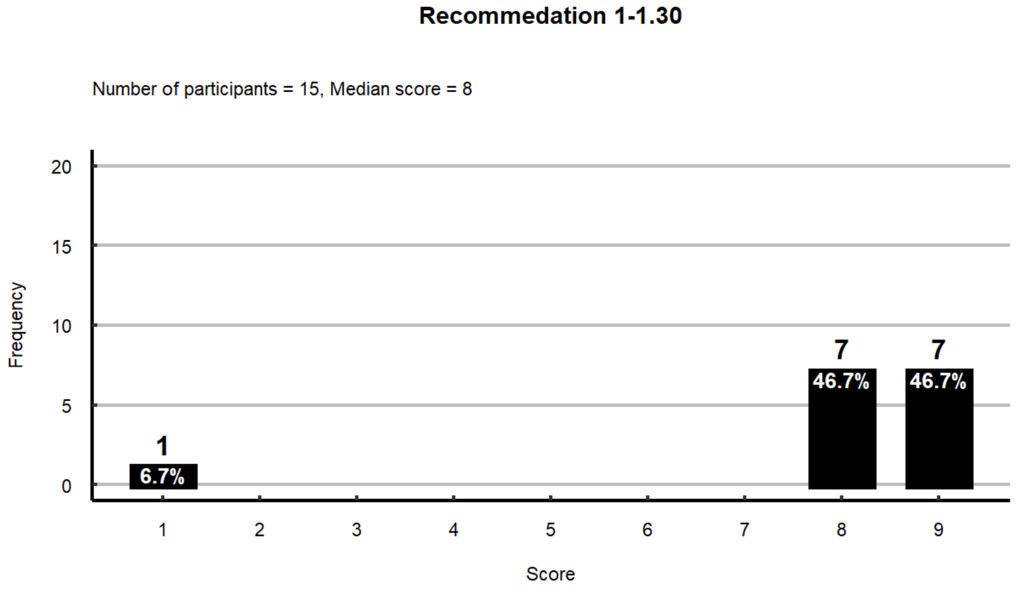

In-vivo[1], very good consensus, strong recommendation

Comments of voting panelists

	No evidence
9	
9	Absolutely agree
8	
8	
9	
8	
8	Weak evidence
8	Even it seems interesting, not enough data
9	
9	
1	
8	
9	Not clear for me why here we specify MDI whereas for the other recommendations on ventilator setting we do not specify.

9	
8	This should be merged with 1-1.27, 1-1.28, and 1.1.29

Recommendation 1-1.31：It is not recommended to change the positive end-expiratory pressure (PEEP) for the sole purpose of improving aerosol delivery efficiency.

Distribution of voting scores

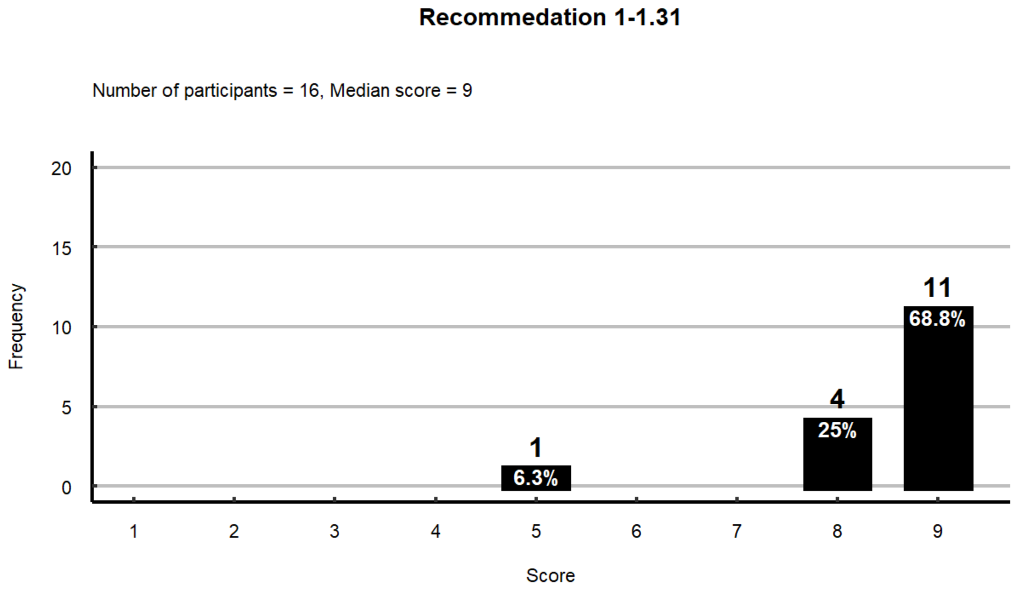

In vitro[1,3], in vivo[2], very good consensus, strong recommendation

Comments of voting panelists

5	It depends of the priority of therapeutic effect of the drug administered.
9	
9	Absolutely agree
8	
9	
9	
9	
9	Changing ventilator settings may have adverse effects. The ventilator settings should foremost be adjusted to meet needs of ventilated patients.
8	Even it seems interesting, not enough data
9	
9	
8	
9	

9	
9	
8	This should be merged with 1-1.27, 1-1.28, 1.1.29, and 1-1.30

Recommendation 1-1.33：Placing a filter on the expiratory limb reduces fugitive aerosols and protects the expiratory sensors. Use of an expiratory filter with frequent changes is recommended.

Distribution of voting scores

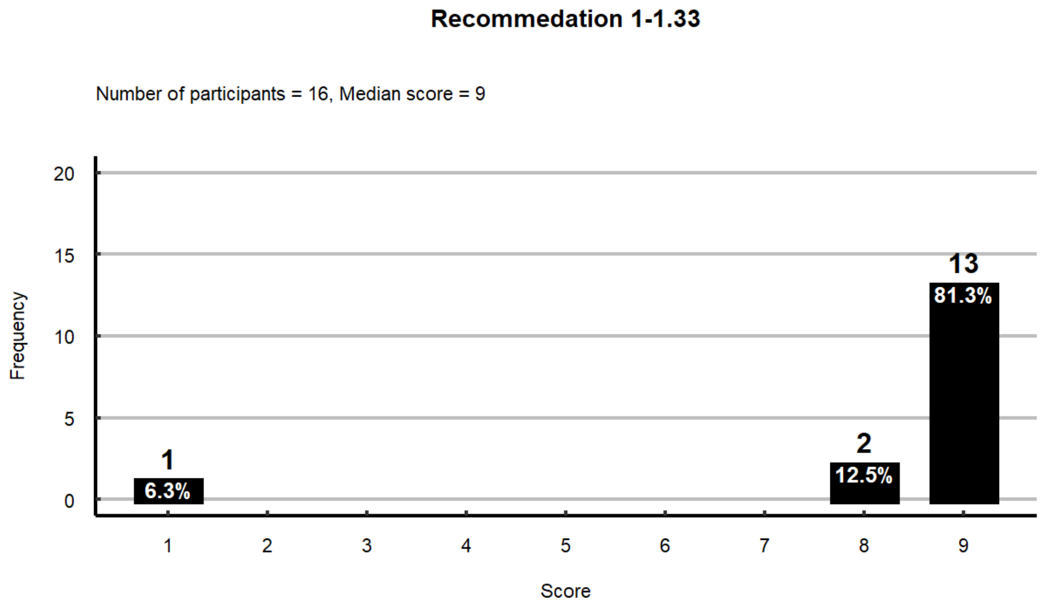

very good consensus, strong recommendation

Comments of voting panelists

1	Filters may be obstructed and cause serious adverse events. For instance, in lipid-base formulations. FDA warnings for occasional cardiac arrests have been reported. I understand it increases costs, but safety should be a priority
9	
9	
9	
9	
9	
9	A hydrophobic pleated breathing filter is recommended for this use. The electrostatic filter is not able to filter massive exhaled drug particles
9	I totally agree with this statement.
9	
9	
8	

9	
9	
9	
9	
8	I would clarify that HEPA filter is preferred. I would add the risk of not changing the filter.

Section 1-2: Antibiotics Aerosol Delivery via Invasive Ventilation for Adult Patients

Recommendation 1-2.1: For antibiotics or other cost-prohibitive medications, changing to a dry circuit immediately before nebulization is recommended.

Distribution of voting scores

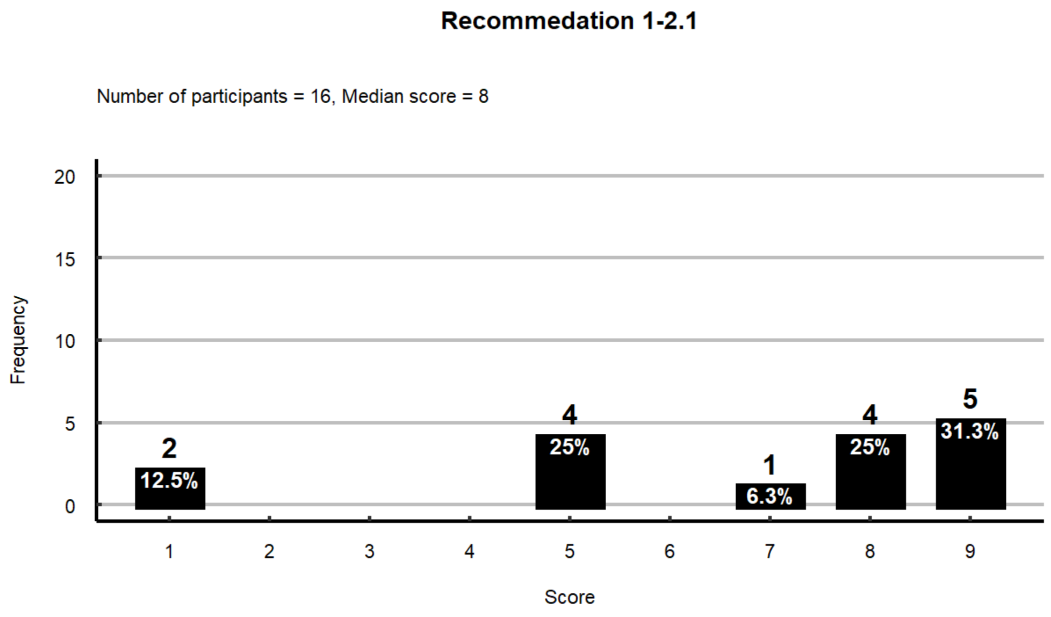

some consensus, weak recommendation

Comments of voting panelists

9	
9	
9	
1	It is unpractical changing the circuit prior each nebulization treatment throughout a course of regimen. Additionally, "break" a ventilator circuit is not recommended and less ventilator circuit change is more likely to induce VAP.
8	
1	
8	I agree with this recommendation. But in patients with severe ARDS, repeated disconnection and reconnection of ventilatory circuits can induce alveolar de-recruitment. This issue should be considered.
8	I totally agree with the statement. It's important to maximize aerosol delivery efficiency when we use cost-prohibitive medications.
8	Even if not a lot of data, we can recommend that I think
5	

5	Changing the circuits is expensive; secondly, it might affect patient ventilation support and cause environmental contamination during the circuit changes. Thus it might not be worthwhile.
9	
7	
5	I would add “if one aims to maximize drug delivery”.
9	
5	Remove other cost-prohibitive medications. This section is for antibiotics.

Recommendation 1-2.2: When delivering inhaled antibiotics for invasively ventilated patients, spontaneous breathing ventilator modes may reduce aerosol delivery efficiency, thus spontaneous breathing should be avoided and volume controlled mode is preferred, and assessing overall benefit/risk ratio especially related to sedation.

Distribution of voting scores

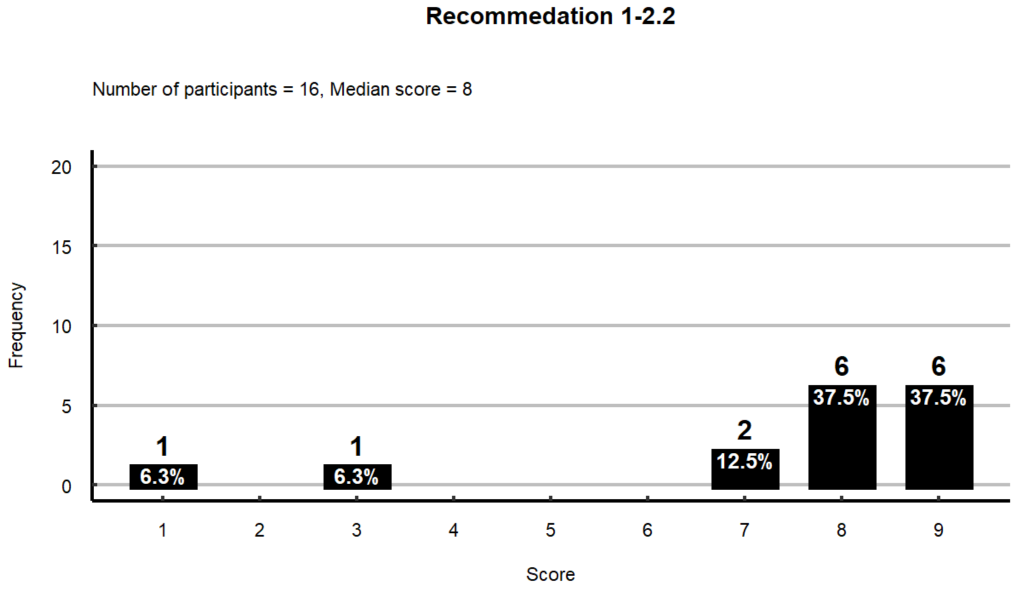

In-vitro[1,4], in vivo[2,3], very good consensus, strong recommendation

Comments of voting panelists

9	
9	
9	
8	
9	
3	
7	Early discontinuation of sedation and daily weaning testing have been shown to improve outcomes in ICU patients (Kress et al, N Eng J Med 2000, Girard et al, Lancet 20080). Thus, there is a conflict between weaning from mechanical ventilation and resuming sedation to apply the volume-controlled mode to optimize the efficiency of aerosol delivery.<br>I think the present recommendation is appropriate for sedated patients ventilated in the controlled mode. In this circumstance, we can recommend the volume-controlled mode with constant flow instead of the pressure-controlled mode

	with decelerating flow. In patients in whom the ventilatory weaning process begins, I agree that weaning from mechanical ventilation should take priority over nebulization, and physicians should assess overall benefit/risk ratio especially related to sedation.
1	I disagree to change ventilator modes and to start/increase sedation of the patient for the sole purpose of increasing aerosol delivery especially since the evidence is weak.<br>If there are situations, when the nebulization is the main objective (more than the ventilation), the recommendation could be different.
8	
8	
7	
8	
8	
9	
9	
8	

Recommendation 1-2.3： When delivering inhaled antibiotics for invasively ventilated patients, it is recommended to set tidal volume of 8ml/kg of patient's predicted body weight, and the clinician must weigh the benefit/risk ratio of increasing tidal volume for improving aerosol delivery with the risk of high tidal volume.

Distribution of voting scores

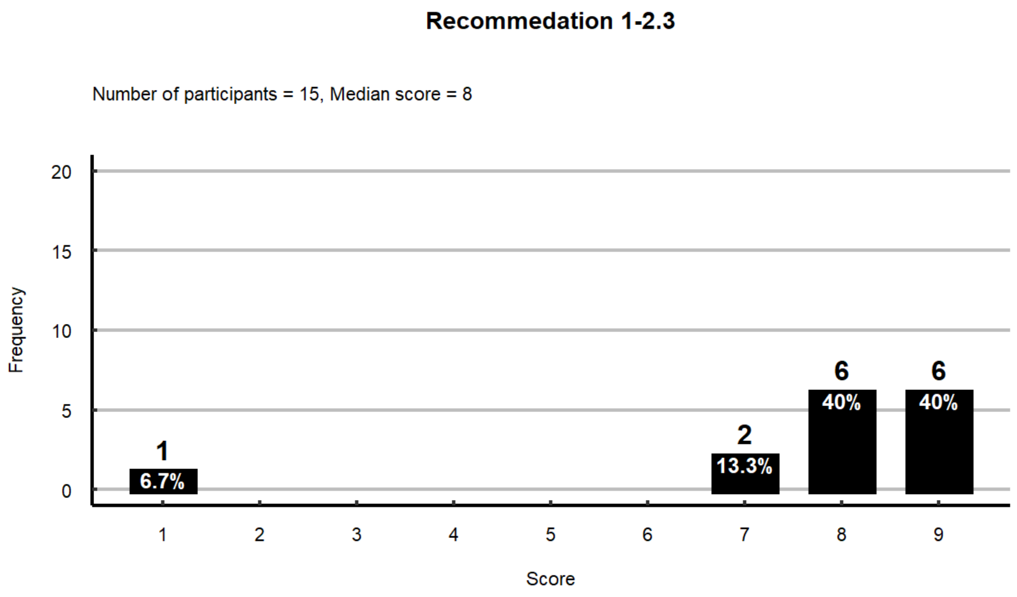

In-vivo[3], in-vitro[1,2,4], very good consensus, strong recommendation

Comments of voting panelists

No score	8ml/kg TV for 1 hour is not a risk for VILI !Fully agree (9) with first sentence. I disagree (1) with the added comment. Delete, added red statement. Recommendations must be unequivocal.
9	
9	
8	
9	
1	
8	
9	I agree with this statement
8	
7	No clear demonstration/evidence about 8ml/kg instead of another value.
7	
9	
8	

9	
8	
8	

Recommendation 1-2.4：When delivering inhaled antibiotics for invasively ventilated patients, it is recommended to keep respiratory rates at 12-15 breaths/min.

Distribution of voting scores

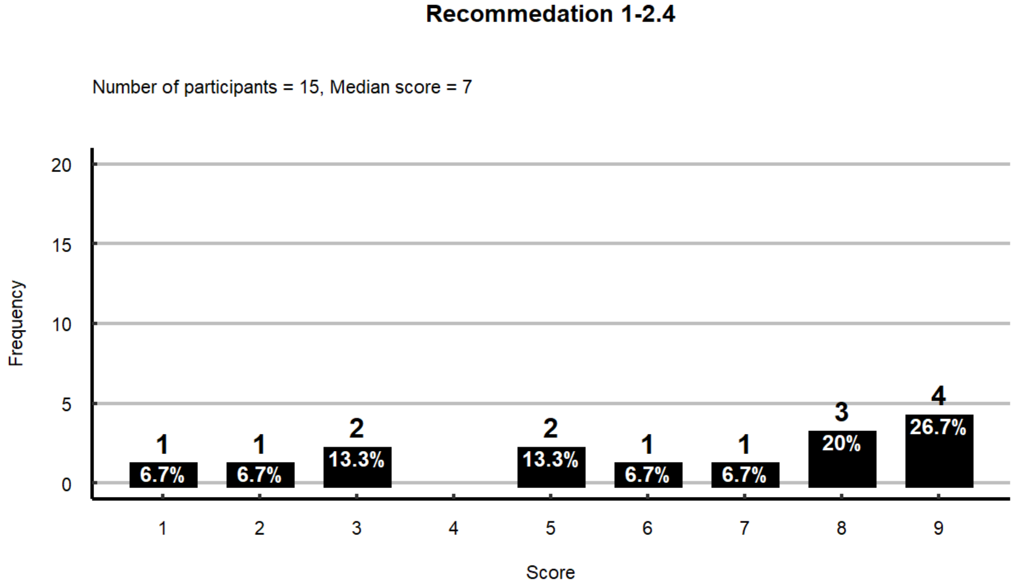

In-vivo[3], in-vitro[1,2,4], no consensus, no recommendation

Comments of voting panelists

	12 or 15?Agreement 9 for 12.Agreement 5 for 15.
9	
9	
5	
9	
3	
8	In patients with severe brain injury, decreasing respiratory rate may induce intracranial hypertension due to hypercapnia. I suggest adding in the recommendation " the clinician should assess the benefit/risk ratio related to hypercapnia.
5	It depends on the I:E ratio. More evidence is needed.
6	Not strong enough and why 12-15?
3	Too low level of evidence and potential risk for the patient
2	First, there is no evidence to show respiratory rates at 12-15 bpm is better than other respiratory rates; second, it might be risky to set such low respiratory rates for patients who have hypercapnic respiratory rates. The respiratory rate setting needs to be set/adjusted based on clinical need.

9	
8	
7	
1	Not recommended but preferred, if possible. The benefit/risk ratio of decreasing respiratory rate should be weighed.
8	

Recommendation 1-2.5：When delivering inhaled antibiotics for invasively ventilated patients, it is recommended to keep inspiratory flow below 40L/min.

Distribution of voting scores

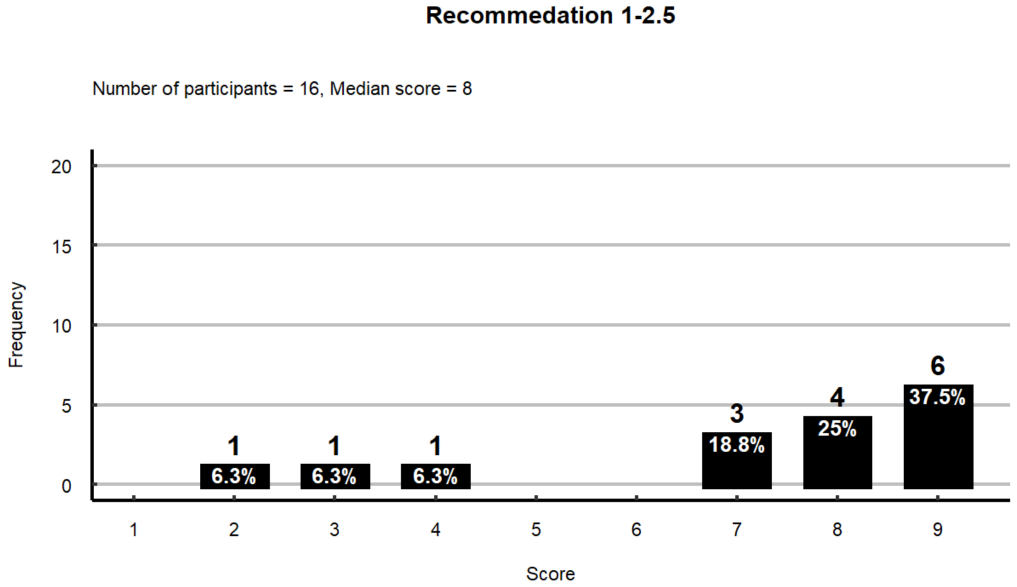

In-vitro[1-6], very good consensus, strong recommendation

Comments of voting panelists

9	
9	
9	
7	
9	
4	
8	I agree with decreasing the inspiratory flow rate to optimize the efficiency of aerosol delivery. But inspiratory flow rate depends on tidal volume and inspiratory time which depends on respiratory rate and I/E.
8	I agree with this statement, but the clinician must first weigh the benefit/risk ratio of keeping inspiratory flow below 40L/min for improving aerosol delivery. More clinical data are needed
7	Not strong enough
3	Too low level of evidence and potential risk for the patient
2	
7	
8	

9	
9	
8	Merge with 1-2.4

Recommendation 1-2.6： When delivering inhaled antibiotics for invasively ventilated patients, it is recommended to use inspiratory to expiratory ratio of 50%.

Distribution of voting scores

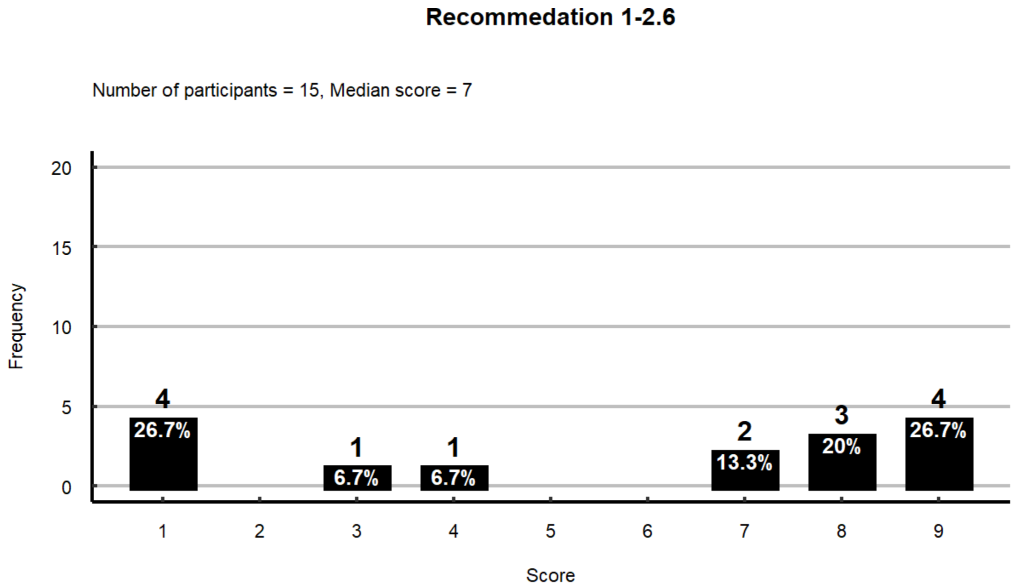

In-vitro[1-6], no consensus, no recommendation

Comments of voting panelists

9	Suggestion to change to "…Prolong I:R to at least 50%"Or "equal or lower to 50%"
9	
9	
4	
9	
1	The potential risks to patient with this I:E are significant and should not be done just to administer antibiotics
7	I agree with the comments of the experts in the second round and we should add a note of caution because reversing inspiration to expiration can induce autopeep and barotrauma in patients with obstructive disease
1	I disagree to recommend an inspiratory to expiratory ratio of 50% for the sole purpose of increasing aerosol delivery especially since the evidence is weak. I:E ratio of 50% may have adverse effects (e.g. increasing sedation, intrinsic PEEP in obstructive patient…)
7	
3	Too low level of evidence and potential risk for the patient

1	First, I:E=1 may cause barotrauma, the risk is over the benefit (improving aerosol delivery); second, how much more aerosol deposition is gained by I:E of 1 compared to 1:E of 1:2?
8	
8	
	I/E ratio is equivalent to inspiratory flow setting. I would not recommend anything, I/E ratio has to be be set for flow being below 40 L/min.
1	Not recommended but preferred, if possible and tolerable for the patient. The benefit/risk ratio of using I/E ratio of 50% rate should be weighed
8	Merge with 1-2.4 1-2.5

Recommendation 1-2.7： When delivering inhaled antibiotics for invasively ventilated patients, it is recommended to use a constant inspiratory flow.

Distribution of voting scores

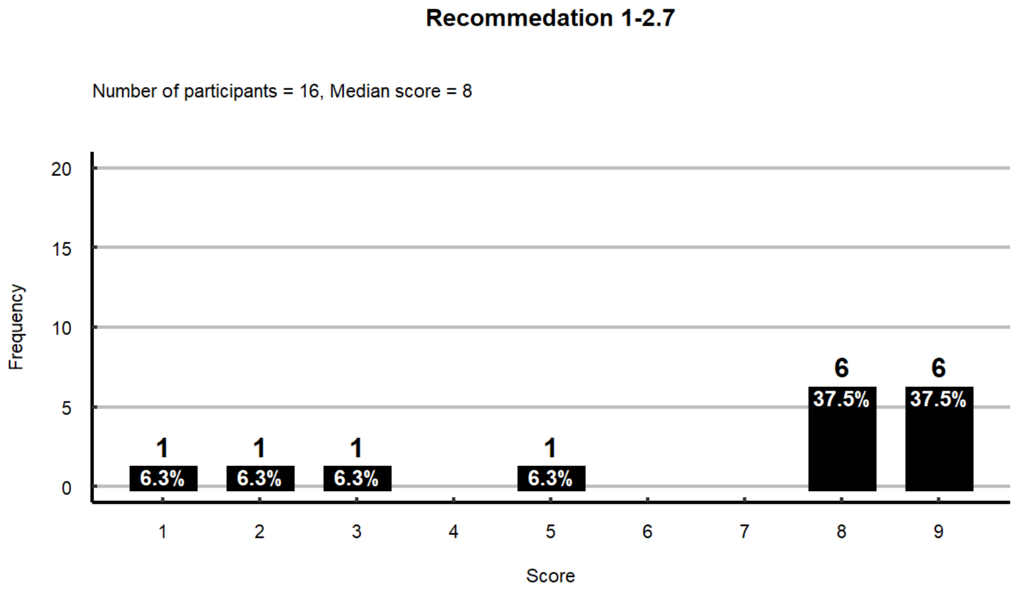

In-vitro[1,3,4], in-vivo[2], some consensus, weak recommendation

Comments of voting panelists

9	
9	
9	
8	
9	
1	
8	It is difficult to control the inspiratory flow pattern unless patients are sedated. A constant flow can only be applied when the patient is in a controlled ventilatory mode. So clinicians should assess the overall benefit/risk ratio of sedation before constant flow can be applied".
8	More clinical data are needed
8	
3	There are not enough clinical data to support this, particularly in terms of aerosol distribution/clinical efficacy. For example, an increasing flow during inspiratory time could be theoretically~~theoretical~~ better to target distal regions~~region~~ than a constant flow (considering a similar tidal volume and inspiratory time).

2	
8	
5	
9	
9	
8	Merge with 1-2.4, 1-2.5, and 1-2.6

Recommendation 1-2.8：When delivering inhaled antibiotics for invasively ventilated patients, it is recommended to set end-inspiratory pause at 20%.

Distribution of voting scores

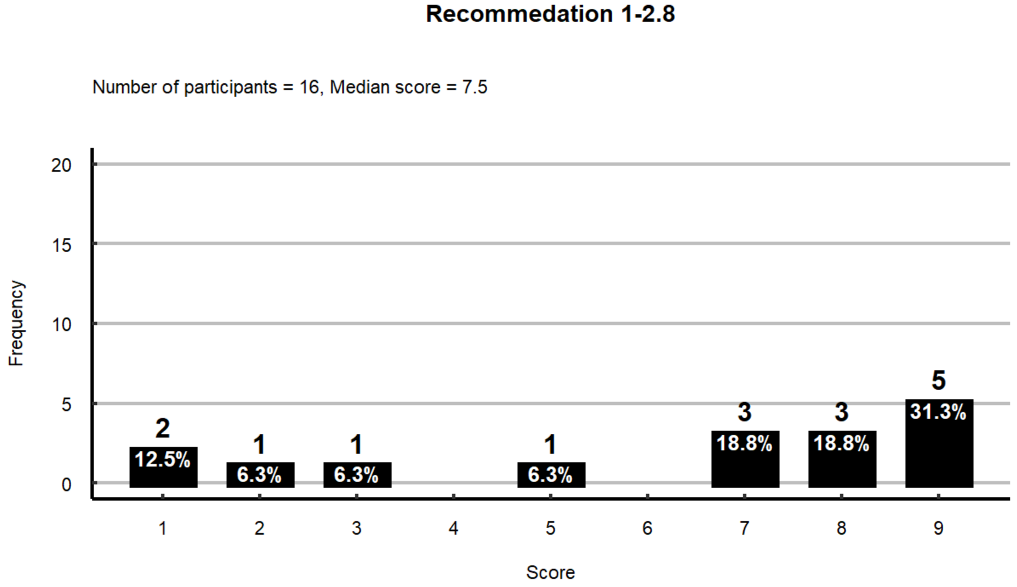

In-vivo[1], no consensus, no recommendation

Comments of voting panelists

9	
9	
9	
2	
9	
1	No evidence that end inspiratory pause improves aerosol delivery efficiency of antibiotics
7	
8	I totally agree with the statement. It's important to maximize aerosol delivery efficiency when we use cost-prohibitive medications.<br>Volume controlled ventilation is rarely used without an end-inspiratory pause. More clinical data are needed.
7	
3	There is no data demonstrating the influence of pause at 20% on drug efficacy using nebulizer to my knowledge. The paper from Zanudin et al (Zainudin BM, Tolfree SE, Short M, Spiro SG. Influence of breathing pattern on lung deposition and bronchodilator response to nebulised salbutamol in patients with

	stable asthma. Thorax. 1988 Dec;43(12):987-91. doi: 10.1136/thx.43.12.987. PMID: 3238642; PMCID: PMC461611.) concludes that inhaling a nebulised aerosol by tidal breathing, is as effective as tidal breathing with deep breaths with or without a breath hold. But it is with a bronchodilatator…
1	
8	
7	
5	
9	
8	Merge with 1-2.4, 1-2.5, 1-2.6, and 1-2.7

Recommendation 1-2.9：When delivering inhaled antibiotics for invasively ventilated patients, it is recommended to set a positive end-expiratory pressure (PEEP) at 5-10 cmH_2O.

Distribution of voting scores

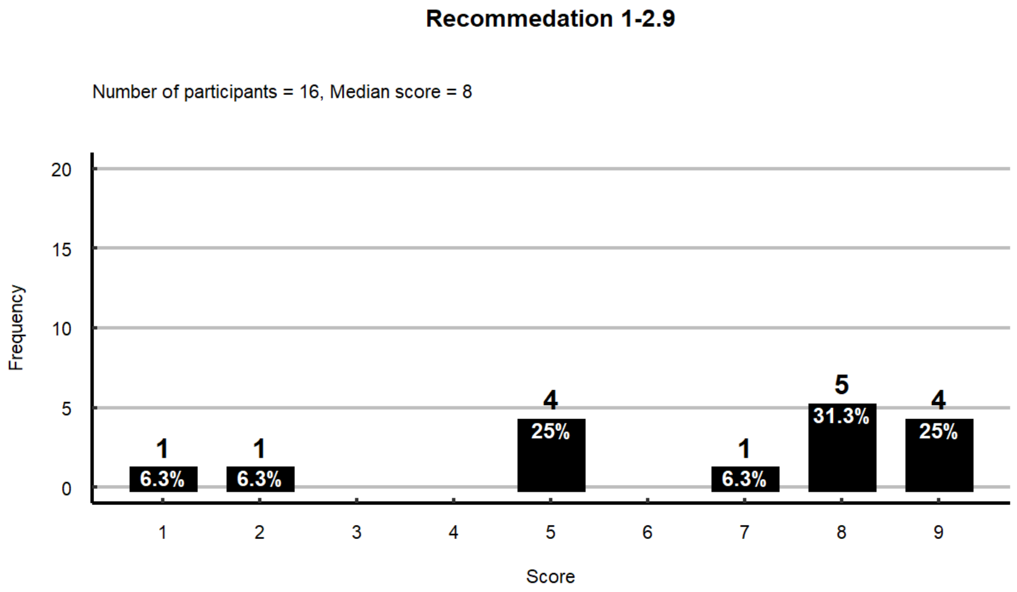

In vitro[1,3], in vivo[2], some consensus, weak recommendation

Comments of voting panelists

9	
9	
9	
8	
9	
5	Only if this is the level of PEEP required to ventilate the patient
5	
8	PEEP levels are rarely set lower than 5 cmH2O. The clinician must first weigh the benefit/risk ratio of increasing a PEEP level (especially high level) for improving aerosol delivery efficiency.
7	
5	No datas supporting this recommendation in mechanical ventilation but few clinical datas reported in ambulatory patients.
1	
8	
8	

5	
2	If a patient has a PEEP of 18 mm H2O for oxygenation purpose, we cannot recommend decreasing the PEEP only for nebulization.<br>Should be changed for "in patients without pre-existing PEEP, ….". Or precise that PEEP setting, if above 10 mm H2O, should not be changed for nebulization.
8	Merge with 1-2.4, 1-2.5, 1-2.6, 1-2.7, and 1-2.8

Section 2: Aerosol Delivery via **Non-invasive Ventilation** for Adult Patients

Section 2: Aerosol Delivery via Non-invasive Ventilation for Adult Patients

Recommendation 2.1：Placing the nebulizer in-line with noninvasive ventilation has similar or higher aerosol delivery efficiency than using the nebulizer with a mask or mouthpiece. Interrupting or discontinuing noninvasive ventilation to administer aerosol via a mask or mouthpiece is unnecessary and not recommended.

Distribution of voting scores

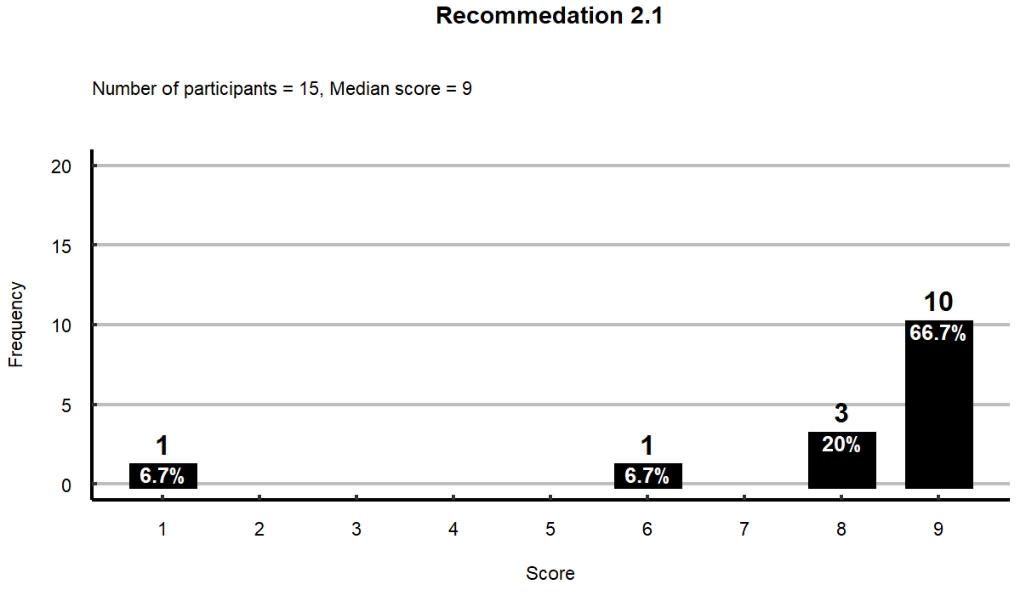

In vivo [1,3-10], in vitro[2,9], very good consensus, strong recommendation

Comments of voting panelists

9	
9	
8	
9	
9	
8	
9	I totally agree with this statement for patient safety. More clinical data are needed to confirm that interrupting or discontinuing NIV is unnecessary.
9	
9	
8	
1	Just a question: At the same time? NIV + NEB?

	Placing the nebulizer in-line with noninvasive ventilation has similar or higher aerosol delivery efficiency….<br>In vivo studies NIV when associated with Neb decrease the aerosol deposition….<br>França EE, de Andrade AFD, Cabral G, et al. Nebulization associated with bi-level noninvasive ventilation: analysis of pulmonary radioaerosol deposition. Respir Med 2006;100:721-8. 10.1016/j.rmed.2005.07.012 [PubMed] [CrossRef] [Google Scholar]<br>47. Galindo-Filho VC, Ramos ME, Rattes CS, et al. Radioaerosol pulmonary deposition using mesh and jet nebulizers during noninvasive ventilation in healthy subjects. Respir Care 2015;60:1238-46. 10.4187/respcare.03667 [PubMed] [CrossRef] [Google Scholar]<br>48. Galindo-Filho VC, Alcoforado L, Rattes C, et al. A mesh nebulizer is more effective than jet nebulizer to nebulize bronchodilators during non-invasive ventilation of subjects with COPD: A randomized controlled trial with radiolabeled aerosols. Respir Med 2019;153:60-7. 10.1016/j.rmed.2019.05.016 [PubMed] [CrossRef] [Google Scholar]
9	
9	
9	
6	

Recommendation 2.3: When placing the continuous nebulizer in-line with noninvasive ventilation, vibrating mesh nebulizer is more efficient in aerosol delivery than jet nebulizer, with no influence on flows or fraction of inspired oxygen. When available, vibrating mesh nebulizer is recommended over jet nebulizer.

Distribution of voting scores

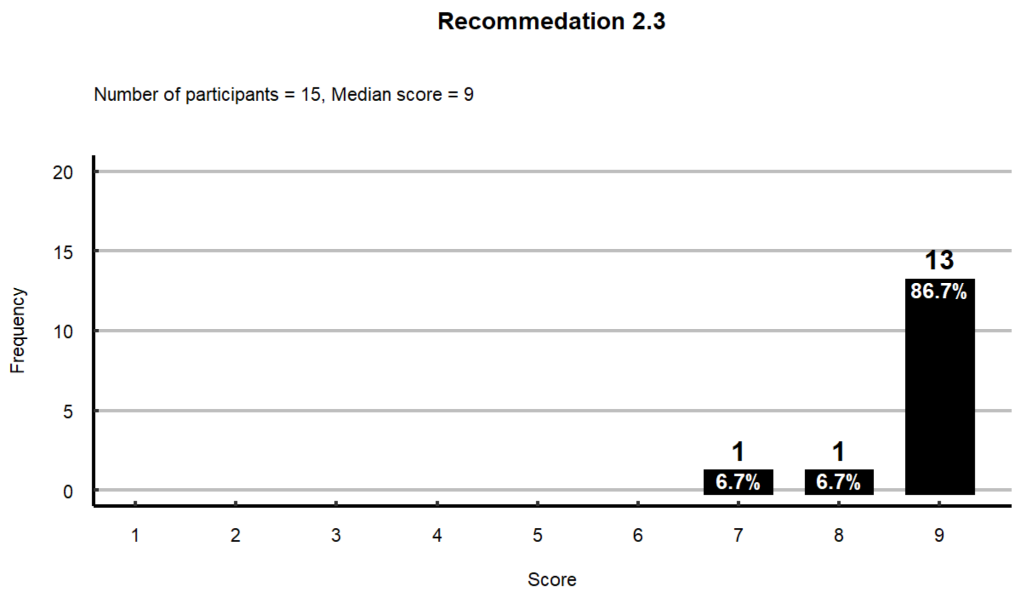

In vitro[1-3,5,7-11,13], in vivo[4,6-7,11-12], perfect consensus, strong recommendation

Comments of voting panelists

9	
9	
8	
9	
9	
9	
9	I totally agree with this statement
9	
9	
9	
9	
9	
9	

9	
7	

Recommendation 2.7：The aerosol delivery efficiency is less affected by the fill volume in the vibrating mesh nebulizer than the continuous jet nebulizer. For continuous jet nebulizers, more dilution is associated with greater aerosol delivery. Increasing fill volume for the sole purpose to improve aerosol delivery efficiency is not recommended.

Distribution of voting scores

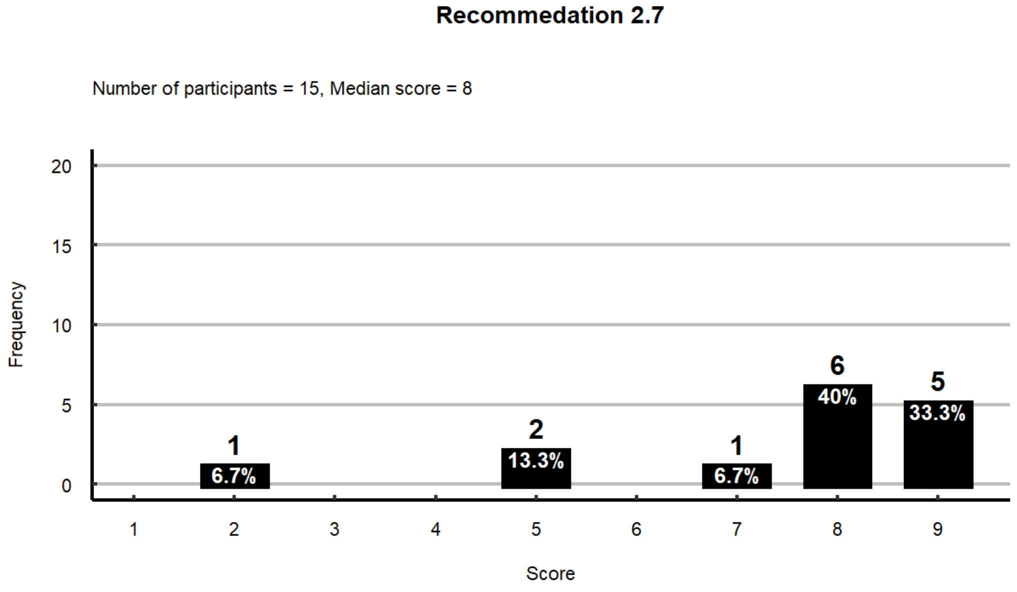

In vitro [1-4], in vivo[2,4], very good consensus, strong recommendation

Comments of voting panelists

9	
9	
8	
9	
8	
8	Aerosol delivery efficiency with JN is affected by fill volume due to its high residual volume, but not affected by the dilution. I suggest making the same recommendation as that for invasive mechanical ventilation (Recommendation 1-1.20.1) as follows:<br>For the jet nebulizer with a residual volume > 0.5 ml, aerosol delivery efficiency is improved with a higher fill volume, but changing fill volume for

	the sole purpose of improving aerosol delivery efficiency is not recommended for FDA approved inhaled medication.
2	I totally agree regarding the first part of the statement (vibrating mesh nebulizer less affected by the fill volume). But disagree for the second part (regarding jet nebulizers).<br>More clinical data are needed.
8	
5	
8	
7	
8	
9	
9	
5	How is this different from the similar recommendation for invasive ventilation

Section 2: Aerosol Delivery via Non-invasive Ventilation for Adult Patients

Recommendation 2.8：The aerosol delivery efficiency is similar between CPAP and noninvasive ventilation, changing the mode for the sole purpose of increasing aerosol delivery is not recommended.

Distribution of voting scores

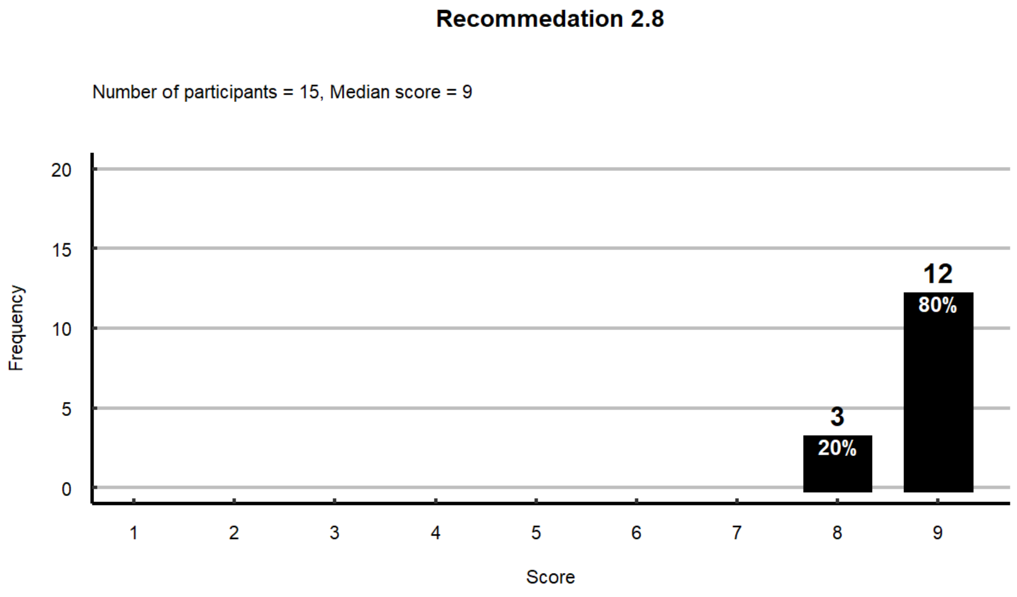

In vitro[2], in vivo[1], perfect consensus, strong recommendation

Comments of voting panelists

9	
9	
9	
9	
9	
8	
9	Changing ventilator modes may have adverse effects (different indications). The ventilator modes should foremost be set to meet needs of ventilated patients
9	The indications are different between both settings then I'm not sure about the meaning of this recommendation
8	
9	

9	
9	
9	
9	
8	

Section 2: Aerosol Delivery via Non-invasive Ventilation for Adult Patients

Recommendation 2.9： When continuous nebulizer is placed between the mask and the exhalation valve during noninvasive ventilation with a single limb circuit, the aerosol delivery efficiency increases as IPAP increases or EPAP decreases. Changing the parameters for the sole purpose to improve aerosol delivery efficiency is not recommended.

Distribution of voting scores

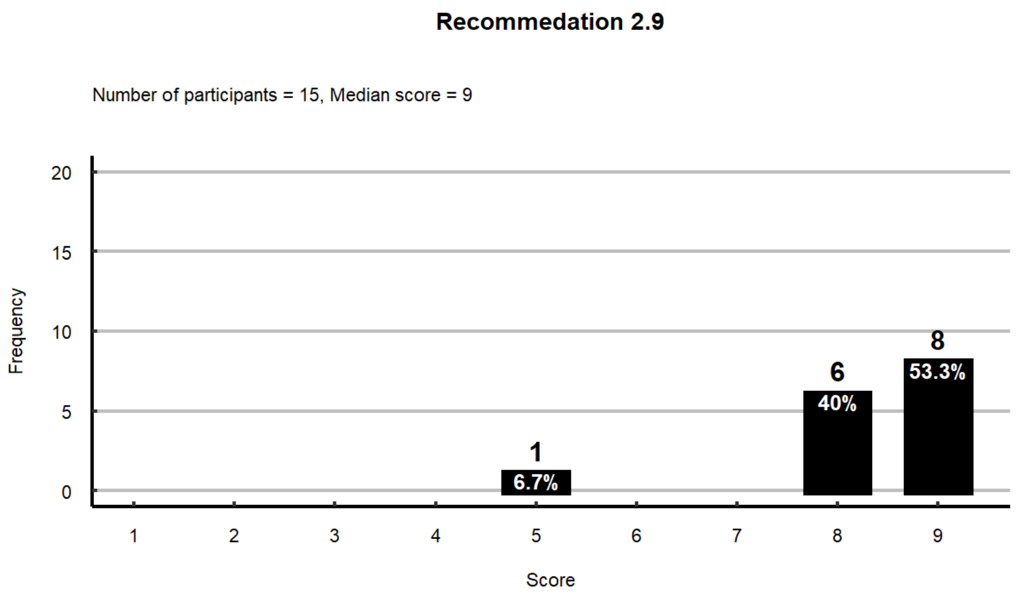

In vitro[1,3-5], in vivo[2], very good consensus, strong recommendation

Comments of voting panelists

9	
9	
8	
9	
9	
8	
9	I agree with the statement.<br>The clinician must first weigh the benefit/risk ratio of changing IPAP and EPAP levels for improving aerosol delivery efficiency. The ventilator parameters should foremost be set to meet needs of ventilated patients.
8	

8	
8	In the bench studies, aerosol delivery efficiency increases as IPAP increases or EPAP decreases is because Vt increases by increasing IPAP or decreasing EPAP. However, clinically, changing the pressure settings does not always result in Vt changes, as Vt also depends on patient’s spontaneous breathing efforts.
5	
9	
9	
9	
8	

Section 2: Aerosol Delivery via Non-invasive Ventilation for Adult Patients

Recommendation 2.10： When a continuous nebulizer is placed in-line with noninvasive ventilation, the aerosol delivery efficiency is higher with a non-vented mask than a vented mask. Aerosol administration with a vented mask is not recommended.

Distribution of voting scores

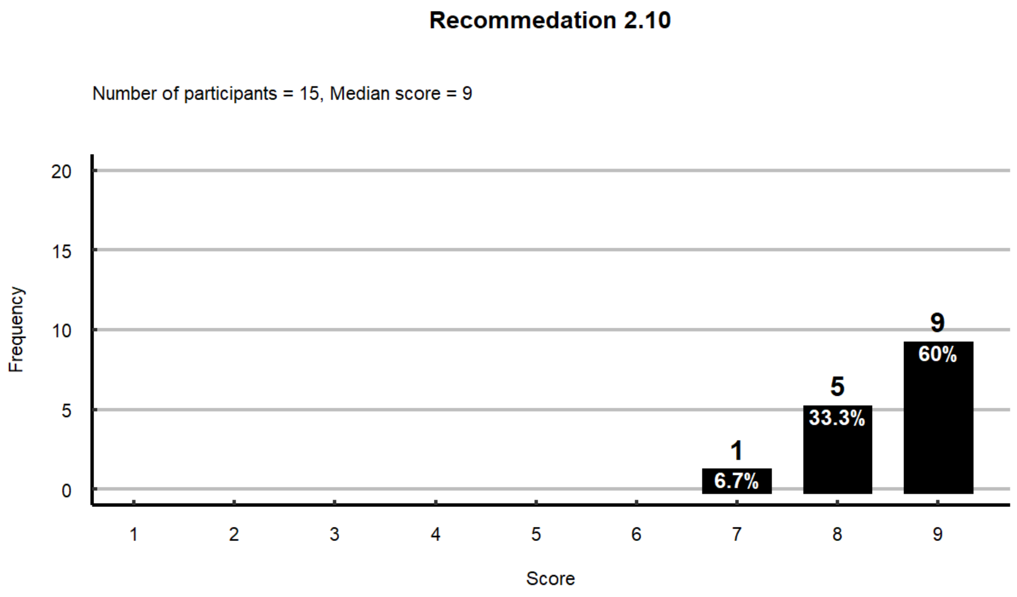

In vitro[1-3], perfect consensus, strong recommendation

Comments of voting panelists

9	
9	
8	
9	
9	
9	
9	I totally agree with this statement. More clinical data are needed.
9	
7	
8	
8	
9	

8	
9	
8	

Section 2: Aerosol Delivery via Non-invasive Ventilation for Adult Patients

Recommendation 2.11：When non-vented mask is used during noninvasive ventilation, the aerosol delivery efficiency with optimal position is similar with the single limb and dual limb circuits. There is no recommendation for the use of single versus dual limb circuits for aerosol delivery.

Distribution of voting scores

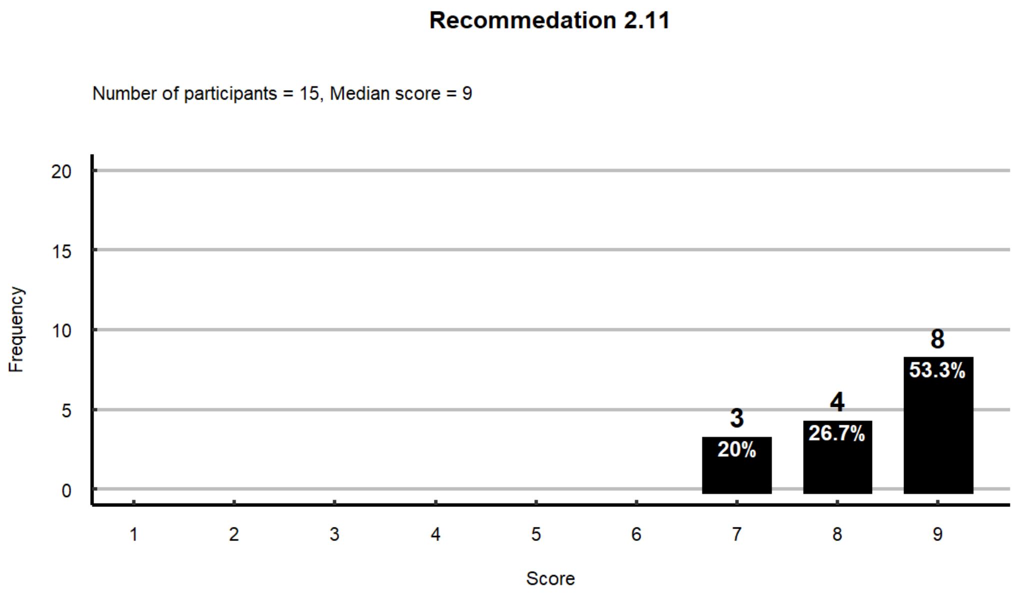

In vitro[1], perfect consensus, strong recommendation

Comments of voting panelists

9	
9	
7	
9	
9	
7	This recommendation is confusing because the optimal position for single limb and dual limb is unknown.<br>The in vitro study by Tan et al, which was under review in the first round, is now published in Respir Care 2022. This study showed that the optimal position was between non-vented mask and 15 cm from the exhalation port in the single-limb circuit or 15 cm from the Y-piece in the inspiratory limb of the

	dual-limb circuit. Low level evidence with a recommendation based on a single in vitro study.
8	I agree with this statement.<br>More clinical data are needed.
8	
9	
8	
7	
9	
9	
9	
8	

Section 3: Aerosol Delivery via **High-flow Nasal Cannula** for Adult Patients

Section 3: Aerosol Delivery via High-flow Nasal Cannula for Adult Patients

Recommendation 3.1：The aerosol delivery efficiency with a nebulizer via high-flow nasal cannula at flow ≤ 35 L/min is similar to that with a nebulizer and a mask or mouthpiece. Discontinuing high-flow nasal cannula treatment to administer nebulizer with a mask or mouthpiece is not recommended.

Distribution of voting scores

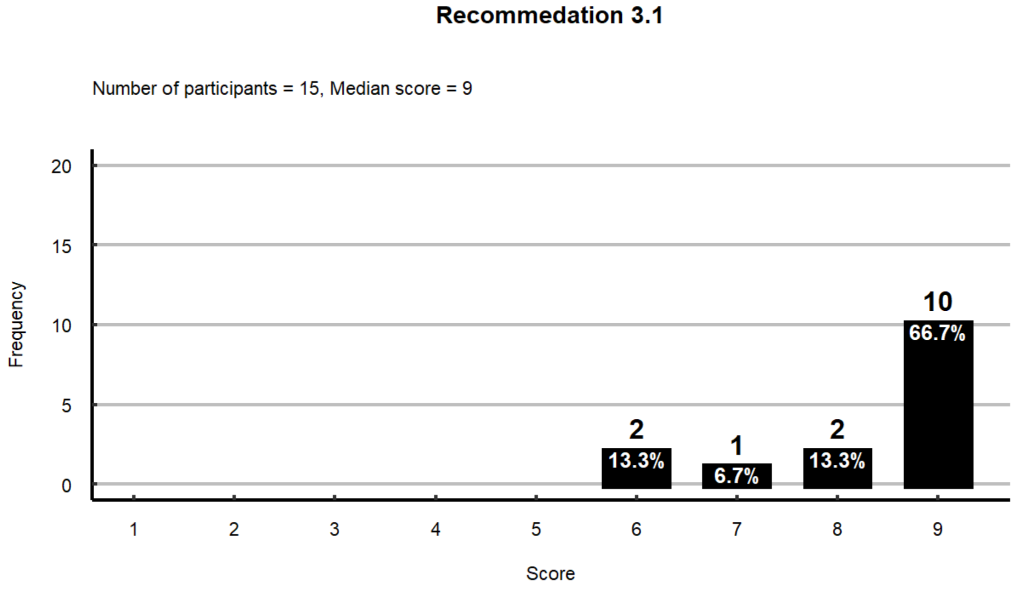

In vivo [1-4], in vitro[5], very good consensus, strong recommendation

Comments of voting panelists

9	
9	
6	
9	
9	
8	
9	I totally agree with this statement for patient safety. More clinical data are needed.
9	
7	
9	
8	

9	
9	I think this only applies to bronchodilators : other drugs have not been evaluated.
9	
6	

Section 3: Aerosol Delivery via High-flow Nasal Cannula for Adult Patients

Recommendation 3.4： Nebulizers are recommended to be placed at the inlet of humidifier at HFNC flows ≥ 10 L/min.

Distribution of voting scores

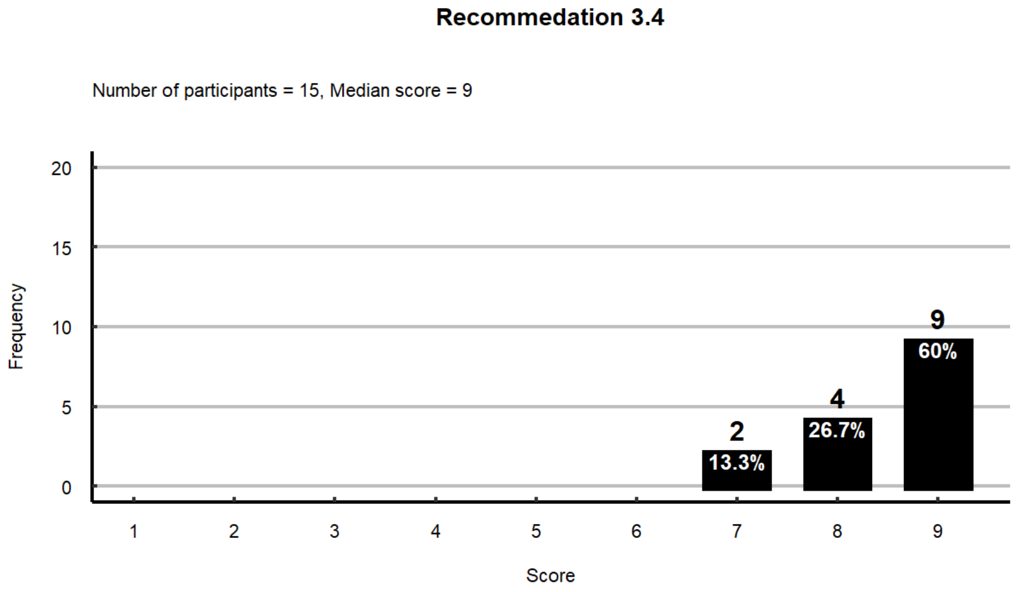

In vitro[1,2], perfect consensus, strong recommendation

Comments of voting panelists

7	
8	
8	
7	
9	
9	
9	Low evidence. More clinical data are needed
9	
9	Yes but with caution when nebulize drugs sensitive to the temperature because the temperature in the humidifier is high and could destroy/modify the active compounds activity (for biological drugs for example…)…
9	
8	
9	

9	
9	
8	

Section 3: Aerosol Delivery via High-flow Nasal Cannula for Adult Patients

Recommendation 3.5： When metered dose inhaler is placed in-line with high-flow nasal cannula, it is recommended to be used with a spacer and placed close to nasal cannula with the aerosol plume directed toward the patient.

Distribution of voting scores

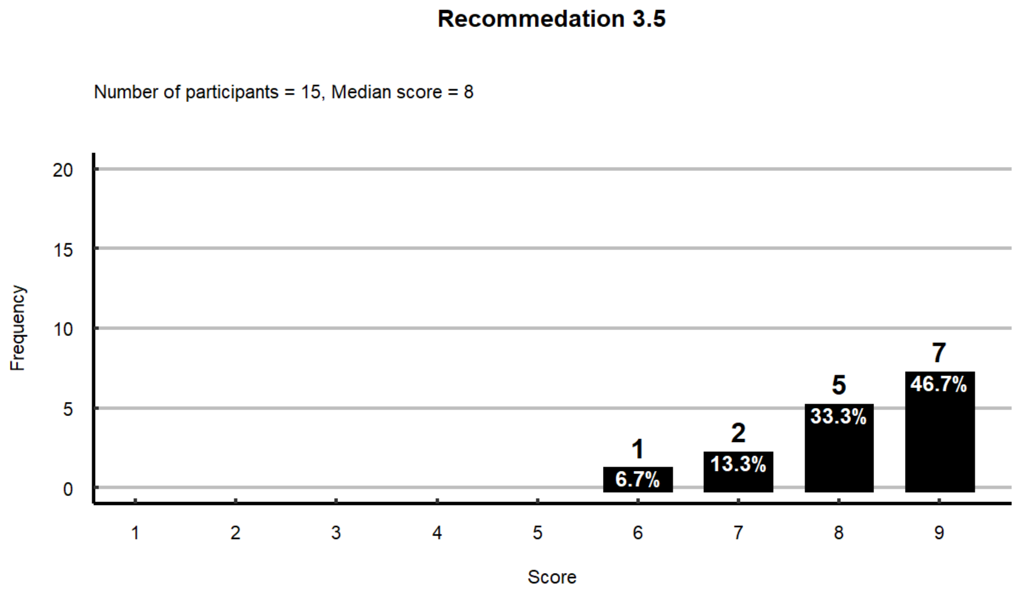

In vitro[1], very good consensus, strong recommendation

Comments of voting panelists

9	
9	
6	
9	
7	
8	
8	Low evidence. More clinical data are needed
7	
9	
8	
9	
8	
9	

9	
8	

Section 3: Aerosol Delivery via High-flow Nasal Cannula for Adult Patients

Recommendation 3.7： Using heliox via high-flow nasal cannula for the sole purpose of improving aerosol delivery is not recommended.

Distribution of voting scores

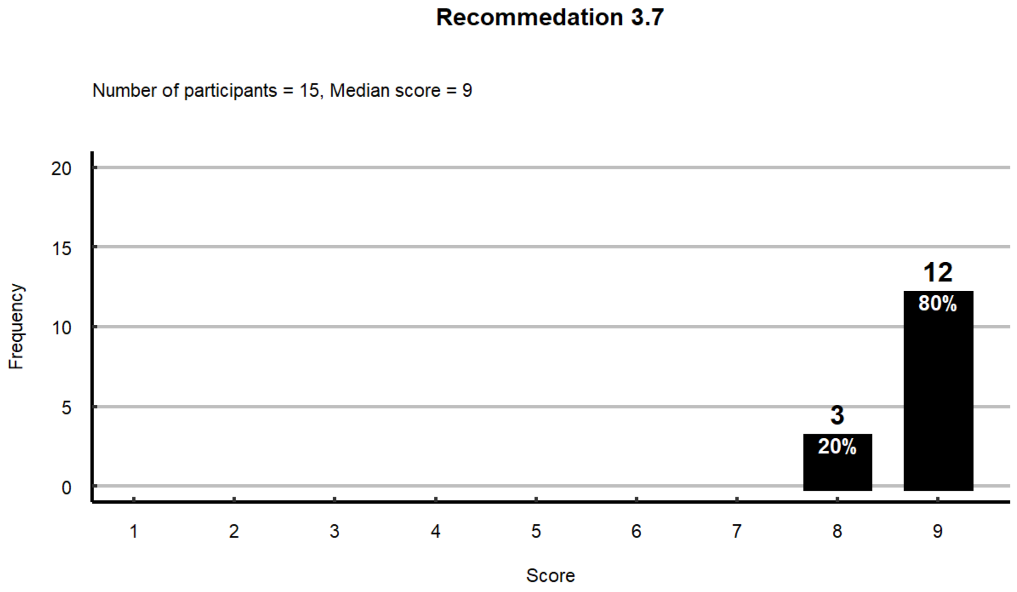

In vitro[1], perfect consensus, strong recommendation

Comments of voting panelists

9	
9	See also my comments above about heliox
8	
9	
9	
8	
9	Too expensive and low evidence, I totally agree with this statement.
9	
9	
9	
9	Using heliox as the carrier gas for the sole purpose of increasing aerosol therapy delivery is not cost-effective<br>Heliox can be used to relieve dyspnea in patients with severe asthma and others severe airway obstruction

9	
9	
9	
8	

Appendix 8

Meeting Minutes for nebulization consensus online meeting

9-11am CST 6/30/22

Attendees: Jim Fink, Rajiv Dhand, Antoni Torres, Paolo Pelosi, Jordi Rello, Ariel Berlinski, Bing Dai, Huiling Lin, Qin Lu, Shan Lyu, Kai Liu, Guoqiang Jing, Jie Li

1. Jie presented the process of generating nebulization consensus:
    a. Background, literature search and review, voting panel selection, and recommendation voting process.
2. Participants reviewed the recommendations for Aerosol delivery via invasive ventilation (non-antibiotics):
    a. 6 reached perfect consensus, 13 reached very good consensus
        i. Dr. Berlinski suggested: 1) some of the recommendations with relevant content can be merged into one; 2) "**Recommendation 1-1.17：**In ventilated patients, using a continuous jet nebulizer means adding compressed gas independent of the ventilator. The effect on tidal volume, FiO2 etc makes this practice unacceptable. The empirical compensations on ventilator settings may be dangerous and should be avoided. If no integrated inspiration-synchronized jet nebulizer is available, the use of continuous jet neb in ventilated patients is not recommended." is too stringent, Dr. Berlinski will draft revised language and send it to the working group. the working group will share all revised recommendations with the entire voting panel.
            ▪ **Dr. Michotte:** Concerning the recommendation 1-1.17: Inspiration-synchronized jet nebulizer mode on ventilators performs sometimes poorly (Respir Care October 2014, 59 (10) 1508-1516) and require longer nebulization time (Respir Care 2014;59(10):1494 –1500). Therefore, if available, I would recommend using a continue jet nebulizer mode rather than inspiration-synchronized nebulization mode
            ▪ **Dr. Lin:** A jet nebulizer with an external gas source is commonly used in Asia (China, Taiwan, Singapore), perhaps due to a shorter nebulization time. Other than that, using a jet nebulizer with external gas flow is unacceptable, I would also recommend that using a

continue mode rather than inspiration-synchronized nebulization by the ventilator integrated function.

- **Dr. Berlinski:** The use of in-line continuous jet nebulizer (unless integrated with the ventilator) results in changes in tidal volume, inspiratory flow patterns and FIO2, thus potentially requiring transient adjustment of the ventilator settings during nebulization which may add risk to the patient thus continuous jet nebulizer is not a preferred device.

ii. Voting panelists mentioned most of the evidence was from in vitro, they suggested making a clear statement in the introduction to warn readers. Dr. Pelosi suggested: "we should acknowledge that in vivo data is lacking for most of the recommendations, but the in vivo evidence is hard to generate."

iii. A mark of in vivo (human and animal) and in vitro evidence will be placed on each recommendation as well.

- **Dr. Rello:** I emphasized that in vitro papers have to be used as "Proof-of-Concept" but they do not represent high degree evidence. A second level is experimental studies. But anatomy, ventilatory strategy and duration, prone-supine positioning & the pneumonia-model are very different from ventilated humans. Regulatory agencies always require phase II-III studies for approval ! I manifested that evidence should be based on human studies, whereas the other should remain advice that needs to be validated. I disagree that the current version of 2aiii needs to be updated reflecting this important observation.

iv. Voting panelists suggest simplify the statement in recommendations, to make them more concise.

b. 6 recommendations that had not reached consensus were discussed:

i. "When vibrating mesh nebulizer is utilized during invasive ventilation with bias flow, it is recommended to be placed close to the ventilator.": all the voting panelists agreed to change it to very good consensus and make it as a recommendation, but needs to mark that the evidence to support this recommendation is from in vitro studies. Dr. Berlinski suggested changing the statement to "When vibrating mesh nebulizer is utilized during invasive

ventilation with bias flow, in vitro evidence shows that the nebulizer placed close to the ventilator had higher inhaled dose than placed close to the patient". Dr. Lin suggested making a clear definition of bias flow in the text, since various terms are used in different ventilators.

- **Dr. Michotte:** As far as I know, most of invasive ventilators work with bias flow. The problem is that the bias flow setting depends on the manufacturer (or perhaps can be adjusted by the clinician ?) . I would propose to apply this statement for a minimal bias flow setting.

ii. "When jet nebulizer is utilized during invasive ventilation, it is recommended to be placed near the ventilator.": all the voting panelists agreed to change it to very good consensus and make it as a recommendation. They suggested changing the statement to "When jet nebulizer is utilized during invasive ventilation, in vitro evidence shows that the nebulizer placed near the ventilator had higher inhaled dose than placed close to the patient"

iii. "For the jet or ultrasonic nebulizer with a residual volume > 0.5 ml, aerosol delivery efficiency is improved with a higher fill volume, but changing fill volume for the sole purpose of improving aerosol delivery efficiency is not recommended for FDA approved inhaled medication" was suggested to be: "When a nebulizer is utilized, changing fill volume for the sole purpose of improving aerosol delivery efficiency is not recommended" all the voting panelists voted to approve this change.

iv. "For viscous formulations, increasing diluent volume in vibrating mesh nebulizer to improve aerosol delivery efficiency is recommended.": withdraw this recommendation but make a statement in the text

v. "When heliox is substituted for oxygen to drive continuous jet nebulizer at the same driving flow, nebulizer output is reduced. If driving nebulizer with heliox, it is recommended to set gas flow at 15 L/min": was suggested to be changed as: "If operating jet nebulizer with heliox, it is recommended to set gas flow at 15 L/min"

vi. "1-1.15: The efficiency of aerosol delivery in dry ventilator circuits is higher than that in humidified ventilator circuits. Considering the potential harms of

dry gas on patient airway, and the time lapse required for a humidifier and circuits to cool down, turning off humidifier is not recommended for routine aerosol therapy." and suggested revision from Dr. Rouby "The efficiency of bronchodilator and corticosteroids aerosol delivery in dry ventilator circuits is higher than that in humidified ventilator circuits. Considering that a 30 % decrease in the aerosol delivery to the tracheobronchial tree is not associated with a lack of bronchodilator or anti-inflammatory effect, turning off humidifier is not recommended for routine aerosol therapy": all the voting panelists agreed that "turning off the humidifier is not recommended for routine aerosol therapy", and it was unnecessary to explain the rationale in the recommendation. Thus, this recommendation was voted to be placed back to the consensus but changed to" turning off humidifier is not recommended for routine aerosol therapy"

3. Participants reviewed the recommendations for Aerosol delivery via invasive ventilation (antibiotics):
    a. Participants voted to remove the recommendations of changing ventilator settings, due to the lack of research evidence, except for the four publications with voting panel opinions. Thus the following recommendations are withdrawn and will be presented as pros and cons in the text:
        i. "**Recommendation 1-2.4:** When delivering inhaled antibiotics for invasively ventilated patients, it is recommended to keep respiratory rates at 12-15 breaths/min."
        ii. "**Recommendation 1-2.5：** When delivering inhaled antibiotics for invasively ventilated patients, it is recommended to keep inspiratory flow below 40L/min.":
        iii. **"Recommendation 1-2.6:** When delivering inhaled antibiotics for invasively ventilated patients, it is recommended to use inspiratory to expiratory ratio of 50%."
        iv. "**Recommendation 1-2.7:** When delivering inhaled antibiotics for invasively ventilated patients, it is recommended to use a constant inspiratory flow."

v. "**Recommendation 1-2.8:** When delivering inhaled antibiotics for invasively ventilated patients, it is recommended to set end-inspiratory pause at 20%."

vi. "**Recommendation 1-2.9:** When delivering inhaled antibiotics for invasively ventilated patients, it is recommended to set a positive end-expiratory pressure (PEEP) at 5-10 cmH2O"

- **Dr. Rello:** I still feel that recommendations regarding ventilatory settings for inhaled antibiotics in mechanical ventilated patients can not be removed without subsequent action. These observations are very important for inexperienced attendings which is the main target of the recommendations. I mentioned that it is different to aerosolize a bronchodilator in a MV patient (usually takes a short time) than to delay 1-h infusion in a ventilated patients with severe hypoxemia. Again, it is not the same a severe pneumonia that a stable ventilated patient with steroids or beta-adrenergic. Clinical experience indicates that these ventilatory settings are poorly tolerated by patients with severe hypoxemia (eg. PaO2/FiO2 < 100), limiting its use. It is very important to use short-acting agents (e.g. propofol) rather than midazolam or other long-acting agents during aerosolization of antibiotics in these ventilated patients with pneumonia. That is not a secondary issue. It has implications on tolerability, safety and mechanical ventilation duration: As manifested in the teleconference, we cannot address these concerns just with a step-down of these ventilatory settings. Need to provide proactive information for respiratory therapists and intensivists.

b. "**Recommendation 1-2.1:** For antibiotics or other cost-prohibitive medications, changing to a dry circuit immediately before nebulization is recommended." And suggested revision from Dr. Rouby "The efficiency of antibiotic aerosol delivery in dry ventilator circuits is higher than that in humidified ventilator circuits. Therefore, removing HME or turning off the conventional humidifier immediately before nebulization is recommended" were discussed by all the voting panelists:

i. since “removing HME” is recommended for all aerosol therapy, it does not need to mention here.

ii. For “changing to a dry circuit immediately before nebulization is recommended”, **Dr. Lu mentioned that in their published inhaled antibiotics studies, this was their standard practice.** However, a minimum of five voting panelists disagreed to recommend this practice, due to several disadvantages: frequent changes of ventilator circuits (if nebulization is tid, it means the ventilator circuits need to be changed three times a day), which would significantly increase the possibilities of the environmental contamination and VAP, increase the workload for clinicians and the cost. Additionally, it causes alveoli derecruitment. Thus, “changing to a dry circuit” should not be recommended. **Dr. Lu mentioned that in their published inhaled antibiotics studies, they turned off the humidifier when inhaled antibiotics were delivered.**

- **Dr. Rello:** I supported the Dr. LU experience with at least 5 publications endorsing her practice. It was not just "her voting panel opinion". I think that it is needed to split ventilator circuit changes (out of formal recommendation) from the recommendation to use a dry ventilatory strategy (agreed as preferred by all the panel). Whereas changes in dosage arriving to the alveoli are not important for bronchodilators (the target are airways), a reduction in the alveolar space concentration would be a significant risk for organisms with high MICs, which are the indication of inhaled antibiotics. Removing HME is an effective strategy, easy to implement in Eu (represents overall 19/20 MV patients) but more difficult in other regions where convectional humidifiers are often predominant. Need to be addressed.

iii. For “turning off the conventional humidifier immediately before nebulization”: Dr. Lin mentioned in her in vitro study that nebulization within 1 hour of turning off the conventional humidifier would not improve aerosol delivery, but turning off the humidifier and then waiting one hour to deliver

aerosol is not practical, due to busy clinical workload, not saying clinicians may forget to turn the humidification back on after the aerosol delivery. Dr. Fink mentioned the characteristics of antibiotics are not different from other medications during nebulization, thus it is unnecessary to treat inhaled antibiotics differently. Finally, no consensus can be reached. All voting panelists agreed to withdraw it and present it as pros and cons in the text.

4. Participants reviewed the recommendations for Aerosol Delivery via Non-invasive Ventilation:
    a. 4 reached perfect consensus, 6 reached very good consensus, 1 withdrawn
5. Participants reviewed the recommendations for Aerosol Delivery via High-flow Nasal Cannula
    a. 2 reached perfect consensus, 5 reached very good consensus, 3 withdrawn

Appendix 9

Nebulization consensus on aerosol delivery for adult critically ill patients

Online meeting minutes

Meeting date and time: 8-10 am CST, Nov 3rd, 2022

Meeting attendee: Antoni Torres, Jordi Rello, Pelosi Paolo, Stephan Ehrmann, Rajiv Dhand, Charles-Edouard Luyt, Huiling Lin, Ariel Berlinski, Jean-bernard Michott, Jean-Jacques Rouby, James B Fink, Bing Dai, Shan Lv, Kai Liu, Guoqiang Jing, Jie Li

1. Updates on the in vitro studies of the effects of humidification on aerosol delivery during invasive mechanical ventilation: presented by Drs Ehrmann, Lin, Li, and Pelosi
    a. Dr. Rouby:
        i. The inhaled antibiotics data provided Dr. Ehrmann is convincing, more importantly, it provides the evidence that the humidity did not significantly change the aerosol particle size measured as MMAD, which plays a key role in aerosol deposition, especially for inhaled antibiotics that need to be deposited in peripheral airways or alveoli.
        ii. The in vitro studies did not find any higher deposition with dry circuits, and no better aerosol delivery after the humidifier was turned off.
        iii. Agreement on the change recommendations on turning off humidifier for inhaled antibiotics during invasive ventilation.
    b. Dr. Li:
        i. In our original and current recommendation of humidification section in the consensus, we state "*turning off the humidifier is not recommended for routine*

aerosol therapy", thus no need to change the recommendation in the current consensus.

ii. Moreover, the four studies are great examples to demonstrate the importance of our consensus for identifying knowledge gaps and guiding future research.

iii. These studies were conducted to address the gaps of information on the effects of humidification during the production of the consensus.

c. Dr. Ehrmann:

i. although we saw a small improvement of aerosol delivery with humidifier on when a mesh nebulizer was placed at the inlet of humidifier, I'd suggest we emphasize that the aerosol deposition was not significantly different with humidifier on and off, since this is hard to explain.

ii. It is important that our recommendations are clear and provide a simple approach to aerosol administration.

d. Dr. Rello:

i. Reached out to editorial leadership at the blue journal and they expressed some interest.

ii. Recommended submitting the consensus to blue journal but need to check with Dr. Brochard before submission.

iii. For the four in vitro studies, I suggested submit them to journal *antibiotics* with an ongoing special issue, which suits the paper.

e. Dr. Torres:

i. Had a question regarding the proximal vs distal delivery in the lung. As we know, inhaled antibiotics should be delivered to the distal in the lung to treat pneumonia.

f. Dr. Dhand:

i. Several animal studies reported that antibiotics could be delivered to the distal area in the lung.

ii. In a study with patients receiving pneumonectomy, 4 puffs albuterol via MDI was administered before removing a lung, we detected high-concentrations of albuterol in the distal lung tissue.

g. Dr. Fink:

i. Working with Drs Ehrmann, Lin, and Li, to see if we can combine the in vitro studies into one paper. If we can put all four studies together and submit it to one journal, it might increase the impact of the findings.

ii. Dr. Pelosi offered to share his study data for the combined paper.

h. Brief discussion on muco-ciliary impact of humidification and nebulization: as this is not strictly related nebulization technique which in general last less than one hour, general agreement to suppress this part

2. Dr. Rello proposed to separate the consensus into two consensus: for non-antibiotics and for antibiotics.

a. Dr. Rouby: I agree. Since inhaled antibiotics need to be delivered to terminal airways and alveoli, in order to treat pneumonia. Inhaled antibiotics are difficult to deliver and have to be maximized aerosol delivery, for example, inhaled antibiotics have to be maintained at certain MMAD in order to reach the target organ and the concentrations

need to be high in lung parenchyma. In contrast, for bronchodilators, mucolytics, or steroids, they are easy to deliver and their target organ is bronchial. Ventilator settings do not need to be adjusted for non-antibiotics, but the ventilator settings need to be maximized to improve the antibiotics in the concentrations in lung parenchyma.

b. Dr. Fink proposed proceeding with the current consensus saving antibiotics for a separate paper when additional information is available, with statement in the introduction that antibiotics are not within the scope of the consensus.

c. Dr. Ehrmann:

    i. Not keen to have two consensus, as it is against simplicity. Two consensus on delivery antibiotics and non-antibiotics, especially with different recommendations, are difficult for clinicians to follow, as they might not clearly remember which recommendations applies to which drug.

    ii. We can say delivery inhaled antibiotics to alveoli level is not easy, but delivering inhaled antibiotics to bronchi are as easy as other medications. Indeed, we do have patients mixed with pneumonia and bronchiolitis. In my previous international survey and others' surveys, we saw people used inhaled antibiotics to treat bronchiolitis as well as colonization, using inhaled antibiotics to treat pneumonia is not frequently use of inhaled antibiotics. there is no clear cut between antibiotics and non-antibiotics. thus I disagreed to separate the consensus into antibiotics and non-antibiotics. I would provide the consensus as simple as possible.

d. Dr. Lyut: I agree not to separate, the results of all the RCTs of inhaled antibiotics are negative. I agree with Stephan that two consensus would cause confusion for clinicians.

e. Dr. Lin: I support not to separate. Also, the current consensus is made based on the studies from in vitro, animal, and clinical using bronchodilators, inhaled antibiotics, etc, if we want to separate the consensus, we need to remove all the studies that used inhaled antibiotics, which might change the content of the consensus.

f. Dr. Fink:

   i. Published research show no differences in the aerodynamics of inhaled antibiotics and inhaled.

   ii. Clinical evidence is lacking on changing the ventilator settings to improve the delivery of inhaled antibiotics to the terminal airways and alveoli levels.

   iii. This is also true for surfactants, prostacyclin, etc. We do not need to make a consensus on each drug.

   iv. Our consensus focuses on the aerosol delivery technics, rather than the specific medications.

g. Dr. Torres:

   i. I don't think the clinicians with infectious disease would not agree with the consensus if we don't separate the consensus. And I think we should suggest change the title to be non-antibiotics.

h. Dr. Berlinski:

   i. The only antibiotics approved for inhalation are for treatment of ambulatory patients with cystic fibrosis. Also, inhaled medication has been approved for

use during mechanical ventilation. Thus, technically all the aerosol delivery via invasive ventilation is off-label use.

ii. He also commented that one document would serve better.

i. Dr. Pelosi:

i. Agreed with both sides and understand Stephan's points of using inhaled antibiotics to treat bronchiolitis is at the bronchial level.

ii. Suggested we keep one consensus, but write a paragraph to describe the delivery of inhaled antibiotics, specifically the importance of delivering aerosols to alveoli level if we aim to treat pneumonia. It does not need to be long, can be a brief section. I think it is the best compromise to make everything together.

j. Dr. Rello:

i. I want to point out that inhaled antibiotics should not be used to treat colonization.

ii. I have seen several incorrect practice of using inhaled antibiotics in many other countries (I have seen clinicians used TOBI (not necessarily formulations approved for inhalation) to treat all patients with artificial airways). Thus it is important for clinicians to understand the correct indication.

iii. The clinical practice of using aerosolized antibiotics as well as the systemic use of antibiotics vary greatly in the previous RCTs, is probably why those studies did not find significant differences. I think if we can state the importance and difficulty of delivering aerosolized antibiotics to treat

pneumonia, and clearly state delivering inhaled antibiotics to mechanically ventilated patients is off-label use, it is possible we can reach the consensus.

iv. There is general consensus to write in the recommendation that the group does not endorse unvalidated practice such as treating colonization systematically and that the consensus aims at giving technical guidance on how to perform aerosol therapy without giving guidance on indications.

v. This information needs to be put in the main text, not in the appendix.

k. Dr. Berlinski:

i. I think we should stay away from inhaled antibiotics, since there are many other drugs that need to be delivered to the alveoli level, such as prostacyclin to treat pulmonary hypertension, or surfactant. We should not focus on one medication. Plus, all the inhaled antibiotics for mechanically ventilated are not approved.

3. Summary

a. No call to change the current recommendations

b. Agreed for single Consensus paper (antibiotics and non-antibiotics),

c. Dr. Berlinski will draft a short paragraph regarding off-label use and the role of consensus to identify best methods and not endorse off label use of any medication not approved for inhalation.

d. Dr. Rouby will draft a brief paragraph regarding the delivery of inhaled antibiotics to treat pneumonia

e. Dr. Li will finalize the manuscript of the consensus and post it on google drive for all the voting panelists to review/edit/comment/approve prior to submission to the blue journal, which is the first target journal.

Appendix 10

Additional information and results from the consensus

1. Additional results from the consensus:
   1.1. **USN vs VMN**: When comparing VMN with a USN via IMV, similar inhaled doses were reported with nebulizers placed at the inspiratory limb before Y-piece.[1-4] However, when they were placed close to the ventilator, a higher inhaled dose was found with VMN than USN.[1,3]
   1.2. **USN vs continuous JN**: While comparing USN with continuous JN via IMV, a higher inhaled dose was found with USN than continuous JN [1,5] while one in vitro study[6] did not report significant differences.
   1.3. **Inspiratory-synchronized vs continuous JN:** Not all ventilators have an option for inspiration-synchronized JN, which is driven by part of the gas that is set for the patient, thus it does not affect ventilator delivery. One in vitro study reported a higher inhaled dose with inspiration-synchronized JN than pMDI with spacer.[7] When the nebulizer was placed at 12 inch (30.5 cm) from Y-piece in the inspiratory limb, inspiration-synchronized JN had a higher inhaled dose than with continuous JN.[8] However, when the nebulizer was placed close to the ventilator, no significant differences were found in the inhaled dose.[9] Additionally, the time used for administering aerosol via inspiration-synchronized JN was 2.5 times of the continuous JN.[9]
   1.4. **USN placement:** The findings regarding the placements of USN are contradictory. When there was no bias flow, an in vitro study reported a higher inhaled dose with USN placed close to the patient than close to the ventilator, particularly in the inspiratory limb 15cm from Y-piece.[1] However, a clinical study reported a similar pulmonary bioavailability between the two positions.[10] While in an in vitro study with a bias flow setting, Parker et al reported a higher inhaled dose with USN placed close to the ventilator than in the inspiratory limb before Y-piece.[3] In contrast, two other in vitro studies with unknown settings on bias flow reported a similar inhaled dose with different placements of USN.[6,11] As such, no recommendation could be generated regarding USN placements.
   1.5. **Filtered HME vs non-filtered HME**: Clinically, there are two types of HMEs: nonfilter and filter HMEs. Nonfilter HME was shown to reduce aerosol deposition by

40% with both JN and VMN, compared to no HME. While the filtered HMEs reduced the delivered dose to <0.5%.[12]

1.6. **Adding an extension tubing to improve aerosol delivery via NIV.** When nebulizer was placed close to the patient, adding an extension tubing from nebulizer to the exhalation valve helped improve aerosol delivery. However, adding the extension tubing may increase dead space, especially when it was added between the mask and exhalation valve. Therefore, to simplify the set-up, the continuous nebulizers are recommended to be placed between the mask and the exhalation valve.

1.7. **Heliox vs oxygen during aerosol delivery via HFNC:** an in vitro study reported that the inhaled dose in aerosol delivery via HFNC with heliox was higher than that with oxygen when HFNC gas flow was ≥30 L/min, but the difference did not reach significance.[13] Additionally, considering the cost of using heliox at such a high flow, it is not cost-effective to use heliox for the sole purpose of improving aerosol delivery.

1.8. **Aerosol delivery via different HFNC devices.** Some HFNC devices might create a high velocity of the gas, which might create a more turbulent flow, reducing the aerosol delivery. HiVNI was reported to deliver <2% of the inhaled dose in an in vitro study with an adult manikin, regardless of nebulizer placement and HFNC flow settings (20-40 L/min).[14]

1.9. ***HFNC gas flow.*** During quiet breathing, the inhaled dose increased as HFNC gas flows decreased. [13,15-19] . However, the inhaled dose at 20-30 L/min was higher or similar to the inhaled dose at 10 L/min during distressed breathing.[13,17-19] As such, the ratio of HFNC gas flow to patient peak inspiratory flow rather than each flow alone was proposed to play a key role in aerosol delivery via HFNC,[17] and the inhaled dose was optimal when the HFNC gas flow was set at 50% of patient peak inspiratory flow.[17] No device is commercially available to measure patient peak inspiratory flow breath by breath, thus titrating gas flow according to patient response to the inhaled medication might be a pragmatic solution, especially for the inhaled medication with quick onset such as prostacyclin.[20] Furthermore, median peak inspiratory flow during tidal breathing among patients with acute hypoxemic respiratory failure was 31 (27,42) L/min,[21] implying that if tolerable, HFNC flow set at 20 L/min might be reasonable during aerosol delivery for adult critically ill patients.[22] Using a high concentrated solution

might help shorten the flow reduction periods and minimize the potential harms of flow reduction.[22]

References

1. Ari A, Areabi H, Fink JB. Evaluation of aerosol generator devices at 3 locations in humidified and non-humidified circuits during adult mechanical ventilation. *Respir Care.* 2010;55(7):837-844.
2. Pedersen KM, Handlos VN, Heslet L, Kristensen HG. Factors influencing the in vitro deposition of tobramycin aerosol: a comparison of an ultrasonic nebulizer and a high-frequency vibrating mesh nebulizer. *J Aerosol Med.* 2006;19(2):175-183.
3. Parker DK, Shen S, Zheng J, et al. Inhaled Treprostinil Drug Delivery During Mechanical Ventilation and Spontaneous Breathing Using Two Different Nebulizers. *Pediatr Crit Care Med.* 2017;18(6):e253-e260.
4. Ferrari F, Liu ZH, Lu Q, et al. Comparison of lung tissue concentrations of nebulized ceftazidime in ventilated piglets: ultrasonic versus vibrating plate nebulizers. *Intensive Care Med.* 2008;34(9):1718-1723.
5. Harvey CJ, O'Doherty MJ, Page CJ, Thomas SH, Nunan TO, Treacher DF. Comparison of jet and ultrasonic nebulizer pulmonary aerosol deposition during mechanical ventilation. *Eur Respir J.* 1997;10(4):905-909.
6. O'Doherty MJ, Thomas SH, Page CJ, Treacher DF, Nunan TO. Delivery of a nebulized aerosol to a lung model during mechanical ventilation. Effect of ventilator settings and nebulizer type, position, and volume of fill. *Am Rev Respir Dis.* 1992;146(2):383-388.
7. Diot P, Morra L, Smaldone GC. Albuterol delivery in a model of mechanical ventilation. Comparison of metered-dose inhaler and nebulizer efficiency. *Am J Respir Crit Care Med.* 1995;152(4 Pt 1):1391-1394.
8. Miller DD, Amin MM, Palmer LB, Shah AR, Smaldone GC. Aerosol delivery and modern mechanical ventilation: in vitro/in vivo evaluation. *Am J Respir Crit Care Med.* 2003;168(10):1205-1209.
9. Wan GH, Lin HL, Fink JB, et al. In vitro evaluation of aerosol delivery by different nebulization modes in pediatric and adult mechanical ventilators. *Respir Care.* 2014;59(10):1494-1500.
10. Moraine JJ, Truflandier K, Vandenbergen N, Berre J, Melot C, Vincent JL. Placement of the nebulizer before the humidifier during mechanical ventilation: Effect on aerosol delivery. *Heart Lung.* 2009;38(5):435-439.

11. Thomas SH, O'Doherty MJ, Page CJ, Treacher DF, Nunan TO. Delivery of ultrasonic nebulized aerosols to a lung model during mechanical ventilation. *Am Rev Respir Dis.* 1993;148(4):872-877.
12. Ari A, Dang T, Al Enazi FH, et al. Effect of Heat Moisture Exchanger on Aerosol Drug Delivery and Airway Resistance in Simulated Ventilator-Dependent Adults Using Jet and Mesh Nebulizers. *J Aerosol Med Pulm Drug Deliv.* 2018;31(1):42-48.
13. Dailey PA, Harwood R, Walsh K, et al. Aerosol Delivery Through Adult High Flow Nasal Cannula With Heliox and Oxygen. *Respir Care.* 2017;62(9):1186-1192.
14. Bennett G, Joyce M, Fernandez EF, MacLoughlin R. Comparison of aerosol delivery across combinations of drug delivery interfaces with and without concurrent high-flow nasal therapy. *Intensive Care Med Exp.* 2019;7(1):20.
15. Reminiac F, Vecellio L, Heuze-Vourc'h N, et al. Aerosol Therapy in Adults Receiving High Flow Nasal Cannula Oxygen Therapy. *J Aerosol Med Pulm Drug Deliv.* 2016;29(2):134-141.
16. McGrath JA, O'Toole C, Bennett G, Joyce M, Byrne MA, MacLoughlin R. Investigation of Fugitive Aerosols Released into the Environment during High-Flow Therapy. *Pharmaceutics.* 2019;11(6):254.
17. Li J, Gong L, Fink JB. The Ratio of Nasal Cannula Gas Flow to Patient Inspiratory Flow on Trans-nasal Pulmonary Aerosol Delivery for Adults: An in Vitro Study. *Pharmaceutics.* 2019;11(5):225.
18. Li J, Wu W, Fink JB. In vitro comparison between inspiration synchronized and continuous vibrating mesh nebulizer during trans-nasal aerosol delivery. *Intensive Care Med Exp.* 2020;8(1):6.
19. Li J, Williams L, Fink JB. The Impact of High-Flow Nasal Cannula Device, Nebulizer Type, and Placement on Trans-Nasal Aerosol Drug Delivery: An In Vitro Study. *Respir Care.* 2021:1-8.
20. Li J, Gurnani PK, Roberts KM, Fink JB, Vines D. The Clinical Impact of Flow Titration on Epoprostenol Delivery via High Flow Nasal Cannula for ICU Patients with Pulmonary Hypertension or Right Ventricular Dysfunction: A Retrospective Cohort Comparison Study. *J Clin Med.* 2020;9(2):464.

21. Li J, Scott JB, Fink JB, Reed B, Roca O, Dhand R. Optimizing high-flow nasal cannula flow settings in adult hypoxemic patients based on peak inspiratory flow during tidal breathing. *Ann Intensive Care.* 2021;11(1):164.

22. Li J, Fink JB, MacLoughlin R, Dhand R. A narrative review on trans-nasal pulmonary aerosol delivery. *Crit Care.* 2020;24(1):506.

Appendix 11

Pros and cons of changing ventilator settings and humidifiers when inhaled antibiotics

Recommendation	Pros	Cons
For antibiotics or other cost-prohibitive medications, changing to a dry circuit immediately before nebulization is recommended.	• It is not the same a severe pneumonia that a stable ventilated patient with steroids or beta-adrenergic<br>• It's important to maximize aerosol delivery efficiency when we use cost-prohibitive medications<br>• Turning off the humidifier during the nebulization phase rather than changing the circuits for dry circuits appears as a simple and easy to implement option to provide high lung deposition of aerosolized antibiotics.	• More clinical data are needed<br>• Repeated disconnection and reconnection of ventilatory circuits can induce alveolar de-recruitment;<br>• Changing the circuits increase the workload for clinicians and the cost<br>• 1 hour of turning off the conventional humidifier would not improve aerosol delivery<br>• Turning off the humidifier and then waiting one hour to deliver aerosol is not practical<br>• The characteristics of antibiotics are not different from other medications during nebulization
When delivering inhaled antibiotics for invasively ventilated patients, spontaneous breathing ventilator modes may reduce aerosol delivery efficiency, thus spontaneous breathing should be avoided and volume-controlled mode is preferred, and assessing overall benefit/risk ratio especially related to sedation.	• It's important to maximize aerosol delivery efficiency when we use cost-prohibitive medications<br>• Inspiratory flow turbulences resulting from spontaneous breathing, may reduce aerosol delivery efficiency<br>• With optimized sedation with propofol.	• More clinical data are needed<br>• Weaning from mechanical ventilation should take priority over nebulization
When delivering inhaled antibiotics for invasively ventilated patients, it is recommended to set tidal volume of 8ml/kg of patient's predicted body weight, and the clinician must weigh the benefit/risk ratio of increasing tidal volume for improving aerosol delivery with the risk of high tidal volume.	• It's important to maximize aerosol delivery efficiency when we use cost-prohibitive medications<br>• 8ml/kg TV for 1 hour is not a risk for ventilator-induced lung injury.	• More clinical data are needed<br>• Increasing tidal volume may have adverse effects<br>• If the patients have severe ARDS this is not acceptable.
When delivering inhaled antibiotics for invasively ventilated patients, it is recommended to keep respiratory rates at 12-15 breaths/min	• Lower respiratory rates improve aerosol delivery	• More clinical data are needed<br>• It might be risky to set such low respiratory rates for patients who have hypercapnic, and may induce

		intracranial hypertension<br>• It is hard to control respiratory rate unless sedate or even paralyze the patients, which may delay MV weaning.<br>• It depends on the I:E ratio
When delivering inhaled antibiotics for invasively ventilated patients, it is recommended to keep inspiratory flow below 40L/min.	• Decreasing the inspiratory flow improves aerosol delivery	• More clinical data are needed<br>• Potential risk for the patient<br>• It is hard to control inspiratory flow unless sedate or even paralyze the patients.<br>• The potential risks for patient
When delivering inhaled antibiotics for invasively ventilated patients, it is recommended to use inspiratory to expiratory ratio of 50%.	• Lengthening inspiration time and using low constant inspiratory flows to decrease inspiratory flow turbulences reduce the impaction of aerosolized particles in ventilator circuits and bronchi and promote lung parenchymal deposition.	• Induce auto PEEP and barotrauma in patients with obstructive disease<br>• Need increasing sedation<br>• Inspiratory to expiratory ratio is only a (complex) indirect way to set the inspiratory flow.<br>• Only tolerated with stiff lungs and severe ARDS.
When delivering inhaled antibiotics for invasively ventilated patients, it is recommended to use a constant inspiratory flow.	• Lengthening inspiration time and using low constant inspiratory flows to decrease inspiratory flow turbulences reduce the impaction of aerosolized particles in ventilator circuits and bronchi and promote lung parenchymal deposition.	• More clinical data are needed<br>• It is difficult to control the inspiratory flow pattern unless patients are sedated.<br>• A constant flow can only be applied when the patient is in a controlled ventilatory mode
When delivering inhaled antibiotics for invasively ventilated patients, it is recommended to set end-inspiratory pause at 20%.	• Volume controlled ventilation is rarely used without an end-inspiratory pause.<br>• End-inspiratory pause improves aerosol delivery of aerosolized antibiotics	• More clinical data are needed<br>• More dangerous potential for Cardiovascular insult
When delivering inhaled antibiotics for invasively ventilated patients, it is recommended to set a positive end-expiratory pressure (PEEP) at 5-10 cmH2O	• PEEP implementation during nebulization improves aerosol delivery of aerosolized antibiotics by recruiting the infected lung	• More clinical data are needed<br>• The priority of the PEEP setting should remain the ventilation<br>• The clinician must first weigh the benefit/risk ratio of increasing a PEEP level (especially high level)<br>• No significant differences of aerosol deposition with PEEP at 0-10 cmH2O

RCT Studies of inhaled antibiotics

Author, year	Design	Population	Intervention	Delivery device and Position	Ventilation setting	Outcome
Niederman MS, 2012	Multicenter, randomized, double-blind, placebo-controlled, phase II study	Adults, mechanical ventilation > 3 days, clinical diagnosis of GNB HAP, VAP or HCAP	Inhaled amikacin 400 mg bid (n=21), inhaled amikacin 400 mg daily (n=26), inhaled placebo (n=22) for 7~14 days; standard of care systemic antibiotics	VMN with PDDS system, between ETT and Y connector, or hand-held	Ventilator settings were not mentioned<br>Humidity was not mentioned	The primary endpoint was achieved in 50% (6/12) and 16.7% (3/18) of patients in the q12h and q24h groups (p = 0.102), respectively. Clinical cure rates, in the 48 patients getting C7 days of therapy, were 93.8% (15/ 16), 75.0% (12/16), and 87.5% (14/ 16) in the q12h, q24h, and placebo groups, respectively (p = 0.467). By the end of aerosol therapy, the mean number of antibiotics per patient per day was 0.9 in the q12h, 1.3 in the q24h, and 1.9 in the placebo groups, respectively (p = 0.02 for difference between groups).
Kollef MH, 2017 (IASIS)	Multicenter, randomized, double-blind, placebo-controlled, phase II study	GNB VAP	Inhaled amikacin 300 mg/Fosfomycin 120 mg bid (n=71) vs. Placebo (n=72) for 10 days, with intravenous meropenem or imipenem	Continuously VMN, proximal to Y connector	Bais flow <4 L/min<br>Ventilator settings were not changed<br>Nebulizer was left in place during the treatment period<br>Humidity was maintained during treatment	No difference in CPIS during therapy (p=0.7), no mortality and clinical cure at Day 14 or earlier was also not significant (P=0.68) nor the hierarchical endpoint of no mortality and ventilator free days (P=0.06), mortality was 17 (24%) vs 12 (17%), (P=0.32), higher rate of negative tracheal cultures form gram-negatives at day 3 and 7m with aerosol therapy

Hassan NA, 2018	Single center randomized, controlled, not blinded	Post cardiac surgery adult patients with VAP or HAP caused by MDR GNB	Inhaled amikacin 300 mg/fosfomycin 120 mg bid (n =71) vs. Placebo (n=72) for 10 days, with intravenous meropenem or imipenem	JN for ventilated patients, UN for non-ventilated patients	Ventilator settings were not mentioned Humidity was not mentioned	Nebulized amikacin showed less ICU stay (p=0.010), fewer days to reach complete recovery (p=0.001), fewer days on mechanical ventilator (p = 0.035), and fewer days on amikacin treatment (p=0.022), less nephrotoxic (p <0.001) compared to IV amikacin
Niederman MS, 2020 (INHALE)	Multicenter, prospective, double-blind, randomized, placebo-controlled, phase III study	Adult, MDR GNB pneumonia, intubated and mechanically ventilated, impaired oxygenation within 48 h; modified CPIS at least 6	Inhaled amikacin 400 mg bid (n=354) vs. Inhaled placebo (n=358) for 10 days; standard of care systemic antibiotics	VMN with PDDS system, between ETT and Y connector, or hand-held	Remove HME Ventilator settings were not mentioned Humidity was not mentioned	Similar proportions of patients survived until days 28–32 (191 (75%) vs 196 (77%), odds ratio 0·841, 95% CI 0·554–1·277; p=0·43) and similar treatment-emergent adverse event (295 [84%] vs 303 [84%]) or a serious treatment-emergent adverse event (101 [29%] vs 97 [27%])
Stokker J,2020 (VAPORISE)	Single-center, randomized, double-blind, placebo-controlled study	VAP	Inhaled tobramycin inhalation 300 mg bid (n=13) vs inhaled placebo (n=13) for 8 days; standard intravenous antibiotic	Continuously VMN	Ventilator settings were not mentioned Humidity was not mentioned	The study was terminated prematurely due to insufficient inclusion. Treatment failure was present in four patients (31%) of the treatment group and in eight control patients (62%) (p=0.24, relative risk=0.5). There was no difference in 30-day mortality (treatment, n=4 (31%) vs control, n=4 (31%). The number of ventilation free days at day 28 was 18 days [0–21] in the treatment group and 17 days [5–22] in the control group.

GNB Gram-negative bacilli, HAP hospital-acquired pneumonia, VAP ventilator-associated pneumonia, HCAP healthcare-associated pneumonia, MDR multidrug resistant, VMN vibrating mesh nebulizer, PDDS pulmonary drug delivery system, JN jet nebulizer, UN ultrasonic nebulizer, MV minute ventilation, Vt tidal volume, I: E inspiratory to expiratory ratio, RR respiratory rate, PEEP positive end-expiration pressure, HME heat and moisture exchanger, HH heated humidifiers, CPIS clinical pulmonary infection score, ETT

endotracheal tube

1. Niederman MS, Chastre J, Corkery K, Fink JB, Luyt CE, García MS. BAY41-6551 achieves bactericidal tracheal aspirate amikacin concentrations in mechanically ventilated patients with Gram-negative pneumonia. Intensive Care Med. 2012 Feb;38(2):263-71.
2. Kollef MH, Ricard JD, Roux D, Francois B, Ischaki E, Rozgonyi Z, Boulain T, Ivanyi Z, János G, Garot D, Koura F, Zakynthinos E, Dimopoulos G, Torres A, Danker W, Montgomery AB. A Randomized Trial of the Amikacin Fosfomycin Inhalation System for the Adjunctive Therapy of Gram-Negative Ventilator-Associated Pneumonia: IASIS Trial. Chest. 2017 Jun;151(6):1239-1246.
3. Hassan NA, Awdallah FF, Abbassi MM, Sabry NA. Nebulized Versus IV Amikacin as Adjunctive Antibiotic for Hospital and Ventilator-Acquired Pneumonia Postcardiac Surgeries: A Randomized Controlled Trial. Crit Care Med. 2018 Jan;46(1):45-52.
4. Niederman MS, Alder J, Bassetti M, Boateng F, Cao B, Corkery K, Dhand R, Kaye KS, Lawatscheck R, McLeroth P, Nicolau DP, Wang C, Wood GC, Wunderink RG, Chastre J. Inhaled amikacin adjunctive to intravenous standard-of-care antibiotics in mechanically ventilated patients with Gram-negative pneumonia (INHALE): a double-blind, randomised, placebo-controlled, phase 3, superiority trial. Lancet Infect Dis. 2020 Mar;20(3):330-340.
5. Stokker J, Karami M, Hoek R, Gommers D, van der Eerden M. Effect of adjunctive tobramycin inhalation versus placebo on early clinical response in the treatment of ventilator-associated pneumonia: the VAPORISE randomized-controlled trial. Intensive Care Med. 2020 Mar;46(3):546-548.

Recommendations for nebulized antibiotics in the consensuses and reviews.

Author, year	Nebulizer	Position	Mode	Vt	Inspiratory flow	I: E	RR	Bias flow	End-inspiratory pause	PEEP	Others
Rello J,2017	VMN	10–15 cm before the Y piece on the inspiratory limb	Volume-controlled	8ml/kg	Constant inspiratory flow	≤ 50%	12-15 bpm	/	20%	5-10 cmH_2O	• Avoid sharp angles and rough inner surfaces in circuit<br>• Avoid asynchronies and triggering<br>• Increase level of sedation if necessary<br>• Remove HME<br>• Turn off HH<br>• Change expiratory filter after each nebulization
Rouby JJ, 2020	VMN	10–15 cm before the Y piece on the inspiratory limb	Volume-controlled	8ml/kg	Limiting inspiratory flow velocity	1: 2	12-15 bpm	/	20%	5-10 cmH_2O	• Remove HME<br>• Turn off HH
Monsel A, 2021	VMN	15 cm before the Y piece on the inspiratory limb	Volume-controlled	8ml/kg	Constant inspiratory flow	1: 1	12-15 bpm	Absence or minimum (2 l/min)	20%	/	• Smooth angles and inner surface tube<br>• Increase level of sedation if necessary<br>• Remove HME<br>• Turn off HH

VMN vibrating mesh nebulizer, PDDS pulmonary drug delivery system, JN jet nebulizer, UN ultrasonic nebulizer, MV minute ventilation, Vt tidal volume, I: E inspiratory to expiratory ratio, RR respiratory rate, PEEP positive end-expiration pressure, HME heat and moisture exchanger, HH heated humidifiers,

1. Rello J, Rouby JJ, Sole-Lleonart C, Chastre J, Blot S, Luyt CE, Riera J, Vos MC, Monsel A, Dhanani J, Roberts JA. Key considerations on nebulization of antimicrobial agents to mechanically ventilated patients. Clin Microbiol Infect. 2017 Sep;23(9):640-646.
2. Rouby JJ, Sole-Lleonart C, Rello J; European Investigators Network for Nebulized Antibiotics in Ventilator-associated Pneumonia. Ventilator-associated pneumonia caused by multidrug-resistant Gram-negative bacteria: understanding nebulization of aminoglycosides and colistin. Intensive Care Med. 2020 Apr;46(4):766-770.
3. Monsel A, Torres A, Zhu Y, Pugin J, Rello J, Rouby JJ; European Investigators Network for Nebulized Antibiotics in Ventilator-associated Pneumonia (ENAVAP). Nebulized

antibiotics for ventilator-associated pneumonia: methodological framework for future multicenter randomized controlled trials. Curr Opin Infect Dis. 2021 Apr 1;34(2):156-168.
